# Iron‐Photocatalyzed C(sp^3^)–H Phosphonylation of Alkanes

**DOI:** 10.1002/anie.6650940

**Published:** 2026-05-05

**Authors:** Ya Dong, Wangyujing Han, Hanwen Zhang, Santosh K. Pagire, Harry Meats, Adam Noble, Varinder K. Aggarwal

**Affiliations:** ^1^ School of Chemistry University of Bristol Cantock's Close Bristol UK

**Keywords:** C(sp^3^)–H functionalization, hydrogen atom transfer, ligand‐to‐metal charge transfer, phosphonylation, photocatalysis

## Abstract

Alkyl phosphonate esters are valuable motifs that have broad synthetic utility and are commonly found in bioactive molecules. Therefore, many methods have been developed to enable efficient phosphonylations of organic molecules, typically involving the construction of C(sp^3^)–P bonds by substitutions of common functional groups with phosphorus(III) reagents. However, direct phosphonylations of unactivated C(sp^3^)–H bonds are rare and currently lack the substrate generality required for late‐stage introduction of phosphonate groups into complex molecules. Herein, we report a photoinduced C(sp^3^)–H phosphonylation of unactivated alkanes using a hydrogen atom transfer (HAT) strategy with an iron photocatalyst. Key to the success of the process was the development of a novel mandelonitrile‐derived phosphite radical trap, which is equipped with an oxidizing phenylacetonitrile radical leaving group that enables effective turnover of the photocatalyst. The method displays good functional group tolerance, high selectivity for phosphonylations of sterically unhindered C─H bonds, and was found to be applicable to regioselective late‐stage installation of phosphonate esters into complex molecules.

## Graphical abstract

Alkyl phosphonates are valuable motifs that can be prepared by phosphonylations of various common functional groups. However, direct phosphonylations of C(sp^3^)–H bonds are limited to activated positions. Herein, we report an iron‐photocatalyzed phosphonylation of unactivated C(sp^3^)–H bonds using a novel phosphite radical trap, which is equipped with an oxidizing radical leaving group to enable effective photocatalyst turnover.

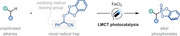

## 1 Introduction

**SCHEME 1:**
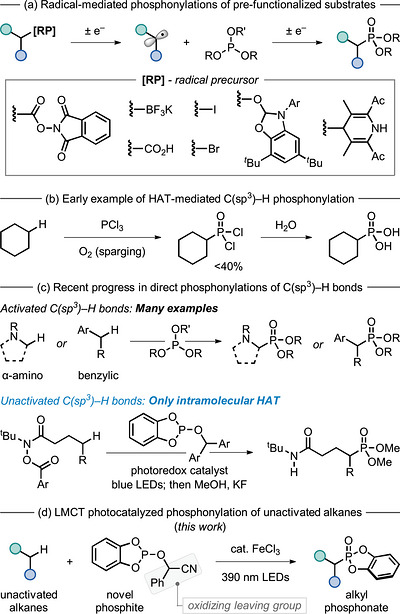
Alkyl phosphonates by C(sp^3^)–P bond formation from alkyl radicals.

Alkyl phosphonic acids and esters have widespread applications in the chemical sciences, including as reagents for organic synthesis and key motifs in materials, pharmaceuticals, and agrochemicals [1, 2, 3, 4]. Their importance has led to the development of numerous methods to introduce phosphonate groups into organic molecules through C(sp^3^)–P bond formation [1, 5, 6, 7]. These commonly involve reactions of nucleophilic phosphorus(III) reagents with electrophilic carbon centers, such as alkyl halides or carbonyls [8, 9, 10, 11, 12], or reactions of organometallic species with phosphorus(V) electrophiles [13]. However, there has been a growing interest in radical‐mediated alternatives, wherein C(sp^3^)–P bonds are constructed by phosphonylation of alkyl radical intermediates [14, 15, 16], either through direct radical addition to form phosphoranyl radical intermediates [17, 18, 19, 20, 21, 22, 23, 24, 25, 26], transition metal‐catalyzed cross‐coupling [27, 28, 29], or radical–polar crossover [30, 31, 32]. These methods provide opportunities to broaden the range of accessible alkyl phosphonate products by substitution of diverse alkyl radical precursors that cannot engage in traditional polar phosphonylation chemistry, including carboxylic acids [17, 18, 19, 20, 21, 27, 28, 30, 31, 32], trifluoroborate salts [19], alkyl halides [22, 29], alcohols [23], and aldehydes (Scheme 1a) [24].

While radical‐mediated phosphonylations have seen significant developments over the last few years, most of these rely on pre‐functionalized substrates [17, 18, 19, 20, 21, 22, 23, 24, 25, 26, 27, 28, 29, 30, 31, 32]. In contrast, the use of hydrogen atom transfer (HAT) to generate alkyl radicals for subsequent phosphonylation is underdeveloped, yet such an approach would allow the introduction of phosphonate groups onto unfunctionalized and typically unreactive feedstock alkanes through substitution of strong C(sp^3^)–H bonds [33, 34]. HAT‐mediated synthesis of alkyl phosphonates was first reported over 75 years ago, where simple alkanes reacted with PCl_3_ under an O_2_ atmosphere to form low yields of alkyl‐dichlorophosphonates, which readily hydrolyzed to phosphonic acids (Scheme 1b) [35, 36, 37]. However, there has been little progress in the development of more general phosphonylations of unactivated C(sp^3^)–H bonds [38]. Recent methods have provided milder conditions, using less reactive phosphorus reagents [39], but these are limited to phosphonylations of activated positions, such as *α*‐amino and benzylic C(sp^3^)–H bonds (Scheme 1c, top) [40, 41, 42, 43, 44, 45, 46, 47, 48, 49, 50, 51, 52, 53, 54]. One exception was reported by Li and coworkers, who developed a photocatalytic C(sp^3^)–H phosphonylation via 1,5‐HAT from *O‐*benzoylhydroxamic acids (Scheme 1c, bottom) [55]. However, this intramolecular HAT strategy has limited generality because of the requirement for the hydroxamic acid directing group.

Ligand‐to‐metal charge transfer (LMCT) photocatalysis using first‐row transition metal chlorides has recently gained attention as a straightforward approach to functionalize unactivated alkanes via chlorine radical‐mediated HAT [56]. These methods have allowed the substitution of strong C(sp^3^)–H bonds with a broad range of carbon‐ and heteroatom‐based functional groups [57, 58, 59, 60, 61, 62, 63, 64, 65]. Notably, the formation of C(sp^3^)–P bonds has also been reported using FeCl_3_ as a photocatalyst; however, this has been limited to the synthesis of alkyl phosphines using highly reactive chlorophosphines as radical traps [66, 67, 68], whereas related phosphonylations to access phosphonate esters are unknown. We reasoned that LMCT photocatalysis could be extended to phosphonate synthesis if a sufficiently reactive phosphonylating agent could be identified, which could provide a more general approach for the synthesis of alkyl phosphonates from alkanes. Herein, we describe the development of an LMCT‐mediated C(sp^3^)–H phosphonylation of unactivated alkanes using FeCl_3_ as a photocatalyst and a novel mandelonitrile‐derived phosphonylating agent, which possesses an electron‐deficient radical leaving group to facilitate photocatalyst turnover by reoxidation of Fe^II^ to Fe^III^ (Scheme 1d).

## 2 Results and Discussion

### 2.1 Reaction Development

**SCHEME 2:**
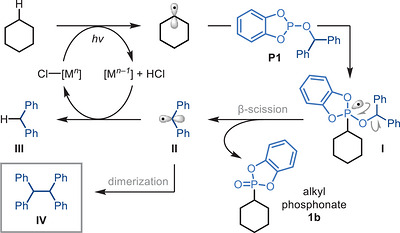
Fate of the radical leaving group in phosphonylations with **P1**.

We commenced our studies by investigating the phosphonylation of cyclohexane using FeCl_3_ as a photocatalyst under irradiation with 390 nm LEDs (Table 1) [67]. To enable productive phosphonate formation, we required a phosphite that could effectively trap the secondary alkyl radical formed upon LMCT‐mediated HAT. We previously developed benzhydryl catechol phosphite **P1** for photoredox‐catalyzed phosphonylations of primary and secondary alkyl radicals, where the aromatic diol ligand facilitates radical addition to phosphorus by stabilizing the intermediate phosphoranyl radical, and the benzhydryl acts as an efficient radical leaving group to promote subsequent phosphonate formation via *β*‐scission [19]. Pleasingly, phosphonylation of cyclohexane with **P1** was successful, providing phosphonate **1a** after partial methanolysis of the initially generated catechol phosphonate **1b** (see Scheme 2), but only in a low yield of 15% (entry 1). Although the yield of **1a** could be increased to 43% by changing the catalyst to FeCl_2_ (entry 2 and Table S1), no further improvements were made when using phosphite **P1**.

**TABLE 1:** Optimization Studies^a^.

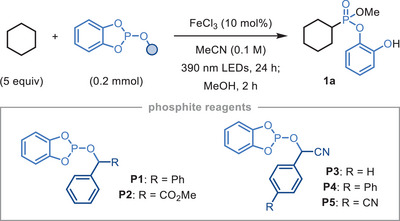			
Entry	Phosphite	Other modifications	3a (%)^b^
1	**P1**		15 (15)^c^
2	**P1**	FeCl_2_ (20 mol%)	43 (45)^c^
3	**P2**		4
4	**P3**		**85**
5	**P4**		48
6	**P5**		72
7	**P3**	20 mol% FeCl_3_	76
8	**P3**	5 mol% FeCl_3_	39
9	**P3**	FeCl_2_ (20 mol%)	83
10	**P3**	CuCl_2_ (20 mol%)	43
11	**P3**	In the dark	0
12	**P3**	No catalyst	3

^a^ Reaction performed with phosphite (0.20 mmol), cyclohexane (5 equiv), and FeCl_3_ (10 mol%) in MeCN (0.10 M) under irradiation with 390 nm LEDs for 24 h; MeOH (1 mL) was added and stirred for 2 h to cause partial methanolysis of the catechol phosphonate product for analysis.

^b^ Yields determined by ^31^P NMR with P(O)Ph_3_ as an internal standard.

^c^ Yield in parentheses is 1,1,2,2‐tetraphenylethane (**IV**) determined by ^1^H NMR analysis.

Our mechanistic hypothesis for phosphonylations with **P1** involves *β*‐scission of phosphoranyl radical intermediate **I** to form phosphonate **1b** and benzhydryl radical **II**, which is then reduced to a carbanion by the photocatalyst before protonation gives diphenyl methane **III** (Scheme 2) [19]. Surprisingly, **III** was not observed in our crude product mixtures, but we found significant amounts of 1,1,2,2‐tetraphenylethane (**IV**) (15% and 45% yield for entries 1 and 2, respectively, Table 1), the product of dimerization of the benzhydryl radical. This is indicative of a slow rate of reduction of the benzhydryl radical by the photocatalyst and, hence, an inefficient catalyst turnover. It was clear that we had to design a new radical phosphonylating agent. Therefore, we tested several novel catechol phosphites possessing more electron‐deficient radical leaving groups, with the aim of generating a more oxidizing radical that would facilitate catalyst turnover by reoxidation of Fe^II^ to Fe^III^. While substituting a phenyl group in **P1** for a methyl ester (**P2**) led to a dramatic reduction in yield (Table 1, entry 3), incorporating a nitrile group had the opposite effect, with **1a** obtained in 85% yield when using mandelonitrile‐derived phosphite **P3** (entry 4). Alternative phosphites possessing a benzyl nitrile‐based radical leaving group were then explored, including biphenyl and benzonitrile phosphites **P4** and **P5**, but these did not provide further improvements over **P3** (entries 5–6). The catechol group on the phosphite was found to be essential, since only trace product was observed with ethylene glycol or pinacol phosphites (see Table S2). Investigating alternative catalysts and catalyst loadings confirmed 10 mol% FeCl_3_ as optimal, although a comparable yield was obtained with 20 mol% FeCl_2_ (entries 7–10 and Table S3). Finally, control reactions demonstrated the essential roles of light and photocatalyst in the C(sp^3^)–H phosphonylation (entries 11–12).

### 2.2 Substrate Scope

**SCHEME 3:**
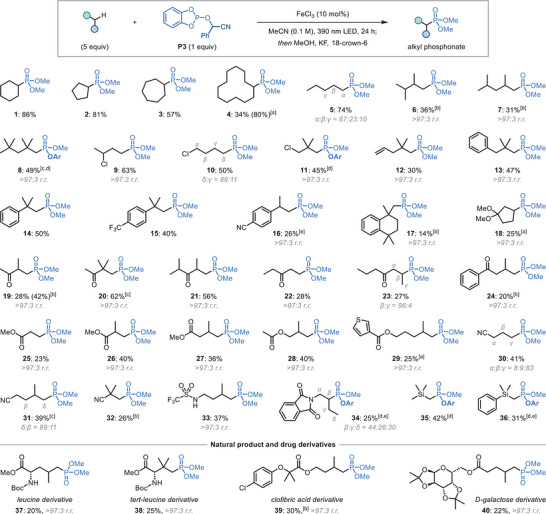
Substrate scope of the C–H phosphonylation. Standard conditions: **P3** (0.20 mmol), alkane (5 equiv.), and FeCl_3_ (10 mol%) in MeCN (0.10 M) were irradiated with 390 nm LEDs at 40 °C for 20 h; methanolysis of the catechol phosphonate ester was achieved by treatment with MeOH (2 mL), KF (10 equiv.), and 18‐crown‐6 (3 mol%) and stirring overnight at room temperature. Yields are of isolated products after chromatographic purification. Regioisomeric ratios (r.r.) were determined by ^31^P NMR analysis of crude reaction mixtures. (a) FeCl_2_ (20 mol%) and 370 nm LEDs were used. (b) Benzylidenemalononitrile (BMN, 1 equiv) was used as an additive. (c) 10 equiv. alkane substrate was used. (d) Only partial methanolysis of the catechol phosphonate ester was performed; Ar = 2‐hydroxyphenyl. (e) MeCN/ClCH_2_CN (9:1) was used as a solvent.

With optimized conditions in hand, we subsequently explored the scope of this C(sp^3^)–H phosphonylation by investigating the reaction of phosphite **P3** with a range of alkanes (Scheme 3). To simplify purification and analysis of the alkyl phosphonate products, the hydrolytically labile catechol phosphonates (cf. **1b** in Scheme 2) were converted to dimethyl phosphonates through methanolysis in the presence of KF and catalytic 18‐crown‐6, as previously reported by Li and coworkers [22, 55]. Using this protocol, both cyclic (**1**‐**4**) and acyclic alkanes (**5**‐**8**) could be converted to the corresponding phosphonylation products in moderate to high yields. Notably, selective substitution of less sterically hindered C(sp^3^)–H bonds was observed, leading to preferential phosphonylation of methyl groups over methylene (**5**, **7**, **8**) and methine (**6**, **7**) positions, which mirrors the selectivity previously observed for chlorine radical‐mediated borylations and phosphorylations using LMCT photocatalysis [61, 62, 67]. Chloroalkanes reacted with **P3** with high selectivity for the formation of primary alkyl phosphonates distal to the chlorine atom (**9**‐**11**). Methyl groups were also successfully phosphonylated in substrates bearing weak allylic (**12**) and benzylic (**13**) C(sp^3^)–H bonds; however, steric shielding of these potentially more reactive sites was required (see Section S2.29 for examples of unsuccessful substrates). Various alkyl‐substituted aromatic rings were also converted to their respective homobenzylic phosphonates (**14**‐**17**). For cyclic and acyclic ketone‐containing substrates, phosphonylation was found to occur with high selectivity for the formation of *β*‐ketophosphonates (**18**‐**23**) [59, 67], unless this position was sterically shielded (**24**). Unexpectedly, the ketone in the cyclopentanone‐derived product was converted to dimethyl acetal **18** during methanolysis of the phosphonate ester, whereas acyclic ketones were preserved during this process. Additional compatible functional groups included esters (**25**‐**29**), nitriles (**30**‐**32**), sulfonamides (**33**), and imides (**34**), with high regioselectivity generally observed for reactions at distal C(sp^3^)–H bonds. Methylsilanes were also phosphonylated to provide *α*‐silyl phosphonate products (**35**‐**36**), which were isolated as the mixed methyl‐catechol phosphonate esters (cf. **1a** in Table 1) due to their instability under the KF‐mediated transesterification conditions. We subsequently investigated the utility of this C(sp^3^)–H phosphonylation for late‐stage installation of phosphonate groups onto complex molecules. Gratifyingly, derivatives of amino acids (**37**, **38**), drug molecules (**39**), and monosaccharides (**40**) were transformed into the corresponding phosphonylation products with high regioselectivities and in synthetically useful yields.

The phosphonylation conditions shown in Scheme 3 were found to be general across a broad range of alkanes; however, minor modifications proved beneficial for certain substrates. These included using FeCl_2_ (20 mol%) as the photocatalyst under irradiation with 370 nm LEDs (**4**, **18**, **29**) or changing to a mixed solvent system of MeCN/chloroacetonitrile (**16**, **34**, **36**) [67]. Intriguingly, when investigating the regioselectivity of HAT in the phosphonylation of 2,3‐dimethylbutane through a competition reaction between **P3** and benzylidenemalononitrile (BMN, vide infra), we observed a significant improvement in the yield of phosphonate product **6** (see Table S8). We reasoned that BMN could intercept tertiary alkyl radicals that failed to react with **P3**, thus providing an alternative mechanism for catalyst turnover via reduction of the resulting 1,1‐dicyanoalkyl radical [59]. This modification also proved beneficial in phosphonylations of other alkanes possessing weaker tertiary C(sp^3^)–H bonds (**7**, **19**, **24**, **39**) and several other substrates (**17**, **32**) where the reasons for the improved yields with BMN are unclear.

### 2.3 Mechanistic Studies

**SCHEME 4:**
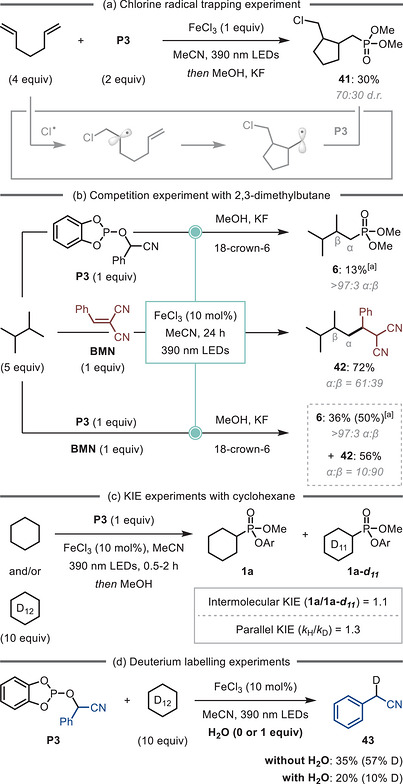
Mechanistic experiments. (a) Yields were determined by ^31^P NMR analysis. Ar = 2‐hydroxyphenyl.

To gain further insight into this phosphonylation reaction, a series of mechanistic studies was carried out. First, the involvement of chlorine radicals was supported by a radical trapping experiment with hepta‐1,6‐diene, which provided cyclic γ‐chlorophosphonate **41** in 30% yield (Scheme 4a). Given that regioselectivities in chlorine radical‐mediated HAT from unactivated C(sp^3^)–H bonds typically follow the trend of tertiary > secondary > primary [69], we subsequently investigated the origin of the high primary selectivity in our reactions by performing the phosphonylation of 2,3‐dimethylbutane in the presence of the competitive radical trap benzylidenemalononitrile (BMN, Scheme 4b) [58, 67]. The reaction of 2,3‐dimethylbutane with **P3** led to exclusive formation of primary alkyl phosphonate **6**, whereas reaction with BMN led to **42** with only moderate selectivity for alkylation of the primary position (*α*:*β* = 61:39). The competition experiment with equimolar amounts of **P3** and BMN gave a mixture of **6** and **42**, with exclusive primary selectivity still observed for **6** but the regioselectivity of **42** switched in favor of tertiary C–H alkylation (*α*:*β* = 10:90). These results confirm that tertiary alkyl radicals are generated but are unreactive in phosphonylations with **P3** [19], and **P3** and BMN competitively trap primary alkyl radicals, which leads to the observed reduction in the primary selectivity of **42**. This supports a mechanism involving unselective HAT‐mediated generation of alkyl radicals followed by selectivity‐determining C─P bond formation. The improved yield of **6** in the presence of BMN (vide supra) suggests that unproductive tertiary alkyl radical formation otherwise leads to catalyst deactivation, but this is prevented by the cooperative tertiary radical alkylation with BMN [59].

Intermolecular and parallel kinetic isotope effect (KIE) experiments with cyclohexane and cyclohexane‐*d*_12_ provided values of 1.1 and 1.3, respectively (Scheme 4c). These small KIE values suggest that HAT is not the turnover‐limiting step of the reaction [70], which is in line with previous reports of chlorine radical‐mediated HAT via LMCT photocatalysis with FeCl_3_ [59, 60, 61, 66, 67]. Analysis of the phenylacetonitrile by‐product **43** formed during the reaction of cyclohexane‐*d*_12_ with **P3** revealed 57% deuterium incorporation, which was reduced to 10% when an equivalent of water was added to the reaction (Scheme 4d). These results support a mechanism involving single‐electron reduction of the phenylacetonitrile radical, followed by proton transfer from the HCl generated by HAT between cyclohexane and a chlorine radical.

**SCHEME 5:**
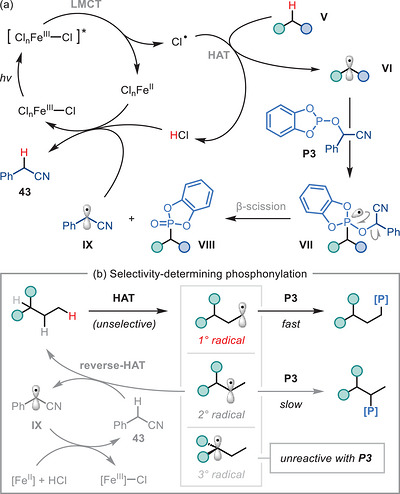
Proposed mechanism.

Based on the above results, our proposed mechanism for this C(sp^3^)–H phosphonylation is shown in Scheme 5a. Photoinduced LMCT of FeCl_3_, or [Fe^III^Cl_4_]^−^, formed through self‐ionization [59, 71], generates a chlorine radical and an Fe^II^ species. HAT from alkane **V** to the chlorine radical produces HCl and alkyl radical **VI**. Addition of **VI** to phosphite **P3** then gives phosphoranyl radical **VII**, which is stabilized by delocalization of the unpaired electron onto the catecholate group [19, 72]. Subsequent *β*‐scission forms cyclic catecholate phosphonate **VIII** and the stabilized phenylacetonitrile radical **IX**. Finally, reduction of **IX** by the Fe^II^ state of the photocatalyst and proton transfer with HCl gives by‐product **43** and regenerates the ground state Fe^III^ chloride complex to complete the photocatalytic cycle. The increased reaction efficiency with mandelonitrile phosphite **P3** compared to benzhydryl phosphite **P1** can be explained by the higher oxidation potential of radical leaving group **IX** (*E*_1/2_ [PhCHCN^•^/PhCHCN^–^] = −0.54 V vs. SCE) compared to benzhydryl radical **II** (*E*_1/2_ [Ph_2_CH^•^/Ph_2_CH^–^] = −1.14 V vs. SCE) [73, 74].

Based on the competition experiments in Scheme 4b, the high selectivity for phosphonylation of methyl over methylene and methine groups results from a more efficient reaction of **P3** with primary alkyl radicals compared to secondary and tertiary radicals (Scheme 5b). This is likely because of the faster reaction rate and lower reversibility in the formation of phosphoranyl radical **VII** from less stabilized and less sterically hindered primary radicals [26]. The slower rates of phosphonylation of secondary and tertiary alkyl radicals enable competing pathways to dominate. For example, HAT from the weak benzylic C─H bonds in by‐product **43** (bond dissociation energy = 82 kcal/mol) could occur to regenerate the starting alkane and form phenylacetonitrile radical **IX** [75], which could reoxidize Fe^II^ to Fe^III^. This reverse‐HAT with concomitant photocatalyst turnover (mediated by **43**) could explain why high‐yielding primary‐selective phosphonylation is still observed despite comparatively low selectivity for primary alkyl radical formation in the initial HAT (e.g., product **5**, Scheme 3). The beneficial effect of BMN in methine‐containing substrates could arise from a less favorable reverse‐HAT to the more stabilized tertiary alkyl radicals (compared to secondary radicals), which necessitates the addition of BMN as a cooperative radical trap to enable photocatalyst turnover.

## 3 Conclusion

In conclusion, we have developed a direct C(sp^3^)–H phosphonylation of unactivated alkanes via a photoinduced LMCT process using an inexpensive iron catalyst. The reactions proceed under mild conditions and display good tolerance for a variety of functional groups, which allowed the installation of phosphonate esters onto a broad range of substrates. Crucial for the success of the reaction was the design of a novel radical phosphonylating agent, mandelonitrile catechol phosphite (**P3**), which contains a key phenylacetonitrile group that (1) acts as a good radical leaving group to facilitate phosphonate formation through rapid *β*‐scission of phosphoranyl radical intermediates and (2) generates a strongly oxidizing radical that enables photocatalyst turnover by re‐oxidation of Fe^II^ to Fe^III^. For acyclic substrates, high selectivity for phosphonylation of methyl groups was observed, and this was found to result from a high preference for C─P bond formation of sterically unhindered primary alkyl radicals. As a result, the C(sp^3^)–H phosphonylation method was suitable for regioselective late‐stage installation of phosphonate esters onto complex organic molecules. Although the reaction efficiencies are generally only modest, we believe the simple conditions provide a practical approach for the synthesis of diverse phosphonate products.

## Conflicts of Interest

The authors declare no conflicts of interest.

## Supporting information




**Supporting File**: anie72379‐sup‐0001‐SuppMat.pdf

## Data Availability

The data that support the findings of this study are available in the supplementary material of this article.
