# Supplementary material for: Iron‐Photocatalyzed C(sp3)–H Phosphonylation of Alkanes
Source: Angew Chem Int Ed Engl. 2026 May 5;65(26):e6650940. doi: 10.1002/anie.6650940 (PMC13285471; doi:10.1002/anie.6650940)
Supplement: Supplementary file 1 — Supporting File: anie72379‐sup‐0001‐SuppMat.pdf [file ANIE-65-e6650940-s001.pdf]

SUPPORTING INFORMATION

Iron-Photocatalyzed C(sp^3)–H Phosphonylation of Alkanes

Ya Dong, Wangyujing Han, Hanwen Zhang, Santosh K. Pagire, Harry Meats, Adam Noble* and Varinder K. Aggarwal*

School of Chemistry, University of Bristol, Cantock's Close, Bristol BS8 1TS, United Kingdom

*e-mail: a.noble@bristol.ac.uk, v.aggarwal@bristol.ac.uk

TABLE OF CONTENTS

1. MATERIALS AND GENERAL METHODS 3
1.1. Glassware, Solvents and Reagents 3
1.2. Chromatography and Instrumentation 3
1.3. Naming of Compounds 4
1.4. Photochemical Equipment and Reaction Setup 4
2. EXPERIMENTAL DATA 5
2.1. Synthesis of Substrates 5
2.1.1. Synthesis of complex alkanes 5
2.1.2. Synthesis of alcohols 6
2.1.3. Synthesis of phosphites 7
2.2. Optimization Studies 12
2.2.1. Catalyst and light source screening with benzhydryl phosphite **P1** 12
2.2.2. Phosphite screening 13
2.2.3. Catalyst and light source screening with mandelonitrile phosphite **P3** 14
2.2.4. Effect of $FeCl_3$ loading with mandelonitrile phosphite **P3** 15
2.2.5. Effects of alkane equivalents with mandelonitrile phosphite **P3** 15
2.2.6. Solvent screening with mandelonitrile phosphite **P3** 16
2.2.7. Control experiments 16
2.2.8. Optimization with 2,3-dimethylbutane 17
2.2.9. Unsuccessful substrates 17
2.3. General Procedures 18
2.3.1. General Procedure A 18
2.3.2. General Procedure B 18
2.3.3. General Procedure C 19
2.3.4. Work-up Procedure 19
3. PRODUCT CHARACTERIZATION 20
4. MECHANISTIC STUDIES 44
4.1. Chlorine Radical Trapping Experiment 44
4.2. Competition Experiments with 2,3-Dimethylbutane 45
4.2.1. Trapping with benzylidenemalononitrile 45
4.2.2. Trapping with ethyl acrylate 47
4.3. Kinetic Isotope Effect Experiments 51
4.3.1. Intermolecular KIE experiment with cyclohexane 51
4.3.2. Parallel KIE experiment with cyclohexane 52
4.4. Deuterium-Labeling Experiments 54
4.4.1. Deuterium incorporation into phenylacetontrile by-product **43** 54
4.4.2. Deuterium/hydrogen exchange in the unsuccessful phosphonylation of toluene-d_8 57
4.5. UV-Vis Spectroscopy 59
5. SPECTROSCOPIC DATA 60
6. REFERENCES 169

1. MATERIALS AND GENERAL METHODS

1.1. Glassware, Solvents and Reagents

All reactions were conducted under an inert atmosphere of nitrogen using standard Schlenk manifold techniques unless mentioned otherwise. All glassware was oven- and/or flame-dried prior to use.

All anhydrous solvents (MeOH, MeCN, acetone, DMF, ethyl acetate etc.) were commercially supplied (ACROS) or dried using an Anhydrous Engineering alumina column drying system (CH_2Cl_2, THF, Et_2O etc.) and stored over 4 Å mol sieves. All reagents were purchased from commercial sources [Sigma Aldrich (Merck), Across, Fischer, Fluorochem Ltd, TCI, etc.] and used as received. Irradiation of reaction mixtures was achieved using a 40 W Kessil A160WE LED – 370 nm or 390 nm (set up: max intensity). Glass vials (10 mL) with PTFE/silicon septum lined caps were used as the standard reaction vessel for photoreaction. Brine refers to a saturated aqueous solution of NaCl.

1.2. Chromatography and Instrumentation

All reactions were carried out under an atmosphere of dry and deoxygenated argon in a glovebox (H_2O and O_2 < 0.1 ppm).

Thin layer chromatography (TLC) was performed to monitor reactions when practical using Merck Kieselgel 60 F254 fluorescent treated silica, which was visualized under UV light, or by staining with aqueous basic potassium permanganate followed by heating, *p*-anisaldehyde solution followed by heating, Hanessian's stain (CAM stain) followed by heating, or an ethanolic solution of phosphomolybdic acid followed by heating, as stated.

Flash column chromatography (FCC) was carried out using Sigma-Aldrich silica gel (60 Å, 230–400 mesh, 40–63 μm) or a Biotage IsoleraTM flash purification system.

NMR spectra were recorded at various field strengths, as indicated, using Varian VNMR 400 MHz, Varian VNMR 500 MHz, or Bruker Cryo 600 MHz for ^{1}H, ^{13}C, ^{31}P, and ^{19}F acquisitions. All NMR spectra were recorded at ~25 °C in $CDCl_3$ unless otherwise stated. Chemical shifts (δ) are reported in parts per million (ppm) and referenced to $CDCl_3$ (^{1}H: 7.26 ppm; ^{13}C: 77.16 ppm). Coupling constants (*J*) are given in Hertz (Hz) and refer to corresponding multiplicities (bs = broad singlet/signal, s = singlet, d = doublet, t = triplet, q = quartet, quin = quintet, sex = sextet, h = heptet, m = multiplet, dd = doublet of doublets, etc.). The ^{1}H NMR spectra are reported as follows: chemical shift (multiplicity, coupling constants, number of protons, assignment). NMR assignments were made according to spin systems, using two-dimensional NMR spectroscopy (COSY, HSQC, HMBC) to assist the characterization. Where an assignment could not be made unambiguously, no assignments are given. NMR yields were determined by ^{1}H NMR analysis using 1,1,2,2-Tetrachloroethane (TCE) or triphenylphosphine oxide as an internal standard.

High resolution mass spectra (HRMS) were recorded on a Bruker Daltonics MicrOTOF II by Electrospray Ionisation (ESI); a Thermo Scientific QExactive by Electron Ionisation (EI); a Thermo Scientific Orbitrap Elite by ESI or Atmospheric Pressure Chemical Ionisation (APCI); or a Bruker UltrafleXtreme by Matrix-assisted Laser Desorption/Ionisation (MALDI).

IR spectra were recorded neat as a thin film on a Perkin Elmer Spectrum One FT-IR. Selected absorption

maxima (ν_{max}) are reported in wavenumbers (cm^{-1}).

UV-Vis absorption spectra were recorded using an Agilent Technologies Cary 300 UV/Vis spectrophotometer, in quartz cuvettes with a path length of 10 mm.

1.3. Naming of Compounds

Compound names are those generated by ChemDraw Professional 20.0 software (PerkinElmer), following the IUPAC nomenclature.

1.4. Photochemical Equipment and Reaction Setup

The violet LED lamps were either 40 W Kessil PR160-390 nm (used with intensity dial turned fully clockwise) or 40 W Kessil PR160-370 nm Gen 2 (used with the intensity dial set to 100). The reaction vials were positioned ~5 cm from a single 40 W Kessil LED lamp (Figure S1). During the photoinduced reactions, heat generated from the LED lamps resulted in warming of the reactions to approximately 40–45 ºC.

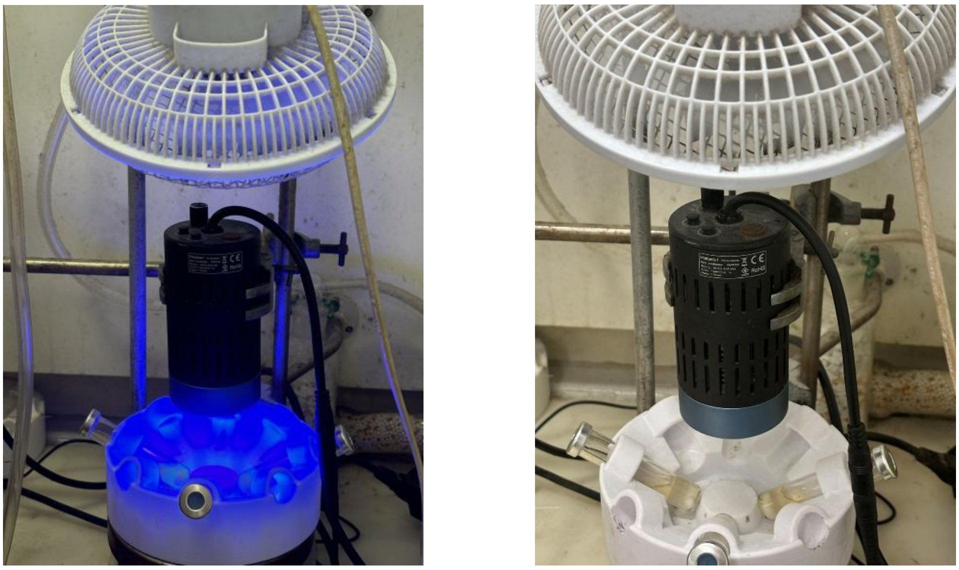

Figure S1. Photochemical reaction setup.

2. EXPERIMENTAL DATA

2.1. Synthesis of Substrates

2.1.1. Synthesis of complex alkanes

Methyl (*tert*-butoxycarbonyl)-*L*-leucinate (37-S)

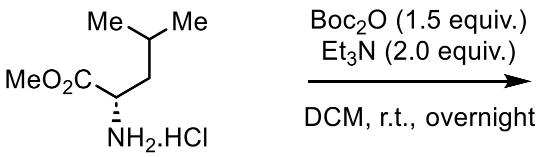

37-S

Di-*tert*-butyldicarbonate (1.8 g, 8.2 mmol. 1.5 equiv.) was added to a solution of L-Leucine methyl ester hydrochloride (1.0 g, 5.6 mmol, 1.00 equiv.) and triethylamine (1.54 mL, 11 mmol, 2 equiv.) in dichloromethane (20 mL). The reaction mixture was stirred at room temperature overnight, then washed with citric acid (10%), saturated aqueous $NaHCO_3$, and brine subsequently, and dried over sodium sulfate. The solution was concentrated in vacuum. The residue was purified by flash chromatography using EtOAc/hexane (10%-20%), giving the corresponding product **37-S** as colorless oil (quantitative).

NMR Spectroscopy (*see spectra*):

> ^{1}**H NMR** (400 MHz, $CDCl_3$) δ_H 4.88 (d, *J* = 8.8 Hz, 1H), 4.36 – 4.24 (m, 1H), 3.72 (s, 3H), 1.69 (dq, *J* = 8.0, 6.2 Hz, 1H), 1.60 (td, *J* = 12.0, 6.8 Hz, 1H), 1.52 – 1.45 (m, 1H), 1.43 (s, 9H), 0.93 (dd, *J* = 6.6, 3.4 Hz, 6H) ppm;
>
> ^{13}**C NMR** (101 MHz, $CDCl_3$) δ_C 174.1, 155.5, 79.9, 79.9, 52.3, 52.1, 41.9, 28.4, 24.9, 22.9, 22.0 ppm.

All recorded spectroscopic data matched those previously reported in the literature.[1]

Methyl (*L*)-2-((*tert*-butoxycarbonyl)amino)-3,3-dimethylbutanoate (38-S)

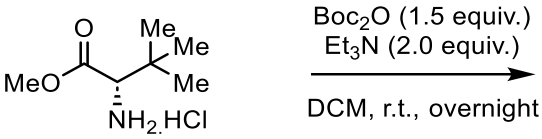

38-S

Di-*tert*-butyldicarbonate (1.8 g, 8.2 mmol. 1.5 equiv.) was added to a solution of *L*-*tert*-Leucine methyl ester hydrochloride (1.0 g, 5.6 mmol,1.00 equiv.) and triethylamine (1.54 mL, 11 mmol, 2 equiv.) in dichloromethane (20 mL). The reaction mixture was stirred at room temperature overnight, washed with citric acid (10%), saturated aqueous $NaHCO_3$, and brine subsequently, and dried over sodium sulfate. The solution was concentrated in vacuum. The residue was purified by flash chromatography using EtOAc/hexane (10%-20%), giving the corresponding product **38-S** as colorless oil (quantitative).

NMR Spectroscopy (*see spectra*):

^{1}H NMR (400 MHz, $CDCl_3$) δ_H 5.10 (d, *J* = 9.6 Hz, 1H), 4.10 (d, *J* = 9.6 Hz, 1H), 3.72 (s, 3H), 1.43 (s, 9H), 0.96 (s, 9H) ppm;

^{13}C NMR (101 MHz, $CDCl_3$) δ_C 172.7, 155.6, 79.8, 61.7, 51.8, 34.7, 28.4, 26.5 ppm.

All recorded spectroscopic data matched those previously reported in the literature.[2]

2.1.2. Synthesis of alcohols

2-([1,1'-Biphenyl]-4-yl)-2-hydroxyacetonitrile (P4-S)

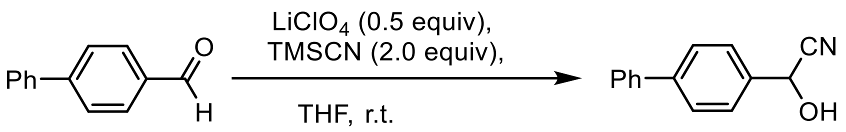

Under N_2 atmosphere, $LiClO_4{\cdot}3H_2O$ (10.0 mmol, 0.5 equiv.) was added to a solution of [1,1'-biphenyl]-4-carbaldehyde (3.6 g, 20.0 mmol, 1.0 equiv.) and TMSCN (40 mmol, 2.0 equiv.) in dry THF (20 mL), then the reaction mixture was stirred at room temperature for 30-60 minutes until the completely conservation of aldehyde A detected by TLC. Then the lithium perchlorate was filtered off and CH_2Cl_2 (50 mL) was added. The organic layer was washed with 10% aqueous HCl solution, dried over anhydrous Na_2SO_4, and evaporated in vacuo to directly afford **P4-S** (3.6 g, 86%) as a white solid without further purification.

NMR Spectroscopy (*see spectra*):

^{1}H NMR (400 MHz, $CDCl_3$) δ_H 7.69 – 7.52 (m, 6H), 7.49 – 7.42 (m, 2H), 7.41 – 7.34 (m, 1H), 5.55 (s, 1H) ppm.

^{13}C NMR (101 MHz, $CDCl_3$) δ_C 143.1, 140.1, 134.2, 129.1, 128.1, 128.1, 127.3, 118.8, 63.7 ppm.

All recorded spectroscopic data matched those previously reported in the literature.[3]

4-(Cyano(hydroxy)methyl)benzonitrile (P5-S)

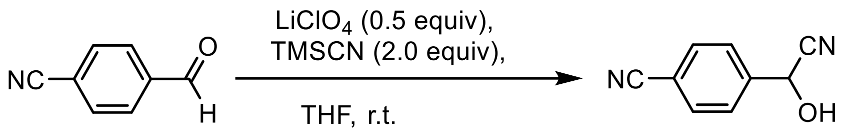

Under N_2 atmosphere, $LiClO_4{\cdot}3H_2O$ (10.0 mmol, 0.5 equiv.) was added to a solution of 4-formylbenzonitrile (20 mmol, 1.0 equiv.) and TMSCN (40 mmol, 1.0 equiv.) in dry THF (20 mL), then the reaction mixture was stirred at room temperature for 30-60 minutes until the completely conservation of aldehyde A detected by TLC. Then the lithium perchlorate was filtered off and CH_2Cl_2 (50 mL) was added. The organic layer was washed with 10% aqueous HCl solution, dried over anhydrous Na_2SO_4, and evaporated in vacuo to directly afford **P5-S** (2.8 g, 89%) as a white solid without further purification.

NMR Spectroscopy (*see spectra*):

^{1}H NMR (400 MHz, $CDCl_3$) δ_H 7.90 – 7.49 (m, 4H), 5.65 (d, *J* = 5.1 Hz, 1H), 3.03 (s, 1H) ppm.

^{13}C NMR (101 MHz, $CDCl_3$) δ_C 40.0, 133.1, 127.4, 118.1, 118.0, 113.8, 62.8 ppm.

All recorded spectroscopic data matched those previously reported in the literature.[4]

2.1.3. Synthesis of phosphites

The following phosphites (**P1**, **P6**, and **P8-P11**) were synthesized according to previous literature procedures.[5]

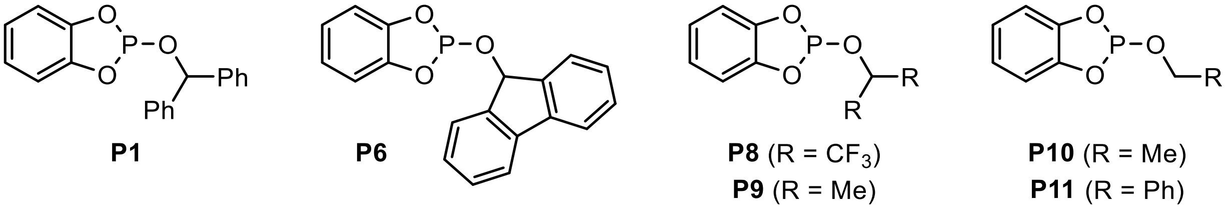

P1 **P6** **P8** (R = CF_3) **P9** (R = Me) **P10** (R = Me) **P11** (R = Ph)

Phosphites **P2-P5**, **P7** and **P12-P14** were prepared according to a modified literature procedure.[5]

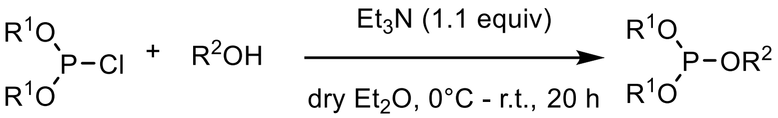

Using a modified literature procedure.[5] Under a N_2 atmosphere, chlorophosphite (1.1 equiv.) was added by dropwise to a stirred and cooled (0 °C) solution of triethylamine (1.1 equiv.) and the corresponding alcohol (1.0 equiv.) in 0.15 M of dry diethyl ether. The mixture was allowed to warm up to ambient temperature. After 20 h the Et_3N hydrochloride was filtered off through Celite pad and rinsed well with diethyl ether under a N_2 atmosphere. Solvent was removed from the combined filtrate under reduced pressure to give the phosphites.

Methyl 2-(benzo[*d*][1,3,2]dioxaphosphol-2-yloxy)-2-phenylacetate (P2)

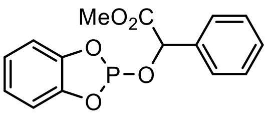

Prepared following the above **General Procedure** with methyl 2-hydroxy-2-phenylacetate (1.66 g, 10.0 mmol). The title compound (2.7 g, 90%) was used without further purification as a white solid.

NMR Spectroscopy (*see spectra*):

^{1}H NMR (400 MHz, $CDCl_3$) δ_H 7.41-7.35 (m, 1H), 7.34 – 7.27 (m, 2H), 7.21 (d, *J* = 5.5 Hz, 2H), 7.10 (d, *J* = 7.2 Hz, 1H), 7.03 – 6.89 (m, 3H), 5.21 (d, *J* = 8.3 Hz, 1H), 3.61 (s, 3H) ppm.

^{13}C NMR (101 MHz, $CDCl_3$) δ_C 169.3 (d, *J* = 2.0 Hz), 145.0 (t, *J* = 15.1 Hz), 135.2 (d, J = 2.0 Hz), 128.9, 128.4, 126.6, 122.8 (d, *J* = 5.0 Hz), 112.2, 112.0, 74.5 (d, *J* = 2.0 Hz), 52.5 ppm.

^{31}P NMR (162 MHz, $CDCl_3$) δ 126.7 (s) ppm.

IR (film) *V* max: 3056, 1740, 1475, 1229, 824, 745, 626 cm^{-1}.

HRMS (EI^+):calcd. for $C_{15}H_{13}O_3P$[M-MeOH] $^+$272.0233, found 272.0230.

2-(Benzo[*d*][1,3,2]dioxaphosphol-2-yloxy)-2-phenylacetonitrile (P3)

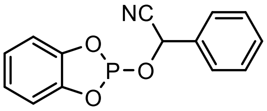

Prepared following the above procedure with 2-hydroxy-2-phenylacetonitrile (1.33 g, 10.0 mmol). The title compound (2.4 g, 90%) was used without further purification as the yellow oil.

NMR Spectroscopy (*see spectra*):

^{1}H NMR (400 MHz, $CDCl_3$) δ_H 7.38 (d, *J* = 7.3 Hz, 3H), 7.33 – 7.27 (m, 2H), 7.23 (d, *J* = 7.2 Hz, 1H), 7.06 (d, *J* = 3.1 Hz, 3H), 5.40 (d, *J* = 7.1 Hz, 1H) ppm.

^{13}C NMR (101 MHz, $CDCl_3$) δ_C 145.0 (d, *J* = 7.0 Hz), 133.0 (d, *J* = 2.0 Hz), 130.36, 129.25, 127.64, 127.15, 124.47, 123.8 (d, *J* = 13.1 Hz), 116.5 (d, *J* = 2.0 Hz), 114.2, 112.8 (d, *J* = 44.4 Hz), 63.5 ppm.

^{31}P NMR (162 MHz, $CDCl_3$) δ_P 127.5 (s) ppm.

IR (film) *V* max: 3003, 2251, 1759, 1475, 1304, 1051, 906, 840, 725, 534 cm^{-1}.

HRMS (EI^+):calcd. for $C_{14}H_{10}NO_3P(M)^{+:}$ 271.0393, found 271.0388.

2-([1,1'-Biphenyl]-4-yl)-2-(benzo[*d*][1,3,2]dioxaphosphol-2-yloxy)acetonitrile (P4)

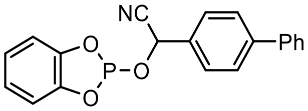

Prepared following the above procedure with 2-([1,1'-biphenyl]-4-yl)-2-hydroxyacetonitrile (2.09 g, 10.0 mmol). The title compound (3.1 g, 89%) was used without further purification as a light yellow solid.

NMR Spectroscopy (*see spectra*):

^{1}H NMR (400 MHz, $CDCl_3$) δ_H 7.63 – 7.59 (m, 4H), 7.51 – 7.47 (m, 2H), 7.44 – 7.38 (m, 3H), 7.27 (dd, *J* = 8.3, 1.4 Hz, 1H), 7.15 – 7.08 (m, 3H), 5.47 (d, *J* = 7.0 Hz, 1H) ppm.

^{13}C NMR (101 MHz, $CDCl_3$) δ_C 145.1, 145.0, 143.4, 140.0, 131.8, 129.1, 128.1, 127.9, 127.6, 127.3, 124.5, 123.8, 123.7, 116.4, 113.0, 112.5, 63.3 ppm.

^{31}P NMR (162 MHz, $CDCl_3$) δ 126.8 ppm.

IR (film) *V* max: 3032, 2251, 1600, 1474, 1228, 994, 826, 555 cm^{-1}.

HRMS (ESI^+): calcd. for $C_{20}H_{14}N_2O_3PNa$[M+Na] 370.0604, found 370.0608.

4-((Benzo[*d*][1,3,2]dioxaphosphol-2-yloxy)(cyano)methyl)benzonitrile (P5)

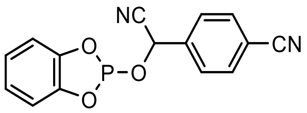

Prepared following the above procedure with 4-(cyano(hydroxy)methyl)benzonitrile (1.58 g, 10.0 mmol). The title compound (2.7 g, 93%) was used without further purification as a yellow oil.

NMR Spectroscopy (*see spectra*):

^{1}H NMR (400 MHz, $CDCl_3$) δ 7.41 – 7.37 (m, 2H), 7.30 (dt, *J* = 6.5, 1.8 Hz, 2H), 7.25 – 7.21 (m, 1H), 7.07 (td, *J* = 3.3, 1.5 Hz, 2H), 5.41 (d, *J* = 7.1 Hz, 1H) ppm.

^{13}C NMR (101 MHz, $CDCl_3$) δ_C 144.4 (d, *J* = 12.1 Hz), 144.0 (d, *J* = 7.1 Hz), 137.4(d, *J* = 1.0 Hz), 132.7, 127.4, 124.2, 123.7 (d, *J* = 12.1 Hz), 117.6, 115.4 (d, *J* = 2.0 Hz), 113.9, 112.8, 112.4, 62.3 ppm;

^{31}P NMR (162 MHz, $CDCl_3$) δ 126.8 ppm.

IR (film) *V* max: 3067, 2452, 1886, 1474, 1227, 1007, 827, 741, 529 cm^{-1}.

HRMS (EI^+): calcd. for $C_{14}H_{10}NO_3P[M]^+$ 271.0393, found 271.0386.

2-(2,2,2-Trifluoro-1-phenylethoxy)benzo[*d*][1,3,2]dioxaphosphole (P7)

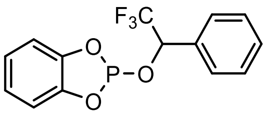

Prepared following the above procedure with 2-hydroxy-2-phenylacetonitrile (1.76 g, 10.0 mmol). The title compound (2.7 g, 87%) was used without further purification as the yellow oil.

NMR Spectroscopy (*see spectra*):

^{1}H NMR (400 MHz, $CDCl_3$) δ_H 7.52 (dt, *J* = 31.4, 7.3 Hz, 3H), 7.33 (dd, *J* = 13.5, 7.7 Hz, 3H), 7.27 – 7.19 (m, 1H), 7.14 (qd, *J* = 7.7, 2.5 Hz, 1H), 6.94 (d, *J* = 7.9 Hz, 1H), 5.18 - 5.12 (m, 1H) ppm.

^{13}C NMR (101 MHz, $CDCl_3$) δ_C 145.2 (d, *J* = 7.1 Hz), 144.6 (d, *J* = 8.1 Hz), 132.0, 129.9, 128.4, 127.7, 123.3 (d, *J* = 10.1 Hz), 112.6, 112.2, 74.4 (q, *J* = 101 Hz) ppm.

^{19}F NMR (377 MHz, $CDCl_3$) δ 77.5 (t, *J* = 2.4 Hz) ppm.

^{31}P NMR (162 MHz, $CDCl_3$) δ 126.7 (s) ppm.

IR (film) *V* max: 3294, 1475, 1229, 1175, 1132, 1023, 822, 699 cm^{-1}.

HRMS (EI^+):calcd. for $C_{14}H_{10}O_3PF_3(M^+)$ 314.0314, found 314.0308.

2-((1,3,2-Dioxaphospholan-2-yl)oxy)-2-phenylacetonitrile (P12)

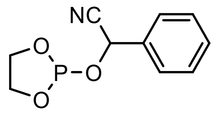

Prepared following the above procedure with 2-hydroxy-2-phenylacetonitrile (1.33 g, 10.0 mmol). The title compound (2.0 g, 91%) was used without further purification as a light green oil.

NMR Spectroscopy (see spectra):

^{1}**H NMR** (400 MHz, $CDCl_3$) δ_H 7.49 (dd, *J* = 5.4, 2.1 Hz, 2H), 7.48 – 7.39 (m, 4H), 5.78 (d, *J* = 6.8 Hz, 1H), 4.31 – 4.19 (m, 2H), 4.12 – 4.00 (m, 2H) ppm.

^{13}**C NMR** (101 MHz, $CDCl_3$) δ_C 134.0 (d, *J* = 2.0 Hz), 129.9, 129.1, 126.8, 117.5 (d, *J* = 2.0 Hz), 64.4 (q, *J* = 8.8 Hz), 62.5 (q, *J* = 12.2 Hz) ppm.

^{31}**P NMR** (162 MHz, $CDCl_3$) δ_P 138.5 (s) ppm.

IR (film) *V* max: 3292, 2447, 1454, 1258, 1134, 907, 733, 578, 414 cm^{-1}.

HRMS (EI^+) calcd. for $C_{10}H_{10}NO_3P$[M^+] 223.0393, found 223.0390.

2-Phenyl-2-((4,4,5,5-tetramethyl-1,3,2-dioxaphospholan-2-yl)oxy)acetonitrile (P13)

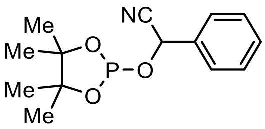

Prepared following the above procedure with 2-hydroxy-2-phenylacetonitrile (1.33 g, 10.0 mmol). The title compound (2.5 g, 89%) was used without further purification as a light green oil.

NMR Spectroscopy (see spectra):

^{1}**H NMR** (400 MHz, $CDCl_3$) δ_H 7.47 (dt, *J* = 8.2, 2.6 Hz, 2H), 7.44 – 7.39 (m, 3H), 1.39 (d, *J* = 34.4 Hz, 6H), 1.27 (d, *J* = 12.8 Hz, 6H) ppm.

^{13}**C NMR** (101 MHz, $CDCl_3$) δ_C 134.6 (d, *J* = 4.0 Hz), 129.8, 129.2, 126.9, 117.8 (d, *J* = 3.4 Hz), 86.0 (t, *J* = 9.4 Hz), 63.2 (d, *J* = 20.3 Hz), 25.4(d, *J* = 2.7 Hz), 25.3 (d, *J* = 2.7 Hz), 25.0, 24.8 ppm.

^{31}**P NMR** (162 MHz, $CDCl_3$) δ_P 148.8 (s) ppm.

IR (film) *V* max: 3069, 2851,1806, 1455, 1373, 1137, 955, 905, 729, 550, 423 cm^{-1}.

HRMS (EI^+) calcd. for $C_{14}H_{18}NO_3P$[M^+] 279.1019, found 279.1016.

Cyano(phenyl)methyl diethyl phosphite (P14)

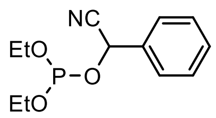

Prepared following the above procedure with 2-hydroxy-2-phenylacetonitrile (1.33 g, 10.0 mmol). The title compound (2.4 g, 90%) was used without further purification as a yellow oil.

NMR Spectroscopy (*see spectra*):

^{1}H NMR (400 MHz, $CDCl_3$) δ_H 7.54 – 7.48 (m, 2H), 7.45 – 7.38 (m, 3H), 5.94 (d, *J* = 7.1 Hz, 1H), 4.03 – 3.93 (m, 2H), 3.92 – 3.77 (m, 2H), 1.31 (t, *J* = 7.0 Hz, 3H), 1.24 (t, *J* = 7.0 Hz, 3H). ppm.

^{13}C NMR (101 MHz, $CDCl_3$) δ_C 134.6 (d, *J* = 1.0 Hz), 129.78, 129.14, 127.26, 118.06, 60.7 (d, *J* = 7.1 Hz), 59.7 (q, *J* = 39.4 Hz), 16.9 (d, *J* = 6.1 Hz) ppm.

^{31}P NMR (162 MHz, $CDCl_3$) δ_P 139.4 (s) ppm.

IR (film) *V* max: 3067, 2979, 1709, 1495, 1455, 1193, 1035, 1017, 916, 739, 530, 402 cm^{-1}.

HRMS (EI^+) calcd. for $C_{12}H_{16}NO_3P$ [M^+] 253.0862, found 253.0860.

2.2. Optimization Studies

2.2.1. Catalyst and light source screening with benzhydryl phosphite P1

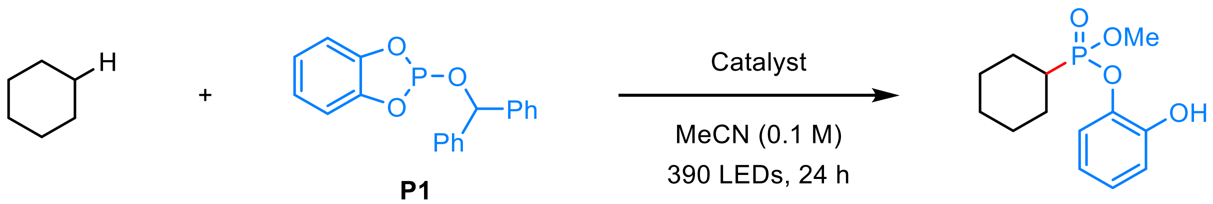

(5 equiv)

P1
(1 equiv)

1a

Entry[a]	catalyst	Yield of 1a
1	$FeCl_3$ (10 mol%)	15% (15)[b]
2	$FeCl_3$ (20 mol%)	7%
3	$FeCl_3$•$6H_2O$ (10 mol%)	5%
4	$FeCl_2$ (20 mol%)	43% (45%)[b]
5	$FeCl_2$•$4H_2O$ (20 mol%)	34%
6	$CuCl_2$ (20 mol%)	32%
7	$FeBr_2$ (20 mol%)	0%

Table S1. Catalyst and light source screening with phosphite P1

[a] Cyclohexane (5 equiv.), **P1** (0.2 mmol, 1 equiv.), MeCN (0.1 M, 2.0 mL), 390 nm LEDs, N_2, 40 °C, 24 h, then MeOH (1.0 mL), 2 h. Yields were determined by ^{31}P NMR with $OPPh_3$ as internal standard. [b] Yields in parentheses are those of 1,1,2,2-tetraphenylethane (**IV**) as determined by ^{1}H NMR analysis. NMR data for **IV** matched those previously reported in the literature.[6]

2.2.2. Phosphite screening

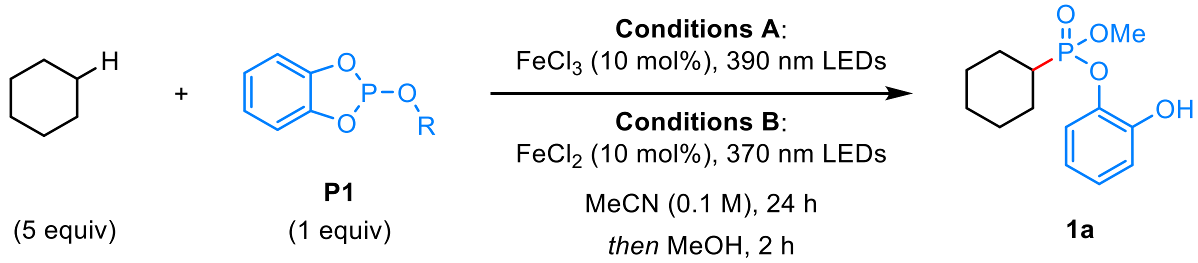

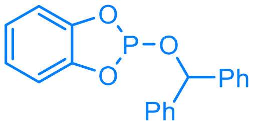

P1
43% (conditions A)
44% (conditions B)

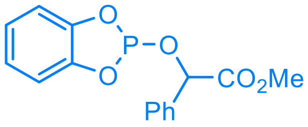

P2
4% (conditions A)
4% (conditions B)

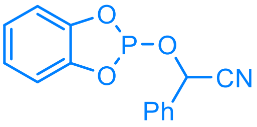

P3
85% (conditions A)
90% (conditions B)

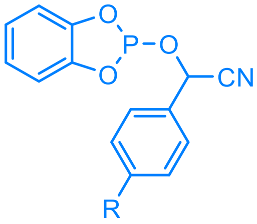

P4 (R = Ph)
48% (conditions A)

P5 (R = CN)
72% (conditions A)

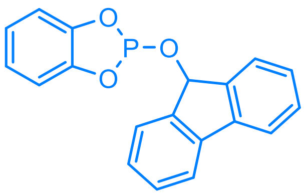

P6
29% (conditions B)

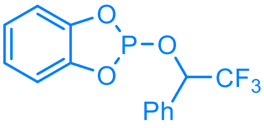

P7
4% (conditions A)
4% (conditions B)

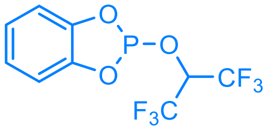

P8
4% (conditions B)

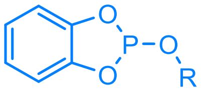

P9 (R = *i*-Pr): 0% (conditions B)
P10 (R = Et): 0% (conditions B)
P11 (R = Bn): 0% (conditions B)

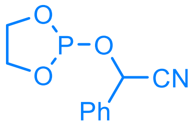

P12
2% (conditions A)

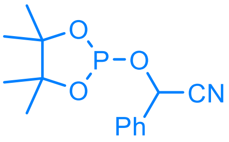

P13
2% (conditions A)

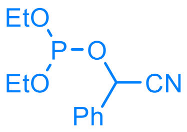

P14
2% (conditions A)

Table S2. Phosphite screening

Reaction conditions: Cyclohexane (5 equiv.), phosphite (0.2 mmol, 1 equiv.), $FeCl_X$ (10-20 mol%), MeCN (0.1 M, 2.0 mL), irradiation with Kessil LEDs (370 nm or 390 nm), N_2, 40 °C, 24 h, then MeOH (1.0 mL), 2 h. Yields were determined by ^{31}P NMR with $OPPh_3$ as internal standard. **Conditions A**: $FeCl_3$ (10 mol%), 390 nm LEDs. **Conditions B**: $FeCl_2$ (20 mol%), 370 nm LEDs.

2.2.3. Catalyst and light source screening with mandelonitrile phosphite P3

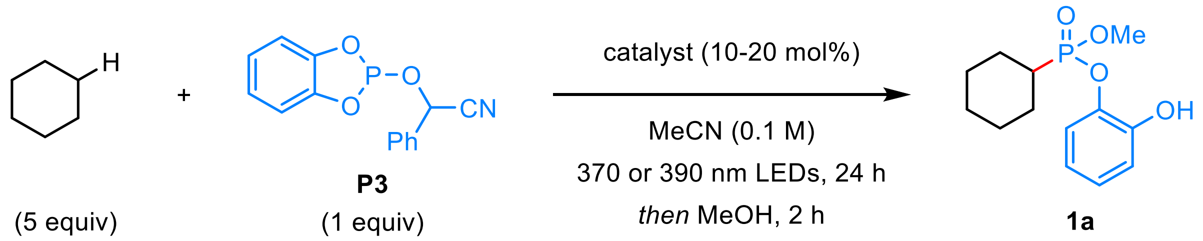

Entry[a]	Catalyst	Light source	**Yield of 1a**
1	$FeCl_3$ (10 mol%)	390 nm	85%[b]
2	$FeCl_2$ (20 mol%)	390 nm	83%
3	$CuCl_2$ (20 mol%)	390 nm	43%
4	$FeCl_3$ (10 mol%)	370 nm	30%
5	$FeCl_2$ (20 mol%)	370 nm	90%
6	$CuCl_2$ (20 mol%)	370 nm	14%

Table S3. Catalyst and light source screening with P3

[a] Cyclohexane (5 equiv.), **P3** (0.2 mmol, 1 equiv.), catalyst (10-20 mol%), MeCN (0.1 M, 2.0 mL), 390 or 370 nm Kessil LEDs, N_2, 40 °C, 24 h, then MeOH (1.0 mL), 2 h. Yields were determined by ^{31}P NMR with $OPPh_3$ as internal standard. [b] Reaction time = 20 h.

2.2.4. Effect of $FeCl_3$ loading with mandelonitrile phosphite P3

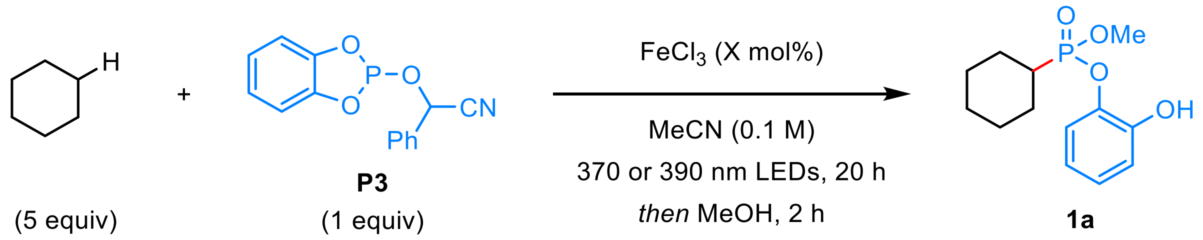

Entry[a]	$FeCl_3$ loading	**Yield of 1a**
1	20 mol%	76%
2	10 mol%	85%
3	5 mol%	39%

Table S4. Effect of $FeCl_3$ loading with P3

[a] Cyclohexane (5 equiv.), **P3** (0.2 mmol, 1 equiv.), $FeCl_3$ (X mol%), MeCN (0.1 M, 2.0 mL), 390 nm Kessil LEDs, N_2, 40 °C, 20 h, then MeOH (1.0 mL), 2 h. Yields were determined by ^{31}P NMR with $OPPh_3$ as internal standard.

2.2.5. Effects of alkane equivalents with mandelonitrile phosphite P3

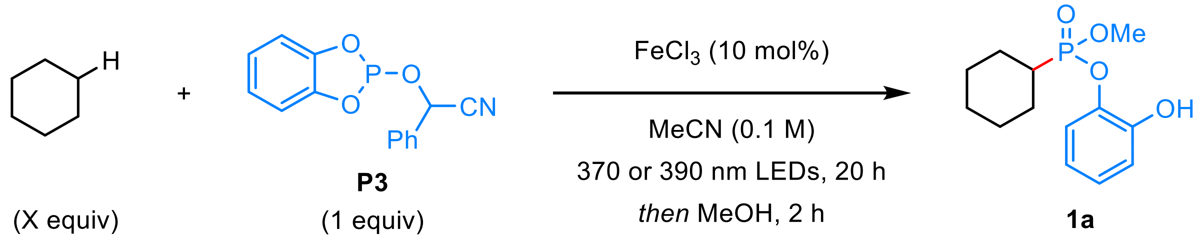

Entry[a]	cyclohexane equivalents	**Yield of 1a**
1	2.5	6%
2	5	85%
3	10	86%

Table S5. Effect of alkane equivalents with P3

[a] Cyclohexane (2.5-10 equiv.), **P3** (0.2 mmol, 1 equiv.), $FeCl_3$ (10 mol%), MeCN (0.1 M, 2.0 mL), 390 nm Kessil LEDs, N_2, 40 °C, 20 h, then MeOH (1.0 mL), 2 h. Yields were determined by ^{31}P NMR with $OPPh_3$ as internal standard.

2.2.6. Solvent screening with mandelonitrile phosphite P3

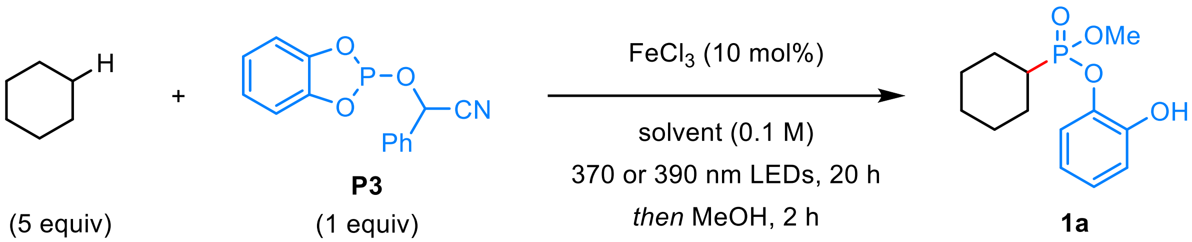

(5 equiv) **P3** (1 equiv) **1a**

Entry[a]	solvent	**Yield of 1a**
1	MeCN	85%
2	acetone	1%
3	DMF	0%
4	EtOAc	20%

Table S6. Solvent screening with P-9

Cyclohexane (5 equiv.), **P3** (0.2 mmol, 1 equiv.), $FeCl_3$ 10 mol%, solvent (0.1 M, 2.0 mL), 390 nm Kessil LEDs, N_2, 40 °C, 20 h, then MeOH (1.0 mL), 2 h. Yields were determined by ^{31}P NMR with $OPPh_3$ as internal standard.

2.2.7. Control experiments

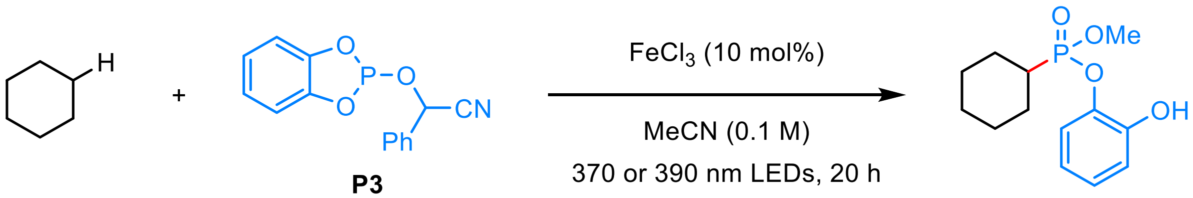

(5 equiv) **P3** (1 equiv) **1a**

Entry[a]	variation from above conditions	**Yield of 1a**
1	none	85%
2	no light	0%
3	no catalyst	3%

Table S7. Control experiments

Cyclohexane (5 equiv.), **P3** (0.2 mmol, 1 equiv.), $FeCl_3$ 10 mol%, MeCN (0.1 M, 2.0 mL), 390 nm Kessil LEDs, N_2, 40 °C, 20 h, then MeOH (1.0 mL), 2 h. Yields were determined by ^{31}P NMR with $OPPh_3$ as internal standard.

2.2.8. Optimization with 2,3-dimethylbutane

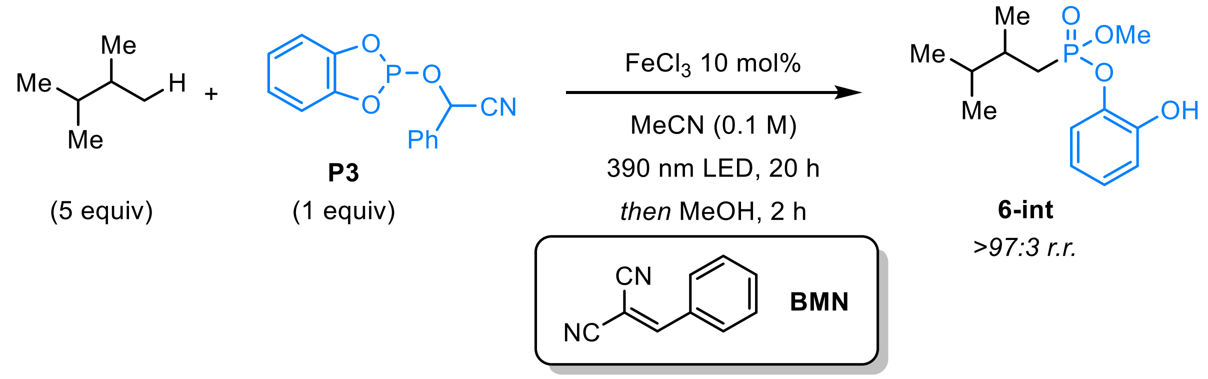

Entry	variation to standard conditions[a]	**Yield of 1a**
1	no change	13%
2	1 equiv. of **BMN** as additive	50%
3	MeCN/CH_2ClCN (9:1) as the solvent	38%
4	1 equiv. of **BMN** as additive, MeCN/CH_2ClCN (9:1) as the solvent	20%

Table S8. Optimization with 2,3-dimethylbutane

[a] Standard conditions: 2,3-dimethylbutane (5.0 equiv.), **P3** (0.2 mmol, 1.0 equiv.), $FeCl_3$ (10 mol%), MeCN (0.1 M, 2.0 mL), 390 nm LEDs, N_2, 40 °C, 20 h, then MeOH (1.0 mL), 2 h. Yields were determined by ^{31}P NMR with $OPPh_3$ as internal standard.

2.2.9. Unsuccessful substrates

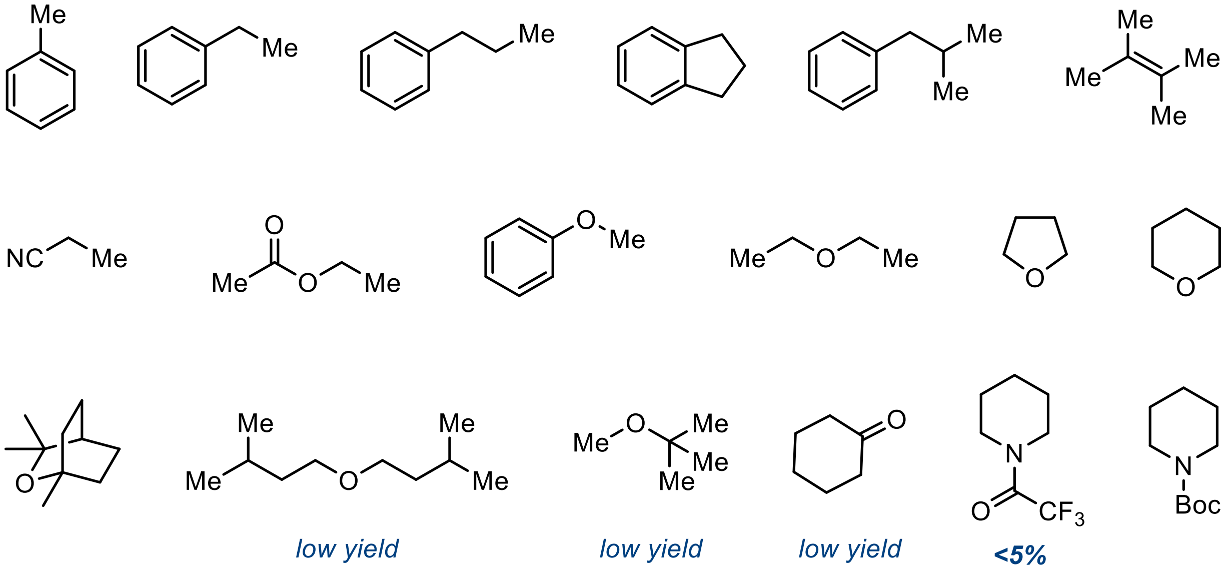

2.3. General Procedures

2.3.1. General Procedure A

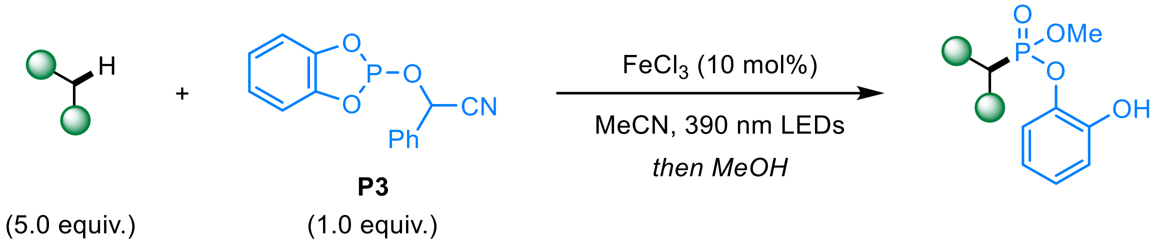

A flame dried 8 mL Biotage® microwave reaction vial equipped with a magnetic stir bar was transferred into an anhydrous, argon-filled glovebox. The vial was charged with $FeCl_3$ (3.2 mg, 0.020 mmol, 0.10 equiv.) and phosphite **P3** (54 mg, 0.20 mmol, 1.0 equiv.). Anhydrous acetonitrile (2.0 mL, c = 0.10 M) was added followed by the alkane (1.0 mmol, 5.0 equiv.). The vial was crimped with a cap with septum, removed from the glovebox, and placed 4 cm away from one Kessil PR160-390 nm LED lamp (see Figure S1). The reaction mixture was stirred at 1000 rpm and irradiated with fan cooling (reaction temperature = 40-45 °C) for 24 h. After irradiation, anhydrous methanol (1.0 mL) was added under N_2 and stirring was continued for 2 h. The reaction mixture was filtered through silica gel (2.3 cm diameter sintered funnel packed with 10 cm silica gel), eluting with ethyl acetate (50 mL), then concentrated *in vacuo*. The crude catechol phosphonate ester was then purified by flash column chromatography with silica gel or subjected to the work-up procedure to form the corresponding dimethyl phosphonate ester.

2.3.2. General Procedure B

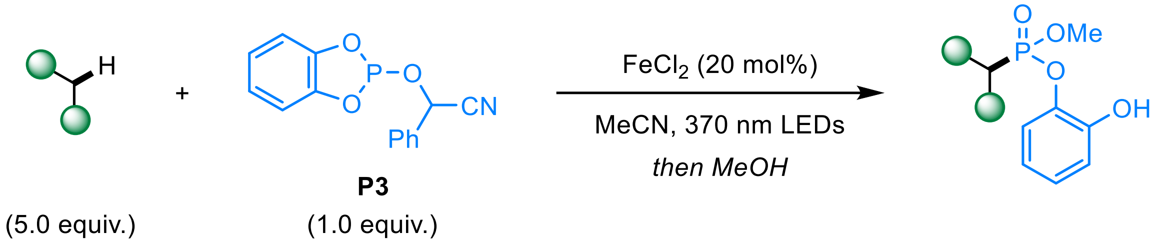

A flame dried 8 mL Biotage® microwave reaction vial equipped with a magnetic stir bar was transferred into an anhydrous, argon-filled glovebox. The vial was charged with $FeCl_2$ (5.1 mg, 0.040 mmol, 0.20 equiv.) and phosphite **P-9** (54 mg, 0.20 mmol, 1.0 equiv.). Anhydrous acetonitrile (2.0 mL, c = 0.10 M) was added followed by the alkane (1.0 mmol, 5.0 equiv.). The vial was crimped with a cap with septum, removed from the glovebox, and placed 4 cm away from one Kessil PR160-370 nm LED lamp (see Figure S1). The reaction mixture was stirred at 1000 rpm and irradiated with fan cooling (reaction temperature = 40-45 °C) for 24 h. After irradiation, anhydrous methanol (1.0 mL) was added under N_2 and stirring was continued for 2 h. The reaction mixture was filtered through silica gel (2.3 cm diameter sintered funnel packed with 10 cm silica gel), eluting with ethyl acetate (50 mL), then concentrated *in vacuo*. The crude catechol phosphonate ester was then purified by flash column chromatography with silica gel or subjected to the work-up procedure to form the corresponding dimethyl phosphonate ester.

2.3.3. General Procedure C

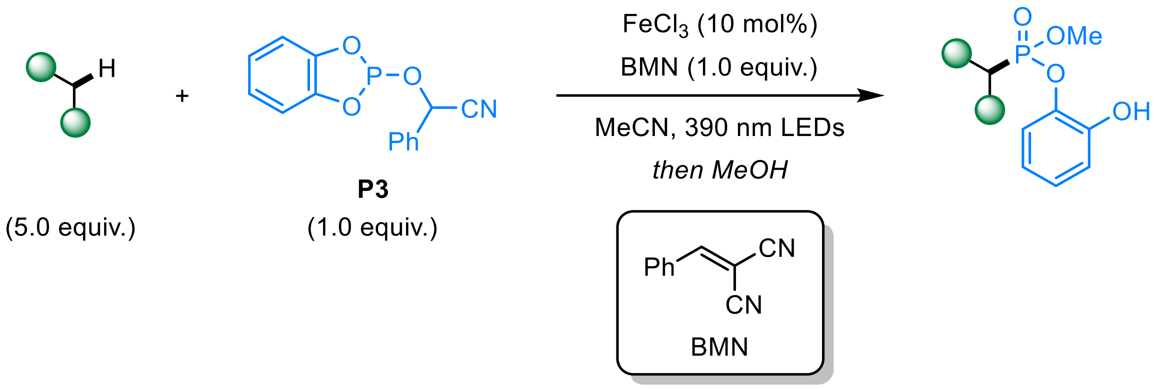

A flame dried 8 mL Biotage® microwave reaction vial equipped with a magnetic stir bar was transferred into an anhydrous, argon-filled glovebox. The vial was charged with $FeCl_3$ (3.2 mg, 0.020 mmol, 0.10 equiv.), phosphite **P-9** (54 mg, 0.20 mmol, 1.0 equiv.) and benzylidenemalononitrile (BMN, 31 mg, 0.20 mmol, 1.0 equiv.). Anhydrous acetonitrile (2.0 mL, c = 0.10 M) was added followed by the alkane (1.0 mmol, 5.0 equiv.). The vial was crimped with a cap with septum, removed from the glovebox, and placed 4 cm away from one Kessil PR160-390 nm LED lamp (see Figure S1). The reaction mixture was stirred at 1000 rpm and irradiated with fan cooling (reaction temperature = 40-45 °C) for 24 h. After irradiation, anhydrous methanol (1.0 mL) was added under N_2 and stirring was continued for 2 h. The reaction mixture was filtered through silica gel (2.3 cm diameter sintered funnel packed with 10 cm silica gel), eluting with ethyl acetate (50 mL), then concentrated in vacuo. The crude catechol phosphonate ester was then purified by flash column chromatography with silica gel or subjected to the work-up procedure to form the corresponding dimethyl phosphonate ester.

2.3.4. Work-up Procedure

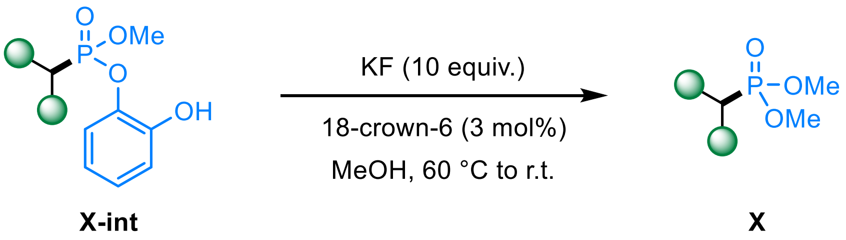

The catechol phosphonate ester **X-int** was dissolved in MeOH (2 mL) before adding KF (116 mg, 2.0 mmol) and 18-crown-6 (2 mg, 0.006 mmol). The resulting mixture was stirred and heated to 60 °C for 10 min to ensure KF was fully dissolved before allowing to cool to room temperature. The solution was stirred at room temperature and monitored by TLC. When **X-int** was fully consumed (1–12 h), the reaction mixture was concentrated *in vacuo* and purified by flash column chromatography with silica gel.

3. PRODUCT CHARACTERIZATION

Dimethyl cyclohexyl phosphonate (1)

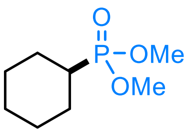

Prepared following **General Procedure A** and the **Work-up Procedure**, using cyclohexane (84.0 mg, 108 μL, 1.00 mmol, 5.00 equiv.). Purification by flash column chromatography (Biotage Isolera™, SNAP 5 g silica cartridge, 0–5% methanol in EtOAc) gave **1** (31 mg, 86% yield) as a colorless oil.

R_f = 0.2 (EtOAc, $KMnO_4$).

NMR Spectroscopy (*see spectra*):

> **^{1}H NMR** (600 MHz, $CDCl_3$) δ_H 3.73 (d, *J* = 10.5 Hz, 6H), 1.98 – 1.91 (m, 2H), 1.81 – 1.74 (m, 3H), 1.72 – 1.66 (m, 1H), 1.43 – 1.33 (m, 2H), 1.27 – 1.18 (m, 3H) ppm;
>
> **^{13}C NMR** (151 MHz, $CDCl_3$) δ_C 52.6 (d, *J* = 6.9 Hz), 35.4 (d, *J* = 142.3 Hz), 26.2 (d, *J* = 16.6 Hz), 26.0 (d, *J* = 4.9 Hz), 25.9 ppm;
>
> **^{31}P NMR** (162 MHz, $CDCl_3$) δ_P 35.9 ppm.

All recorded spectroscopic data matched those previously reported in the literature.[5]

Dimethyl cyclopentylphosphonate (2)

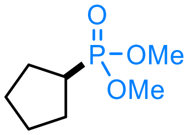

Prepared following **General Procedure A** and the **Work-up Procedure**, using cyclopentane (70.0 mg, 93.0 μL, 1.00 mmol, 5.00 equiv.). Purification by flash column chromatography (Biotage Isolera™, SNAP 5 g silica cartridge, 0–5% methanol in EtOAc) gave **2** (31 mg, 81%) as a colorless oil.

R_f = 0.2 (EtOAc, $KMnO_4$).

NMR Spectroscopy (*see spectra*):

> **^{1}H NMR** (400 MHz, $CDCl_3$) δ_H 3.76 (s, 3H), 3.74 (s, 3H), 2.14 (dq, *J* = 17.7, 8.8 Hz, 1H), 1.96 – 1 .83 (m, 3H), 1.83 – 1.66 (m, 5H). ppm;
>
> **^{13}C NMR** (151 MHz, $CDCl_3$) δ_C 52.6 (d, *J* =7.6 Hz), 34.7 (d, *J* =146.5 Hz), 27.2 (d, *J* = 1.2 Hz), 26.5 (d, *J* = 12.1 Hz) ppm.
>
> **^{31}P NMR** (162 MHz, $CDCl_3$) δ_P 38.5 (s) ppm.

All recorded spectroscopic data matched those previously reported in the literature.[7]

Dimethyl cycloheptylphosphonate (3)

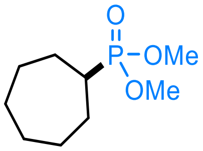

Prepared following **General Procedure A** and the **Work-up Procedure**, using cycloheptane (98.0 mg, 121 µL, 1.00 mmol, 5.00 equiv.). Purification by flash column chromatography (Biotage Isolera™, SNAP 5 g silica cartridge, 0–5% methanol in EtOAc) gave **3** (23 mg, 57%) as a colorless oil.

R*f* = 0.3 (EtOAc, $KMnO_4$)

NMR Spectroscopy (*see spectra*):

> **^{1}H NMR** (600 MHz, $CDCl_3$) δ_H 3.74 (d, *J* = 10.5 Hz, 6H), 2.08 – 1.96 (m, 2H), 1.95 – 1.83 (m, 1H), 1.82 – 1.72 (m, 2H), 1.62 – 1.44 (m, 8H). ppm.
>
> **^{13}C NMR** (101 MHz, $CDCl_3$) δ_C 52.7 (d, *J* = 7.0 Hz), 36.5 (d, *J* = 136.2 Hz), 28.3, 27.8 (d, *J* = 17.6 Hz), 27.4 (d, *J* = 4.2 Hz).
>
> **^{31}P NMR** (162 MHz, $CDCl_3$) δ_P 38.5 (s) ppm.

All recorded spectroscopic data matched those previously reported in the literature.[7]

Dimethyl cyclododecylphosphonate (4)

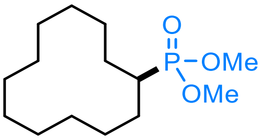

Prepared following **General Procedure B** and the **Work-up Procedure**, using cyclododecane (168 mg, 1.00 mmol, 5.00 equiv.). Purification by flash column chromatography (Biotage Isolera™, SNAP 5 g silica cartridge, 0–5% methanol in EtOAc) gave **4** (44 mg, 80%) as a colorless oil.

Using **General procedure A** and the **Work-up Procedure**, phosphonate **4** was isolated in 34% yield (19 mg).

R*f* = 0.4 (EtOAc, $KMnO_4$)

NMR Spectroscopy (*see spectra*):

> **^{1}H NMR** (600 MHz, $CDCl_3$) δ_H 3.73 (d, *J* = 10.5 Hz, 6H), 1.89 (dtt, *J* = 20.8, 7.2, 5.0 Hz, 1H), 1.65 (dd, *J* = 14.2, 6.9 Hz, 2H), 1.57 (dt, *J* = 13.9, 6.9 Hz, 2H), 1.47 (dq, *J* = 28.9, 6.7 Hz, 4H), 1.40 – 1.27 (m, 14H) ppm.
>
> **^{13}C NMR** (151 MHz, $CDCl_3$) δ_C 52.5 (d, *J* = 6.0 Hz), 31.6 (d, *J* = 138.9 Hz), 24.2, 24.2 (d, *J* = 4.5 Hz), 23.7 (d, *J* = 4.5 Hz), 23.7, 22.9 (d, *J* = 9.0 Hz) ppm.
>
> **^{31}P NMR** (162 MHz, $CDCl_3$) δ_P 38.8 (s) ppm.

Dimethyl pentylphosphonate (5)

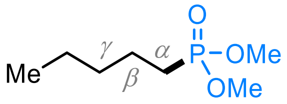

Prepared following **General Procedure A** and the **Work-up Procedure**, using pentane (72 mg, 115 μL, 1.00 mmol, 5.00 equiv.). Purification by flash column chromatography (Biotage Isolera™, SNAP 5 g silica cartridge, 0–5% methanol in EtOAc) gave **5** (27 mg, 74%) as a colorless oil. The r.r. (*α:β:γ = 67:23:10*) was determined by ^{31}P NMR analysis of the crude reaction mixture before the KF work-up (*see spectrum*). Only the major product (*α* isomer) is listed for characterisation.

R*f* = 0.4 (EtOAc, $KMnO_4$).

NMR Spectroscopy (*see spectra*):

^{1}**H NMR** (400 MHz, $CDCl_3$) δ_H 3.74 (d, *J* = 10.7 Hz, 6H), 1.78 – 1.69 (m, 2H), 1.39 – 1.25 (m, 5H), 0.99 – 0.93 (m, 1H), 0.90 (t, *J* = 6.9 Hz, 3H) ppm.

^{13}**C NMR** (151 MHz, $CDCl_3$) δ_C 52.4 (d, *J* = 7.6 Hz), 32.9 (d, *J* = 16.6 Hz), 24.8 (d, *J* = 140.4 Hz), 22.3, 22.1 (d, *J* = 4.5 Hz), 14.0 ppm.

^{31}**P NMR** (162 MHz, $CDCl_3$) δ_P 35.9 (s) ppm.

All recorded spectroscopic data matched those previously reported in the literature.[8]

Dimethyl (2,3-dimethylbutyl)phosphonate (6)

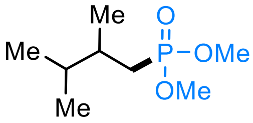

Prepared following **General Procedure C** and the **Work-up Procedure**, using 2,3-dimethylbutane (86.0 mg, 130 μL, 1.00 mmol, 5.00 equiv.). Purification by flash column chromatography (98:2 EtOAc/MeOH) gave **6** (14 mg, 36%) as a colorless oil. The r.r. (*>97:3*) was determined by ^{31}P NMR analysis of the crude reaction mixture before the KF work-up (*see spectrum*).

R*f* = 0.70 (95/5 EtOAc/MeOH, $KMnO_4$).

NMR Spectroscopy (*see spectra*):

^{1}**H NMR** (400 MHz, $CDCl_3$) δ_H 3.74 (d, *J* = 1.0 Hz, 3H), 3.71 (d, *J* = 1.0 Hz, 3H), 1.88 – 1.77 (m, 2H), 1.68 – 1.59 (m, 1H), 1.58 – 1.45 (m, 1H), 0.97 (d, *J* = 7.1 Hz, 3H), 0.86 (d, *J* = 6.9 Hz, 3H), 0.82 (d, *J* = 6.9 Hz, 3H) ppm;

^{13}**C NMR** (101 MHz, $CDCl_3$) δ_C 52.2 (d, *J* = 6.6 Hz), 52.1 (d, *J* = 6.6 Hz), 33.5 (d, *J* = 4.3 Hz), 33.3 (d, *J* = 15.2 Hz), 28.9 (d, *J* = 139.2 Hz), 19.3, 17.9, 16.6 (d, *J* = 4.9 Hz) ppm;

^{31}**P NMR** (162 MHz, $CDCl_3$) δ_P 35.9 (s) ppm.

IR (film) $\nu_{max:}$: 2957, 2876, 2852, 1379, 1232, 1027, 843, 811 cm^{-1}.

HRMS (EI) m/z calc'd for $C_8H_{19}O_3P$ [M-Me]$^+$, 179.0832; found, 179.0829.

Dimethyl (2,4-dimethylpentyl)phosphonate (7)

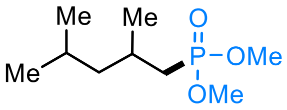

Prepared following **General Procedure C** and the **Work-up Procedure**, using 2,4-dimethylpentane (100 mg, 143 µL, 1.0 mmol, 5.0 equiv.). Purification by flash column chromatography (98:2 EtOAc/MeOH) gave **7** (13 mg, 31%) as a colorless oil. The r.r. (>*97:3*) was determined by ^{31}P NMR analysis of the crude reaction mixture (*see spectrum*).

R$_f$ = 0.70 (95/5 EtOAc/MeOH, KMnO$_4$).

NMR Spectroscopy (*see spectra*):

^{1}**H NMR** (600 MHz, Acetone-d_6) δ_H 3.67 (s, 3H), 3.65 (s, 3H), 1.96 – 1.88 (m, 1H), 1.73 (ddd, J = 19.4, 15.3, 4.7 Hz, 1H), 1.68 – 1.63 (m, 1H), 1.53 (ddd, J = 17.6, 15.4, 8.5 Hz, 1H), 1.28 (dt, J = 14.0, 7.1 Hz, 1H), 1.14 – 1.06 (m, 2H), 1.01 (d, J = 6.6 Hz, 3H), 0.87 (t, J = 6.2 Hz, 6H) ppm;

^{13}**C NMR** (151 MHz, Acetone-d_6) δ_C 52.5 (d, J = 6.7 Hz), 52.5 (d, J = 6.7 Hz), 48.9 (d, J = 13.8 Hz), 32.7 (d, J = 138.2 Hz), 27.1 (d, J = 4.2 Hz), 26.2, 23.7, 22.8, 21.6 (d, J = 6.7 Hz) ppm.

^{31}**P NMR** (162 MHz, Acetone-d_6) δ_P 33.3 (s) ppm.

IR (film) ν_{max}: 2955, 2872, 1467, 1242, 1026, 873, 800 cm^{-1}.

HRMS (EI) m/z calc'd for $C_9H_{21}O_3P$ [M-Me]$^+$, 193.0988; found, 193.0985.

2-Hydroxyphenyl methyl (2,2,4,4-tetramethylpentyl)phosphonate (8)

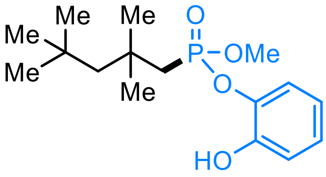

Prepared following **General Procedure A**, using 2,2,4,4-tetramethylpentane (256 mg, 356 µL, 2.0 mmol, 10.0 equiv.). Purification by flash column chromatography (50:50 hexane/EtOAc) gave **8** (31 mg, 49%) as a colorless oil. The r.r. (>*97:3*) was determined by ^{31}P NMR analysis of the crude reaction mixture (*see spectrum*).

Note: Product **8** was isolated as the mixed methyl-catechol phosphonate ester because incomplete methanolysis was observed when submitted to the Work-up Procedure.

R$_f$ = 0.50 (50/50 hexane/EtOAc, KMnO$_4$).

NMR Spectroscopy (*see spectra*):

^{1}**H NMR** (400 MHz, $CDCl_3$) δ_H δ_H 7.11 – 7.06 (m, 1H), 7.04 (dd, J = 8.1, 2.1 Hz, 1H), 7.00 (dt, J = 8.1, 1.5 Hz, 1H), 6.87 – 6.81 (m, 1H), 3.75 (d, J = 11.1 Hz, 3H), 2.17 – 2.02 (m, 2H), 1.52 – 1.43 (m, 2H), 1.24 (s, 3H), 1.23 (s, 3H), 0.99 (s, 9H) ppm;

^{13}**C NMR** (101 MHz, $CDCl_3$) δ_C 148.0 (d, J = 3.0 Hz), 139.0 (d, J = 9.6 Hz), 126.7, 122.0 (d, J = 4.5 Hz), 121.0, 120.0, 55.8 (d, J = 11.3 Hz), 53.3 (d, J = 7.6 Hz), 39.0 (d, J = 133.5 Hz), 34.7 (d, J = 3.8 Hz), 32.5, 32.1, 30.2 (d, J = 7.7 Hz) ppm.

^{31}**P NMR** (162 MHz, $CDCl_3$) δ_P 35.9 (s) ppm.

IR (film) ν_{max}: 3169, 2953, 2874, 1291, 1230, 1175, 1101, 1043, 936, 828, 752 cm^{-1}.

HRMS (ESI) m/z calc'd for $C_{16}H_{27}O_4P$ $[M+H]^+$, 315.1720; found, 315.1712.

Dimethyl (3-chlorobutyl)phosphonate (9)

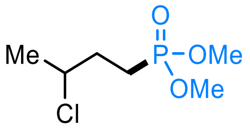

Prepared following **General Procedure A** and the **Work-up Procedure**, using 2-chlorobutane (93.0 mg, 106 µL, 1.00 mmol, 5.00 equiv.). Purification by flash column chromatography (Biotage Isolera™, SNAP 5 g silica cartridge, 0–5% methanol in EtOAc) gave **9** (28 mg, 63%) as a colorless oil. The r.r. (> *97:3*) was determined by ^{31}P NMR analysis of the crude reaction mixture before the KF work-up (*see spectrum*).

R*f* = 0.4 (EtOAc, $KMnO_4$)

NMR Spectroscopy (*see spectra*):

^{1}**H NMR** (400 MHz, $CDCl_3$) δ_H 4.14 – 4.02 (m, 1H), 3.75 (d, J = 10.8 Hz, 6H), 2.12 – 1.98 (m, 2H), 1.95 – 1.82 (m, 2H), 1.53 (d, J = 6.5 Hz, 3H) ppm;

^{13}**C NMR** (101 MHz, $CDCl_3$) δ_C 58.5 (d, J = 18.5 Hz), 52.6 (d, J = 4.1 Hz), 52.5 (d, J = 3.7 Hz), 33.1 (d, J = 4.1 Hz), 25.2, 22.1 (d, J = 142.7 Hz) ppm;

^{31}**P NMR** (162 MHz, $CDCl_3$) δ_P 34.5 (s) ppm.

IR (film) ν_{max}: 3459, 2955, 1875, 1447, 1259, 1026, 818, 750, 536 cm^{-1}.

HRMS (ESI$^+$) m/z calc'd for $C_6H_{14}O_3P$ $[M-Cl]^+$: 165.0675, found: 165.0671.

Dimethyl (4-chlorobutyl)phosphonate (10)

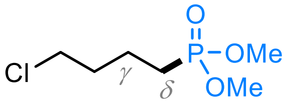

Prepared following **General Procedure A** and the **Work-up Procedure**, using 1-chlorobutane (93.0 mg, 105 µL, 1.00 mmol, 5.00 equiv.). Purification by flash column chromatography (Biotage Isolera™, SNAP 5 g silica

cartridge, 0–5% methanol in EtOAc) gave **10** (25 mg, 50%) as a colorless oil. The r.r. (*δ*:*γ* = *89:11*) was determined by ^{31}P NMR analysis of the crude reaction mixture before the KF work-up (see spectrum). Only the major product (*δ* isomer) is listed for characterisation.

R*f* = 0.4 (EtOAc, $KMnO_4$)

NMR Spectroscopy (see spectra):

^{1}**H NMR** (400 MHz, $CDCl_3$) δ_H 3.75 (d, *J* = 10.6 Hz, 6H), 3.55 (t, *J* = 6.2 Hz, 2H), 1.92 – 1.83 (m, 2H), 1.82 – 1.70 (m, 4H) ppm;

^{13}**C NMR** (151 MHz, $CDCl_3$) δ_C 52.5 (d, *J* = 6.7 Hz), 44.3, 33.1 (d, *J* = 15.9 Hz), 24.1 (d, *J* = 141.8 Hz), 20.0 (d, *J* = 5.1 Hz) ppm;

^{31}**P NMR** (162 MHz, $CDCl_3$) δ_P 34.7 (s) ppm.

IR (film) $\nu_{max:}$ 2955, 1875, 1459, 1260, 1027, 818, 749, 538 cm^{-1}.

HRMS (ESI$^+$) calcd for $C_6H_{15}ClO_3P$ [M+H]$^+$: 201.0442, found: 201.0436.

2-Hydroxyphenyl methyl (3-chloro-2,2-dimethylpropyl)phosphonate (11)

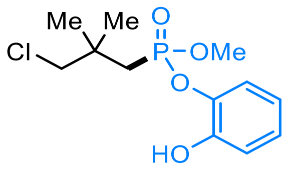

Prepared following **General Procedure A**, using methyl 1-chloro-2,2-dimethylpropane (107 mg, 1.00 mmol, 5.00 equiv.). Purification by flash column chromatography (Biotage Isolera™, SNAP 5 g silica cartridge, 10-100% EtOAc in pentane) gave **11** (26 mg, 45%) as a colorless oil. The r.r. (> *97:3*) was determined by ^{31}P NMR analysis of the crude reaction mixture (see spectrum).

Note: Product **11** was isolated as the mixed methyl-catechol phosphonate ester because a significant reduction in isolated yield was observed when submitted to the Work-up Procedure.

R*f* = 0.7 (1:1 EtOAc/pentane, CAM).

NMR Spectroscopy (see spectra):

^{1}**H NMR** (400 MHz, $CDCl_3$) δ_H δ 8.46 (s, 1H), 7.15 – 6.99 (m, 3H), 6.85 (m, 1H), 3.77 (d, *J* = 11.1 Hz, 3H), 3.53 (d, *J* = 1.2 Hz, 2H), 2.15 (d, *J* = 19.1 Hz, 2H), 1.24 (s, 6H) ppm;

^{13}**C NMR** (101 MHz, $CDCl_3$) δc 147.8 (d, *J* = 3.1 Hz), 138.8 (d, *J* = 10.0 Hz), 126.8 (d, *J* = 1.8 Hz), 121.9 (d, *J* = 4.9 Hz), 121.0, 120.0, 55.7 (d, *J* = 11.8 Hz), 53.6 (d, *J* = 7.5 Hz), 35.0 (d, *J* = 2.5 Hz), 33.6 (d, *J* = 137.9 Hz), 26.6 (d, *J* = 8.1 Hz) ppm;

^{31}**P NMR** (162 MHz, $CDCl_3$) δ_P 34.2 (s) ppm.

IR (film) $\nu_{max:}$ 2956, 1726, 1594, 1514, 1459, 1371, 1294, 1174, 1100, 1038, 938, 828, 752, 571 cm^{-1}.

HRMS (ESI) m/z calcd for $C_{12}H_{18}ClO_4P$ [M+H]$^+$: 293.0704, found: 293.0705

Dimethyl (2,2-dimethylpent-4-en-1-yl)phosphonate (12)

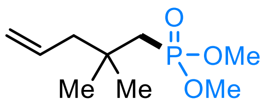

Prepared following **General Procedure A** and the **Work-up Procedure**, using 4,4-dimethyl-1-pentene (98.0 mg, 143 μL, 1.00 mmol, 5.00 equiv.). Purification by flash column chromatography (Biotage Isolera™, SNAP 5 g silica cartridge, 0–5% methanol in EtOAc) gave **12** (12 mg, 30%) as a colorless oil. The r.r. (> *97:3*) was determined by ^{31}P NMR analysis of the crude reaction mixture before the KF work-up (*see spectrum*).

R*f* = 0.4 (EtOAc, $KMnO_4$)

NMR Spectroscopy (*see spectra*):

^{1}H NMR (400 MHz, $CDCl_3$): δ_H 5.87 – 5.70 (m, 1H), 5.10 – 5.00 (m, 2H), 3.70 (d, *J* = 10.9 Hz, 6H), 2.10 (d, *J* = 7.3 Hz, 2H), 1.07 (s, 6H) ppm;

^{13}C NMR (101 MHz, $CDCl_3$): δ_C 134.9, 118.2, 52.0 (d, *J* = 4.0 Hz), 48.1 (d, *J* = 7.1 Hz), 32.7 (d, *J* = 2.0 Hz), 28.8 (d, *J* = 135.3 Hz), 28.2 ppm.

^{31}P NMR (162 MHz, $CDCl_3$) δ_P 34.0 (s) ppm.

IR (film) $\nu_{max:}$ 3187, 2955, 1730, 1457, 1275, 1260, 1037, 1028, 764, 750, 552 cm^{-1}.

HRMS (ESI$^+$) calcd for $C_9H_{20}O_3P$ $[M+H]^+$: 207.1145, found: 207.1143.

Dimethyl (2,2-dimethyl-3-phenylpropyl)phosphonate (13)

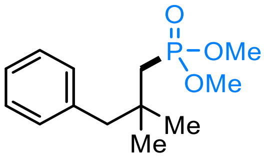

Prepared following **General Procedure A** and the **Work-up Procedure**, using Neopentylbenzene (148 mg, 172 μL, 1.00 mmol, 5.00 equiv.). Purification by flash column chromatography (Biotage Isolera™, SNAP 5 g silica cartridge, 0–5% methanol in EtOAc) gave **13** (24 mg, 47%) as a colorless oil. The r.r. (> *97:3*) was determined by ^{31}P NMR analysis of the crude reaction mixture before the KF work-up (*see spectrum*).

Using **General Procedure C** and the **Work-up Procedure**, phosphonate **17** was isolated in 35% yield (17 mg).

R*f* = 0.4 (EtOAc, $KMnO_4$)

NMR Spectroscopy (*see spectra*):

^{1}H NMR (400 MHz, $CDCl_3$) δ_H 7.30 – 7.25 (m, 2H), 7.24 – 7.20 (m, 1H), 7.19 – 7.14 (m, 2H), 3.71 (d, *J* = 10.9 Hz, 6H), 2.68 (d, *J* = 1.1 Hz, 2H), 1.72 (d, *J* = 18.9 Hz, 2H), 1.09 (s, 6H) ppm;

^{13}C NMR (126 MHz, $CDCl_3$) δ_C 138.5, 130.9, 128.0, 126.3, 52.1 (d, *J* = 7.6 Hz), 49.6 (d, *J* = 11.3 Hz), 36.2 (d, *J* = 137.3 Hz), 33.5 (d, *J* = 3.8 Hz), 28.2 (d, *J* = 7.5 Hz) ppm.

^{31}P NMR (162 MHz, $CDCl_3$) δ_P 39.6 (s) ppm.

IR (film) ν_{max}: 3051, 2928, 2365, 1500, 1469, 1275, 1260, 1183, 1038, 1028, 964, 764, 749, 542 cm^{-1}.

HRMS (ESI$^+$) calcd for $C_{13}H_{22}O_3P$ [M+H]$^+$: 257.1301, found: 257.1301.

Dimethyl (2-methyl-2-phenylpropyl)phosphonate (14)

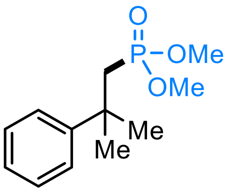

Prepared following **General Procedure A** and the **Work-up Procedure**, using tert-butylbenzene (134 mg, 155 µL, 1.00 mmol, 5.00 equiv.). Purification by flash column chromatography (Biotage Isolera™, SNAP 5 g silica cartridge, 0–5% methanol in EtOAc) gave **14** (24 mg, 50%) as a colorless oil.

R*f* = 0.4 (EtOAc, $KMnO_4$)

NMR Spectroscopy (*see spectra*):

> ^{1}**H NMR** (400 MHz, $CDCl_3$) δ_H 7.43 – 7.36 (m, 2H), 7.32 (ddd, *J* = 8.2, 5.8, 2.0 Hz, 2H), 7.23 – 7.15 (m, 1H), 3.46 (d, *J* = 2.6 Hz, 3H), 3.44 (d, *J* = 2.6 Hz, 3H), 2.19 (dd, *J* = 18.5, 2.6 Hz, 2H), 1.52 (s, 6H) ppm;
>
> ^{13}**C NMR** (101 MHz, $CDCl_3$) δ_C 148.6 (d, *J* = 8.1 Hz), 128.32, 126.20, 125.74, 51.9 (d, *J* = 6.1 Hz), 39.9 (d, *J* = 137.4 Hz), 36.1 (d, *J* = 3.0 Hz), 30.0 (d, *J* = 8.1 Hz) ppm;
>
> ^{31}**P NMR** (162 MHz, $CDCl_3$) δ_P 32.1 (s) ppm.

IR (film) ν_{max}: 3058, 2929, 1733, 1501, 1445, 1275, 1260, 1183, 1057, 1028, 842, 793, 749, 699, 543 cm^{-1}.

HRMS (ESI$^+$) calcd for $C_{12}H_{19}O_3P$ [M]$^+$:242.1066, found: 242.1061.

Dimethyl (2-methyl-2-(4-(trifluoromethyl)phenyl)propyl)phosphonate (15)

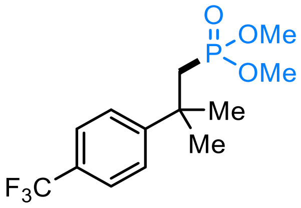

Prepared following **General Procedure A** and the **Work-up Procedure**, using 1-(*tert*-butyl)-4-(trifluoromethyl)benzene (202 mg, 189 µL, 1.00 mmol, 5.00 equiv.). Purification by flash column chromatography (98:2 EtOAc/MeOH) gave **15** (25 mg, 40%) as a colorless oil.

R*f* = 0.50 (95/5 EtOAc/MeOH, $KMnO_4$).

NMR Spectroscopy (*see spectra*):

> ^{1}**H NMR** (400 MHz, Methanol-*d*$_4$) δ_H 7.67 – 7.60 (m, 4H), 3.49 (d, *J* = 10.9 Hz, 6H), 2.33 (d, *J* = 18.5 Hz, 2H), 1.54 (s, 6H) ppm;

^{13}**C NMR** (101 MHz, Methanol-d_4) δ_C 154.0 (d, J = 6.6 Hz), 129.4 (q, J = 32.4 Hz), 127.7, 126.0 (q, J = 3.9 Hz), 125.8 (q, J = 270.8 Hz), 52.6 (d, J = 6.9 Hz), 39.3 (d, J = 138.4 Hz), 37.4 (d, J = 3.2 Hz), 30.4 (d, J = 9.4 Hz) ppm.

^{31}**P NMR** (162 MHz, Methanol-d_4) δ_P 31.8 (s) ppm.

^{19}**F NMR** (377 MHz, Methanol-d_4) δ_F -63.88 ppm.

IR (film) ν_{max}: 2969, 1618, 1460, 1410, 1328, 1167, 1123, 1068, 1015, 841 cm^{-1}.

HRMS (ESI) m/z calc'd for $C_{13}H_{18}F_3O_3P$ $[M+H]^+$, 311.1018; found, 311.1016.

Dimethyl (2-(4-cyanophenyl)propyl)phosphonate (16)

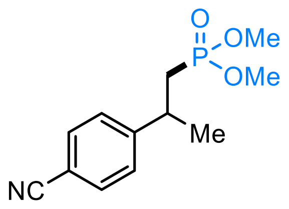

Prepared following **General Procedure A** and the **Work-up Procedure**, using MeCN/ClCH$_2$CN (9:1, 0.1 M) as solvent, 4-isopropylbenzonitrile (145.0 mg, 137.0 µL, 1.00 mmol, 5.00 equiv.). Purification by flash column chromatography (98:2 EtOAc/MeOH) gave **16** (14 mg, 26%) as a white solid. The r.r. (>97:3) was determined by ^{31}P NMR analysis of the crude reaction mixture before the KF work-up (*see spectrum*).

R*f* = 0.50 (95/5 EtOAc/MeOH, KMnO$_4$).

NMR Spectroscopy (*see spectra*):

^{1}**H NMR** (400 MHz, CDCl$_3$) δ_H 7.60 (d, J = 8.4 Hz, 2H), 7.34 (d, J = 8.3 Hz, 2H), 3.65 (d, J = 10.9 Hz, 3H), 3.57 (d, J = 10.8 Hz, 3H), 3.26 (dq, J = 11.1, 7.1 Hz, 1H), 2.06 (ddd, J = 18.3, 7.2, 3.2 Hz, 2H), 1.38 (d, J = 7.0 Hz, 3H) ppm;

^{13}**C NMR** (151 MHz, CDCl$_3$) δ_C 151.9 (d, J = 11.1 Hz), 132.5, 127.7, 119.0, 110.6, 52.4 (d, J = 6.5 Hz), 52.3 (d, J = 6.7 Hz), 35.0 (d, J = 3.6 Hz), 33.1 (d, J = 140.1 Hz), 23.3 (d, J = 10.4 Hz) ppm.

^{31}**P NMR** (162 MHz, CDCl$_3$) δ_P 33.1 (s) ppm.

IR (film) ν_{max}: 2955, 2852, 2227, 1607, 1506, 1456, 1411, 1231, 1025, 838, 808, cm^{-1}.

HRMS (ESI) m/z calc'd for $C_{12}H_{16}NO_3P$ $[M+H]^+$, 254.0941; found, 254.0940.

Dimethyl ((1,4,4-trimethyl-1,2,3,4-tetrahydronaphthalen-1-yl)methyl)phosphonate (17)

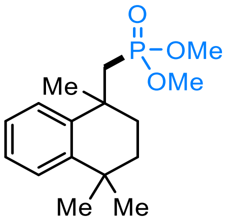

Prepared following **General Procedure C** and the **Work-up Procedure**, using 1,1,4,4-tetramethyl-1,2,3,4-tetrahydronaphthalene (188 mg, 198 µL, 1.00 mmol, 5.00 equiv.). Purification by flash column chromatography (Biotage Isolera™, SNAP 5 g silica cartridge, 0–5% methanol in EtOAc) gave **17** (8 mg, 14%) as a yellow oil. The r.r. (> 97:3) was determined by ^{31}P NMR analysis of the crude reaction mixture before the KF work-up (see spectrum).

R*f* = 0.4 (EtOAc, $KMnO_4$)

NMR Spectroscopy (see spectra):

^{1}H NMR (600 MHz, $CDCl_3$): δ_H 7.34 – 7.30 (m, 1H), 7.29 – 7.26 (m, 1H), 7.14 (qd, *J* = 7.1, 3.7 Hz, 2H), 3.62 (d, *J* = 10.9 Hz, 3H), 3.55 (d, *J* = 10.8 Hz, 3H), 2.32 (ddd, *J* = 12.9, 7.7, 4.5 Hz, 1H), 2.29 – 2.12 (m, 2H), 1.76 (ddd, *J* = 17.6, 7.5, 4.5 Hz, 1H), 1.72 – 1.68 (m, 2H), 1.47 (s, 3H), 1.30 (d, *J* = 18.2 Hz, 6H) ppm;

^{13}C NMR (151 MHz, $CDCl_3$): δ_C 145.1, 143.3 (d, *J* = 15.1 Hz), 126.8 (d, *J* = 43.8 Hz), 126.1 (d, *J* = 71.0 Hz), 52.1 (d, *J* = 6.0 Hz), 51.9 (d, *J* = 9.1 Hz), 38.7 (d, *J* = 135.9 Hz), 36.2 (d, *J* = 2.4 Hz), 34.9, 34.2, 32.1, 32.0 (d, *J* = 3.0 Hz), 31.7, 31.0 (d, *J* = 8.0 Hz) ppm;

^{31}P NMR (162 MHz, $CDCl_3$) δ_P 32.2 (s) ppm.

IR (film) ν_{max}: 3058, 2954, 2190, 1741, 1459, 1243, 1026, 835, 758, 543 cm^{-1}.

HRMS (ESI$^+$) calcd for $C_{16}H_{25}O_3P$ $[M]^+$: 296.1536, found: 296.1531.

Dimethyl (2,2-dimethoxycyclopentyl)phosphonate (18)

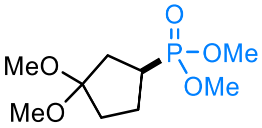

Prepared following **General Procedure B** and the **Work-up Procedure**, using cyclopentanone (84.0 mg, 1.00 mmol, 5.00 equiv.). Purification by flash column chromatography (Biotage Isolera™, SNAP 5 g silica cartridge, 0–5% methanol in EtOAc) gave **18** (12 mg, 25%) as a colorless oil. The r.r. (> 97:3) was determined by ^{31}P NMR analysis of the crude reaction mixture before the KF work-up (see spectrum).

R*f* = 0.5 (EtOAc, CAM)

NMR Spectroscopy (see spectra):

^{1}H NMR (400 MHz, $CDCl_3$) δ_H 3.76 (d, *J* = 1.2 Hz, 3H), 3.73 (d, *J* = 1.2 Hz, 3H), 3.21 (s, 3H), 3.17 (s, 3H),

2.35 (ddq, J = 16.9, 11.1, 8.4 Hz, 1H), 2.12 (ddd, J = 12.7, 8.2, 4.4 Hz, 1H), 1.98 – 1.80 (m, 5H) ppm;

^{13}C NMR (101 MHz, $CDCl_3$) δ_C 118.2 (d, J = 16.3 Hz), 52.8 (d, J = 3.4 Hz), 52.7 (d, J = 3.3 Hz), 50.2, 49.0, 34.9 (d, J = 2.6 Hz), 33.7 (d, J = 9.8 Hz), 32.3 (d, J = 149.9 Hz), 23.9 (d, J = 3.1 Hz) ppm;

^{31}P NMR (162 MHz, $CDCl_3$) δ_P 36.3 (s) ppm.

IR (film) $v_{max:}$ 3462, 2984, 2256, 1736, 1373, 1238, 1044, 915, 730, 608 cm^{-1}.

HRMS (ESI$^+$) calcd for $C_{17}H_{13}O_4P$ [M+H]$^+$: 193.0624, found: 193.0626.

Dimethyl (2-methyl-3-oxobutyl)phosphonate (19)

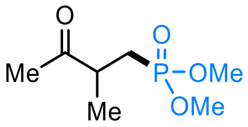

Prepared following **General Procedure C** and the **Work-up Procedure**, using 3-methyl-butan-2-one (86.0 mg, 1.00 mmol, 5.00 equiv.). Purification by flash column chromatography (Biotage Isolera™, SNAP 5 g silica cartridge, 0–5% methanol in EtOAc) gave **19** (18 mg, 42%) as a colorless oil. The r.r. (>*97:3*) was determined by ^{31}P NMR analysis of the crude reaction mixture before the KF work-up (*see spectrum*)**.**

Using **General Procedure A** and the **Work-up Procedure,** phosphonate **18** was isolated in 28% yield (12 mg).

Using **General Procedure B** and the **Work-up Procedure,** phosphonate **18** was isolated in 31% yield (13 mg).

R_f = 0.7 (1:10 Methanol/ EtOAc, CAM).

NMR Spectroscopy (*see spectra*):

^{1}H NMR (400 MHz, $CDCl_3$) δ_H 3.72 (d, J = 2.7 Hz, 3H), 3.69 (d, J = 2.7 Hz, 3H), 2.94 (dq, J = 12.0, 6.9 Hz, 1H), 2.30 (ddd, J = 18.2, 15.6, 7.0 Hz, 1H), 2.19 (s, 3H), 1.67 (ddd, J = 17.8, 15.6, 6.4 Hz, 1H), 1.23 (dd, J = 7.3, 1.0 Hz, 3H) ppm;

^{13}C NMR (101 MHz, $CDCl_3$) δ_C 210.1 (d, J = 9.6 Hz), 52.5 (d, J = 1.8 Hz), 52.4 (d, J = 1.7 Hz), 41.5 (d, J = 3.3 Hz), 28.4, 27.0 (d, J = 141.9 Hz), 18.3 (d, J = 10.6 Hz) ppm;

^{31}P NMR (162 MHz, $CDCl_3$) δ_P 34.69, 34.56 ppm.

IR (film) $v_{max:}$ 3454, 2957, 28533, 1713, 1459, 1264, 1025, 811, 730, 523 cm^{-1}.

HRMS (ESI$^+$) calcd for $C_7H_{15}O_4PNa$ [M+Na]+: 217.0600, found: 217.0597.

Dimethyl (2,2-dimethyl-3-oxobutyl)phosphonate (20)

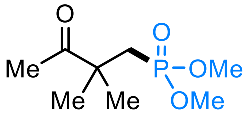

Prepared following **General Procedure A** and the **Work-up Procedure**, using pinacolone (100 mg, 125 µL, 1.00 mmol, 10.0 equiv.). Purification by flash column chromatography (Biotage Isolera™, SNAP 5 g silica

cartridge, 0–5% methanol in EtOAc) gave **20** (24 mg, 62%) as a colorless oil. The r.r. (> *97:3*) was determined by ^{31}P NMR analysis of the crude reaction mixture before the KF work-up (*see spectrum*).

R*f* = 0.1 (EtOAc, $KMnO_4$)

NMR Spectroscopy (*see spectra*):

^{1}H NMR (500 MHz, $CDCl_3$) δ_H 3.70 (d, *J* = 10.8 Hz, 6H), 2.19 (s, 3H), 2.10 (d, *J* = 18.2 Hz, 2H), 1.31 (s, 6H) ppm.

^{13}C NMR (126 MHz, $CDCl_3$) δ_C 212.1 (d, *J* = 6.3 Hz), 52.3 (d, *J* = 6.3 Hz), 45.5 (d, *J* = 2.5 Hz), 34.4 (d, *J* = 141.1 Hz), 26.0 (d, *J* = 10.1 Hz), 25.2 ppm.

^{31}P NMR (162 MHz, $CDCl_3$) δ_P 32.3 (s) ppm

IR (film) $\nu_{max:}$ 2921, 2852, 2188, 1784, 1632, 1462, 1200, 1075, 874, 709, 534 cm^{-1}.

HRMS (ESI$^+$) calcd for $C_8H_{17}O_4P$ [M]+: 208.0859, found: 208.0855.

Dimethyl (2,4-dimethyl-3-oxopentyl)phosphonate (21)

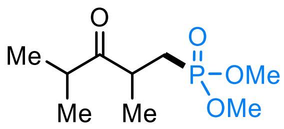

Prepared following **General Procedure A** and the **Work-up Procedure**, using 2,4-dimethyl-3-pentanone (114 mg, 141 μL, 1.00 mmol, 5.00 equiv.). Purification by flash column chromatography (Biotage Isolera™, SNAP 5 g silica cartridge, 0–5% methanol in EtOAc) gave **21** (27 mg, 56%) as a colorless oil. The r.r. (> 97:3) was determined by ^{31}P NMR analysis of the crude reaction mixture before the KF work-up (*see spectrum*).

R*f* = 0.1 (EtOAc, $KMnO_4$)

NMR Spectroscopy (*see spectra*):

^{1}H NMR (400 MHz, $CDCl_3$) δ_H 3.70 (d, *J* = 1.8 Hz, 3H), 3.68 (d, *J* = 1.7 Hz, 3H), 3.20 – 3.06 (m, 1H), 2.80 (p, *J* = 6.9 Hz, 1H), 2.30 (ddd, *J* = 18.1, 15.6, 7.7 Hz, 1H), 1.69 (ddd, *J* = 17.9, 15.6, 5.7 Hz, 1H), 1.20 (dt, *J* = 7.1, 1.0 Hz, 3H), 1.11 (dd, *J* = 6.9, 0.9 Hz, 6H).ppm;

^{13}C NMR (101 MHz, $CDCl_3$) δ_C 215.9 (d, *J* = 8.0 Hz), 52.5 (d, *J* = 6.4 Hz), 52.3 (d, *J* = 6.6 Hz), 39.7, 38.8 (d, *J* = 3.3 Hz), 27.3 (d, *J* = 140.8 Hz), 19.1 (d, *J* = 12.3 Hz), 18.7, 18.2.ppm;

^{31}P NMR (162 MHz, $CDCl_3$) δ_P 33.5 (s) ppm

IR (film) $\nu_{max:}$ 2955, 2451, 1703, 1455, 1176, 1014, 930, 849, 723, 696, 519 cm^{-1}.

HRMS (ESI$^+$) calcd for $C_9H_{19}O_4PNa$ [M+Na]$^+$: 245.0913, found: 245.0902.

Dimethyl (3-oxopentyl)phosphonate (22)

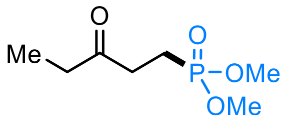

Prepared following **General Procedure A** and the **Work-up Procedure**, using 3-pentanone (86.0 mg, 106 µL, 1.00 mmol, 5.00 equiv.). Purification by flash column chromatography (Biotage Isolera™, SNAP 5 g silica cartridge, 0–5% methanol in EtOAc) gave **22** (11 mg, 28%) as a colorless oil. The r.r. (> *97:3*) was determined by ^{31}P NMR analysis of the crude reaction mixture before the KF work-up (*see spectrum*).

Prepared following **General Procedure C** and the **Work-up Procedure**, the phosphonate **22** was isolated in 21% yield (8 mg).

R*f* = 0.1 (EtOAc, $KMnO_4$)

NMR Spectroscopy (*see spectra*):

^{1}**H NMR** (400 MHz, $CDCl_3$) δ_H 3.73 (d, *J* = 10.7 Hz, 6H), 2.71 (dt, *J* = 11.8, 7.6 Hz, 2H), 2.46 (q, *J* = 7.4 Hz, 2H), 2.04 (dt, *J* = 17.8, 7.8 Hz, 2H), 1.08 (t, *J* = 7.3 Hz, 3H) ppm;

^{13}**C NMR** (101 MHz, $CDCl_3$) δ_C 208.6 (d, *J* = 14.1 Hz), 52.5 (d, *J* = 7.1 Hz), 35.8, 34.8 (d, *J* = 4.0 Hz), 18.3 (d, *J* = 144.4 Hz), 7.84 ppm;

^{31}**P NMR** (162 MHz, $CDCl_3$) δ_P 35.1 (s) ppm

IR (film) $\nu_{max:}$ 3423, 2955,1716, 1458, 1240, 1030, 818, 702, 521 cm^{-1}.

HRMS (ESI$^+$) calcd for $C_7H_{16}O_4P$ [M+H]$^+$: 195.0782, found: 195.0777.

Dimethyl (4-oxoheptyl)phosphonate (23)

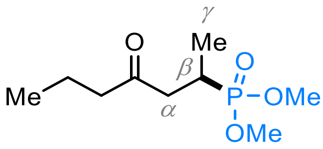

Prepared following **General Procedure A** and the **Work-up Procedure**, using 4-heptanone (114 mg, 140 µL, 1.00 mmol, 5.00 equiv.). Purification by flash column chromatography (Biotage Isolera™, SNAP 5 g silica cartridge, 0–5% methanol in EtOAc) gave **23** (12 mg, 27%) as a colorless oil. The r.r. (*β:γ* = *96:4*) was determined by ^{31}P NMR analysis of the crude reaction mixture before the KF work-up (*see spectrum*).

Using **General Procedure C** and the **Work-up Procedure**, phosphonate **23** was isolated in 20% yield (9 mg).

R*f* = 0.1 (EtOAc, $KMnO_4$)

NMR Spectroscopy (*see spectra*):

^{1}**H NMR** (600 MHz, $CDCl_3$) δ_H 3.78 – 3.64 (m, 6H), 2.82 (ddd, *J* = 16.7, 12.3, 3.7 Hz, 1H), 2.62 – 2.51 (m, 1H), 2.45 (dt, *J* = 16.2, 8.3 Hz, 1H), 2.41 – 2.33 (m, 2H), 1.65 – 1.60 (m, 2H), 1.15 (dd, *J* = 18.4, 7.1 Hz, 3H), 0.92 (t, *J* = 7.4 Hz, 3H) ppm;

^{13}C NMR (151 MHz, $CDCl_3$) δ_C 208.1 (d, J = 13.4 Hz), 52.9 (d, J = 6.0 Hz), 52.7 (d, J = 7.5 Hz), 45.3, 43.1 (d, J = 3.0 Hz), 25.5 (d, J = 145.0 Hz), 17.4, 13.9 (d, J = 4.5 Hz), 13.8 ppm;

^{31}P NMR (162 MHz, $CDCl_3$) δ_P 37.2 (s) ppm

IR (film) ν_{max}: 3249, 2959, 1713, 1459, 1259, 1028, 824, 749, 535 cm^{-1}.

HRMS (ESI$^+$) calcd for $C_9H_{20}O_4P$ [M+H]$^+$: 223.1094, found: 223.1086.

Dimethyl (2-methyl-4-oxo-4-phenylbutyl)phosphonate (24)

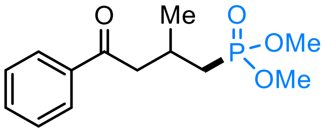

Prepared following **General Procedure C** and the **Work-up Procedure**, using 3-methyl-1-phenylbutan-1-one (162 mg, 167 µL, 1.0 mmol, 5.0 equiv.). Purification by flash column chromatography (98:2 EtOAc/MeOH) gave **24** (11 mg, 20%) as a white solid. The r.r. (>*97:3*) was determined by ^{31}P NMR analysis of the crude reaction mixture (*see spectrum*).

R*f* = 0.50 (95/5 EtOAc/MeOH, $KMnO_4$).

NMR Spectroscopy (*see spectra*):

^{1}H NMR (400 MHz, $CDCl_3$) δ_H 7.96 (d, J = 7.4 Hz, 2H), 7.56 (t, J = 7.3 Hz, 1H), 7.46 (t, J = 7.7 Hz, 2H), 3.74 (d, J = 5.6 Hz, 3H), 3.72 (d, J = 5.7 Hz, 3H), 3.26 (dd, J = 16.8, 6.0 Hz, 1H), 2.90 (dd, J = 16.8, 7.3 Hz, 1H), 2.62 (dp, J = 20.0, 6.7 Hz, 1H), 1.95 (ddd, J = 18.6, 15.4, 6.4 Hz, 1H), 1.79 (ddd, J = 18.6, 15.4, 7.0 Hz, 1H), 1.15 (d, J = 6.7 Hz, 3H) ppm;

^{13}C NMR (101 MHz, $CDCl_3$) δ_C 199.2, 137.1, 133.1, 128.6, 128.1, 52.3 (d, J = 2.2 Hz), 52.2 (d, J = 2.0 Hz), 45.8 (d, J = 10.6 Hz), 31.1 (d, J = 138.7 Hz), 25.0 (d, J = 4.1 Hz), 21.5 (d, J = 9.6 Hz) ppm.

^{31}P NMR (162 MHz, $CDCl_3$) δ_P 33.3 ppm.

IR (film) ν_{max}: 2954, 2851, 1736, 1683, 1597, 1581, 1449, 1218, 1055, 1028, 840, 755 cm^{-1}.

HRMS (ESI) m/z calc'd for $C_{13}H_{19}O_4P$ [M+Na]$^+$, 293.0913; found, 293.0919.

Methyl 3-(dimethoxyphosphoryl)propanoate (25)

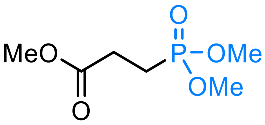

Prepared following **General Procedure A** and the **Work-up Procedure**, using 4-heptanone (114 mg, 140 µL, 1.00 mmol, 5.00 equiv.). Purification by flash column chromatography (Biotage Isolera™, SNAP 5 g silica cartridge, 0–5% methanol in EtOAc) gave **25** (9.0 mg, 23%) as a colorless oil. The r.r. (>*97:3*) was determined by ^{31}P NMR analysis of the crude reaction mixture before the KF work-up (*see spectrum*).

R_f = 0.1 (EtOAc, $KMnO_4$)

NMR Spectroscopy (see spectra):

^{1}H NMR (400 MHz, $CDCl_3$) δ_H 3.76 (s, 3H), 3.73 (s, 3H), 3.71 (s, 3H), 2.61 (dt, *J* = 12.4, 8.0 Hz, 2H), 2.18 – 2.01 (m, 2H) ppm;

^{13}C NMR (151 MHz, $CDCl_3$) δ_C 172.5 (d, *J* = 9.1 Hz), 52.7 (d, *J* = 6.0 Hz), 52.2, 27.3 (d, *J* = 6.0 Hz), 20.2 (d, *J* = 144.7 Hz) ppm;

^{31}P NMR (162 MHz, $CDCl_3$) δ_P 32.7 (s) ppm.

IR (film) ν_{max}: 2925, 1740, 1195, 1034, 487, 459, 409 cm^{-1}.

HRMS (ESI$^+$) calcd for $C_6H_{13}O_5PNa$ [M+Na]$^+$: 219.0393, found: 219.0388.

Methyl 3-(dimethoxyphosphoryl)-2-methylpropanoate (26)

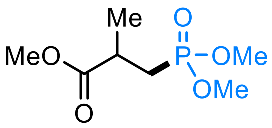

Prepared following **General Procedure A** and the **Work-up Procedure**, using methyl isobutyrate (102 mg, 114 μL, 1.00 mmol, 5.00 equiv.). Purification by flash column chromatography (Biotage Isolera™, SNAP 5 g silica cartridge, 0–5% methanol in EtOAc) gave **26** (17 mg, 40%) as a colorless oil. The r.r. (>*97:3*) was determined by ^{31}P NMR analysis of the crude reaction mixture before the KF work-up (see spectrum).

R_f = 0.2 (EtOAc, $KMnO_4$)

NMR Spectroscopy (see spectra):

^{1}H NMR (400 MHz, $CDCl_3$) δ_H 3.74 (d, *J* = 4 Hz, 3H), 3.71 (d, *J* = 4 Hz, 3H), 3.70 (s, 3H), 2.91-2.79 (m, 1H), 2.30 (ddd, *J* = 18.3, 15.5, 7.0 Hz, 1H), 1.82 (ddd, *J* = 18.0, 15.5, 6.8 Hz, 1H), 1.30 (dd, *J* = 7.1, 0.9 Hz, 3H) ppm;

^{13}C NMR (101 MHz, $CDCl_3$) δ_C 175.8 (d, *J* = 12.1 Hz), 52.6 (d, *J* = 2.0 Hz), 52.5 (d, *J* = 2.0 Hz), 52.23, 34.5 (d, *J* = 3.0 Hz), 28.4 (d, *J* = 142.4 Hz),18.8 (d, *J* = 10.0 Hz) ppm;

^{31}P NMR (162 MHz, $CDCl_3$) δ_P 31.9 (s) ppm.

IR (film) ν_{max}: 3451, 2956, 1734, 1451, 1214, 1026, 838, 527 cm^{-1}.

HRMS (ESI$^+$) calcd for $C_7H_{15}O_5PNa$ [M+Na]$^+$: 233.0549, found: 233.0544.

Methyl 4-(dimethoxyphosphoryl)-3-methylbutanoate (27)

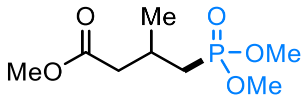

Prepared following **General Procedure A** and the **Work-up Procedure**, using methyl 3-methylbutanoate (116 mg, 1.00 mmol, 5.00 equiv.). Purification by flash column chromatography (Biotage Isolera™, SNAP 5 g silica cartridge, 0–10% methanol in EtOAc) gave **27** (16 mg, 36%) as a colorless oil. The r.r. (*>97:3*) was determined by ^{31}P NMR analysis of the crude reaction mixture (*see spectrum*).

R*f* = 0.4 (EtOAc, CAM).

NMR Spectroscopy (*see spectra*):

^{1}H NMR (400 MHz, $CDCl_3$) δ_H 3.74 (s, 3H), 3.71 (s, 3H), 3.66 (s, 3H), 2.49 (dd, *J* = 15.1, 6.0 Hz, 1H), 2.45 – 2.33 (m, 1H), 2.27 (ddd, *J* = 15.1, 7.1, 1H), 1.89 (ddd, *J* = 19.0, 15.4, 5.8 Hz, 1H), 1.69 (ddd, *J* = 18.6, 15.4, 7.5 Hz, 1H), 1.10 (d, J = 6.7 Hz, 3H) ppm;

^{13}C NMR (101 MHz, $CDCl_3$) δ_C 172.8, 52.39 (d, *J* = 6.8 Hz), 52.37 (d, *J* = 6.7 Hz), 51.7, 41.8 (d, *J* = 12.5 Hz), 31.1 (d, *J* = 139.4 Hz), 25.7 (d, *J* = 3.9 Hz), 21.2 (d, *J* = 9.0 Hz) ppm;

^{31}P NMR (162 MHz, $CDCl_3$) δ_P 33.7 (s) ppm.

IR (film) $\nu_{max:}$ 3455, 2956, 2852, 1733, 1438, 1208, 1026, 839, 547 cm^{-1}.

HRMS (EI$^+$) m/z calc'd for $C_8H_{17}O_5P$ $[M+H]^+$, 225.0886; found, 225.0882.

3-(Dimethoxyphosphoryl)-2-methylpropyl acetate (28)

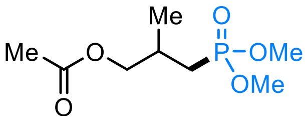

Prepared following **General Procedure A** and the **Work-up Procedure**, using isobutyl acetate (116 mg, 134 μL, 1.00 mmol, 5.00 equiv. Purification by flash column chromatography (Biotage Isolera™, SNAP 5 g silica cartridge, 0–5% methanol in EtOAc) gave **28** (18 mg, 40%) as a colorless oil. The r.r. (> *97:3*) was determined by ^{31}P NMR analysis of the crude reaction mixture before the KF work-up (*see spectrum*)**.**

R*f* = 0.4 (EtOAc, $KMnO_4$)

NMR Spectroscopy (*see spectra*):

^{1}H NMR (400 MHz, $CDCl_3$): δ_H 4.01 – 3.91 (m, 2H), 3.75 (d, *J* = 10.8 Hz, 6H), 2.26 (dd, *J* = 12.9, 6.8 Hz, 1H), 2.07 (s, 3H), 2.00 – 1.87 (m, 1H), 1.68 – 1.58 (m, 1H), 1.10 (d, *J* = 6.8 Hz, 3H) ppm;

^{13}C NMR (151 MHz, $CDCl_3$): δ_C 171.1, 69.1 (d, *J* = 15.1 Hz), 52.5 (d, *J* = 6.0 Hz), 52.4 (d, *J* = 6.0 Hz), 28.5 (d, *J* = 141.5 Hz), 28.3 (d, *J* = 4.1 Hz), 21.0, 18.0 (d, *J* = 7.6 Hz) ppm.

^{31}P NMR (162 MHz, $CDCl_3$) δ_P 33.3 (s) ppm.

IR (film) $\nu_{max:}$ 2957, 1734 1235, 1027, 840, 749, 547, 475 cm^{-1}.

HRMS calcd for $C_8H_{17}O_5PNa$ $[M+Na]^+$: 247.0706, found: 247.0695.

5-(Dimethoxyphosphoryl)-4-methylpentyl thiophene-3-carboxylate (29)

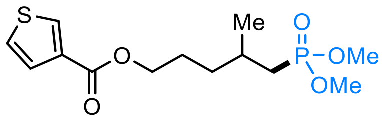

Prepared following **General Procedure B** and the **Work-up Procedure**, using 4-methylpentyl thiophene-3-carboxylate (212 mg, 1.00 mmol, 5.00 equiv.). Purification by flash column chromatography (Biotage Isolera™, SNAP 5 g silica cartridge, 0–5% methanol in EtOAc) gave **29** (16 mg, 25%) as a colorless oil. The r.r. (> *97:3*) was determined by ^{31}P NMR analysis of the crude reaction mixture before the KF work-up (*see spectrum*).

R*f* = 0.4 (EtOAc, $KMnO_4$)

NMR Spectroscopy (*see spectra*):

^{1}H NMR (400 MHz, $CDCl_3$) δ_H 8.10 (d, *J* = 3.1 Hz, 1H), 7.52 (d, *J* = 5.2 Hz, 1H), 7.35 – 7.27 (m, 1H), 4.26 (t, *J* = 6.6 Hz, 2H), 3.74 (s, 3H), 3.71 (s, 3H), 2.07 – 1.90 (m, 1H), 1.77 (ddd, *J* = 19.7, 9.2, 4.6 Hz, 3H), 1.69 – 1.52 (m, 2H), 1.44 – 1.31 (m, 1H), 1.08 (d, *J* = 6.7 Hz, 3H) ppm;

^{13}C NMR (101 MHz, $CDCl_3$) δ_C 163.0, 134.0, 132.7, 128.0, 126.1, 64.8, 52.4 (d, *J* = 5.0 Hz), 52.3 (d, *J* = 5.0 Hz), 34.5 (d, *J* = 13.6 Hz), 31.9 (d, *J* = 138.9 Hz), 28.2 (d, *J* = 4.1 Hz), 26.2, 20.9 (d, *J* = 7.9 Hz) ppm;

^{31}P NMR (162 MHz, $CDCl_3$) δ_P 34.8 (s) ppm.

IR (film) $\nu_{max:}$ 3359, 2919, 2850, 1720, 1467, 1260, 1035, 749 cm^{-1}.

HRMS calcd for $C_{13}H_{21}O_5PSNa$ $[M+Na]^+$: 343.0740, found: 343.0742.

Dimethyl (3-cyanopropyl)phosphonate (30)

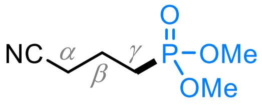

Prepared following **General Procedure A** and the **Work-up Procedure**, using butyronitrile (69.0 mg, 87.0 µL, 1.00 mmol, 5.00 equiv.). Purification by flash column chromatography (Biotage Isolera™, SNAP 5 g silica cartridge, 0–5% methanol in EtOAc) gave **30** (14 mg, 41%) as a colorless oil. The r.r. (*α:β:γ = 8:9:83*) was determined by ^{31}P NMR analysis of the crude reaction mixture before the KF work-up (*see spectrum*). Only the major product (*γ* isomer) is listed for characterisation.

Using **General Procedure C** and the **Work-up Procedure**, phosphonate **32** was isolated in 33% yield (11 mg).

R*f* = 0.3 (EtOAc, $KMnO_4$)

NMR Spectroscopy (*see spectra*):

^{1}H NMR (400 MHz, $CDCl_3$) δ_H 3.78 (d, *J* = 2.9 Hz, 3H), 3.76 (d, *J* = 3.1 Hz, 3H), 2.50 (q, *J* = 6.1 Hz, 2H), 2.05 – 1.84 (m, 4H) ppm;

^{13}**C NMR** (151 MHz, $CDCl_3$) δ_C 118.8, 52.7 (d, J = 6.0 Hz), 24.1 (d, J = 143.4 Hz), 19.2 (d, J = 4.5 Hz), 17.9 (d, J = 15.1 Hz) ppm;

^{31}**P NMR** (162 MHz, $CDCl_3$) δ_P 32.0 (s) ppm.

IR (film) $\nu_{max:}$ 3444, 2955, 2247, 1458, 1260, 1028, 818, 750, 525 cm^{-1}.

HRMS (ESI$^+$) calcd for $C_6H_{13}NO_3P$ [M+H]$^+$: 178.0628, found: 178.0620.

Dimethyl (4-cyano-2-methylbutyl)phosphonate (31)

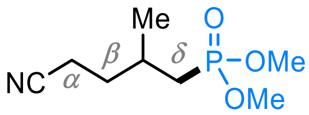

Prepared following **General Procedure A** and the **Work-up Procedure**, using isocapronitrile (97.0 mg, 121 µL, 1.00 mmol, 10.0 equiv.). Purification by flash column chromatography (Biotage Isolera™, SNAP 5 g silica cartridge, 0–5% methanol in EtOAc) gave **31** (16 mg, 39%) as a colorless oil. The r.r. (*δ:β* = *89:11*) was determined by ^{31}P NMR analysis of the crude reaction mixture (*see spectrum*). Only the major product (*δ* isomer) is listed for characterisation.

Using **General Procedure C** and the **Work-up Procedure**, phosphonate **31** was isolated in 22% yield (9 mg).

R*f* = 0.3 (EtOAc, $KMnO_4$)

NMR Spectroscopy (*see spectra*):

^{1}**H NMR** (400 MHz, $CDCl_3$) δ_H 3.75 (d, J = 10.8 Hz, 6H), 2.48 – 2.30 (m, 2H), 2.06 (tt, J = 13.5, 6.7 Hz, 1H), 1.96 – 1.85 (m, 1H), 1.82 – 1.59 (m, 3H), 1.10 (t, J = 7.4 Hz, 3H) ppm;

^{13}**C NMR** (151 MHz, $CDCl_3$) δ_C 119.5, 52.5 (d, J = 6.0 Hz), 33.1 (d, J = 10.6 Hz), 31.6 (d, J = 140.4 Hz), 27.9 (d, J = 4.5 Hz), 20.4 (d, J = 9.0 Hz), 15.0 ppm;

^{31}**P NMR** (162 MHz, CDCl3) δ_P 32.6 (s) ppm.

IR (film) $\nu_{max:}$ 3004, 2851, 2244, 1450, 1260, 1027, 749, 538 cm^{-1}.

HRMS (ESI$^+$) calcd for $C_8H_{17}NO_3P$ [M+H]$^+$: 206.0941, found: 206.0932.

Dimethyl (2-cyano-2-methylpropyl)phosphonate (32)

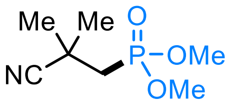

Prepared following **General Procedure C** and the **Work-up Procedure**, using trimethylacetonitrile (83.0 mg, 110 µL, 1.00 mmol, 5.00 equiv.). Purification by flash column chromatography (Biotage Isolera™, SNAP 5 g silica cartridge, 0–5% methanol in EtOAc) gave **32** (10 mg, 26%) as a colorless oil.

R*f* = 0.4 (EtOAc, $KMnO_4$)

NMR Spectroscopy (see spectra):

^{1}**H NMR** (400 MHz, $CDCl_3$): δ_H 3.80 (d, *J* = 11.0 Hz, 6H), 2.09 (d, *J* = 18.5 Hz, 2H), 1.54 (d, *J* = 1.0 Hz, 6H) ppm;

^{13}**C NMR** (101 MHz, $CDCl_3$): δ_C 124.1 (d, *J* = 9.1 Hz), 52.8 (d, *J* = 6.1 Hz), 36.1 (d, *J* = 143.4 Hz), 29.4 (d, *J* = 4.0 Hz), 28.2 (d, *J* = 8.1 Hz) ppm.

^{31}**P NMR** (162 MHz, $CDCl_3$) δ_P 26.8 (s) ppm.

IR (film) $\nu_{max:}$ 3457, 2955, 2237, 1459, 1242, 1030, 848, 731, 533 cm^{-1}.

HRMS (ESI^+) calcd for $C_6H_{11}NO_3P$ $[M\text{-}Me]^+$: 176.0471, found: 176.0467.

Dimethyl (2-methyl-4-((trifluoromethyl)sulfonamido)butyl)phosphonate (33)

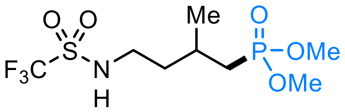

Prepared following **General Procedure A** and the **Work-up Procedure**, using 1,1,1-trifluoro-N-isopentylmethanesulfonamide (219 mg, 1.00 mmol, 5.00 equiv.). Purification by flash column chromatography (Biotage Isolera™, SNAP 5 g silica cartridge, 0–5% methanol in EtOAc) gave **33** (27 mg, 37%) as a colorless oil. The r.r. (> *97:3*) was determined by ^{31}P NMR analysis of the crude reaction mixture before the KF work-up (see spectrum).

R*f* = 0.6 (1:10 Methanol/ EtOAc, CAM).

NMR Spectroscopy (see spectra):

^{1}**H NMR** (400 MHz, $CDCl_3$) δ_H 7.80 (s, 1H), 3.74 (d, *J* = 6.7 Hz, 3H), 3.72 (d, *J* = 6.7 Hz, 3H), 3.39 – 3.22 (m, 2H), 2.07 (dq, *J* = 20.4, 6.5 Hz, 1H), 1.85 – 1.51 (m, 4H), 1.07 (dd, *J* = 6.8, 1.3 Hz, 3H) ppm;

^{13}**C NMR** (101 MHz, $CDCl_3$) δ_C 120.1 (q, *J* = 321.7 Hz), 52.7 (d, *J* = 6.8 Hz), 52.6 (d, *J* = 6.7 Hz), 41.8, 37.4 (d, *J* = 8.7 Hz), 31.2 (d, *J* = 138.9 Hz), 25.3 (d, *J* = 3.8 Hz), 21.6 (d, *J* = 12.3 Hz) ppm;

^{31}**P NMR** (162 MHz, $CDCl_3$) δ_P 34.8 (s) ppm.

^{19}**F NMR** (377 MHz, $CDCl_3$) δ_F -77.4 (s) ppm.

IR (film) $\nu_{max:}$ 3055, 2959, 2883, 1732, 1464, 1371, 1181, 1031, 905, 726, 604 cm^{-1}.

HRMS calcd for $C_8H_{17}O_5F_3NPS$ $[M+H]^+$: 328.0590, found: 328.0574.

2-Hydroxyphenyl methyl (1-(1,3-dioxoisoindolin-2-yl)butan-2-yl)phosphonate (34)

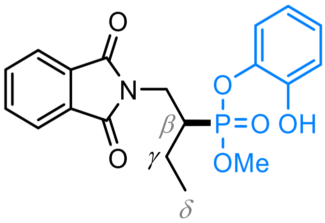

Prepared following **General Procedure A**, using MeCN/$ClCH_2CN$ (9:1, 0.1 M) as solvent, 2-butylisoindoline-1,3-dione (203 mg, 1.0 mmol, 5.0 equiv.). Purification by flash column chromatography (20:80 pentane/Et_2O) gave **34** (20 mg, 25%) as a white solid. The r.r. (*β:γ:δ* = 44:26:30) and d.r. (*d.r.* = 56:44) were determined by ^{31}P NMR analysis of the crude reaction mixture (*see spectrum*). Only the major product (*β* isomer) is listed for characterisation.

R_f = 0.30 (20:80 pentane/Et_2O, $KMnO_4$, CAM).

NMR Spectroscopy (*see spectra*):

^1H NMR (600 MHz, $CDCl_3$) δ_H (*56:44* ratio of diastereomers) 7.89 – 7.85 (m, 2H), 7.73 (ddd, *J* = 8.5, 5.4, 3.0 Hz, 2H), 7.23 (d, *J* = 8.1 Hz, 0.49H), 7.12 (d, *J* = 6.6 Hz, 0.51H), 7.09 – 7.04 (m, 1H), 7.03 (dd, *J* = 7.5, 1.7 Hz, 1H), 6.86 – 6.81 (m, 1H), 4.21 – 4.11 (m, 1H), 4.00 – 3.87 (m, 1H), 3.74 (d, *J* = 10.9 Hz, 3H), 3.60 (d, *J* = 10.9 Hz, 3H), 2.68 – 2.57 (m, 1H), 2.05 – 1.85 (m, 1H), 1.70 (dtd, *J* = 26.9, 14.7, 7.3 Hz, 1H), 1.17 (td, *J* = 7.5, 5.8 Hz, 3H) ppm;

^{13}C NMR (126 MHz, $CDCl_3$) δ_C (*56:44* ratio of diastereomers) 168.3, 147.7 (d, *J* = 3.2 Hz, *diast. 1*), 147.4 (d, *J* = 3.7 Hz, *diast. 2*), 138.6 (d, *J* = 10.2 Hz, *diast. 1*), 138.5 (d, *J* = 10.2 Hz, *diast. 2*), 134.3 (2 C), 131.9 (2 C), 126.6 (*diast. 1*), 126.4 (*diast. 2*), 123.6 (*diast. 1*), 123.5 (*diast. 2*), 121.8 (d, *J* = 4.0 Hz, *diast. 1*), 121.5 (d, *J* = 3.7 Hz, *diast. 2*), 120.7 (*diast. 1*), 120.6 (*diast. 2*), 119.5 (*diast. 1*), 119.0 (*diast. 2*), 53.8 (d, *J* = 3.5 Hz, *diast. 1*), 53.8 (d, *J* = 3.3 Hz, *diast. 2*), 36.9 (d, *J* = 3.1 Hz, *diast. 1*), 36.5 (*diast. 2*), 36.4 (d, *J* = 137.9 Hz, *diast. 1*), 36.3 (d, *J* = 138.4 Hz, *diast. 2*), 20.5 (d, *J* = 4.1 Hz, *diast. 1*), 20.2 (d, *J* = 4.2 Hz, *diast. 2*), 12.1 (d, *J* = 9.3 Hz, *diast. 1*), 11.9 (d, *J* = 8.1 Hz, *diast. 2*) ppm.

^{31}P NMR (162 MHz, $CDCl_3$) δ_P 32.8, 32.4 ppm.

IR (film) $\nu_{max:}$ 3456, 3016, 2970, 1738, 1728, 1436, 1366, 1229. 1217, 1206, 1036 cm^{-1}

HRMS (ESI) m/z calc'd for $C_{19}H_{20}NO_6P$ $[M+H]^+$, 390.1101; found, 390.1098.

2-Hydroxyphenyl methyl ((trimethylsilyl)methyl)phosphonate (35)

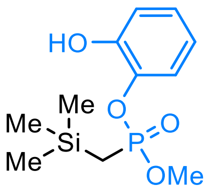

Prepared following **General Procedure A**, using tetramethylsilane (88.0 mg, 137 μL, 1.00 mmol, 5.00 equiv.). Purification by flash column chromatography (83:17 pentane/acetone) gave **35** (23 mg, 42%) as a white solid.

Note: Product **35** was isolated as the mixed methyl-catechol phosphonate ester because a significant reduction in isolated yield was observed when submitted to the Work-up Procedure.

R*f* = 0.45 (83:17 pentane/acetone, $KMnO_4$).

NMR Spectroscopy (*see spectra*):

^{1}**H NMR** (400 MHz, $CDCl_3$) δ_H 7.11 – 6.97 (m, 3H), 6.86 – 6.81 (m, 1H), 3.74 (d, *J* = 11.1 Hz, 3H), 1.36 (d, *J* = 22.6 Hz, 2H), 0.22 (s, 9H) ppm;

^{13}**C NMR** (151 MHz, $CDCl_3$) δ_C 148.0 (d, *J* = 3.0 Hz), 139.0 (d, *J* = 9.6 Hz), 126.5, 121.8 (d, *J* = 4.3 Hz), 120.7, 119.9, 53.2 (d, *J* = 7.4 Hz), 13.3 (d, *J* = 127.1 Hz), -0.4 (d, *J* = 3.9 Hz) ppm.

^{31}**P NMR** (162 MHz, $CDCl_3$) δ_P 38.3 ppm.

IR (film) ν_{max}: 2955, 2853, 1614, 1594, 1514, 1495, 1459, 1370, 1294, 1238, 1175, 1101, 1037, 936, 844, 827, 791, 752 cm^{-1}

HRMS (ESI) m/z calc'd for $C_{11}H_{19}O_4PSi$ $[M+H]^+$, 275.0863; found, 275.0859.

2-Hydroxyphenyl methyl ((dimethyl(phenyl)silyl)methyl)phosphonate (36)

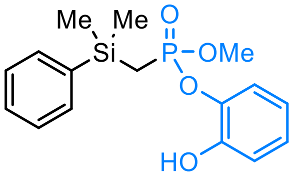

Prepared following **General Procedure A**, using MeCN/$ClCH_2CN$ (9:1, 0.1 M) as solvent, trimethyl(phenyl)silane (150 mg, 173 μL, 1.0 mmol, 5.0 equiv.). Purification by flash column chromatography (50:50 hexane/Et_2O) gave **36** (21 mg, 31%) as a colorless oil.

Using **General Procedure A** with 10 equiv. of trimethyl(phenyl)silane, phosphonate **36** was isolated in 22% yield (15 mg).

Note: Product **36** was isolated as the mixed methyl-catechol phosphonate ester because a significant reduction in isolated yield was observed when submitted to the Work-up Procedure.

R*f* = 0.20 (50/50 hexane/Et_2O, $KMnO_4$).

NMR Spectroscopy (*see spectra*)

^{1}**H NMR** (400 MHz, $CDCl_3$) δ_H 7.57 – 7.52 (m, 2H), 7.42 – 7.36 (m, 3H), 7.09 – 7.05 (m, 1H), 7.03 (dd, *J* = 8.1, 2.0 Hz, 1H), 6.92 (dt, *J* = 7.9, 1.5 Hz, 1H), 6.81 (tdd, *J* = 7.8, 2.0, 0.8 Hz, 1H), 3.63 (d, *J* = 11.3 Hz, 3H), 1.57 (d, *J* = 22.5 Hz, 2H), 0.54 (s, 3H), 0.50 (s, 3H) ppm;

^{13}**C NMR** (151 MHz, $CDCl_3$) δ_C 148.1 (d, *J* = 3.1 Hz), 139.0 (d, *J* = 9.6 Hz), 137.0 (d, *J* = 6.4 Hz), 133.6, 129.8, 128.1, 126.6, 121.9 (d, *J* = 4.3 Hz), 120.8, 119.9, 53.4 (d, *J* = 7.3 Hz), 13.3 (d, *J* = 127.3 Hz), -1.9 (d, *J* = 3.1 Hz), -2.0 (d, *J* = 2.1 Hz) ppm;

^{31}**P NMR** (162 MHz, $CDCl_3$) δ_P 37.8 ppm.

IR (film) ν_{max}: 3071, 2970, 2955, 1738, 1593, 1514, 1495, 1459, 1428, 1366, 1294, 1229, 1217, 1116, 1102, 1039, 937, 832, 819, 754, 736, 700 cm^{-1}

HRMS (ESI) m/z calc'd for $C_{16}H_{21}O_4PSi$ $[M+Na]^+$, 359.0839; found, 359.0841.

Methyl 2-((*tert*-butoxycarbonyl)amino)-5-(dimethoxyphosphoryl)-4-methylpentanoate (37)

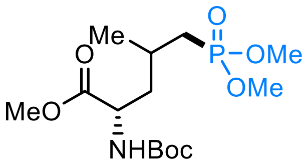

Prepared following **General Procedure A** and the **Work-up Procedure**, using methyl (*tert*-butoxycarbonyl)-*L*-leucinate (**37-S**, 245 mg, 1.00 mmol, 5.00 equiv.). Purification by flash column chromatography (Biotage Isolera™, SNAP 5 g silica cartridge, 0–5% methanol in EtOAc) gave **37** (15 mg, 20%) as a colorless oil. The r.r. (>*97:3*) and d.r. (*61:39*) were determined by ^{31}P NMR analysis of the crude reaction mixture before the KF work-up (*see spectrum*).

R*f* = 0.3 (EtOAc, $KMnO_4$)

NMR Spectroscopy (*see spectra*):

^{1}**H NMR** (400 MHz, $CDCl_3$) δ_H (*61:39* ratio of diastereomers) 5.08 (dd, *J* = 20.7, 8.8 Hz, 1H), 4.32 (d, *J* = 9.8 Hz, 1H), 3.73 (td, *J* = 5.6, 4.1 Hz, 9H), 2.13 – 2.01 (m, 1H), 1.94 – 1.75 (m, 2H), 1.74 – 1.59 (m, 2H), 1.44 (s, 9H), 1.17 – 1.05 (m, 3H) ppm;

^{13}**C NMR** (151 MHz, $CDCl_3$) δ_C 173.6, 155.8, 155.8, 80.1, 52.5, 51.9, 51.6, 40.7 (d, *J* = 13.6 Hz), 40.4 (d, *J* = 10.1 Hz), 29.8, 28.5, 25.5, 21.3 (d, *J* = 7.5 Hz), 20.9 (d, *J* = 9.1 Hz) ppm;

^{31}**P NMR** (162 MHz, $CDCl_3$) δ_P 33.41, 33.37 ppm.

IR (film) $\nu_{max:}$ 3005, 2923, 1725, 1462, 1260, 1027, 764, 403 cm^{-1}.

HRMS (ESI$^+$) calcd for $C_{14}H_{28}NO_7P$ $[M+Na]^+$: 376.1496, found: 376.1484.

Methyl 2-((*tert*-butoxycarbonyl)amino)-4-(dimethoxyphosphoryl)-3,3-dimethylbutanoate (38)

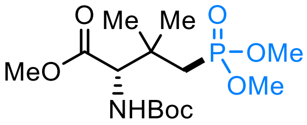

Prepared following **General Procedure A** and the **Work-up Procedure**, using methyl (*L*)-2-((*tert*-butoxycarbonyl)amino)-3,3-dimethylbutanoate (**38-S**, 245 mg, 1.00 mmol, 5.00 equiv.). Purification by flash column chromatography (Biotage Isolera™, SNAP 5 g silica cartridge, 0–5% methanol in EtOAc) gave **38** (17 mg, 25%) as a colorless oil.

R*f* = 0.3 (EtOAc, $KMnO_4$)

NMR Spectroscopy (*see spectra*):

^{1}**H NMR** (400 MHz, $CDCl_3$) δ_H 5.62 (d, *J* = 9.5 Hz, 1H), 4.20 (dd, *J* = 9.6, 1.0 Hz, 1H), 3.74 (d, *J* = 1.9 Hz, 3H), 3.74 (s, 3H), 3.71 (d, *J* = 1.7 Hz, 3H), 1.99 – 1.78 (m, 2H), 1.44 (s, 9H), 1.17 (d, *J* = 8.1 Hz, 6H) ppm;

^{13}**C NMR** (151 MHz, $CDCl_3$) δ_C 172.1, 155.8, 80.1, 68.2, 62.2 (d, *J* = 15.1 Hz), 52.3 (d, *J* = 6.0 Hz), 52.2 (d, *J* = 7.5 Hz), 52.1, 36.4, 33.4 (d, *J* = 140.4 Hz), 28.5, 25.2 (d, *J* = 4.4 Hz), 25.0 (d, *J* = 3.0 Hz) ppm;

^{31}**P NMR** (162 MHz, $CDCl_3$) δ_P 32.4 (s) ppm.

IR (film) $\nu_{max:}$ 2955, 2191, 1711, 1500, 1365, 1242, 1160, 1028, 848, 561 cm^{-1}.

HRMS (ESI^+) calcd for $C_{14}H_{28}NO_7P$ $[M+Na]^+$: 376.1496, found: 376.1491.

5-(Dimethoxyphosphoryl)-4-methylpentyl 2-(4-chlorophenoxy)-2-methylpropanoate (39)

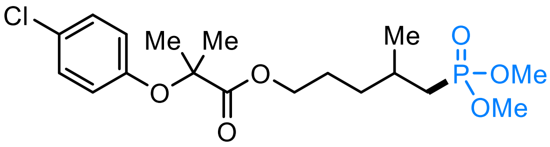

Prepared following **General Procedure C** and the **Work-up Procedure**, using clofibric ester derivative (299 mg, 1.00 mmol, 5.00 equiv.). Purification by flash column chromatography (Biotage Isolera™, SNAP 5 g silica cartridge, 0–5% methanol in EtOAc) gave **39** (24 mg, 30%) as a colorless oil. The r.r. (>*97:3*) was determined by ^{31}P NMR analysis of the crude reaction mixture before the KF work-up (*see spectrum*).

R*f* = 0.3 (EtOAc, $KMnO_4$)

NMR Spectroscopy (*see spectra*):

^{1}**H NMR** (400 MHz, $CDCl_3$) δ_H 7.20 – 7.15 (m, 2H), 6.79 – 6.73 (m, 2H), 4.12 (td, *J* = 6.6, 1.6 Hz, 2H), 3.72 (s, 3H), 3.70 (s, 3H), 1.94 – 1.48 (m, 5H), 1.57 (s, 6H), 1.42 – 1.30 (m, 1H), 1.22 – 1.10 (m, 1H), 0.99 (d, *J* = 6.6 Hz, 3H) ppm;

^{13}**C NMR** (101 MHz, $CDCl_3$) δ_C 174.1, 154.2, 129.2, 127.2, 120.4, 79.6, 65.7, 52.3 (d, *J* = 5.9 Hz), 52.2 (d, *J* = 6.2 Hz), 34.2 (d, *J* = 14.0 Hz), 31.8 (d, *J* = 139.0 Hz), 28.0 (d, *J* = 4.2 Hz), 25.9, δ 25.5, 25.4, 20.8 (d, *J* = 7.7 Hz) ppm;

^{31}P NMR (162 MHz, CDCl$_3$) δ_P 34.7 (s) ppm.

IR (film) $\nu_{max:}$ 3667, 2985, 2902, 1737. 1373, 1234, 1044, 847, 634, 608 cm^{-1}.

HRMS (ESI$^+$) calcd for $C_{18}H_{28}O_7ClP$ [M+H]$^+$: 429.1204, found: 429.1191.

((3a*R*,5*R*,5a*S*,8a*S*,8b*R*)-2,2,7,7-Tetramethyltetrahydro-5*H*-bis([1,3]dioxolo)[4,5-*b*:4',5'-*d*]pyran-5-yl)methyl 5-(dimethoxyphosphoryl)-4-methylpentanoate (40)

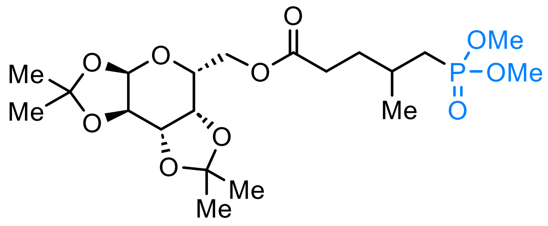

Prepared following **General Procedure A** and the **Work-up Procedure**, using 1,2:3,4-di-*O*-isopropylidene-α-D-galactopyranose ester derivative (358 mg, 1.00 mmol, 5.00 equiv.). Purification by flash column chromatography (Biotage Isolera™, SNAP 5 g silica cartridge, 0–5% methanol in EtOAc) gave **40** (21 mg, 22%) as a colorless oil. The r.r. (>*97:3*) was determined by ^{31}P NMR analysis of the crude reaction mixture before the KF work-up (*see spectrum*) and the d.r. (60:40) was determined by ^{1}H NMR analysis of the purified product.

R*f* = 0.3 (EtOAc, $KMnO_4$)

NMR Spectroscopy (*see spectra*):

^{1}H NMR (400 MHz, CDCl$_3$) δ_H 5.53 (d, *J* = 5.0 Hz, 1H), 4.61 (dd, *J* = 7.9, 2.5 Hz, 1H), 4.34 – 4.26 (m, 2H), 4.23 (dd, *J* = 7.9, 1.9 Hz, 1H), 4.19 (dd, *J* = 7.8, 3.7 Hz, 1H), 4.03 – 3.98 (m 1H), 3.74 (s, 3H), 3.71 (s, 3H), 2.43 – 2.29 (m, 2H), 1.93 (tq, *J* = 12.2, 6.8 Hz, 1H), 1.84 – 1.72 (m, 2H), 1.67 – 1.54 (m, 2H), 1.44 (s, 3H), 1.33 (d, *J* = 3.4 Hz, 6H), 1.05 (d, *J* = 6.7 Hz, 3H) ppm;

^{13}C NMR (101 MHz, CDCl$_3$) δ_C 173.47, 173.44, 109.77, 108.87, 96.41, 76.84, 71.18, 70.82, 70.55, 66.09, 63.54, 60.51, 52.30 (t, *J* = 7.1 Hz), 33.14 (d, *J* = 2.5 Hz) + 32.99 (d, *J* = 2.5 Hz) (diastereomeric peaks), 31.72, 31.68 (d, *J* = 139.0 Hz), 28.02 (t, *J* = 4.3 Hz), 26.10 (d, *J* = 6.8 Hz), 25.07, 24.60, 21.17, 20.54 (d, *J* = 2.7 Hz) + 20.47 (d, *J* = 2.5 Hz) (diastereomeric peaks), 14.32 ppm;

^{31}P NMR (162 MHz, CDCl$_3$) δ_P 34.4 (s) ppm.

IR (film) $\nu_{max:}$ 3667, 2987, 2902, 1735, 1380, 1211, 1066, 842, 731, 512 cm^{-1}.

HRMS (ESI$^+$) calcd for $C_{20}H_{35}O_{10}P$ [M+Na]$^+$: 489.1860, found: 489.1842.

4. MECHANISTIC STUDIES

4.1. Chlorine Radical Trapping Experiment

Dimethyl ((2-(chloromethyl)cyclopentyl)methyl)phosphonate (41)

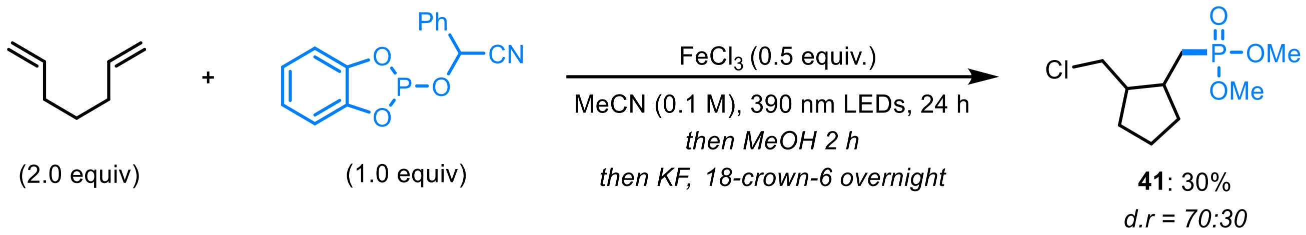

Prepared following a modified **General Procedure A** and the **Work-up Procedure** using $FeCl_3$ (16 mg, 0.10 mmol, 0.50 equiv.), 1,6-heptadiene (54 µL, 39 mg, 0.40 mmol, 2.0 equiv.). Purification by flash column chromatography (98:2 EtOAc/MeOH) gave **41** (7.0 mg, 30% based on $FeCl_3$, 70:30 *d.r.*) as a colorless oil. The d.r. was determined by ^{31}P NMR analysis of the purified product.

R*f* = 0.60 (95/5 EtOAc/MeOH, $KMnO_4$).

NMR Spectroscopy (*see spectra*):

> **^{1}H NMR** (600 MHz, $CDCl_3$) δ_H (*70:30 ratio of diastereomers*) 3.77 – 3.74 (m, 5H), 3.73 (t, *J* = 3.5 Hz, 4H), 3.62 (dd, *J* = 10.9, 4.2 Hz, 0.5H), 3.54 – 3.49 (m, 1H), 3.47 (s, 0.3H), 3.42 (dd, *J* = 10.9, 7.5 Hz, 1H), 2.34 (dt, *J* = 14.1, 6.7 Hz, 2H), 2.08 – 2.01 (m, 1H), 2.01 – 1.76 (m, 6H), 1.76 – 1.47 (m, 7H), 1.46 – 1.35 (m, 0.5H) ppm;

> **^{13}C NMR** (151 MHz, $CDCl_3$) δ_C (*70:30 ratio of diastereomers*) 52.4 (d, *J* = 6.7 Hz, major), 52.3 (d, *J* = 4.4 Hz, minor), 52.3 (d, *J* = 6.2 Hz, major), 52.2 (d, *J* = 5.7 Hz, minor), 48.7 (d, *J* = 15.4 Hz, minor), 48.3 (minor), 45.6 (major), 45.2 (d, *J* = 13.6 Hz, major), 37.7 (d, *J* = 4.8 Hz, minor), 36.3 (d, *J* = 4.4 Hz, major), 34.0 (d, *J* = 5.2 Hz, minor), 31.4 (d, *J* = 5.7 Hz, major), 30.1 (minor), 29.8 (d, *J* = 139.6 Hz, minor), 28.6 (major), 24.4 (d, *J* = 140.9 Hz, major), 23.9 (s, minor), 22.5 (major) ppm;

> **^{31}P NMR** (162 MHz, $CDCl_3$) δ_P 34.26 (major), 33.35 (minor) ppm.

IR (film) ν_{max}: 3451, 2953, 2873, 1455, 1243, 1184, 1055, 1029, 818, 723, 541 cm^{-1}

HRMS (EI) m/z calc'd for $C_9H_{18}O_3PCl$ $[M-Cl]^+$, 205.0988; found, 205.0983.

4.2. Competition Experiments with 2,3-Dimethylbutane

4.2.1. Trapping with benzylidenemalononitrile

In the absence of **P3**, following modified literature conditions for the reaction of 2,3-dimethylbutane with benzylidenemalononitrile, [9] alkylated product was formed in 72% yield with 61:39 regioselectivity for functionalisation of primary:tertiary positions.

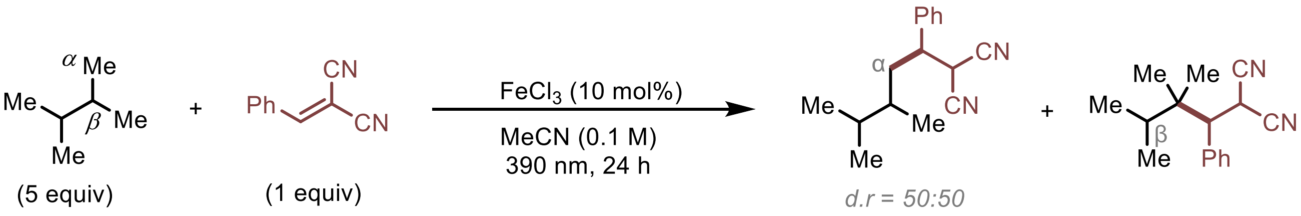

42a **42b**

72% yield *α:β* = *61:39*

A flame dried 8 mL Biotage® microwave reaction vial equipped with a magnetic stir bar was transferred into an anhydrous, argon-filled glovebox. The vial was charged with $FeCl_3$ (3.2 mg, 0.020 mmol, 0.10 equiv.) and the benzylidenemalononitrile (BMN, 31 mg, 0.20 mmol, 1.0 equiv.). Anhydrous acetonitrile was then added (2.0 mL, *c* = 0.10 M) followed by 2,3- dimethylbutane (86.2 mg, 130 μL, 1.00 mmol, 5.00 equiv.) The vial was crimped with a cap with septum, removed from the glovebox, and placed 5 cm away from one Kessil PR160-390 nm LEDs (see Figure S1). The reaction mixture was stirred at 1000 rpm and irradiated with fan cooling for 24 h. The reaction mixture was concentrated in vacuo and purified by flash column chromatography, eluting with EtOAc/hexane (20/80) to give **42** (35 mg, 72%,) as a white solid. The r.r. (*α:β = 61:39*) and d.r (50:50 for the *α* regioisomer) were determined by ^{1}H NMR analysis of the purified product.

R_f = 0.40 (20/80 EtOAc/hexane, $KMnO_4$).

NMR Spectroscopy of 42a (*see spectra*):

> **^{1}H NMR** (400 MHz, $CDCl_3$) δ_H (*50:50 ratio of diastereomers*): 7.47 – 7.29 (m, 5H), 3.88 (d, *J* = 5.9 Hz, 0.5H) 3.85 (d, *J* = 6.3 Hz, 0.5H), 3.33 – 3.25 (m, 1H), 2.17 – 1.99 (m, 1H), 1.81 (ddd, *J* = 14.0, 9.6, 6.3 Hz, 0.5H), 1.73 – 1.60 (m, 1H), 1.53 – 1.47 (m, 0.5H), 1.32 (dq, *J* = 10.6, 3.5 Hz, 0.5H), 1.12 (s, 0.5H), 0.84 (d, *J* = 3.2 Hz, 1.5H), 0.83 – 0.80 (m, 6H) 0.78 (d, *J* = 6.9 Hz, 1.5H) ppm;
>
> **^{13}C NMR** (101 MHz, $CDCl_3$) δ_C (*50:50 ratio of diastereomers*): 137.3, 136.4, 129.3, 129.3, 128.9, 128.9, 128.0, 127.9, 112.0, 112.0, 112.0, 111.9, 44.7, 44.6, 36.9, 35.8, 35.6, 35.3, 32.9, 31.0, 30.1, 20.5, 19.4, 18.5, 16.4, 15.1, 15.0 ppm.

NMR Spectroscopy of 42b (*see spectra*):

> **^{1}H NMR** (400 MHz, $CDCl_3$) δ_H 7.47 – 7.34 (m, 5H), 4.21 (d, *J* = 5.1 Hz, 1H), 3.29 (d, *J* = 5.3 Hz, 1H), 1.60 – 1.54 (m, 1H), 1.12 (s, 3H), 0.93 (s, 3H), 0.90 (d, *J* = 6.3 Hz, 3H), 0.87 (d, *J* = 4.5 Hz, 3H) ppm;
>
> **^{13}C NMR** (101 MHz, $CDCl_3$) δ_C 136.1, 129.8, 128.7, 128.6, 113.4, 113.2, 53.4, 39.9, 34.0, 25.0, 21.5, 20.9, 17.5, 17.2 ppm.

All recorded spectroscopic data matched those previously reported in the literature.[10]

Evidence for the formation of hindered tertiary alkyl radicals was provided upon reacting 2,3-dimethylbutane with **P3** in the presence of benzylidenemalononitrile. **6** was formed in 36% yield with >97:3 regioselectivity for phosphonylation of the primary positions. Alkylated product **42** was formed in 56% yield with 10:90 regioselectivity for functionalisation of primary:tertiary positions.

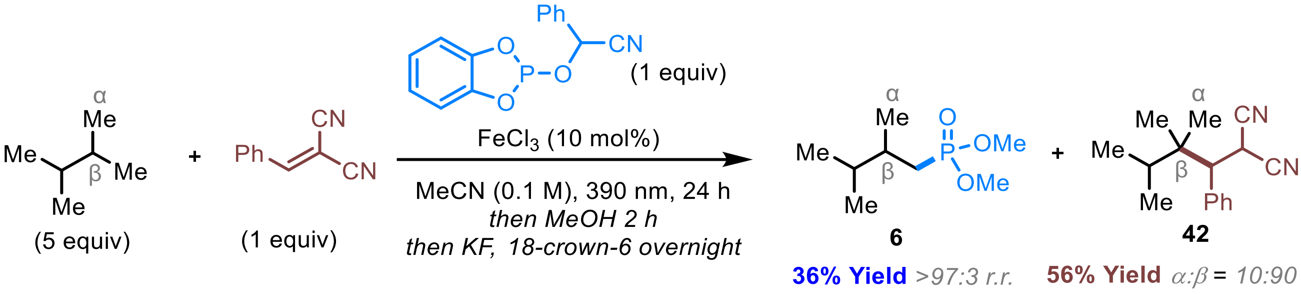

Prepared following **General Procedure C** and the **Work-up Procedure,** using 2,3- dimethylbutane (86.2 mg, 130 µL, 1.00 mmol, 5.00 equiv.). Purification by flash column chromatography (98:2 EtOAc/MeOH) gave **6** (14 mg, 36%) as a colorless oil and **42** (27 mg, 56%) as a colorless oil. The r.r. (*>97:3*) of **6** was determined by ^{31}P NMR analysis of the crude reaction mixture (Figure S2). The r.r. (*α:β = 10:90*) and d.r. (50:50 for the *α* regioisomer) of **42** was determined by ^{1}H NMR analysis of the purified product (Figure S3).

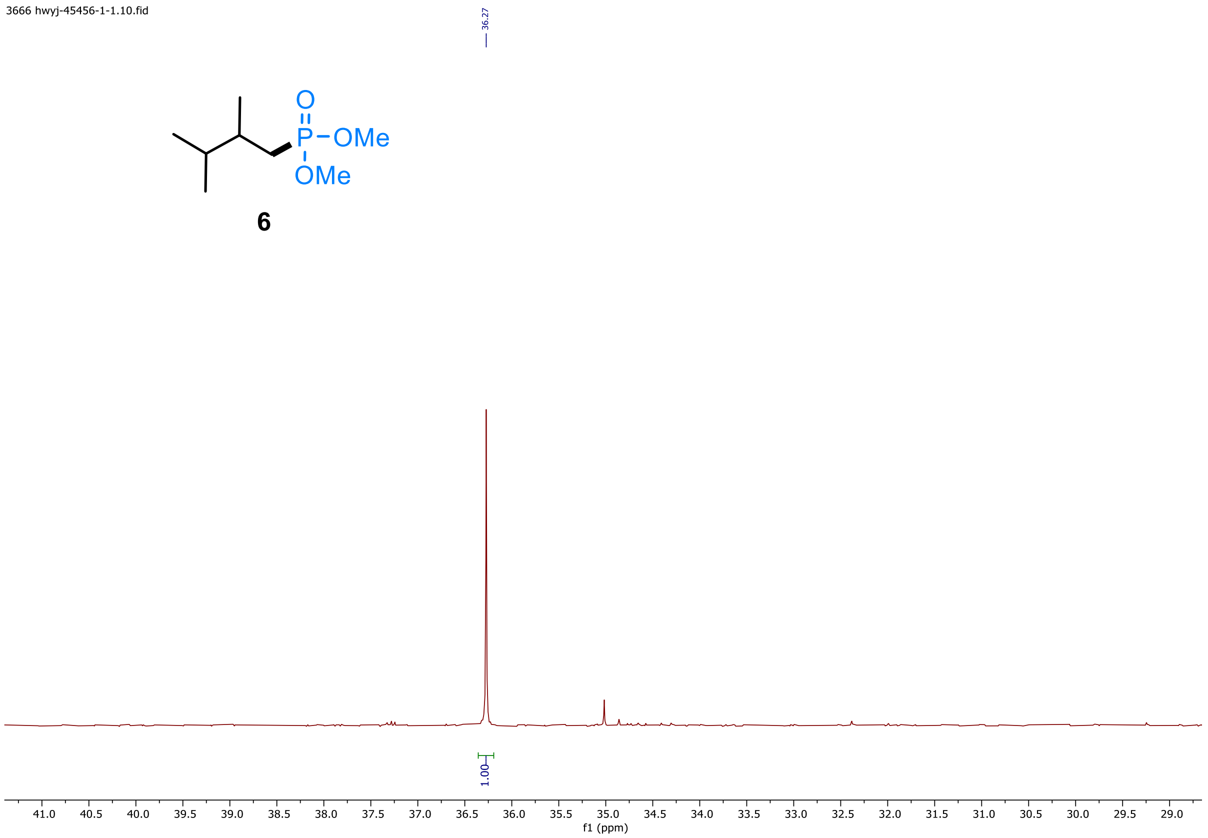

Figure S2. ^{31}P NMR analysis of the competition experiment with BMN

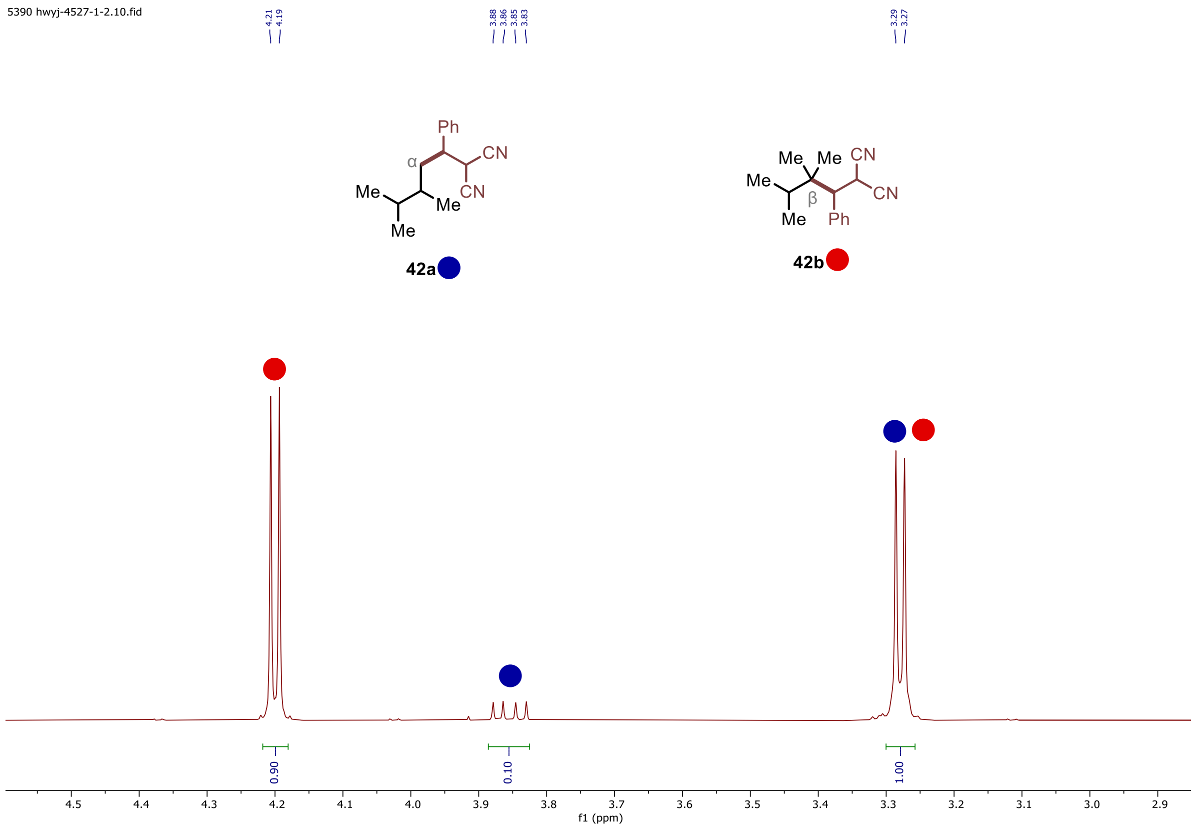

Figure S3. ^{1}H NMR analysis of the competition experiment with BMN

4.2.2. Trapping with ethyl acrylate

In the absence of **P3**, following modified literature conditions for the reaction of 2,3-dimethylbutane with ethyl acrylate,[11] alkylated product was formed in 30% yield with 54:46 regioselectivity for functionalisation of primary:tertiary positions.

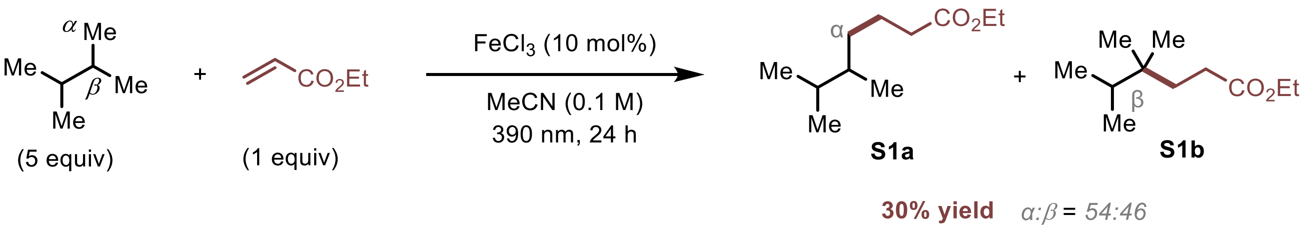

A flame dried 8 mL Biotage® microwave reaction vial equipped with a magnetic stir bar was transferred into an anhydrous, argon-filled glovebox. The vial was charged with $FeCl_3$ (3.2 mg, 0.020 mmol, 0.10 equiv.). Anhydrous acetonitrile (2.0 mL, c = 0.10 M) was then added followed by 2,3- dimethylbutane (86.2 mg, 130 µL, 1.00 mmol, 5.00 equiv.) and ethyl acrylate (20 mg, 0.20 mmol, 1.0 equiv.). The vial was crimped with a cap with septum, removed from the glovebox, and placed 5 cm away from one Kessil PR160-390 nm LEDs (see Figure S1). The reaction mixture was stirred at 1000 rpm and irradiated with fan cooling for 24 h. After irradiation, the crude reaction mixture was filtered through a short plug of silica and the r.r. (*α: β = 54:46*) of alkylated product **S1** was determined by GC-FID analysis (Figure S4). The reaction mixture was concentrated in vacuo and

purified by flash column chromatography, eluting with Et_2O/pentane (5/95) to give **S1** (11 mg, 30%,) as a colorless oil.

R*f* = 0.50 (25/75 Et_2O/pentane, $KMnO_4$).

NMR Spectroscopy of S1a (*see spectra*):

^{1}H NMR (400 MHz, $CDCl_3$) δ_H 4.12 (q, *J* = 7.0 Hz, 2H), 2.31 – 2.26 (m, 2H), 1.61 – 1.52 (m, 3H), 1.25 (t, *J* = 7.0 Hz, 4H), 0.89 – 0.76 (m, 12H) ppm;

^{13}C NMR (101 MHz, $CDCl_3$) δ_C 174.0, 60.3, 38.4, 34.8, 33.7, 31.9, 23.1, 20.3, 18.0, 15.3, 14.3, ppm.

NMR Spectroscopy of S1b (*see spectra*):

^{1}H NMR (400 MHz, $CDCl_3$) δ_H 4.12 (q, *J* = 7.0 Hz, 2H), 2.26 – 2.20 (m, 2H), 1.61 – 1.52 (m, 2H), 1.49 – 1.40 (m, 1H), 1.25 (t, *J* = 7.0 Hz, 3H), 0.87 – 0.81 (m, 6H), 0.81 – 0.77 (m, 6H) ppm;

^{13}C NMR (101 MHz, $CDCl_3$) δ_C 174.8, 60.3, 35.4, 35.0, 34.8, 29.6, 23.8, 17.4, 14.3 ppm.

All recorded spectroscopic data matched those previously reported in the literature.[12]

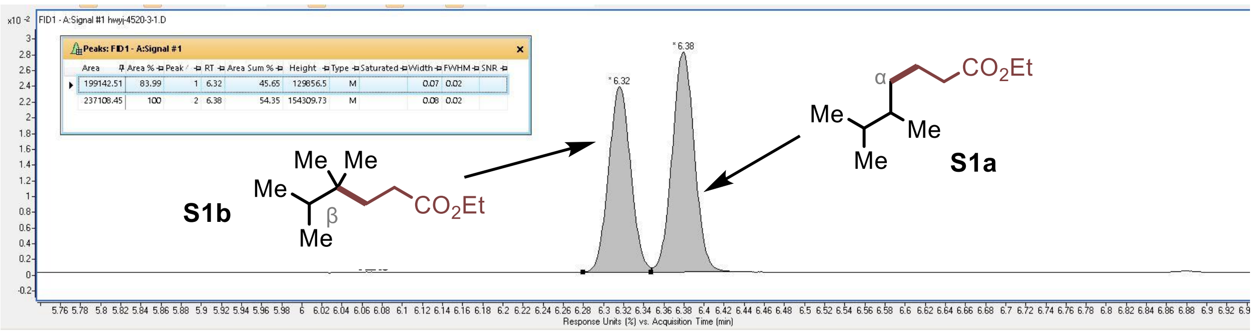

Figure S4. GC-FID analysis of the reaction with ethyl acrylate (in the absence of P3)

Evidence for the formation of hindered tertiary alkyl radicals was provided upon reacting 2,3-dimethylbutane with **P3** in the presence of ethyl acrylate. **6** was formed in 20% yield with >97:3 regioselectivity for phosphonylation of the primary positions. Alkylated product **S1** was formed in 46% yield with 96:4 regioselectivity for alkylation of tertiary positions.

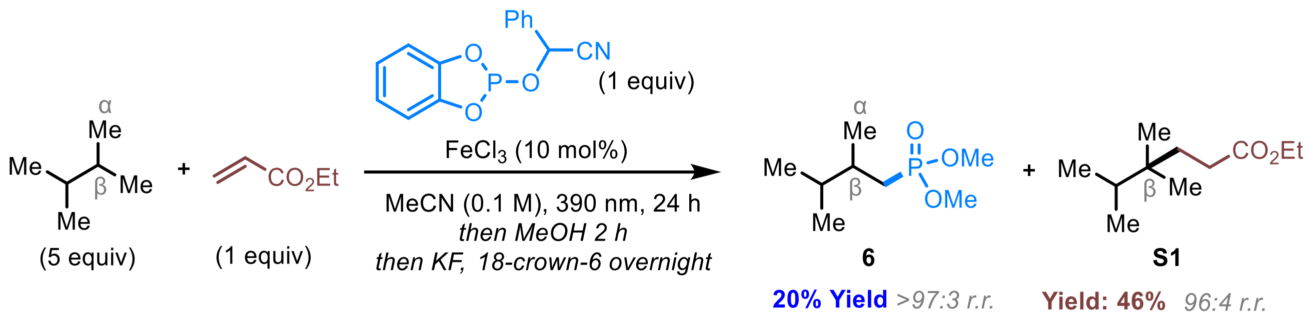

A flame dried 8 mL Biotage® microwave reaction vial equipped with a magnetic stir bar was transferred into an anhydrous, argon-filled glovebox. The vial was charged with $FeCl_3$ (3.2 mg, 0.020 mmol, 0.10 equiv.), phosphite **P3** (54 mg, 0.20 mmol, 1.0 equiv.) and ethyl acrylate (20 mg, 0.20 mmol, 1.0 equiv.). Anhydrous acetonitrile (2.0 mL, c = 0.10 M) was added followed by the 2,3- dimethylbutane (86.2 mg, 130 μL, 1.00 mmol, 5.00 equiv.). The vial was crimped with a cap with septum, removed from the glovebox, and placed 4 cm away from one Kessil PR160-390 nm LEDs (see Figure S1). The reaction mixture was stirred at 1000 rpm and irradiated with fan cooling for 24 h. After irradiation, anhydrous methanol (1.0 mL) was added under N_2 and stirring continued for 2 h. The reaction mixture was filtered through silica gel (2.3 cm diameter sintered funnel packed with 10 cm silica gel), eluting with ethyl acetate (50 mL), then concentrated in vacuo. The crude catechol phosphonate ester was then subjected to the **Work-up procedure**. After that, the crude reaction mixture was filtered through a short plug of silica and the r.r. (*96:4*) of alkylated product **S1** was determined by GC-FID analysis (Figure S5). Then, the reaction mixture was concentrated in vacuo and purified by flash column chromatography, eluting with EtOAc/MeOH (98:2) gave **6** (8 mg, 20%) as a colorless oil and **S1** (17 mg, 46%) as a colorless oil. The r.r. of **6** was determined by ^{31}P NMR analysis of the crude reaction mixture (Figure S6).

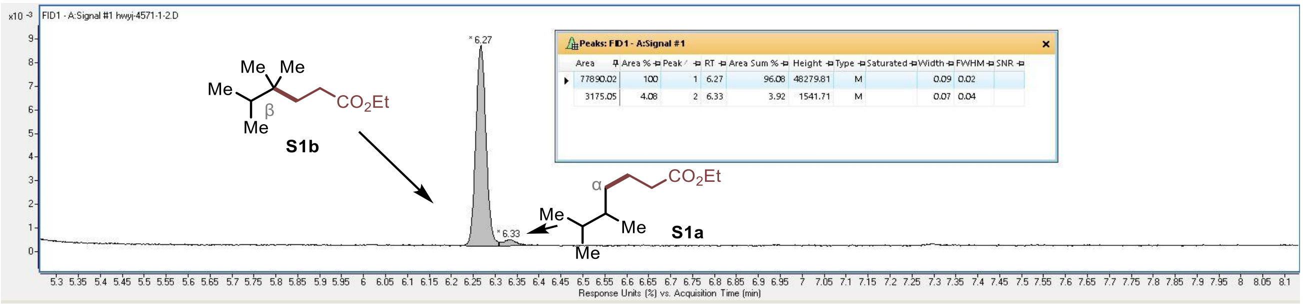

Figure S5. GC-FID analysis of competition reaction with ethyl acrylate

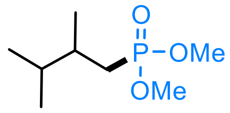

6

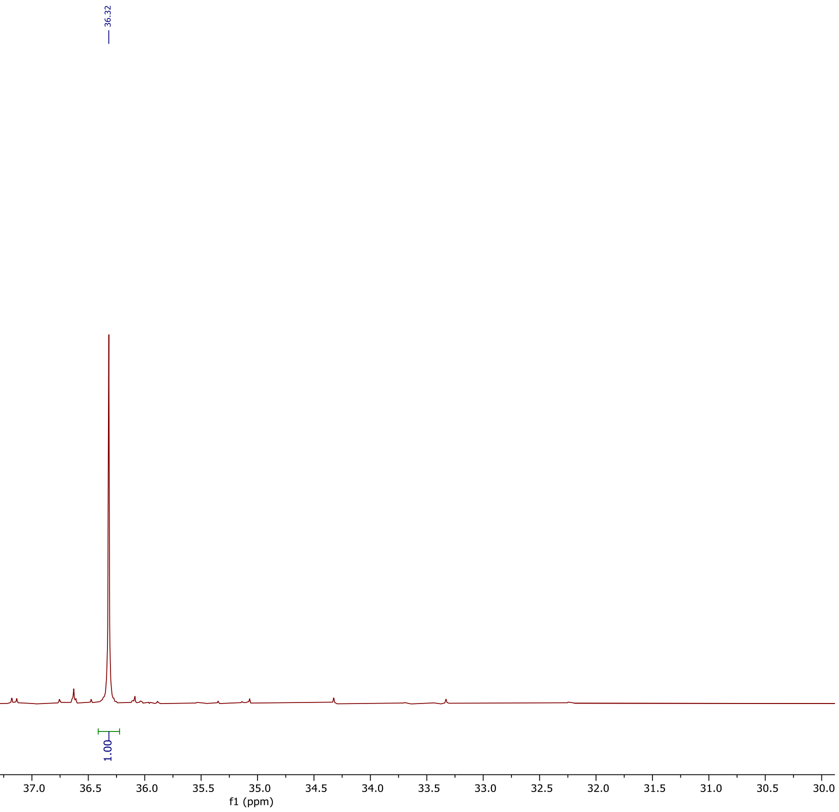

Figure S6. ^{31}P NMR analysis of the competition experiment with ethyl acrylate

4.3. Kinetic Isotope Effect Experiments

4.3.1. Intermolecular KIE experiment with cyclohexane

Using General Procedure A:

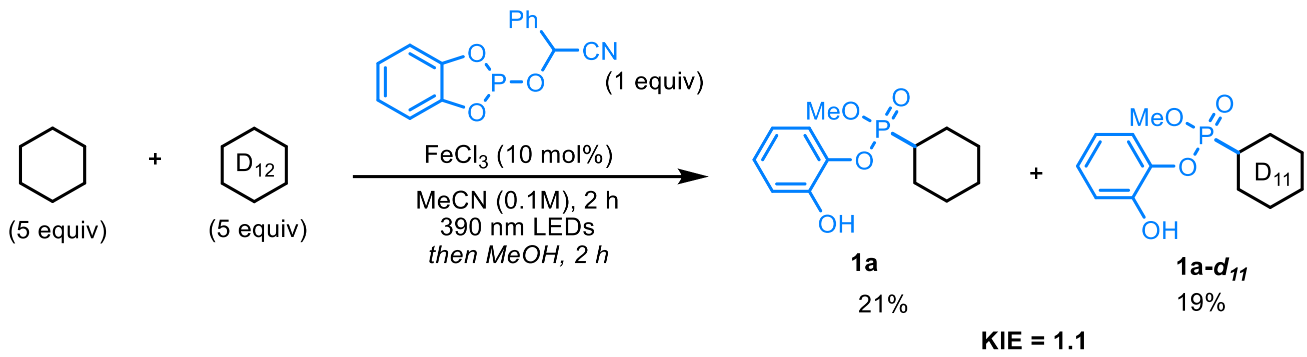

Prepared following **General Procedure A**, from a 1:1 mixture of cyclohexane and cyclohexane-d12 (total alkane = 5 + 5 equiv.). Irradiation time = 2h. Triphenylphosphine oxide as an internal standard was subsequently added. After vigorously shaking for 3 min, the reaction mixture was filtered through silica gel (2.3 cm diameter sintered funnel packed with 10 cm silica gel), eluting with ethyl acetate (50 mL), then concentrated *in vacuo* for ^{31}P NMR analysis. The yields of **1a** and **1a-*d₁₁*** were determined by ^{31}P NMR analysis (Figure S7).

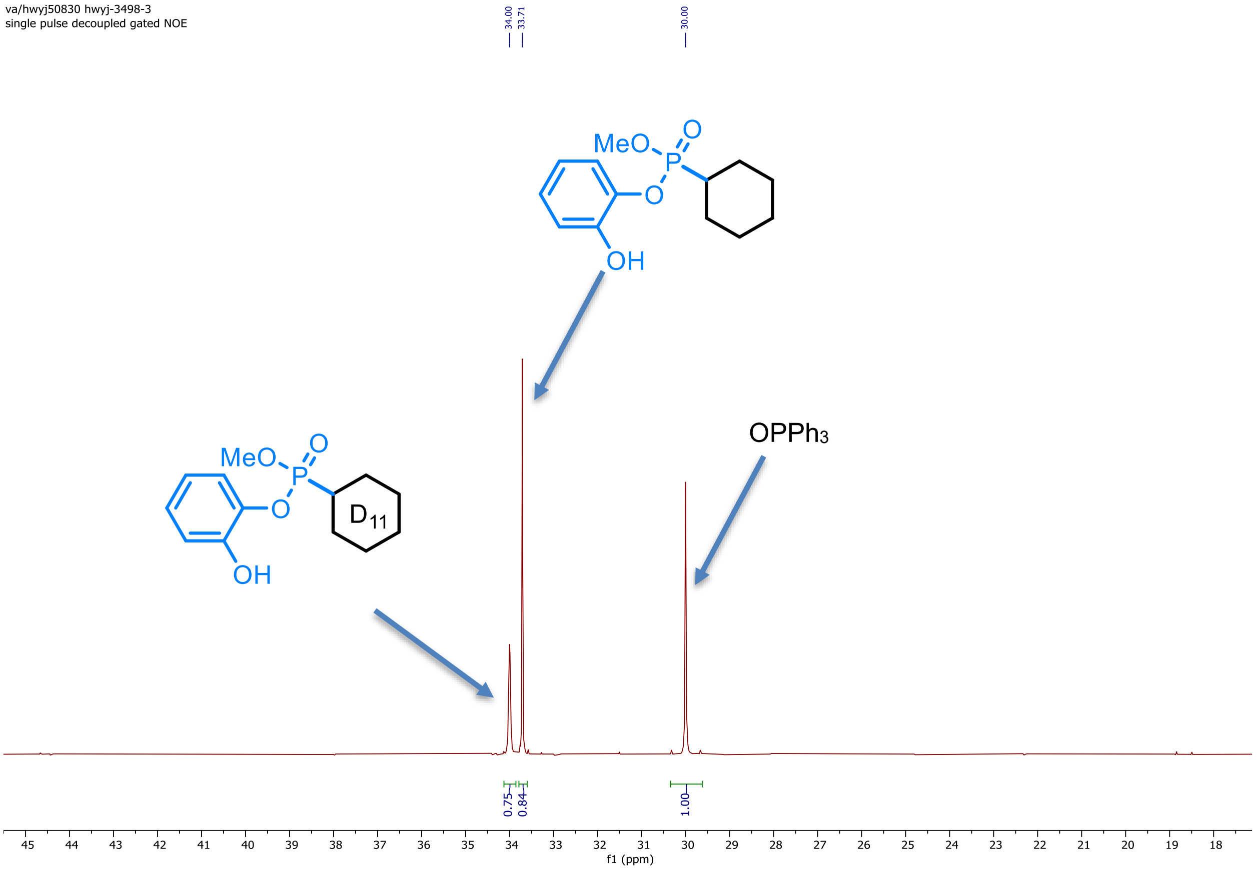

Figure S7. ^{31}P NMR analysis of the intermolecular competition experiment for KIE determination

4.3.2. Parallel KIE experiment with cyclohexane

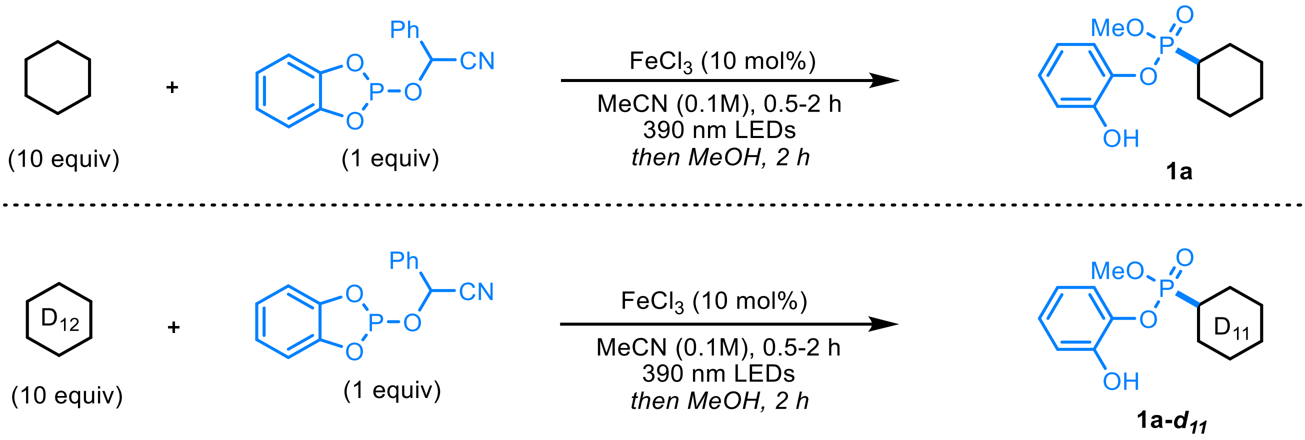

A flame dried 8 mL Biotage® microwave reaction vial equipped with a magnetic stir bar was transferred into an anhydrous, argon-filled glovebox. The vial was charged with $FeCl_3$ (3.2 mg, 0.020 mmol, 0.10 equiv.) and phosphite **P3** (54 mg, 0.20 mmol, 1.0 equiv.). Anhydrous acetonitrile (2.0 mL, *c* = 0.10 M) was added followed by cyclohexane or cyclohexane-d_{12} (1.00 mmol, 5.00 equiv.). The vial was crimped with a cap with septum, removed from the glovebox, and placed 5 cm away from one Kessil PR160-390 nm LEDs (see Figure S1). The reaction mixture was stirred at 1000 rpm and irradiated with fan cooling for specific time. After irradiation, anhydrous methanol (1.0 mL) was added under N_2 and stirring was continued for 2 h. Triphenylphosphine oxide as an internal standard was subsequently added. After vigorously shaking for 3 min, the reaction mixture was filtered through silica gel (2.3 cm diameter sintered funnel packed with 10 cm silica gel), eluting with ethyl acetate (50 mL), then concentrated *in vacuo* and the yield was determined by ^{31}P NMR analysis (Table S9).

**Time (min)**	30	45	60	90	120
**NMR yield of *1a* (%)**	8	18	23	26	41
**NMR yield of *1a-d₁₁* (%)**	9	14	16	25	29

Table S9. NMR yields of 1a/1a-d_{11} at different time points

The KIE value from the parallel experiments was determined from ratio of the gradients of the trendlines shown in Figure S8:

$$\textbf{KIE} = \frac{0.334}{0.262} = 1.27$$

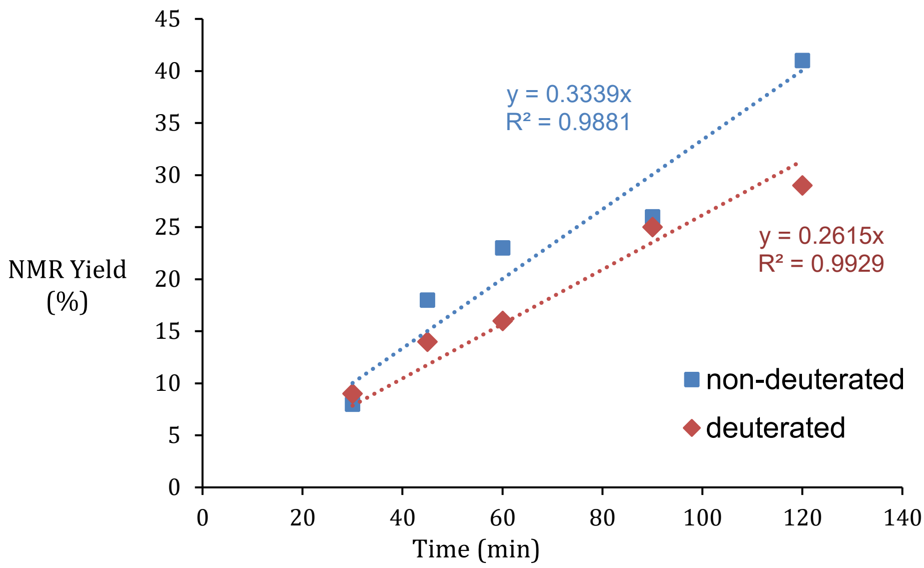

Figure S8. Trendlines of the parallel KIE experiments with cyclohexane

4.4. Deuterium-Labeling Experiments

4.4.1. Deuterium incorporation into phenylacetontrile by-product 43

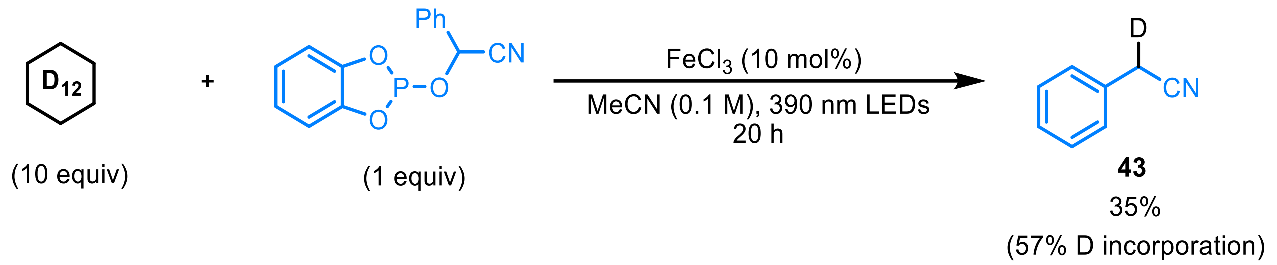

Prepared following **General Procedure A**, using cyclohexane-d12 (96.2 mg, 108 µL, 1.00 mmol, 5.00 equiv.). After irradiation, 1,3,5-Trimethoxybenzene as an internal standard was subsequently added. After vigorously shaking for 3 min, the crude reaction mixture was filtered through a short plug of silica for GC-FID and HR-GCMS analysis. The yield of **43** was determined by GC-FID analysis. The deuterium incorporation (57%) of **43** was determined by HR-GCMS analysis (Figure S9).

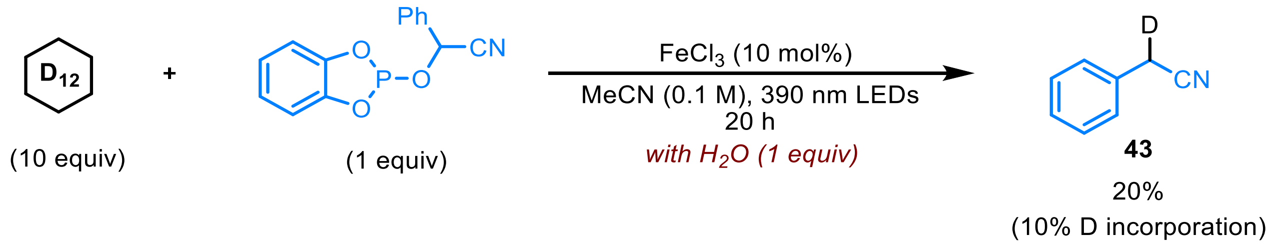

Prepared following **General Procedure A**, using cyclohexane-d12 (96.2 mg, 108 µL, 1.00 mmol, 5.00 equiv.), water (3.6 mg, 3.6 µL, 0.20 mmol, 1.0 equiv.). After irradiation, 1,3,5-Trimethoxybenzene as an internal standard was subsequently added. After vigorously shaking for 3 min, the crude reaction mixture was filtered through a short plug of silica for GC-FID and HR-GCMS analysis. The yield of **43** was determined by GC-FID analysis. The deuterium incorporation (10%) of **43** was determined by HR-GCMS analysis (Figure S10). The drop in deuteration of **43** in the presence of water indicates that part of the proton comes from water in the reaction system.

QExactive Accurate Mass EI-GC-MS
Mass spectrum for 5.34-5.39 mins

Sample: hw-4512-4-1

Filename: va-wh-38233

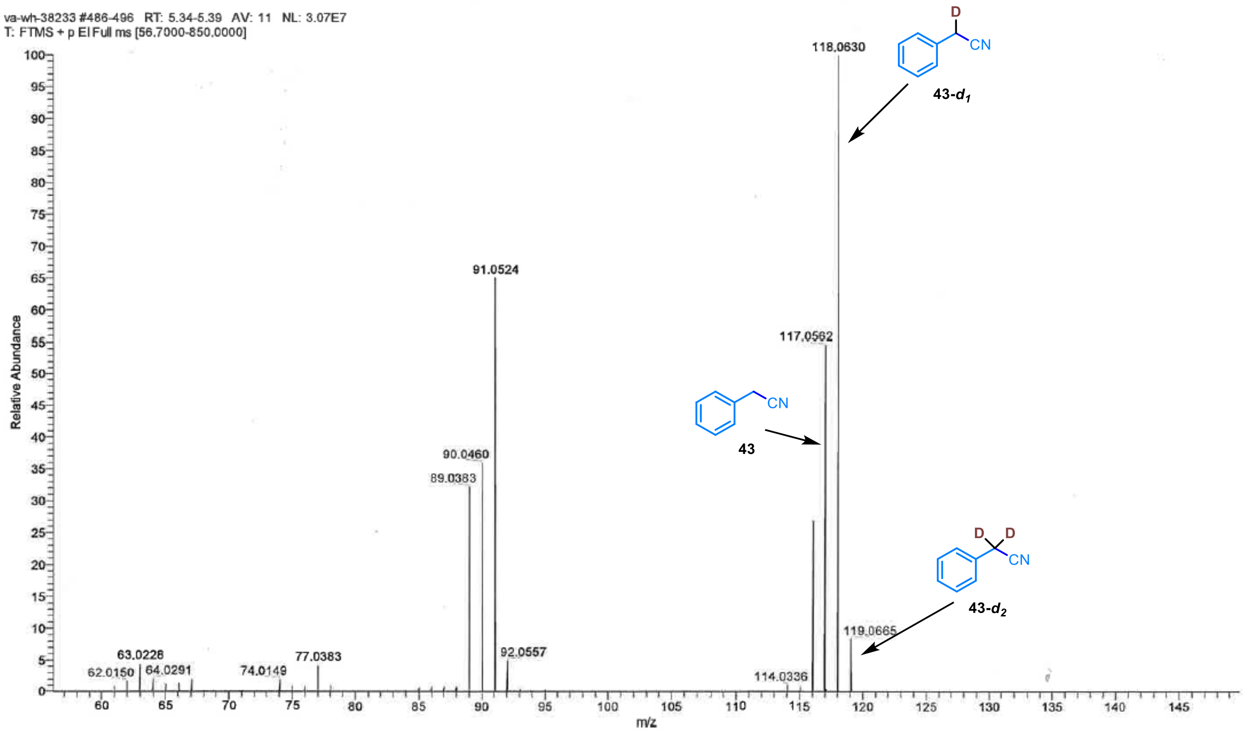

QExactive Accurate Mass EI-GC-MS
Quant for 8.68-8.70 mins

Sample: hw-4512-4-1

Filename: va-wh-38233

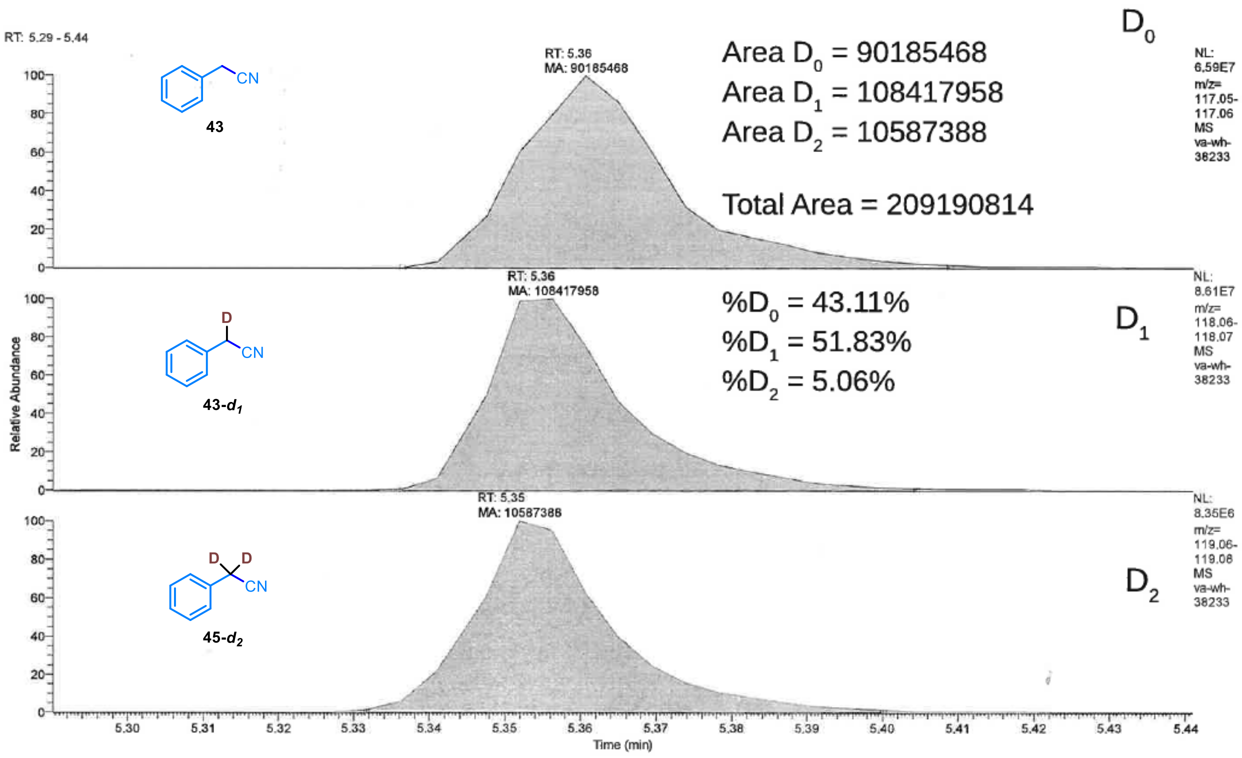

Figure S9. HR-GCMS quantification for the deuterium incorporation experiments

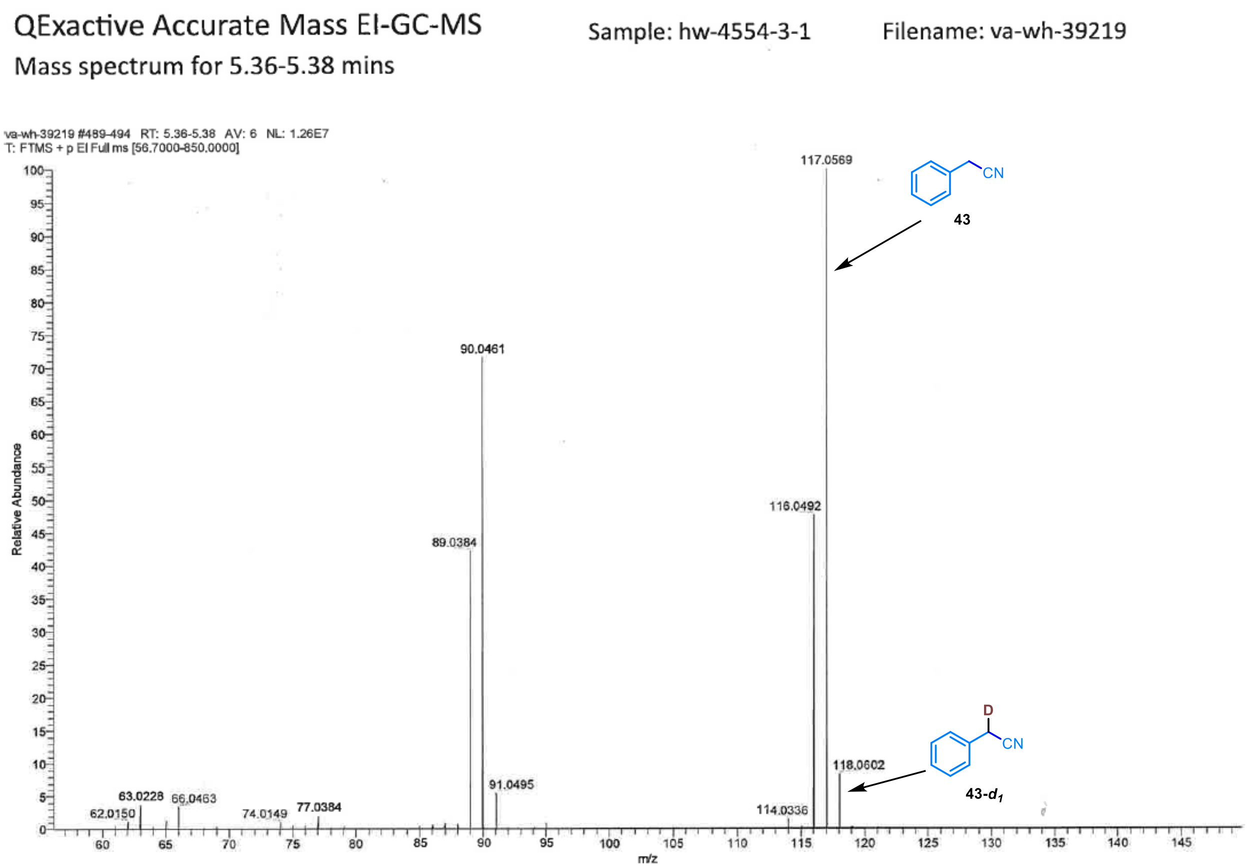

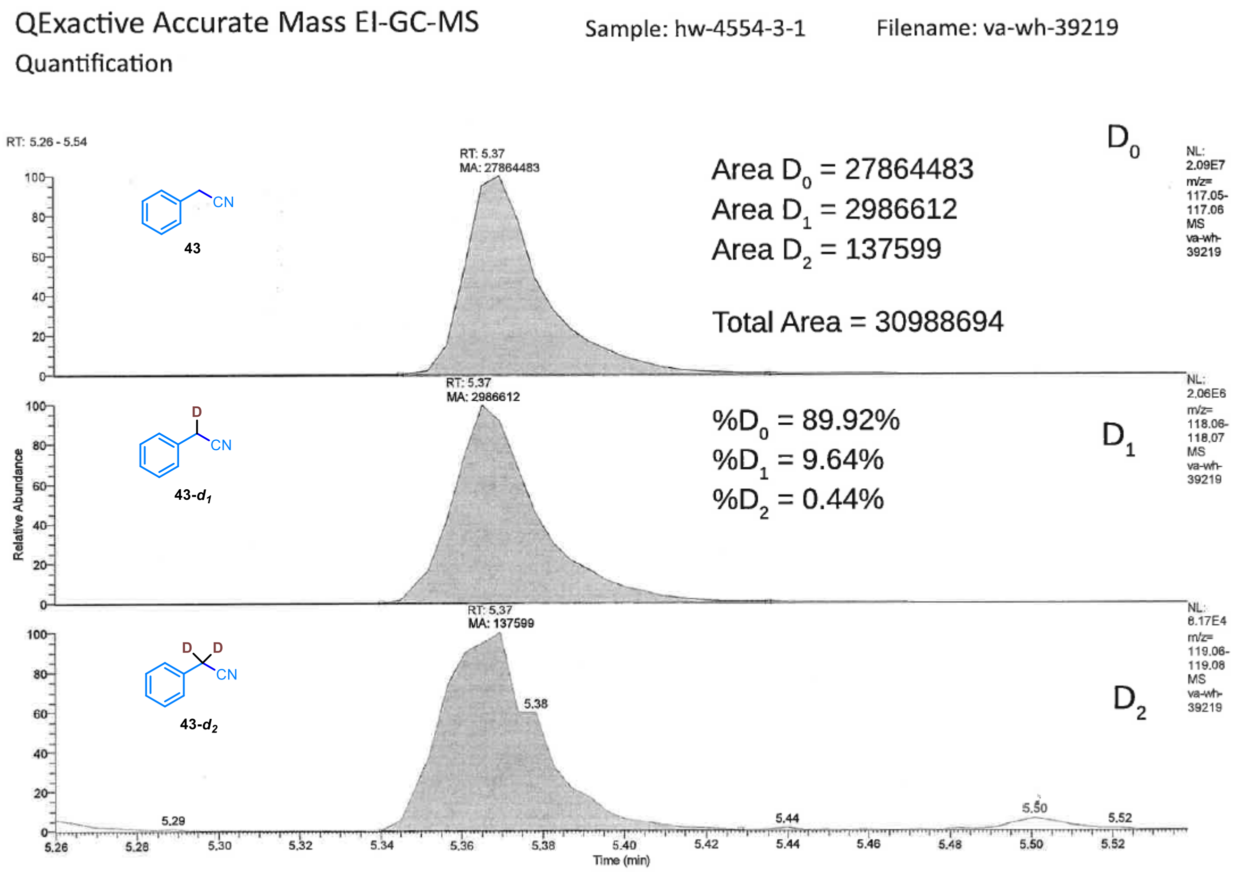

Figure S10. HR-GCMS quantification for the deuterium incorporation experiment with H_2O

4.4.2. Deuterium/hydrogen exchange in the unsuccessful phosphonylation of toluene-d_8

To probe the cause of the unsuccessful phosphonylations of substrates containing sterically unhindered benzylic or allylic C–H bonds (see section 2.2.9), we performed the phosphonylation of toluene-d_8 with phosphite **P3** and analyzed the level of D/H exchange by HRMS. Whilst no phosphonylation product was formed, 59% H-incorporation was observed in the recovered toluene. This indicates that HAT occurs to generate benzylic radicals, but these more stabilized radicals fail to react with **P3**. Instead, a reverse-HAT process occurs, likely similar to that described in Scheme 5 of the manuscript (except with **P3** instead of **43**). We believe this is caused by the lower reactivity of the stabilised benzylic radical, which does not undergo productive reaction with phosphite **P3**.

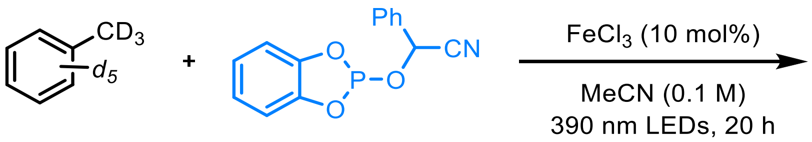

toluene-d8 (5 equiv) **P3** (0.2 mmol) **toluene-d8** **toluene-d7**
ratio: 41 : 59

The reactions were performed following **General Procedure A**, using toluene-d_8 (100.2 mg, 106 µL, 1.00 mmol, 5.00 equiv.) and MeCN (2.0 mL). After irradiation, the crude reaction mixture was filtered through a short plug of silica and submitted for HR-GCMS analysis. The H-incorporation in the recovered toluene was found to be 59% (Figure S11).

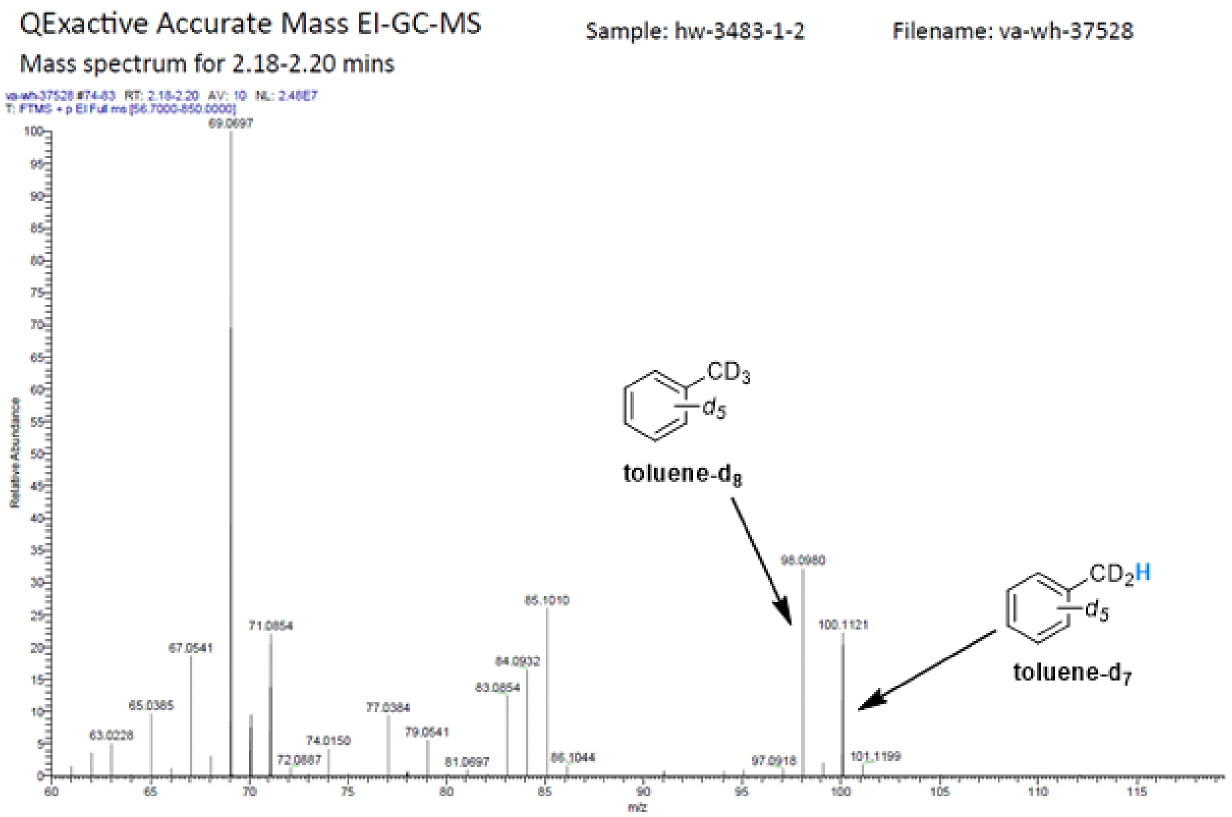

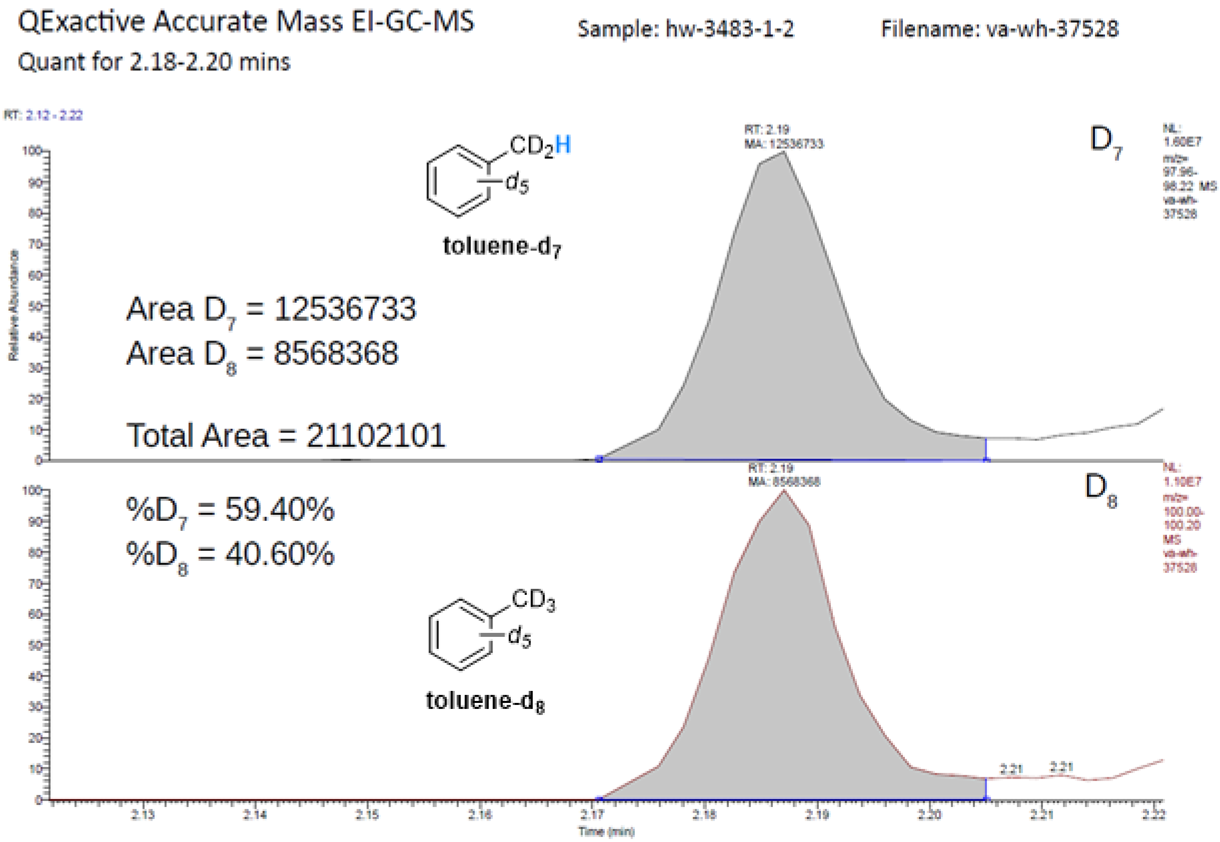

Figure S11. HR-GCMS quantification for the Deuterium/hydrogen exchange with toluene-d_8

4.5. UV-Vis Spectroscopy

The UV-Vis spectra in Figure S12 were measured using the following samples in anhydrous MeCN:

(A) $FeCl_3$ (0.16 mM)
(B) Phosphite **P3** (1.6 mM)
(C) Mixture of $FeCl_3$ (0.16 mM) and **P3** (1.6 mM) [measurement taken 30 min after mixing]

Only minor changes in the absorbance of $FeCl_3$ were observed upon addition of phosphite **P3** (10 equiv), which suggests there is minimal interaction between **P3** and iron. Therefore, iron–phosphite complexation is unlikely to be involved in either photoactivation or C–P bond formation.

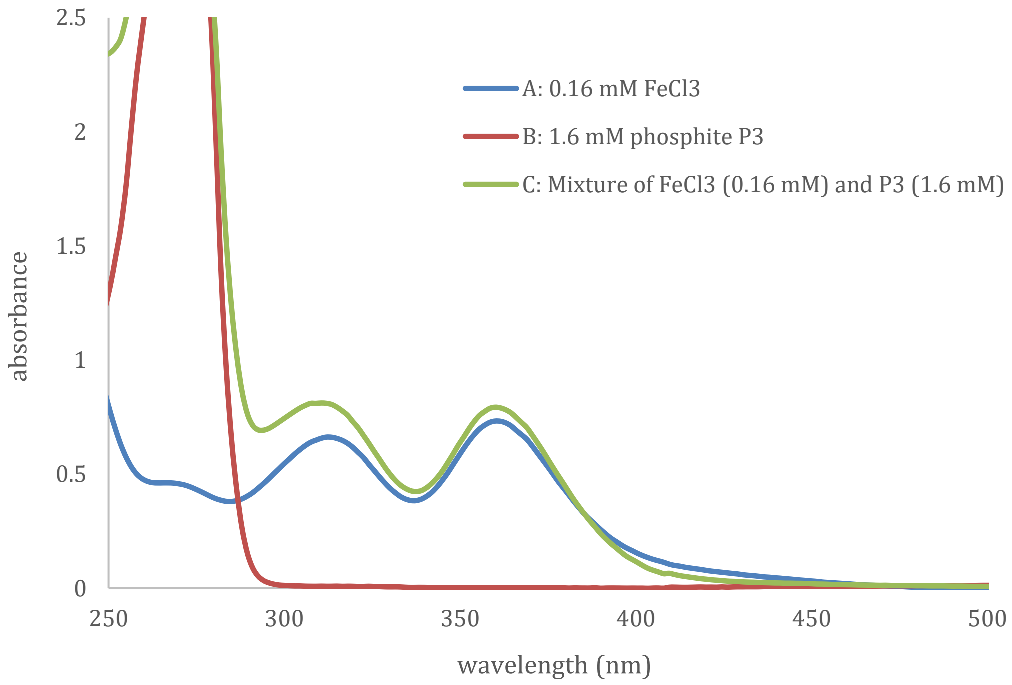

Figure S12. UV-Vis spectra of $FeCl_3$ and phosphite P3 in MeCN

5. SPECTROSCOPIC DATA

^{1}H NMR (400 MHz, $CDCl_3$) of **37-S** (*see procedure*)

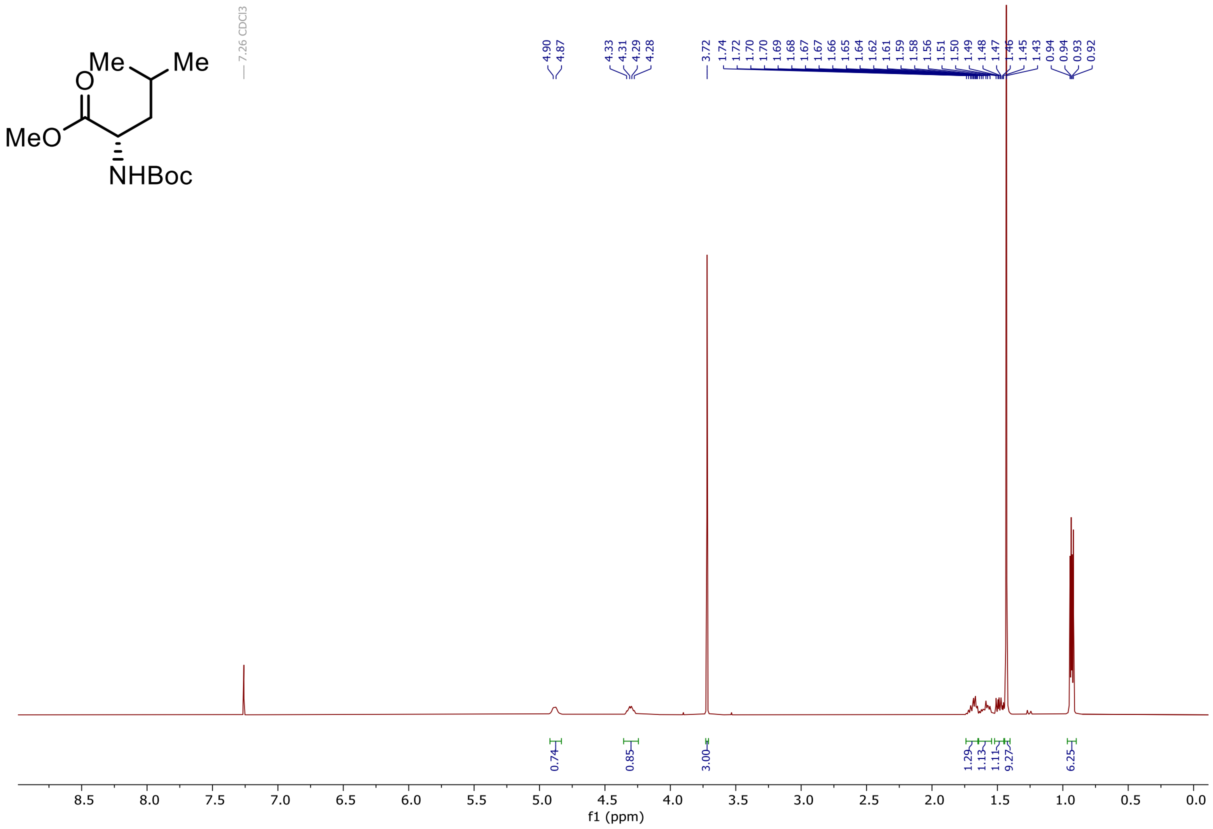

^{13}C NMR (101 MHz, $CDCl_3$) of **37-S**

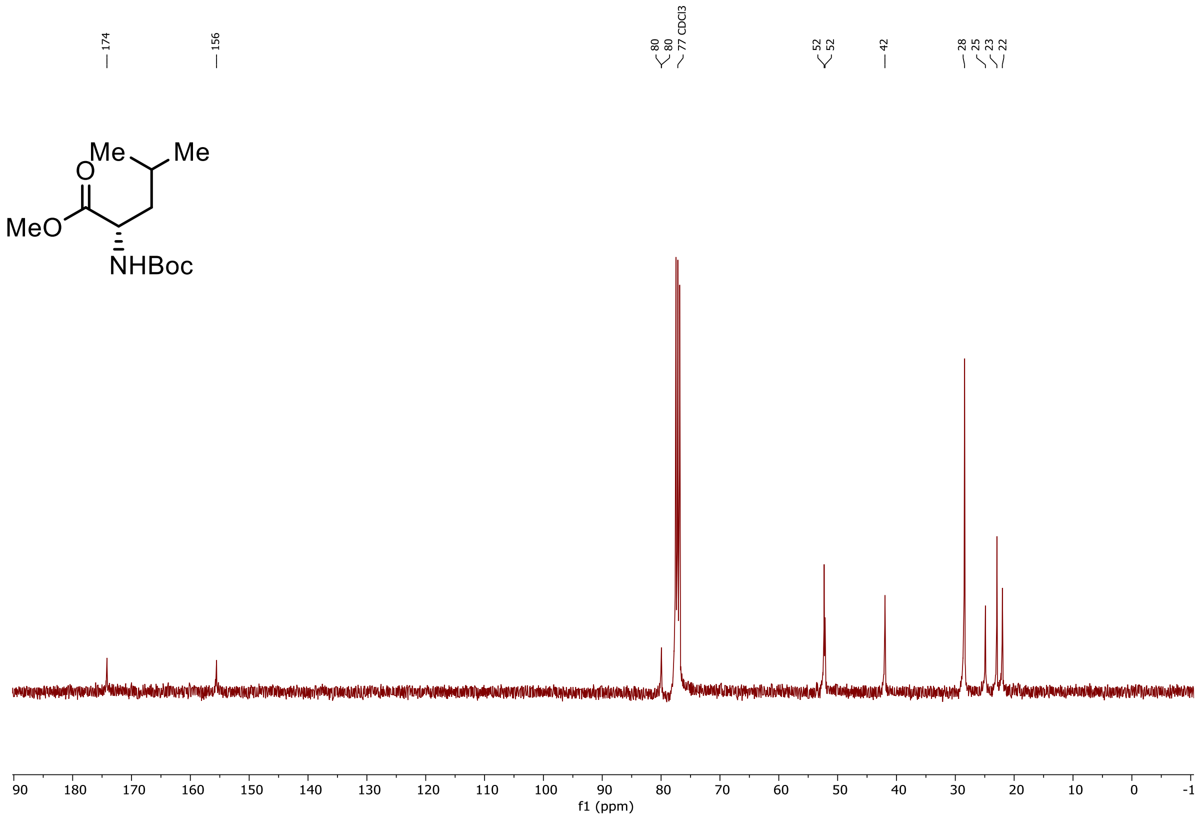

^{1}H NMR (400 MHz, $CDCl_3$) of **38-S** (*see procedure*)

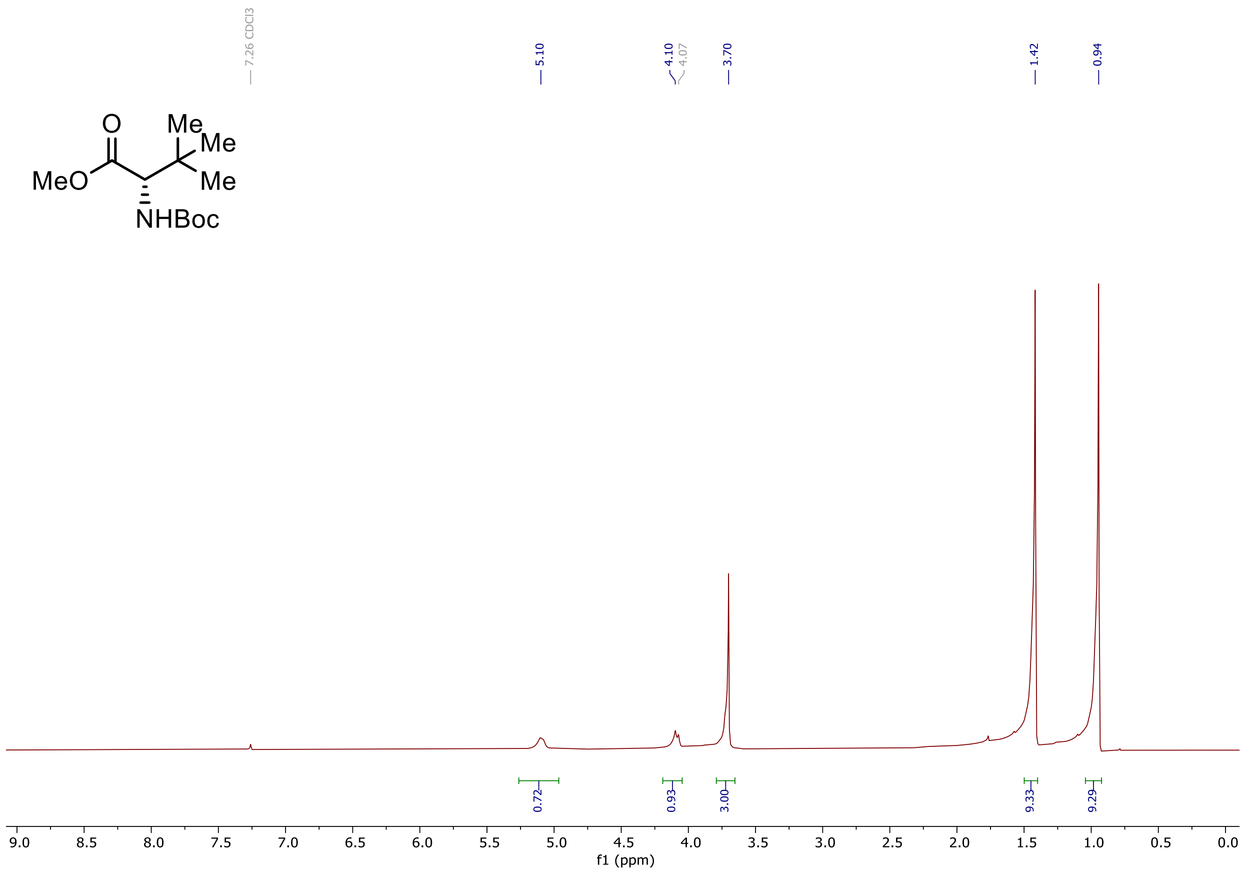

^{13}C NMR (101 MHz, $CDCl_3$) of **38-S**

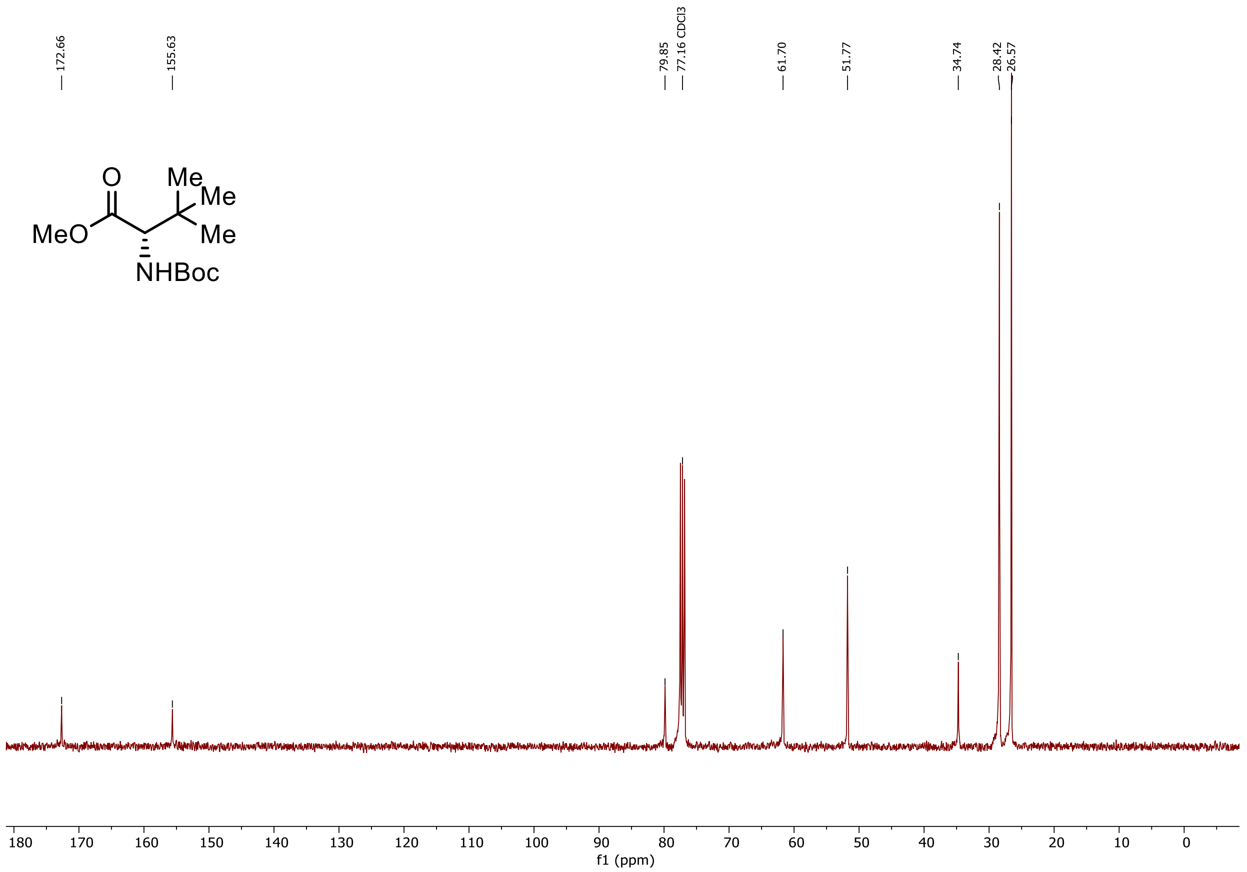

^{1}H NMR (400 MHz, $CDCl_3$) of **P4-S** (*see procedure*)

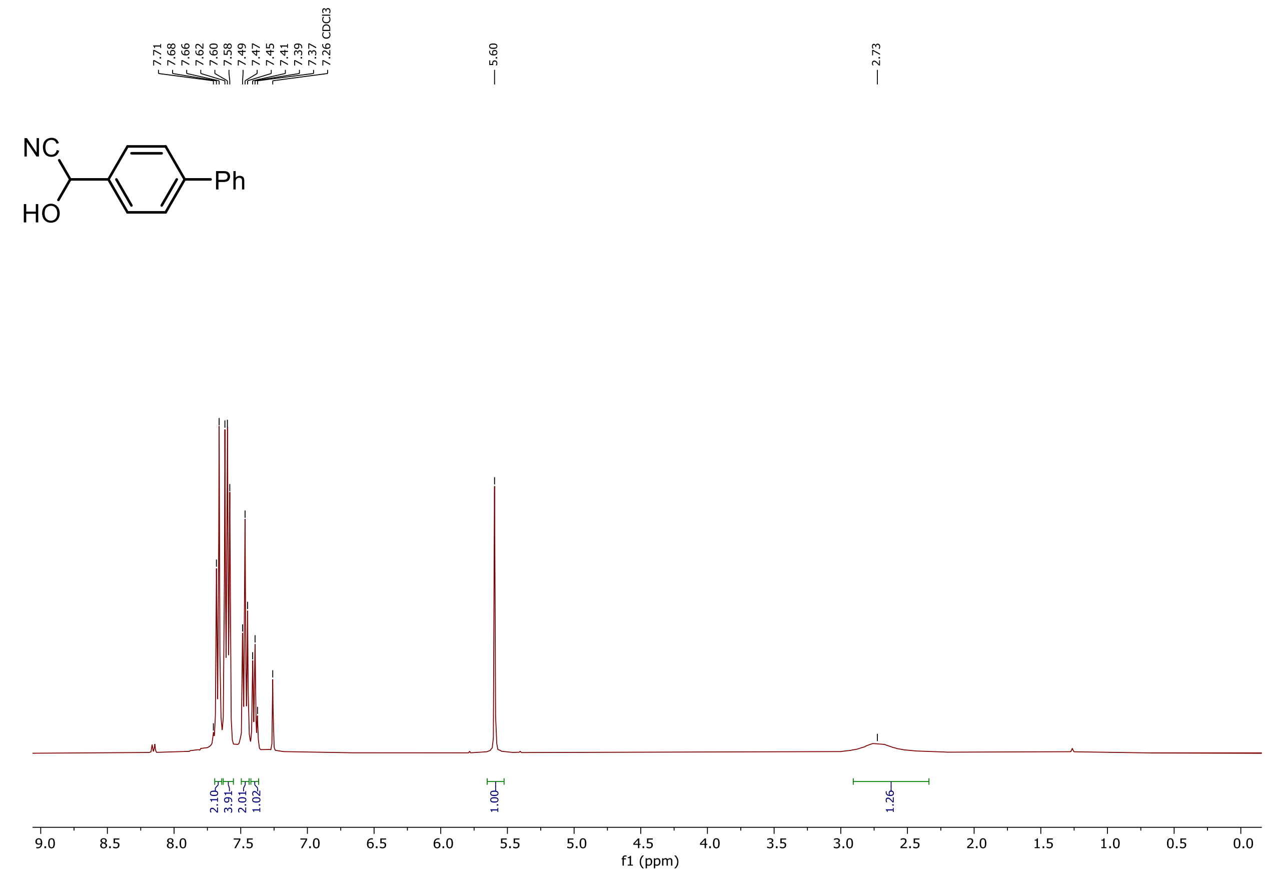

^{13}C NMR (101 MHz, $CDCl_3$) of **P4-S**

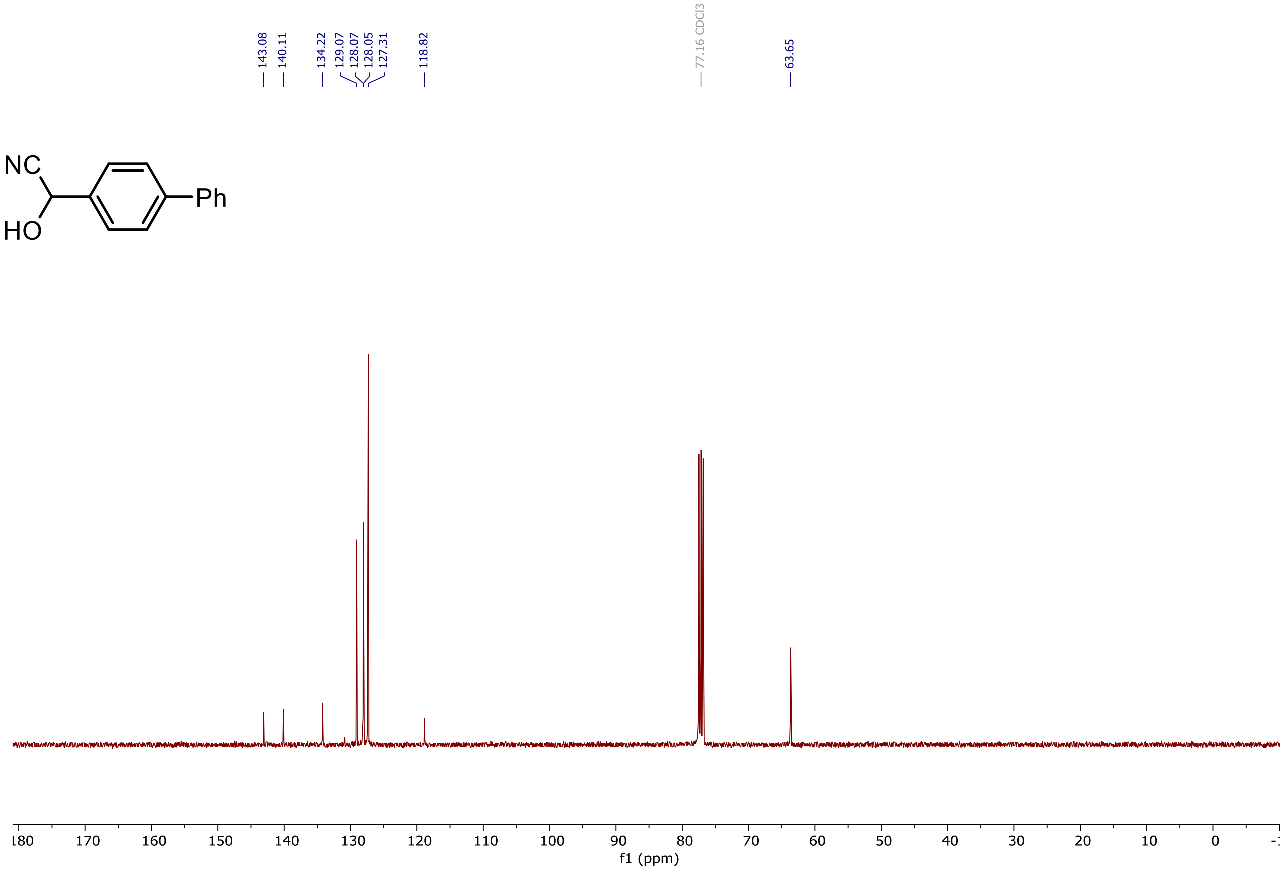

^{1}H NMR (400 MHz, $CDCl_3$) of **P5-S** (see procedure)

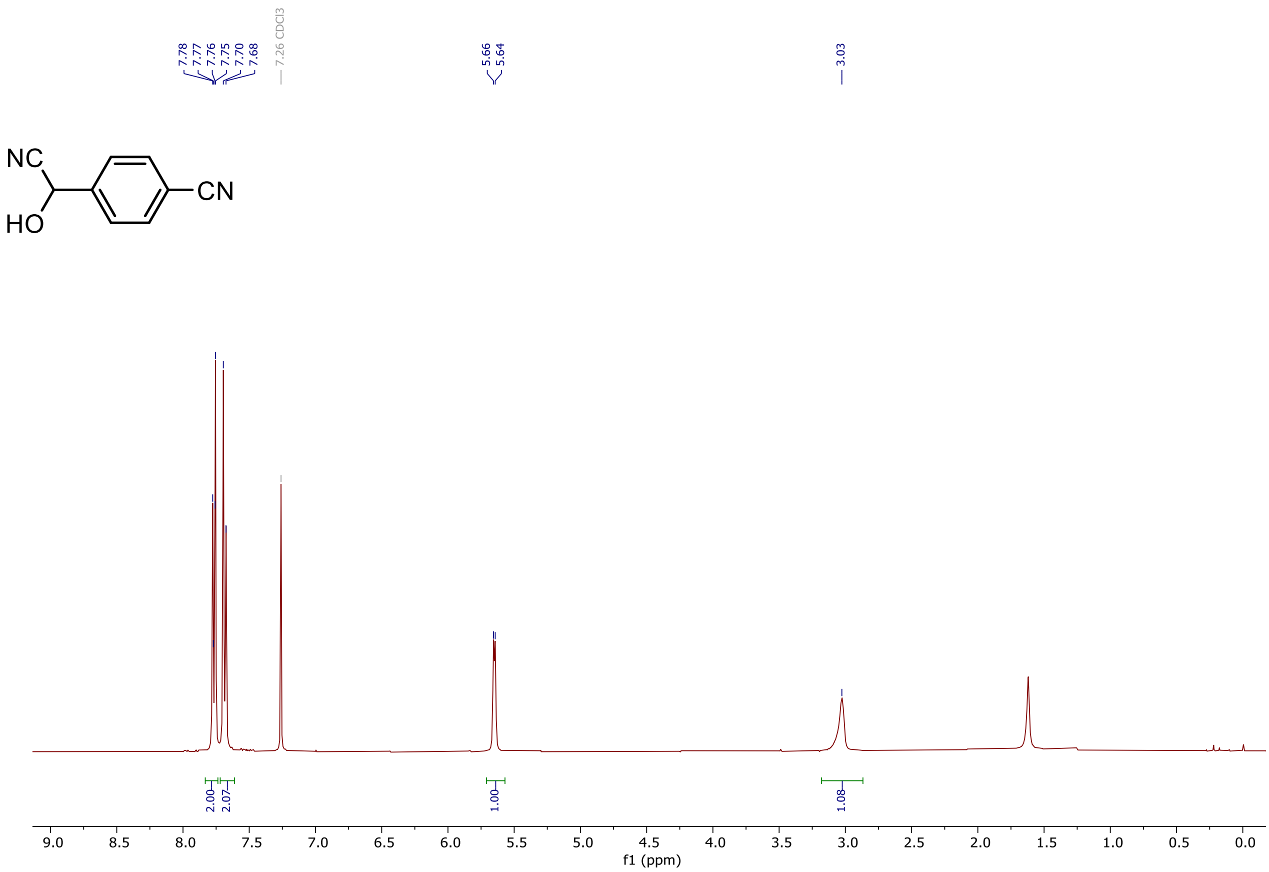

^{13}C NMR (101 MHz, $CDCl_3$) of **P5-S**

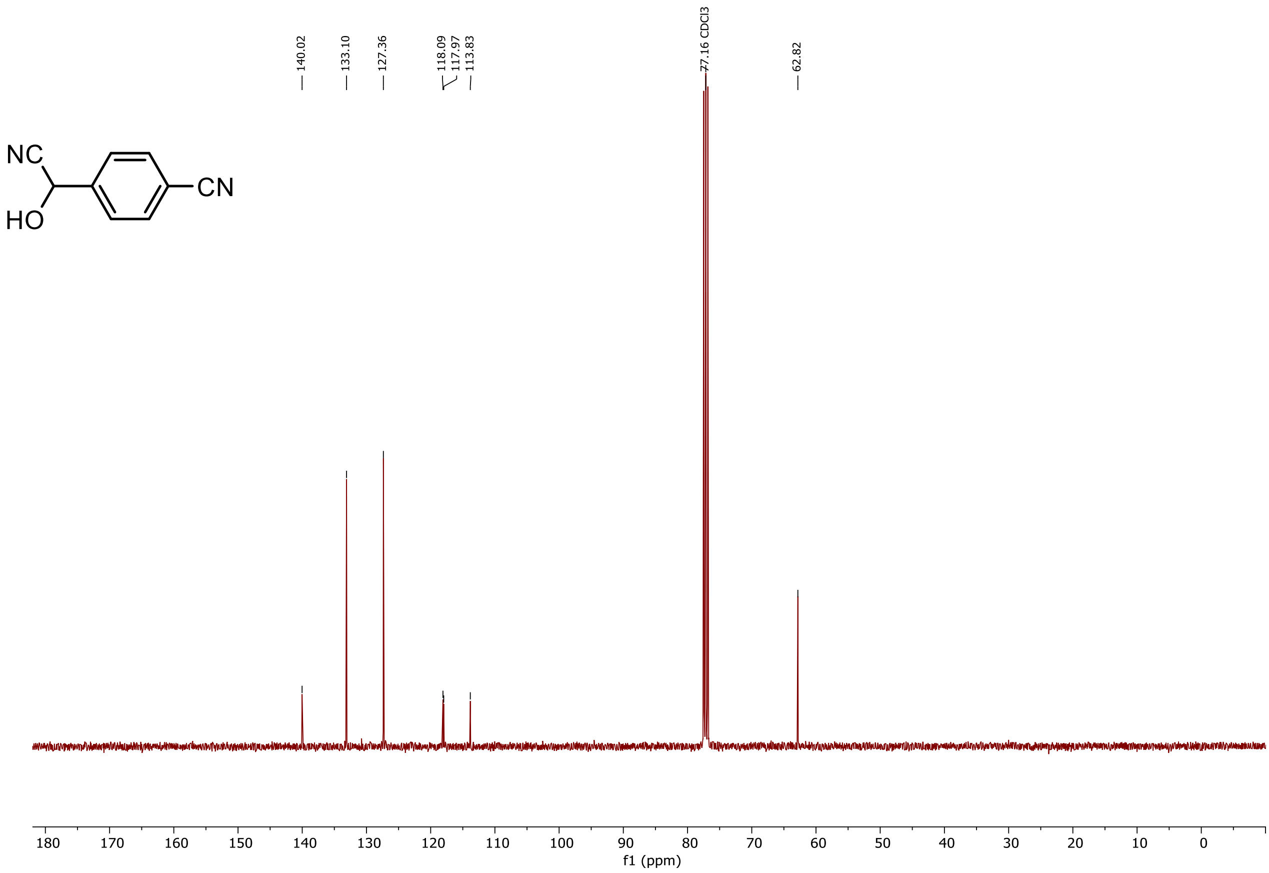

^{1}H NMR (400 MHz, CDCl$_3$) of **P2** (*see procedure*)

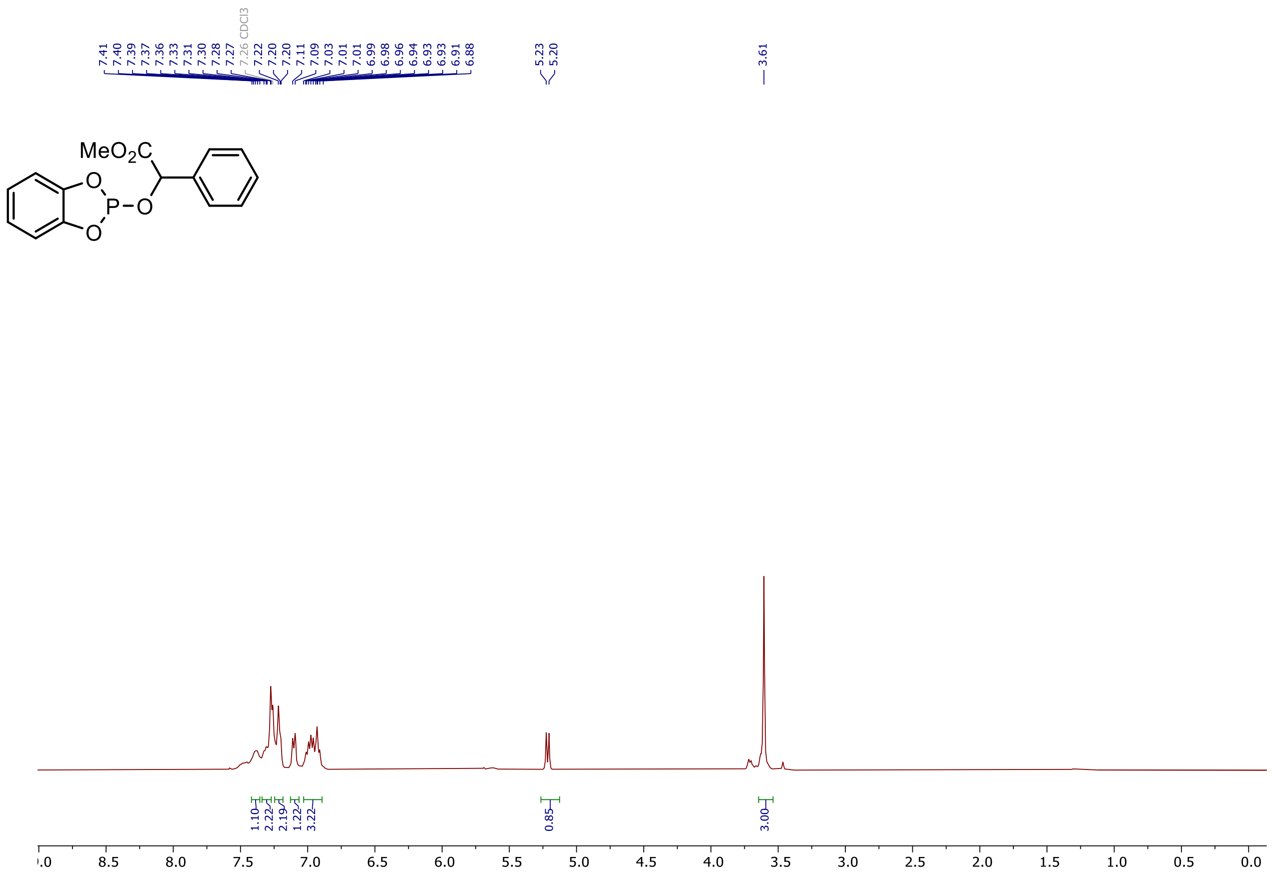

^{13}C NMR (101 MHz, CDCl$_3$) of **P2**

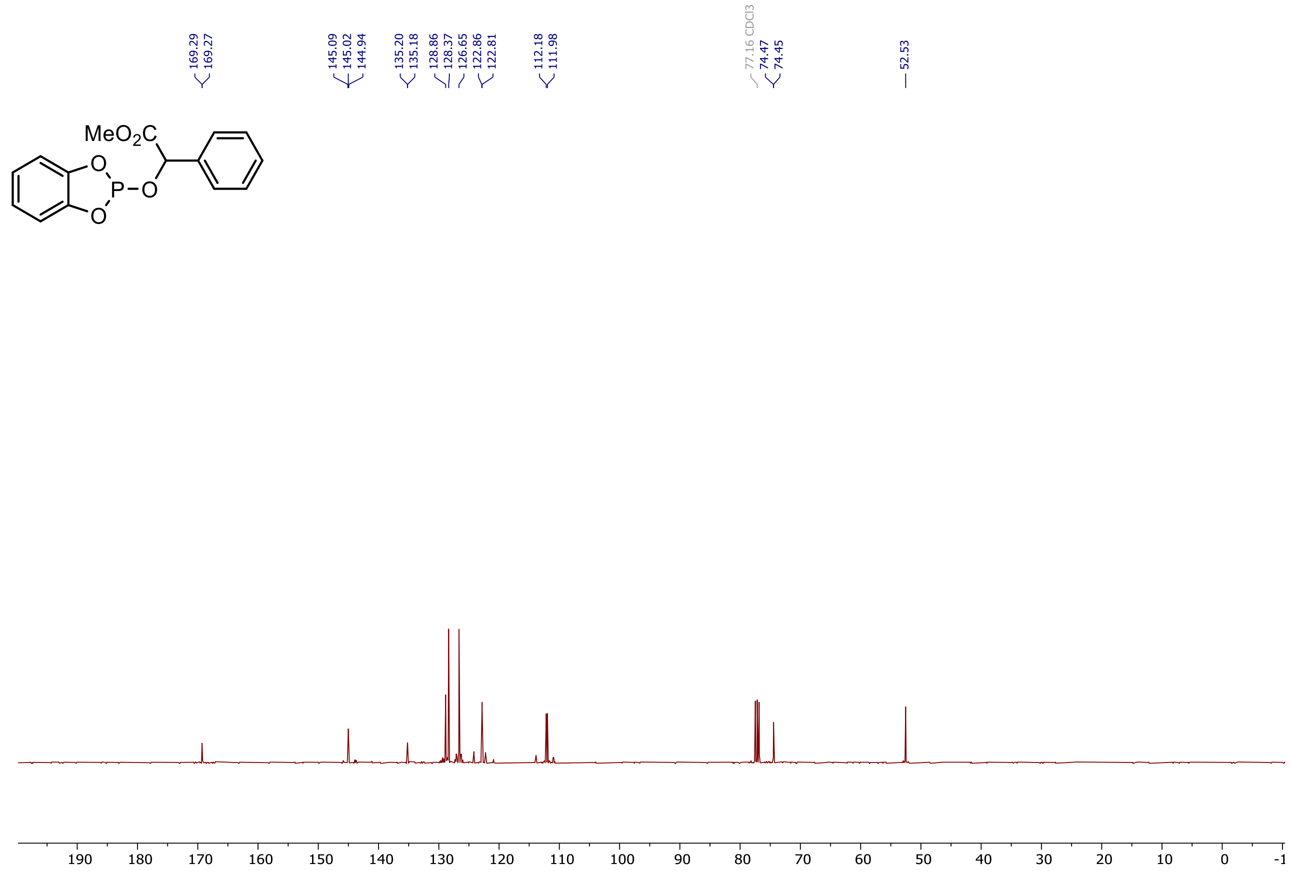

^{31}P NMR (165 MHz, CDCl$_3$) of **P2**

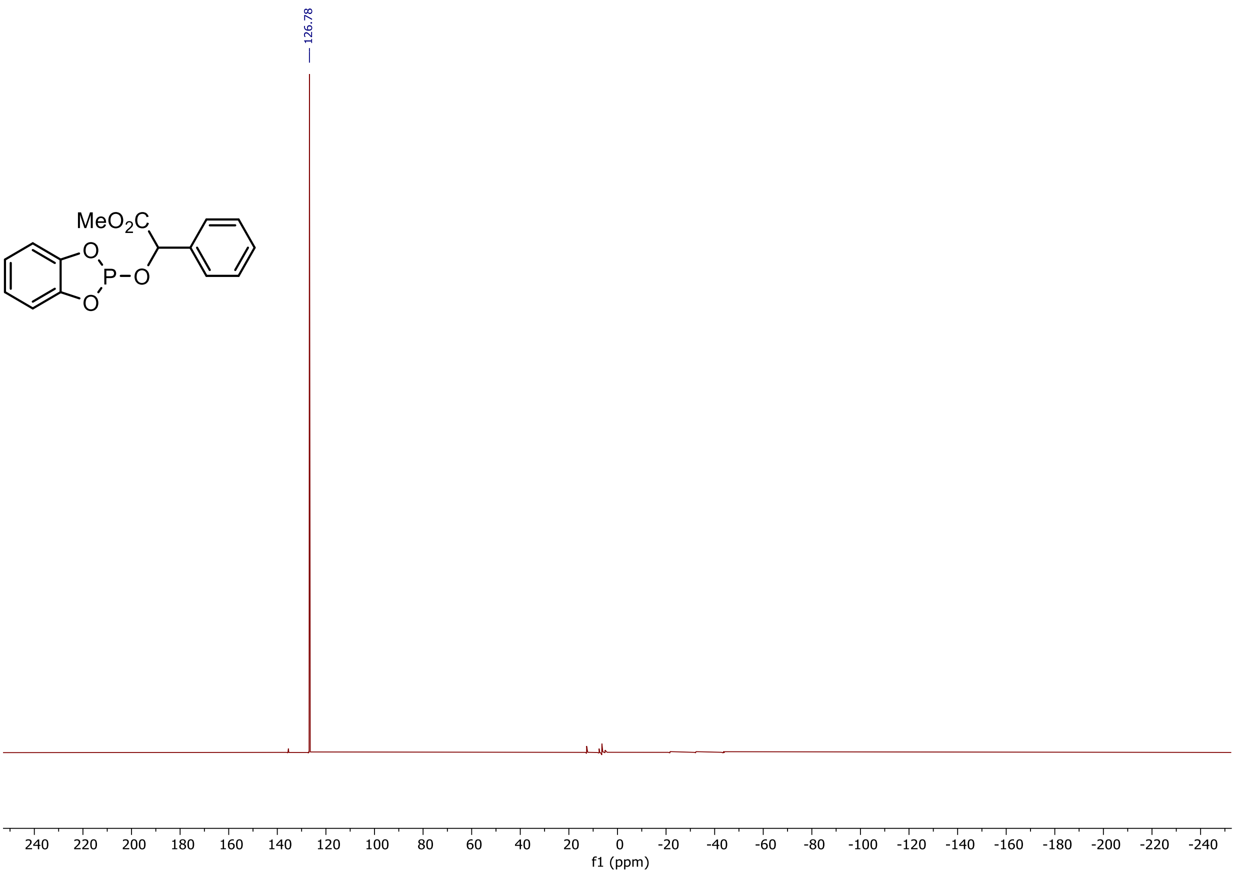

^{1}H NMR (400 MHz, $CDCl_3$) of **P3** (*see procedure*)

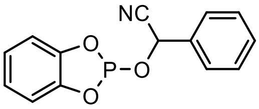

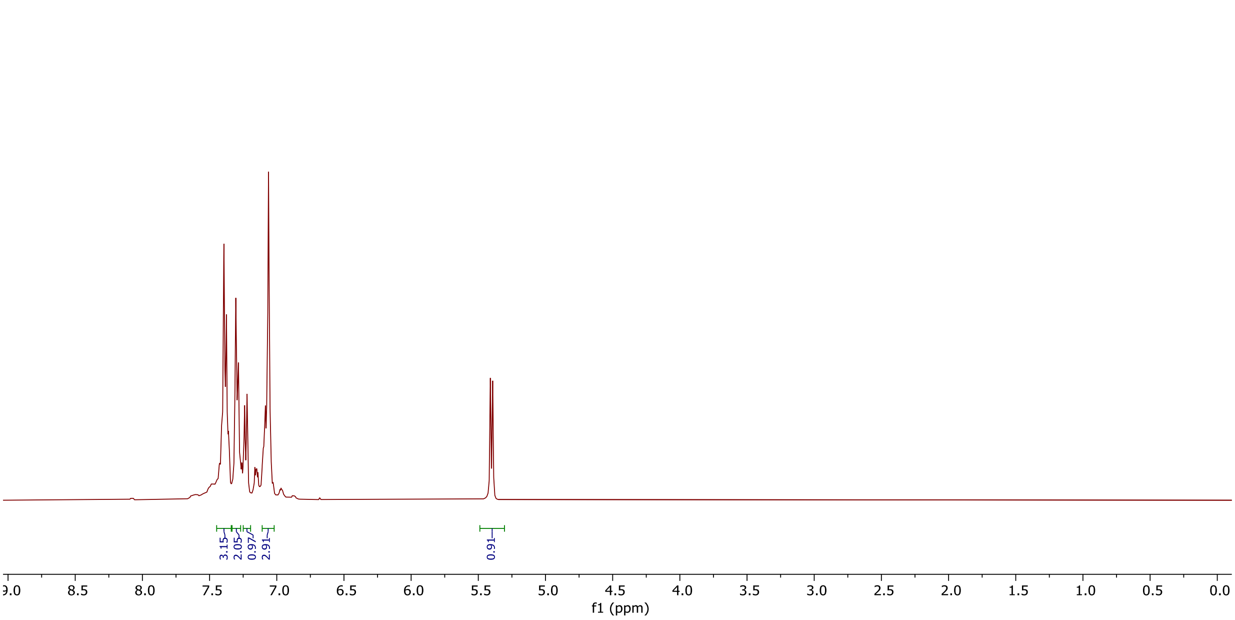

^{13}C NMR (101 MHz, $CDCl_3$) of **P3**

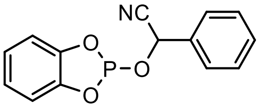

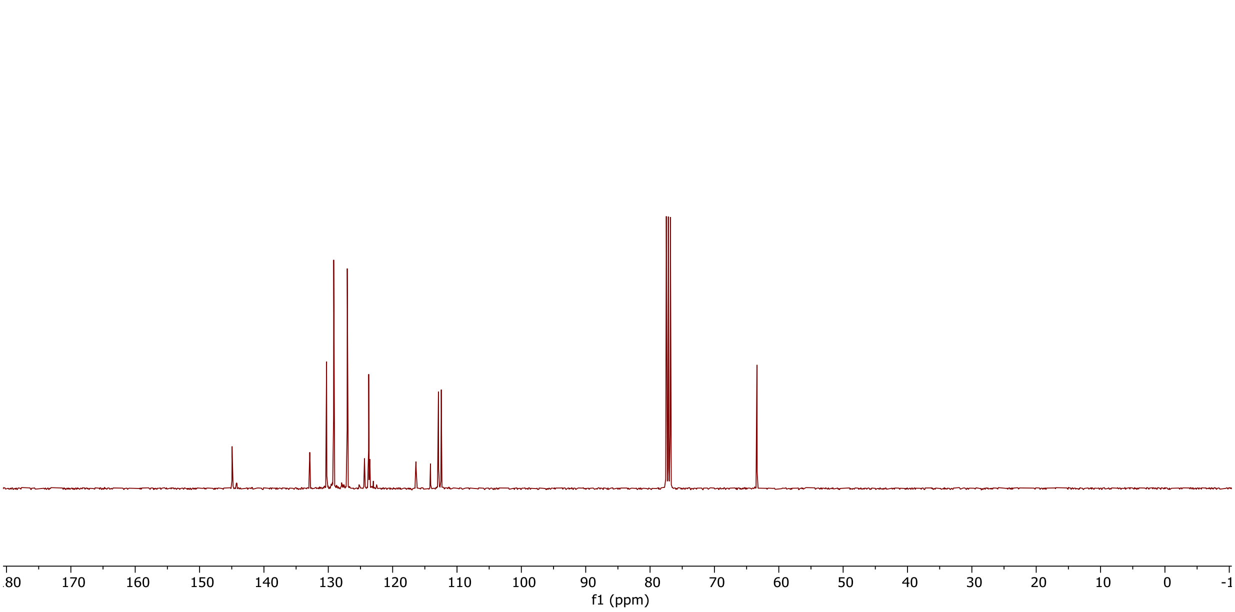

^{31}P NMR (165 MHz, $CDCl_3$) of **P3**

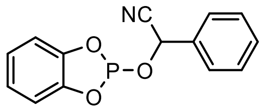

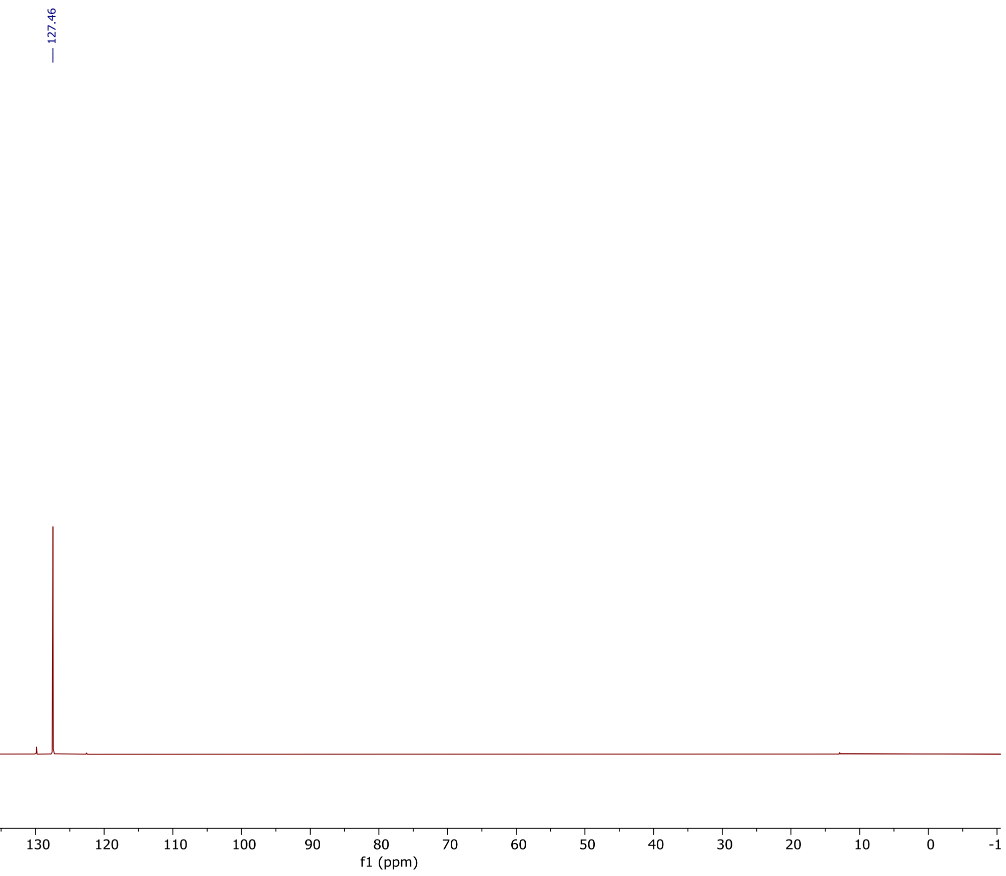

^{1}H NMR (400 MHz, $CDCl_3$) of **P4** (*see procedure*)

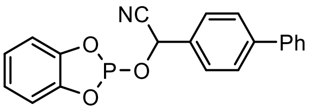

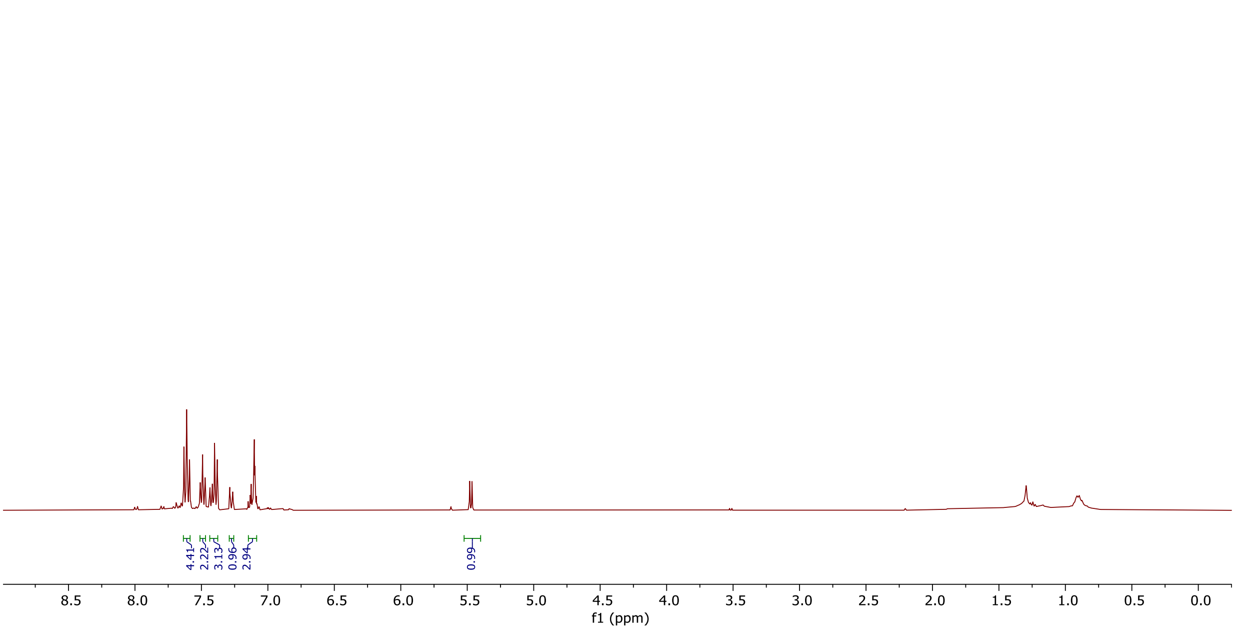

^{13}C NMR (101 MHz, $CDCl_3$) of **P4**

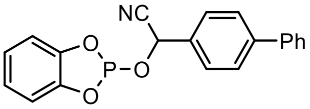

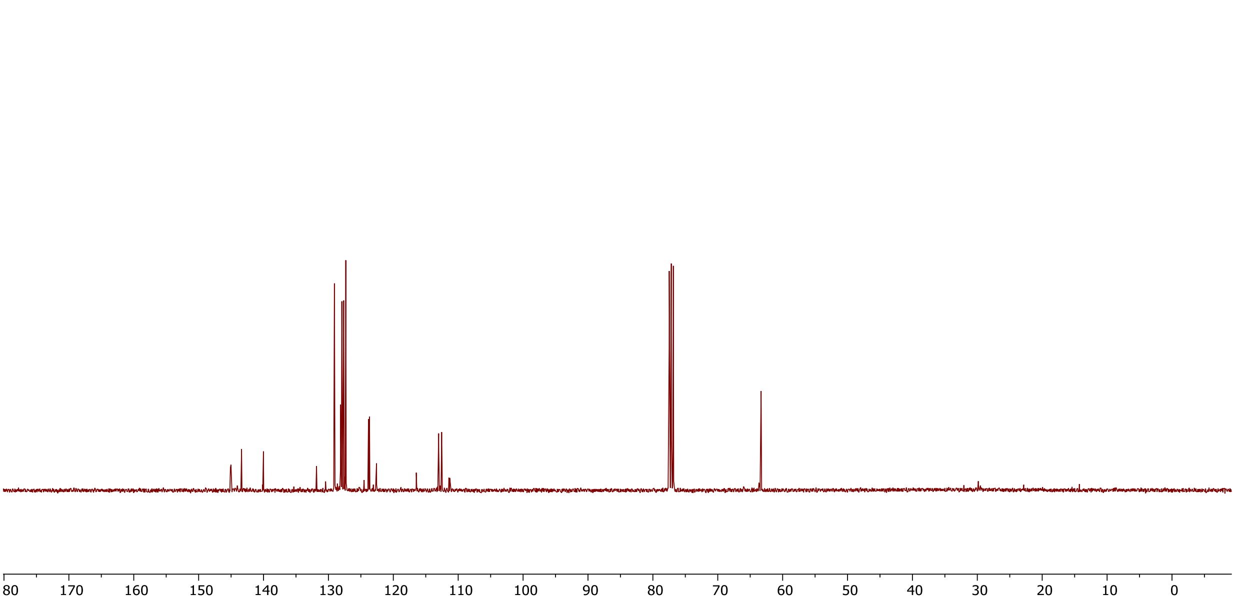

^{31}P NMR (165 MHz, $CDCl_3$) of **P4**

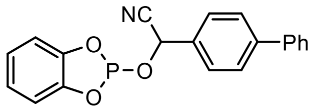

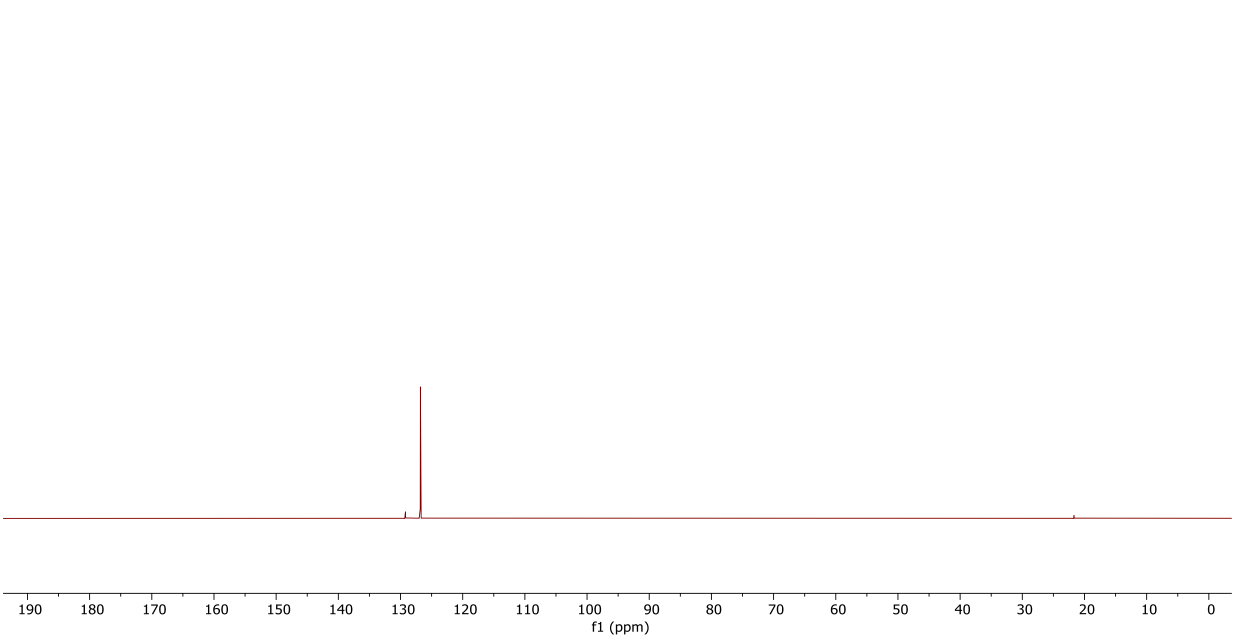

^{1}H NMR (400 MHz, $CDCl_3$) of **P5** (*see procedure*)

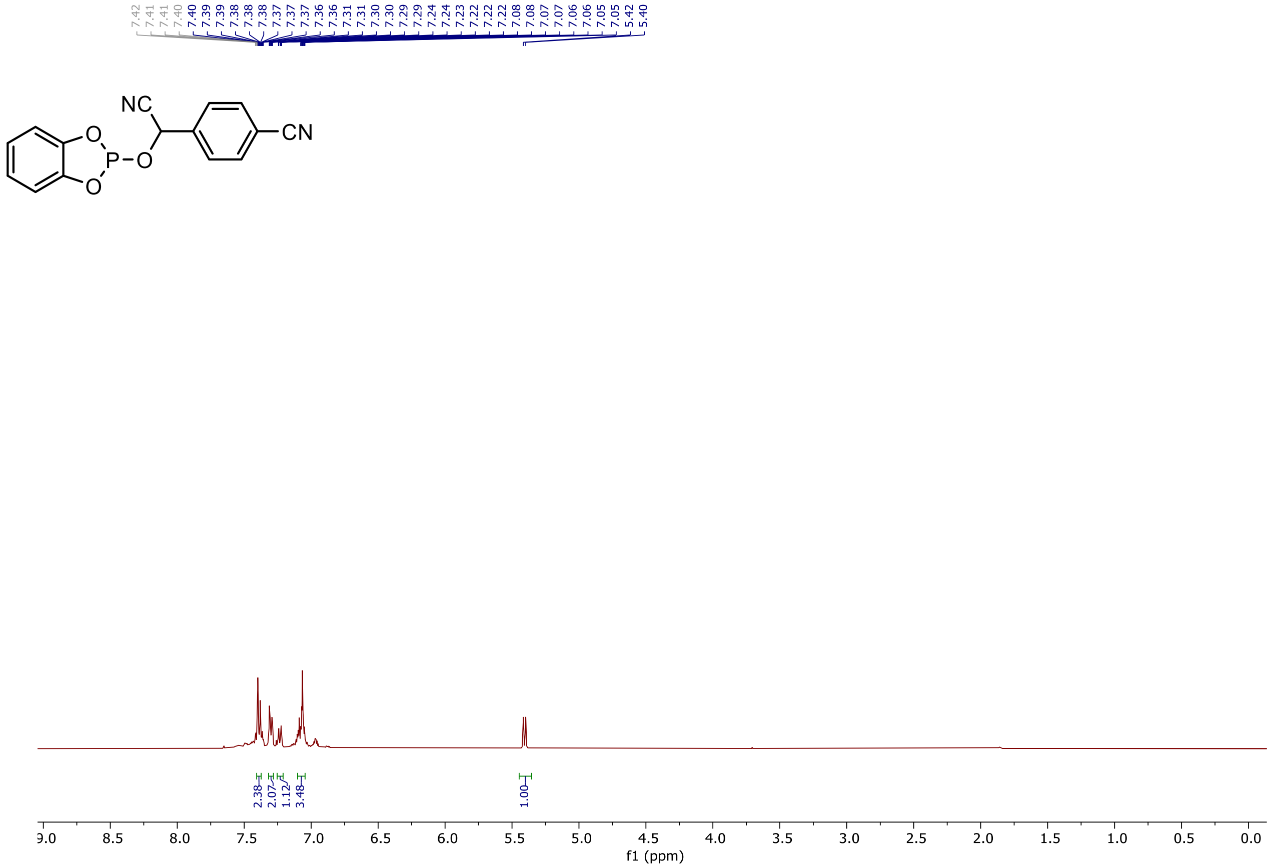

^{13}C NMR (101 MHz, $CDCl_3$) of **P5**

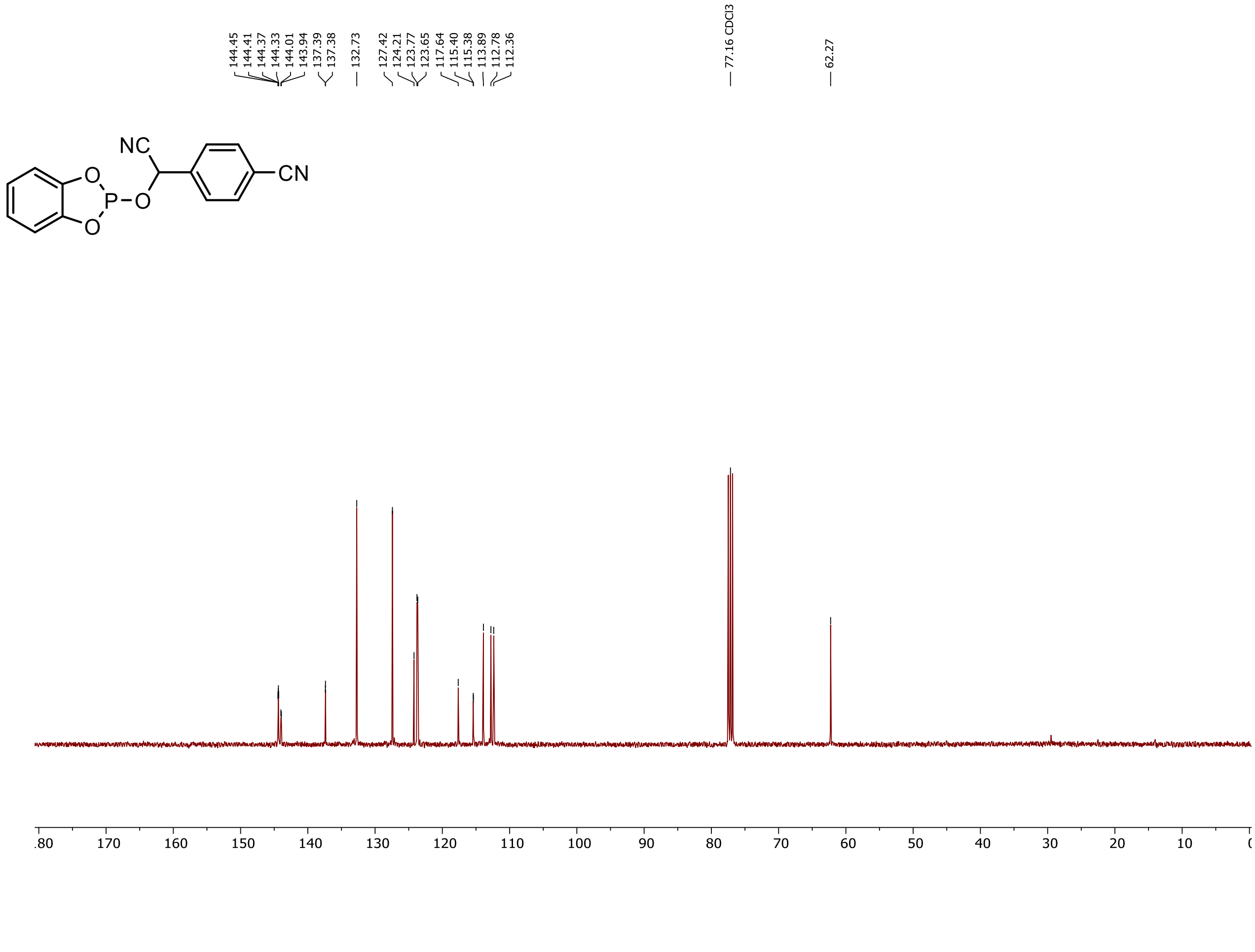

^{31}P NMR (165 MHz, $CDCl_3$) of **P5**

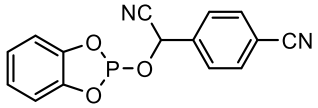

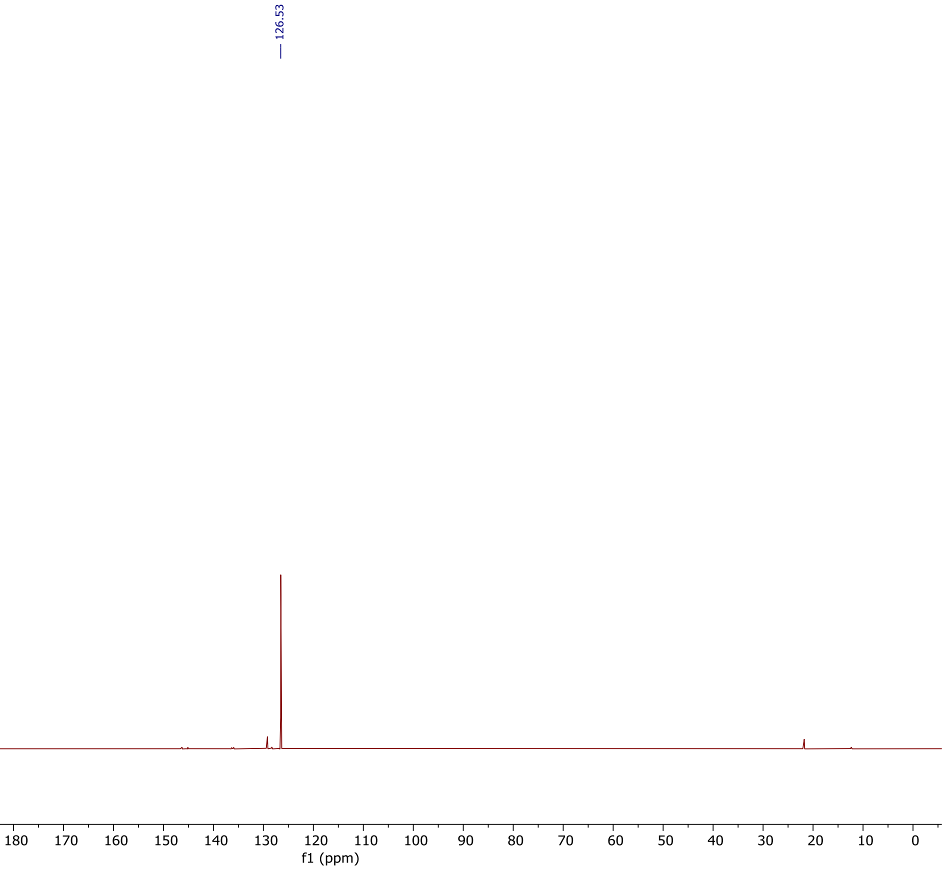

^{1}H NMR (400 MHz, $CDCl_3$) of **P7** (*see procedure*)

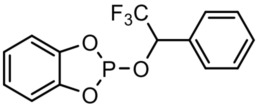

P-7

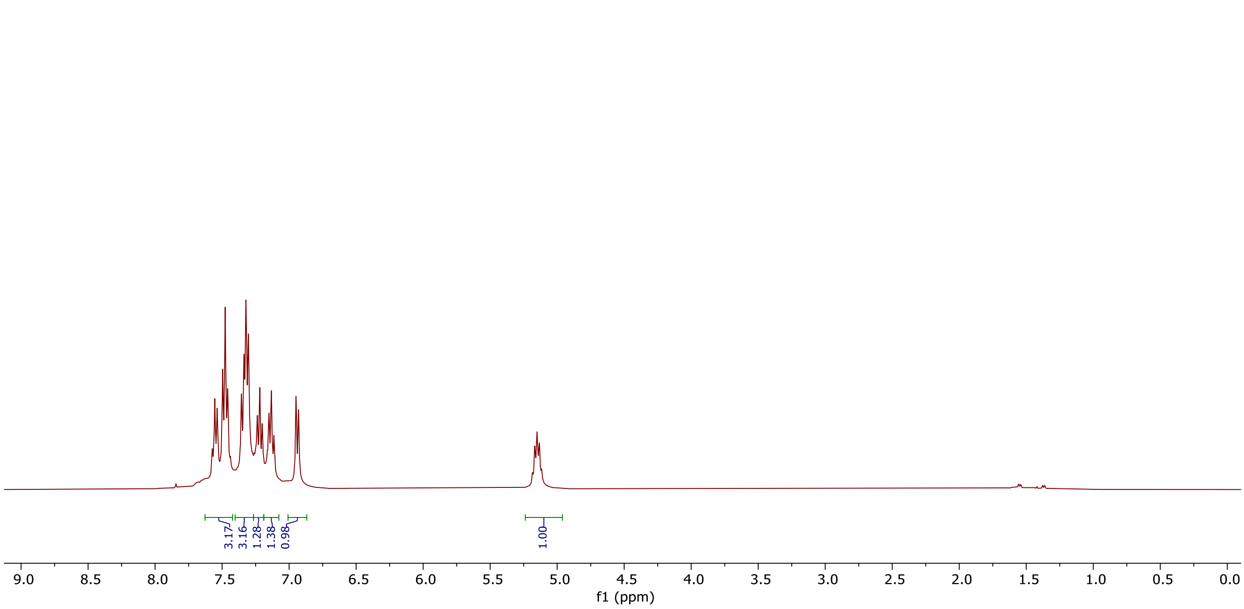

^{13}C NMR (101 MHz, $CDCl_3$) of **P7**

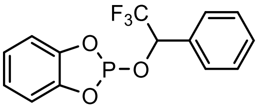

P-7

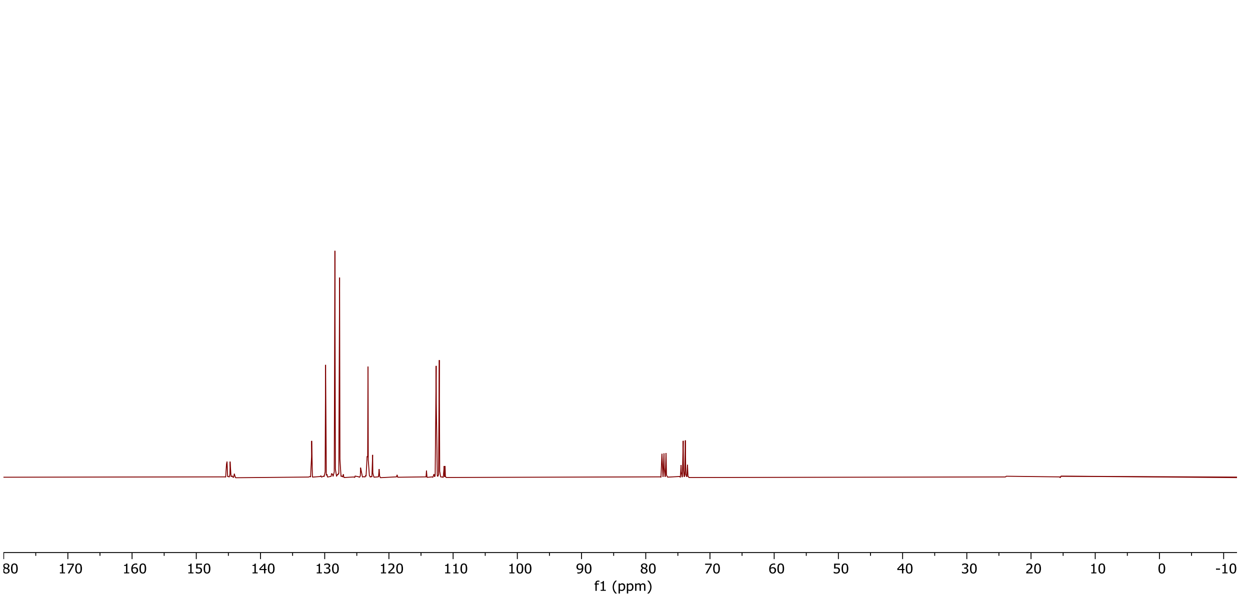

^{31}P NMR (162 MHz, $CDCl_3$) of **P7**

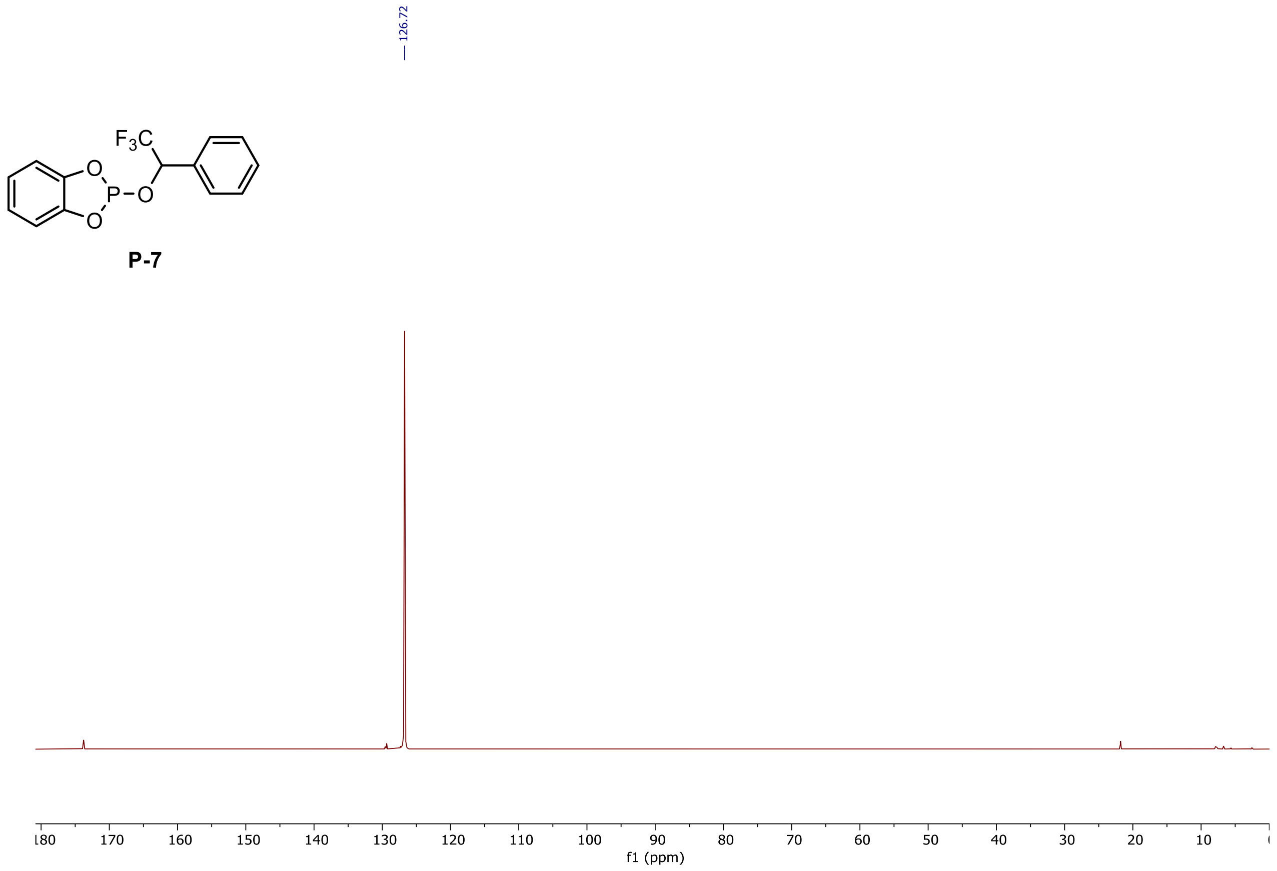

^{19}F NMR (MHz, $CDCl_3$) of **P7**

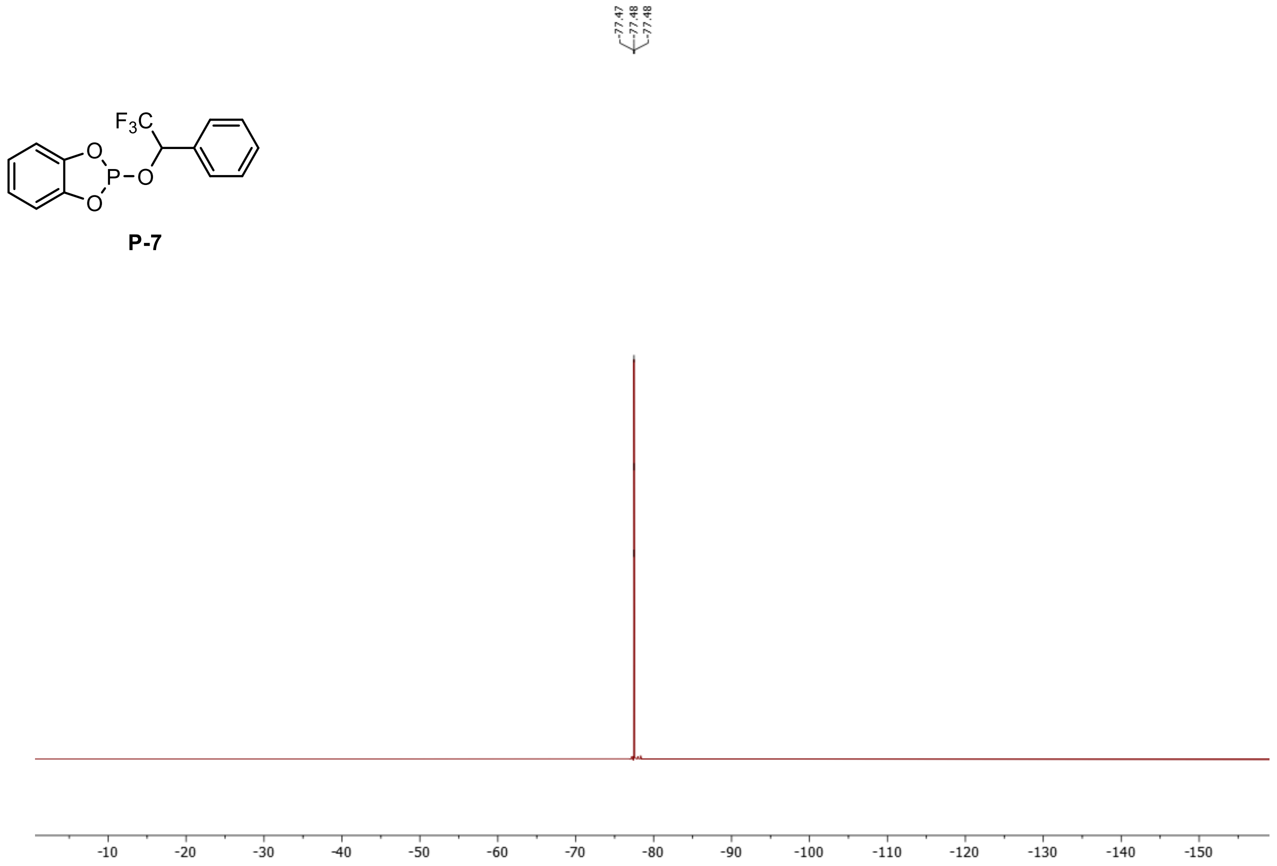

^{1}H NMR (400 MHz, $CDCl_3$) of **P12** (*see procedure*)

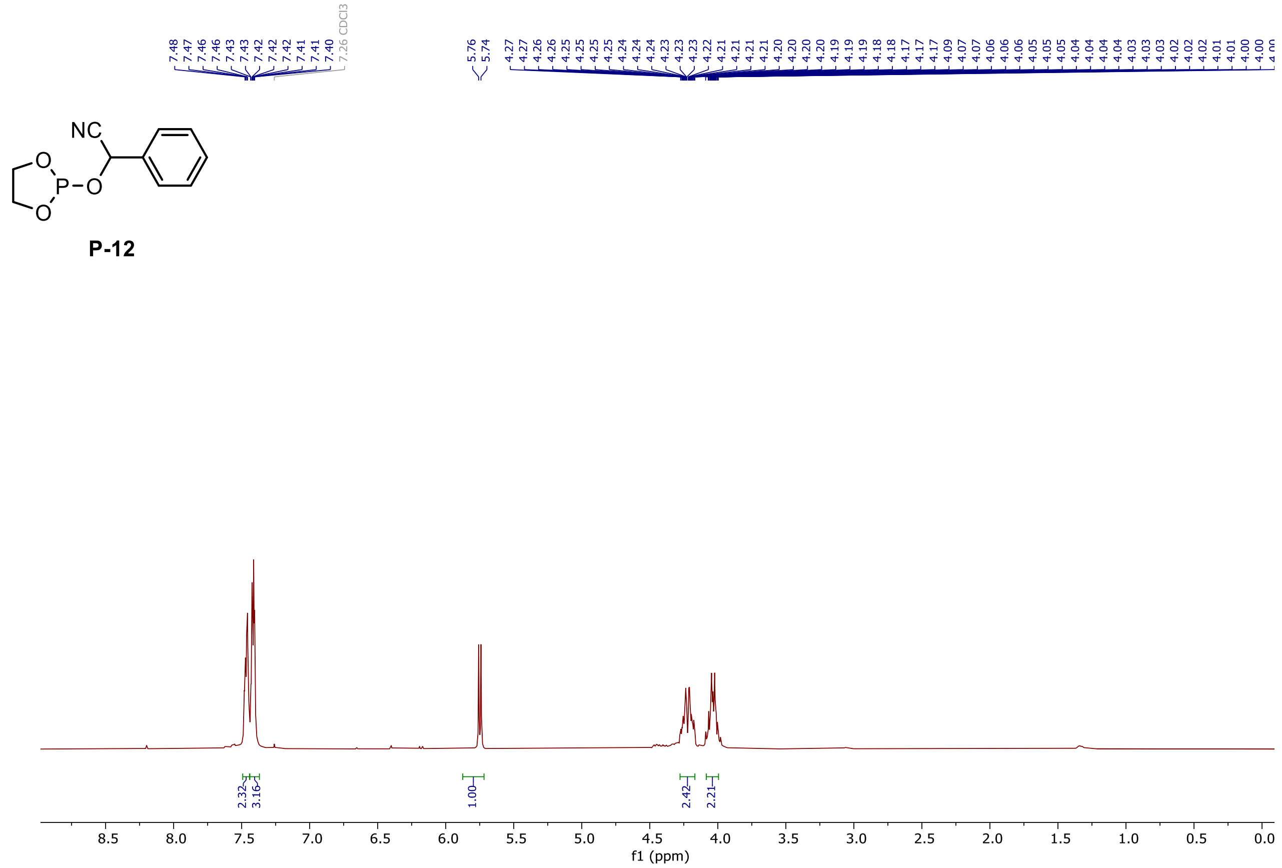

^{13}C NMR (101 MHz, $CDCl_3$) of **P12**

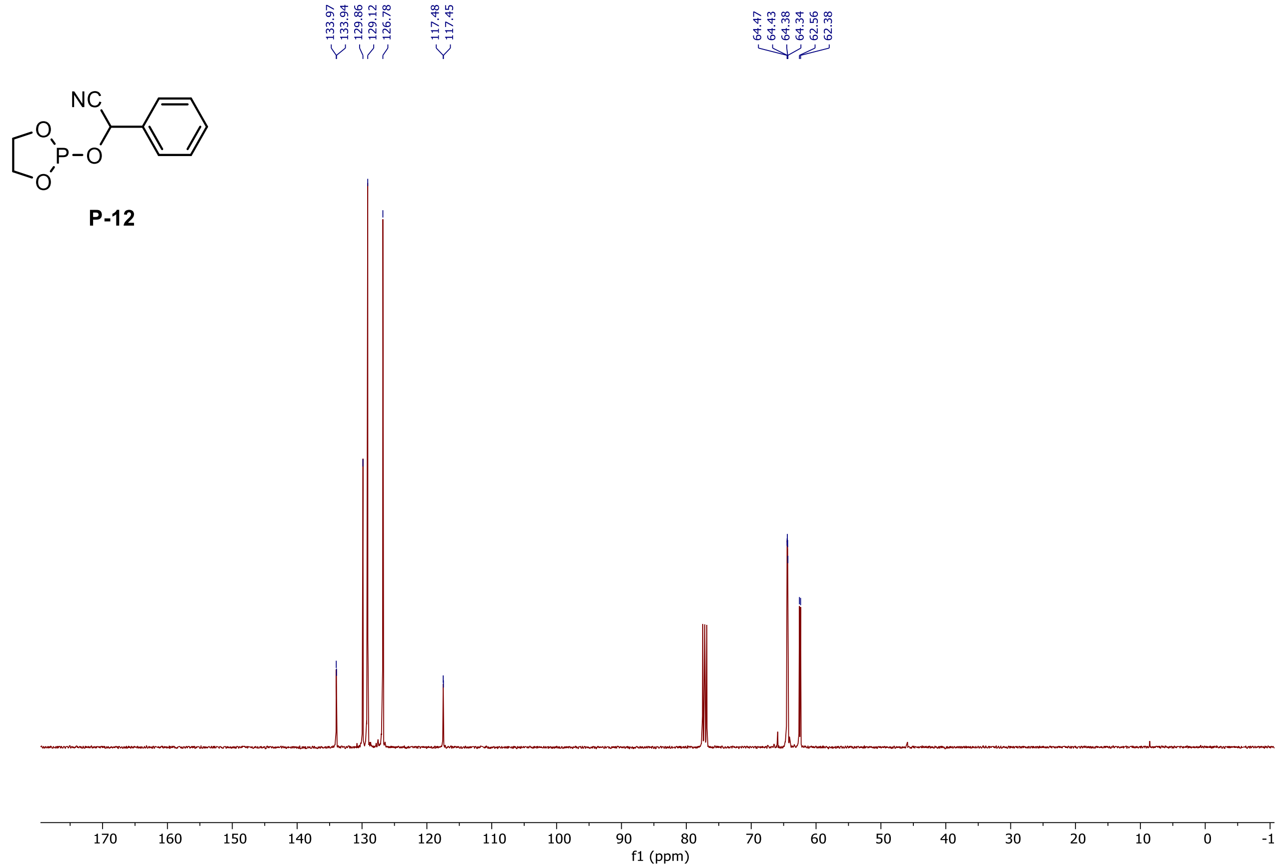

^{31}P NMR (165 MHz, CDCl$_3$) of **P12**

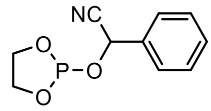

P-12

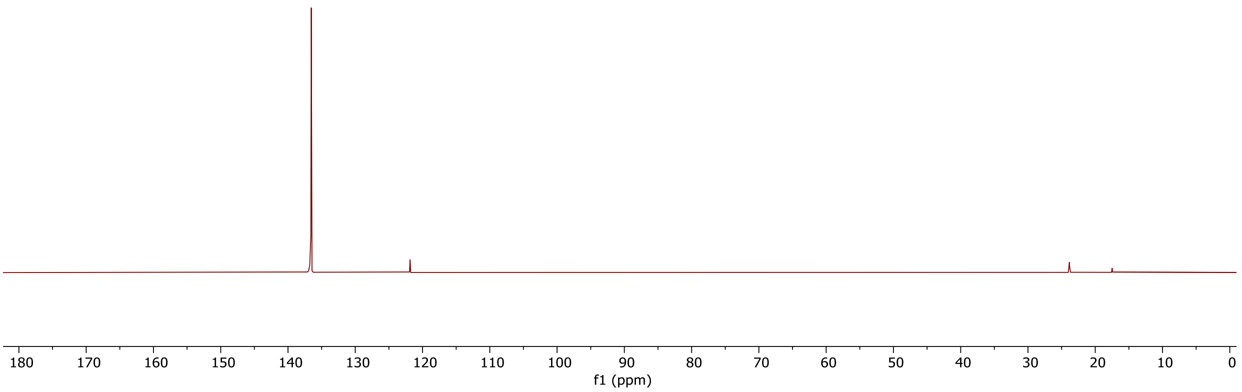

^{1}H NMR (400 MHz, $CDCl_3$) of **P13** (*see procedure*)

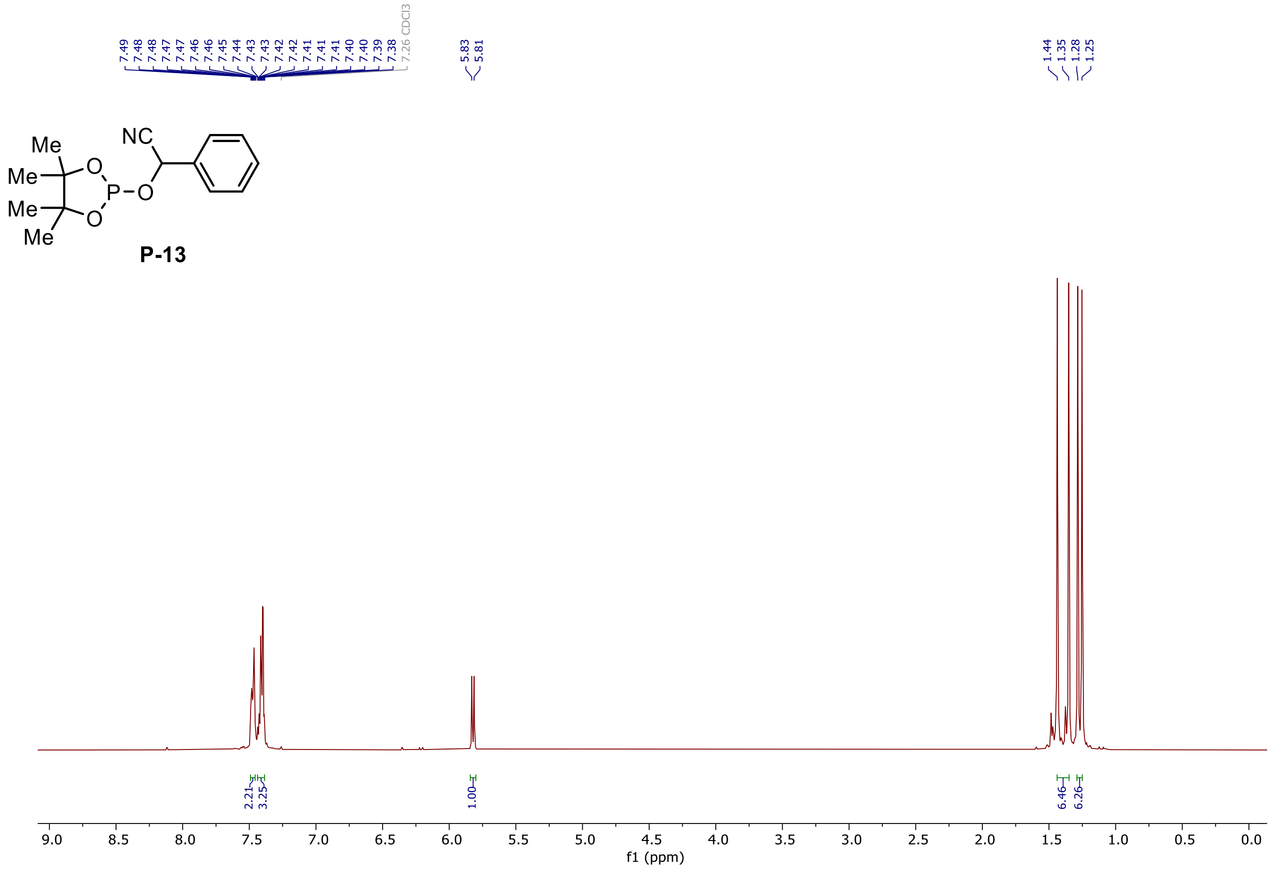

P-13

^{13}C NMR (101 MHz, $CDCl_3$) of **P13**

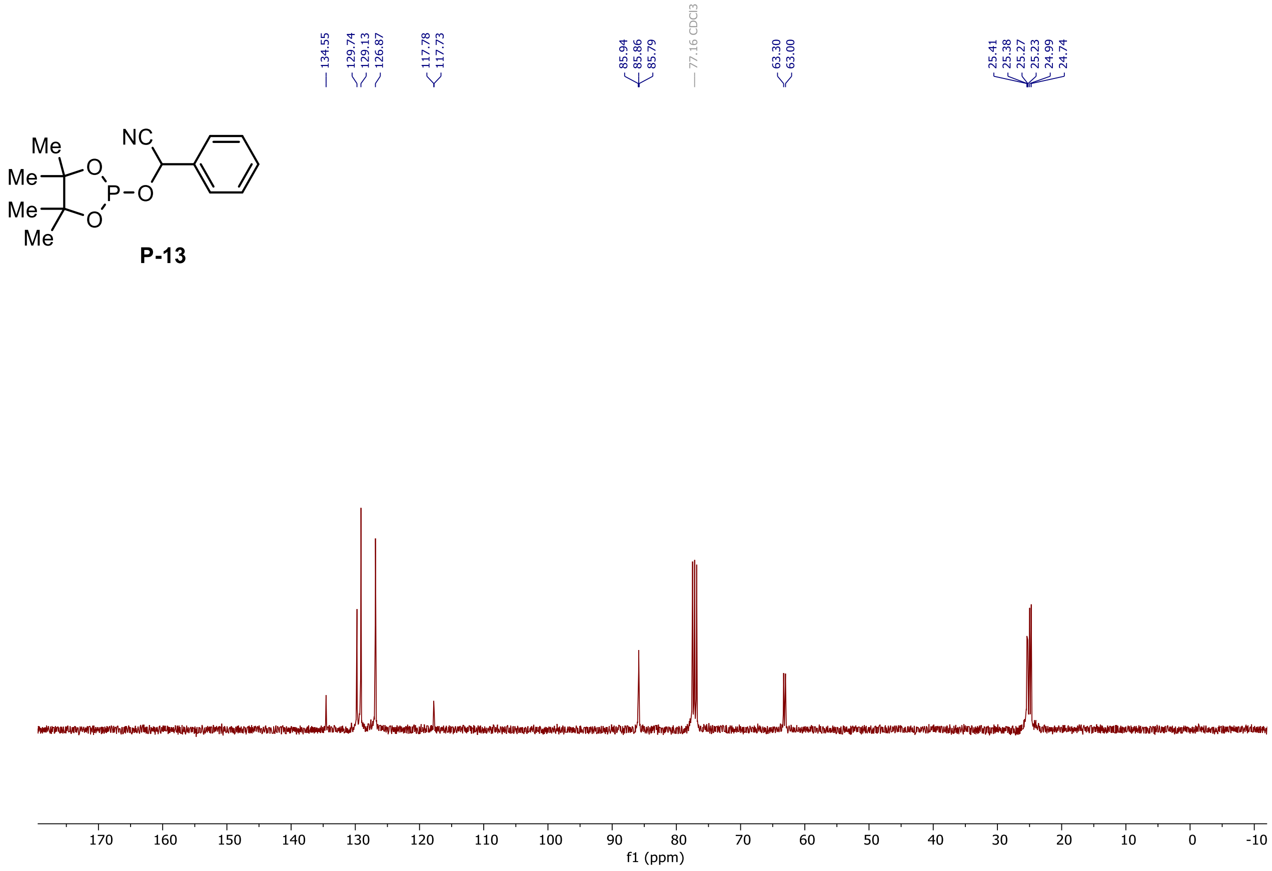

P-13

^{31}P NMR (165 MHz, $CDCl_3$) of **P13**

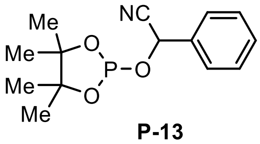

P-13

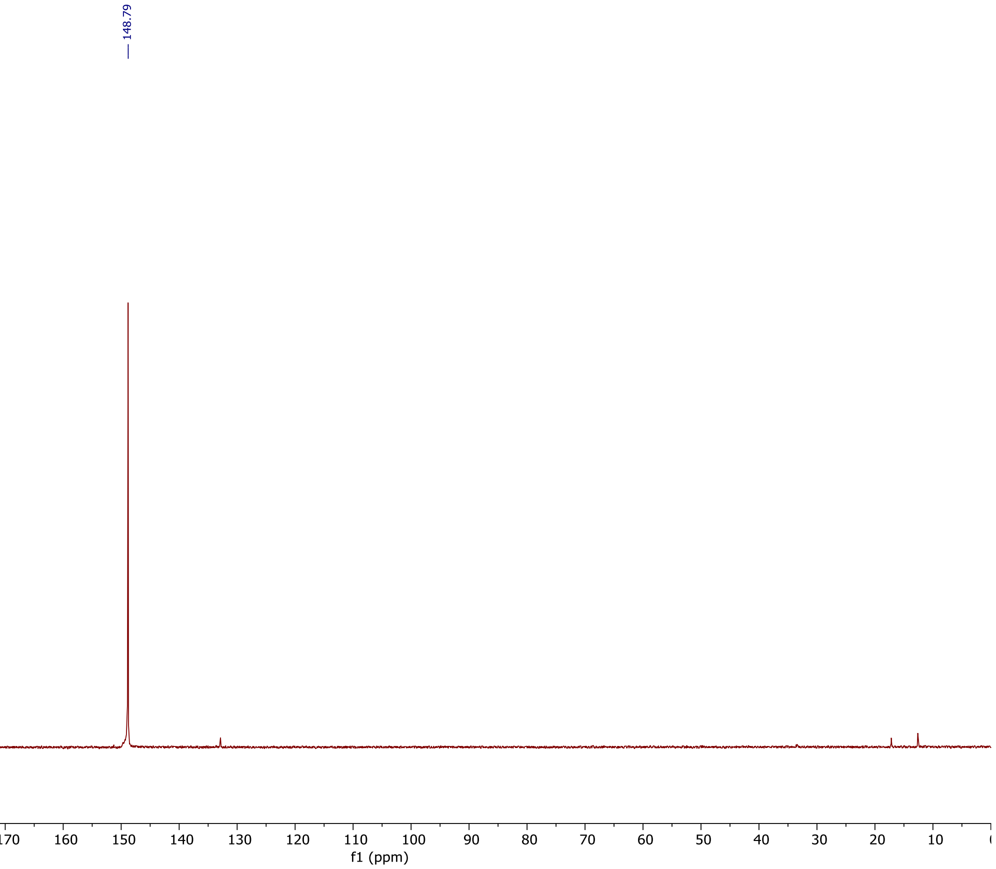

^{1}H NMR (400 MHz, $CDCl_3$) of **P14** (*see procedure*)

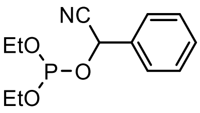

P-14

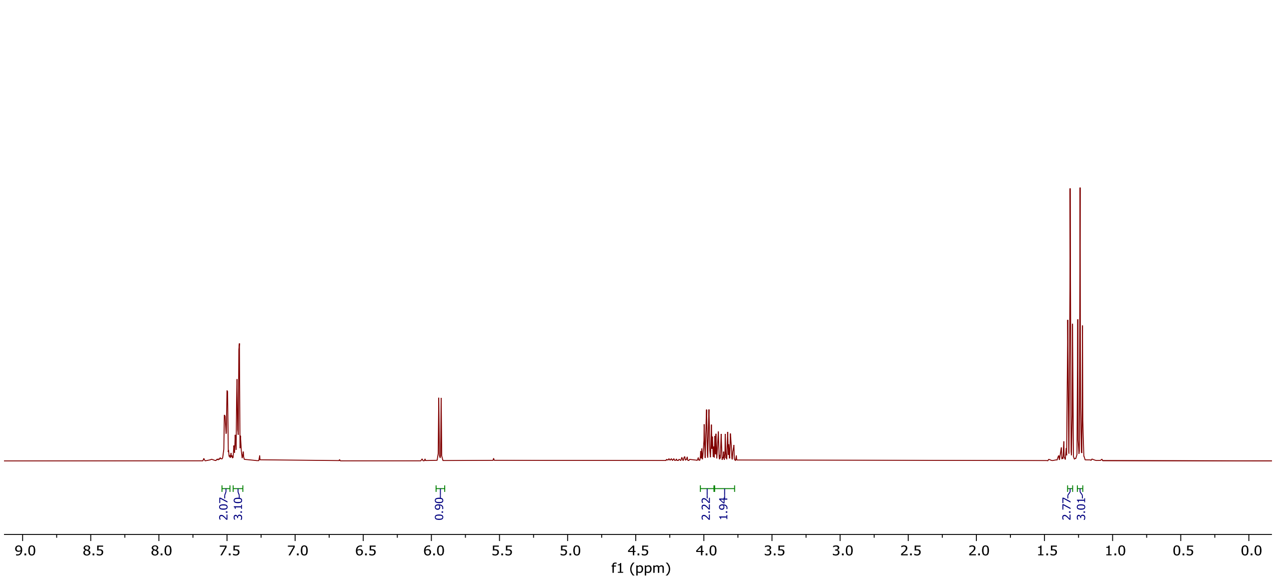

^{13}C NMR (101 MHz, $CDCl_3$) of **P14**

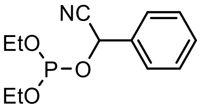

P-14

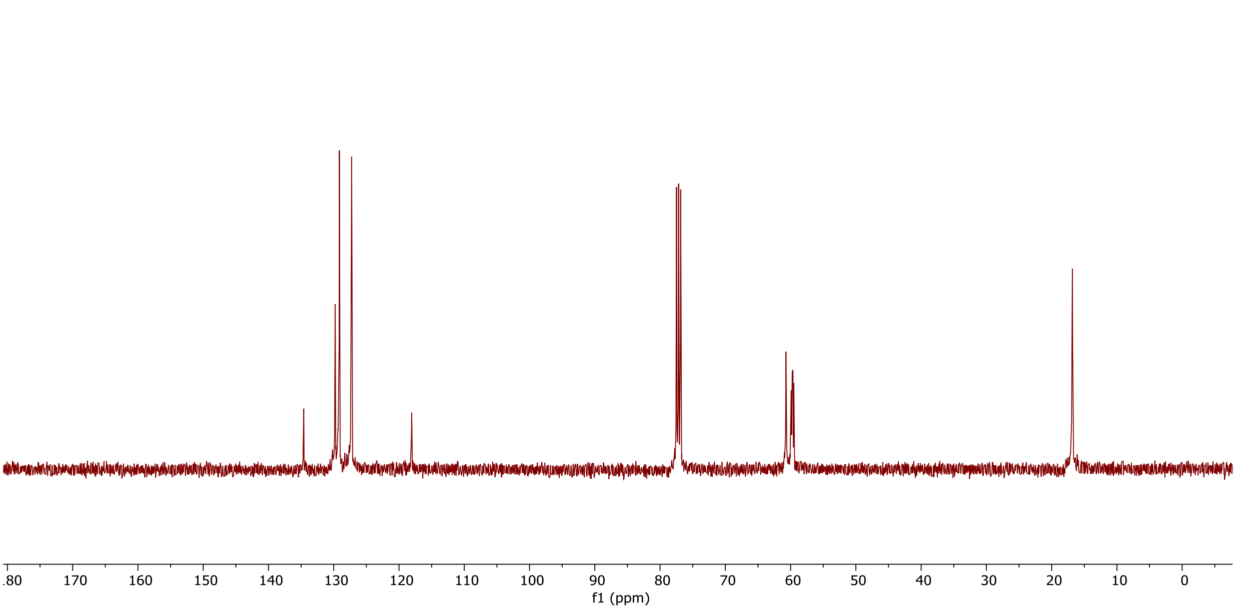

^{31}P NMR (165 MHz, $CDCl_3$) of **P14**

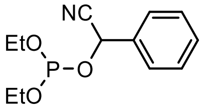

P-14

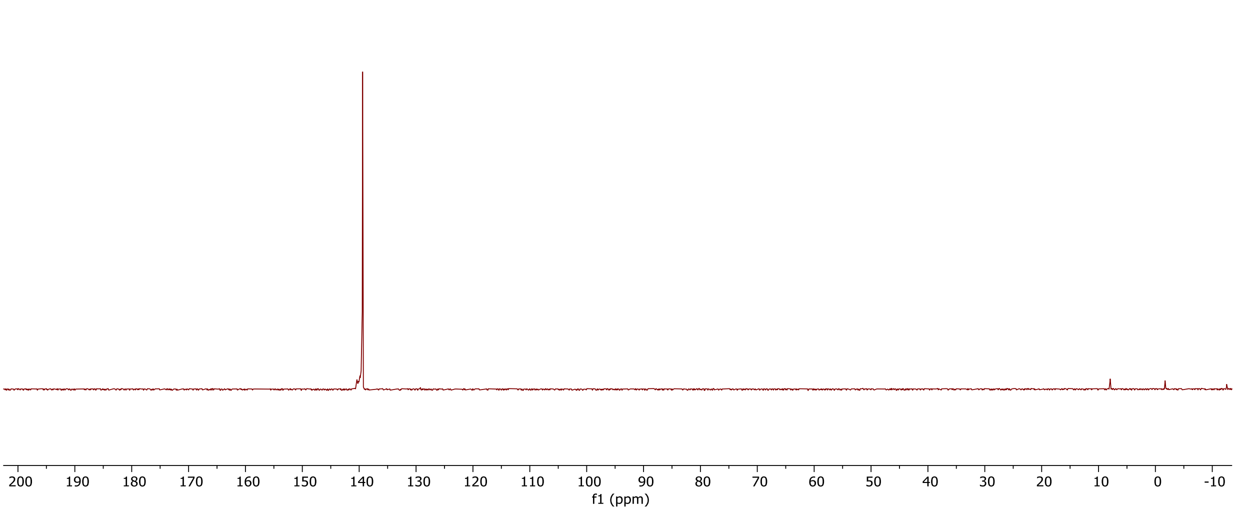

^{1}H NMR (600 MHz, $CDCl_3$) of **1** (*see procedure*)

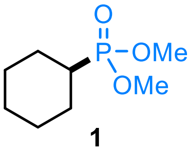

1

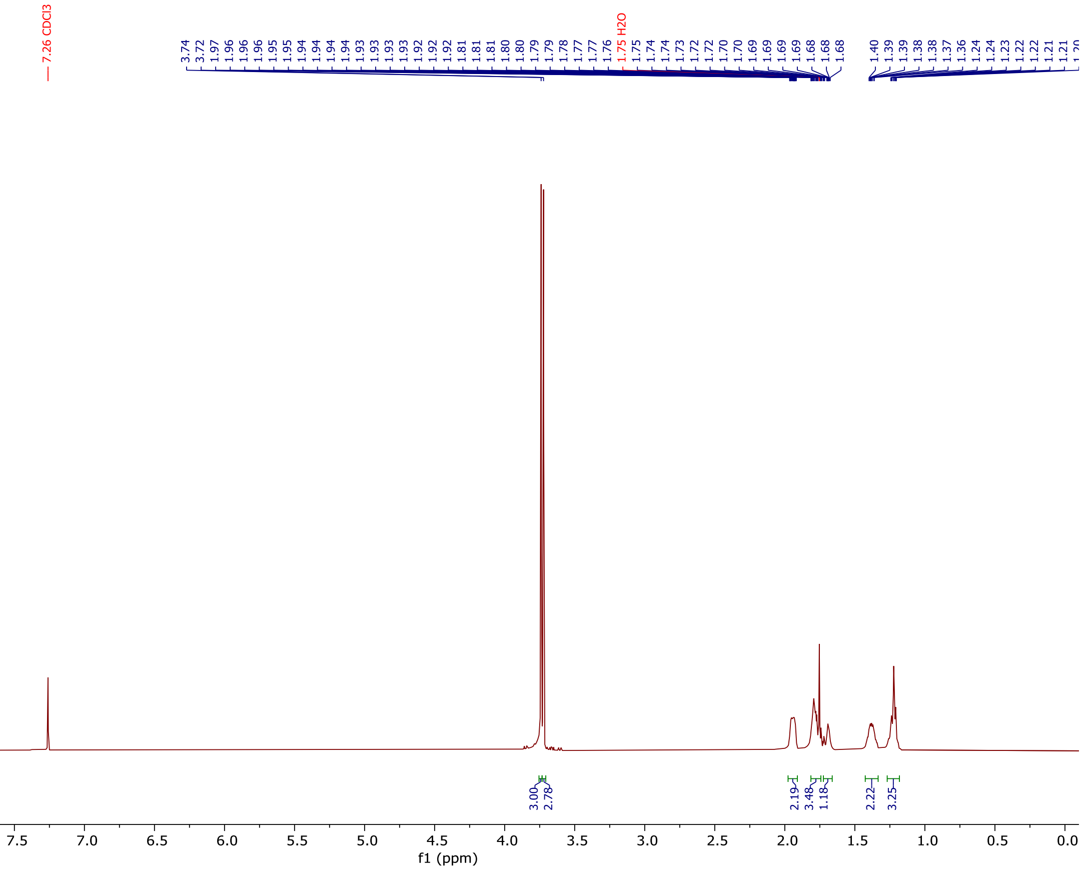

^{13}C NMR (151 MHz, $CDCl_3$) of **1**

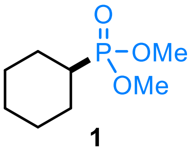

1

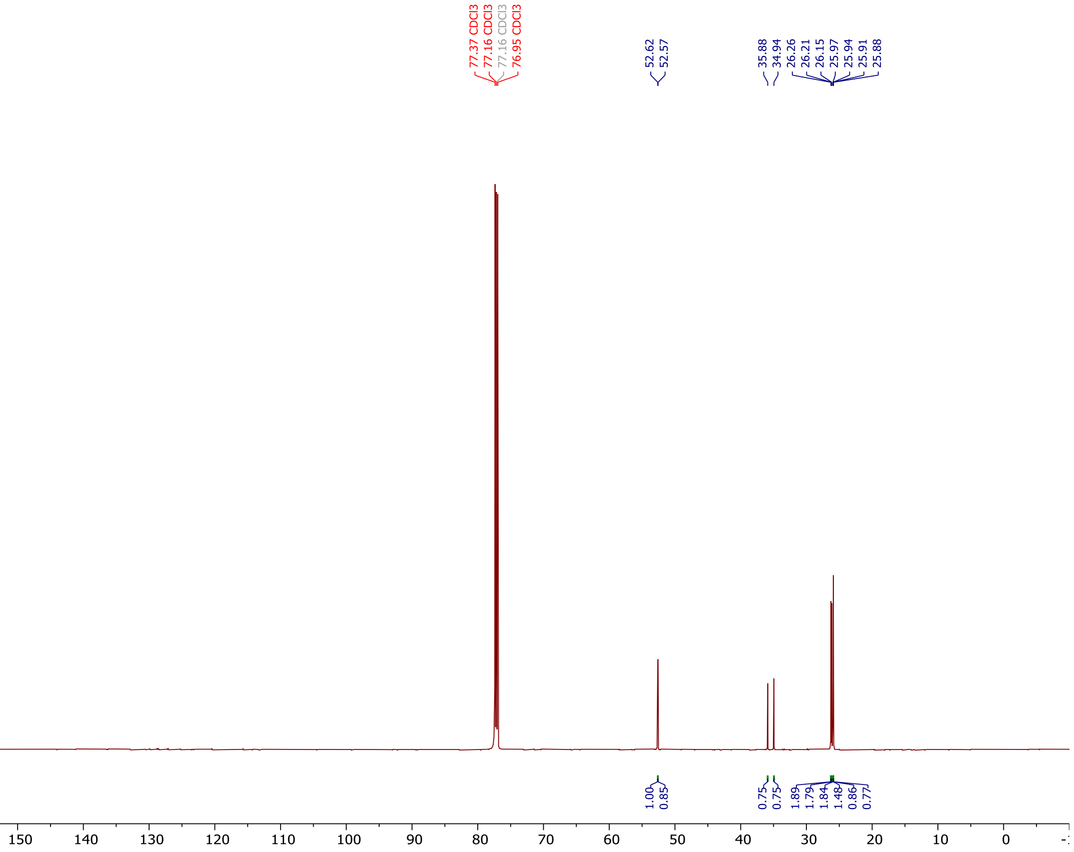

^{31}P NMR (165 MHz, $CDCl_3$) of **1**

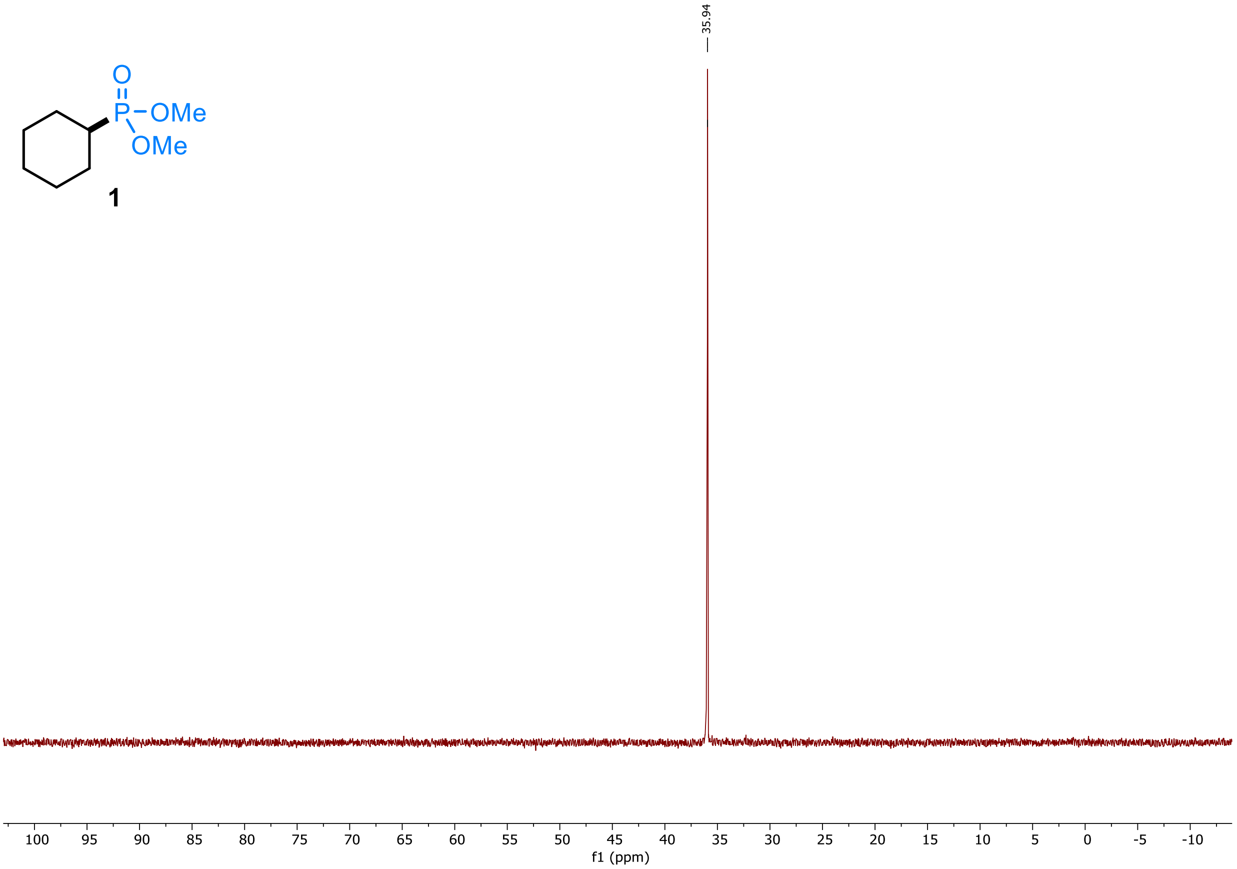

^{1}H NMR (400 MHz, $CDCl_3$) of **2** (*see procedure*)

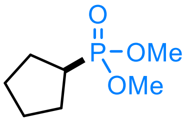

2

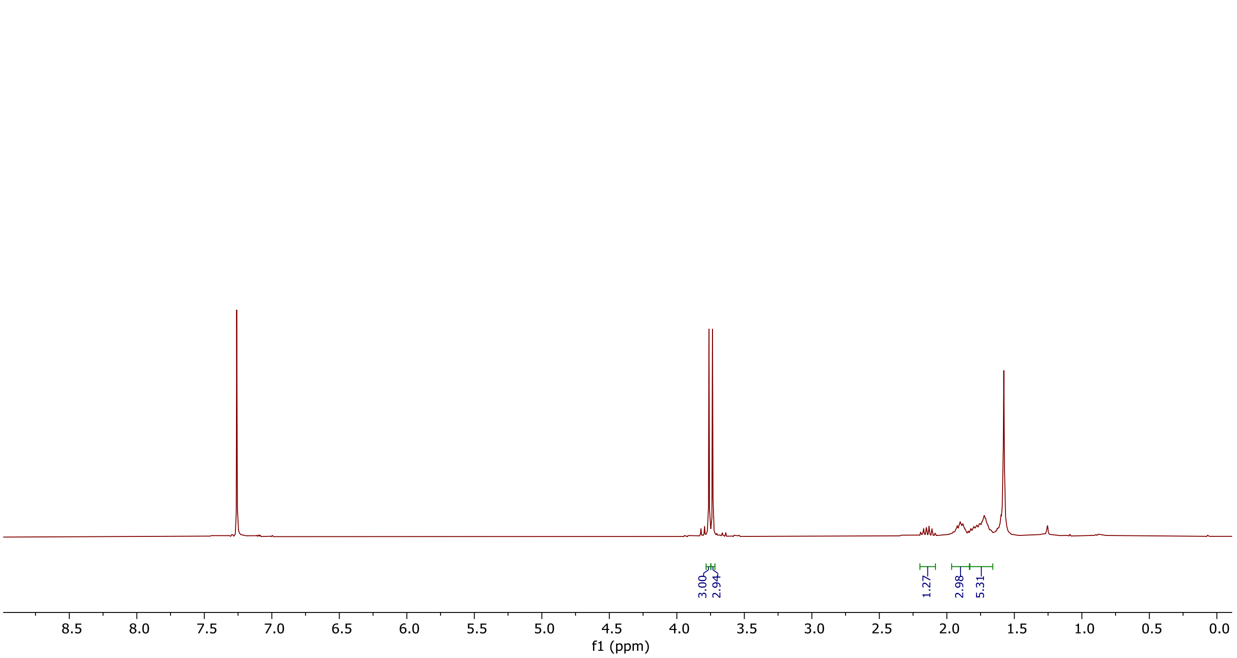

^{13}C NMR (151 MHz, $CDCl_3$) of **2**

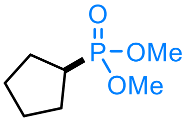

2

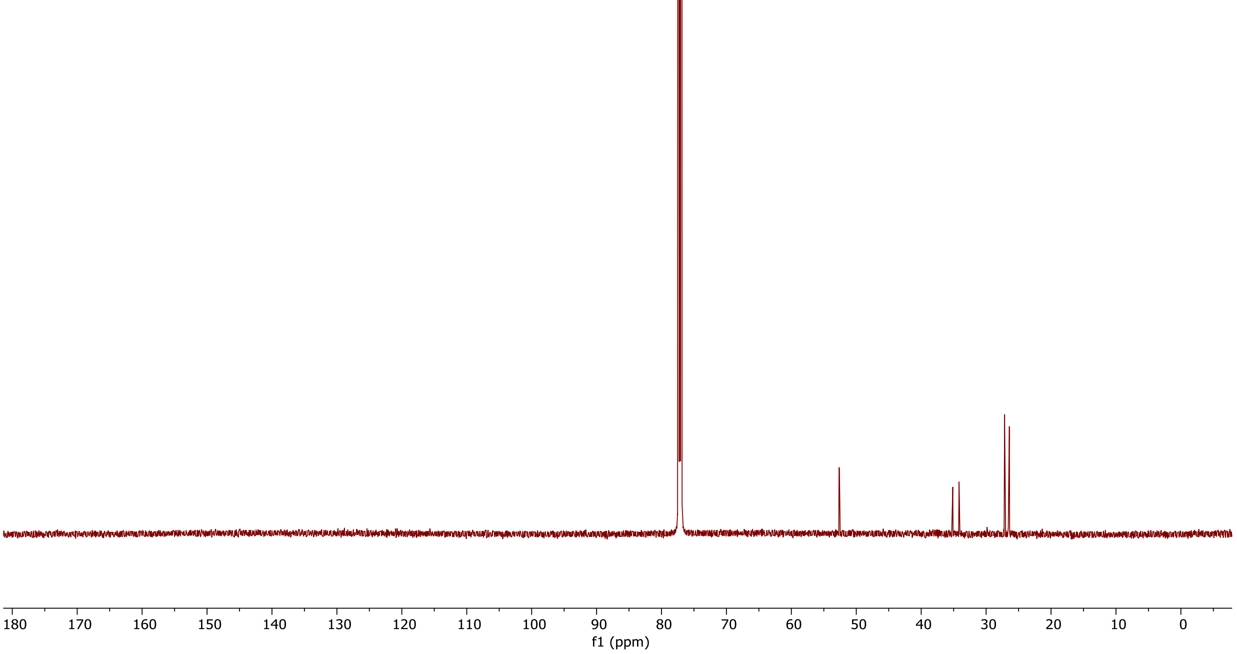

^{31}P NMR (162 MHz, $CDCl_3$) of **2**

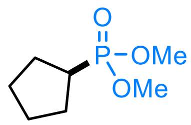

2

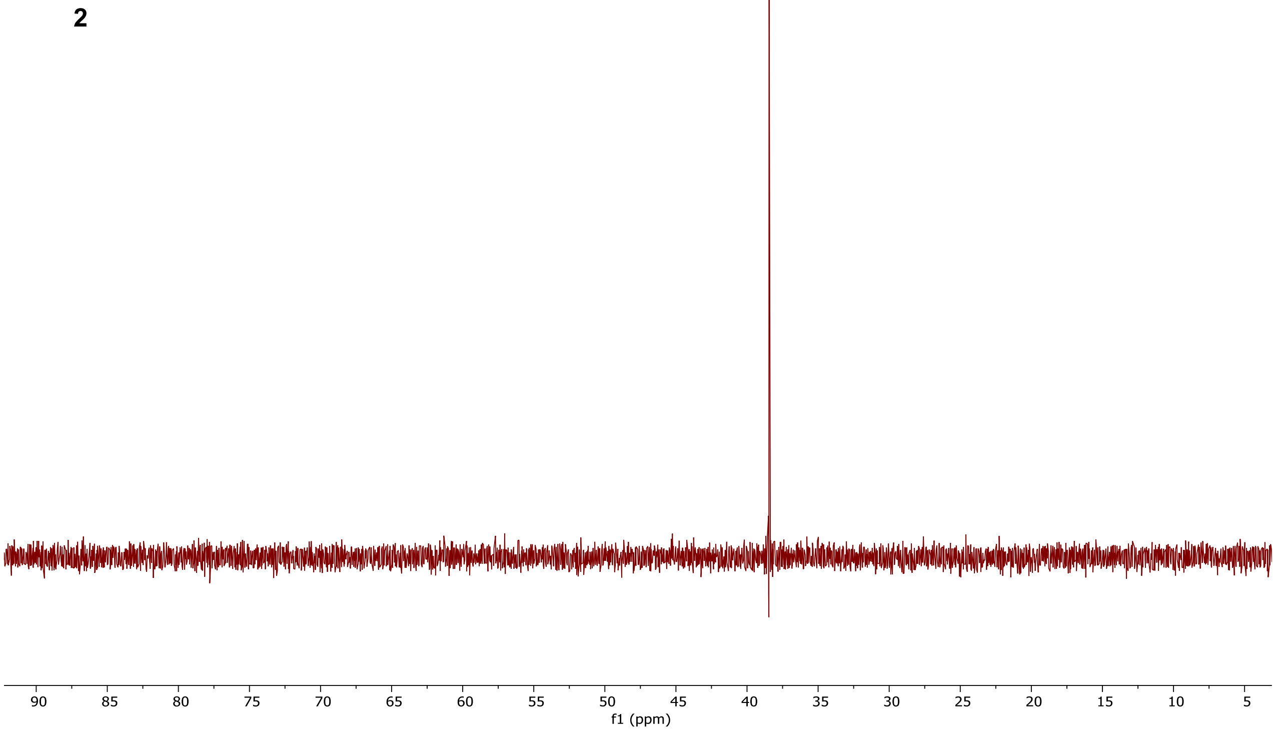

^{1}H NMR (600 MHz, $CDCl_3$) of **3** (*see procedure*)

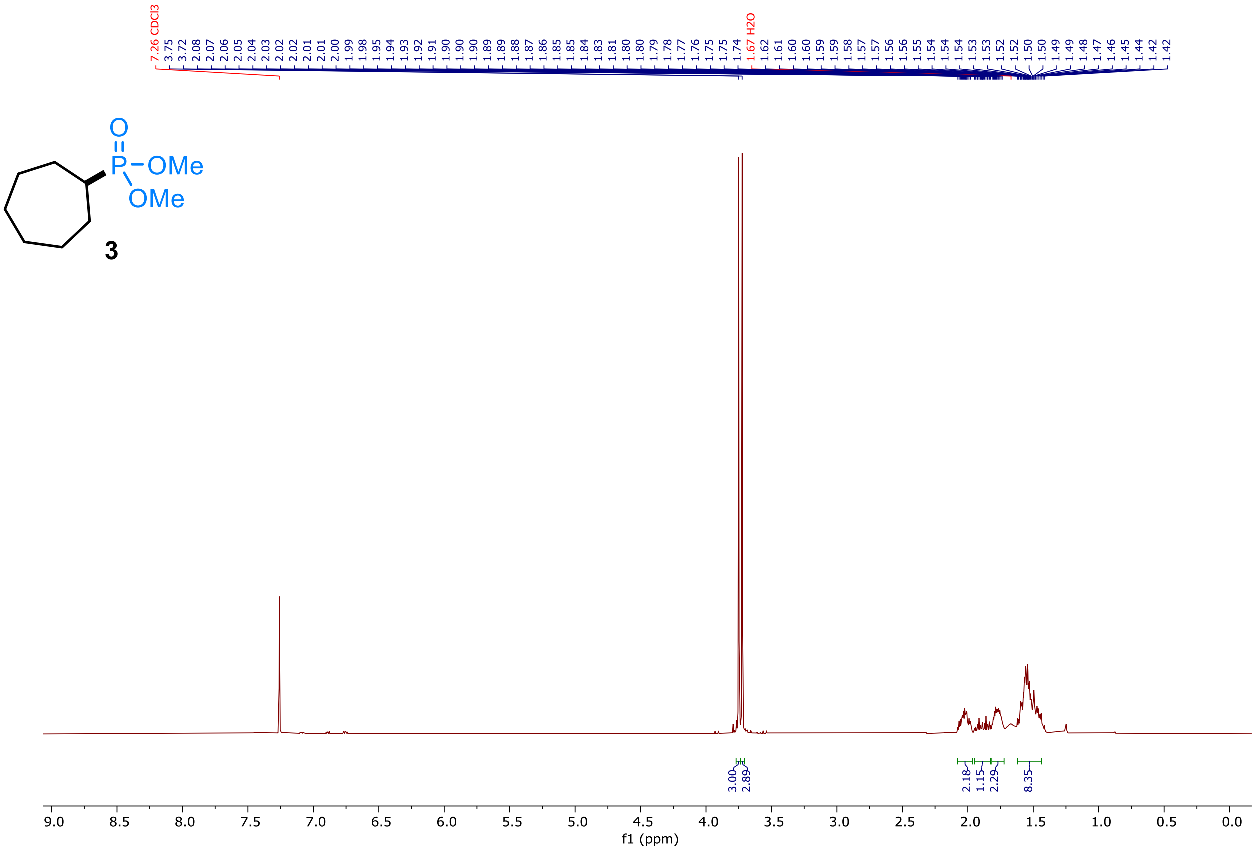

^{13}C NMR (101 MHz, $CDCl_3$) of **3**

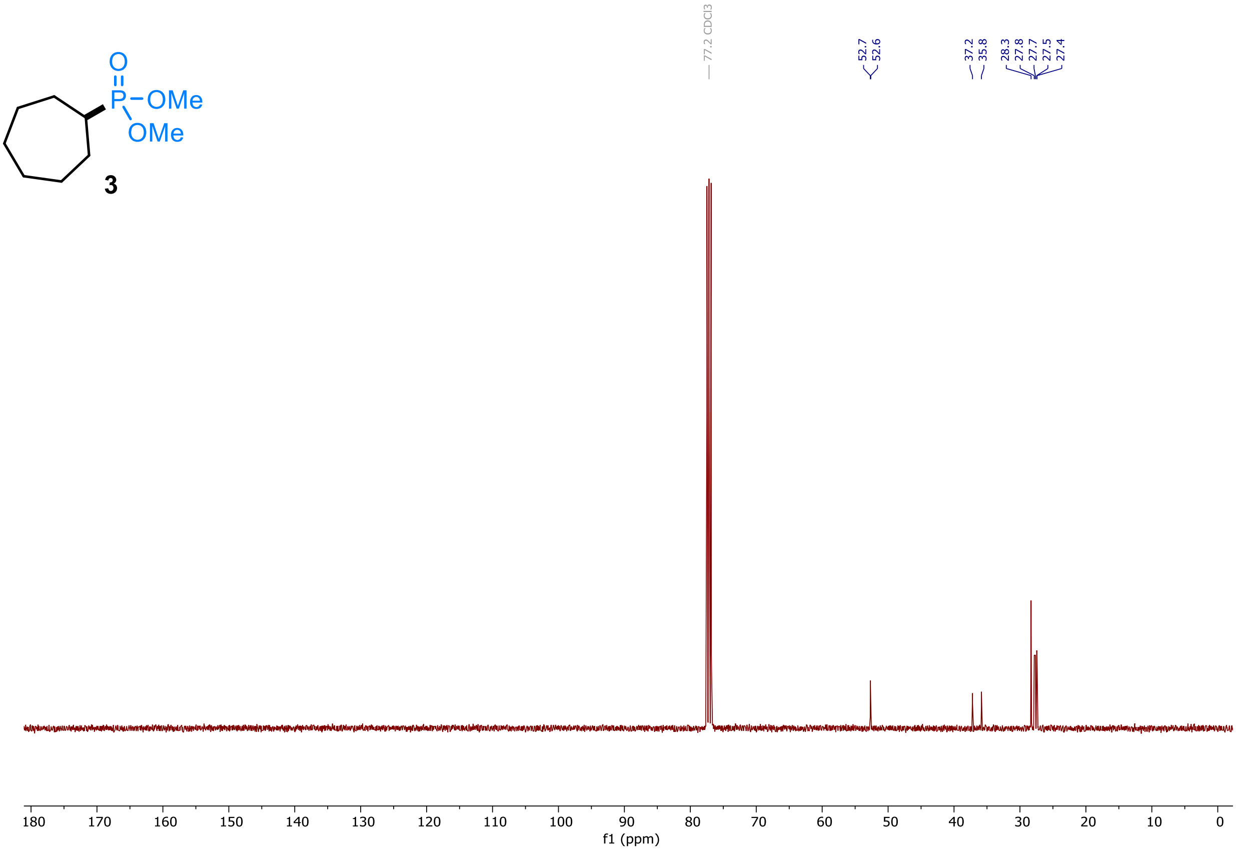

^{31}P NMR (162 MHz, $CDCl_3$) of **3**

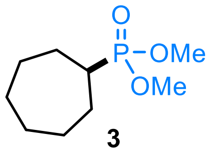

3

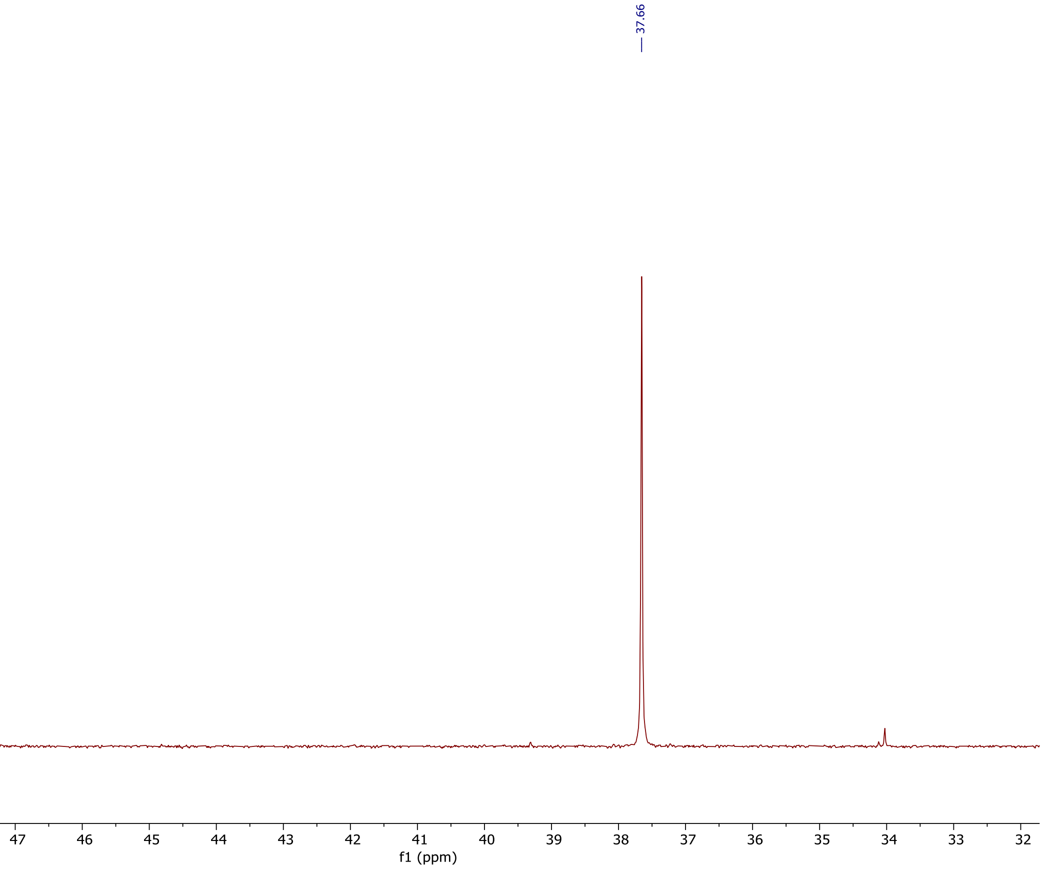

^{1}H NMR (600 MHz, CDCl3) of **4** (*see procedure*)

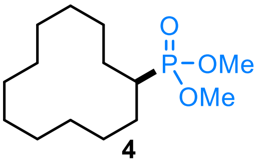

4

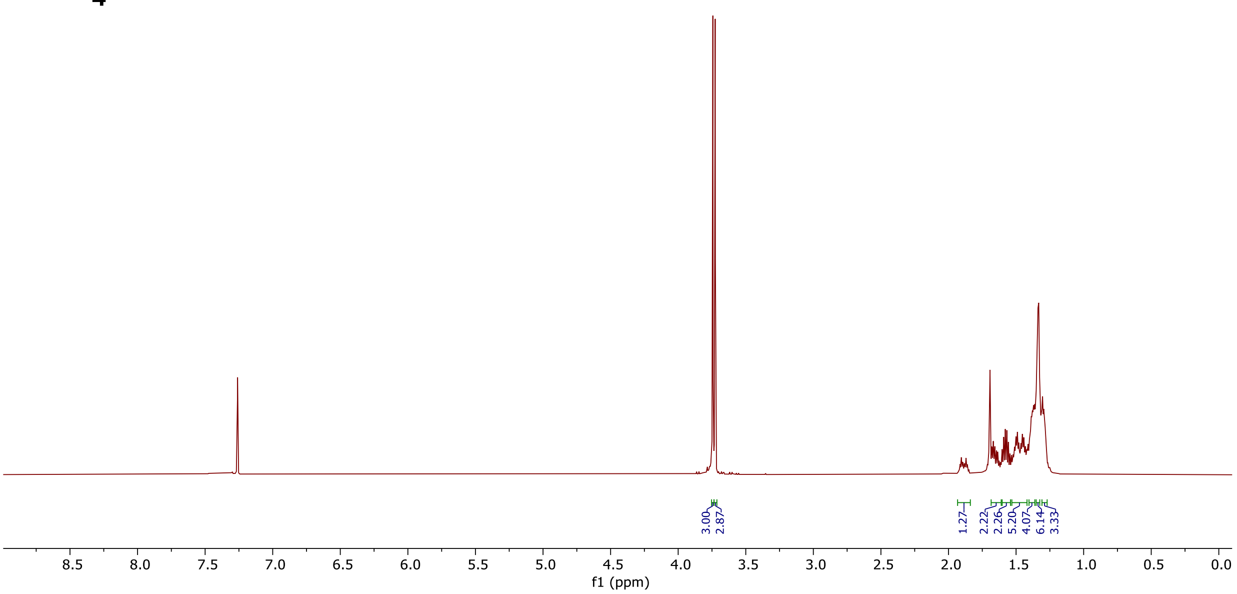

^{13}C NMR (151 MHz, CDCl3) of **4**

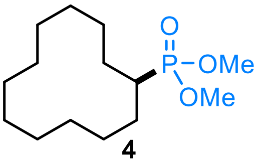

4

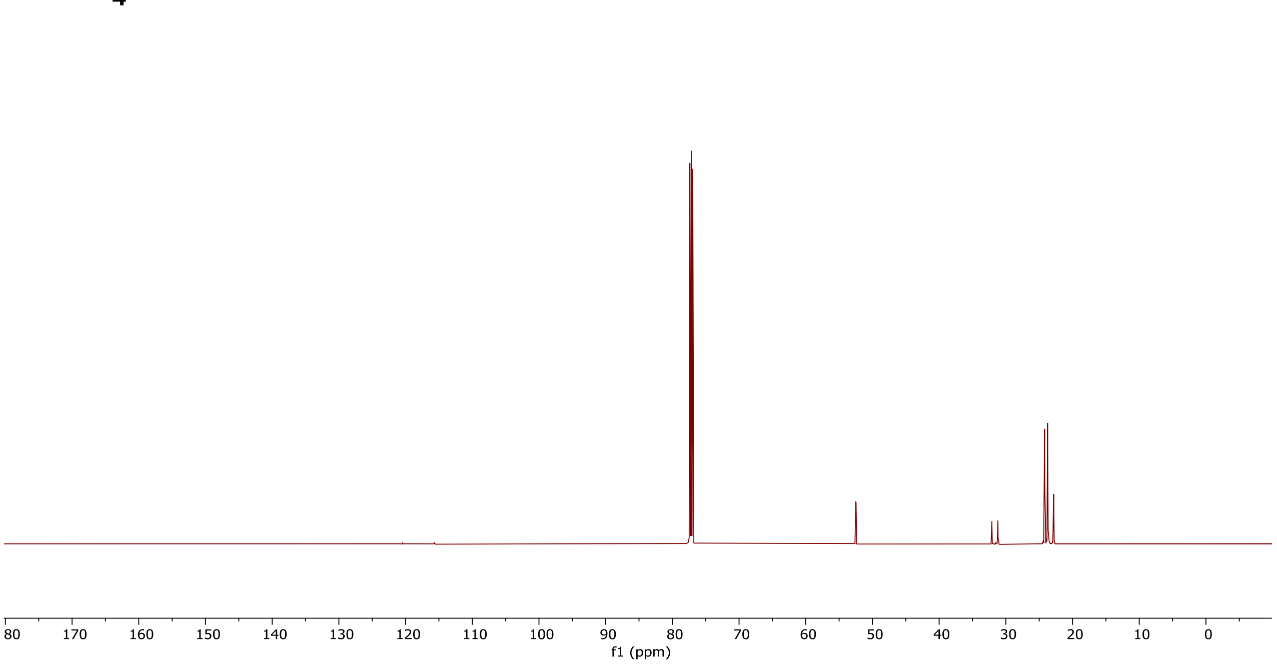

^{31}P NMR (162 MHz, $CDCl_3$ of **4**)

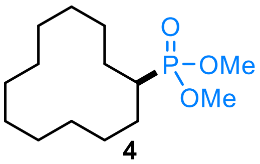

4

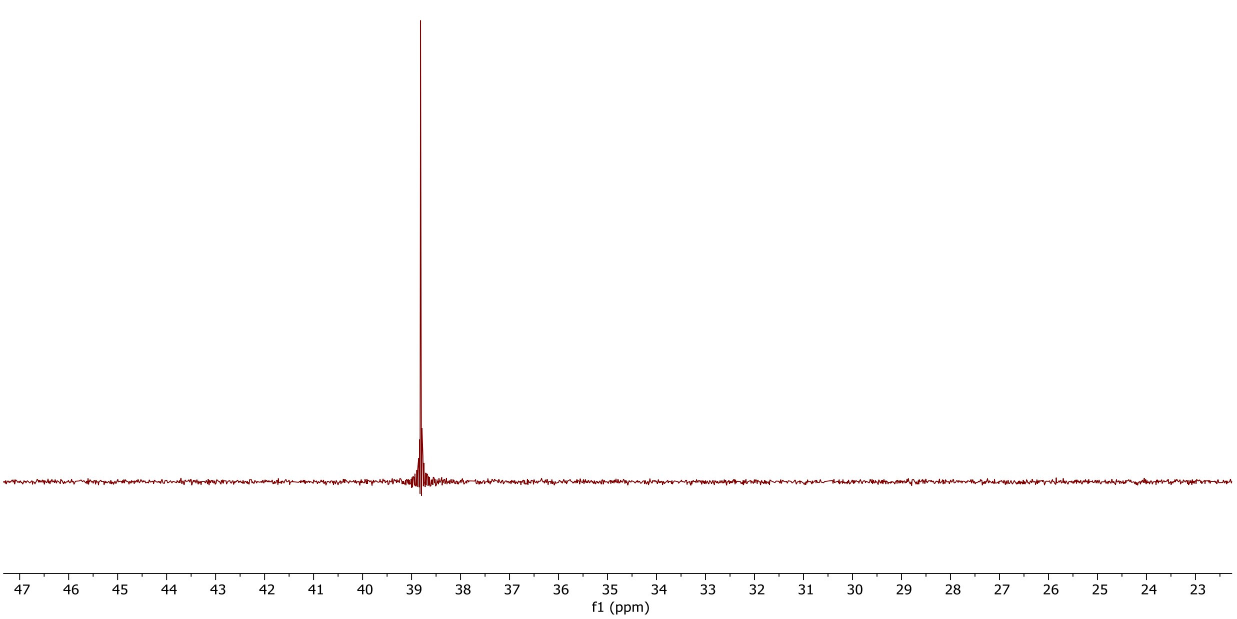

^{31}P NMR (162 MHz, $CDCl_3$) of crude **5-int** (*see procedure*)

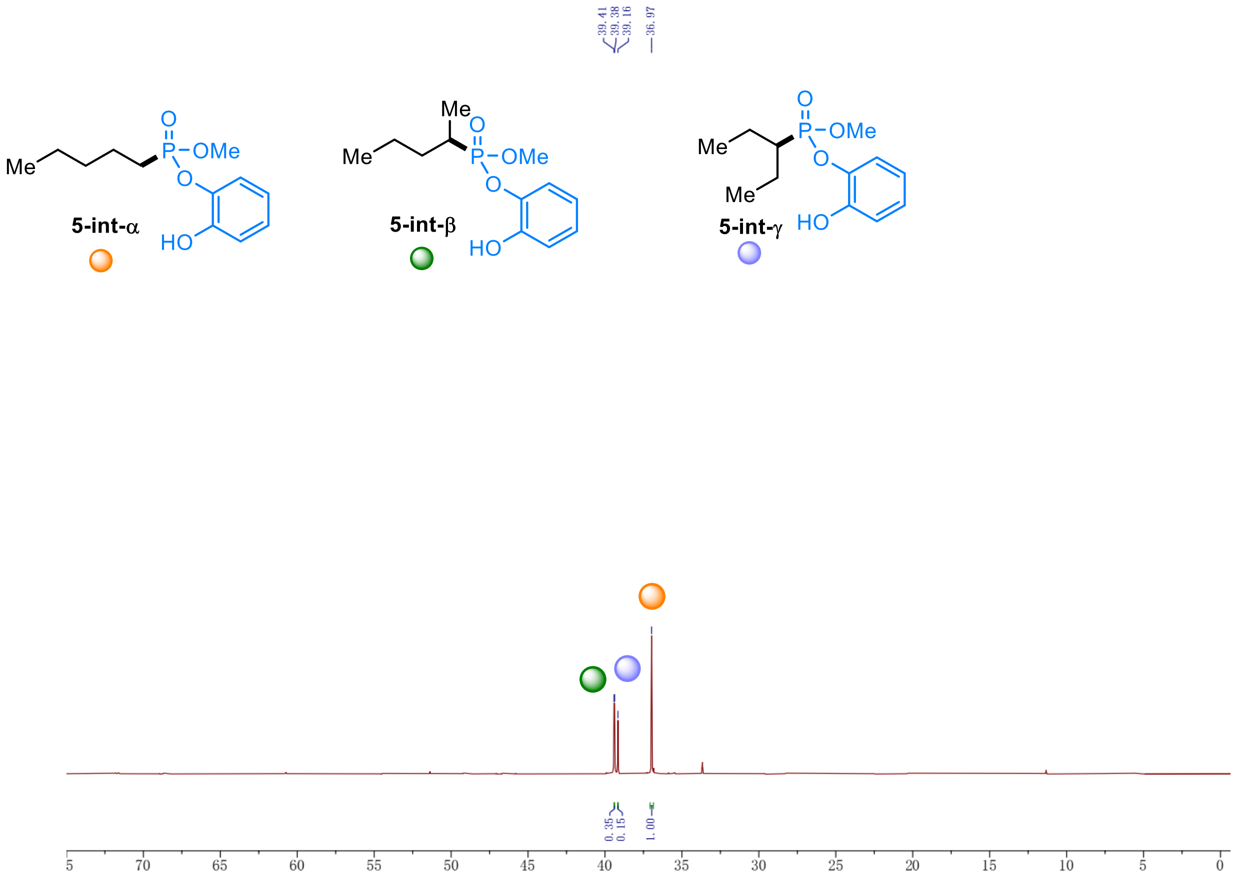

^{1}H NMR (400 MHz, $CDCl_3$) of **5** (*see procedure*)

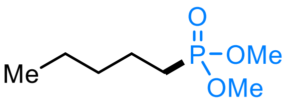

5

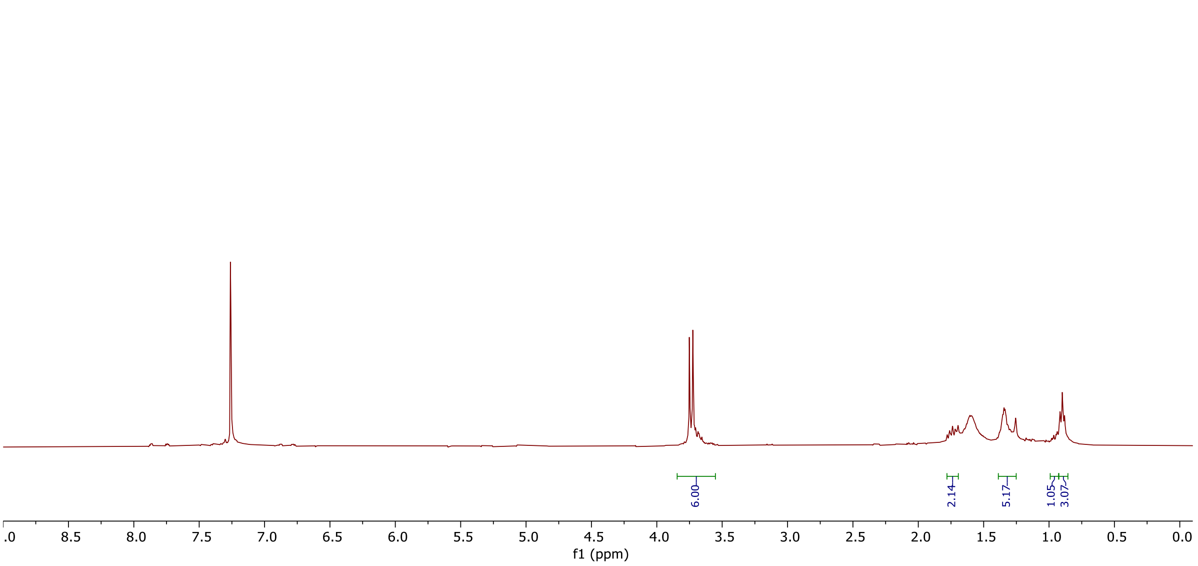

^{13}C NMR (151 MHz, $CDCl_3$) of **5**

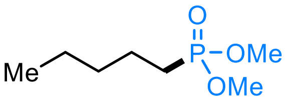

5

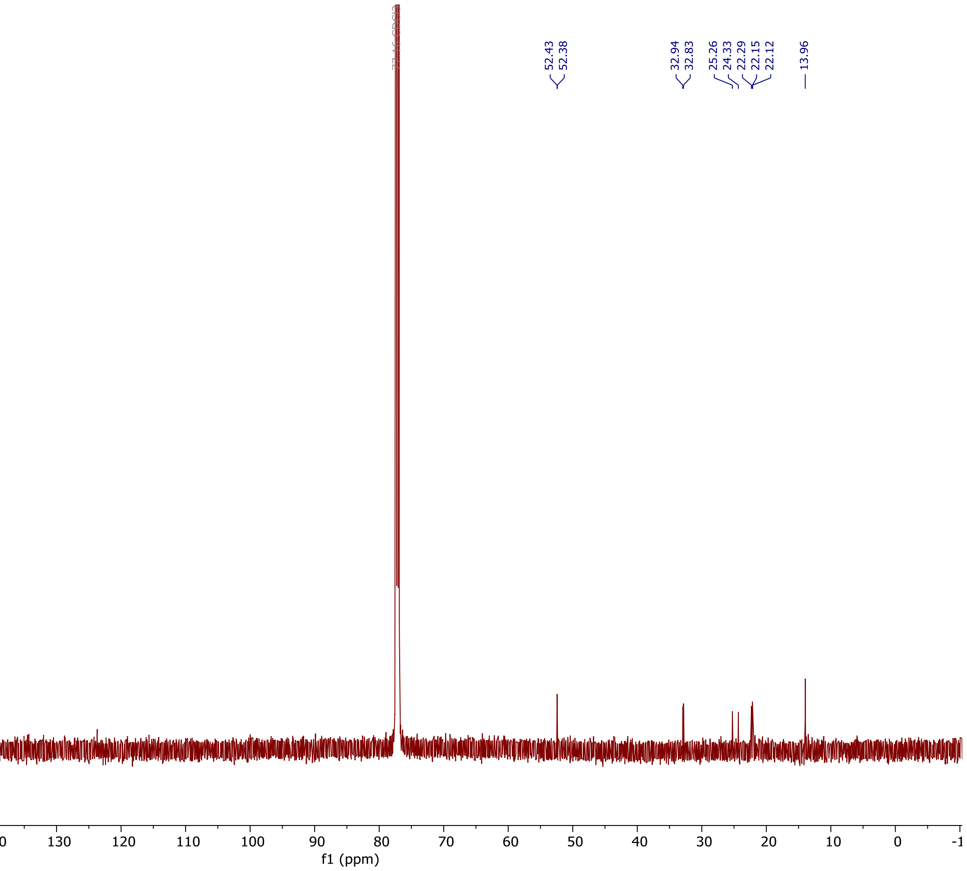

^{31}P NMR (162 MHz, $CDCl_3$) of **5**

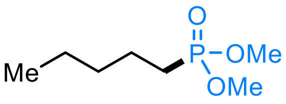

5

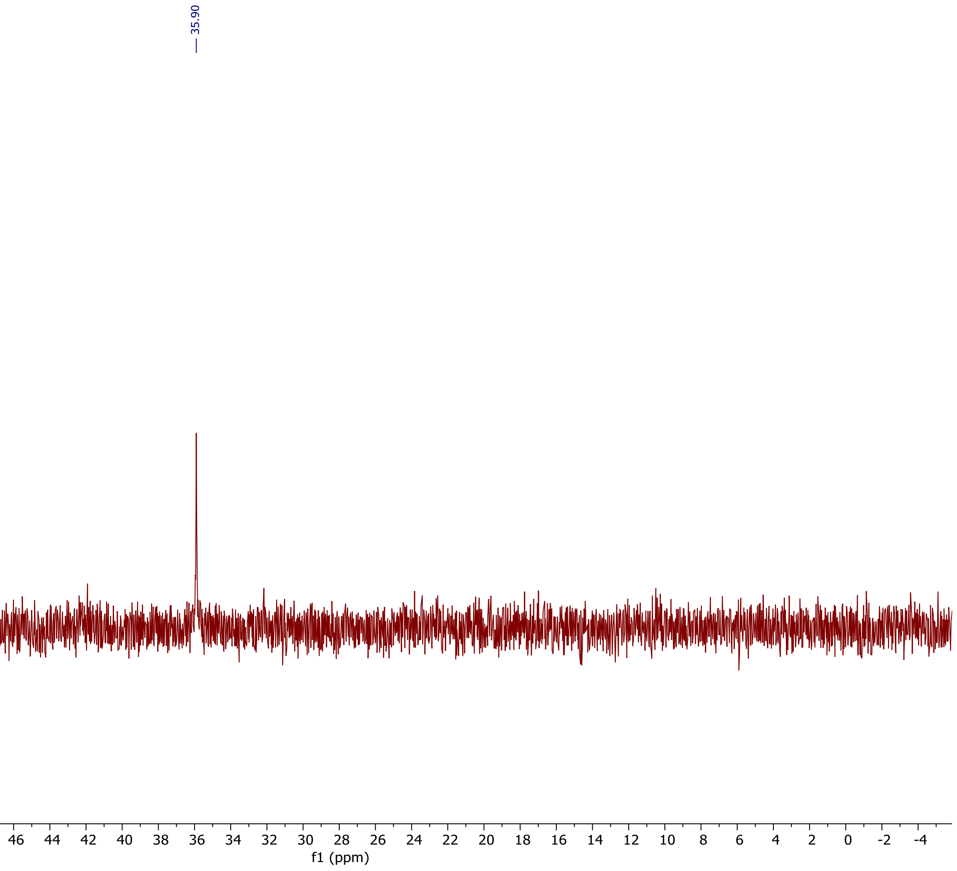

^{31}P NMR of crude reaction mixture of **6-int** (*see procedure*)

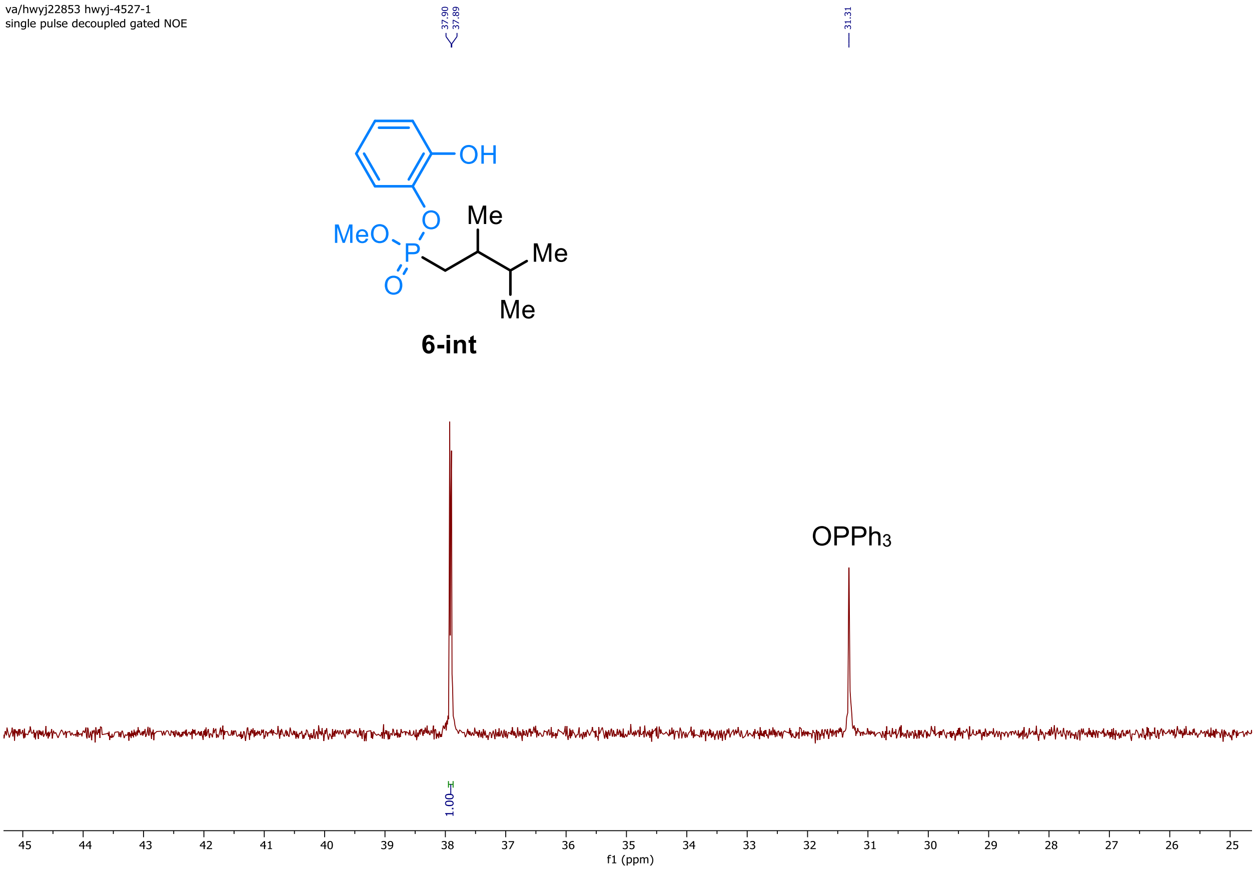

^{1}H NMR (400 MHz, $CDCl_3$) of **6** (*see procedure*)

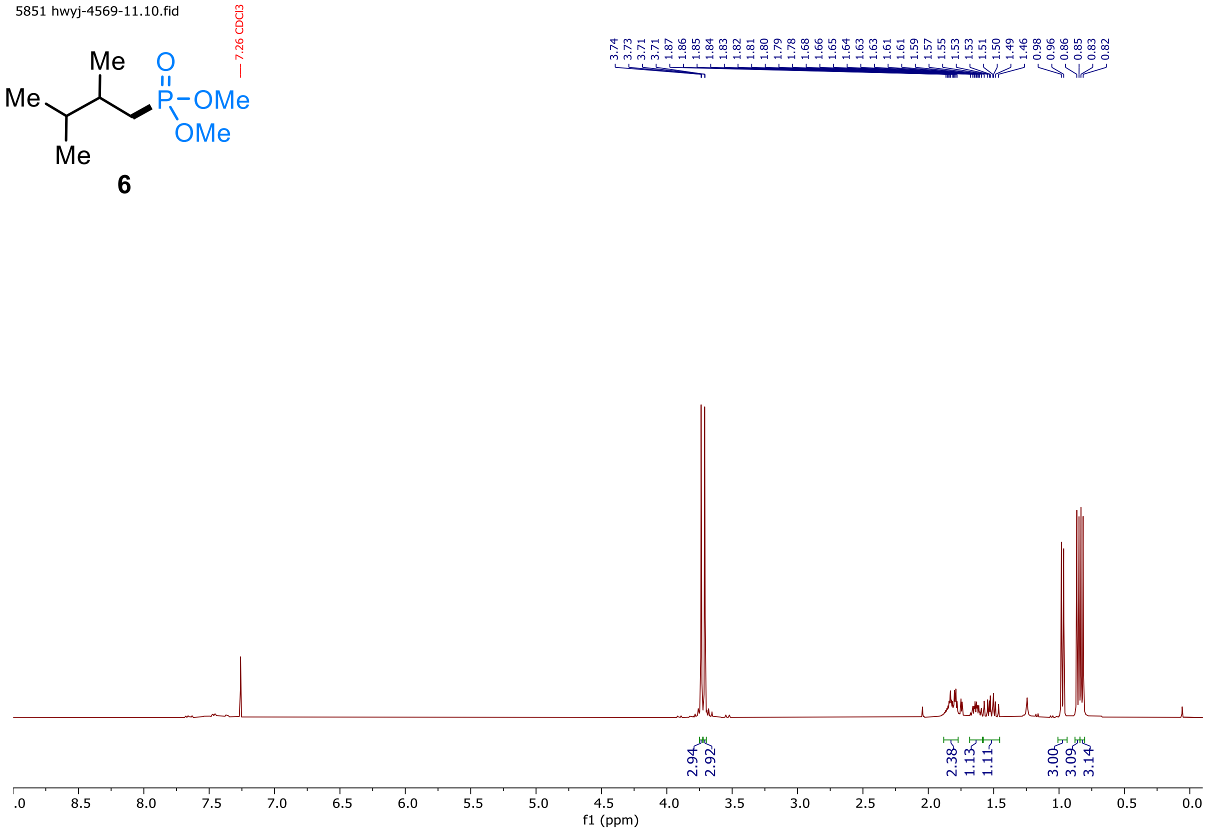

^{13}C NMR (101 MHz, CDCl$_3$) of **6**

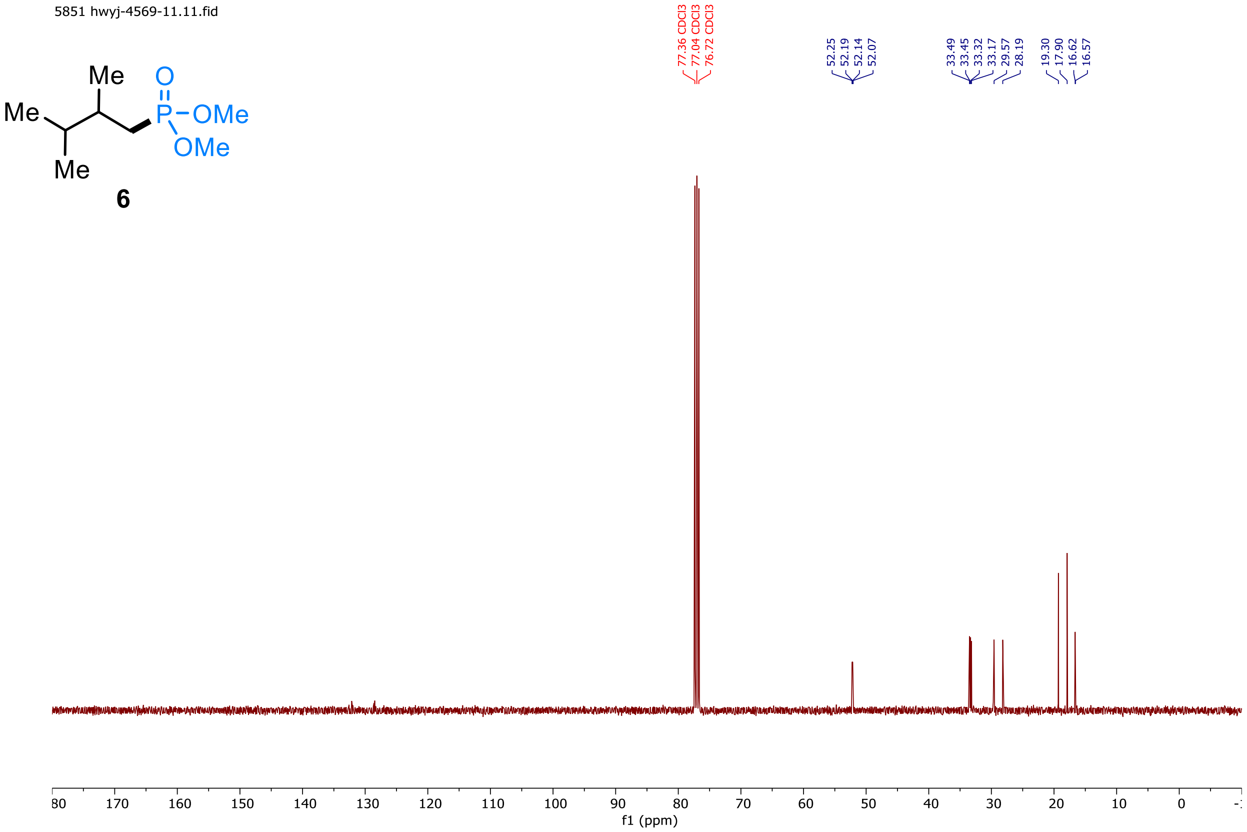

6

^{31}P NMR (162 MHz, CDCl$_3$) of **6**

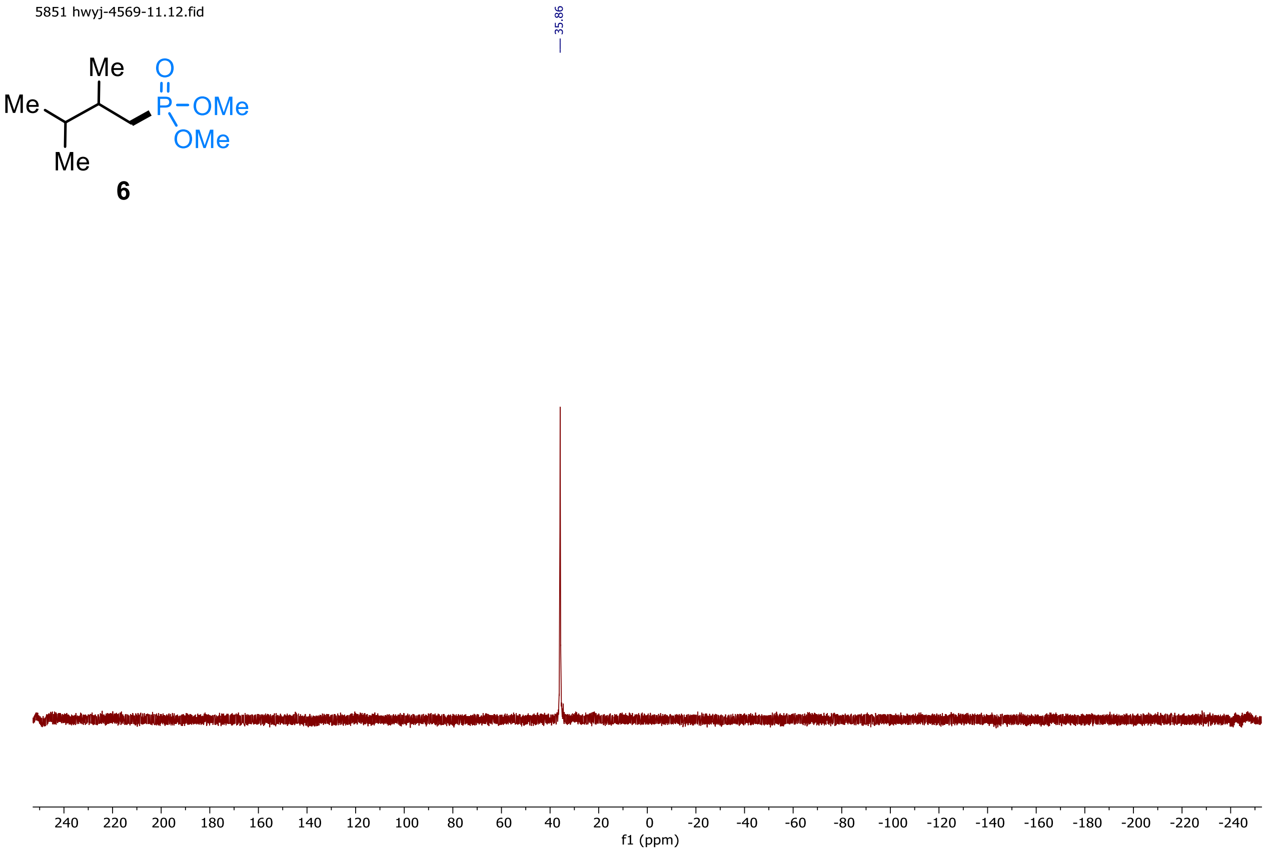

6

^{31}P NMR of crude reaction mixture of **7-int** (*see procedure*)

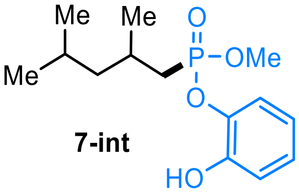

7-int

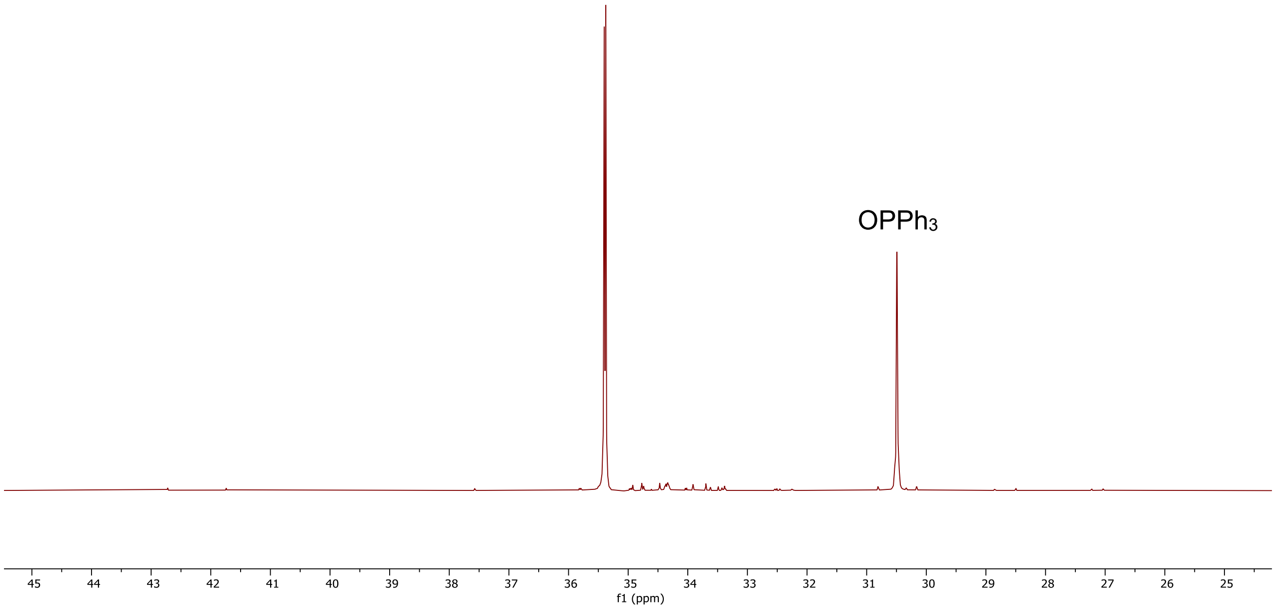

^{1}H NMR (400 MHz, Acetone-d_6) of **7** (*see procedure*)

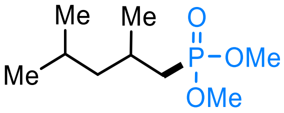

7

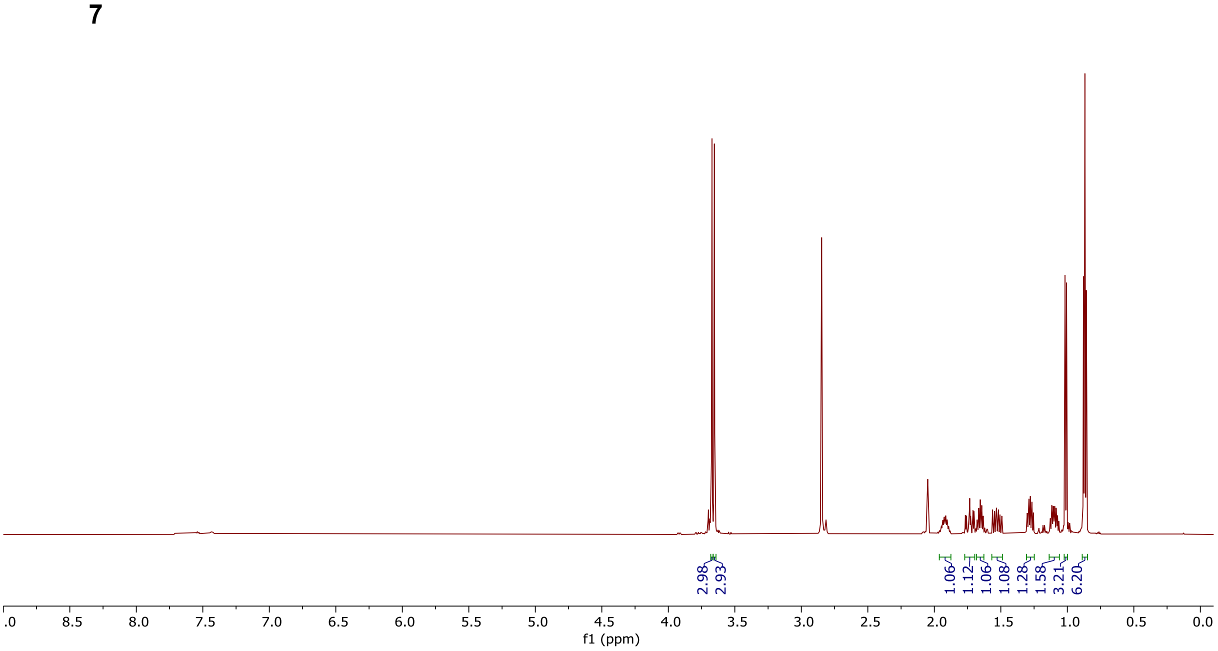

^{13}C NMR (151 MHz, Acetone-d_6) of **7**

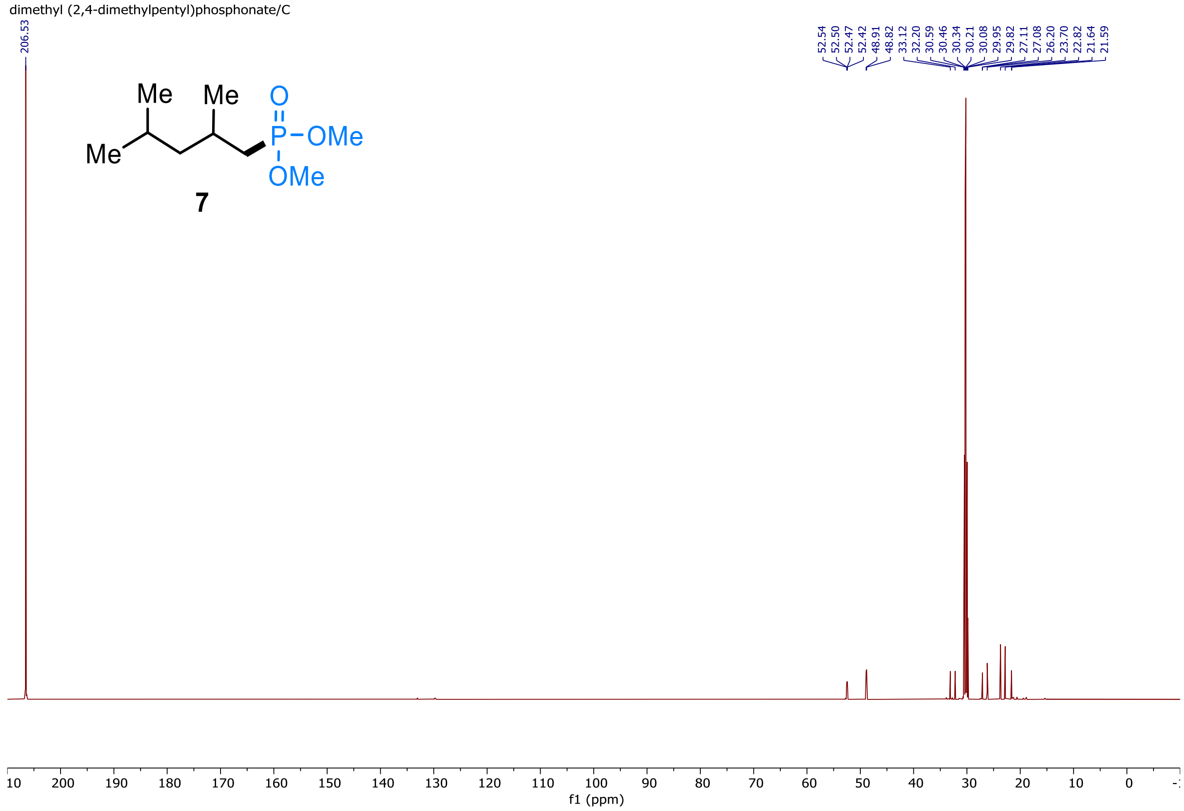

^{31}P NMR (162 MHz, Acetone-d_6) of **7**

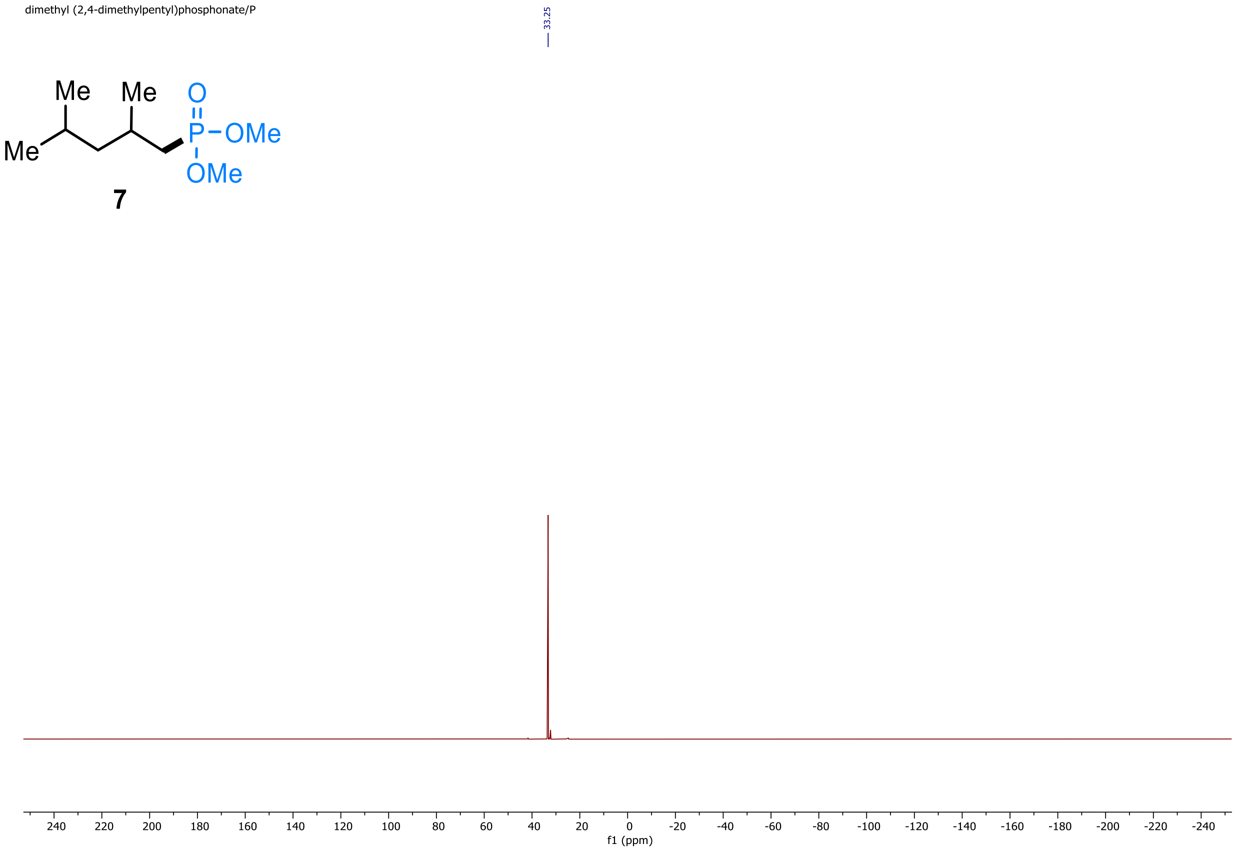

^{31}P NMR of crude reaction mixture of **8** (*see procedure*)

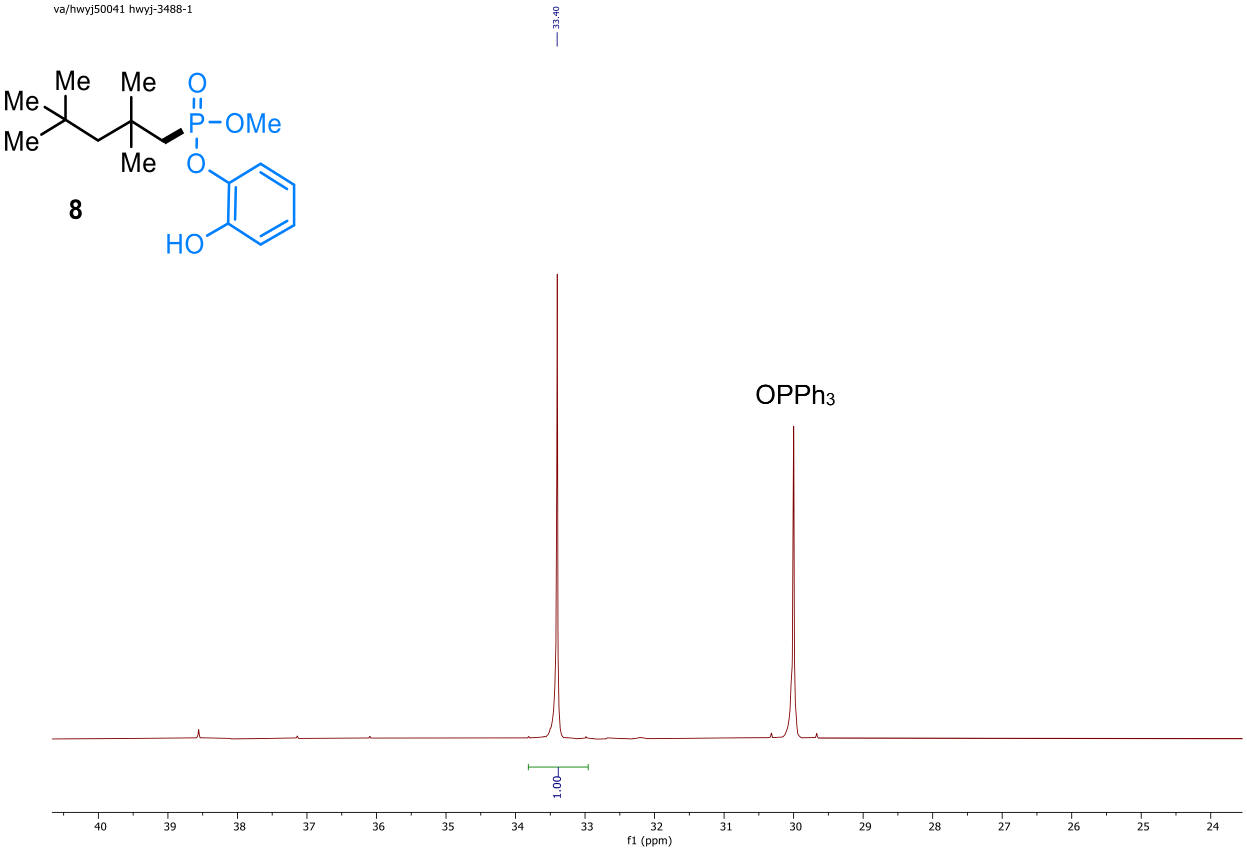

^{1}H NMR (400 MHz, $CDCl_3$) of **8** (*see procedure*)

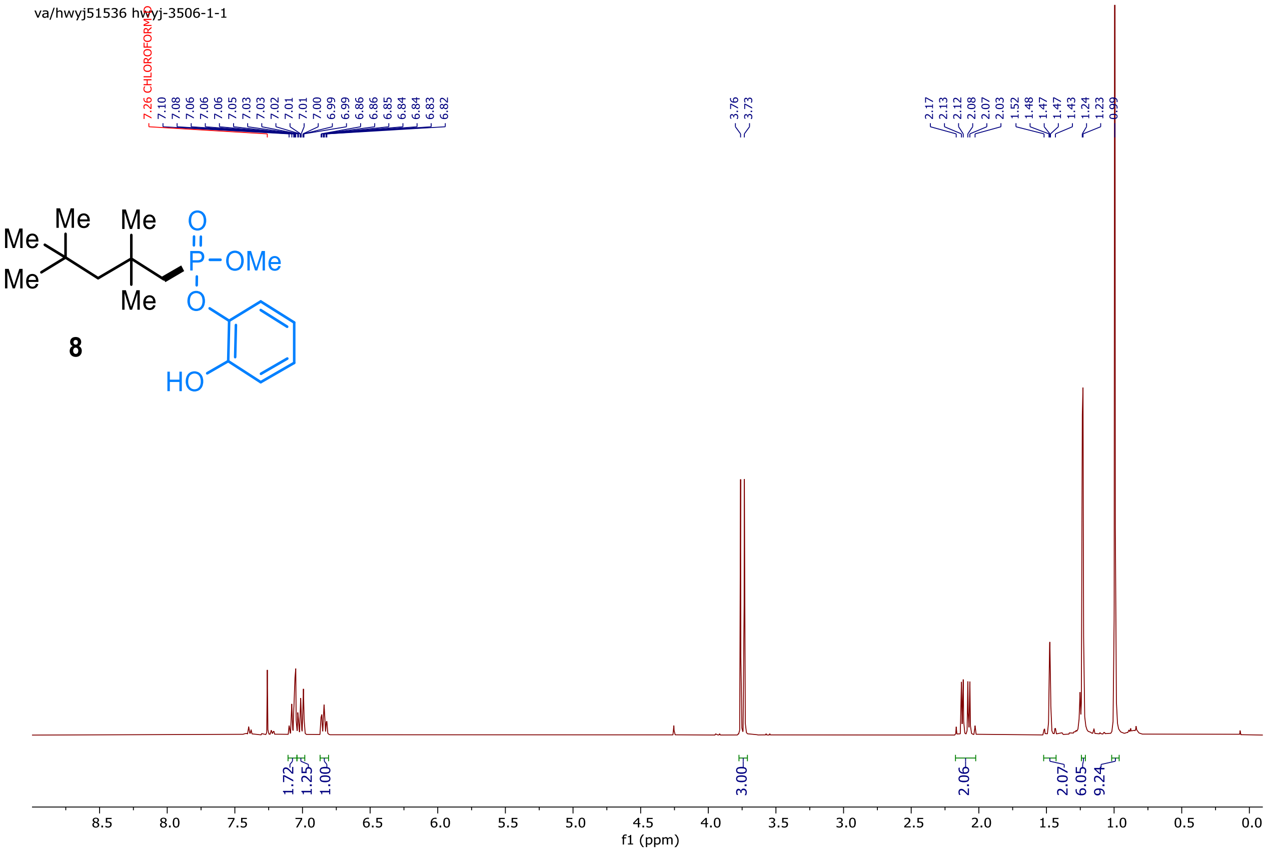

^{13}C NMR (101 MHz, $CDCl_3$) of **8**

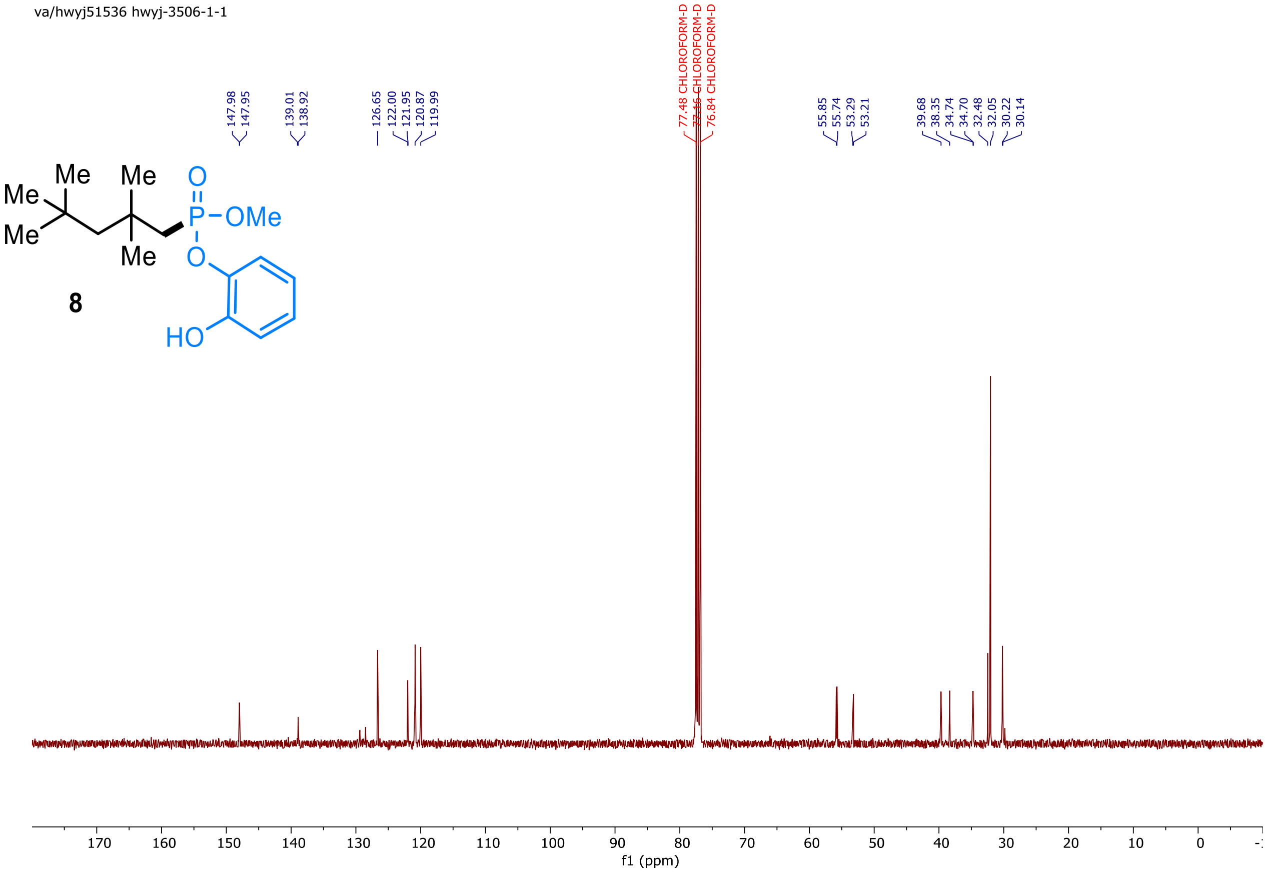

^{31}P NMR (162 MHz, $CDCl_3$) of **8**

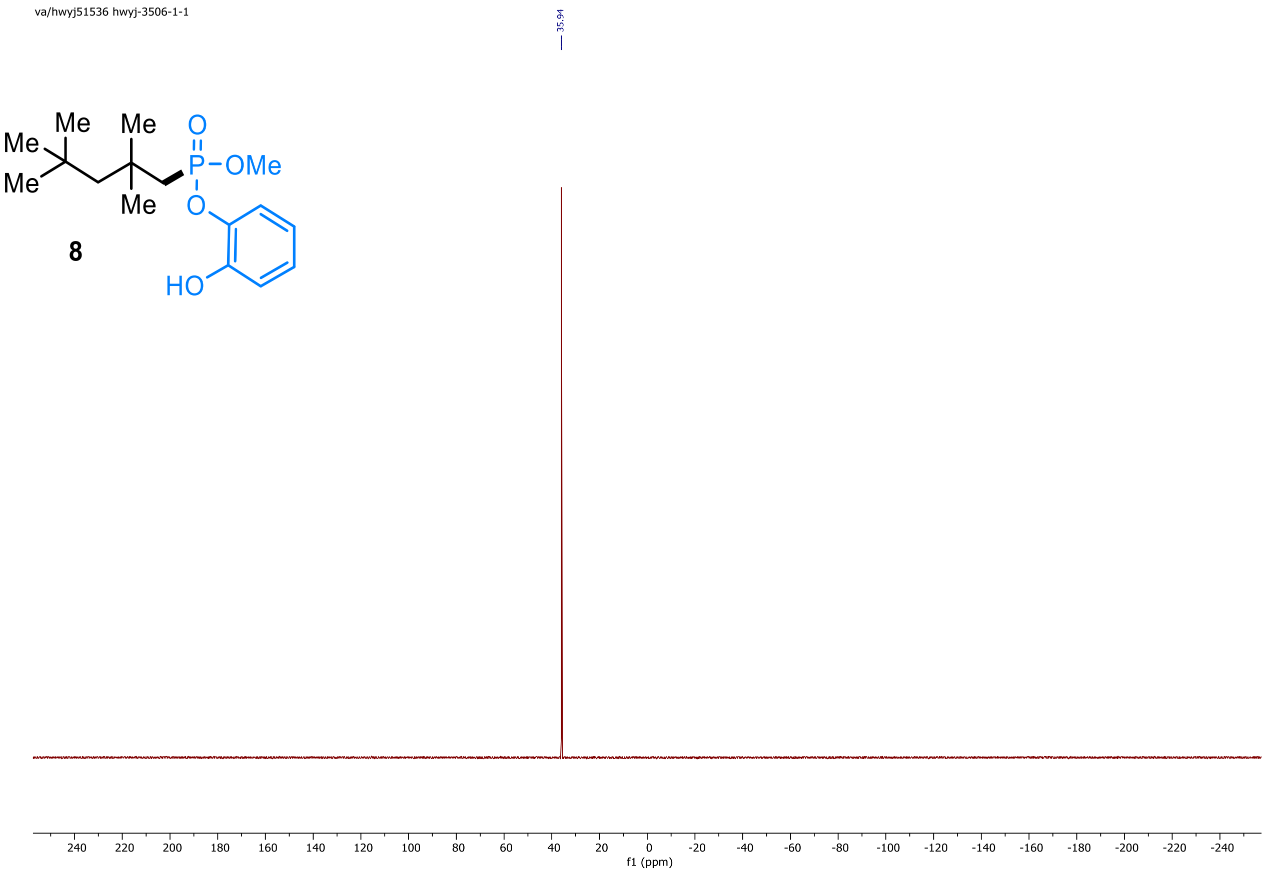

^{31}P NMR (162 MHz, CDCl$_3$) of crude **9-int** (*see procedure*)

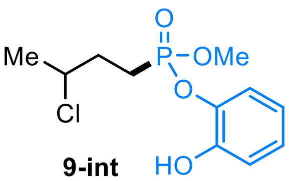

9-int

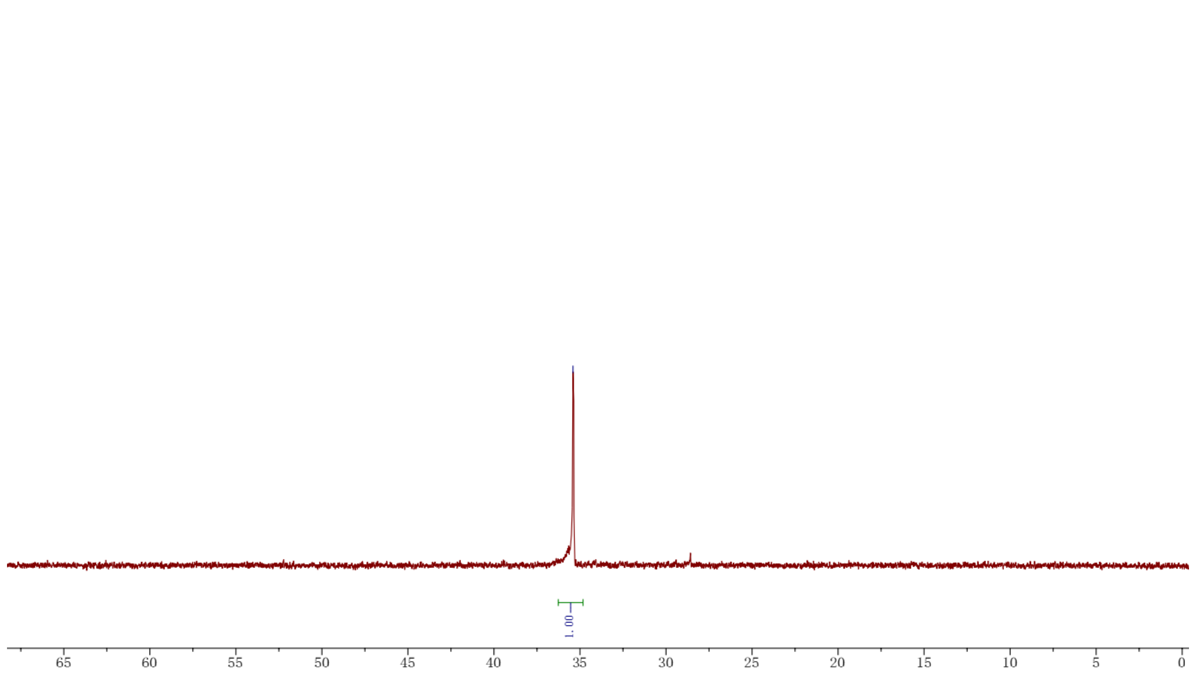

^{1}H NMR (400 MHz, CDCl$_3$) of **9** (*see procedure*)

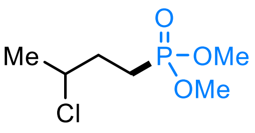

9

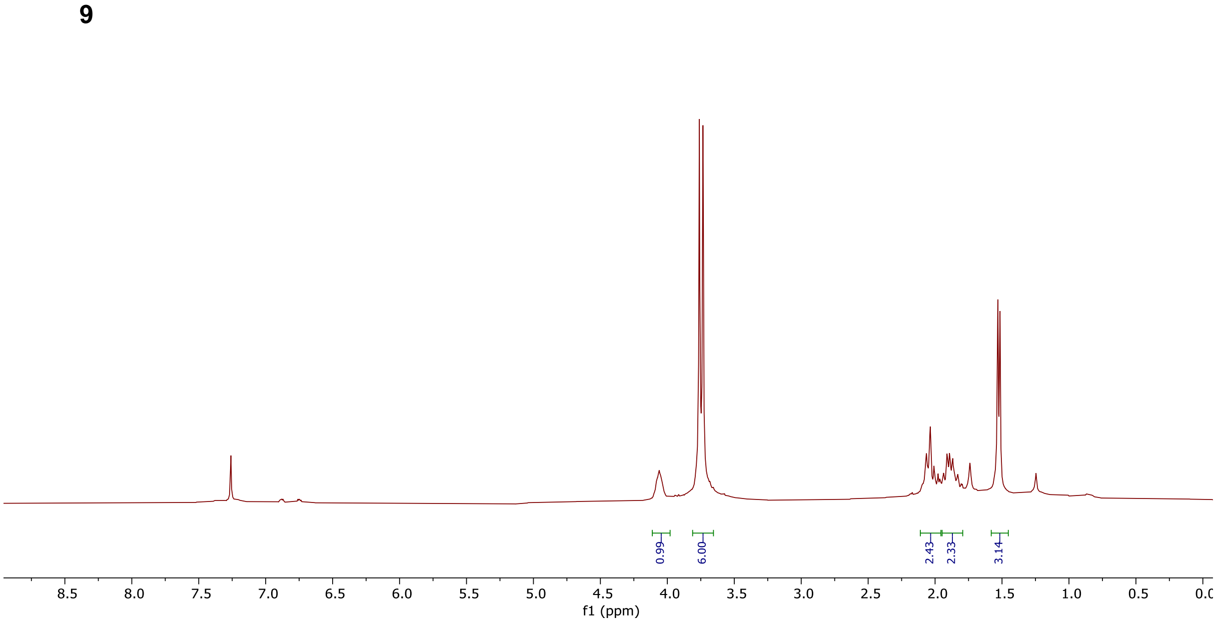

^{13}C NMR (101 MHz, $CDCl_3$) of **9**

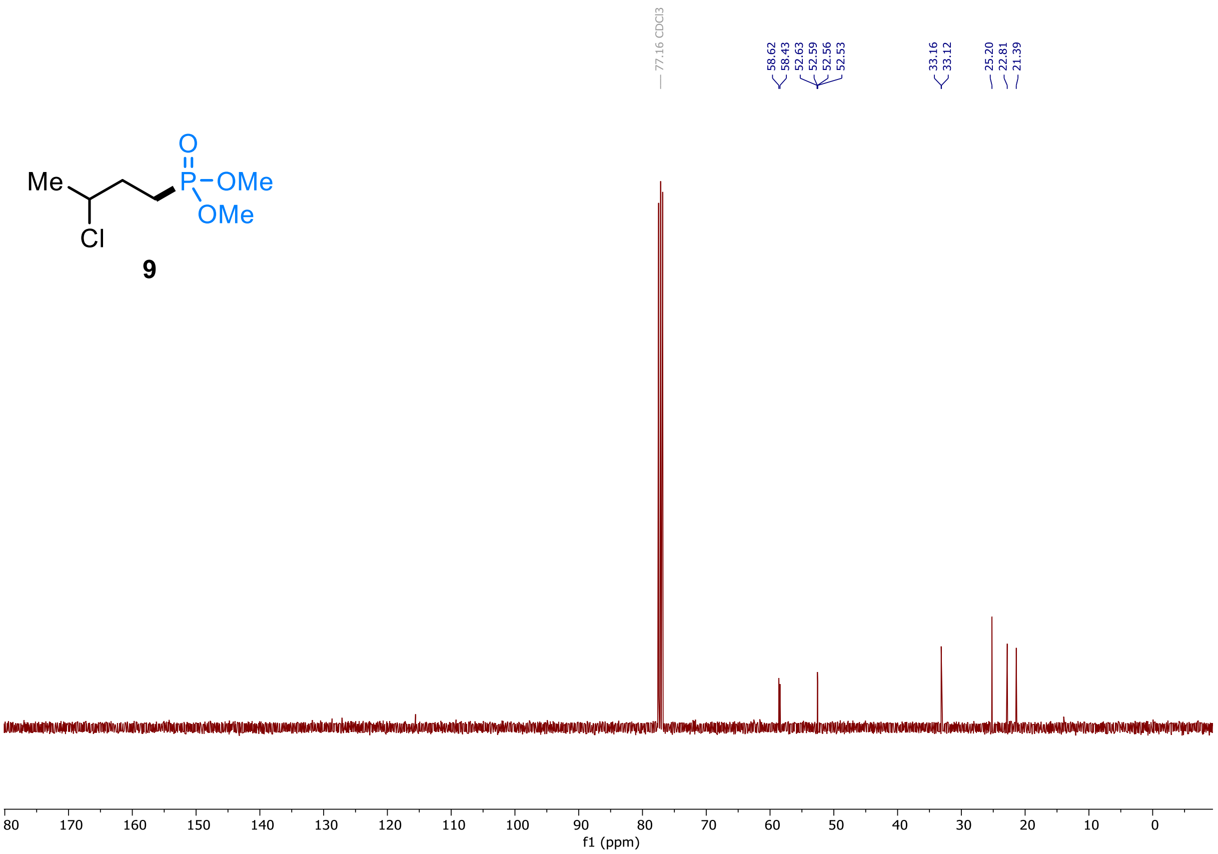

^{31}P NMR (162 MHz, $CDCl_3$) of **9**

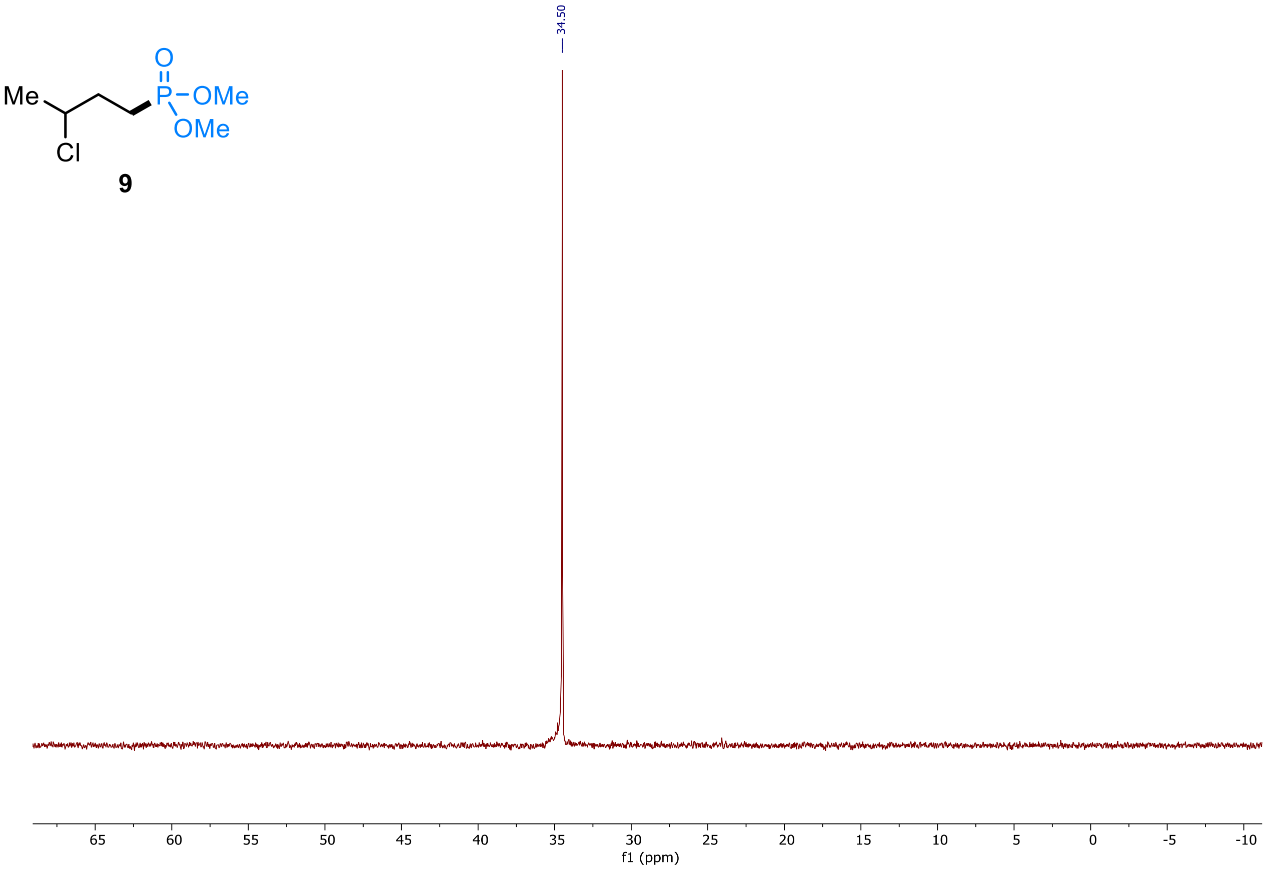

^{31}P NMR (162 MHz, CDCl$_3$) of crude **10-int** (*see procedure*)

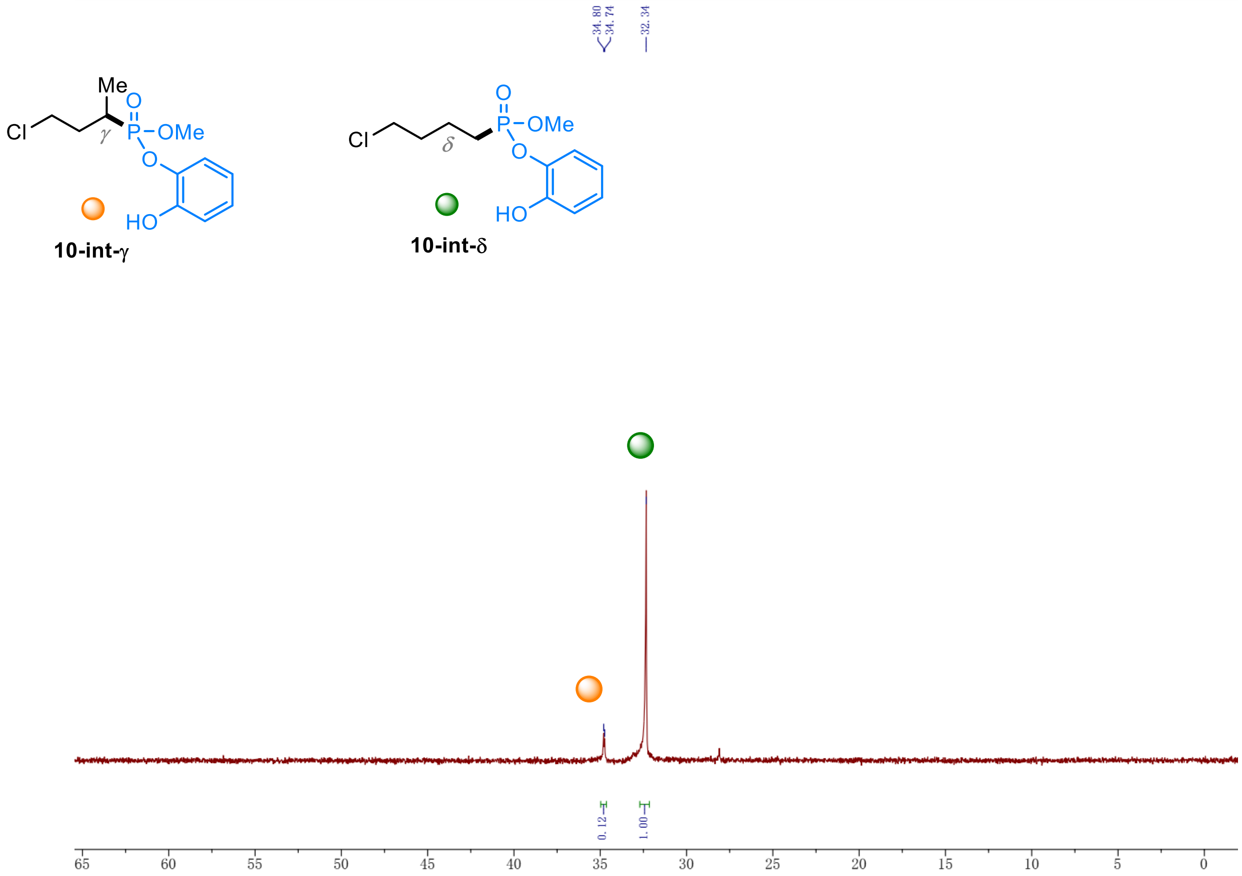

^{1}H NMR (400 MHz, CDCl$_3$) of **10** (*see procedure*)

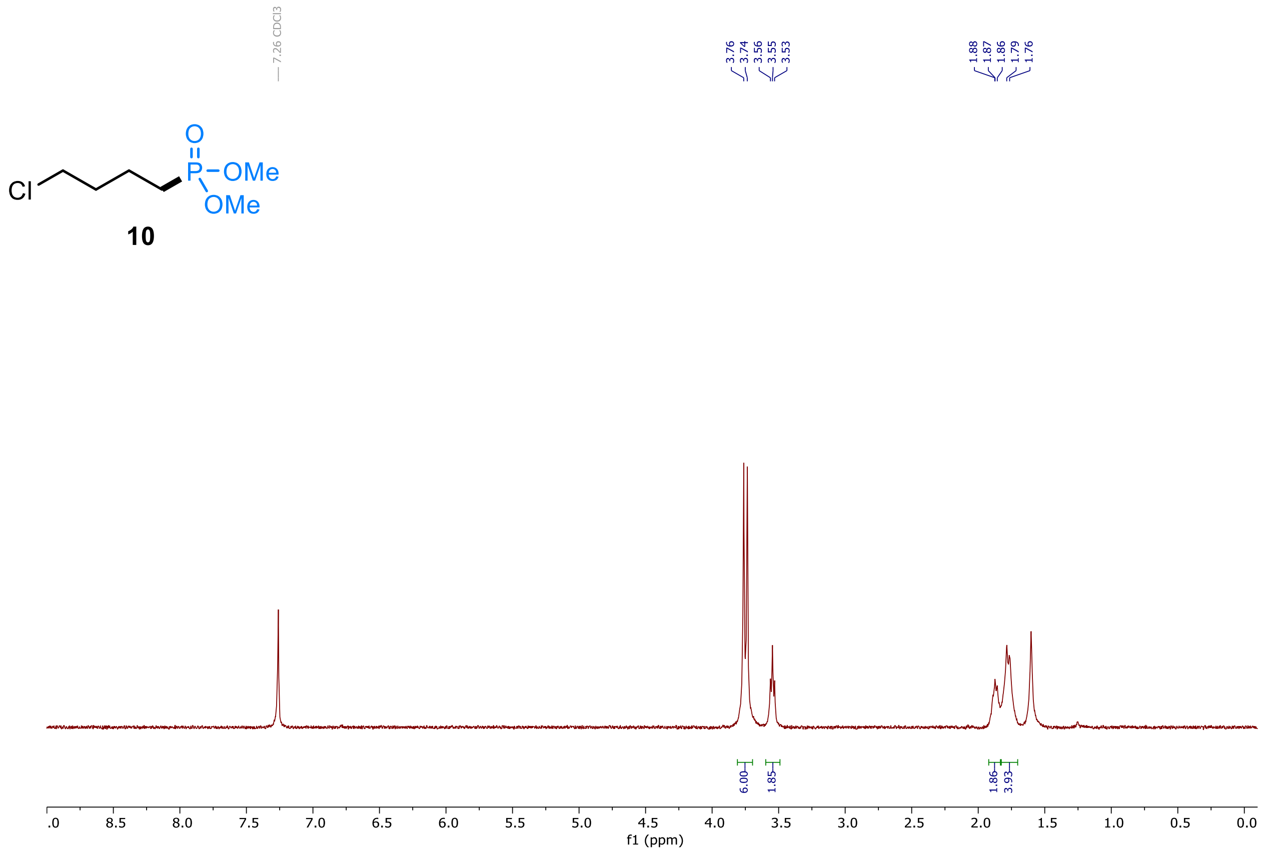

^{13}C NMR (151 MHz, $CDCl_3$) of **10**

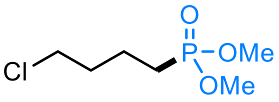

10

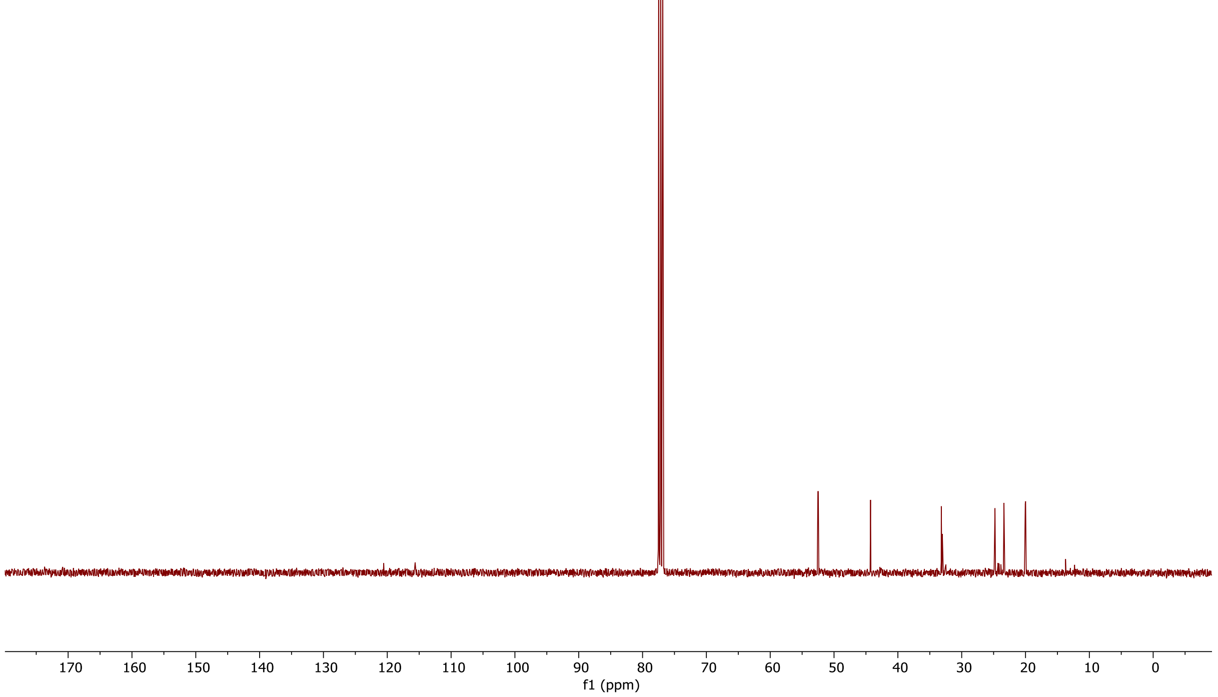

^{31}P NMR (162 MHz, $CDCl_3$) of **10**

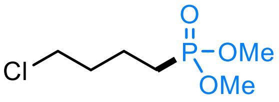

10

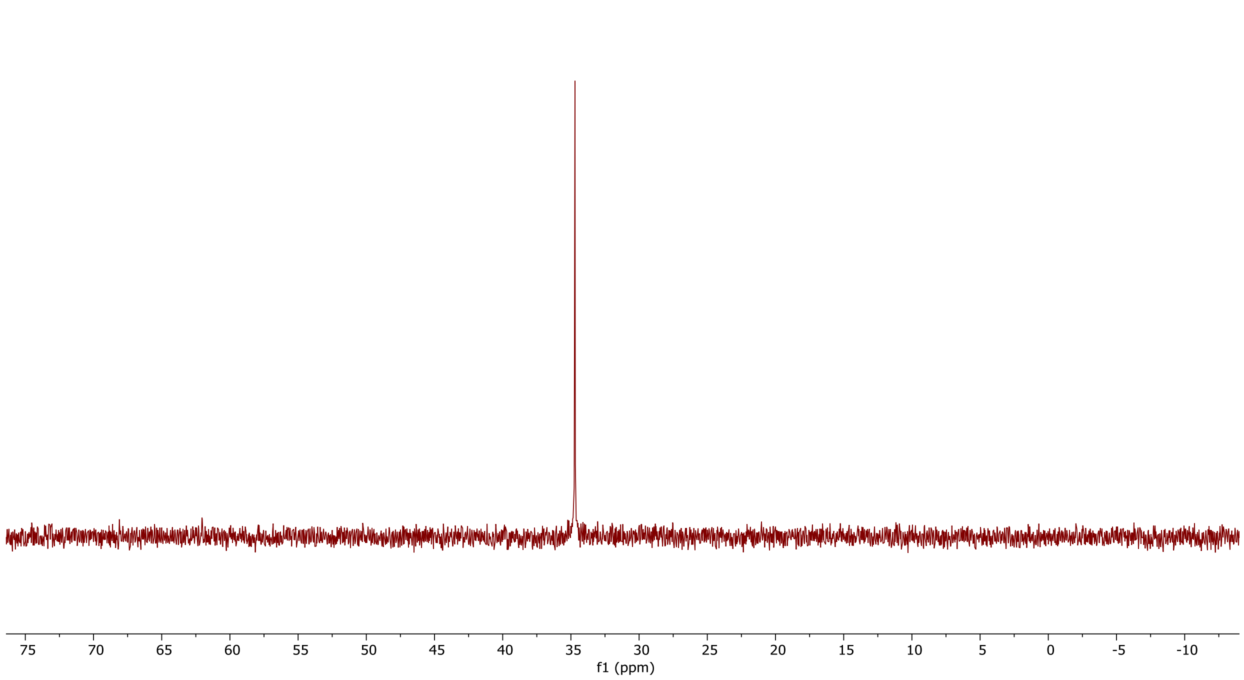

^{31}P NMR (162 MHz, $CDCl_3$) of crude **11** (*see procedure*)

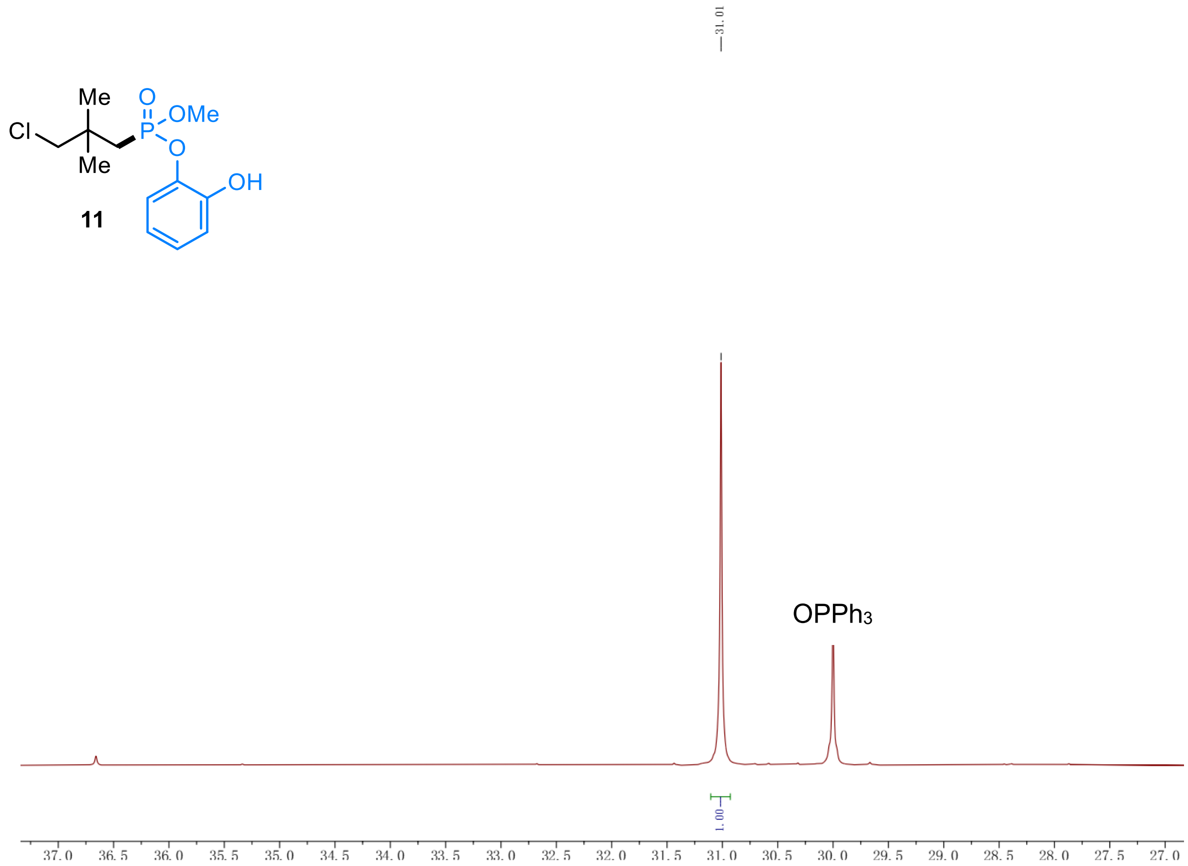

^{1}H NMR (400 MHz, $CDCl_3$) of **11** (*see procedure*)

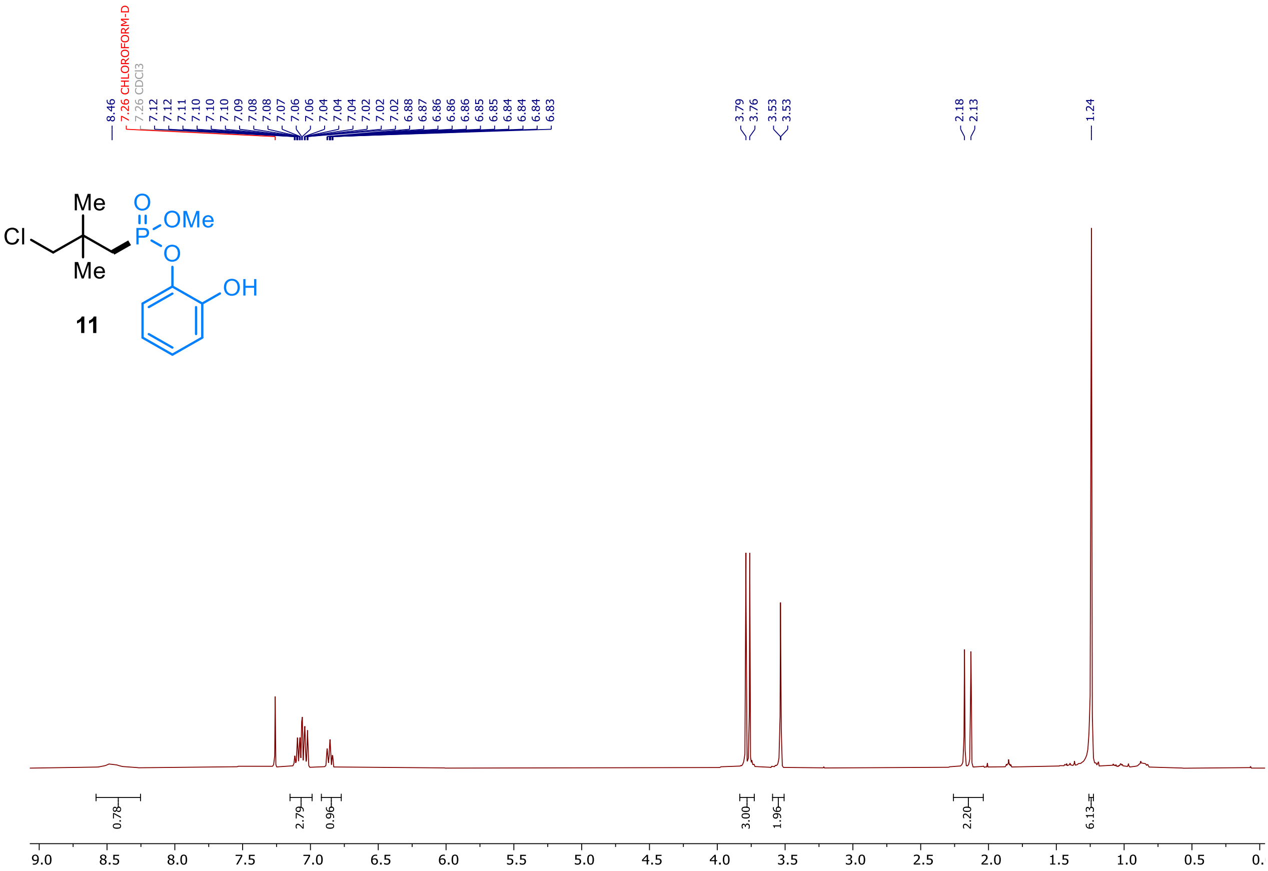

^{13}C NMR (101 MHz, $CDCl_3$) of **11**

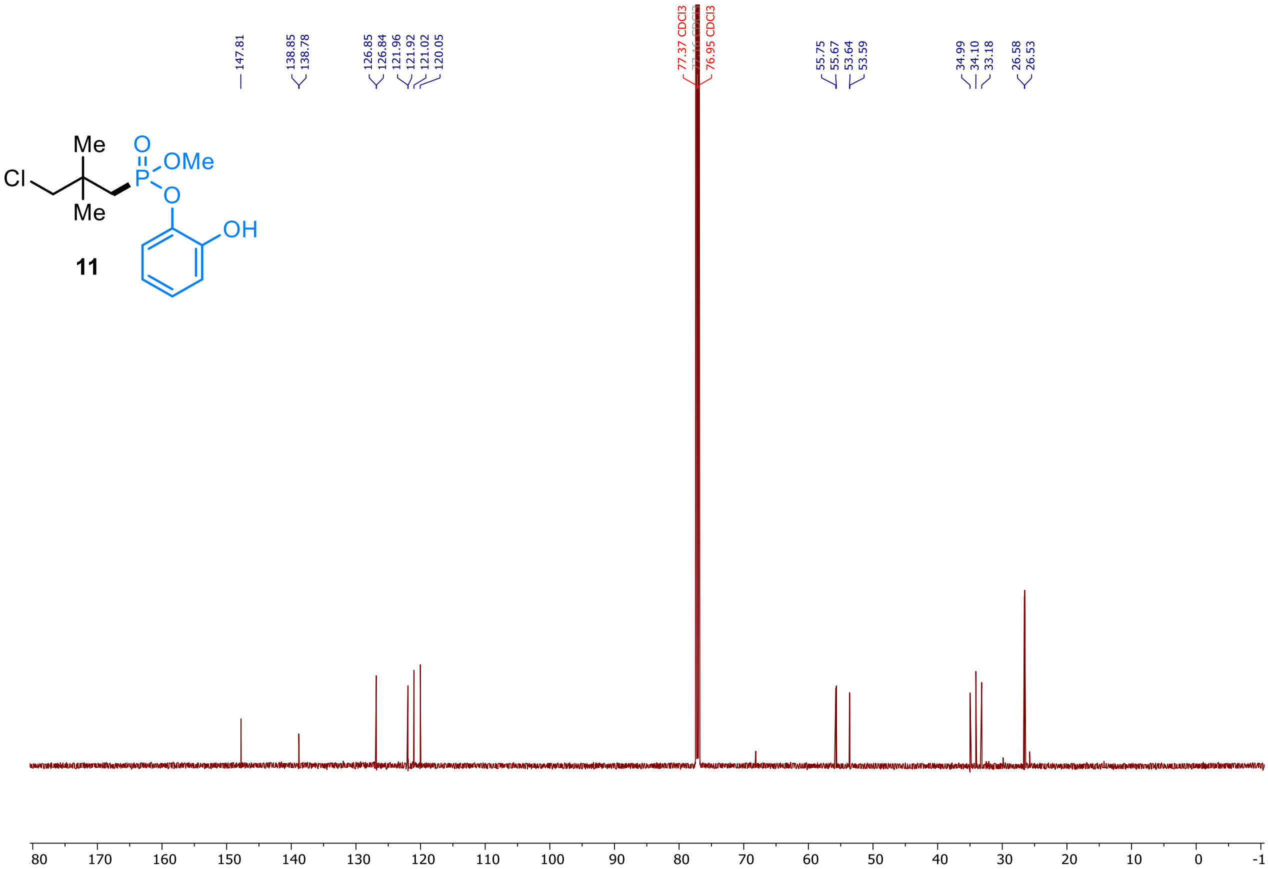

^{31}P NMR (400 MHz, $CDCl_3$) of **11**

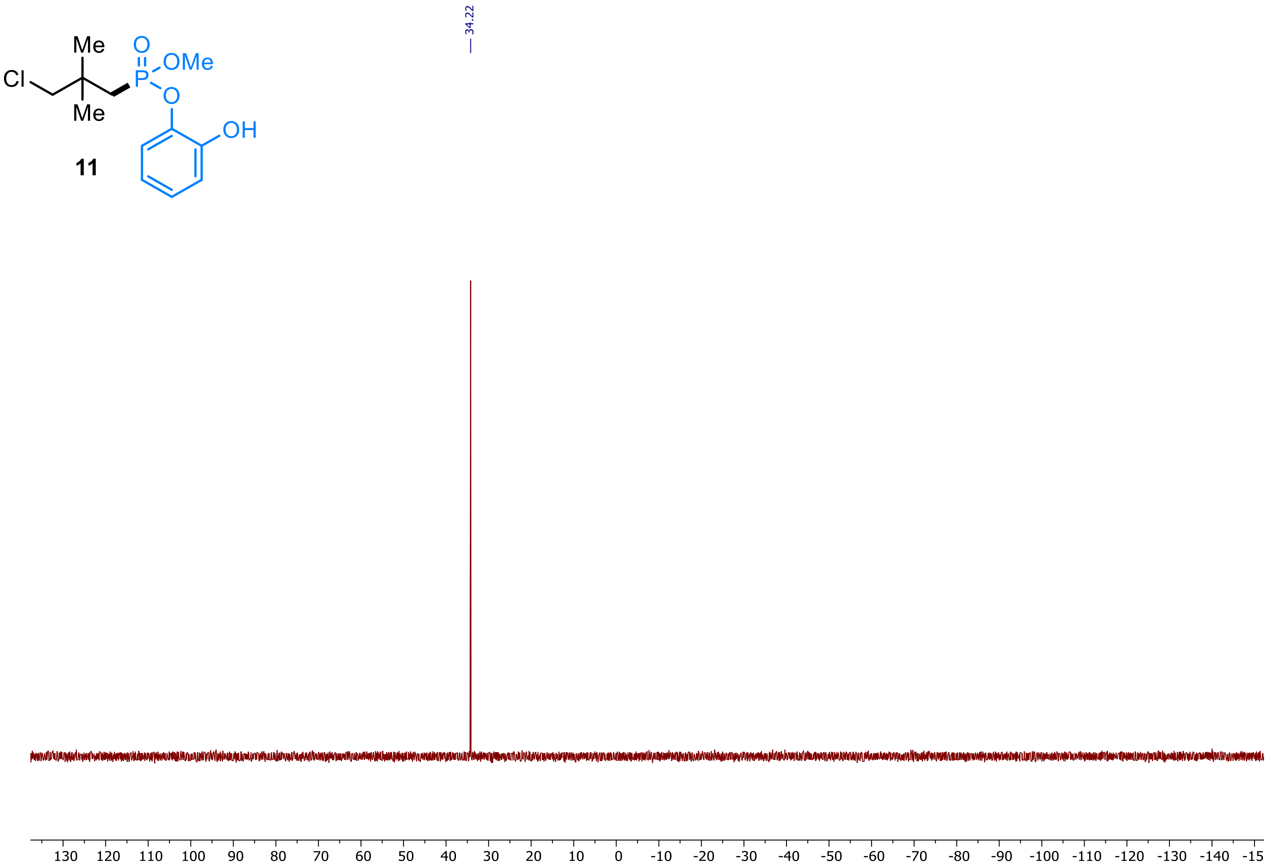

^{31}P NMR (162 MHz, $CDCl_3$) of crude **12-int** (*see procedure*)

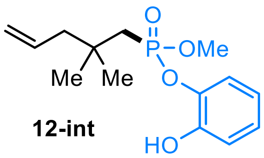

12-int

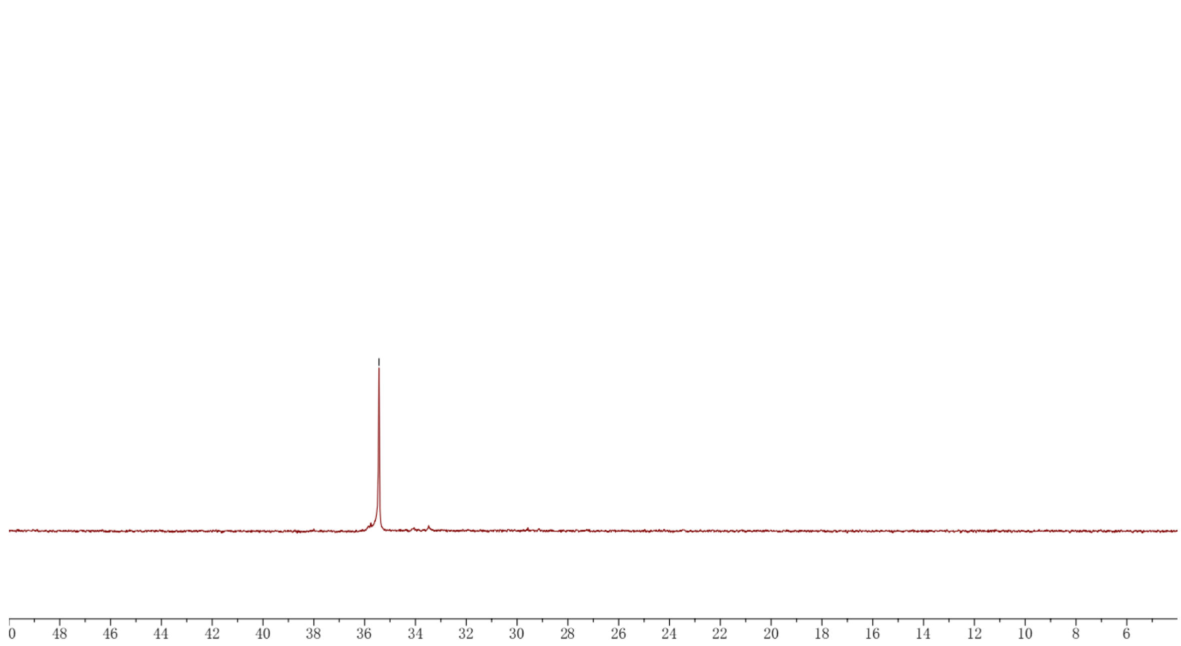

^{1}H NMR (400 MHz, $CDCl_3$) of **12** (*see procedure*)

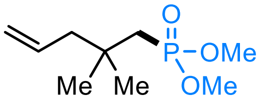

12

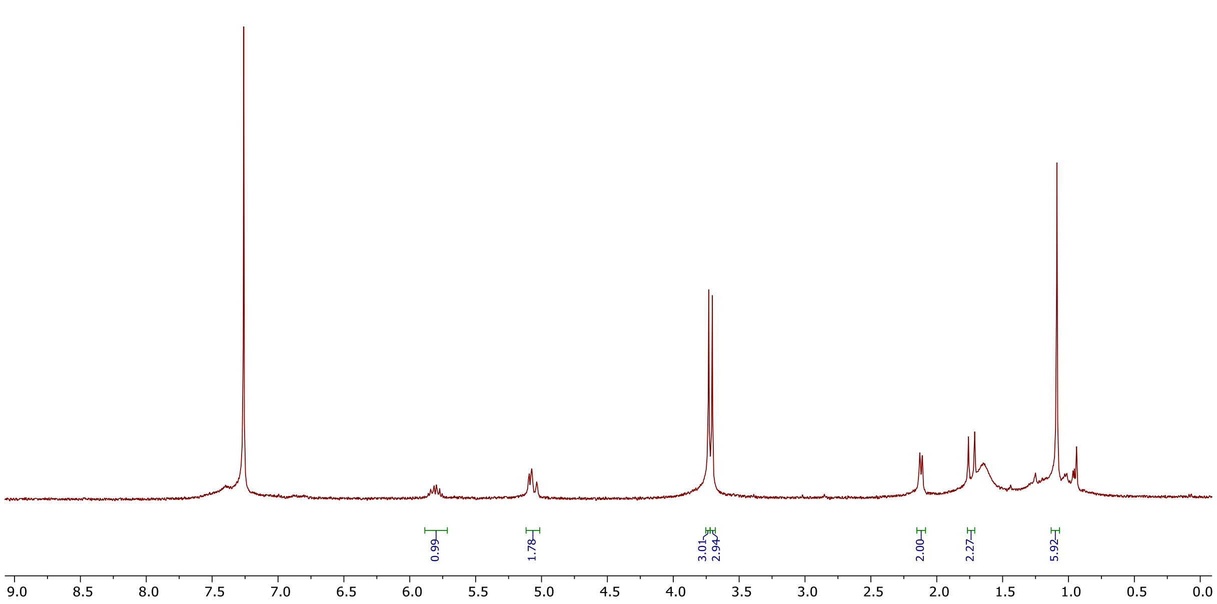

^{13}C NMR (101 MHz, $CDCl_3$) of **12**

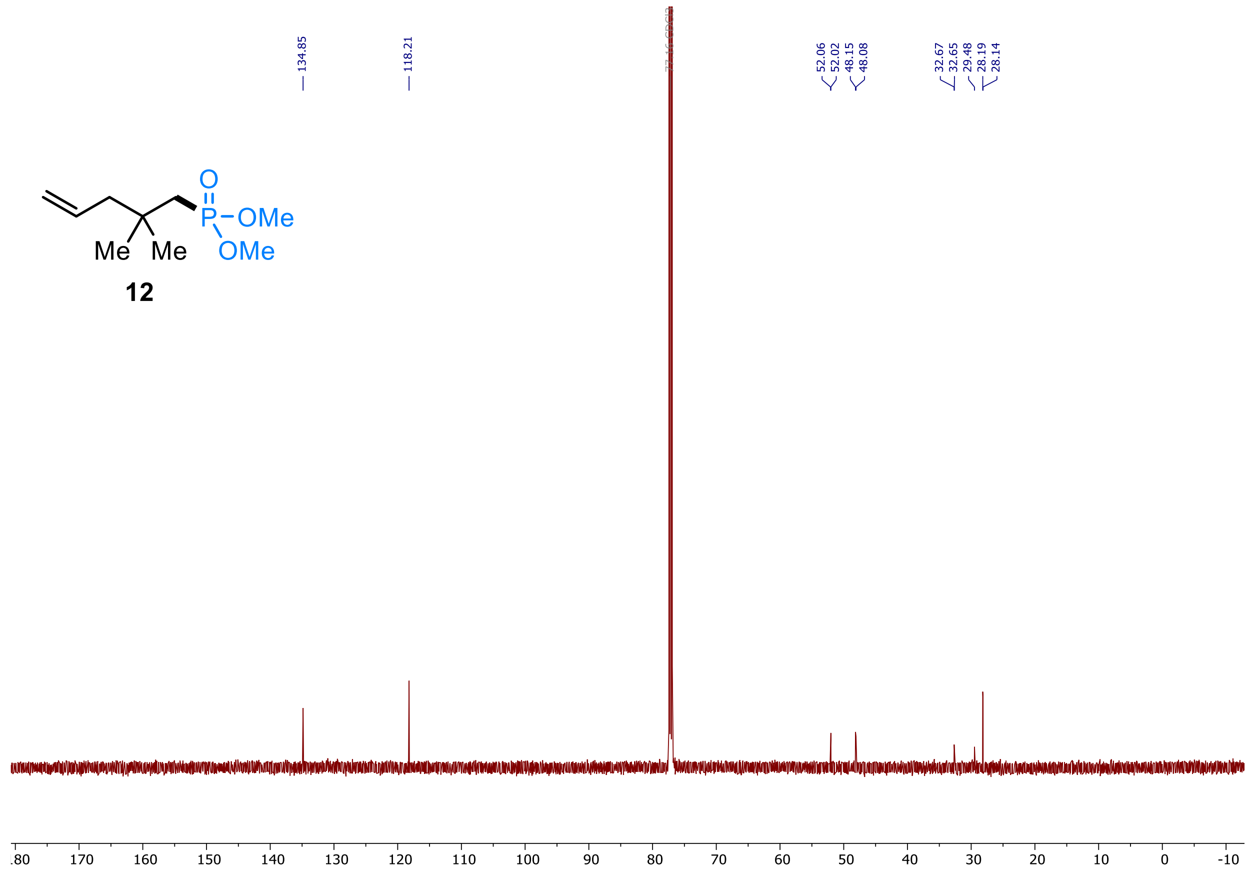

^{31}P NMR (162 MHz, $CDCl_3$) of **12**

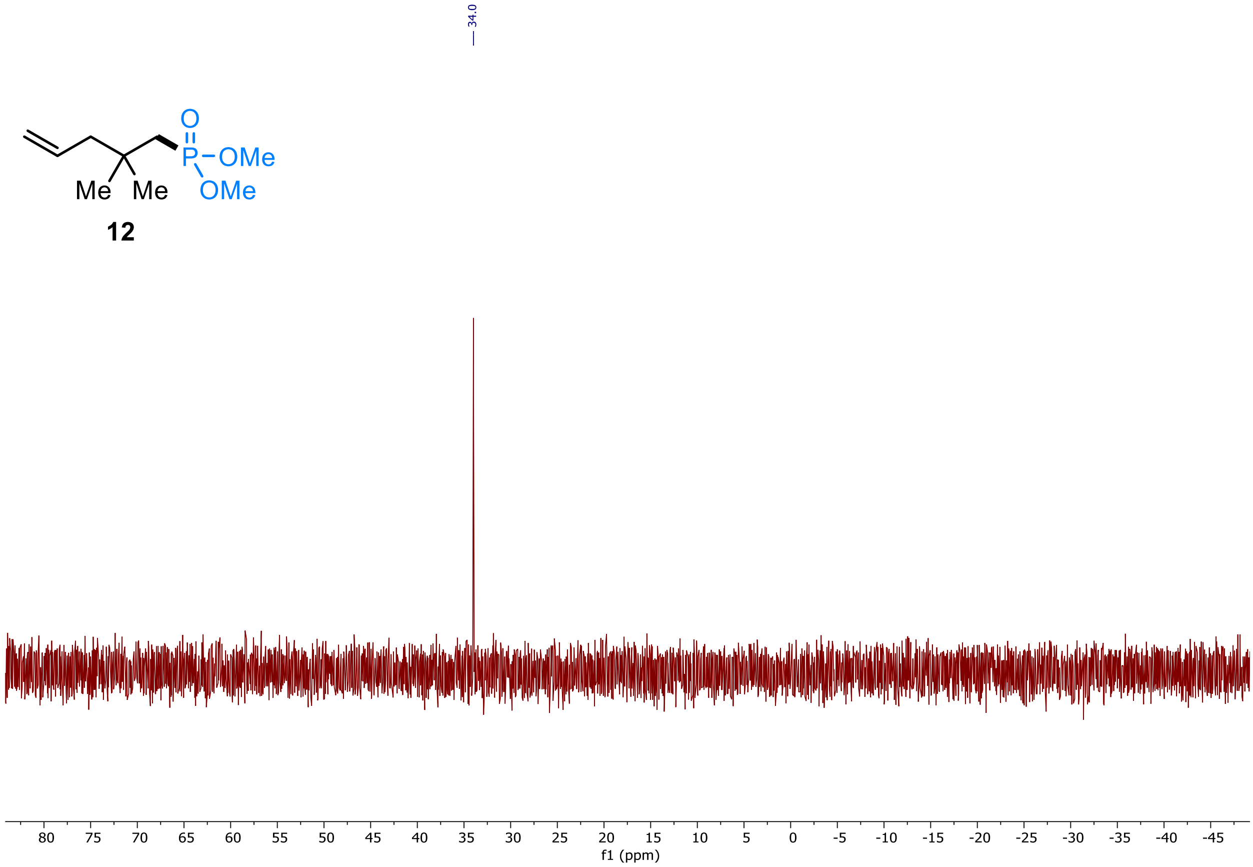

^{31}P NMR (162 MHz, CDCl$_3$) of crude **13-int** (*see procedure*)

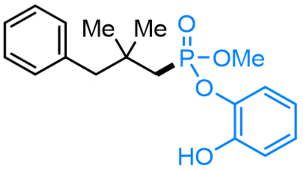

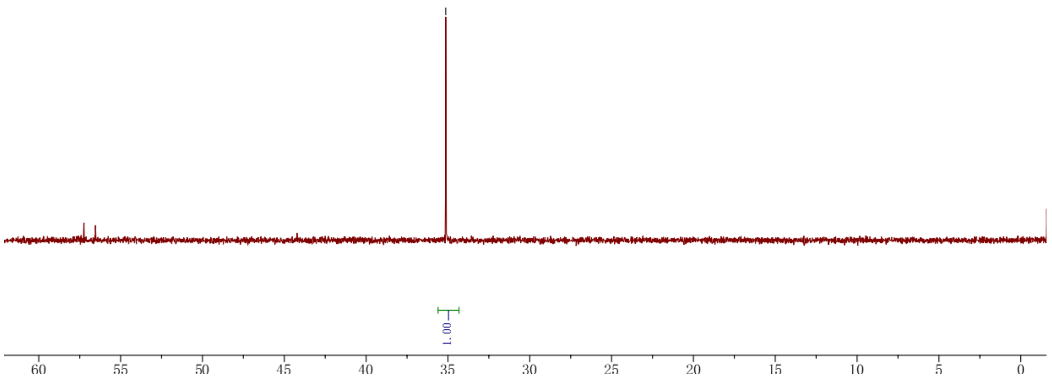

^{1}H NMR (400 MHz, CDCl$_3$) of **13** (*see procedure*)

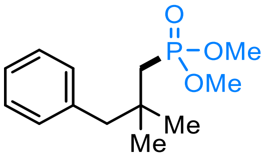

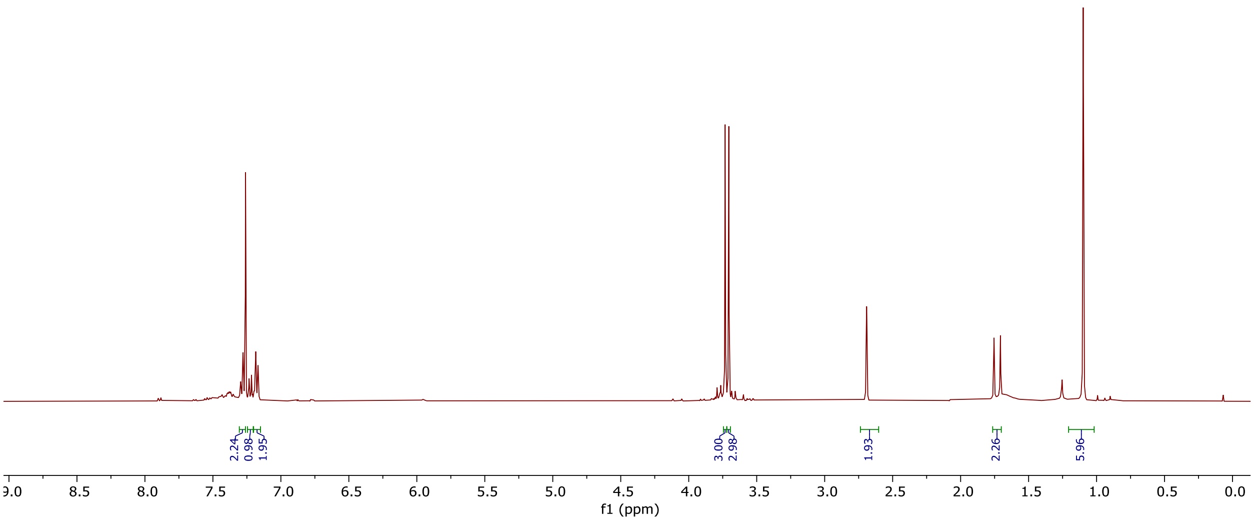

^{13}C NMR (126 MHz, $CDCl_3$) of **13**

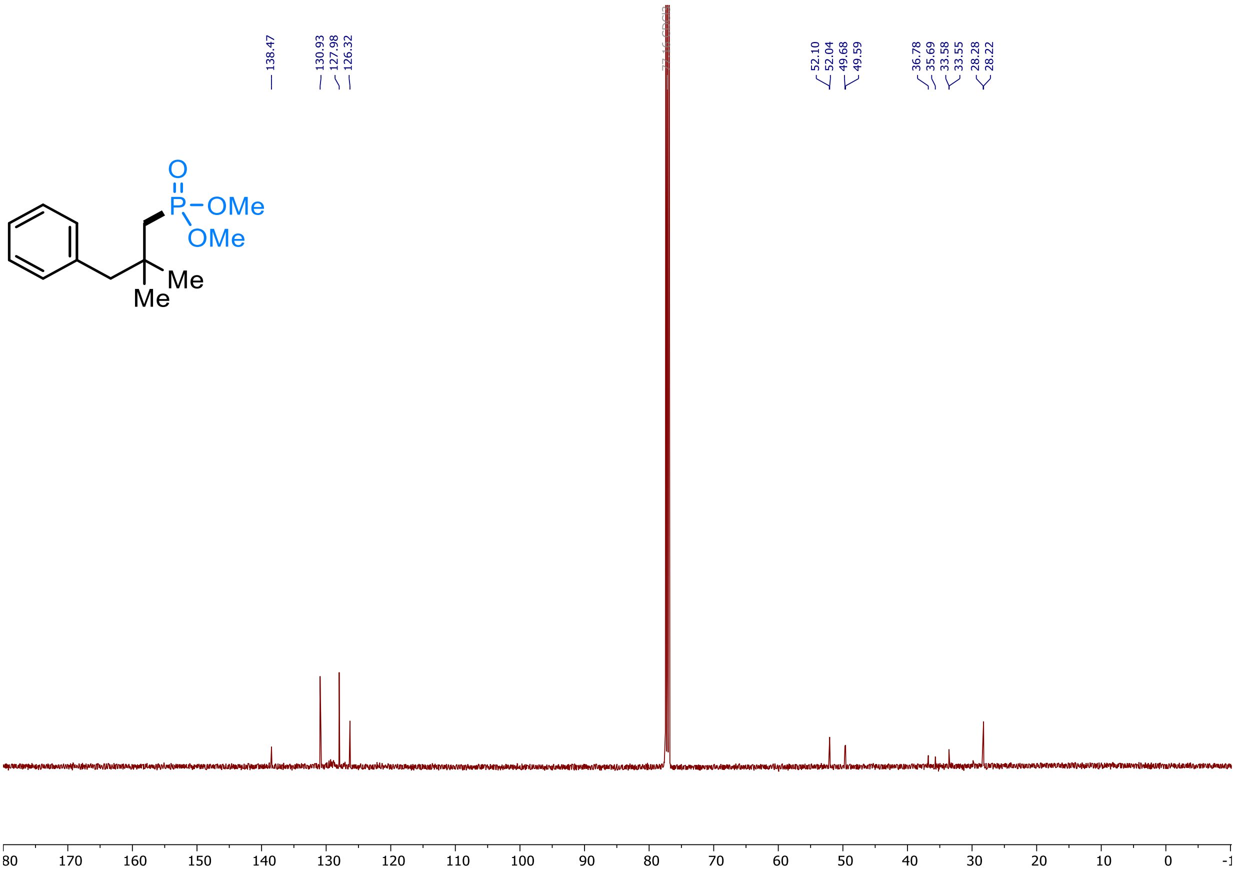

^{31}P NMR (165 MHz, $CDCl_3$) of **13**

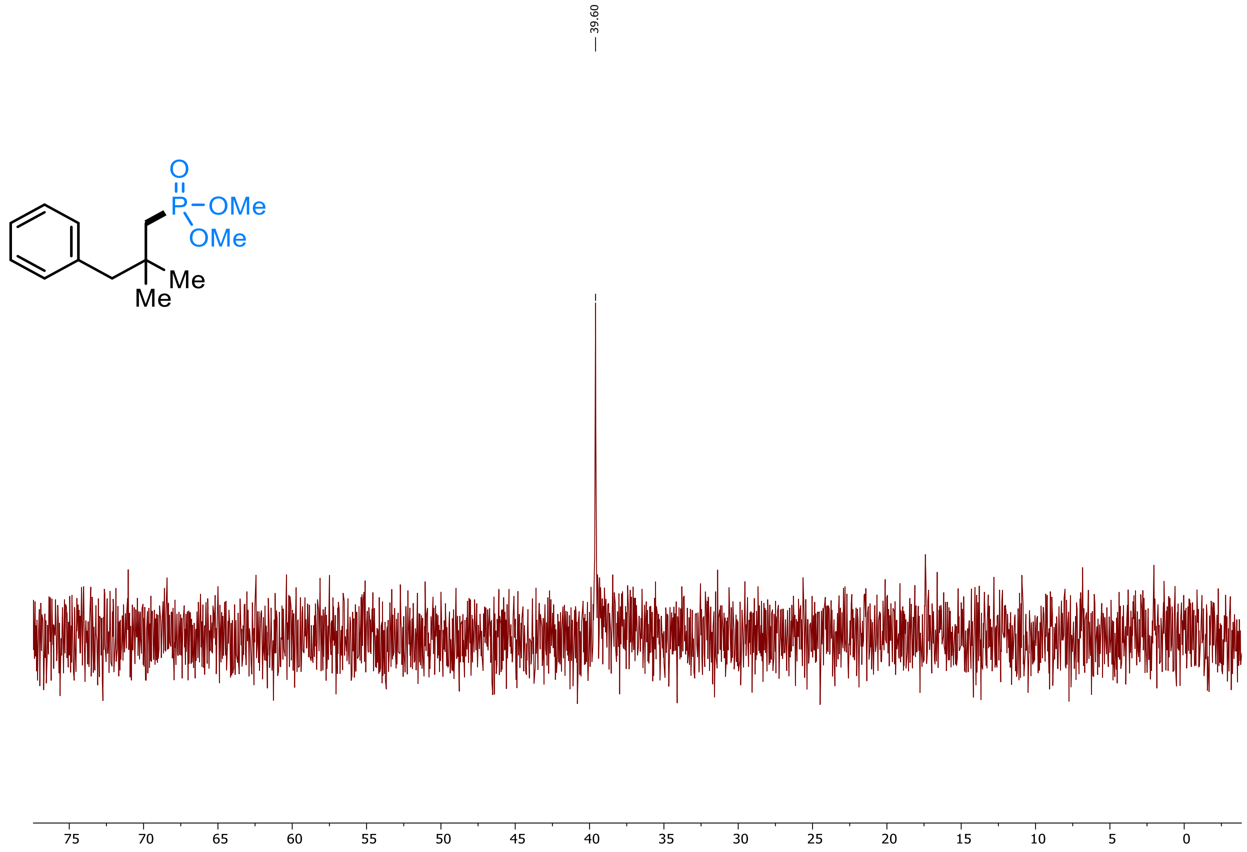

^{1}H NMR (400 MHz, $CDCl_3$) of **14** (*see procedure*)

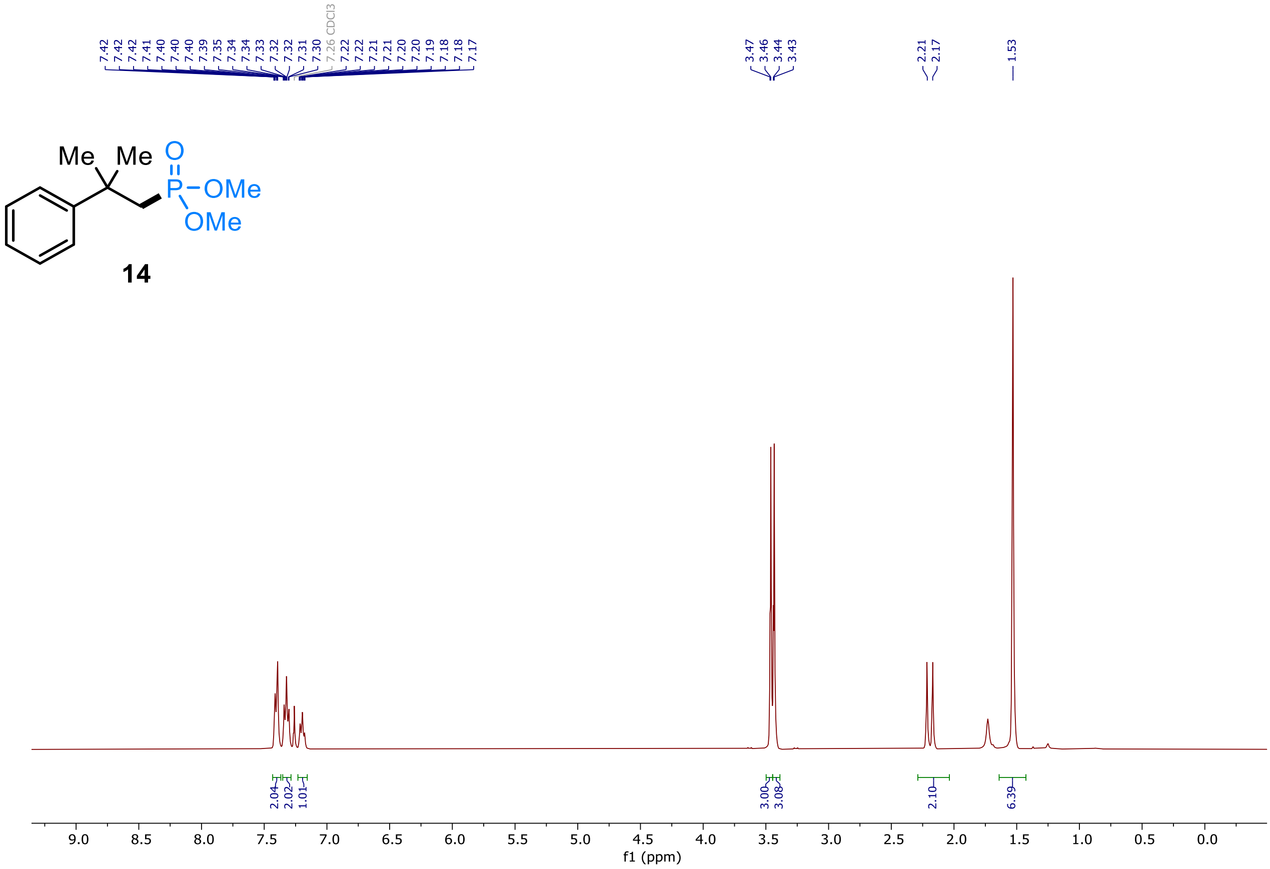

^{13}C NMR (101 MHz, $CDCl_3$) of **14**

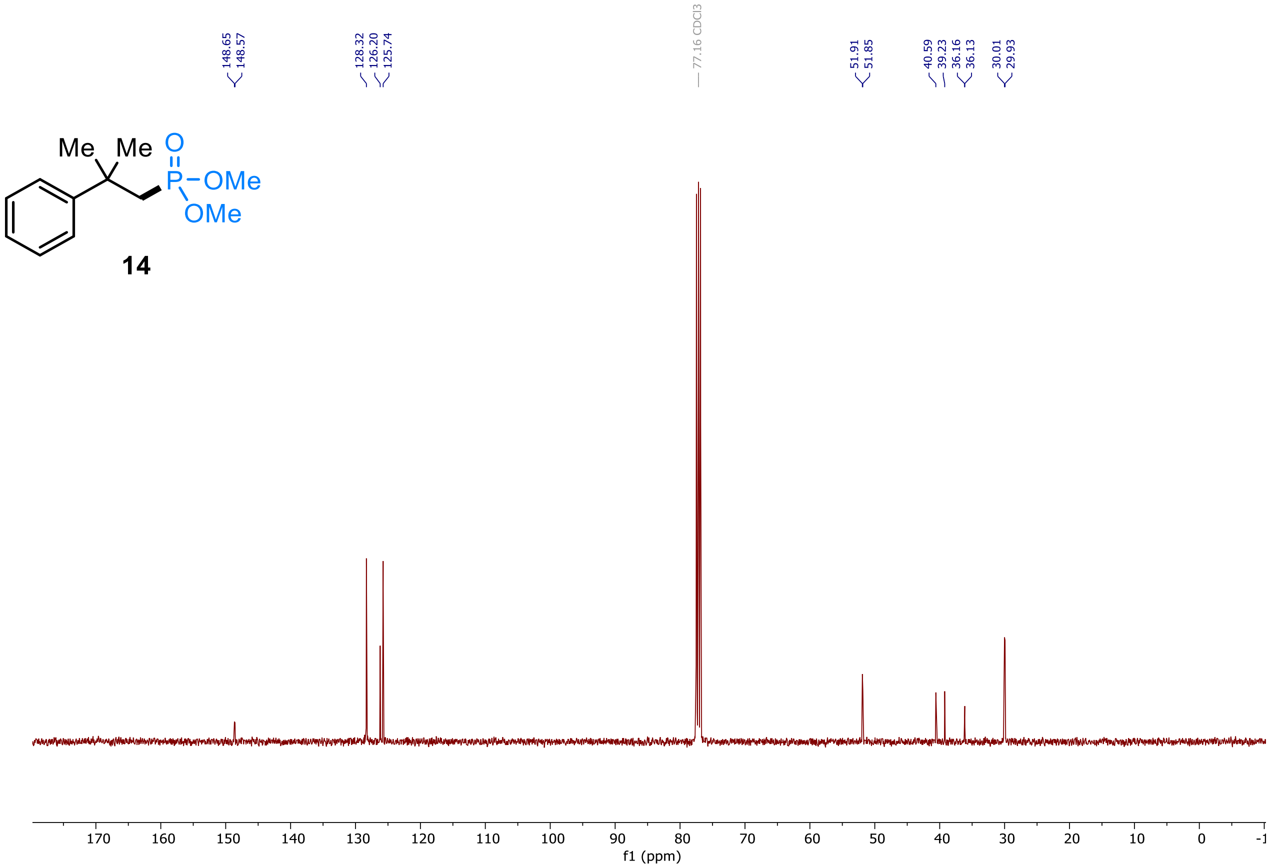

^{31}P NMR (162 MHz, $CDCl_3$) of **14**

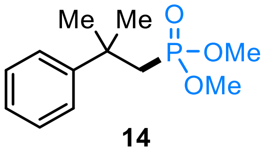

14

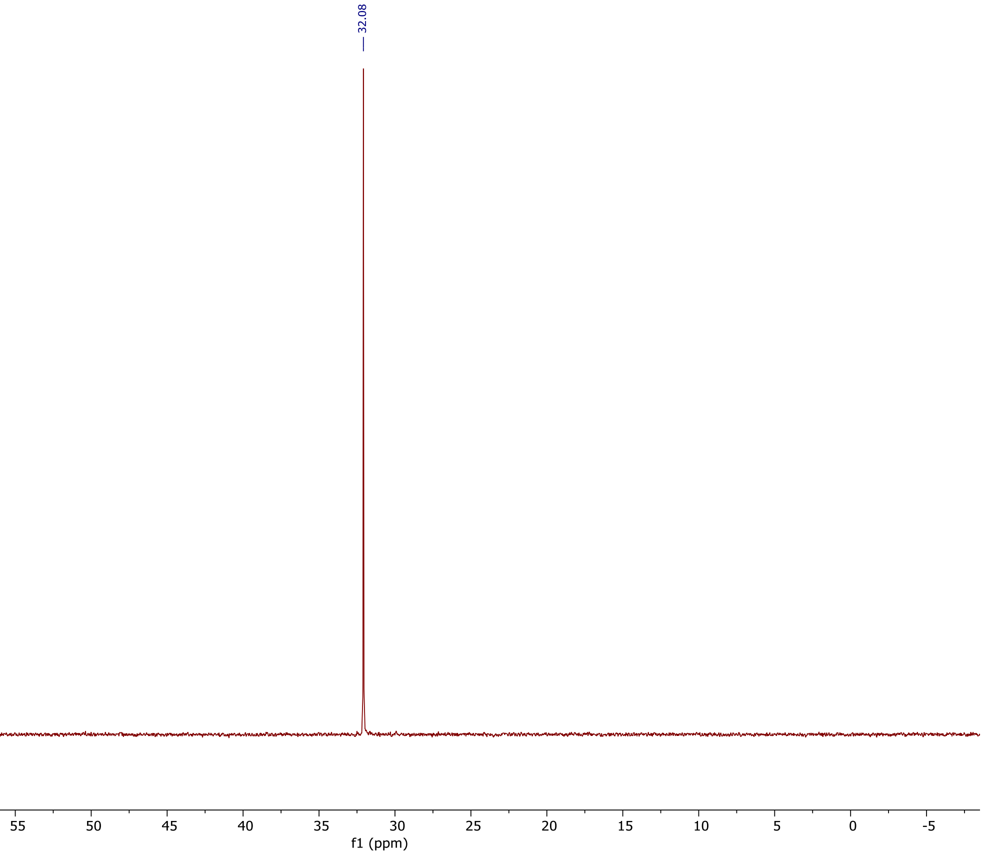

^{1}H NMR (400 MHz, Methanol-d_4) of **15** (see procedure)

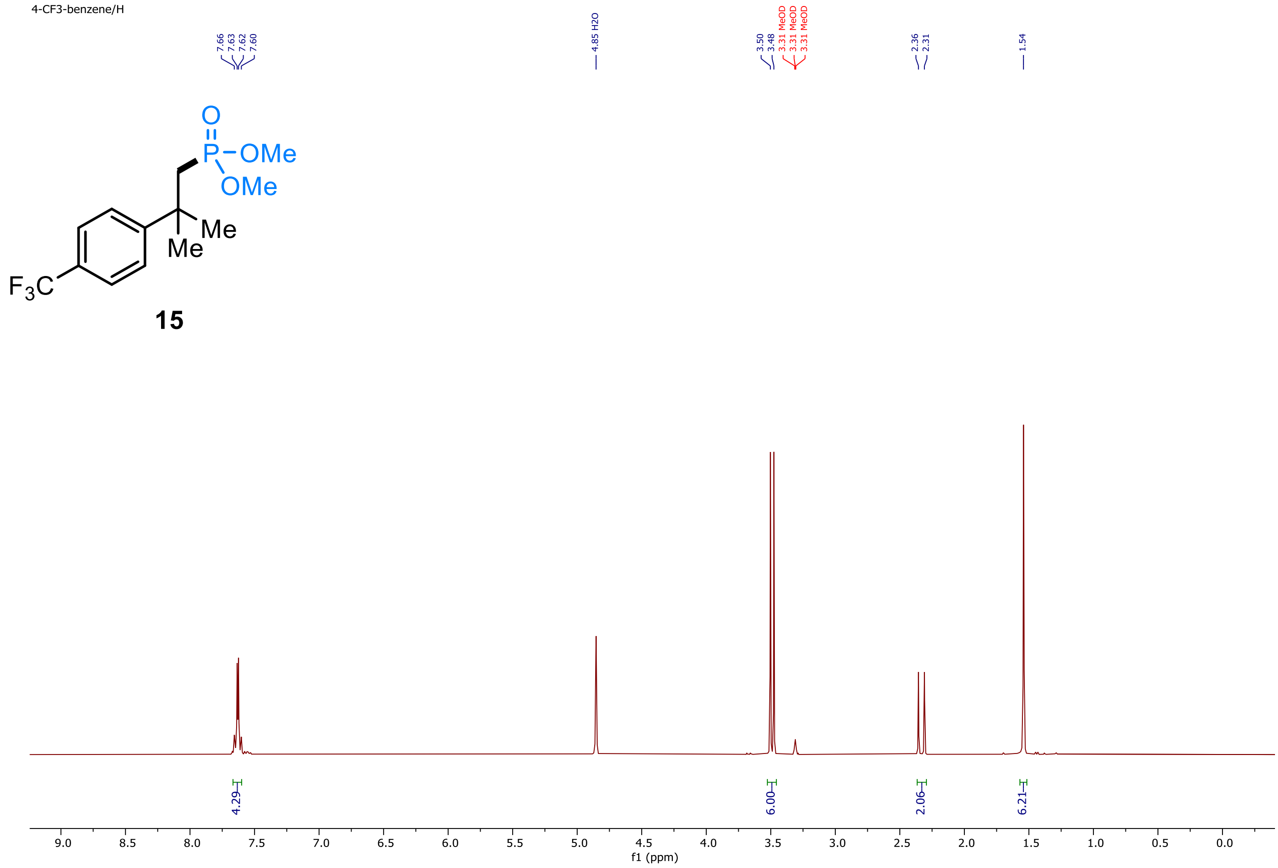

^{13}C NMR (151 MHz, Methanol-d_4) of **15**

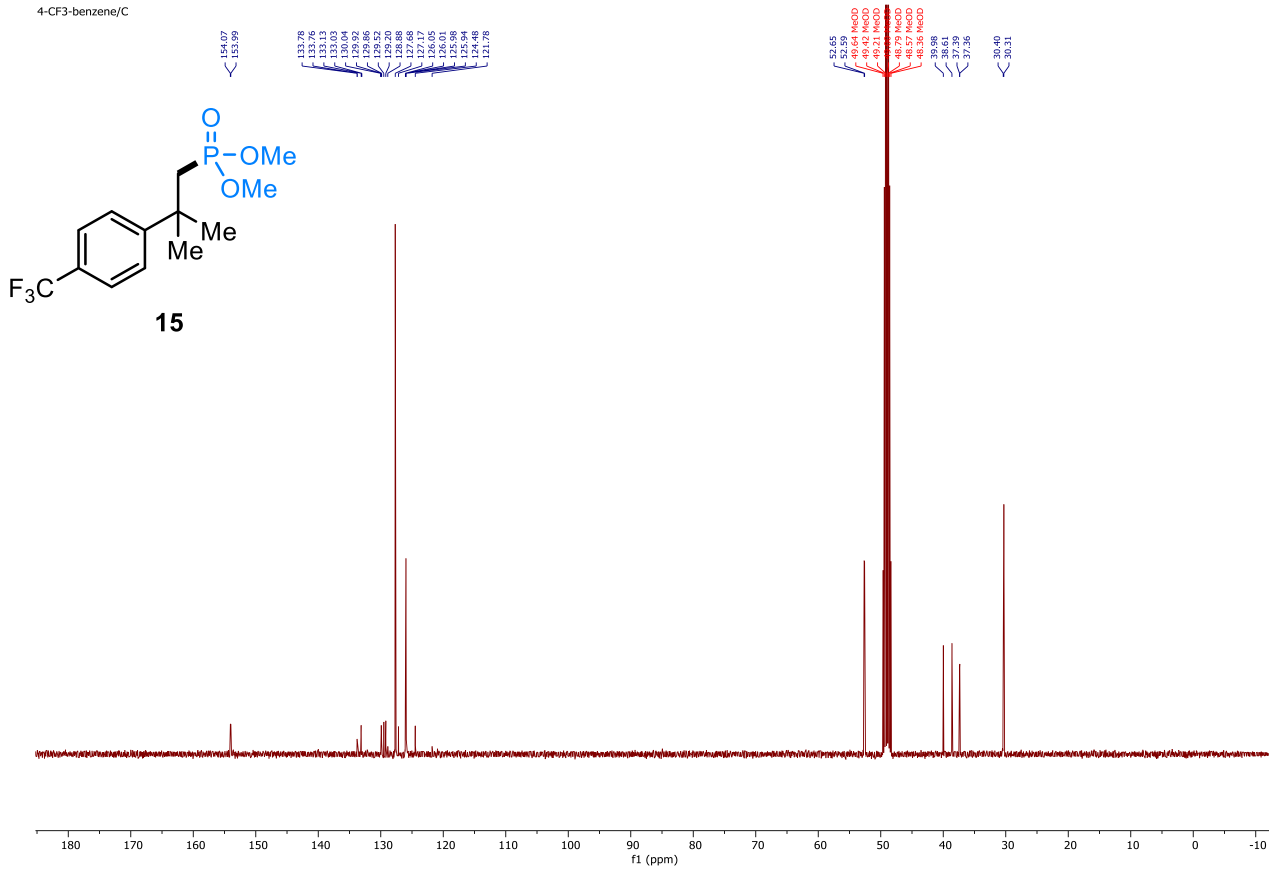

^{31}P NMR (162 MHz, Methanol-d_4) of **15**

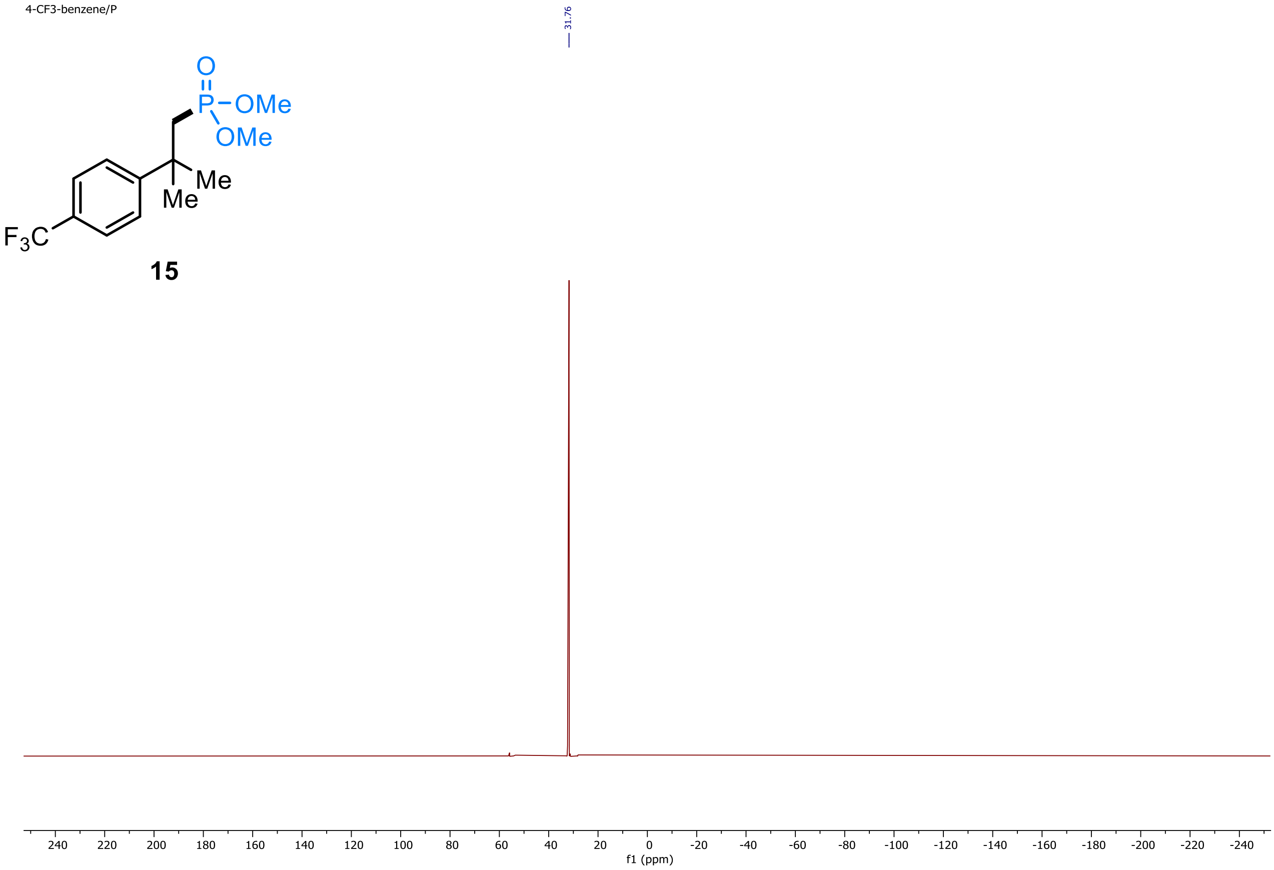

^{19}F NMR (377 MHz, Methanol-d_4) of **15**

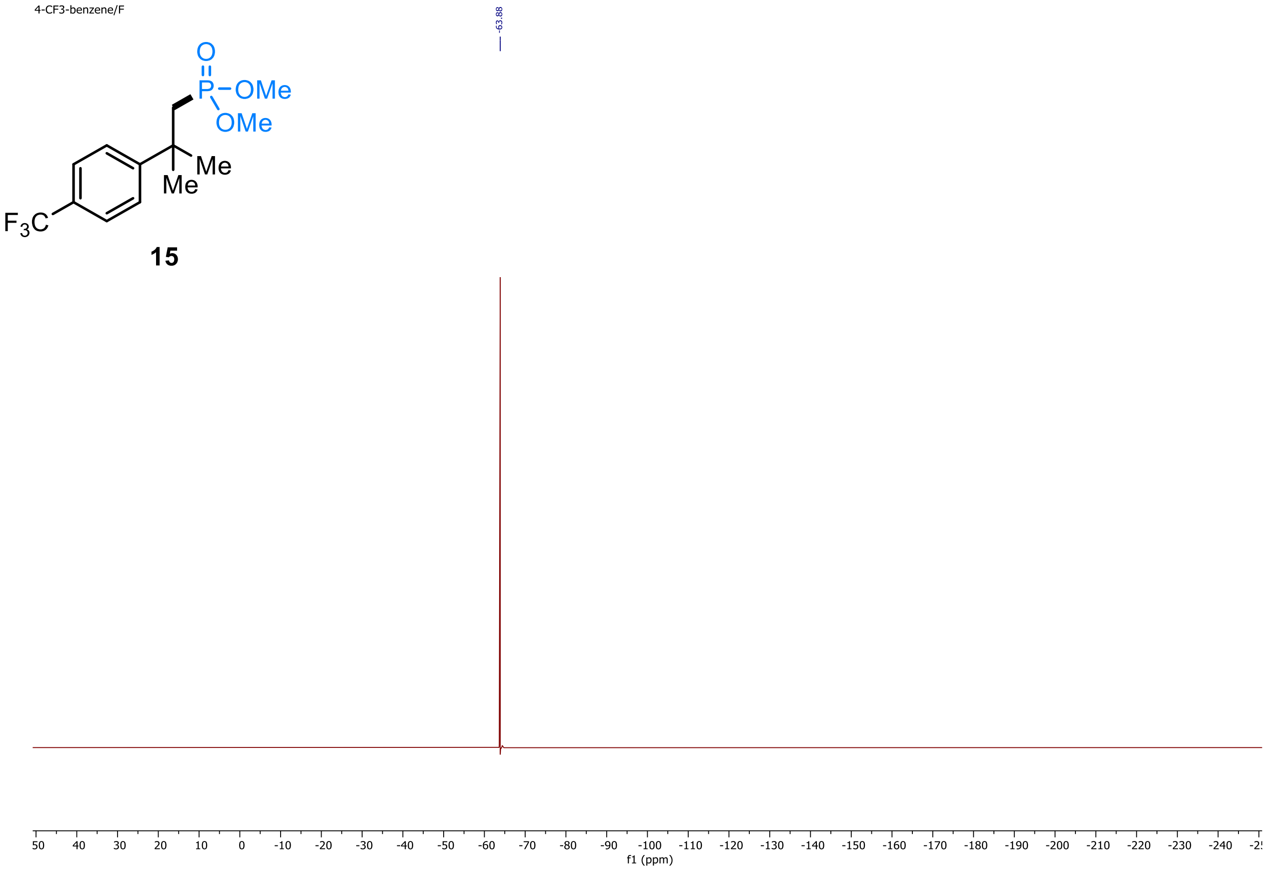

^{31}P NMR of crude reaction mixture of **16-int** (see procedure)

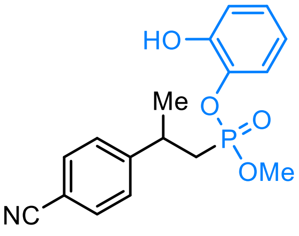

16-int

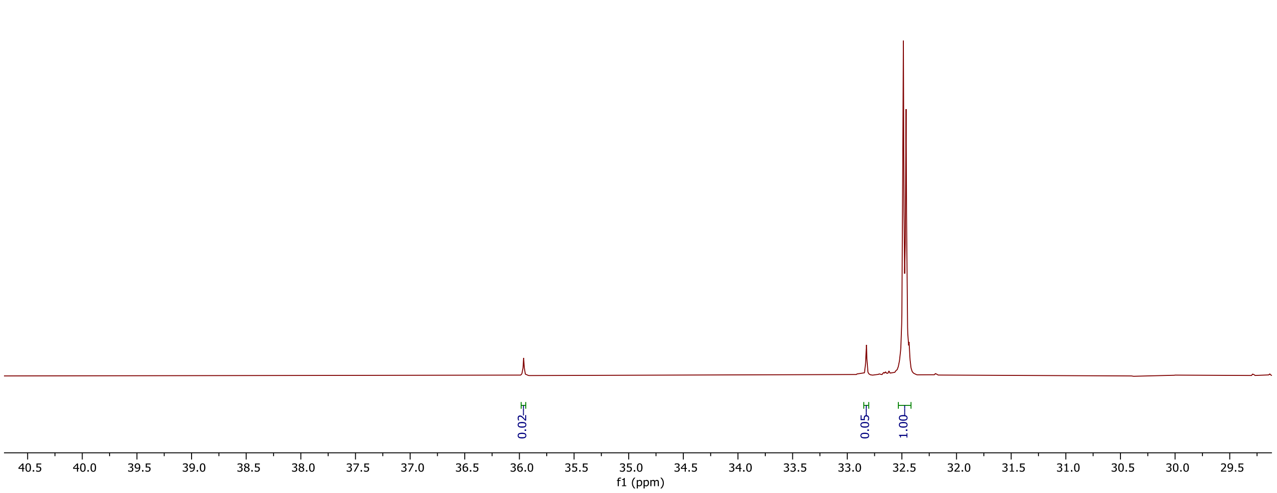

^{1}H NMR (400 MHz, $CDCl_3$) of **16** (see procedure)

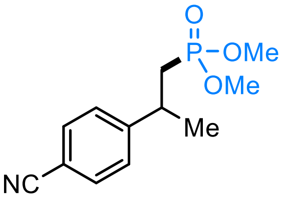

16

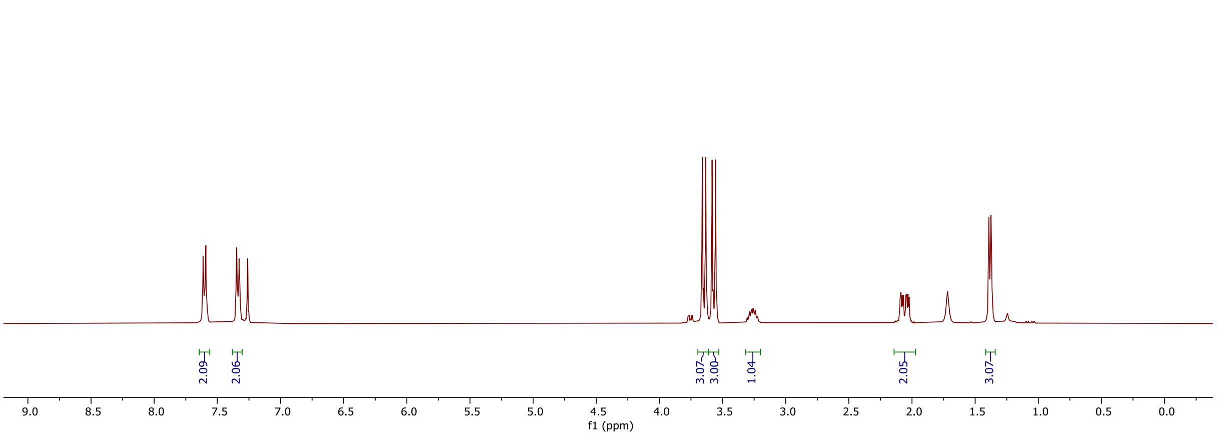

^{13}C NMR (151 MHz, $CDCl_3$) of **16**

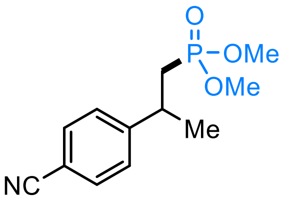

16

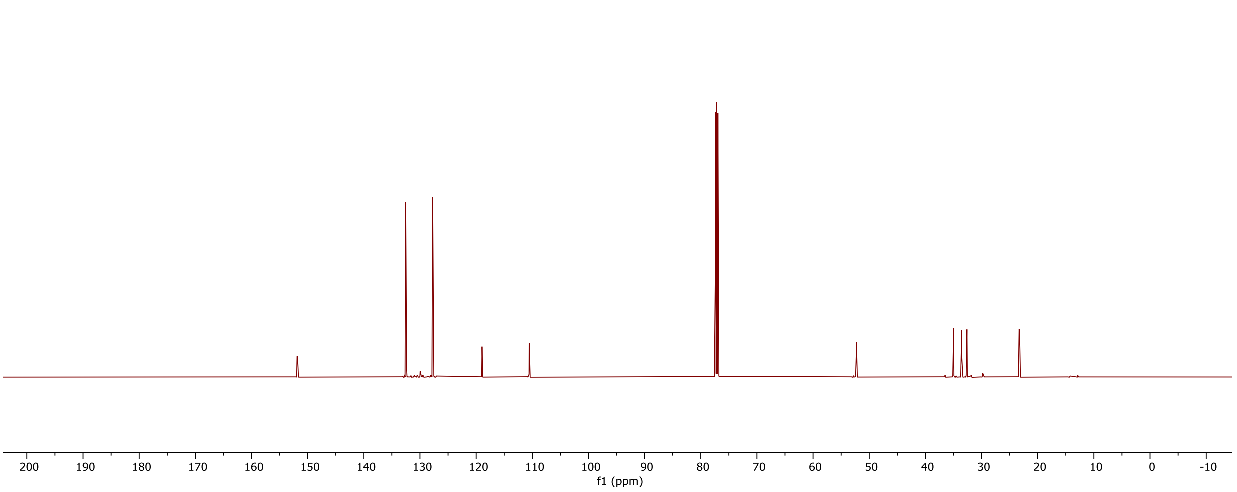

^{31}P NMR (162 MHz, $CDCl_3$) of **16**

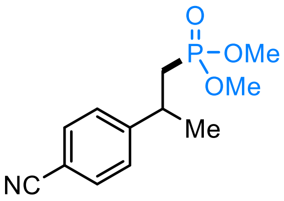

16

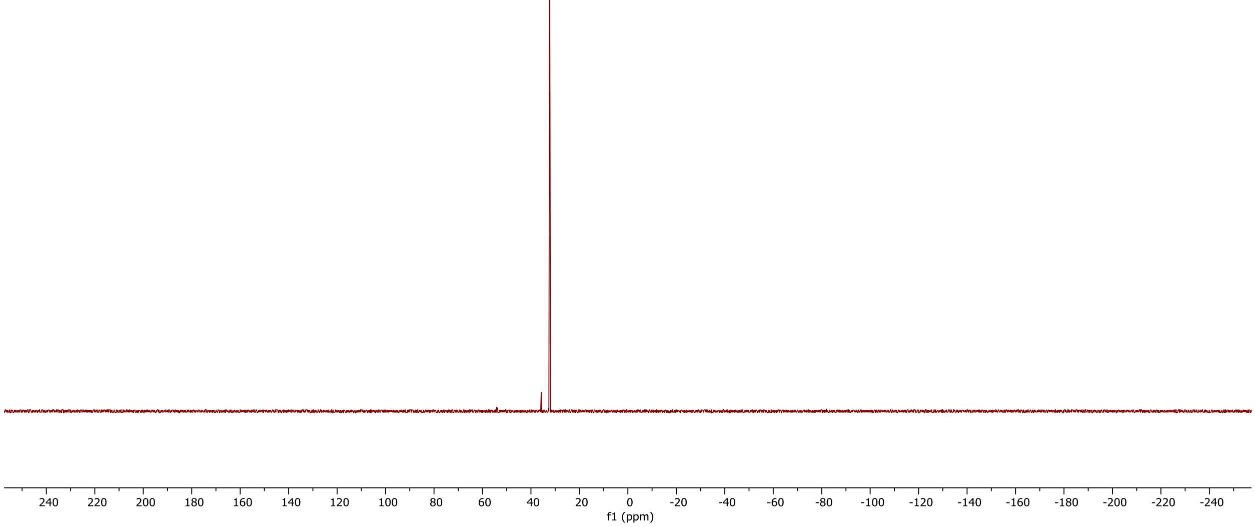

^{31}P NMR (162 MHz, $CDCl_3$) of crude **17** (*see procedure*)

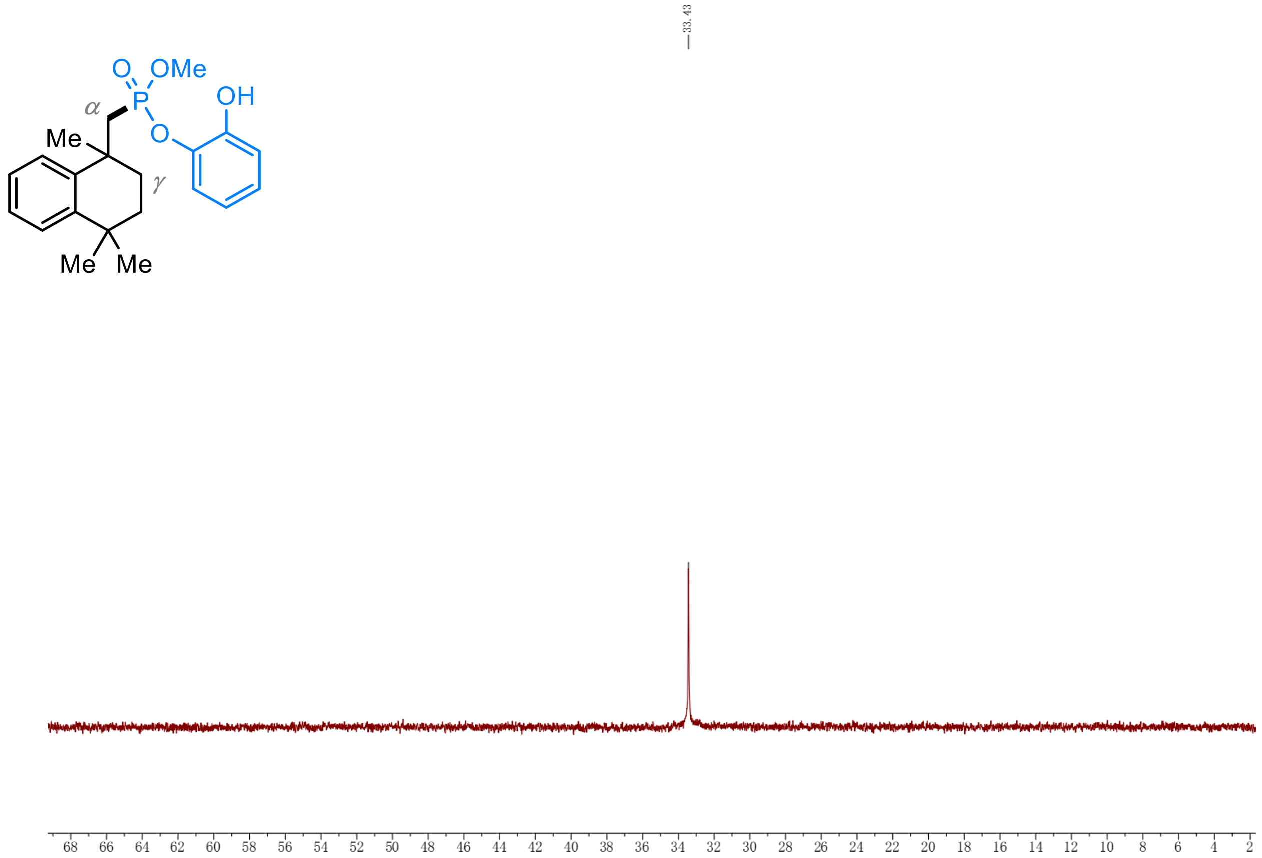

^{1}H NMR (400 MHz, $CDCl_3$) of **17** (*see procedure*)

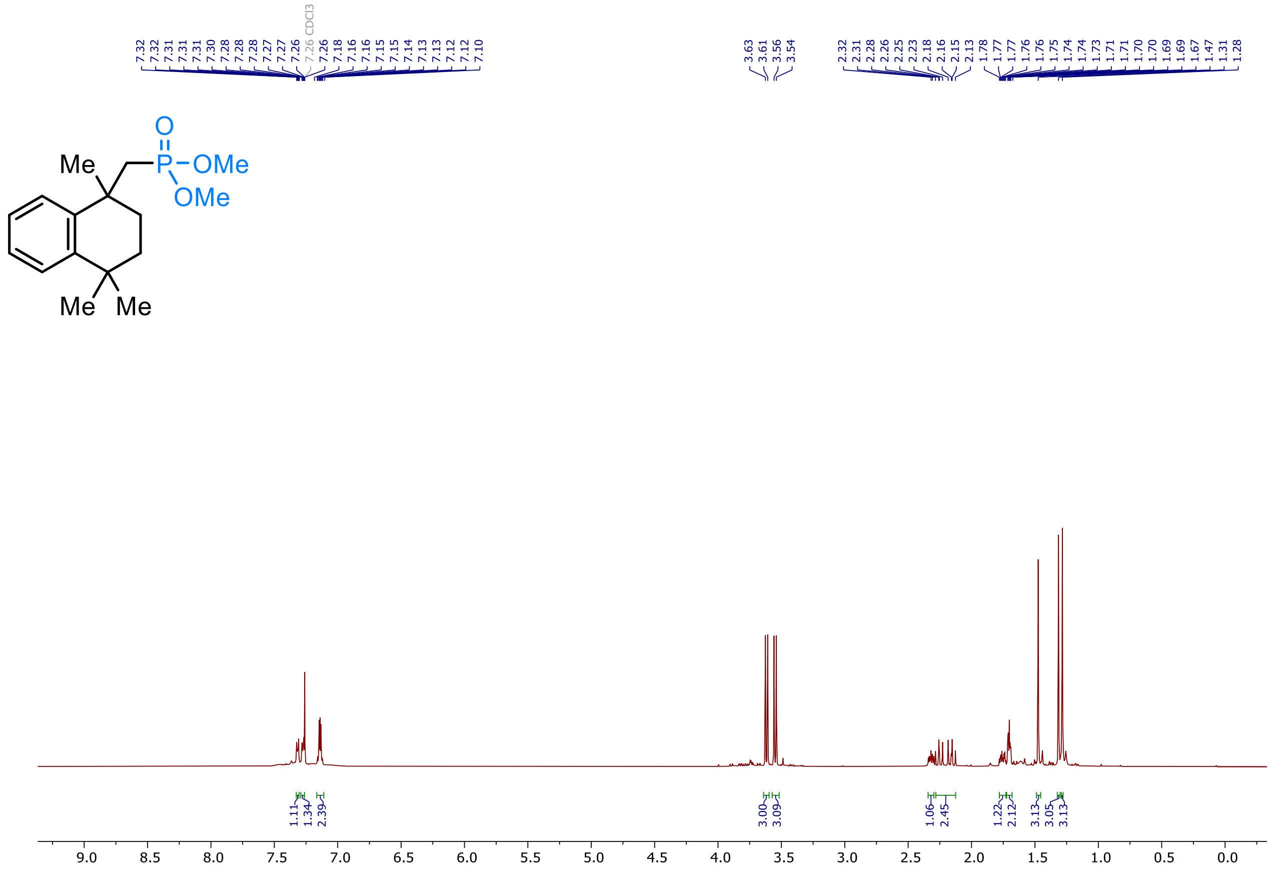

^{13}C NMR (151 MHz, $CDCl_3$) of **17**

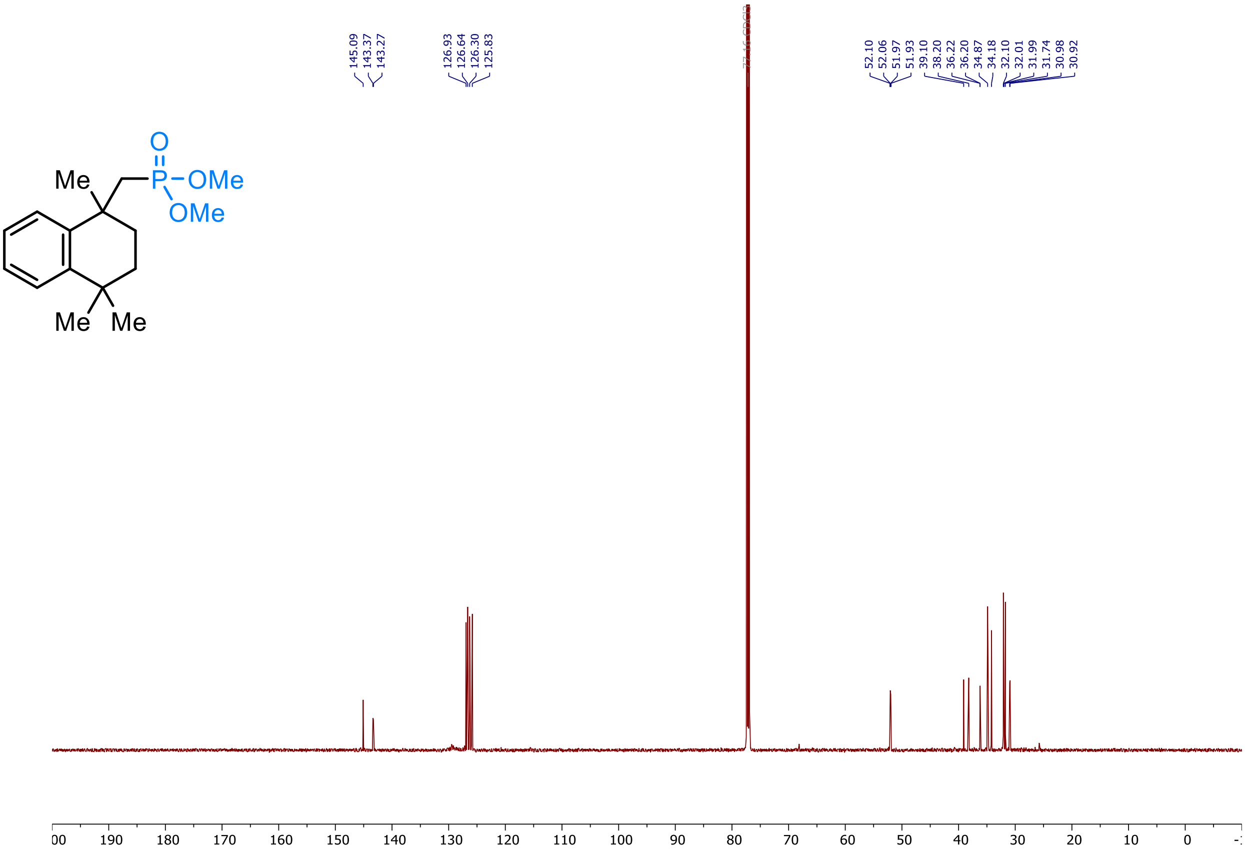

^{31}P NMR (162 MHz, $CDCl_3$) of **17**

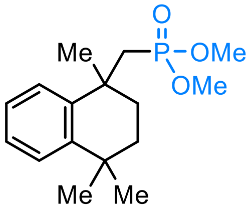

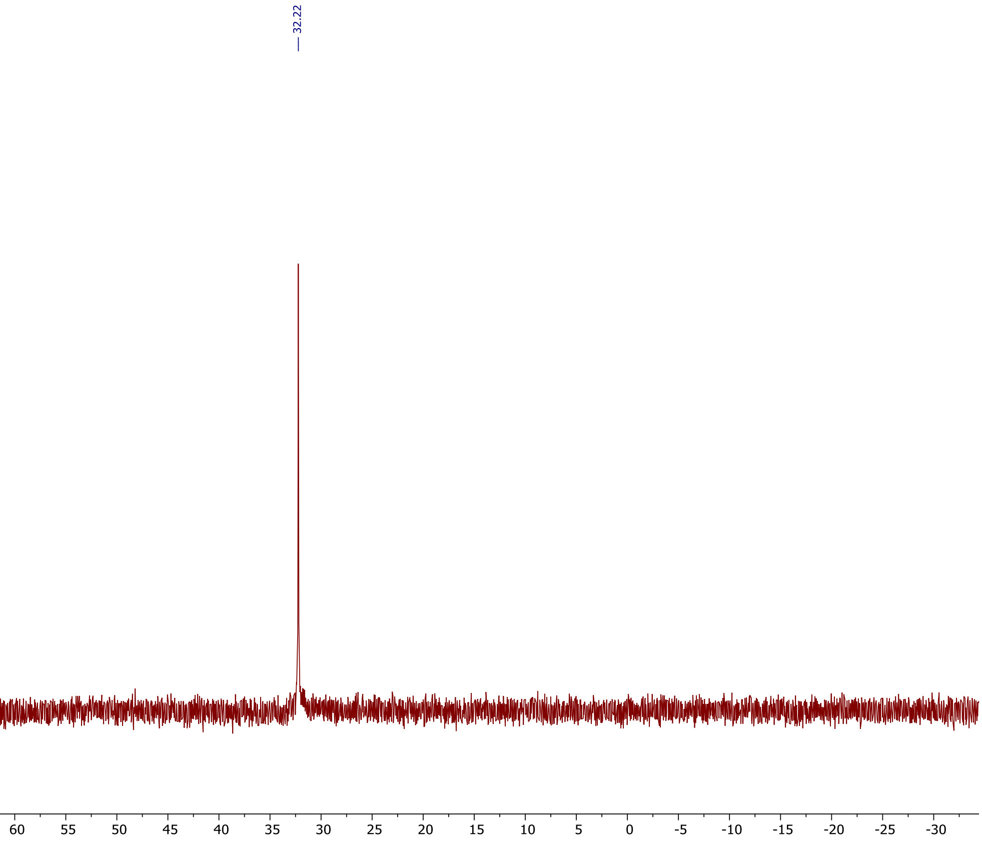

^{31}P NMR (162 MHz, $CDCl_3$) of crude **18-int** (*see procedure*)

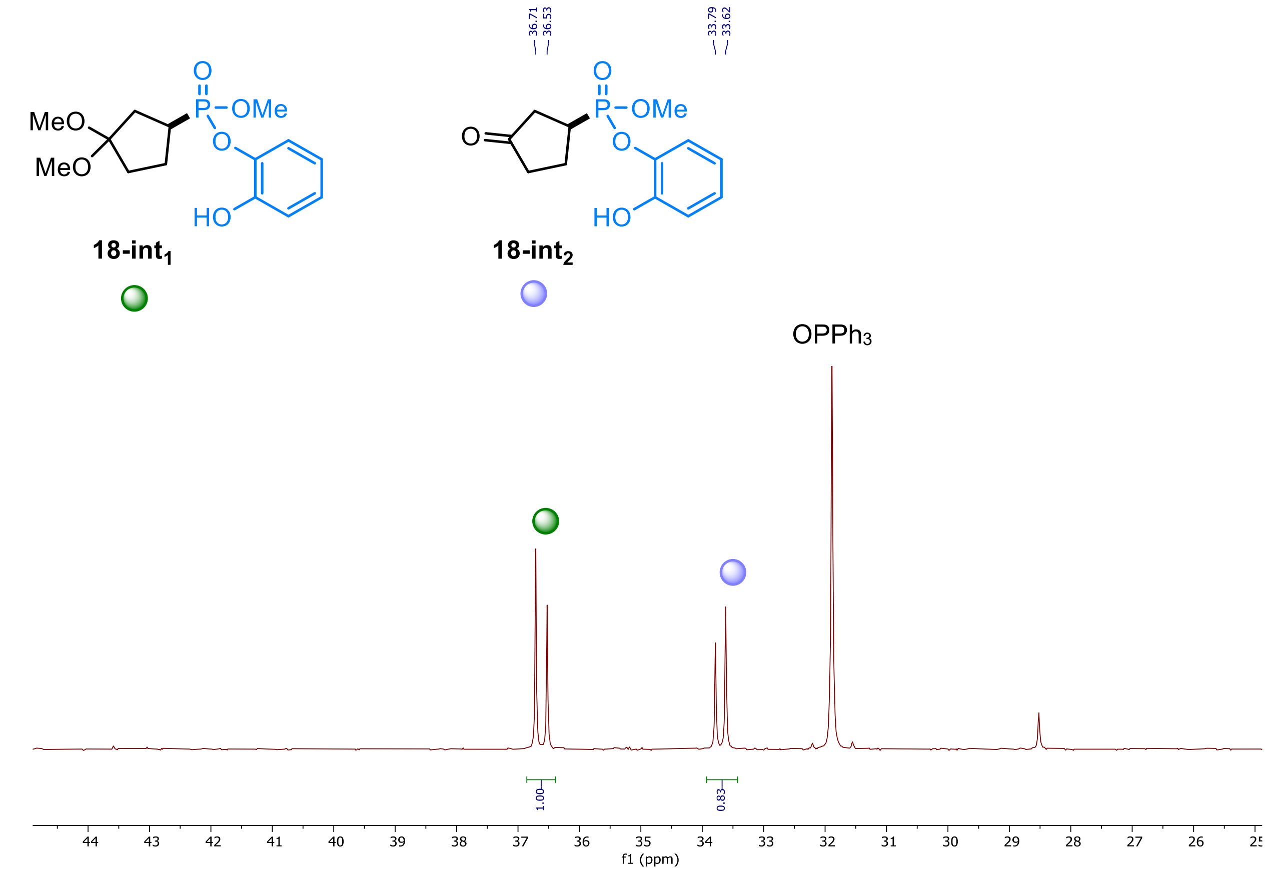

^{1}H NMR (400 MHz, $CDCl_3$) of **18** (*see procedure*)

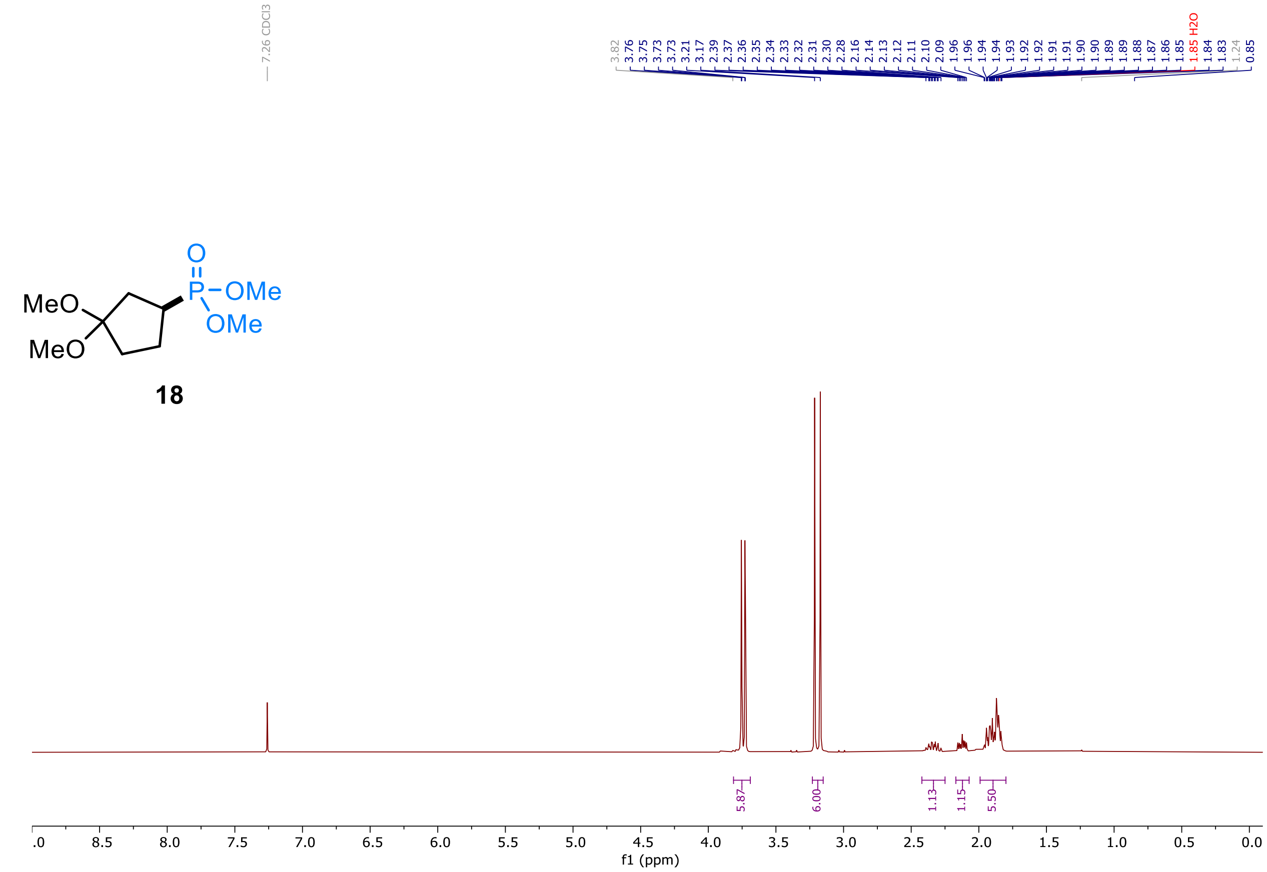

^{13}C NMR (126 MHz, $CDCl_3$) of **18**

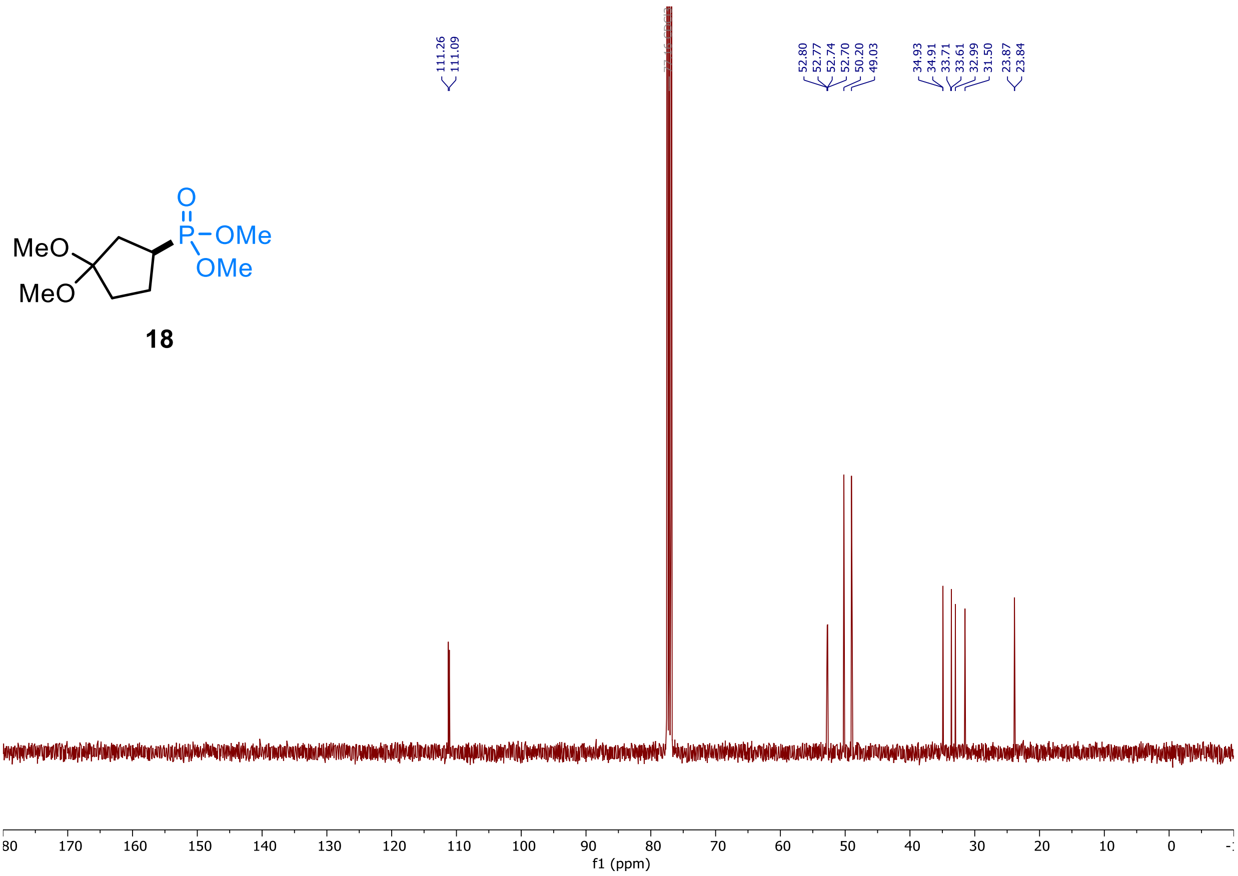

18

^{31}P NMR (165 MHz, $CDCl_3$) of **18**

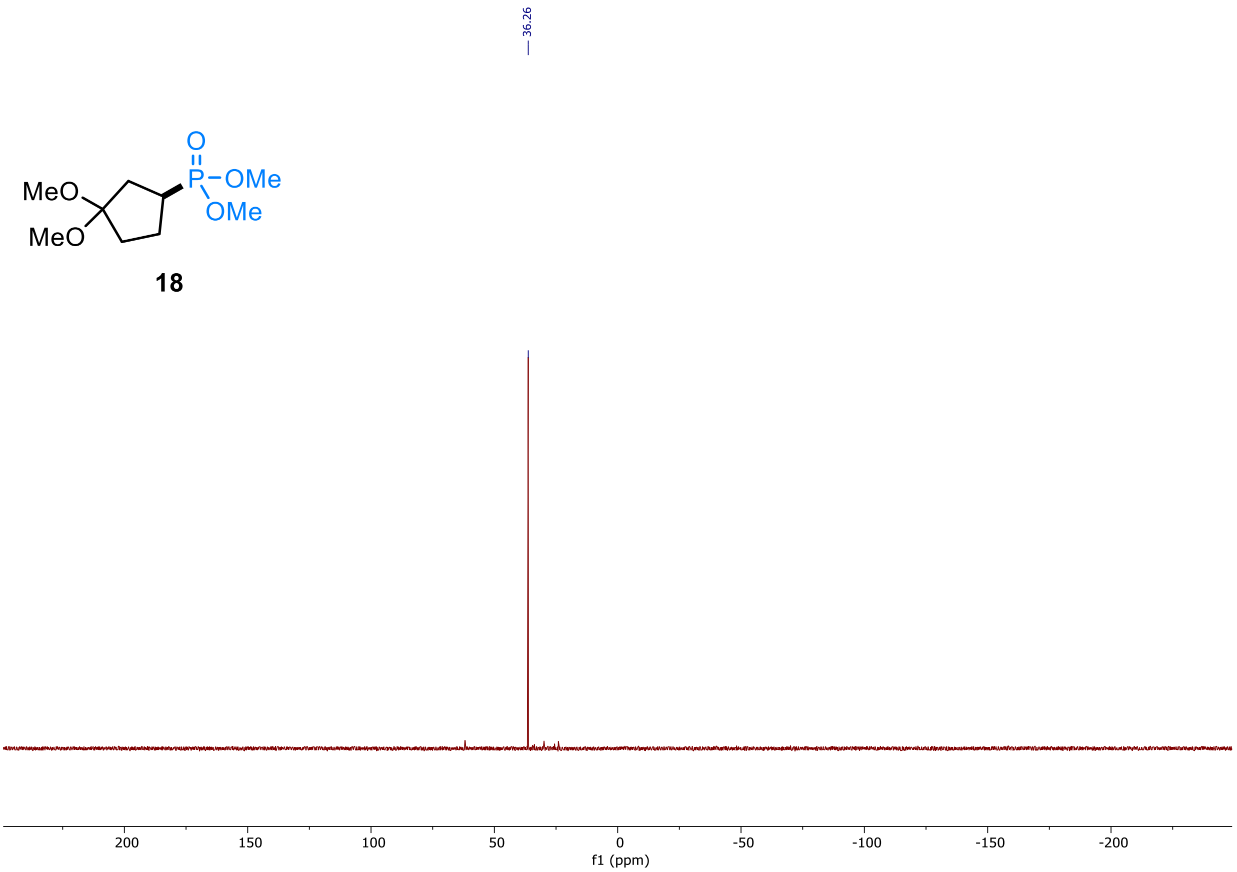

18

HSQC (400 MHz, CDCl3) of **18**

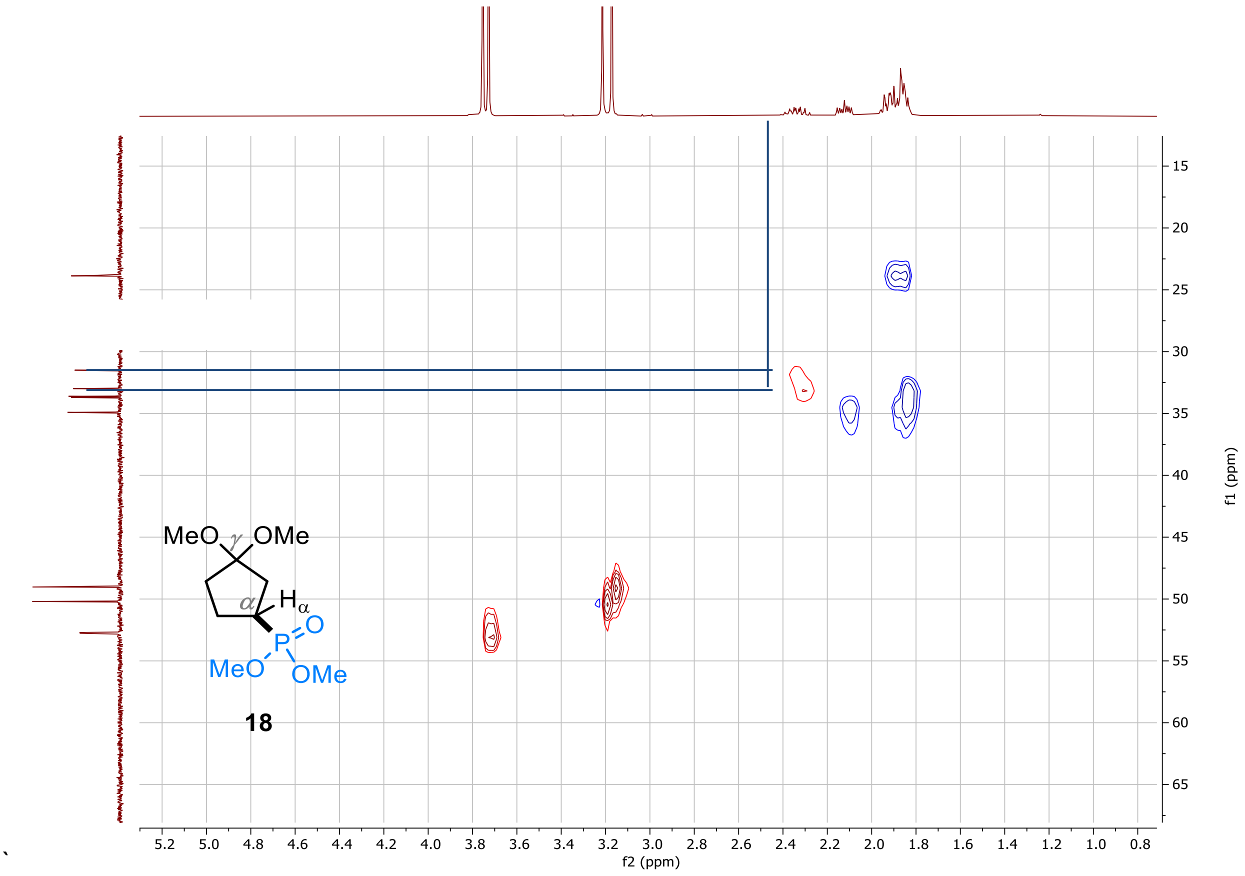

HMBC (600 MHz, CDCl3) of **18**

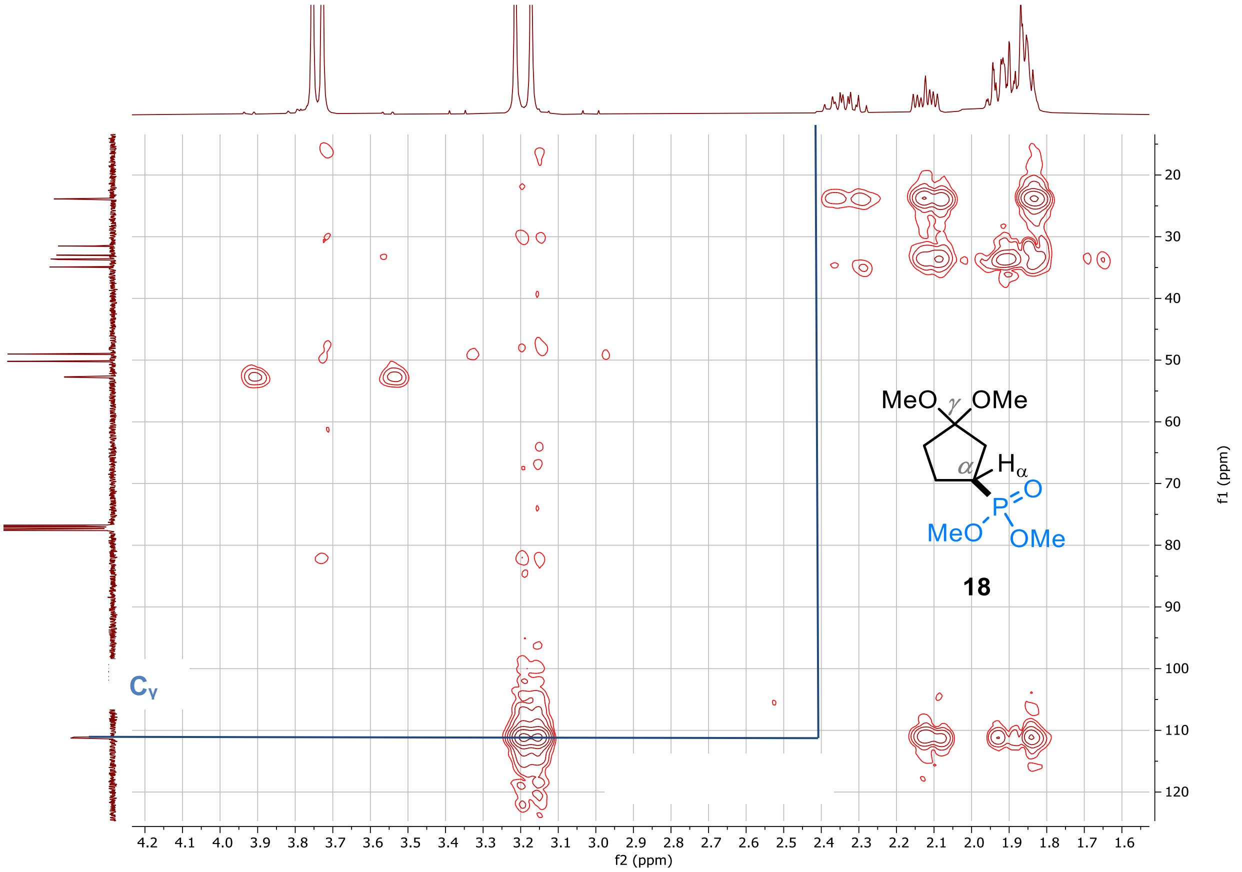

^{31}P NMR (162 MHz, $CDCl_3$) of crude **19-int** (*see procedure*)

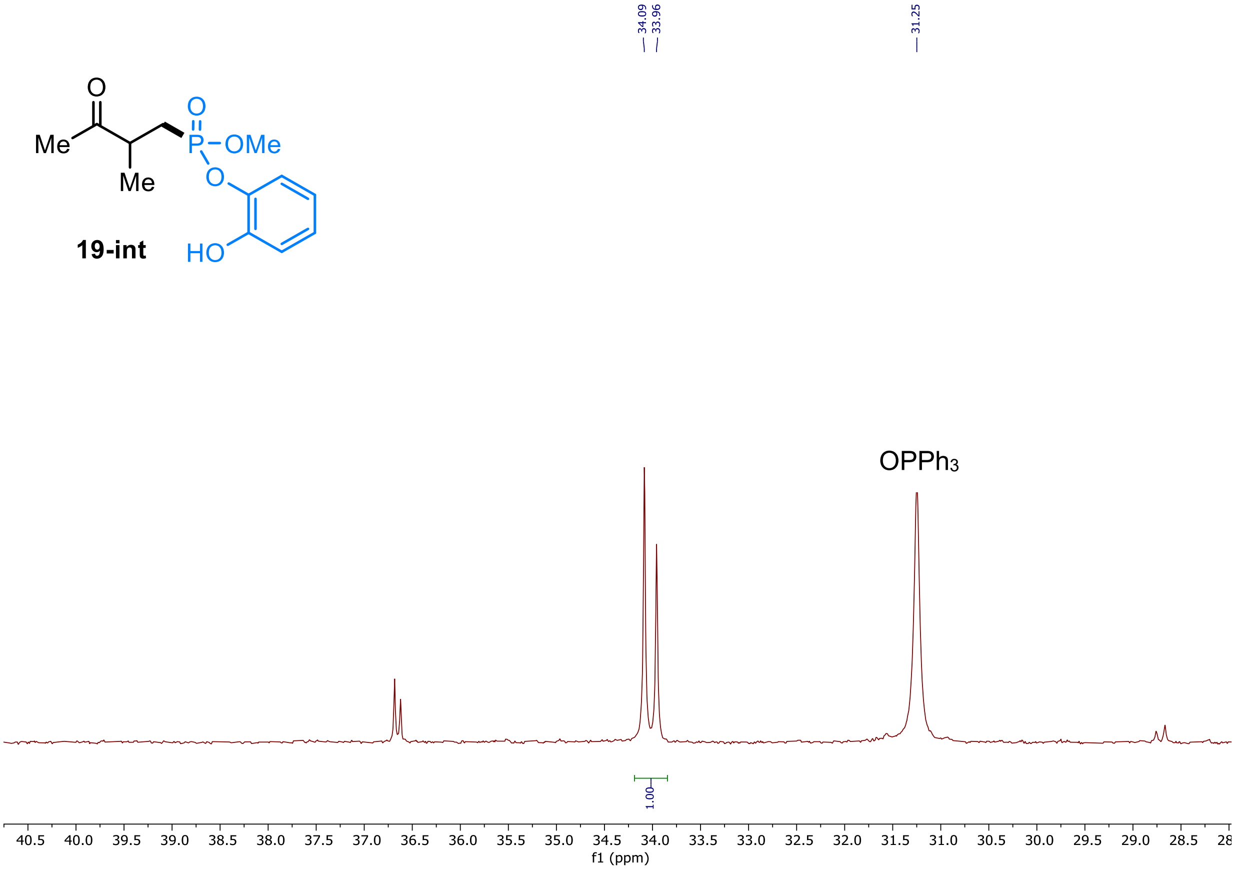

^{1}H NMR (400 MHz, $CDCl_3$) of **19** (*see procedure*)

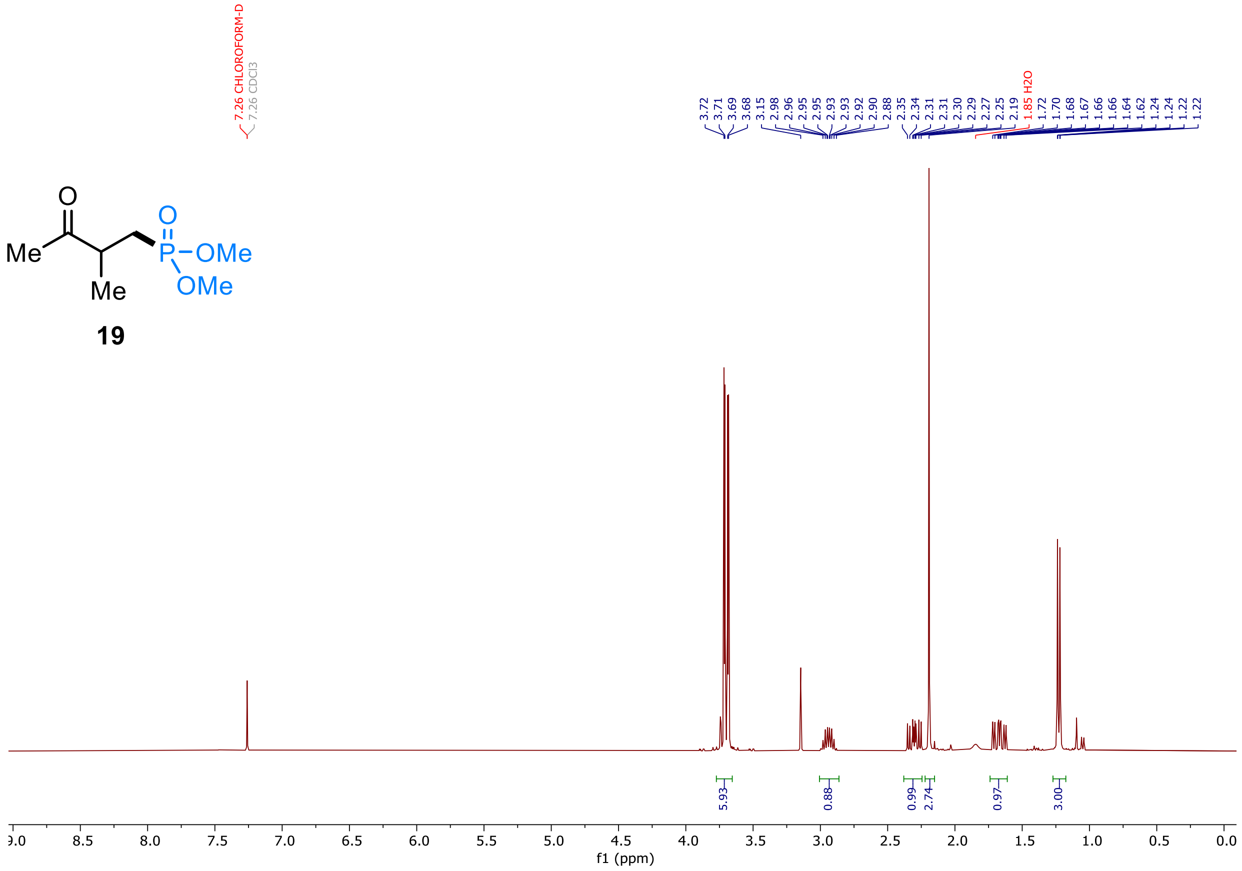

^{13}C NMR (126 MHz, $CDCl_3$) of **19**

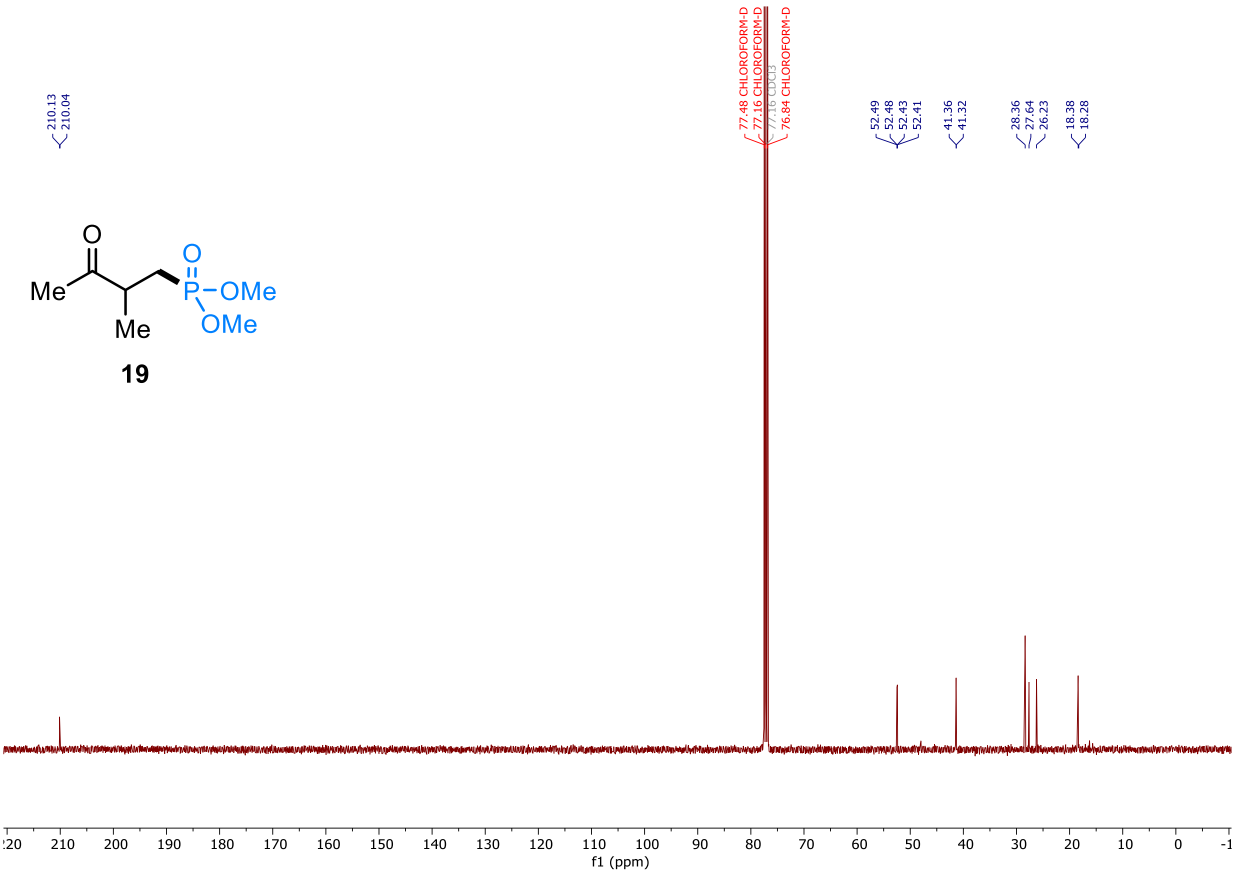

^{31}P NMR (165 MHz, $CDCl_3$) of **19**

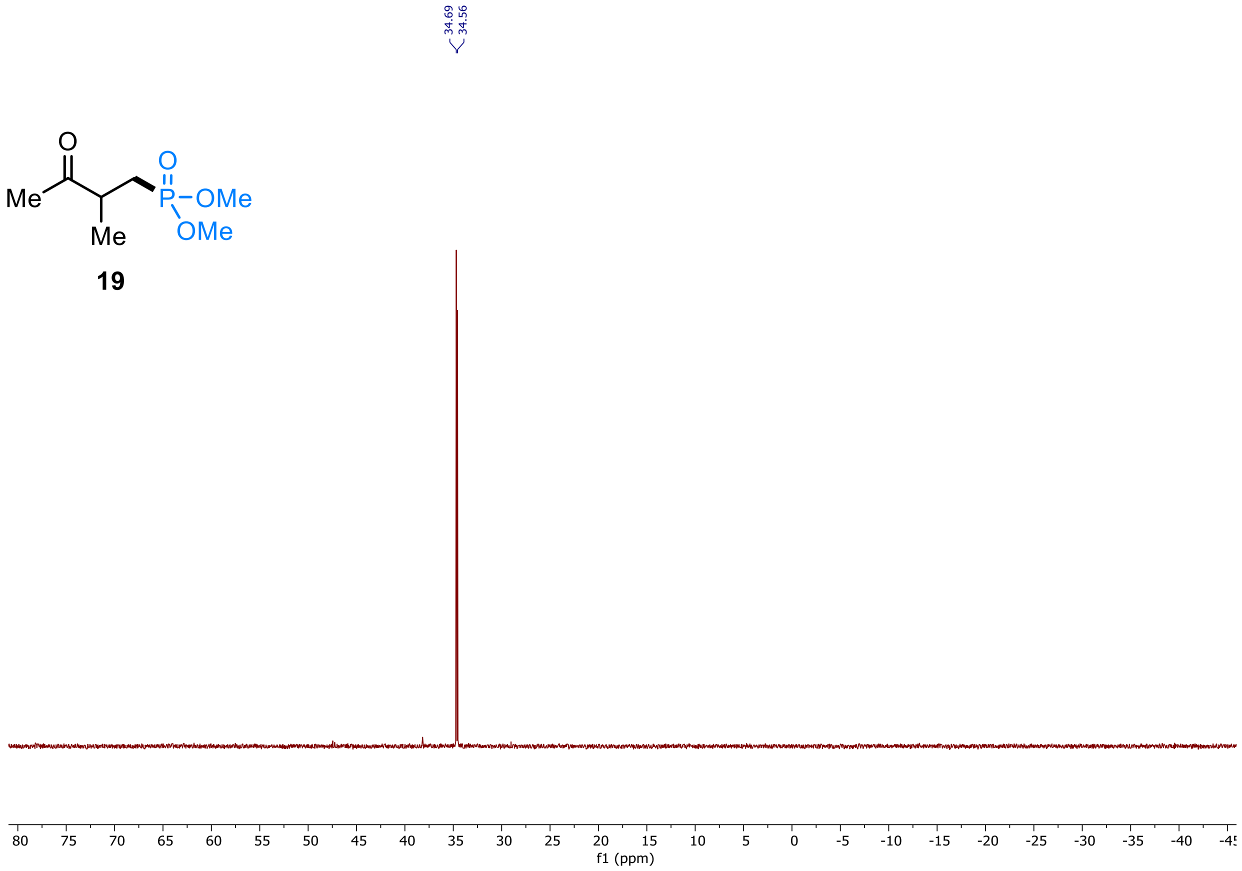

^{31}P NMR (162 MHz, $CDCl_3$) of crude **20-int** (*see procedure*)

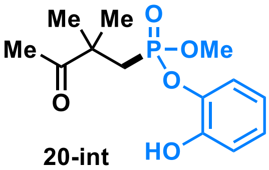

20-int

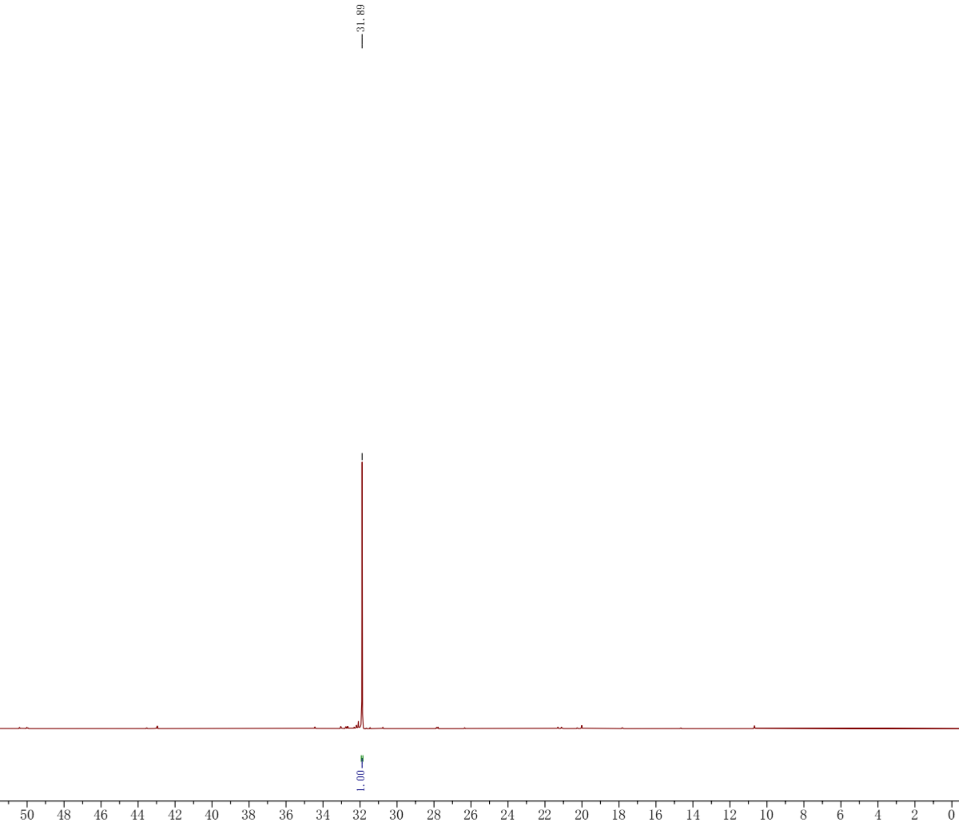

^{1}H NMR (400 MHz, $CDCl_3$) of **20** (*see procedure*)

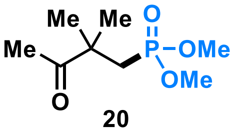

20

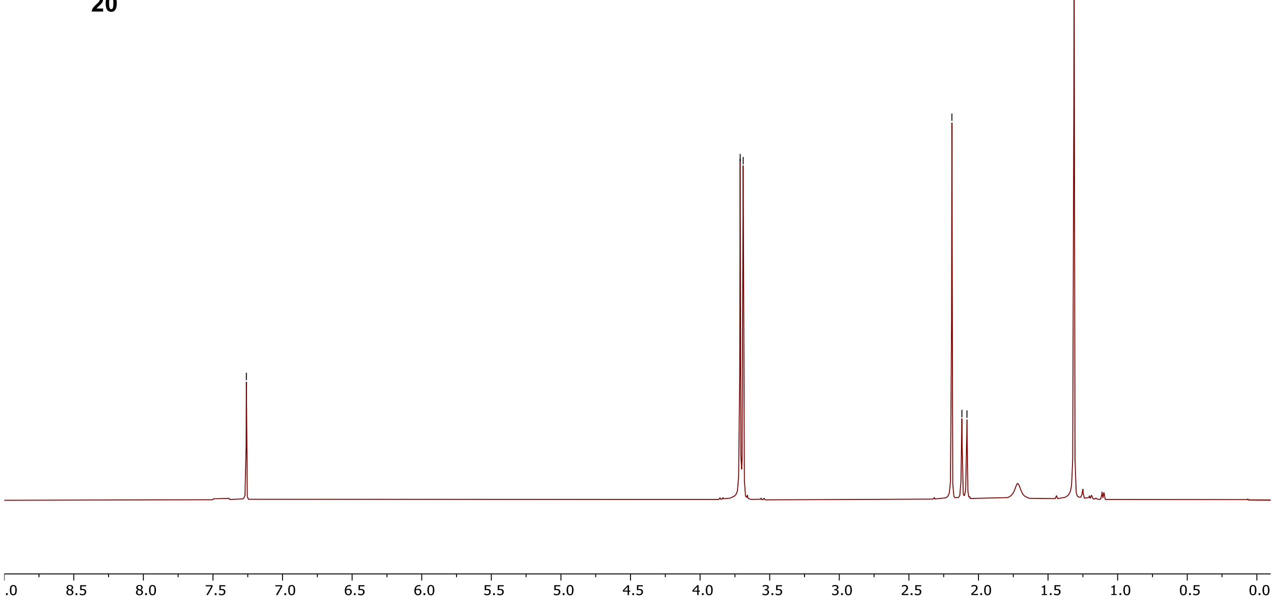

^{13}C NMR (126 MHz, $CDCl_3$) of **20**

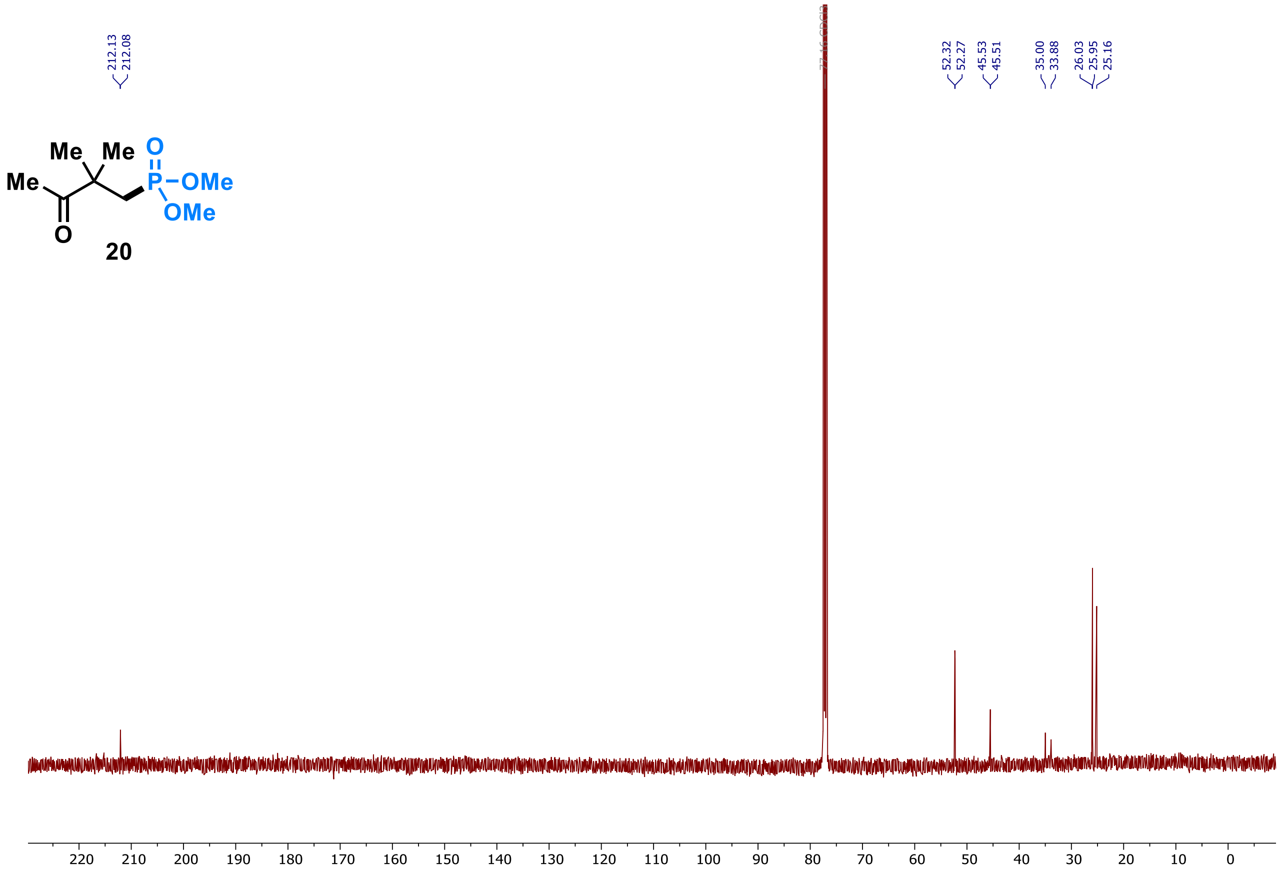

^{31}P NMR (165 MHz, $CDCl_3$) of **20**

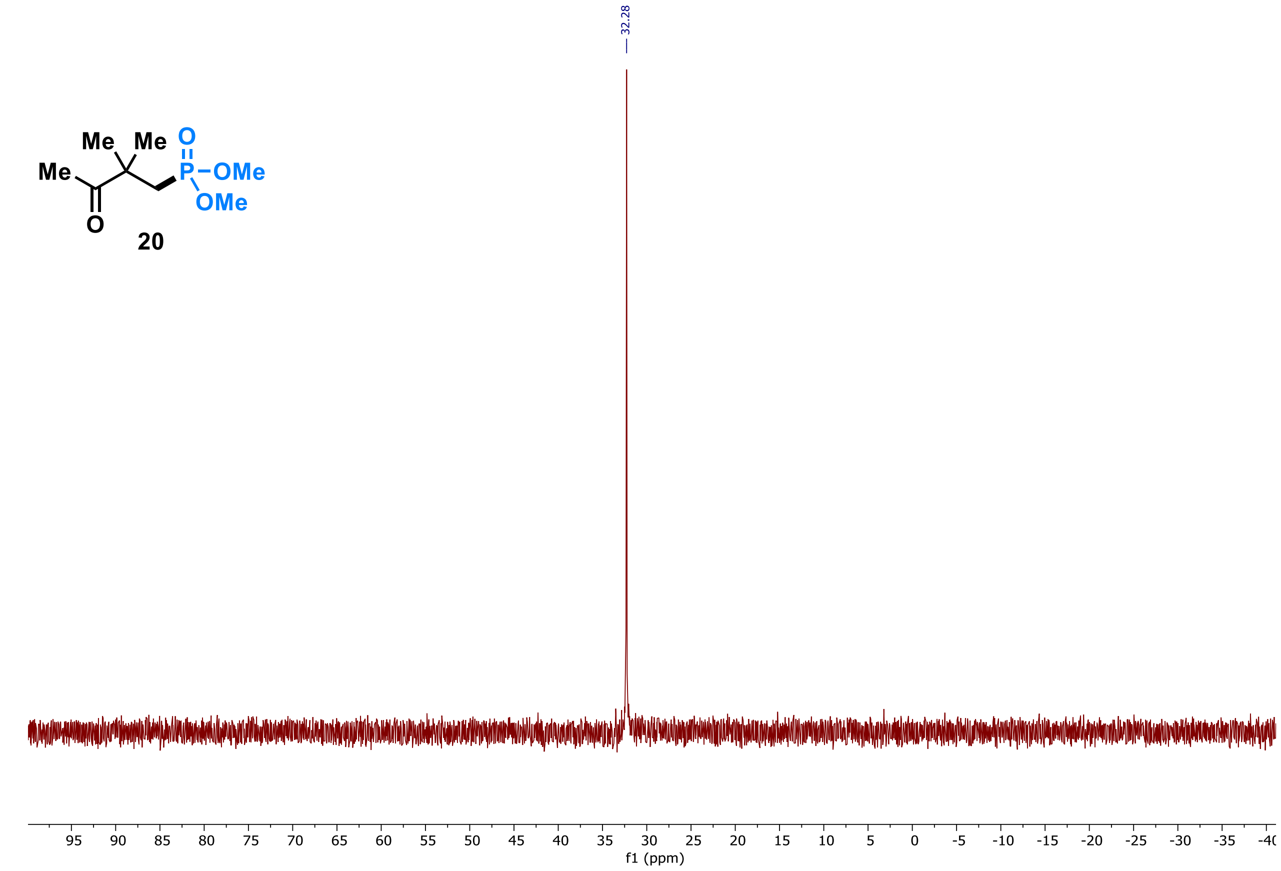

^{31}P NMR (162 MHz, CDCl$_3$) of crude **21-int** (see procedure)

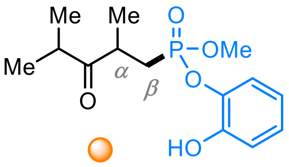

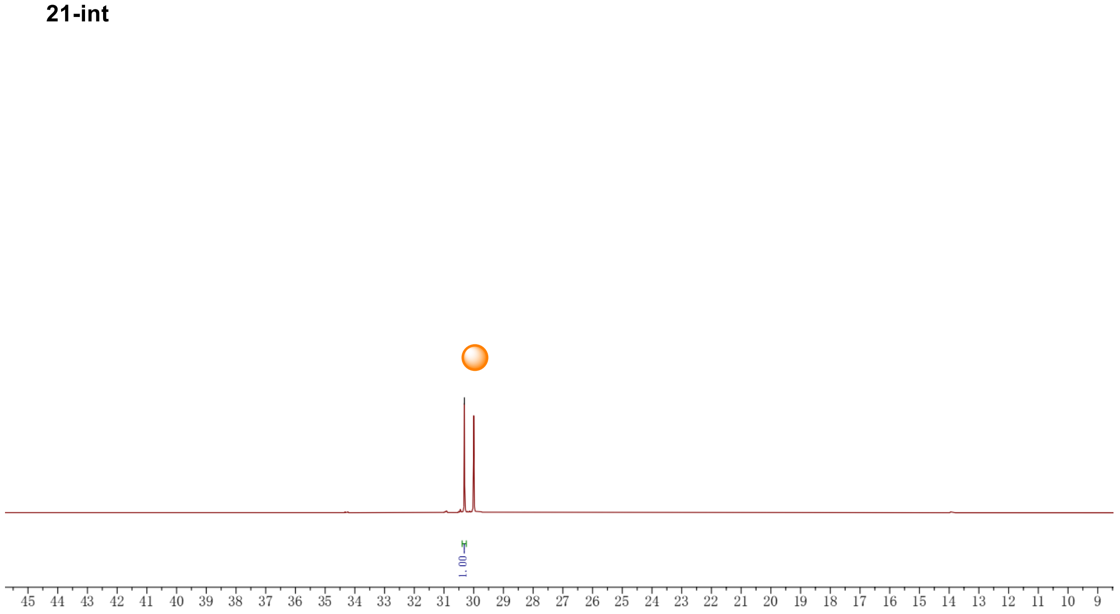

^{1}H NMR (400 MHz, CDCl$_3$) of **21** (see procedure)

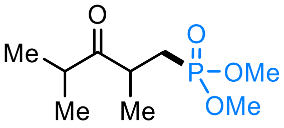

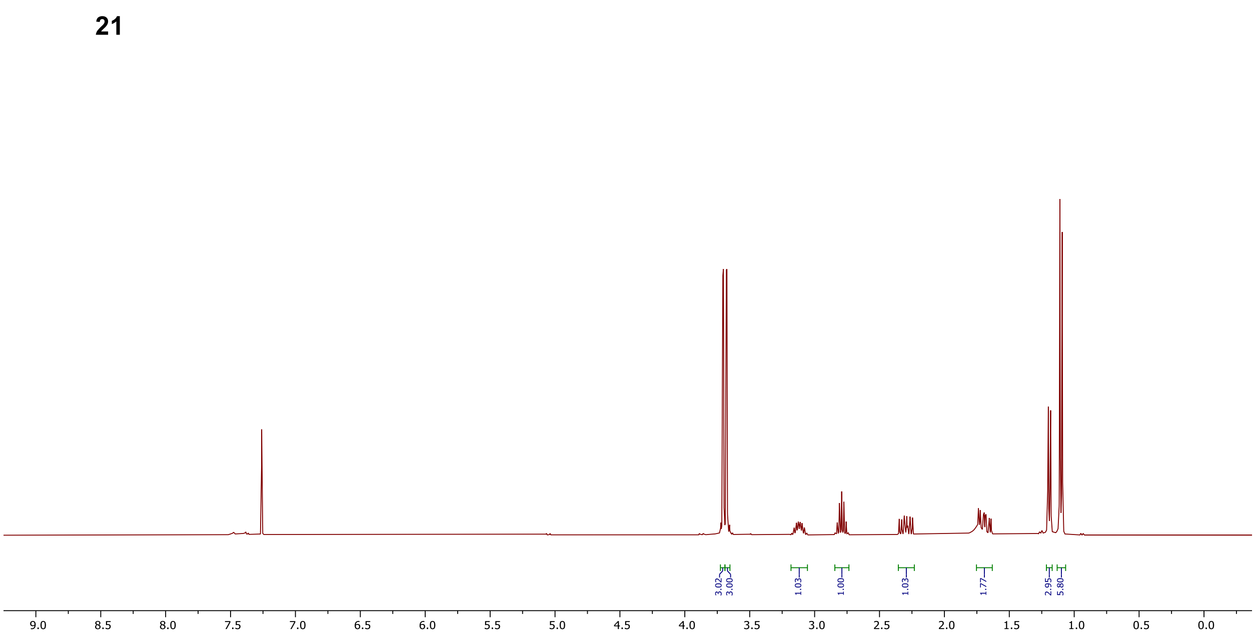

^{13}C NMR (126 MHz, $CDCl_3$) of **21**

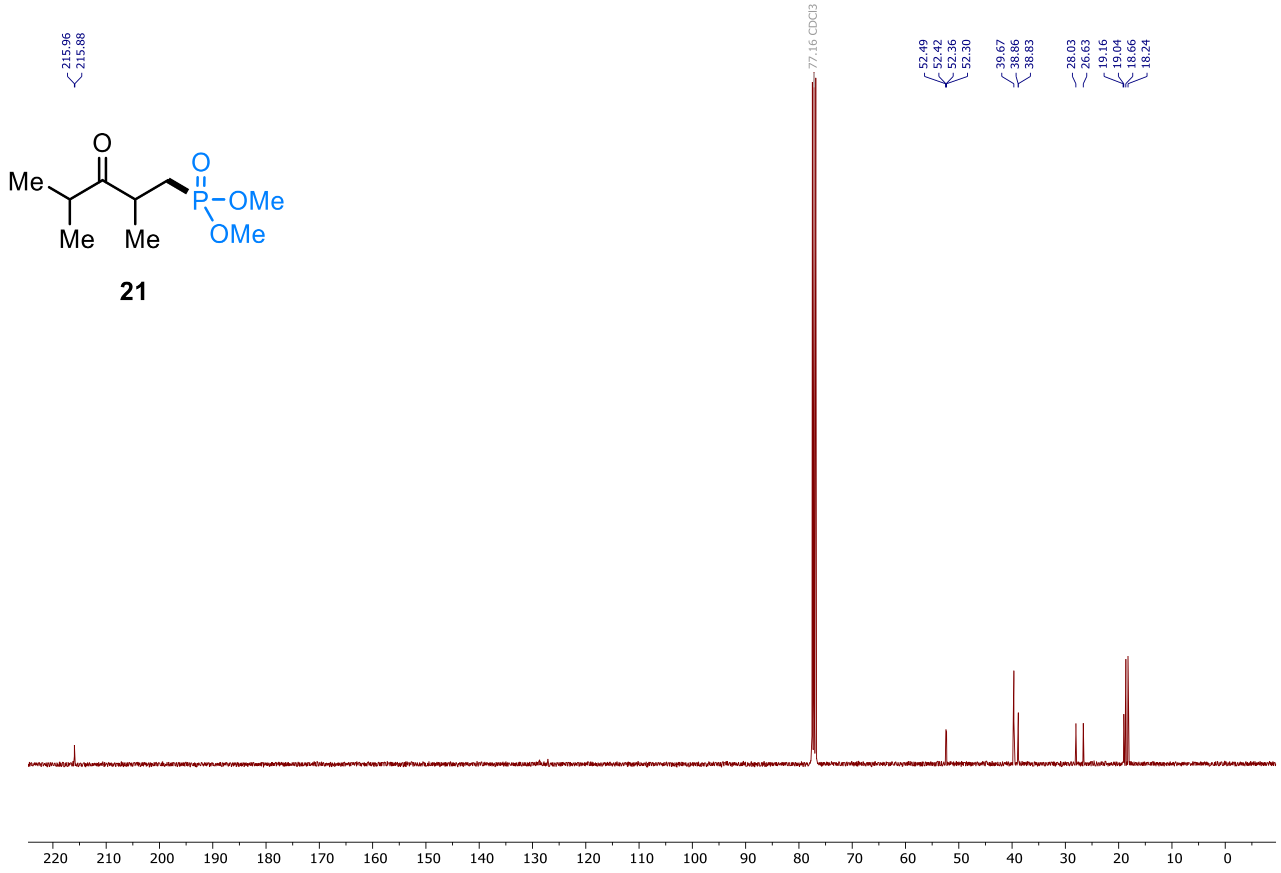

^{31}P NMR (165 MHz, $CDCl_3$) of **21**

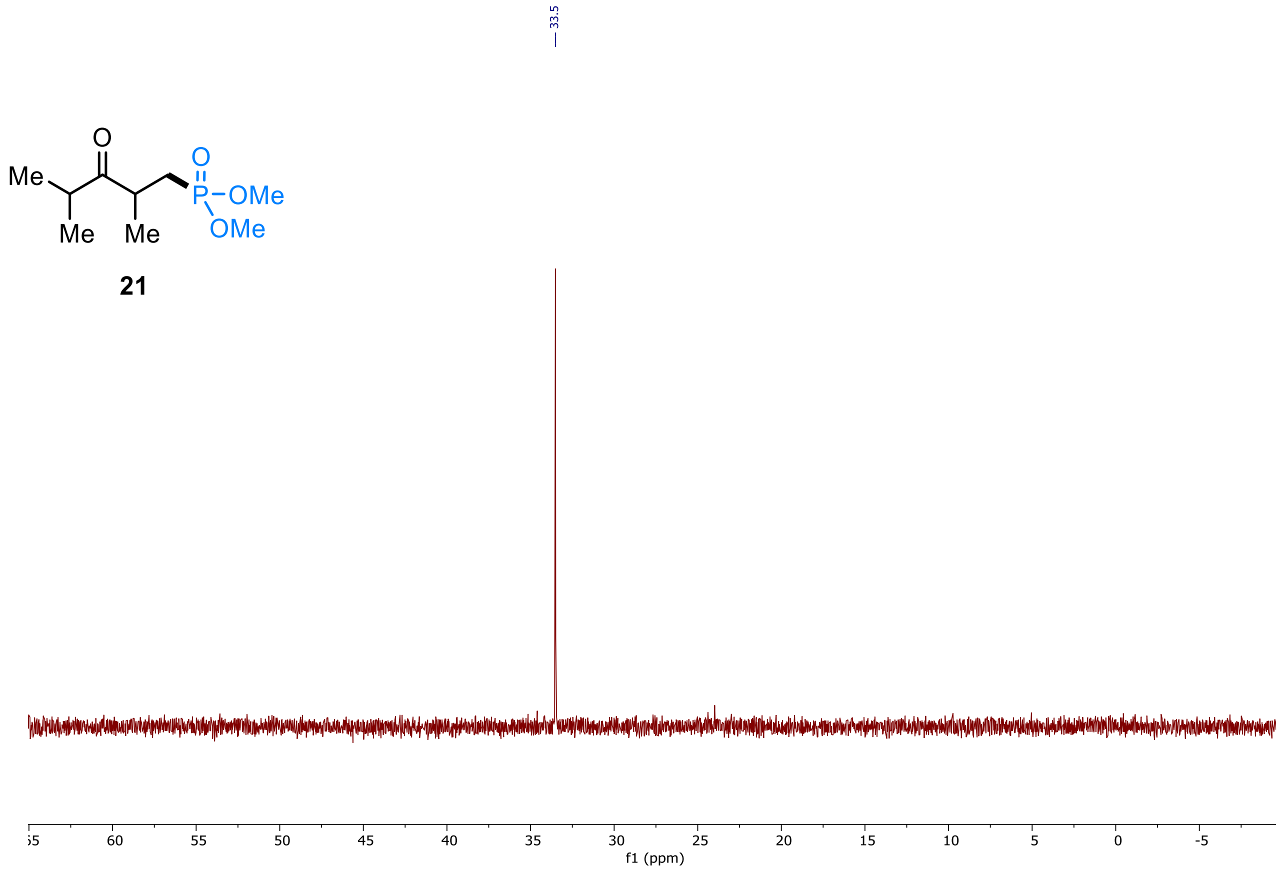

^{31}P NMR (162 MHz, $CDCl_3$) of crude **22-int** (*see procedure*)

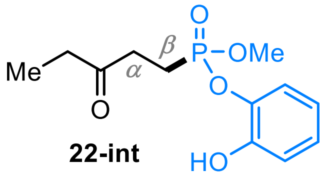

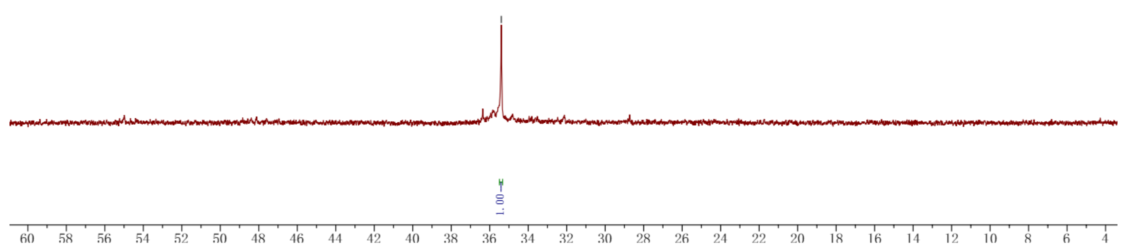

^{1}H NMR (400 MHz, $CDCl_3$) of **22** (*see procedure*)

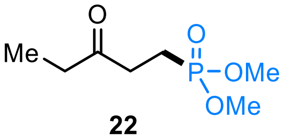

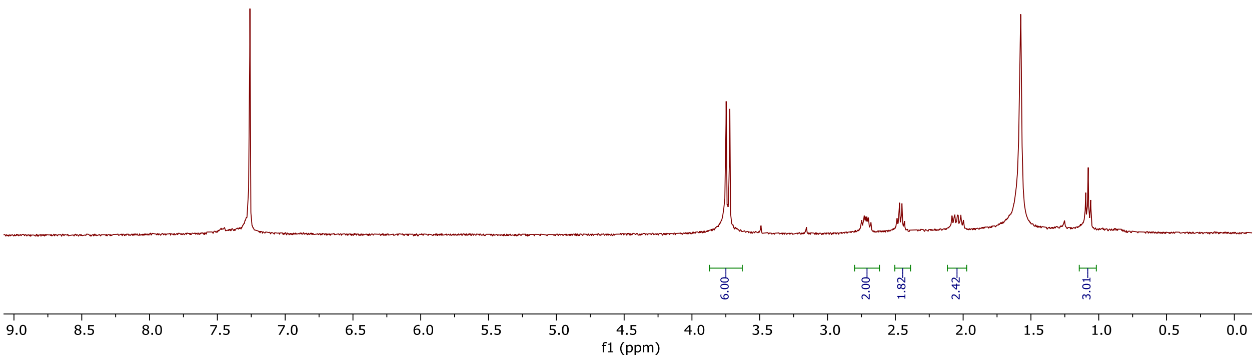

^{13}C NMR (126 MHz, $CDCl_3$) of **22**

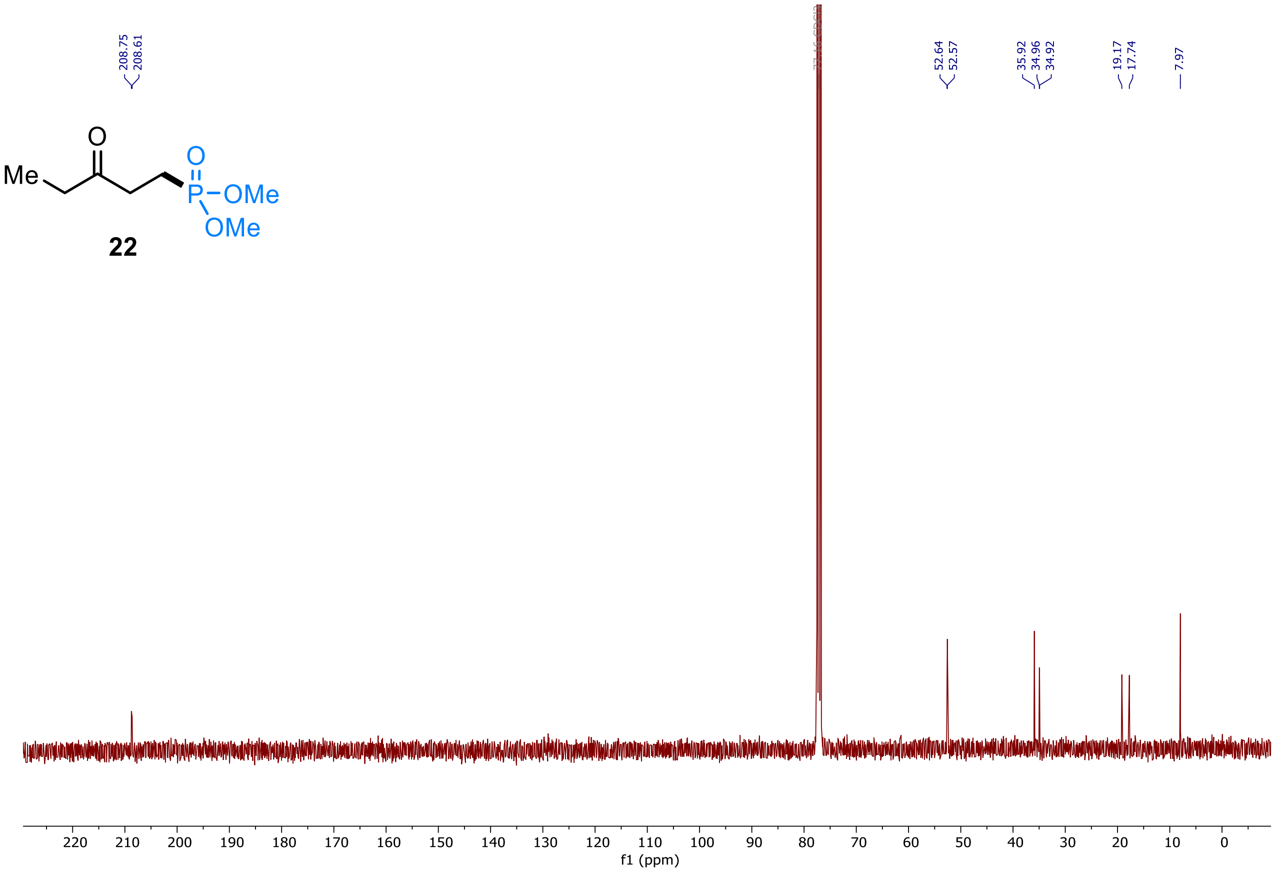

22

^{31}P NMR (165 MHz, $CDCl_3$) of **22**

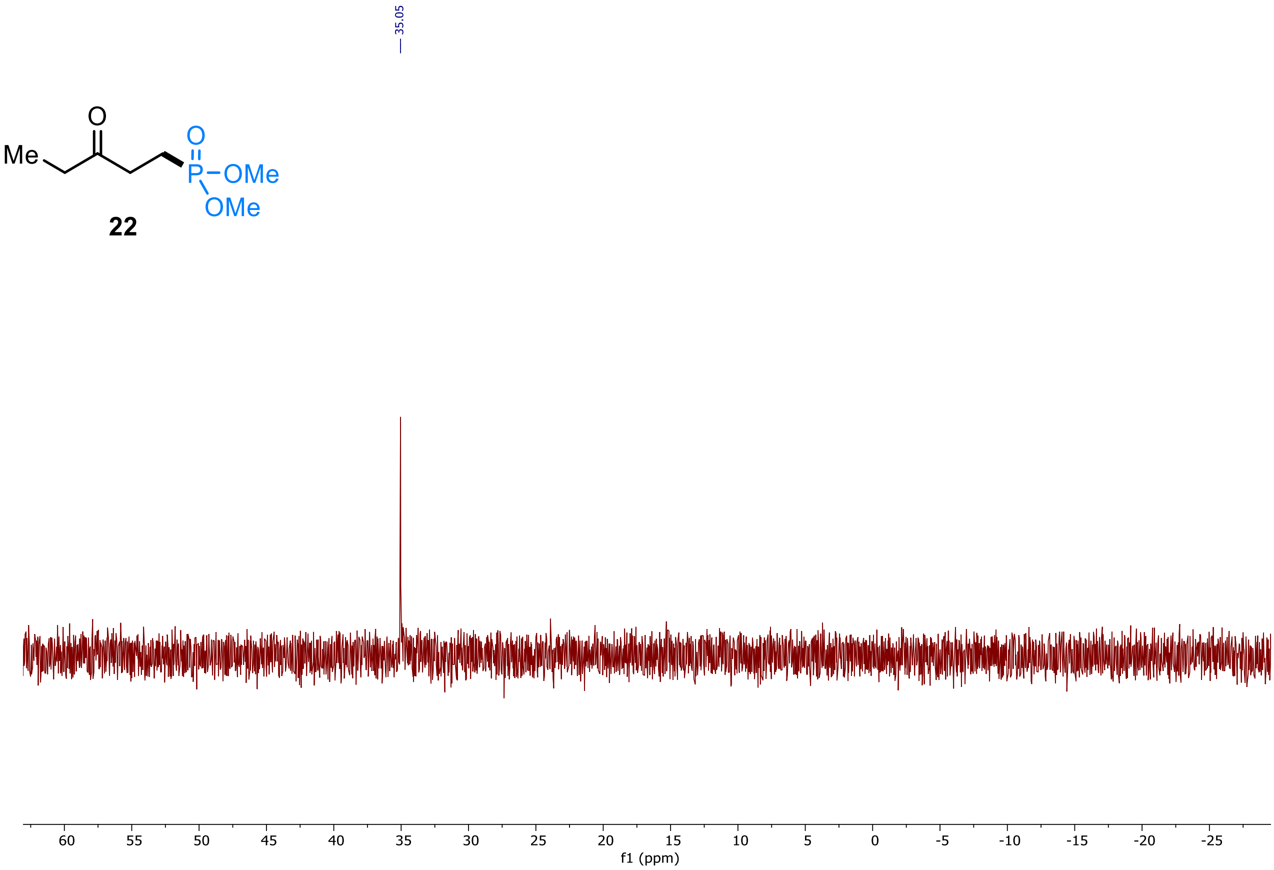

22

^{31}P NMR (162 MHz, $CDCl_3$) of crude **23-int** (see procedure)

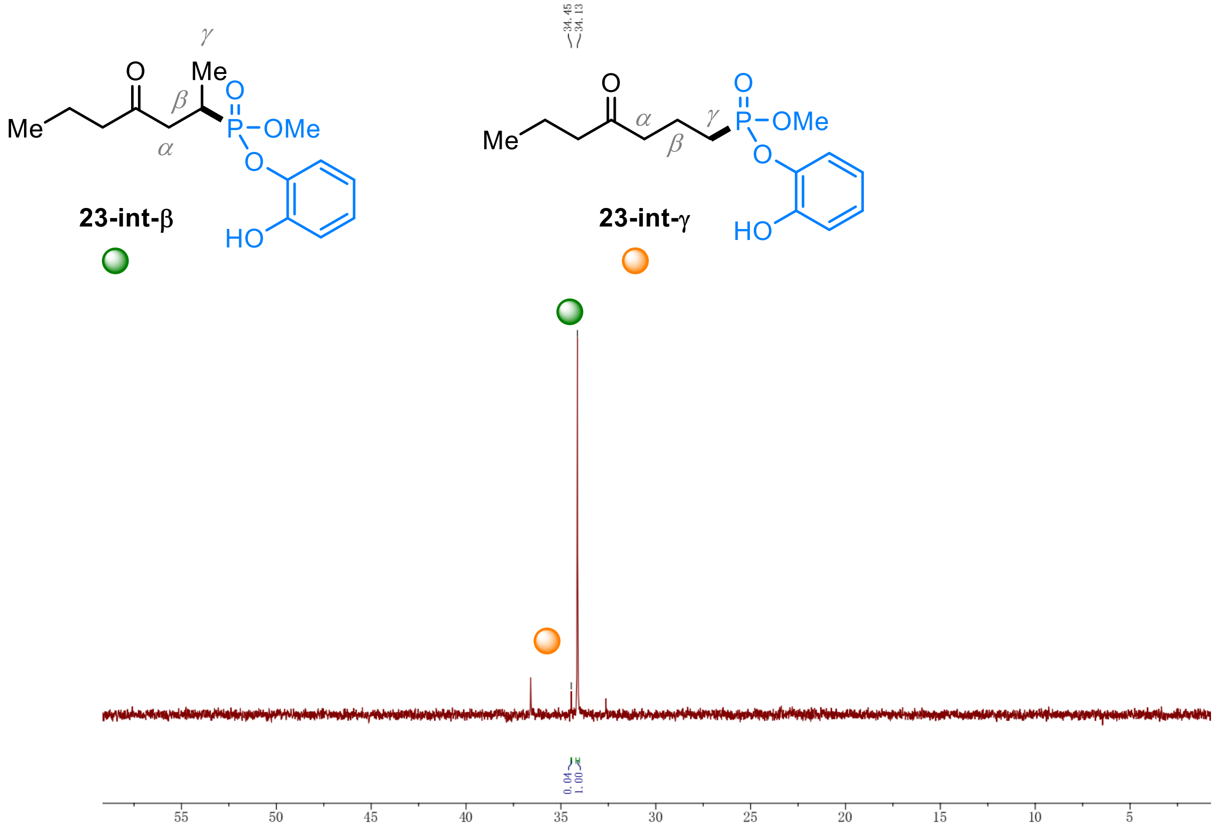

^{1}H NMR (400 MHz, $CDCl_3$) of **23** (see procedure)

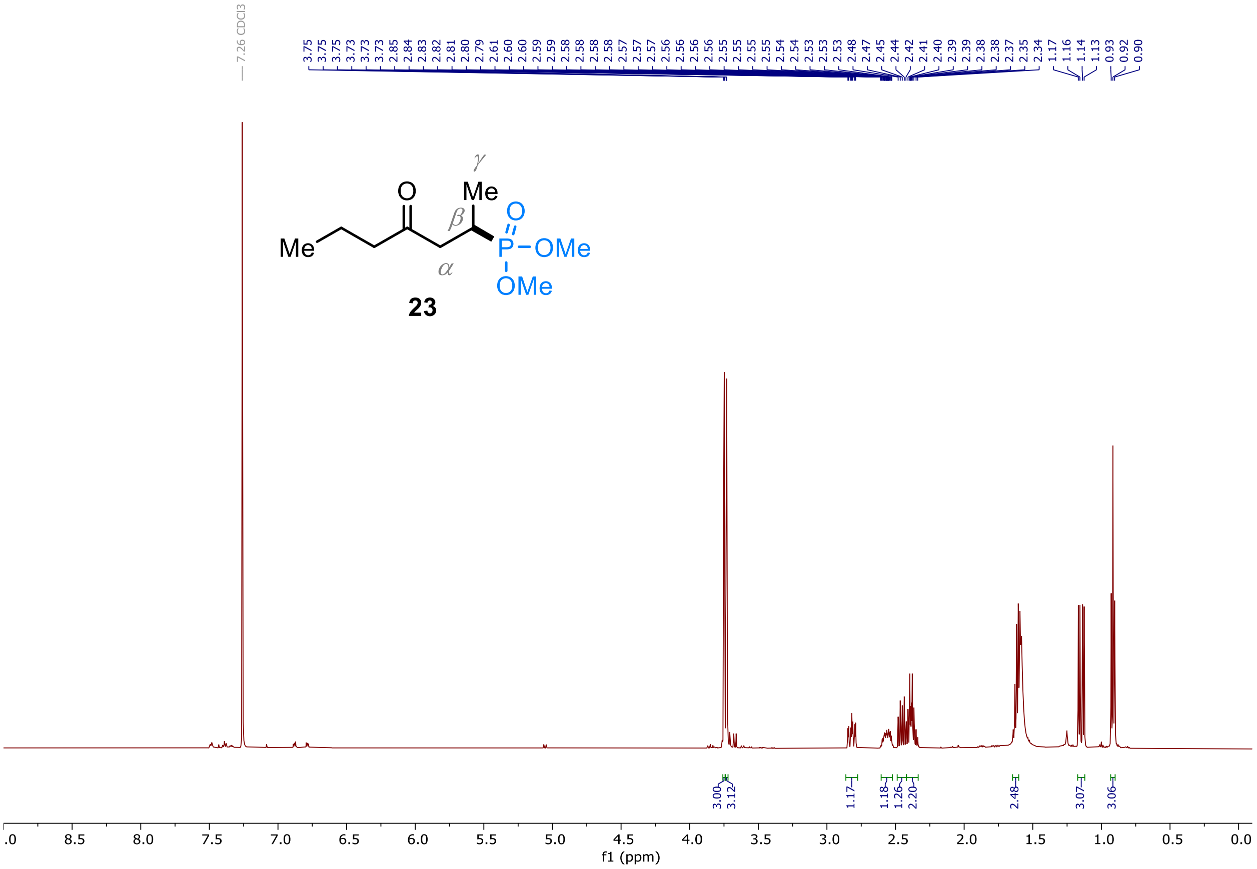

^{13}C NMR (126 MHz, CDCl$_3$) of **23**

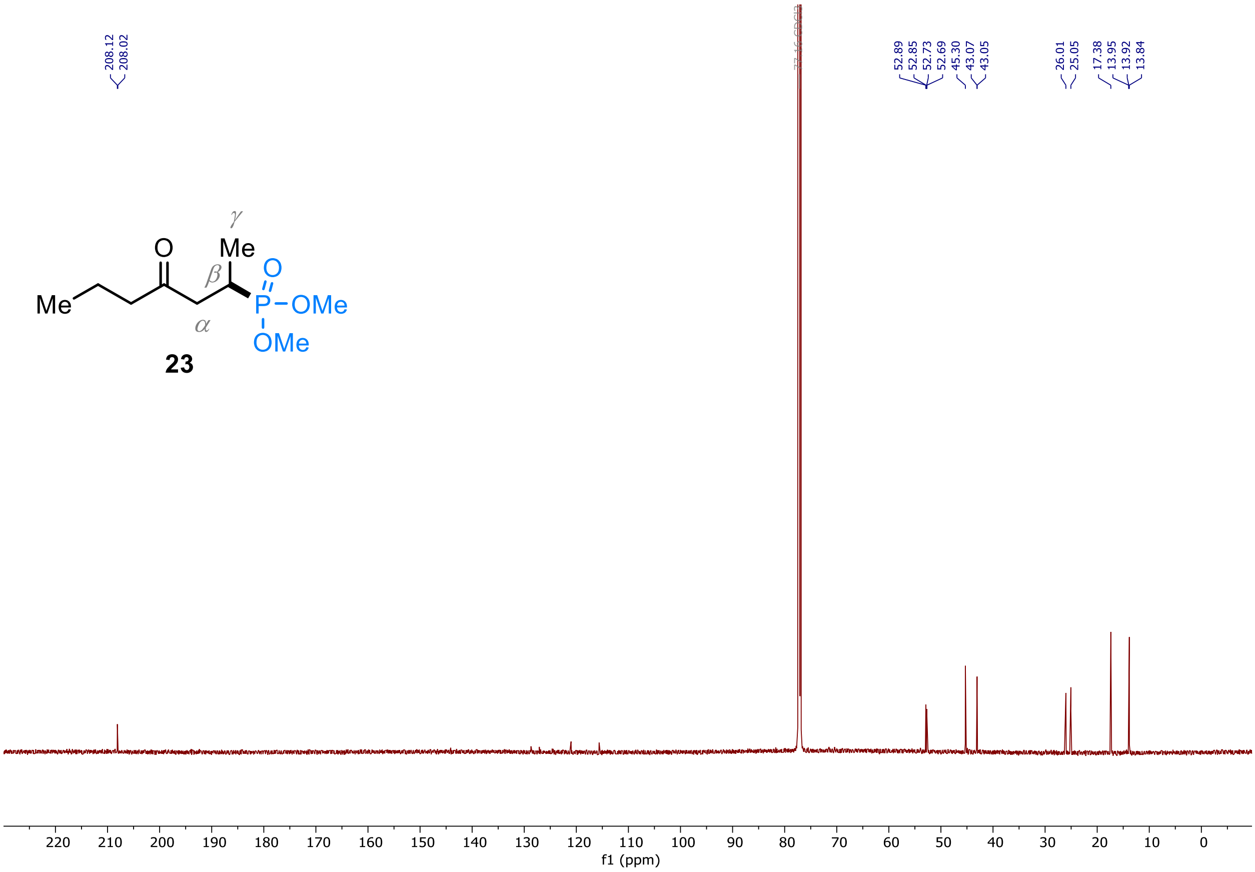

^{31}P NMR (165 MHz, CDCl$_3$) of **23**

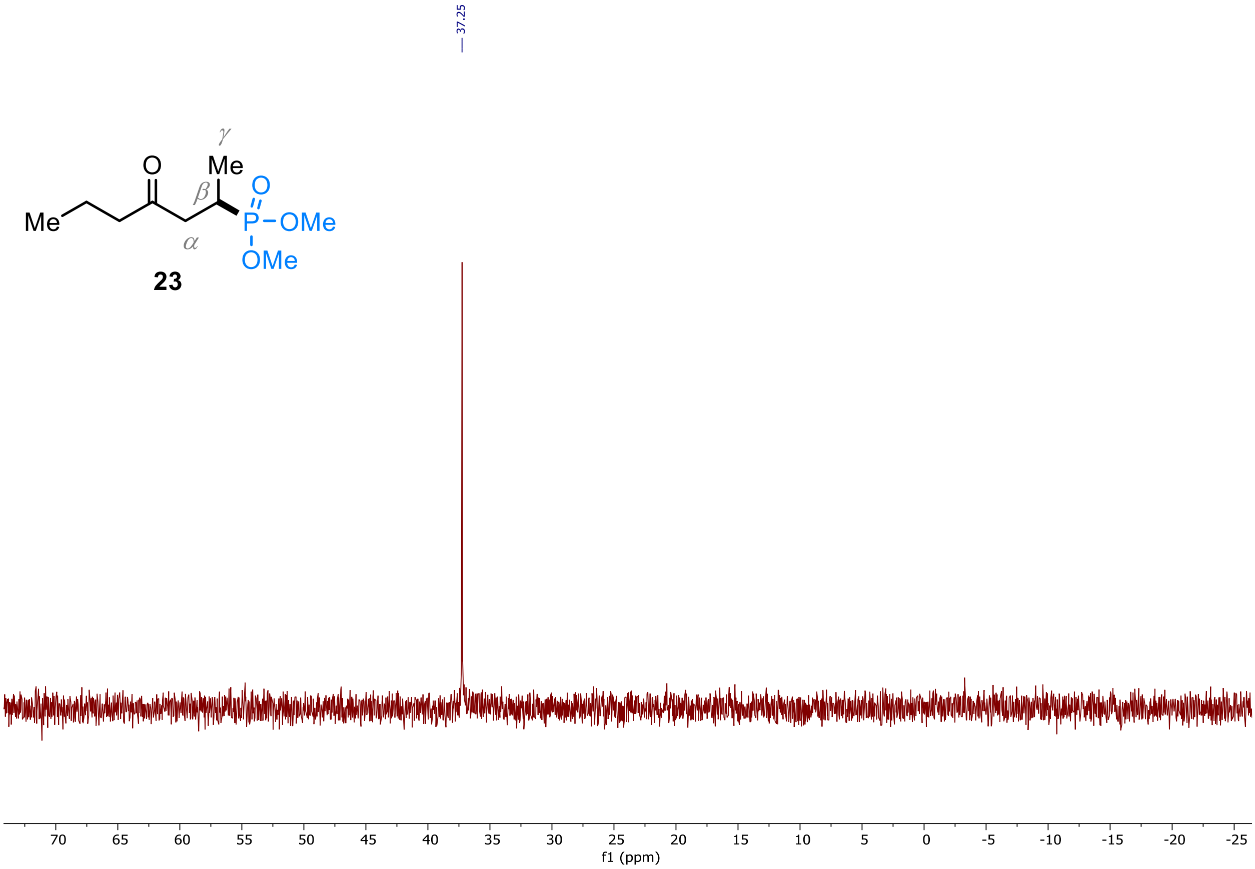

^{31}P NMR of crude reaction mixture of **24** (*see procedure*)

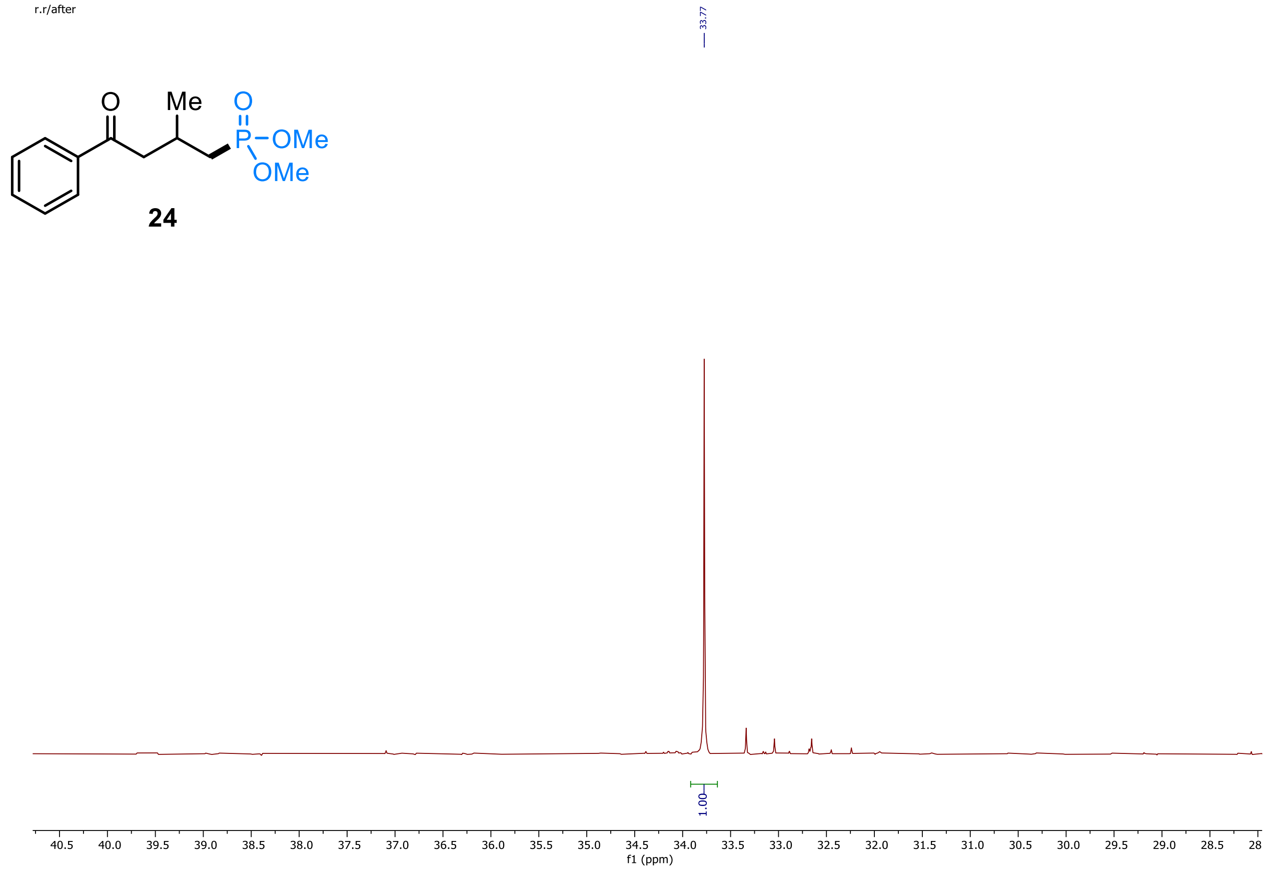

^{1}H NMR (400 MHz, $CDCl_3$) of **24** (*see procedure*)

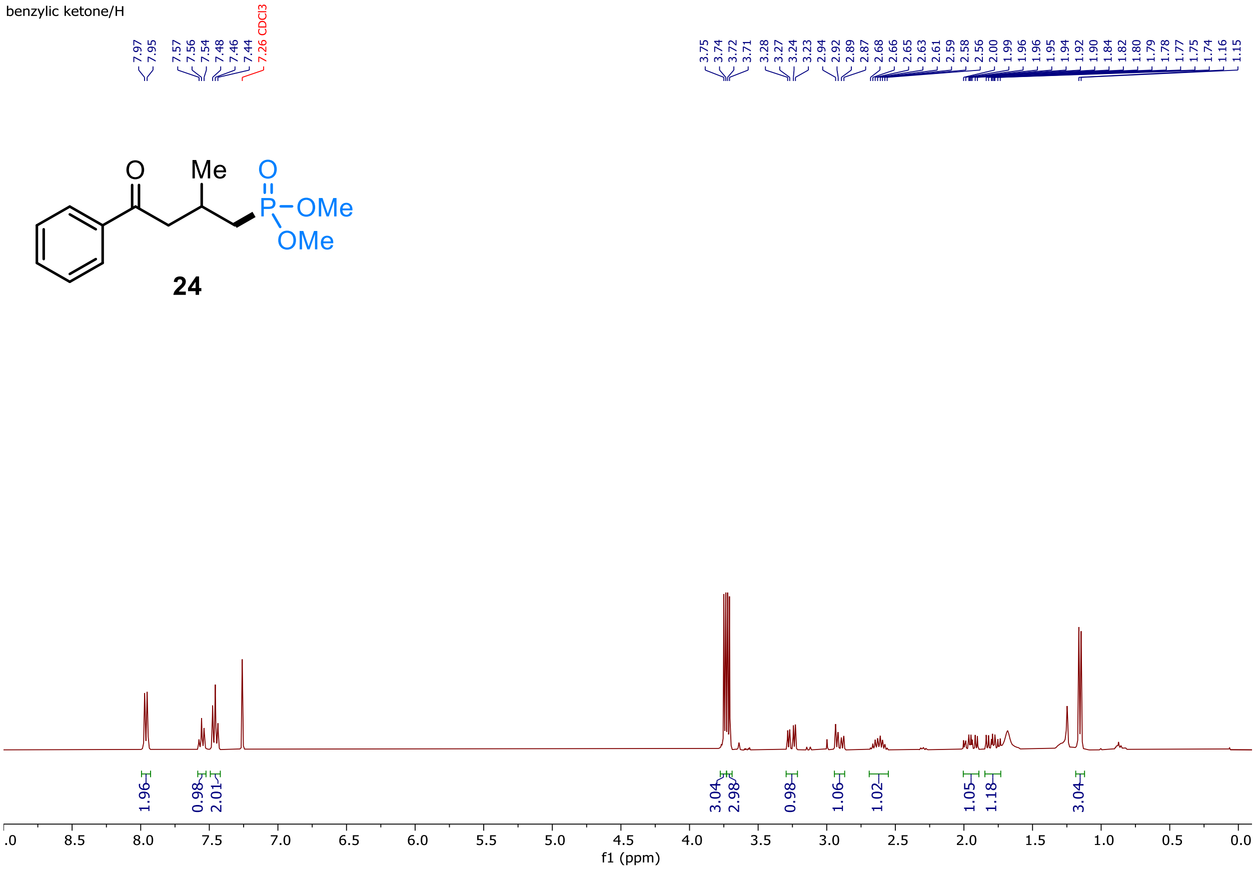

^{13}C NMR (101 MHz, $CDCl_3$) of **24**

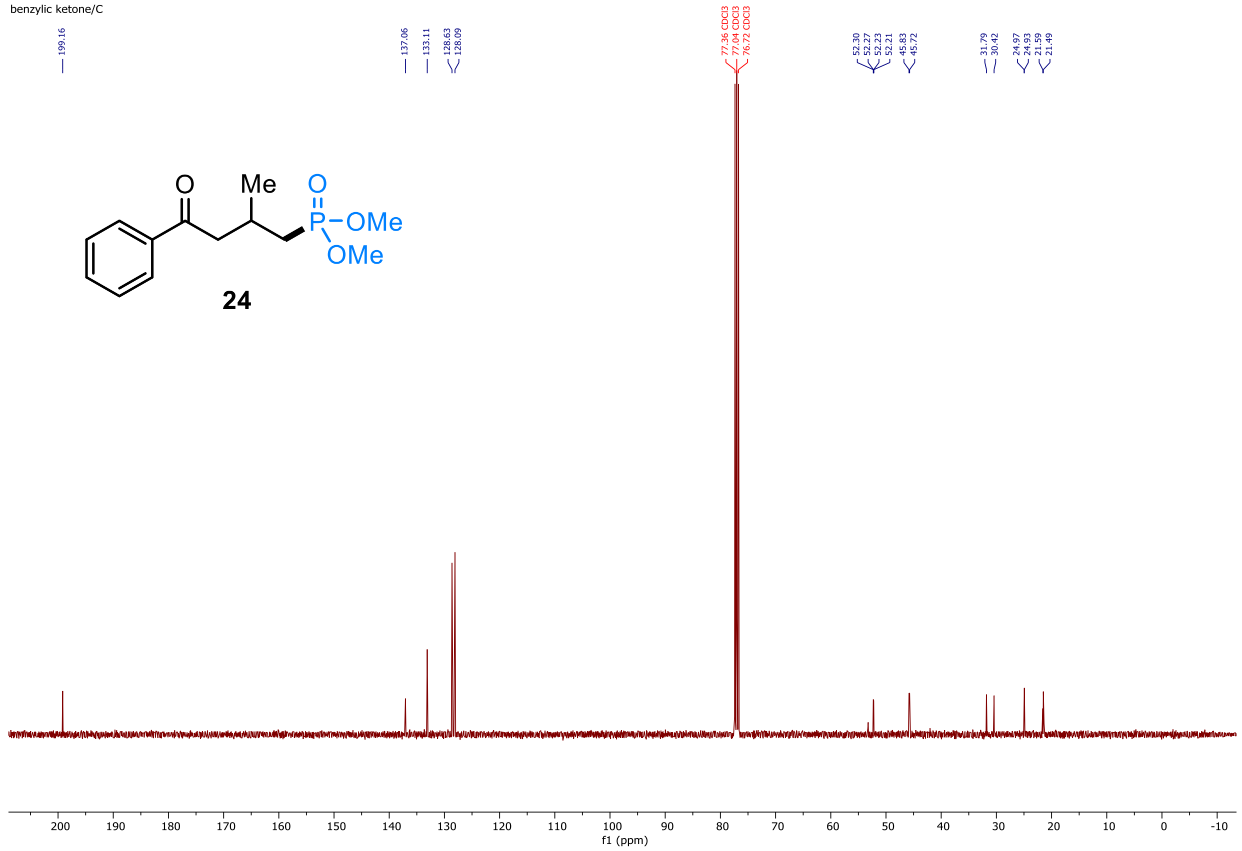

^{31}P NMR (162 MHz, $CDCl_3$) of **24**

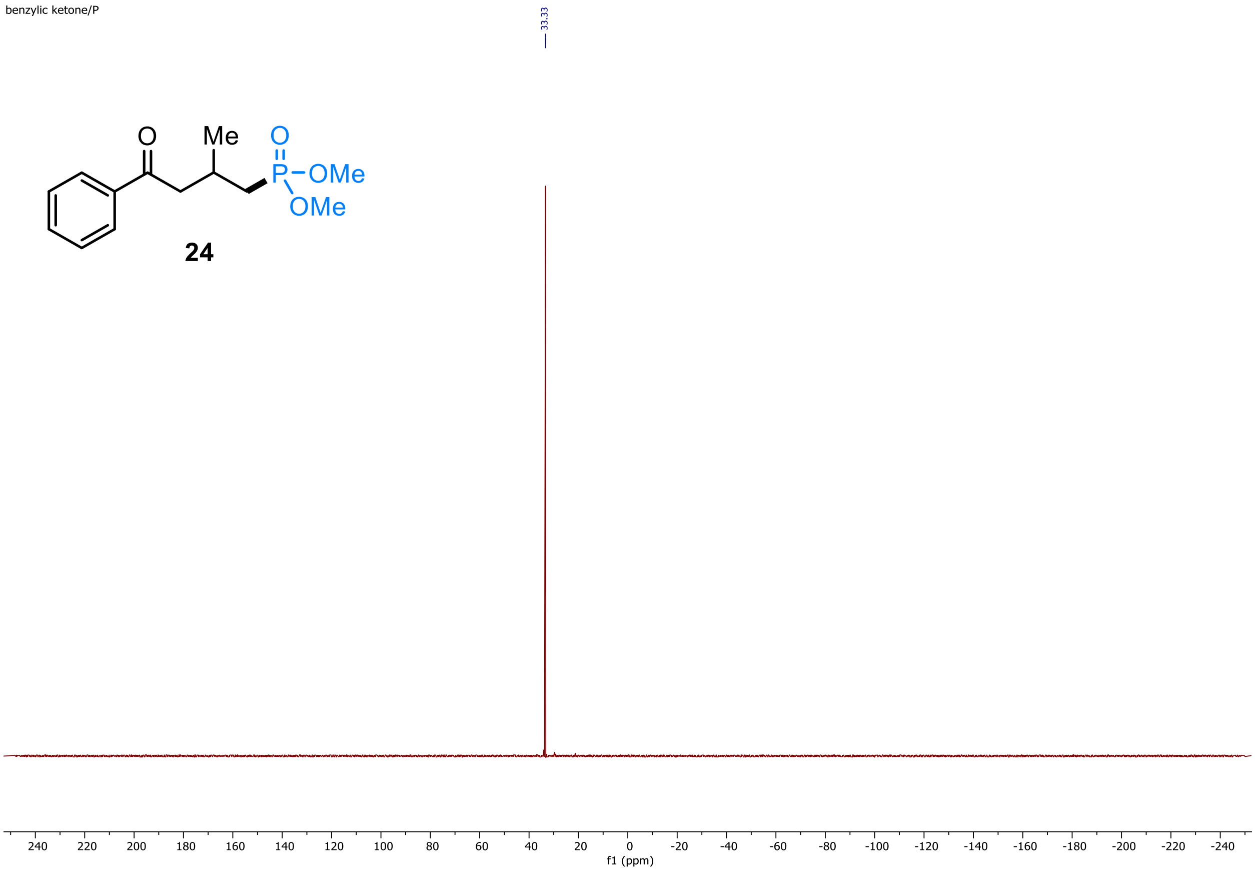

^{31}P NMR of crude reaction mixture of **25-int** (*see procedure*)

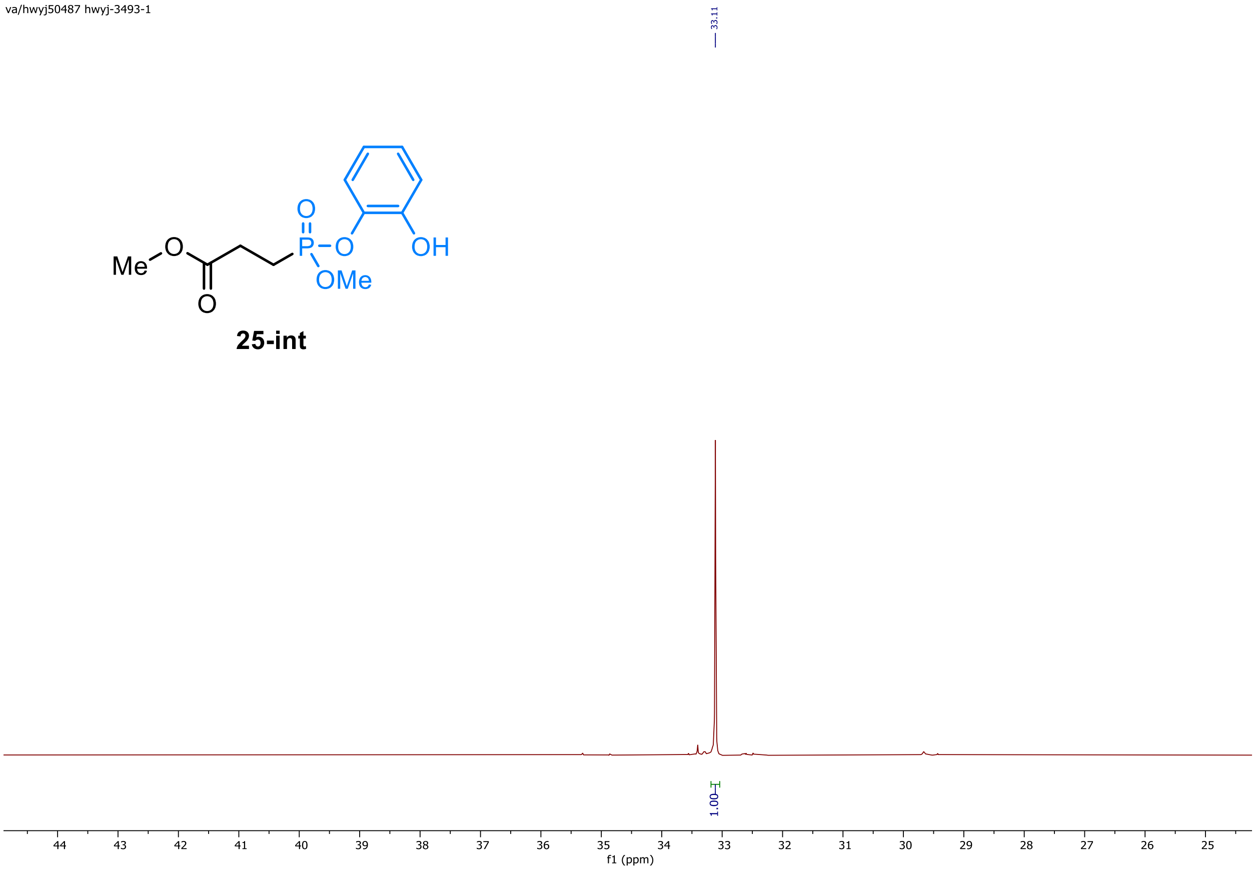

^{1}H NMR (400 MHz, $CDCl_3$) of **25** (*see procedure*)

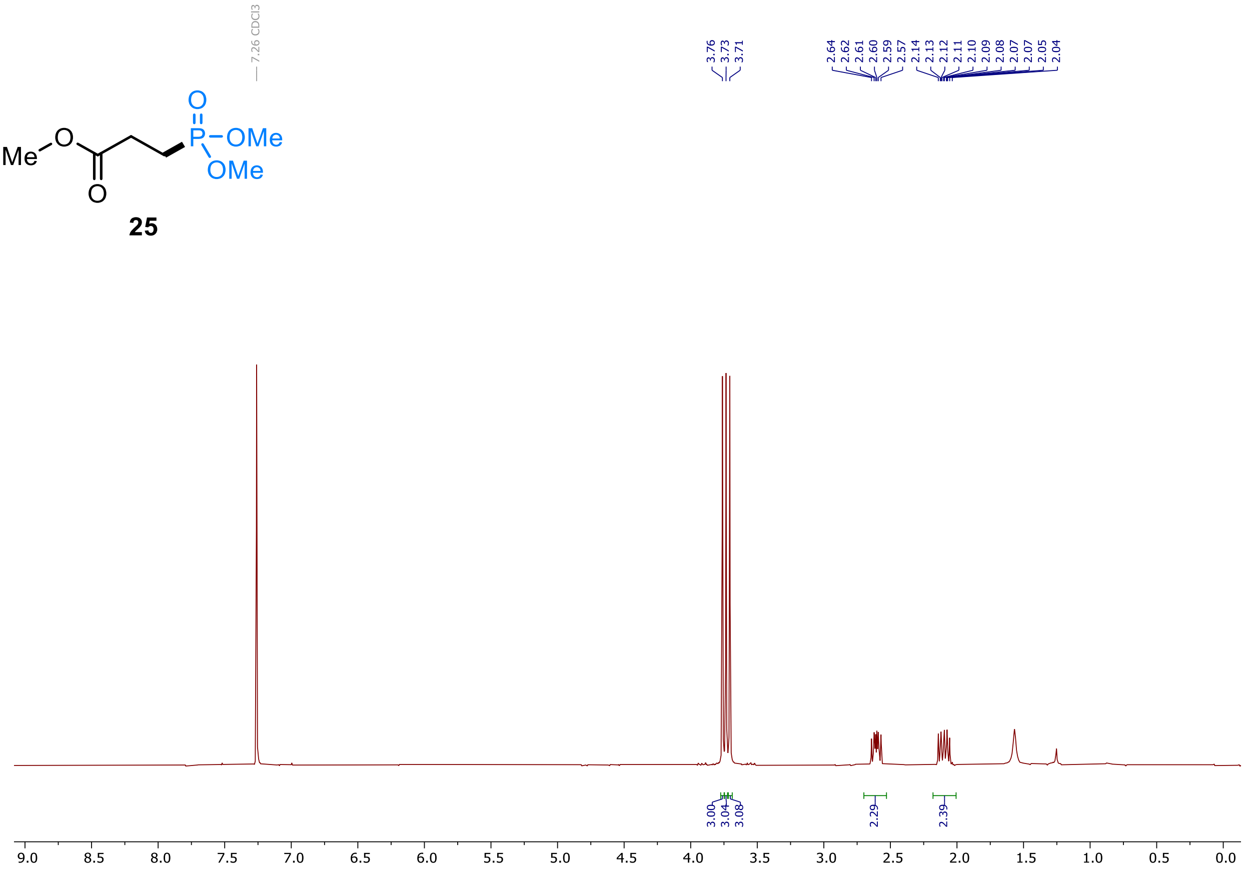

^{13}C NMR (101 MHz, CDCl$_3$) of **25**

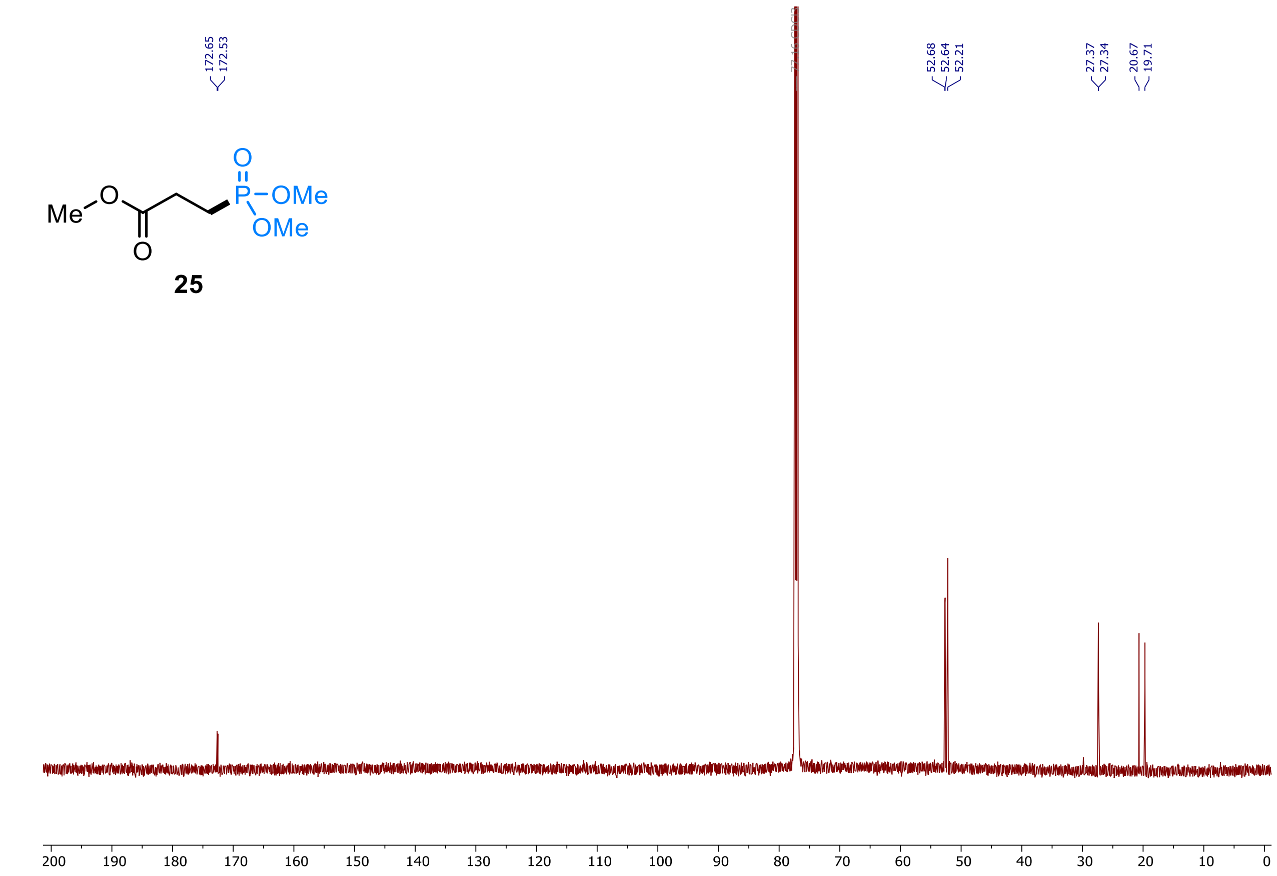

^{31}P NMR (165 MHz, CDCl$_3$) of **25**

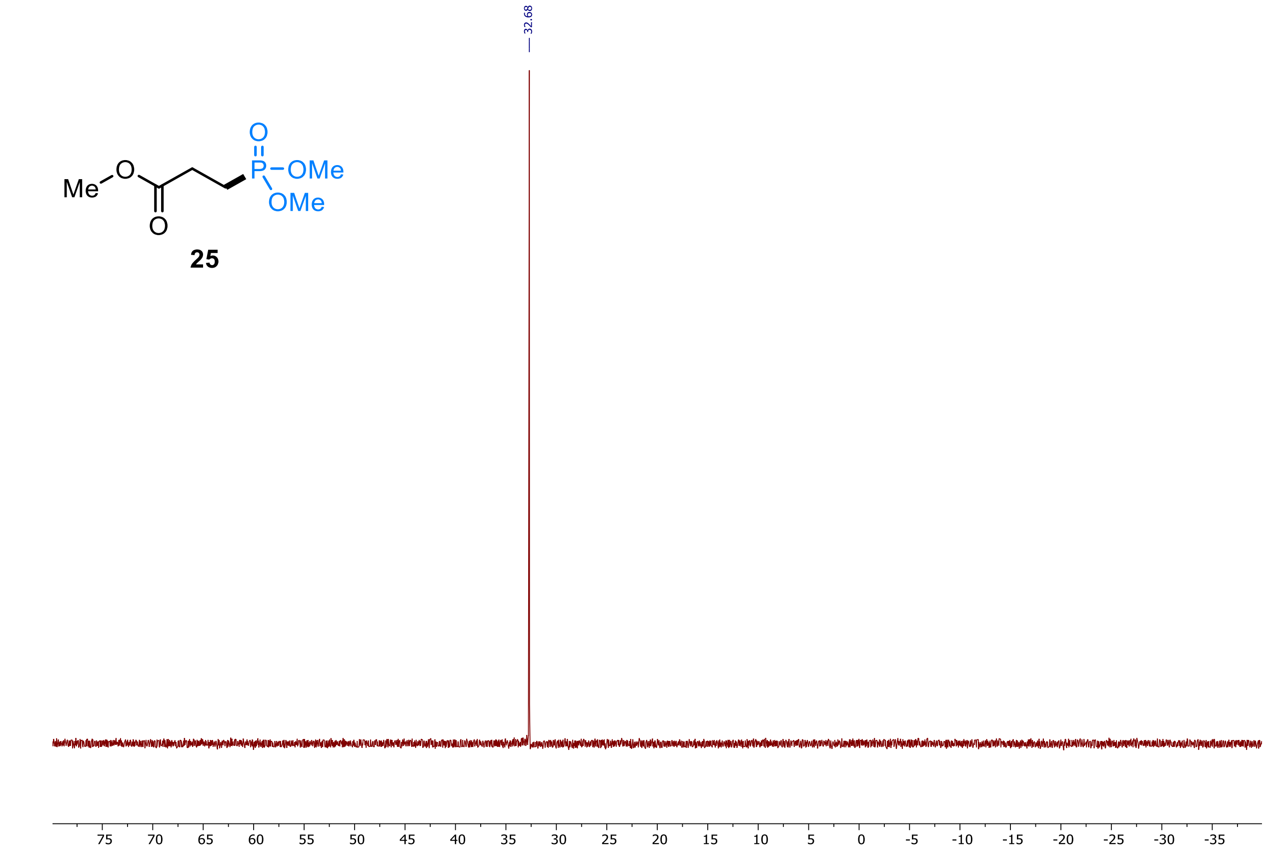

^{31}P NMR of crude reaction mixture of **26-int** (<u>*see procedure*</u>)

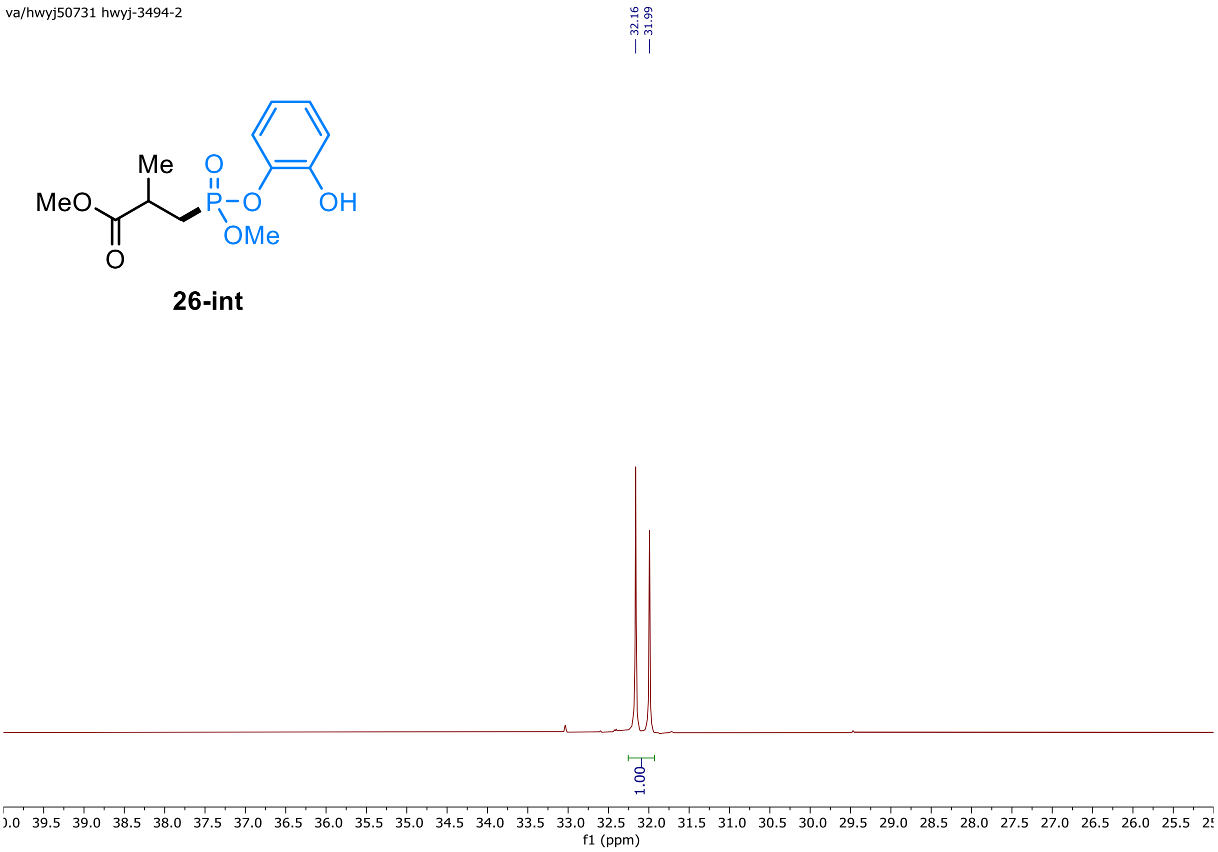

26-int

^{1}H NMR (400 MHz, $CDCl_3$) of **26** (<u>*see procedure*</u>)

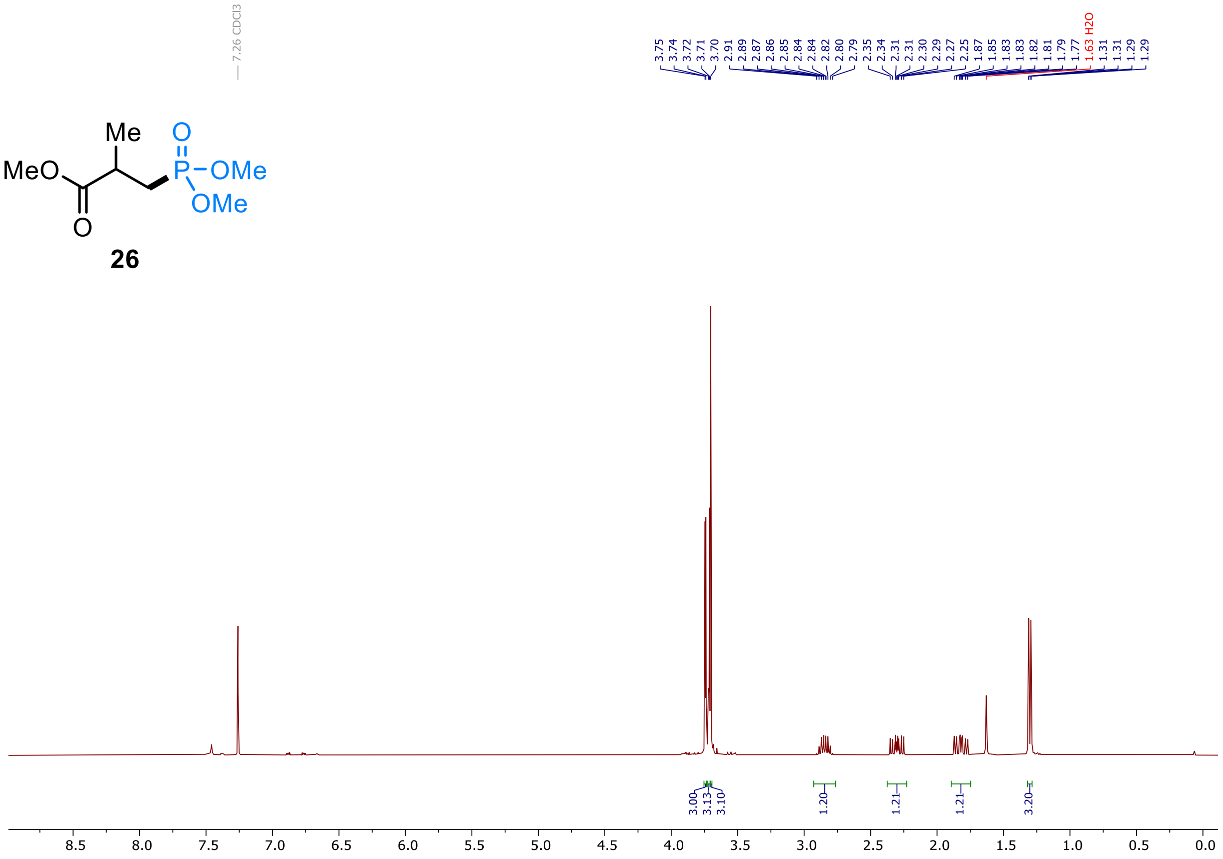

26

^{13}C NMR (101 MHz, CDCl$_3$) of **26**

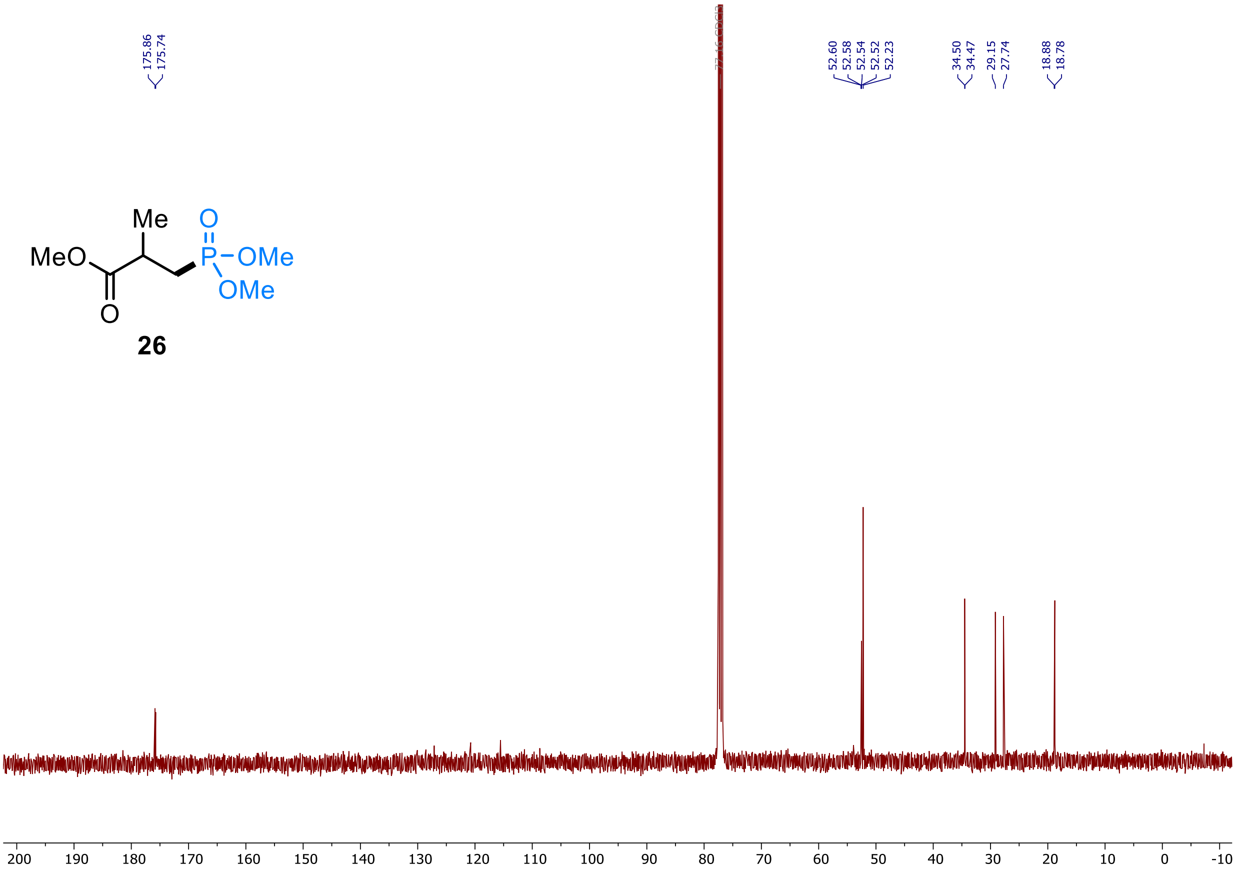

26

^{31}P NMR (400 MHz, CDCl$_3$) of **26**

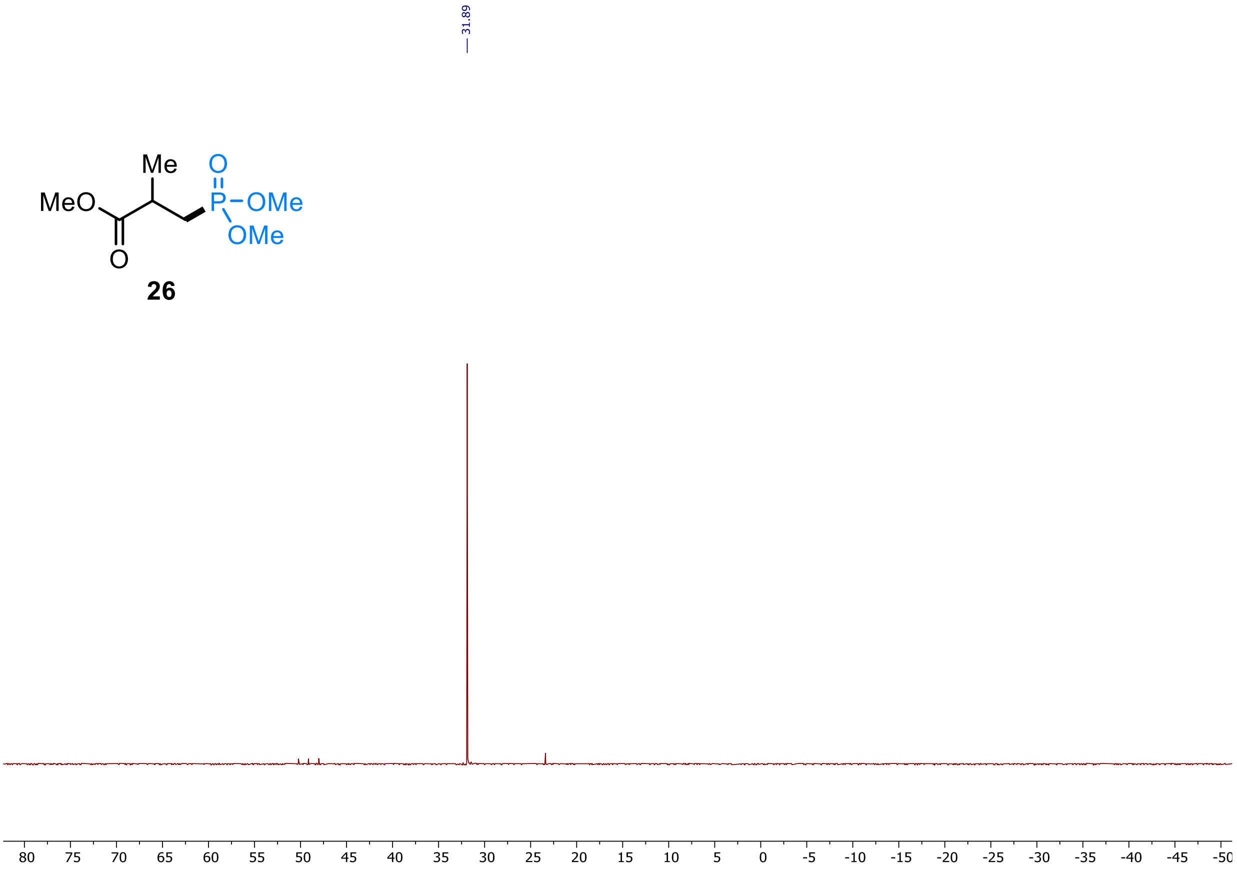

26

^{31}P NMR of crude reaction mixture of **27-int** (*see procedure*)

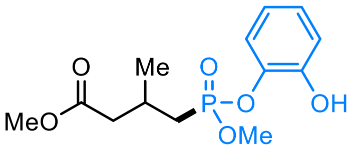

27-int

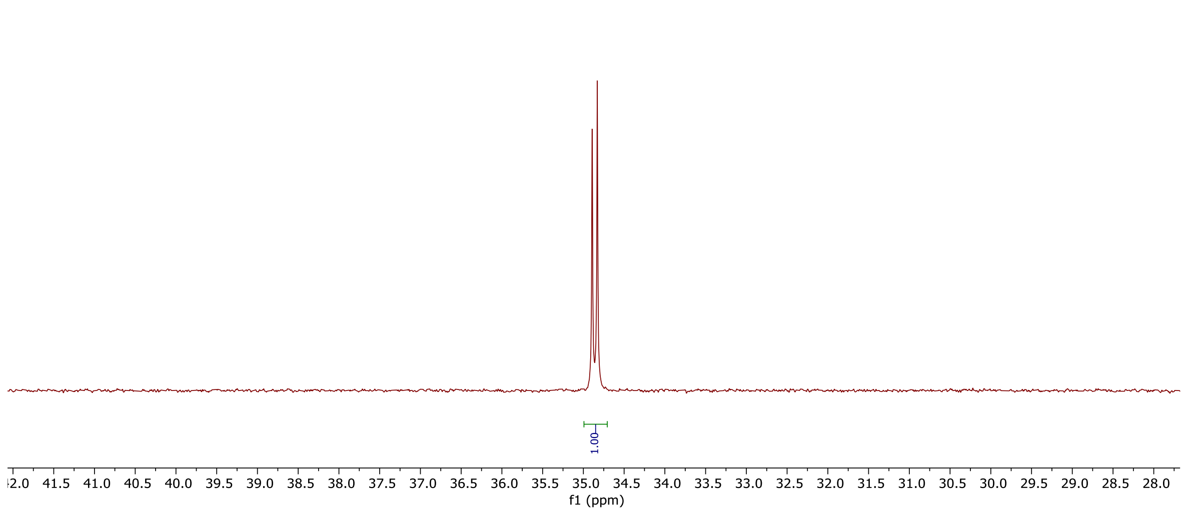

^{1}H NMR (400 MHz, $CDCl_3$) of **27** (*see procedure*)

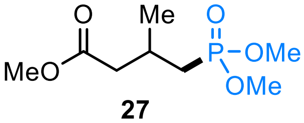

27

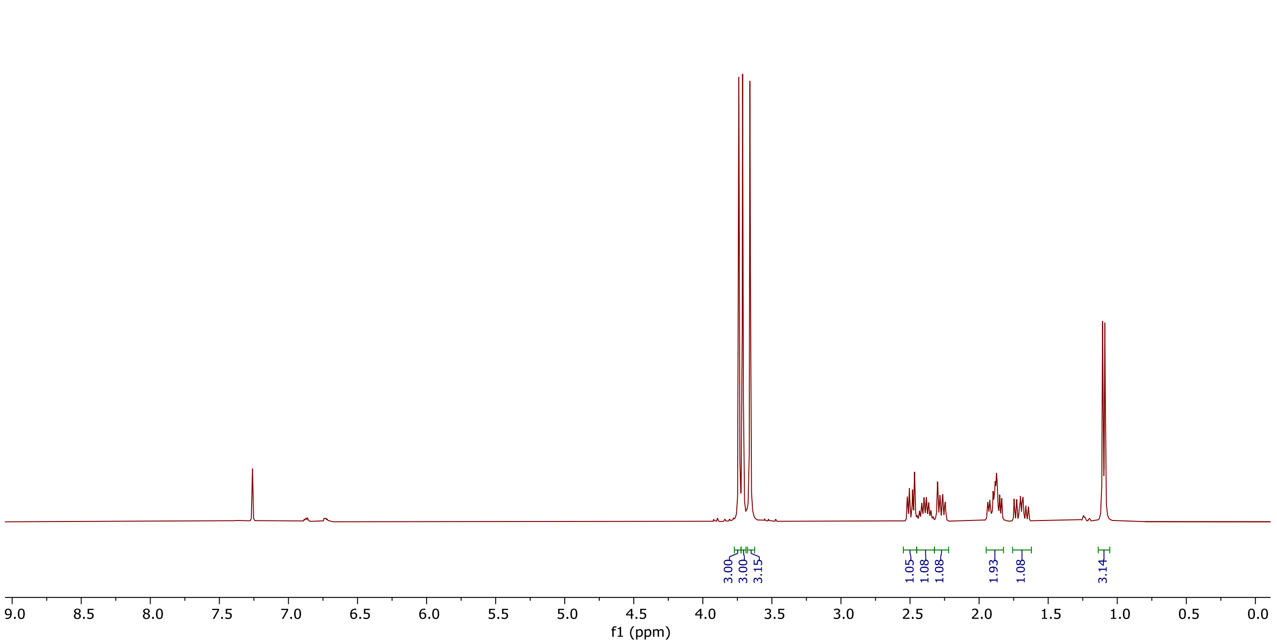

^{13}C NMR (101 MHz, $CDCl_3$) of **27**

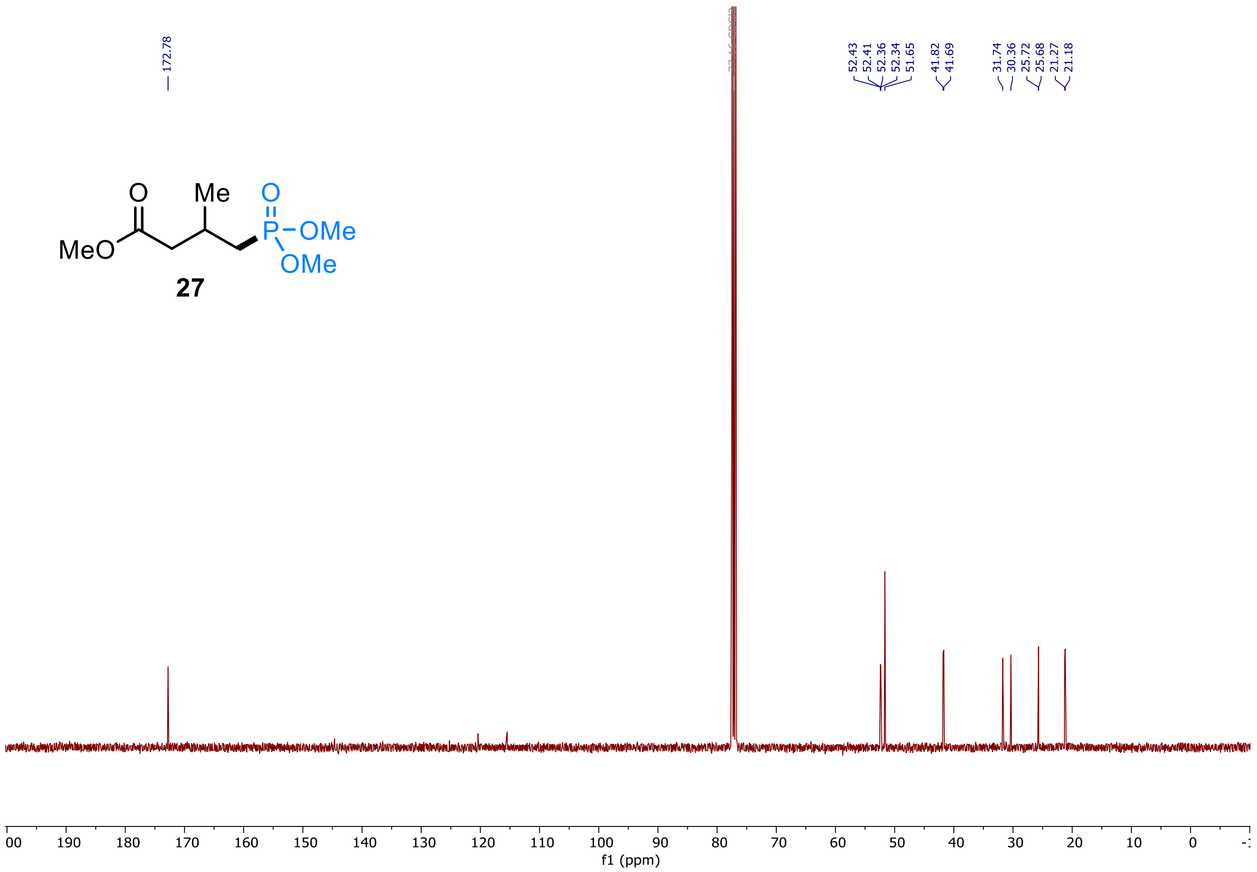

^{31}P NMR (400 MHz, $CDCl_3$) of **27**

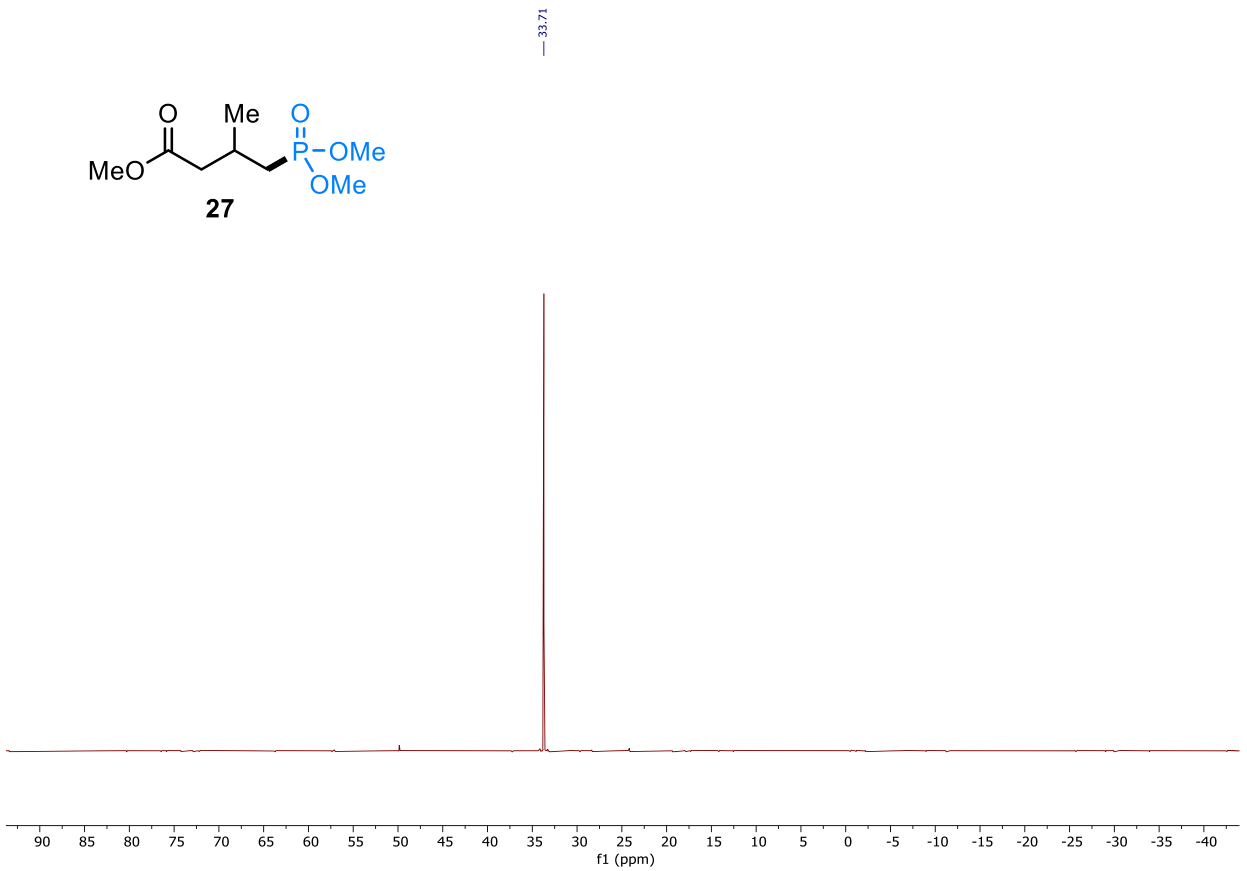

^{31}P NMR (162 MHz, $CDCl_3$) of crude **28-int** (*see procedure*)

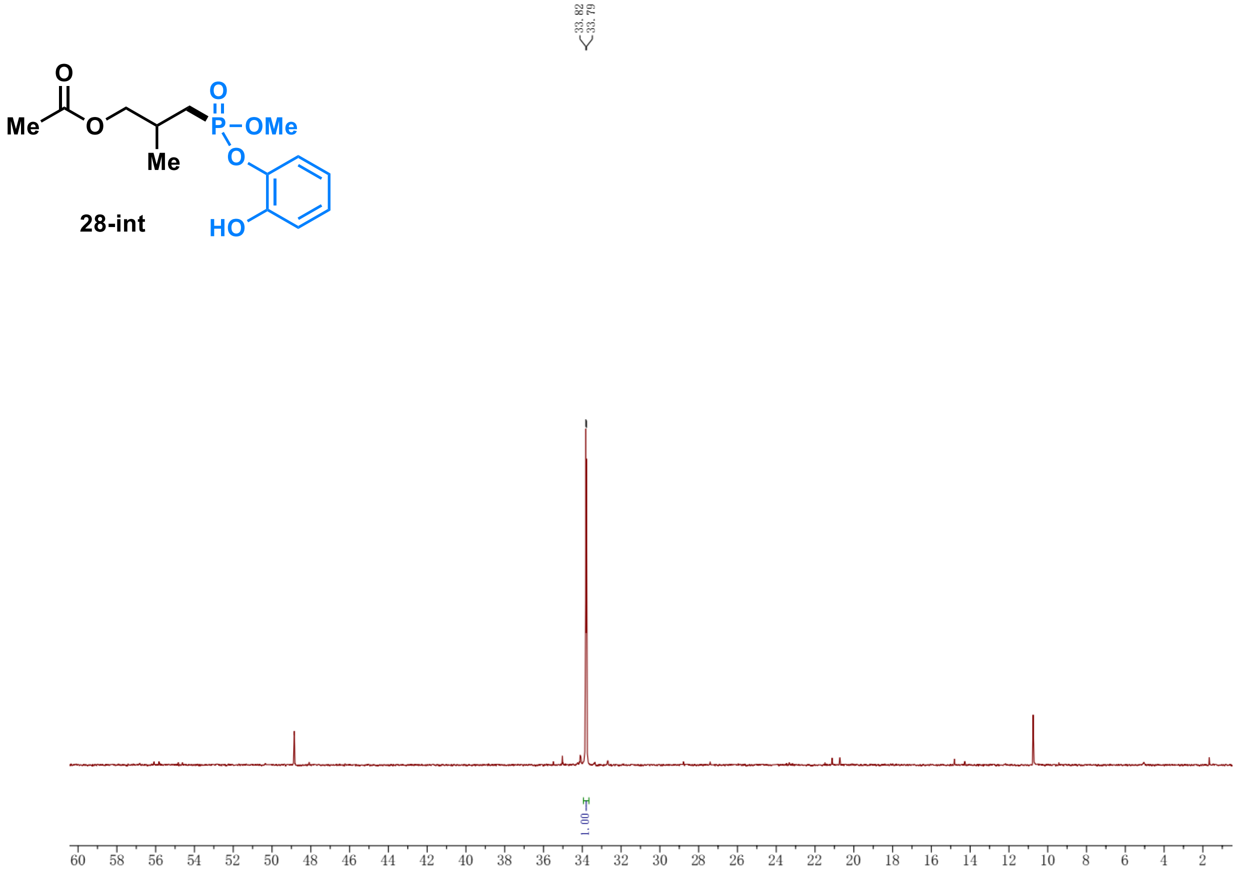

^{1}H NMR (400 MHz, $CDCl_3$) of **28** (*see procedure*)

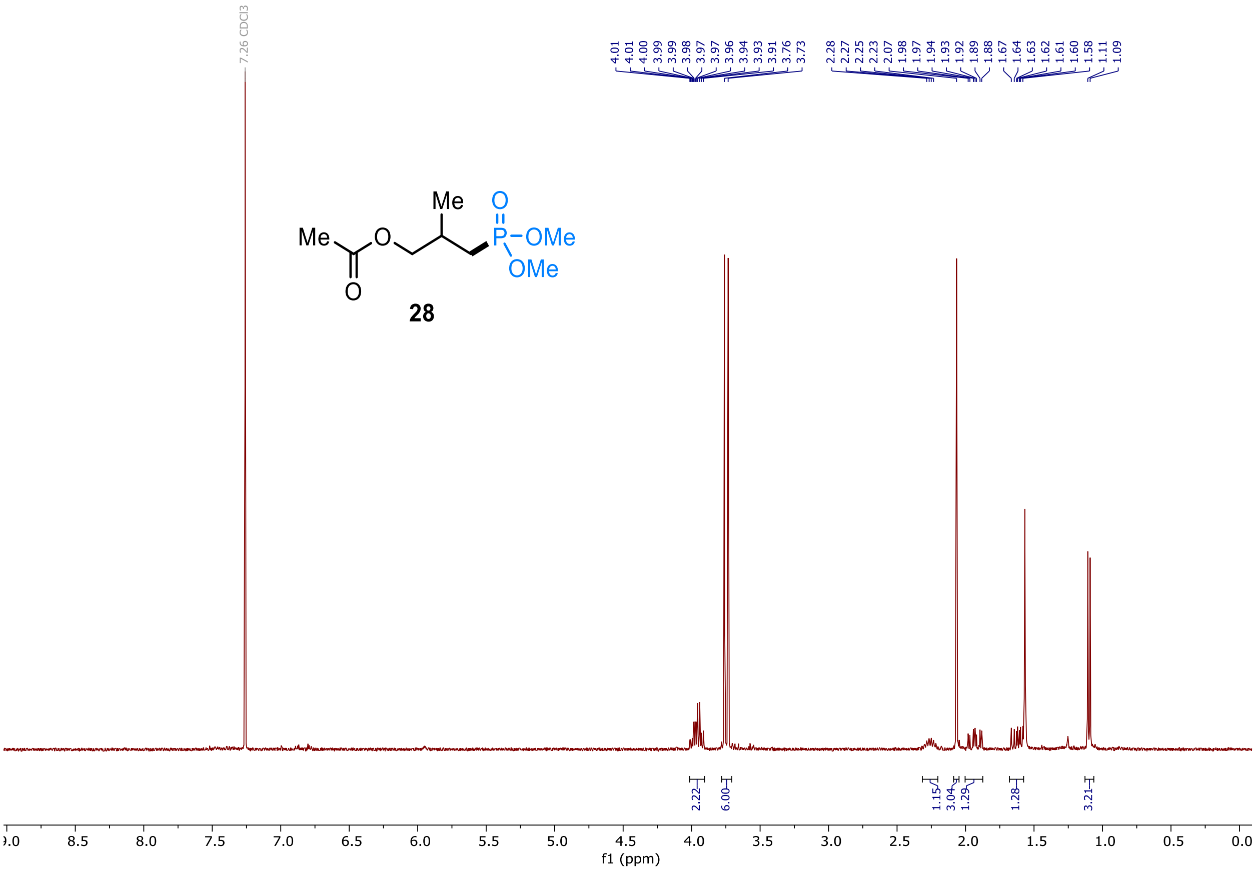

^{13}C NMR (101 MHz, $CDCl_3$) of **28**

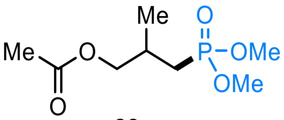

28

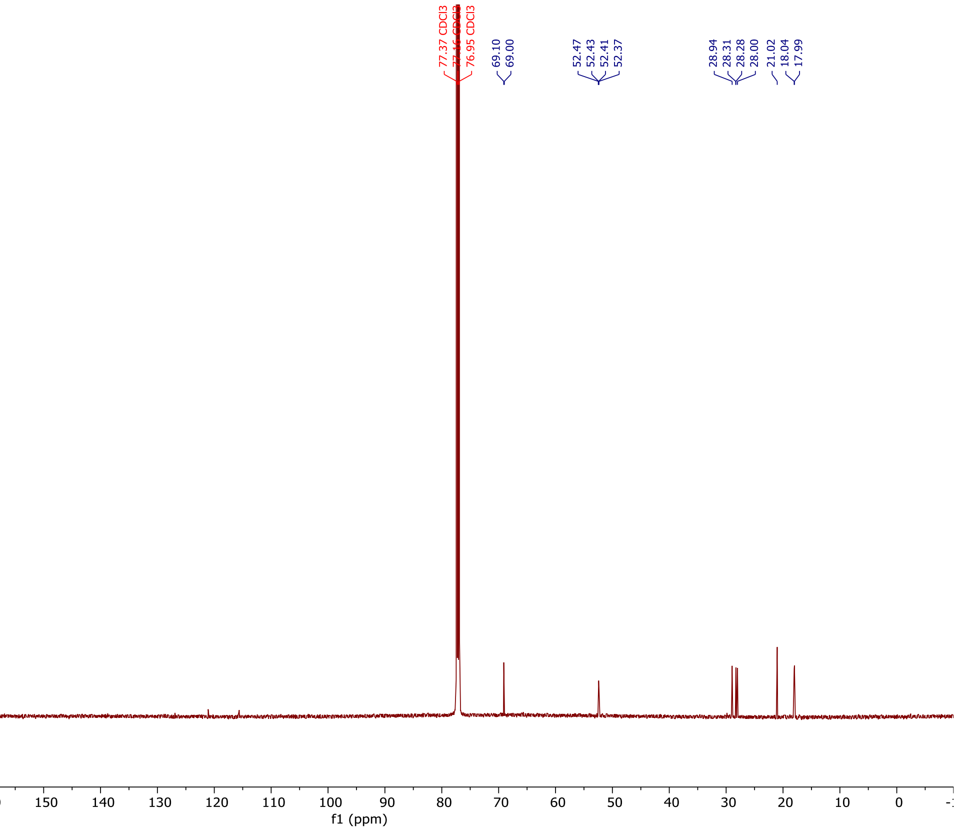

^{31}P NMR (400 MHz, $CDCl_3$) of **28**

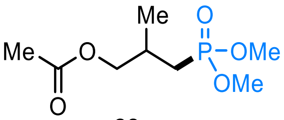

28

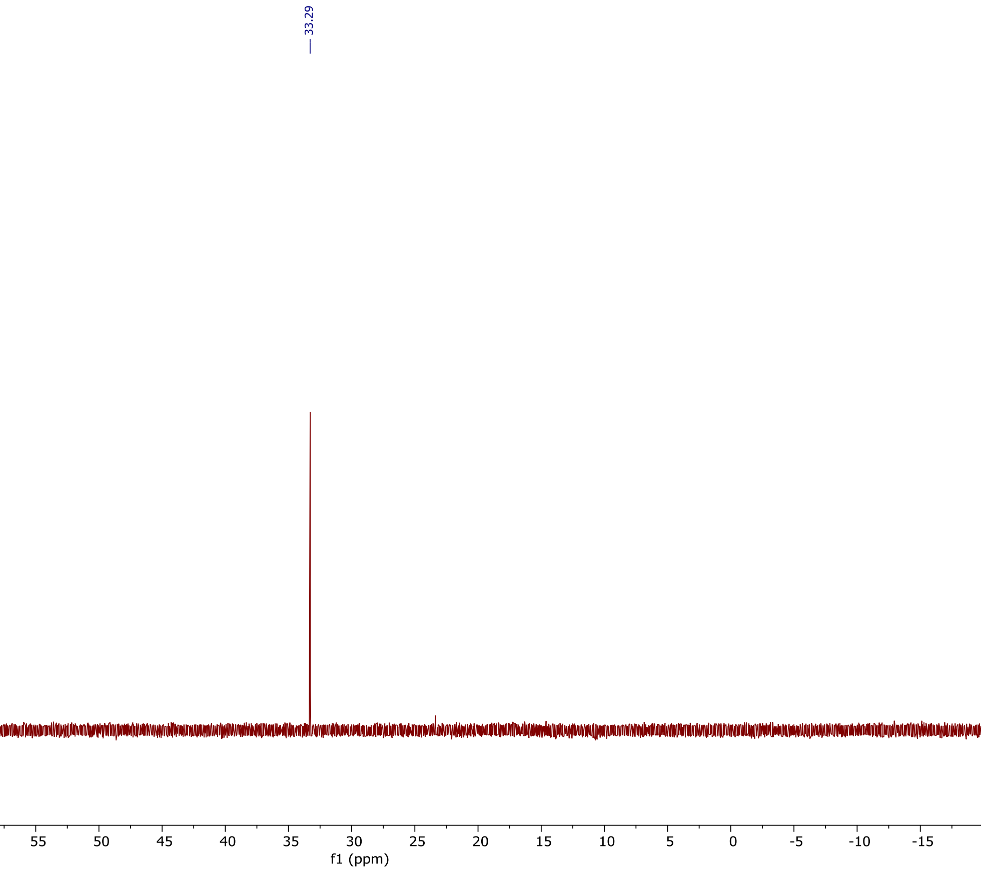

^{31}P NMR (162 MHz, $CDCl_3$) of crude **29-int** (*see procedure*)

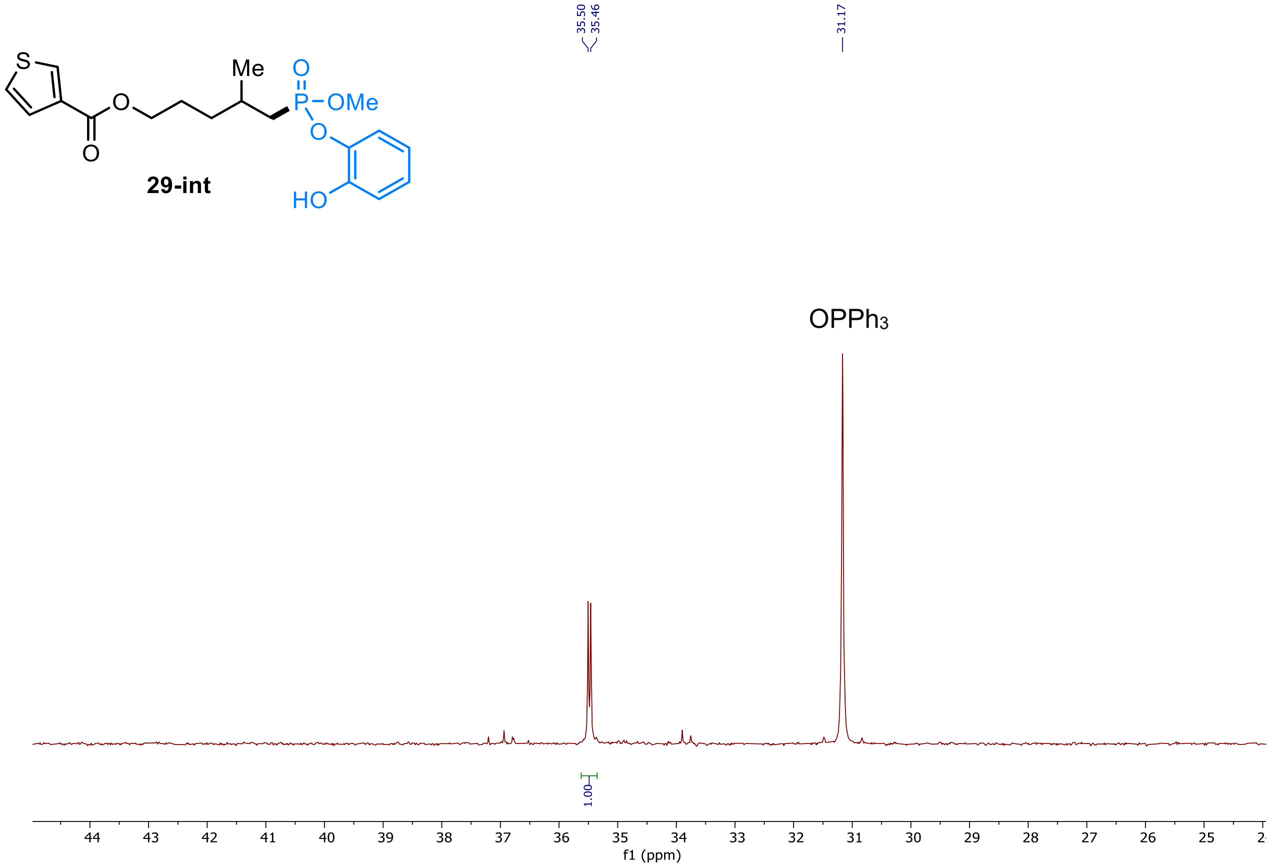

^{1}H NMR (400 MHz, $CDCl_3$) of **29** (*see procedure*)

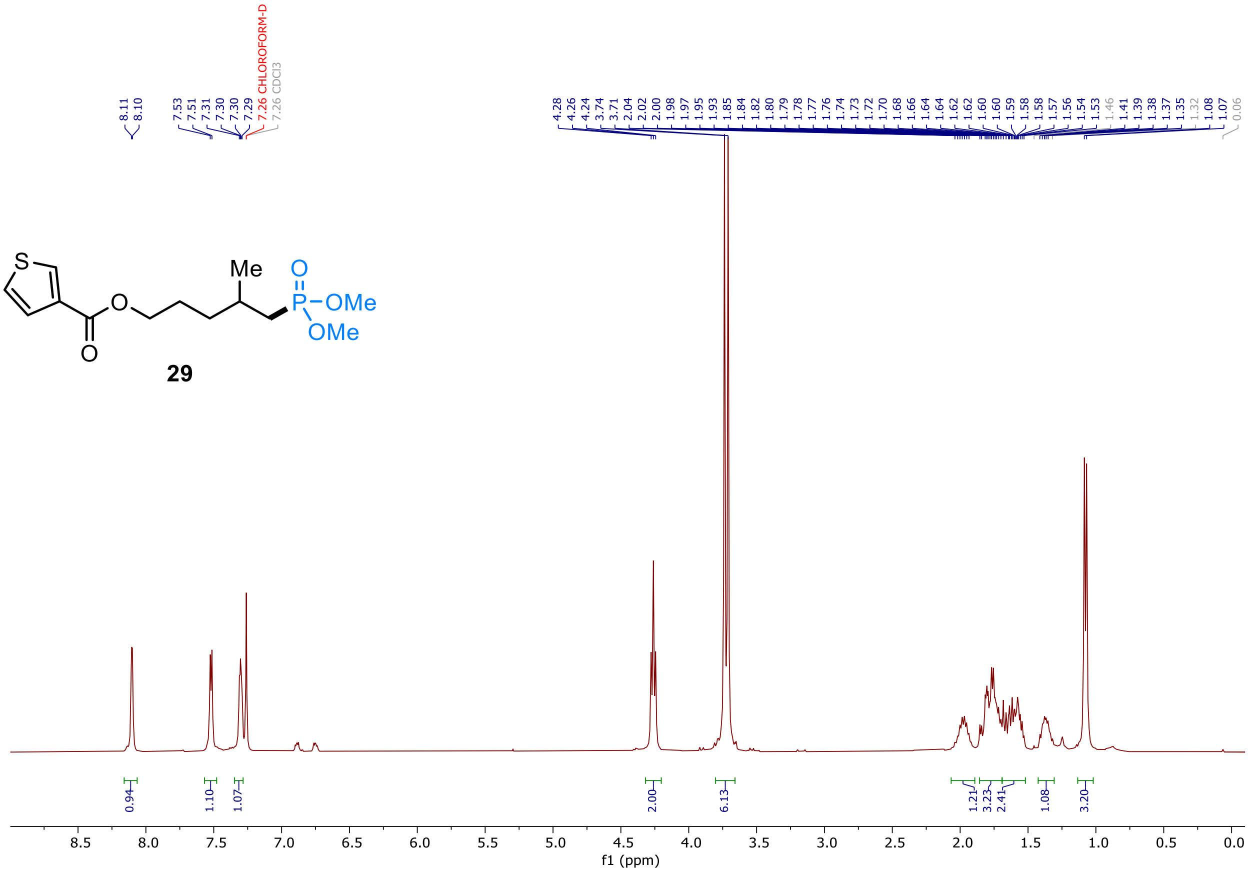

^{13}C NMR (101 MHz, $CDCl_3$) of **29**

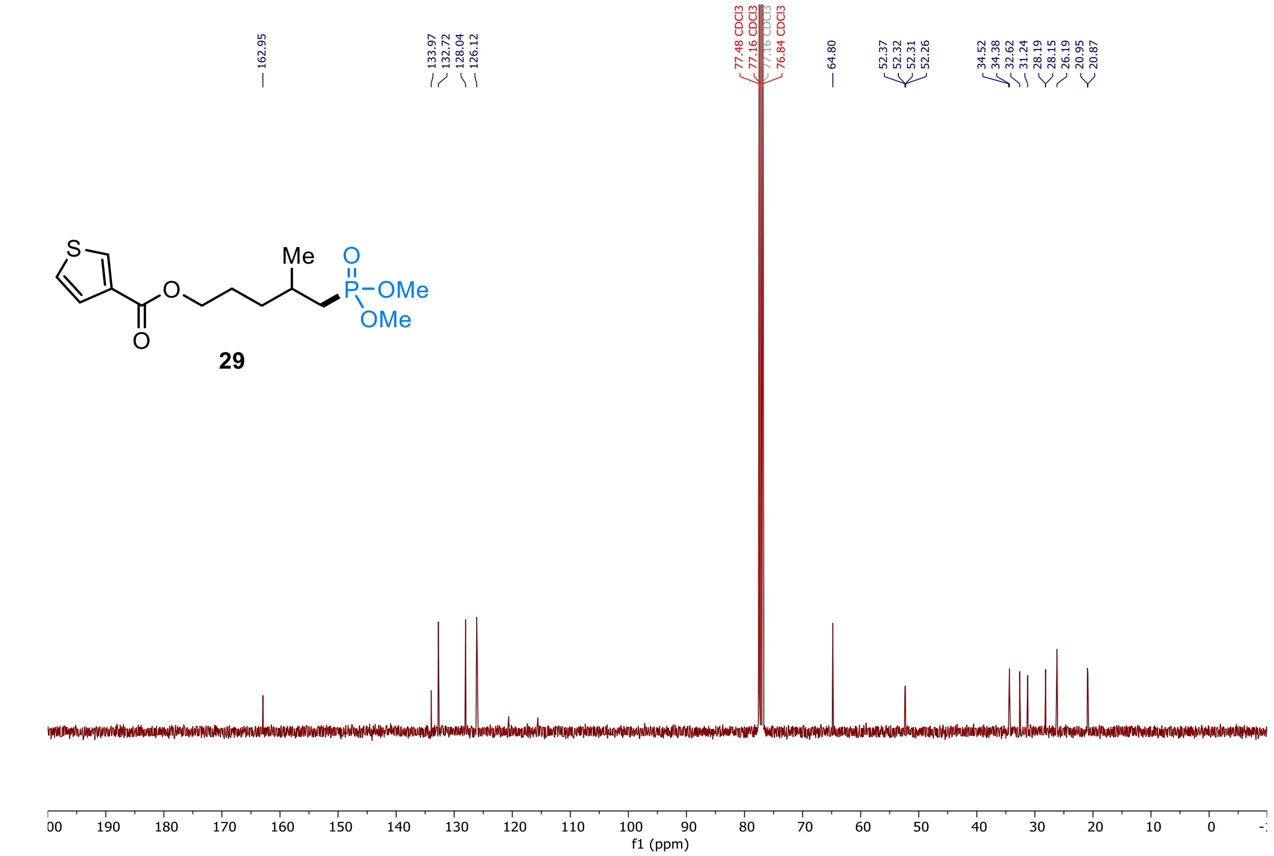

^{31}P NMR (400 MHz, $CDCl_3$) of **29**

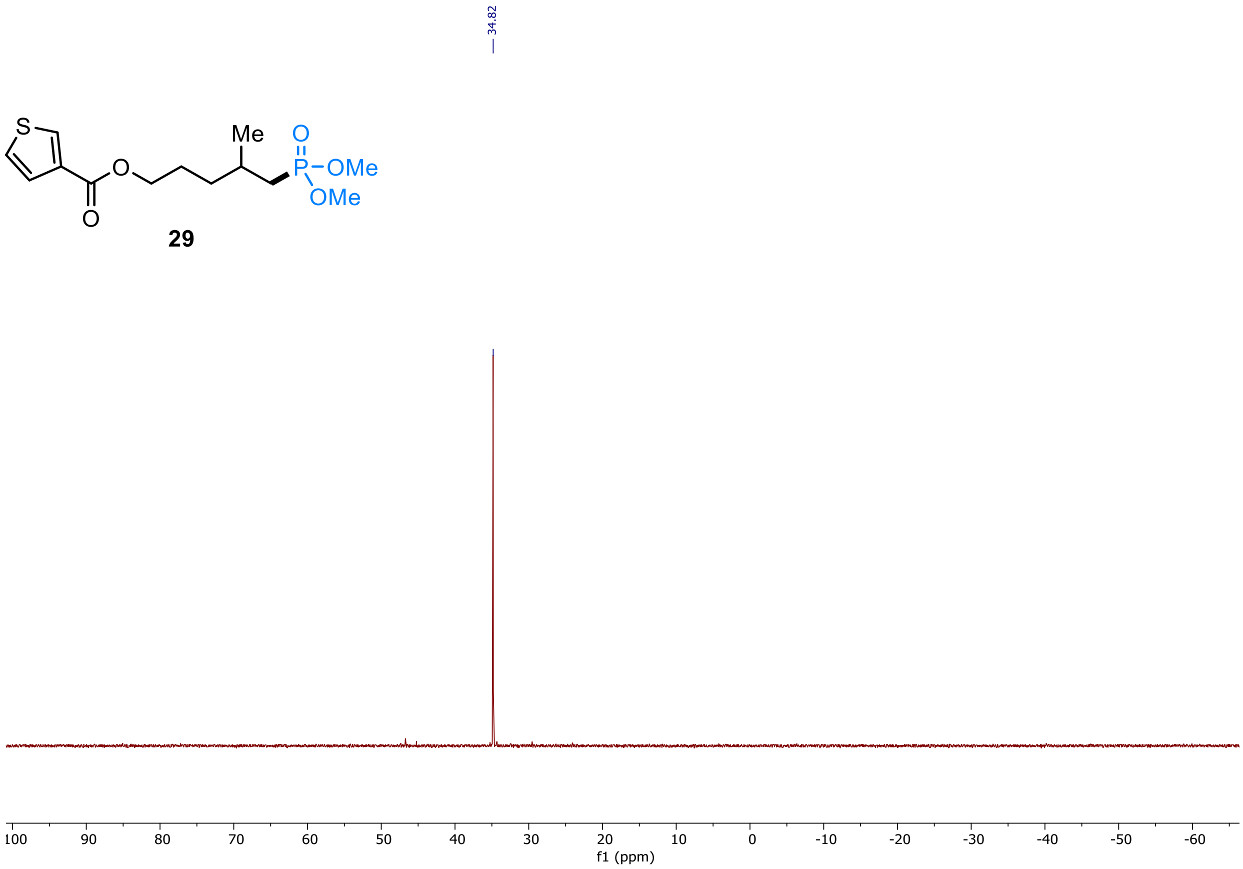

^{31}P NMR (162 MHz, $CDCl_3$) of crude **30-int** (*see procedure*)

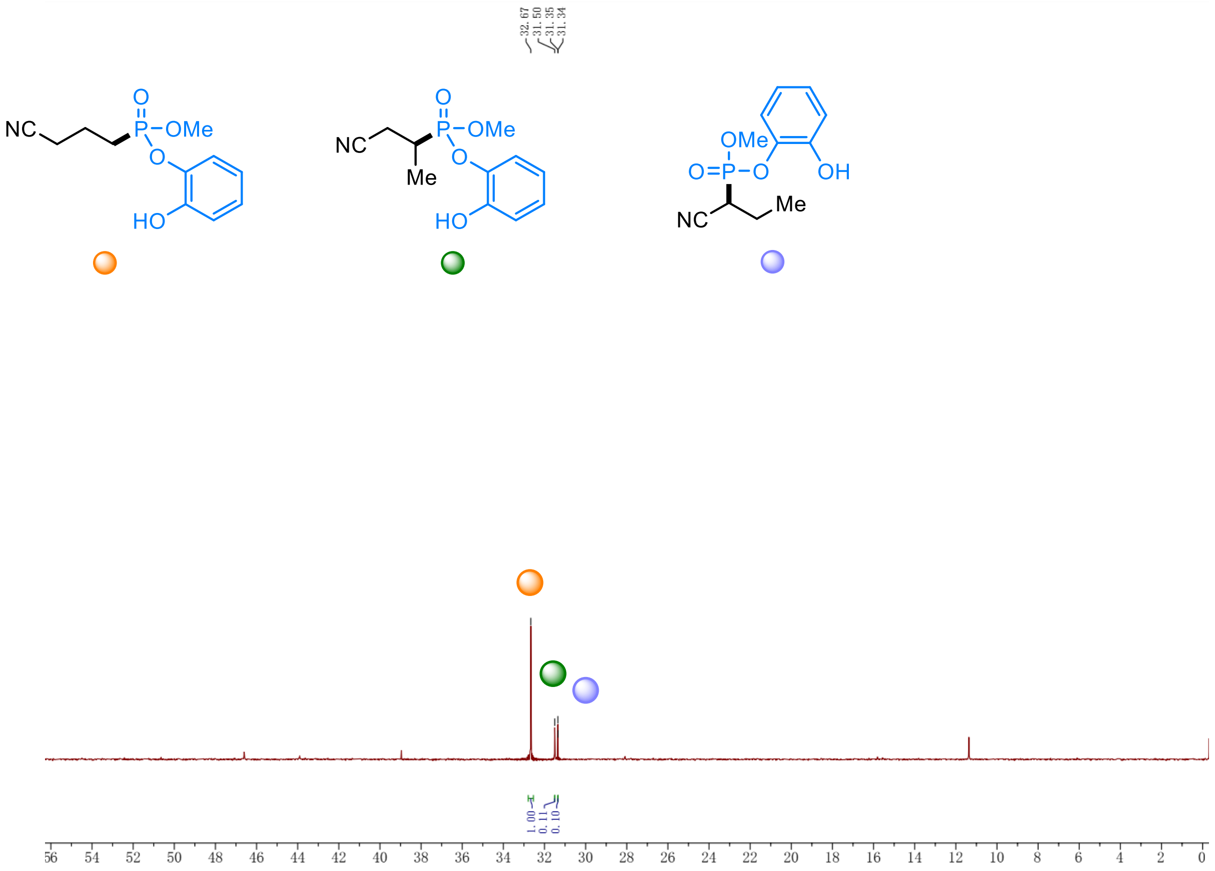

^{1}H NMR (400 MHz, $CDCl_3$) of **30** (*see procedure*)

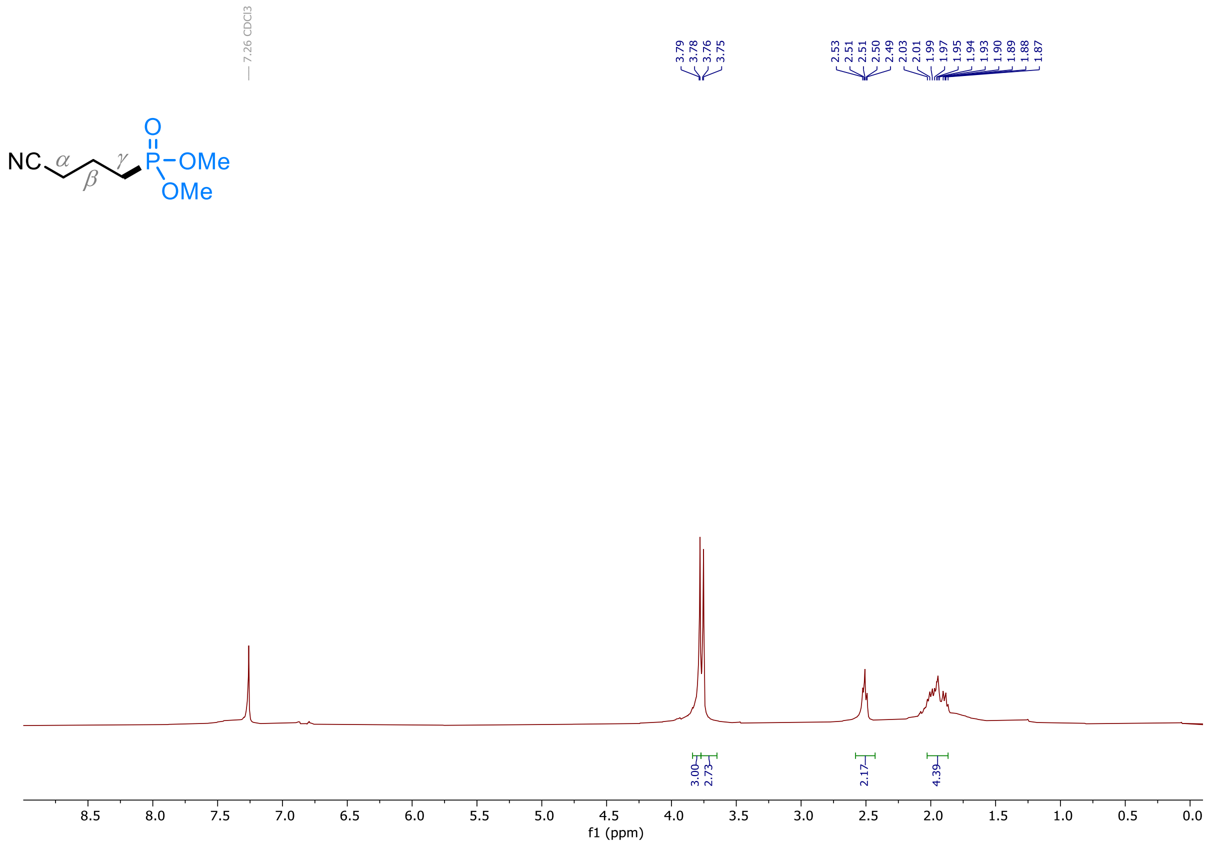

^{13}C NMR (101 MHz, CDCl$_3$) of **30**

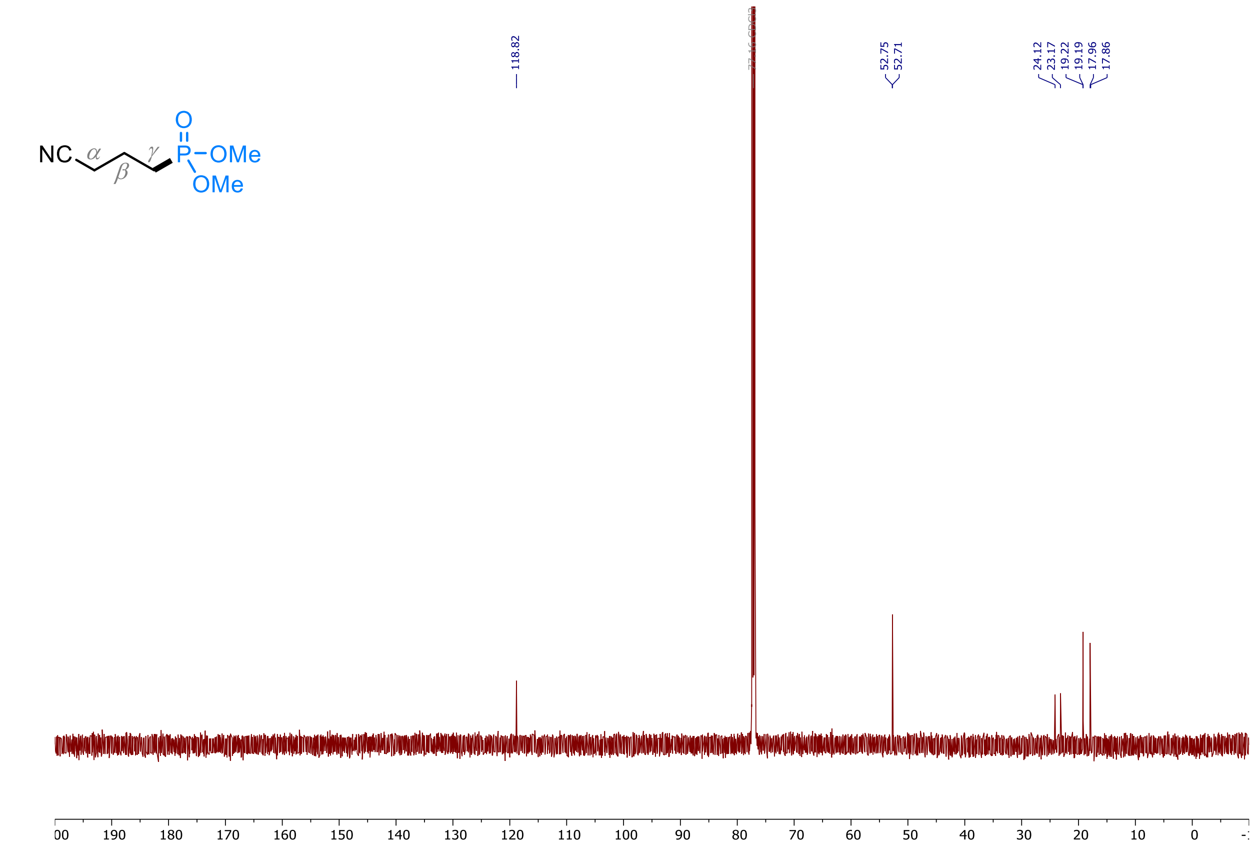

^{31}P NMR (162 MHz, CDCl$_3$) of **30**

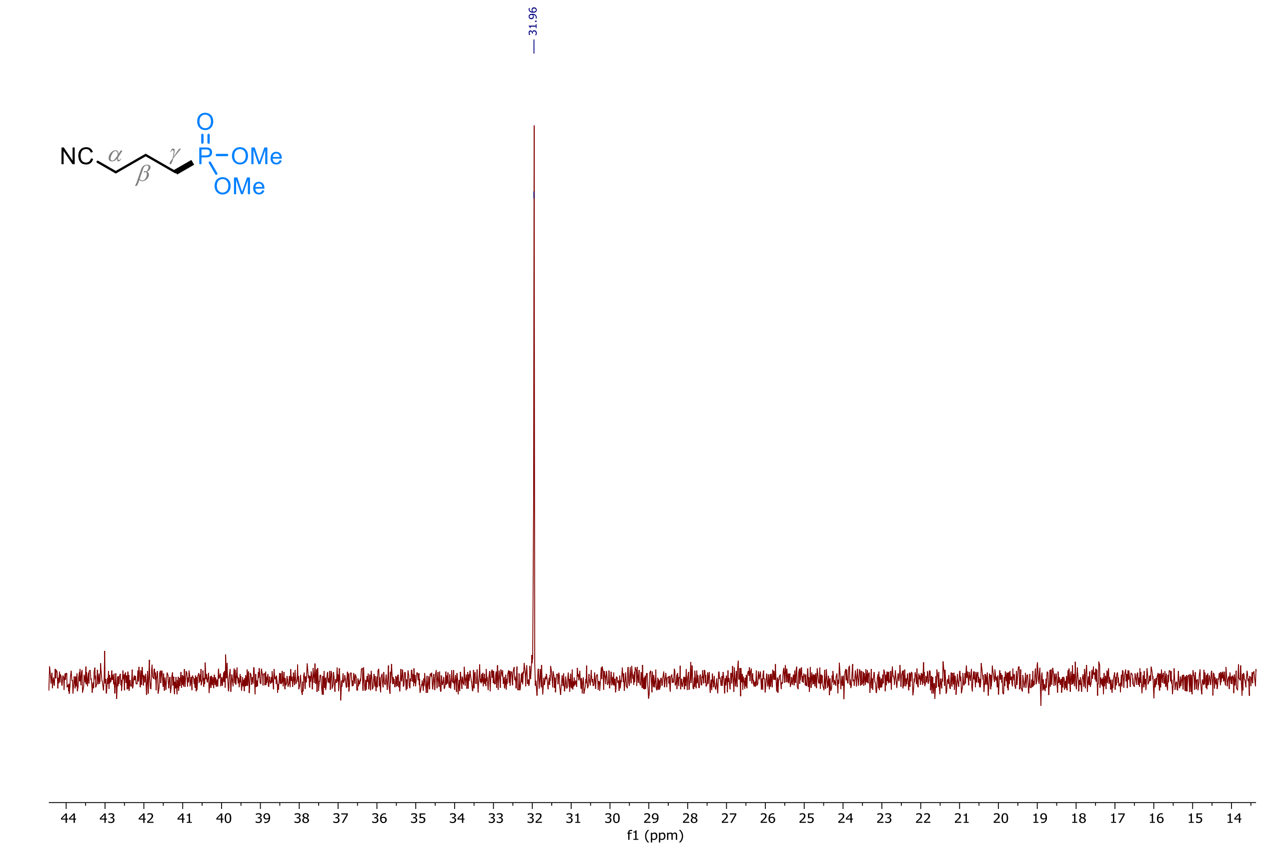

^{31}P NMR (162 MHz, CDCl$_3$) of crude **31-int** (*see procedure*)

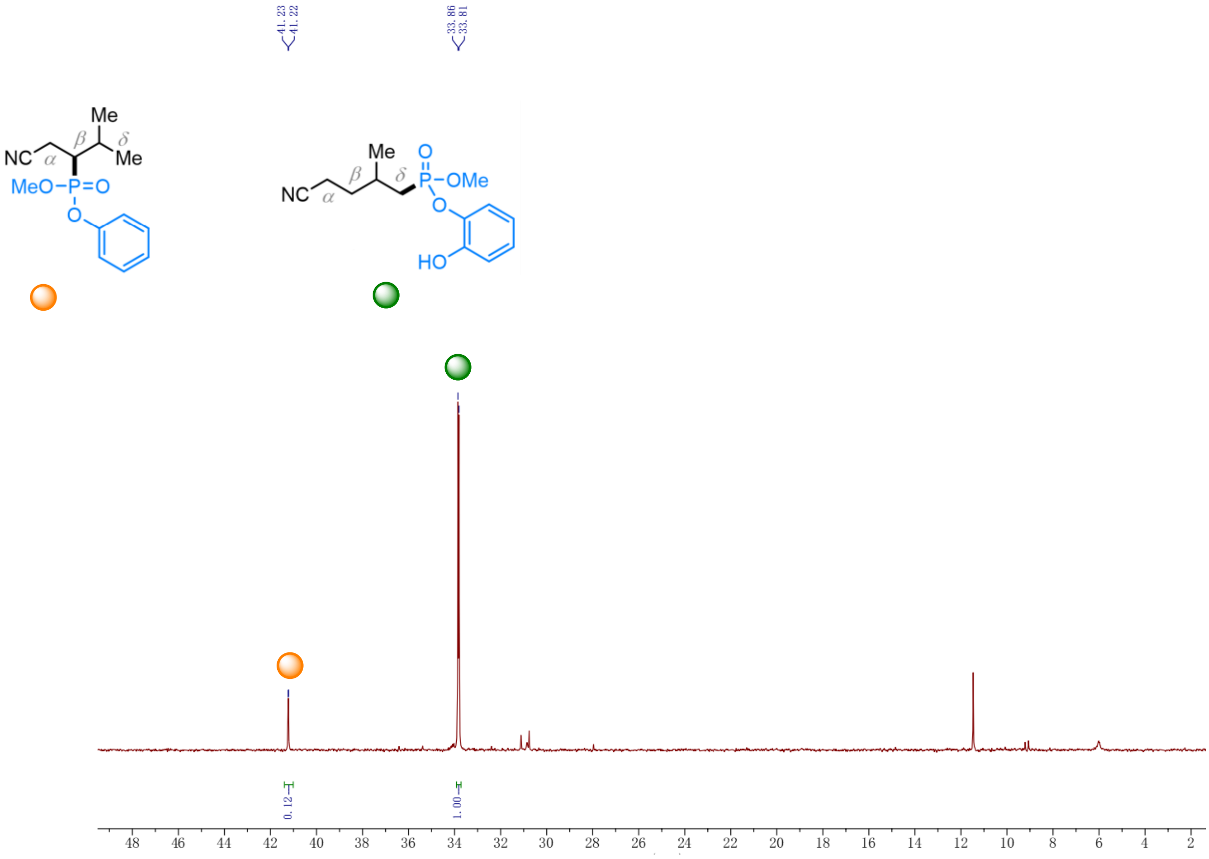

^{1}H NMR (400 MHz, CDCl$_3$) of **31** (*see procedure*)

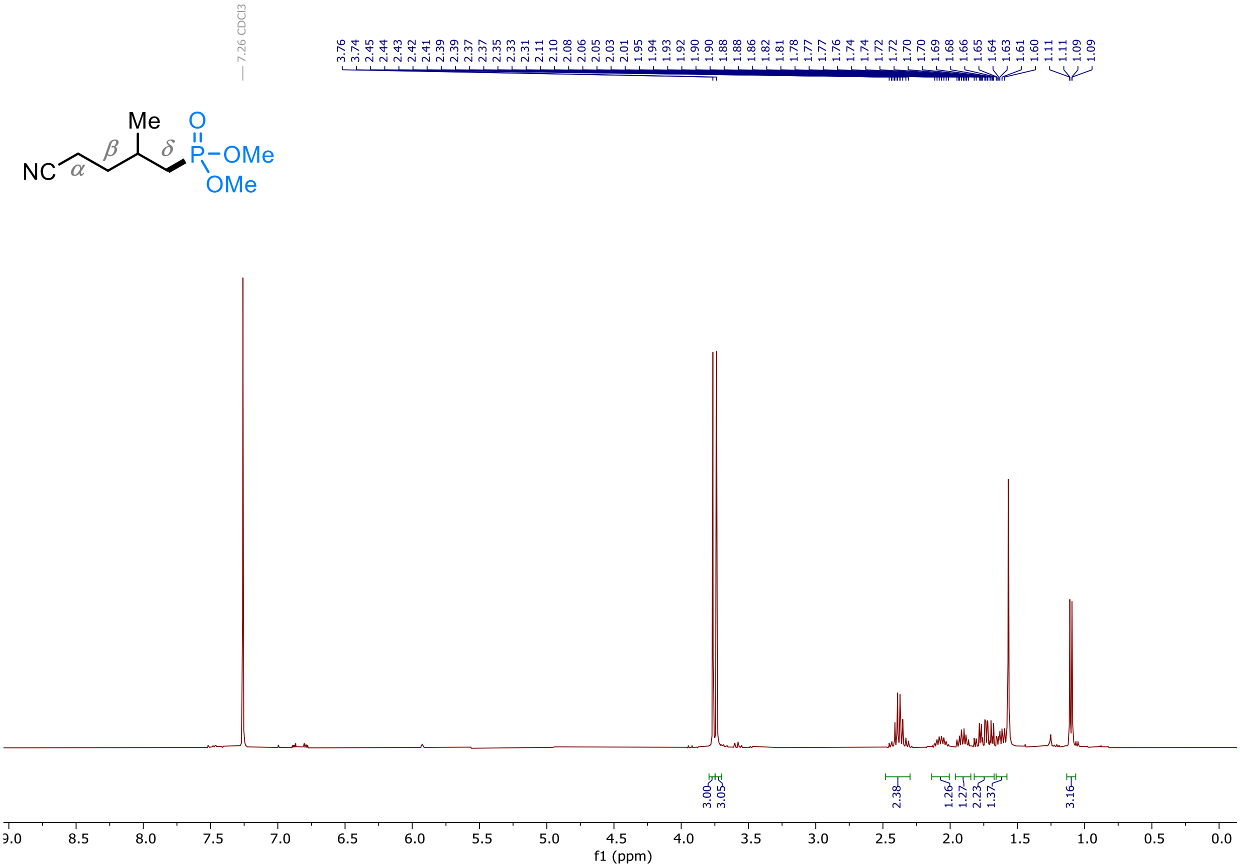

^{13}C NMR (151 MHz, $CDCl_3$) of **31**

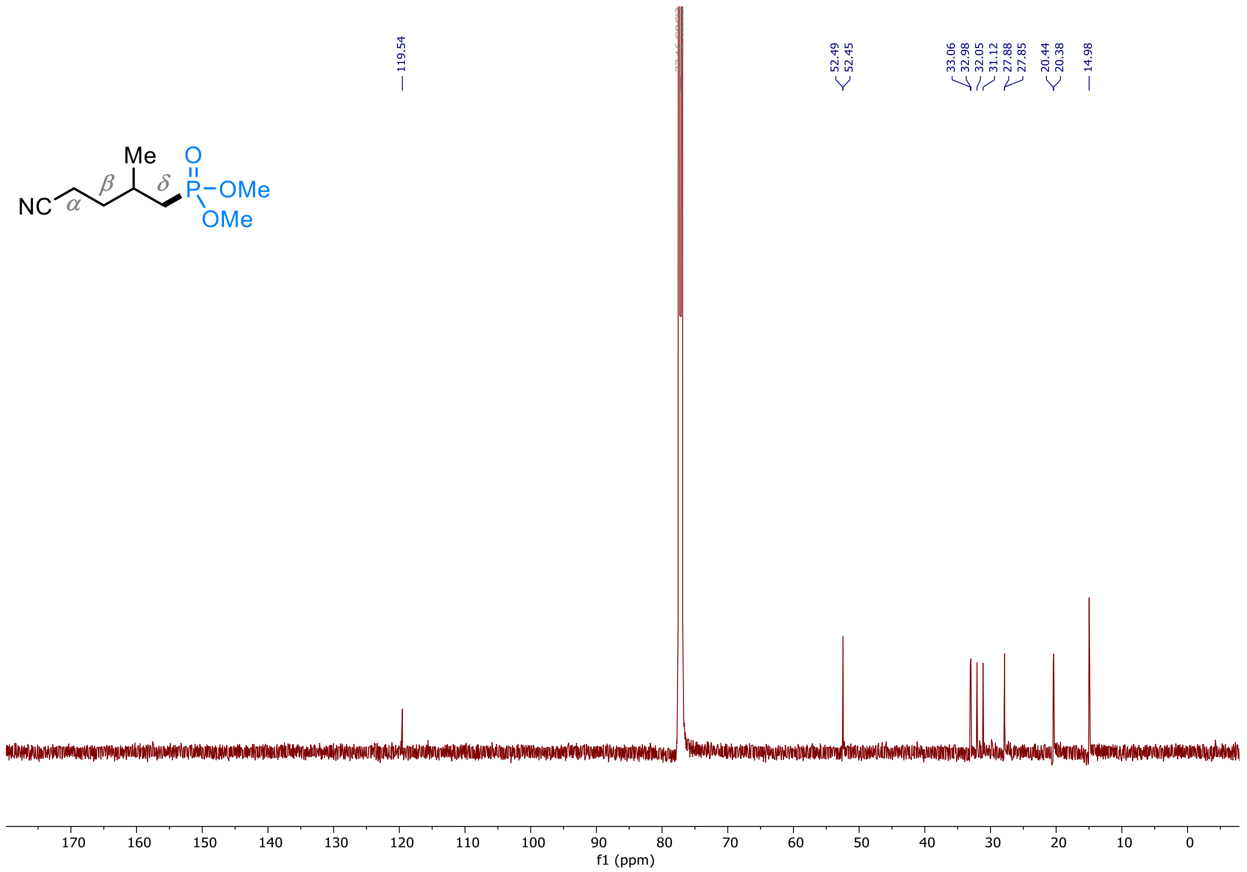

^{31}P NMR (162 MHz, $CDCl_3$) of **31**

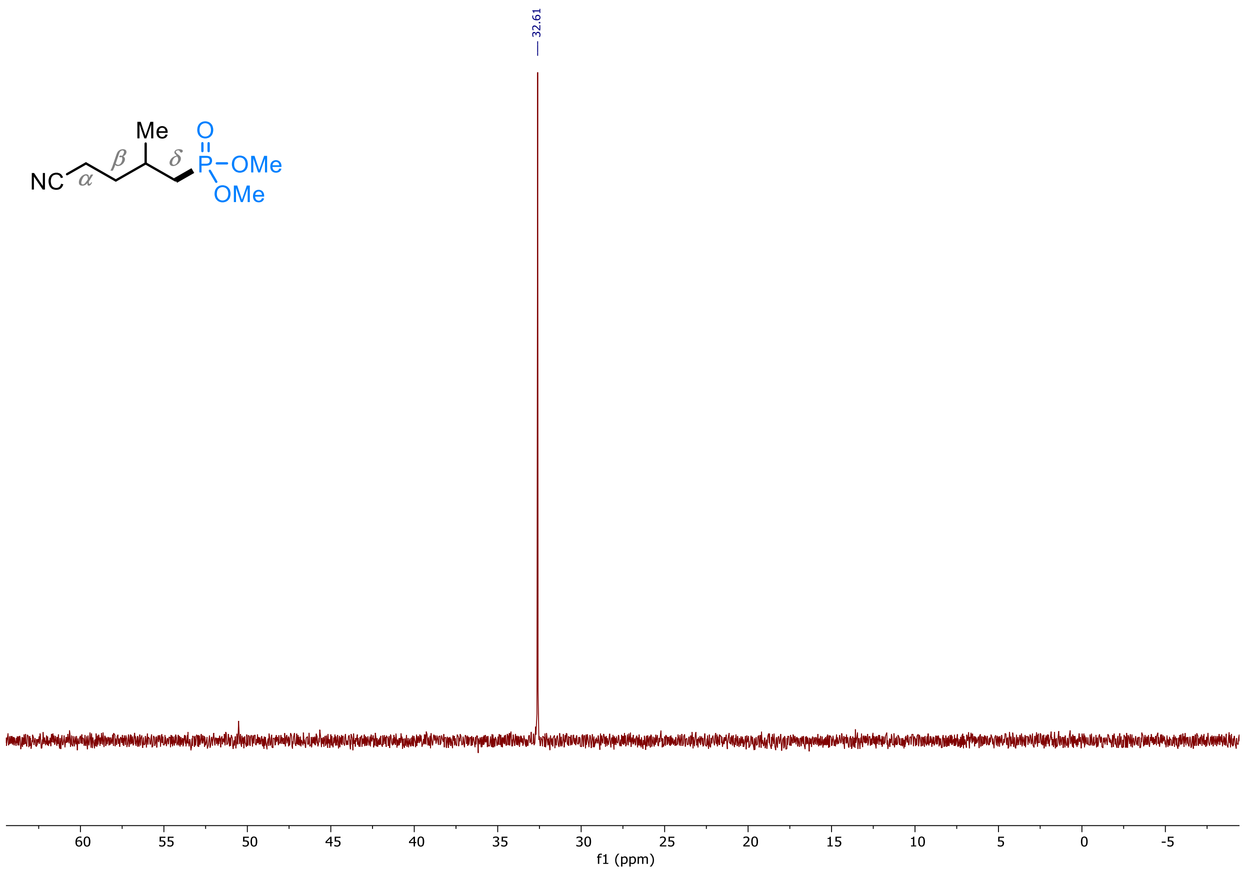

^{1}H NMR (400 MHz, $CDCl_3$) of **32** (*see procedure*)

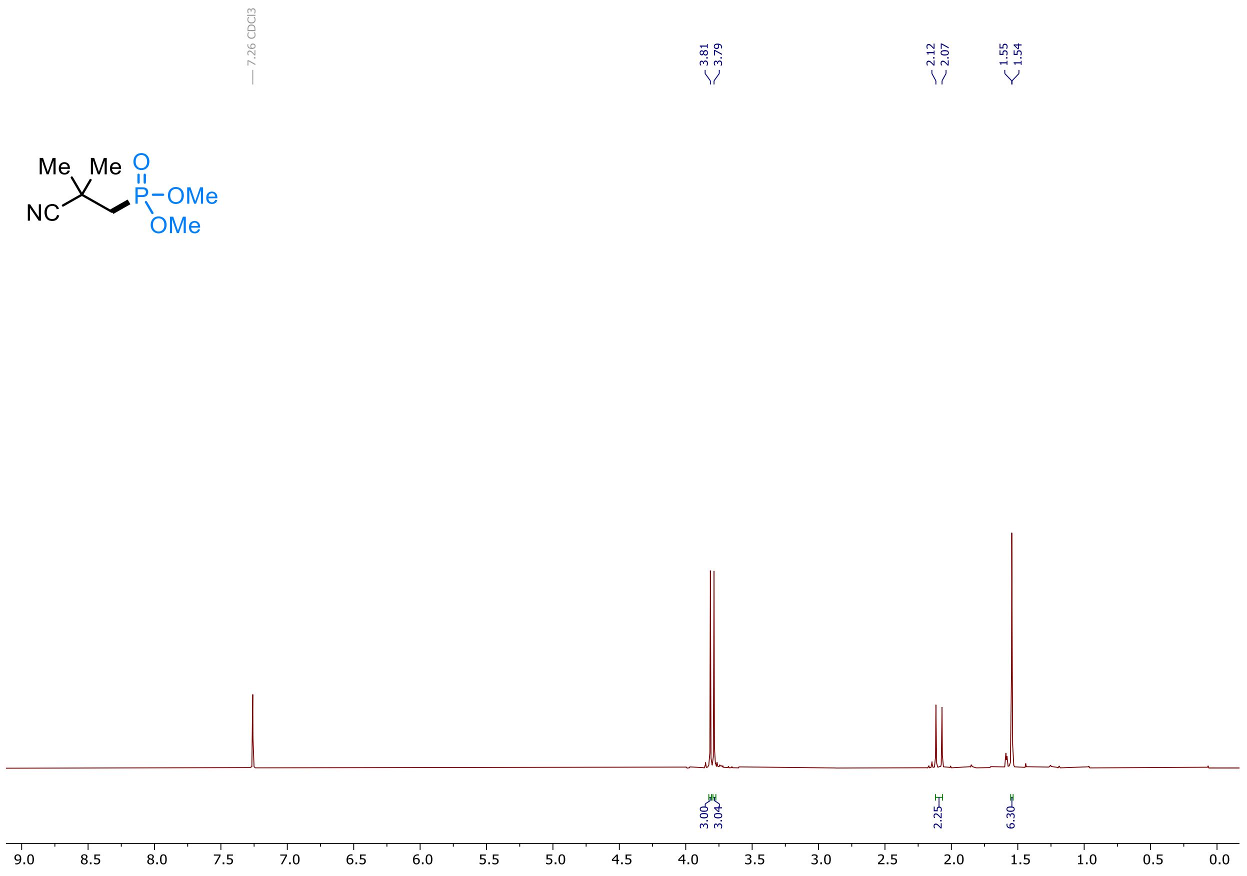

^{13}C NMR (151 MHz, $CDCl_3$) of **32**

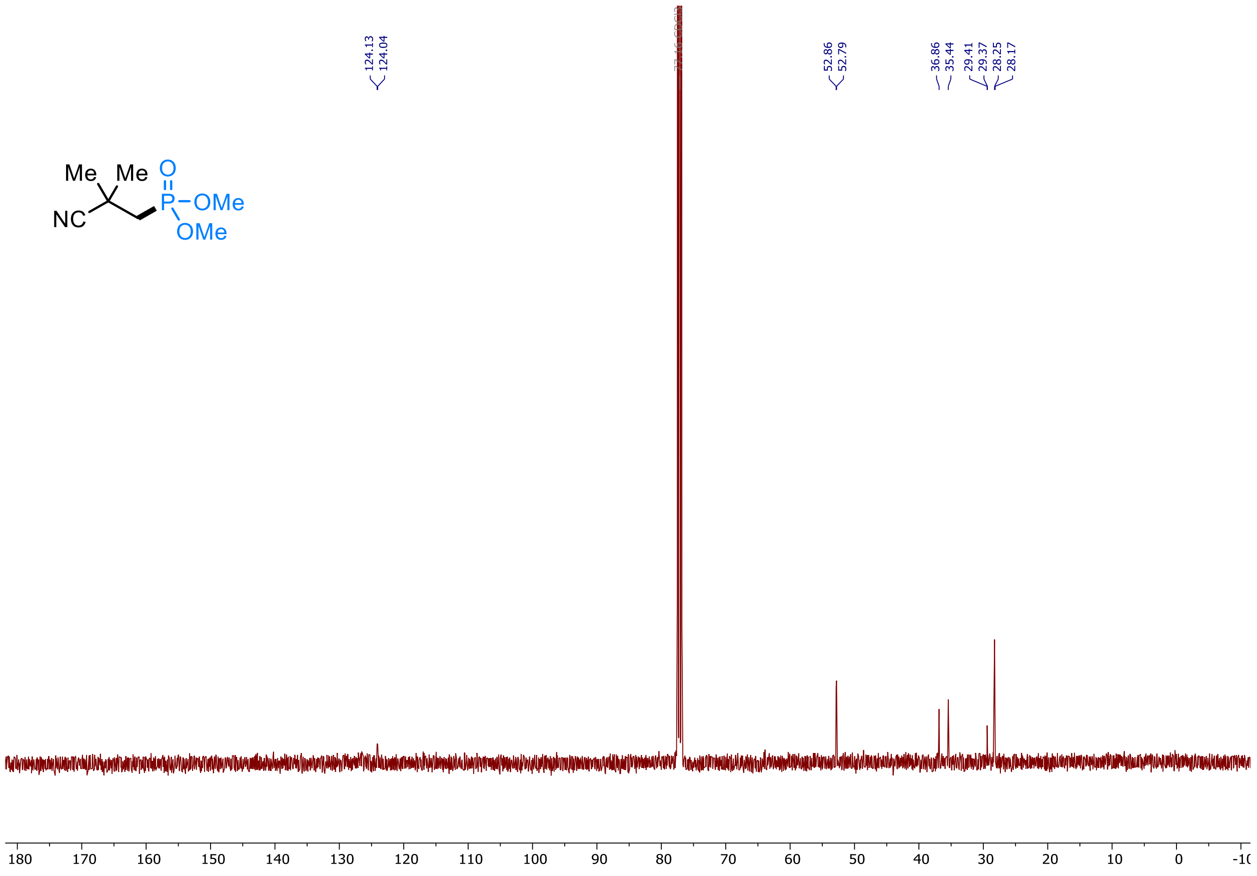

^{31}P NMR (162 MHz, $CDCl_3$) of **32**

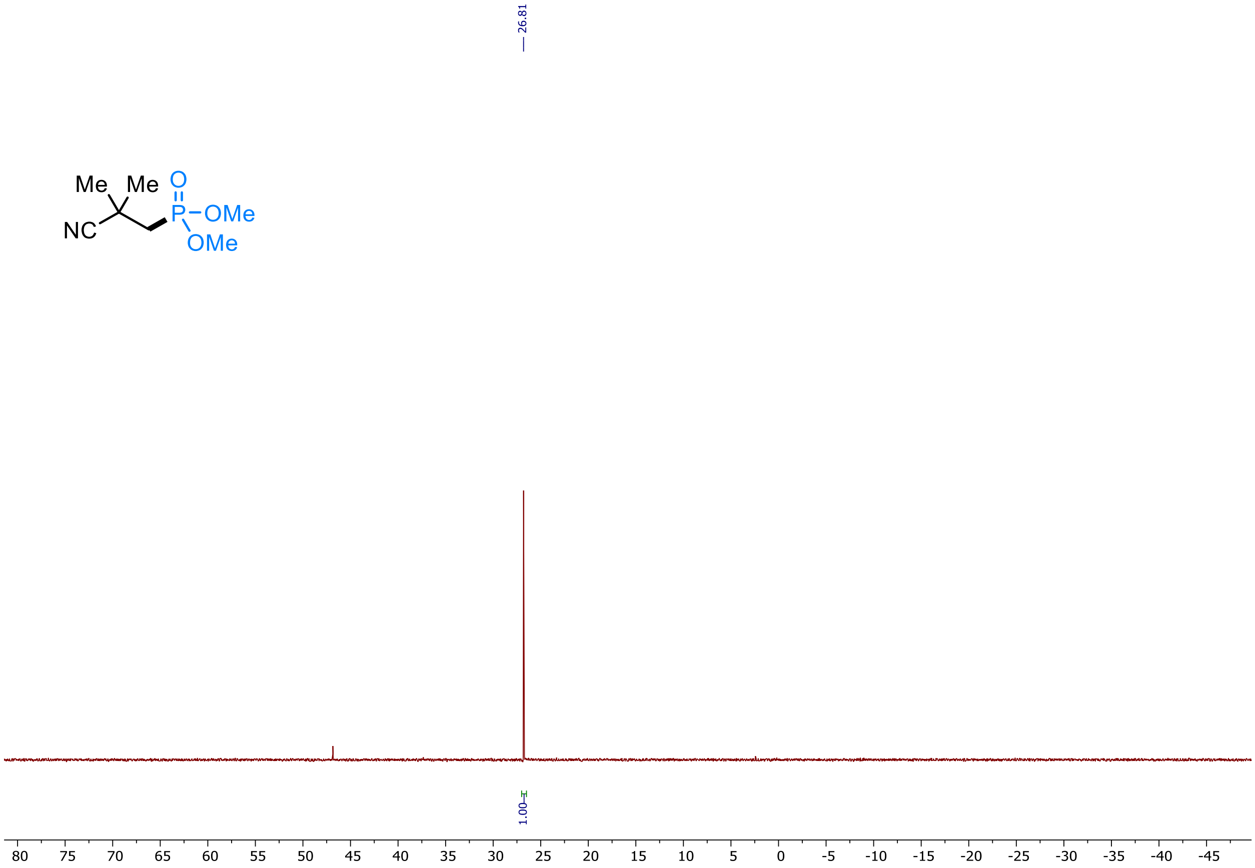

^{31}P NMR (162 MHz, $CDCl_3$) of crude **33-int** (*see procedure*)

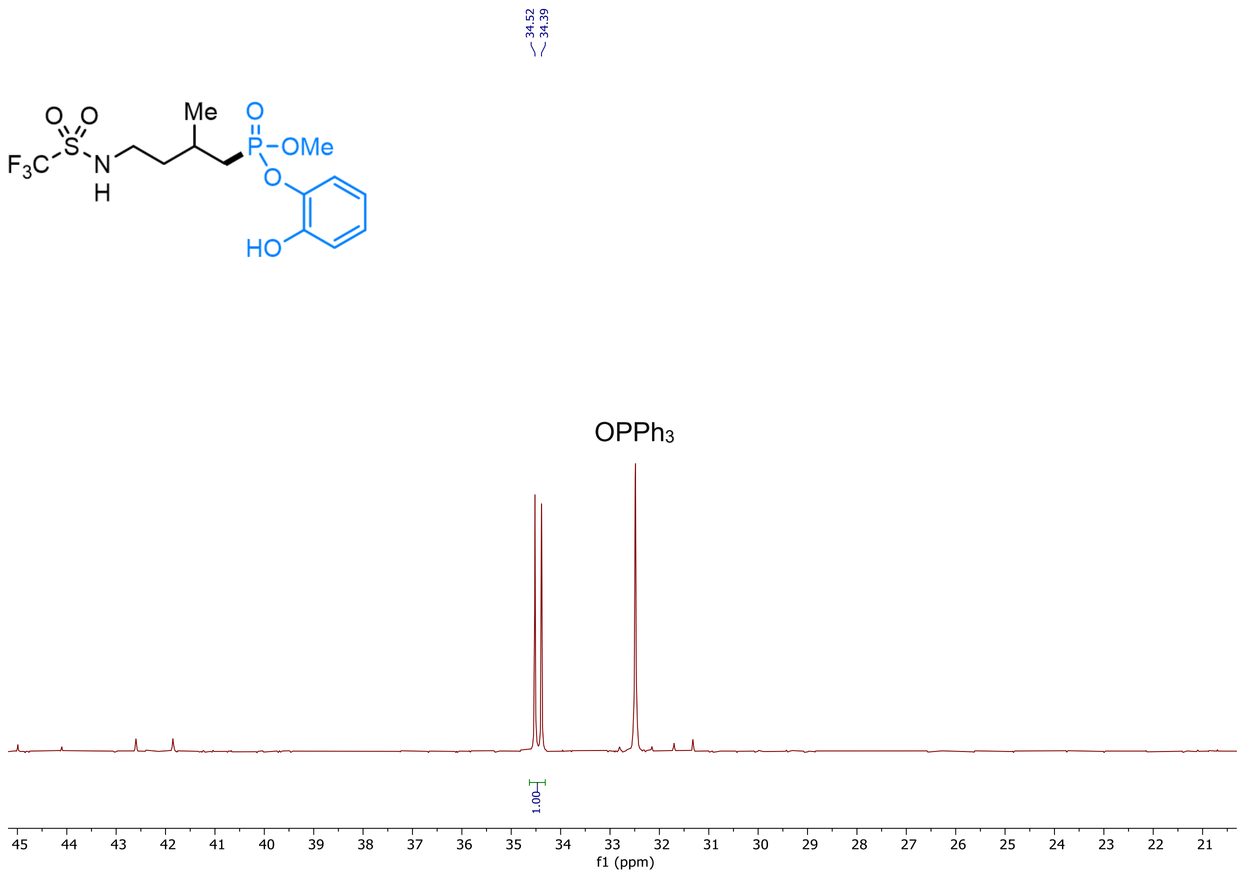

^{1}H NMR (400 MHz, $CDCl_3$) of **33** (*see procedure*)

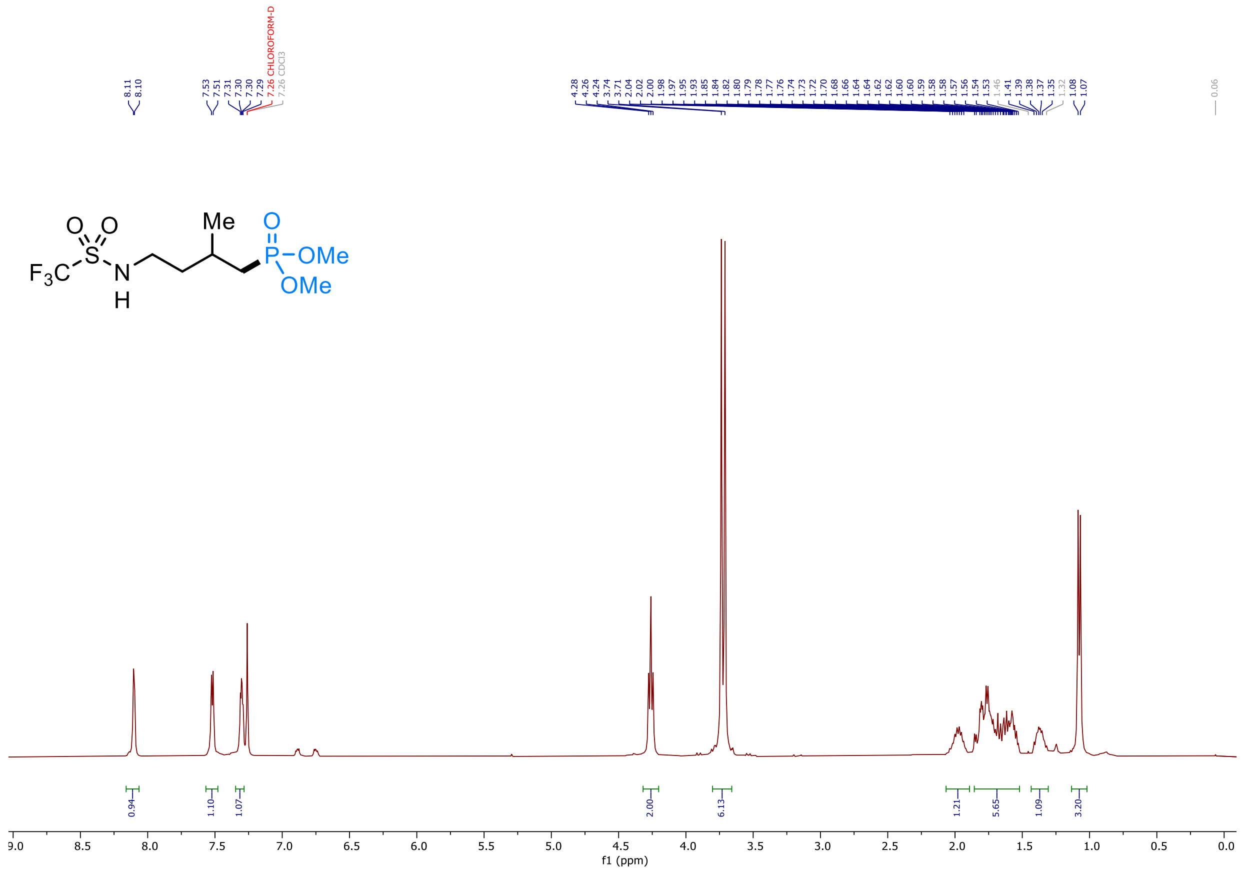

^{13}C NMR (101 MHz, $CDCl_3$) of **33**

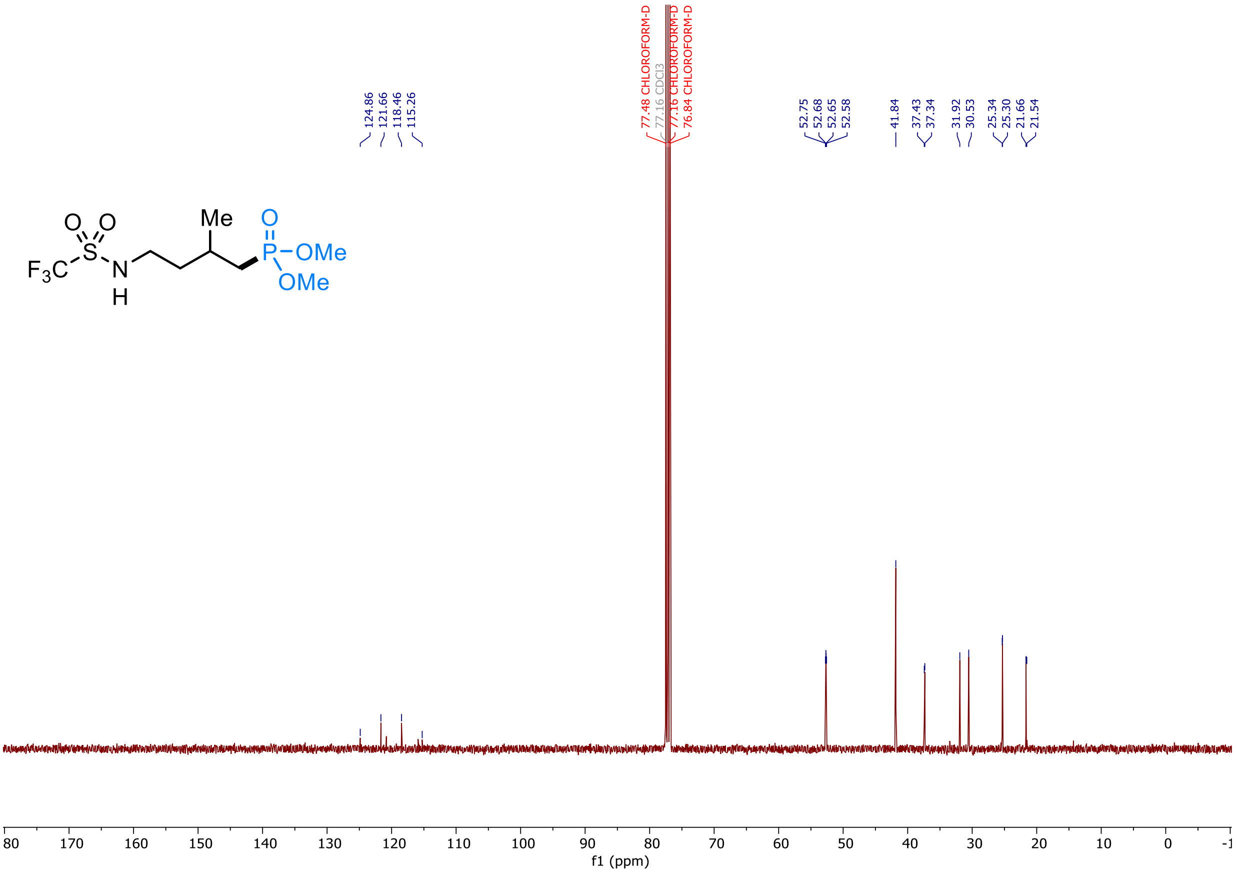

^{31}P NMR (400 MHz, $CDCl_3$) of **33**

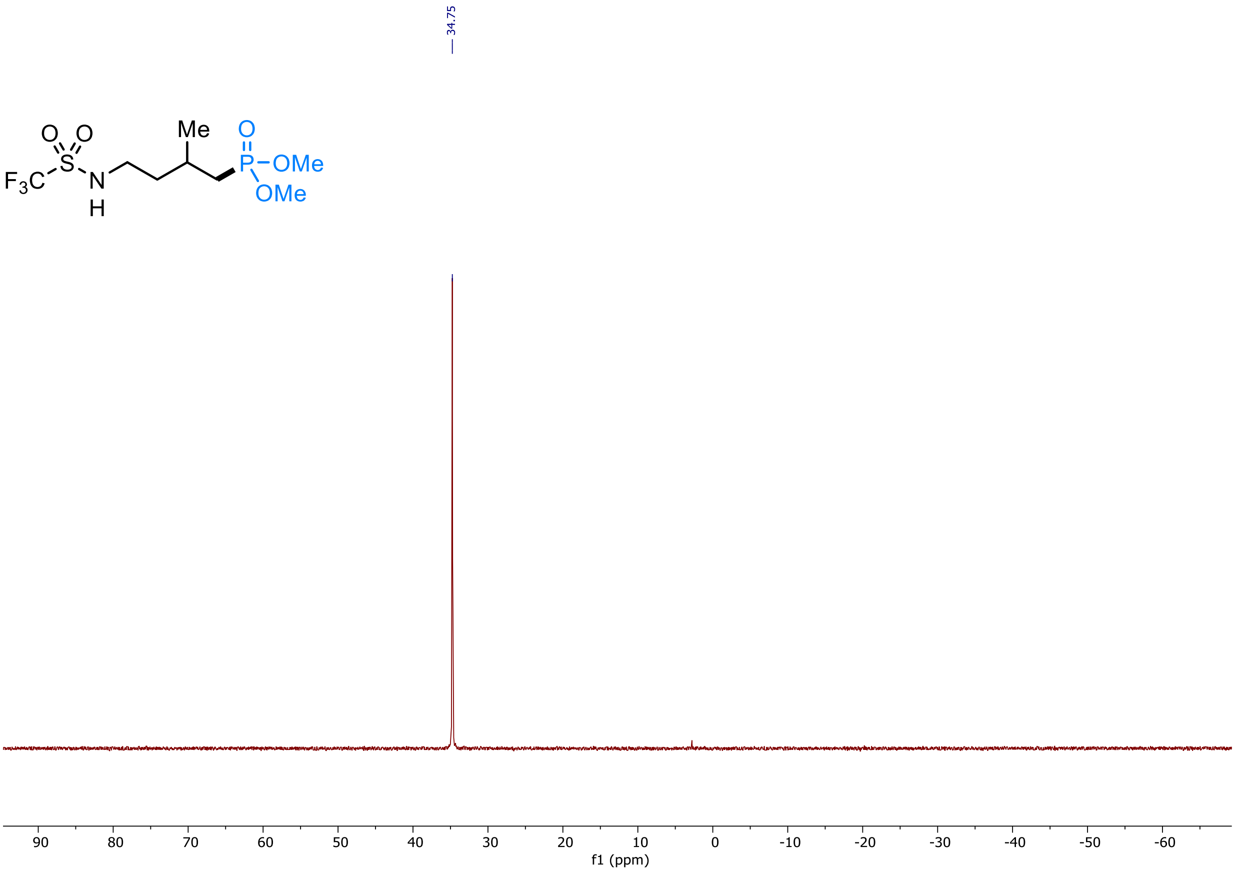

^{19}F NMR (377 MHz, CDCl$_3$) of **33**

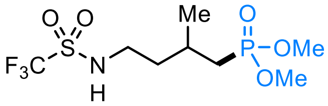

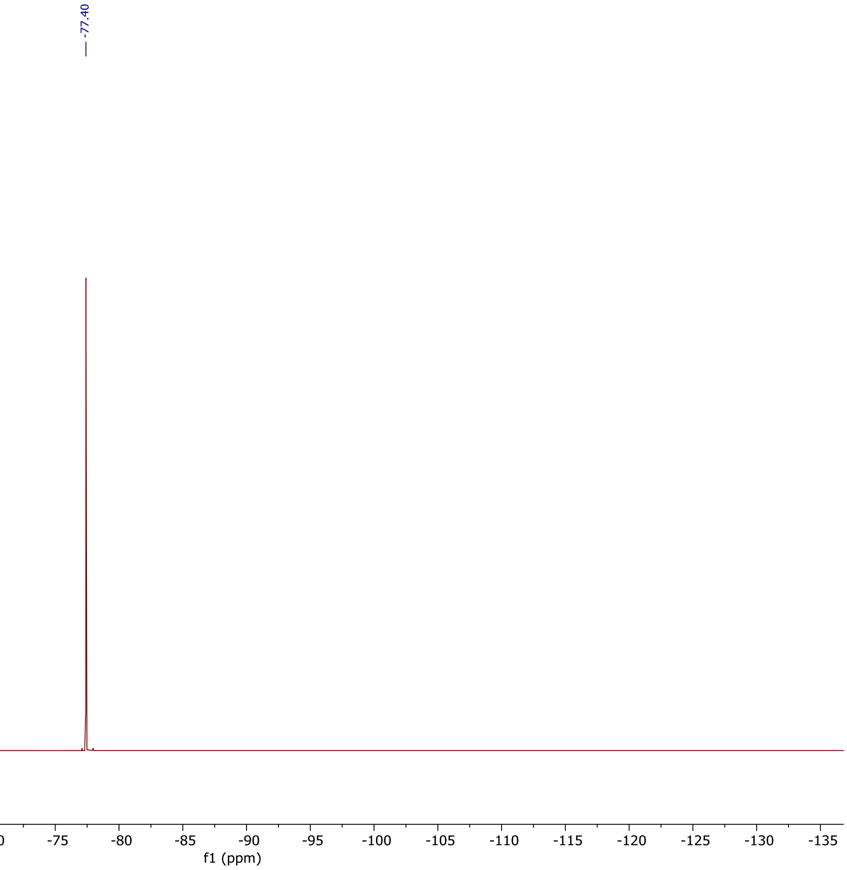

^{31}P NMR of crude reaction mixture of **34** (*see procedure*)

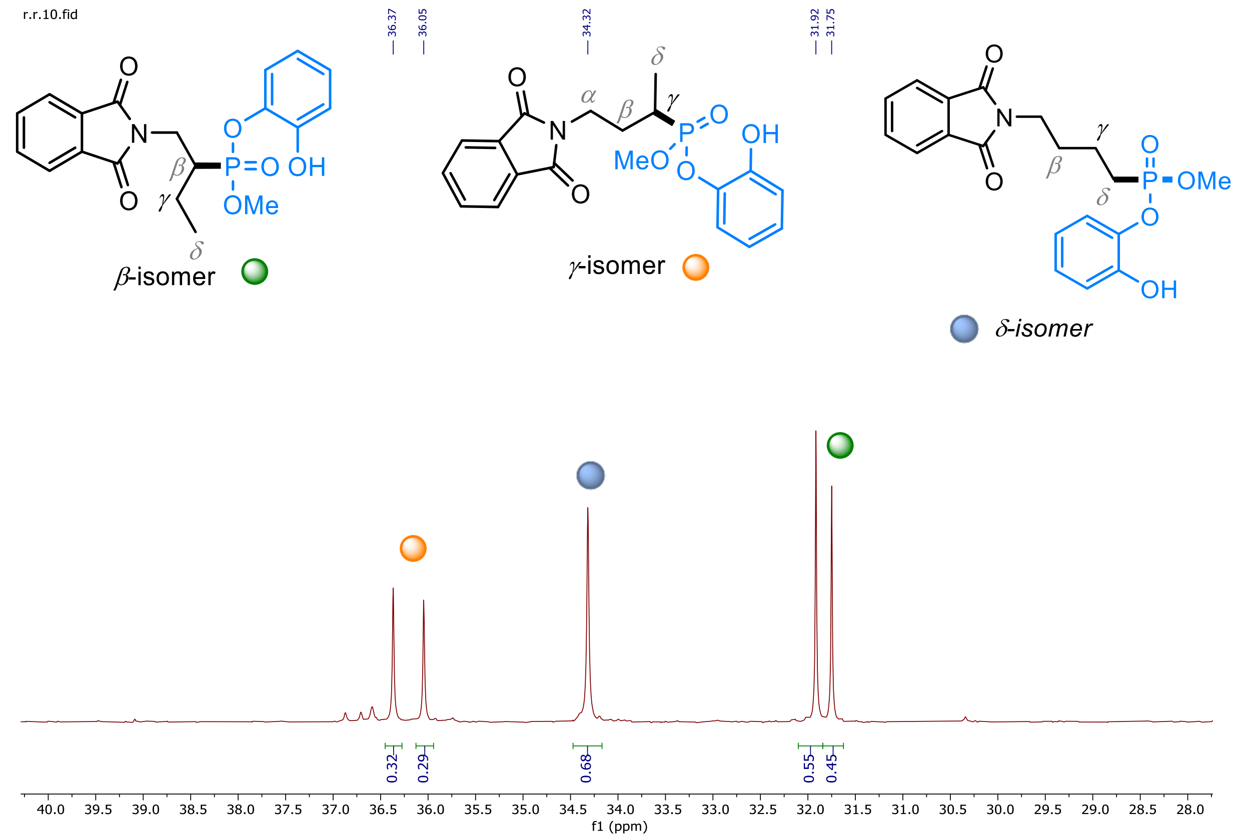

^{1}H NMR (600 MHz, CDCl$_3$) of **34** (*see procedure*)

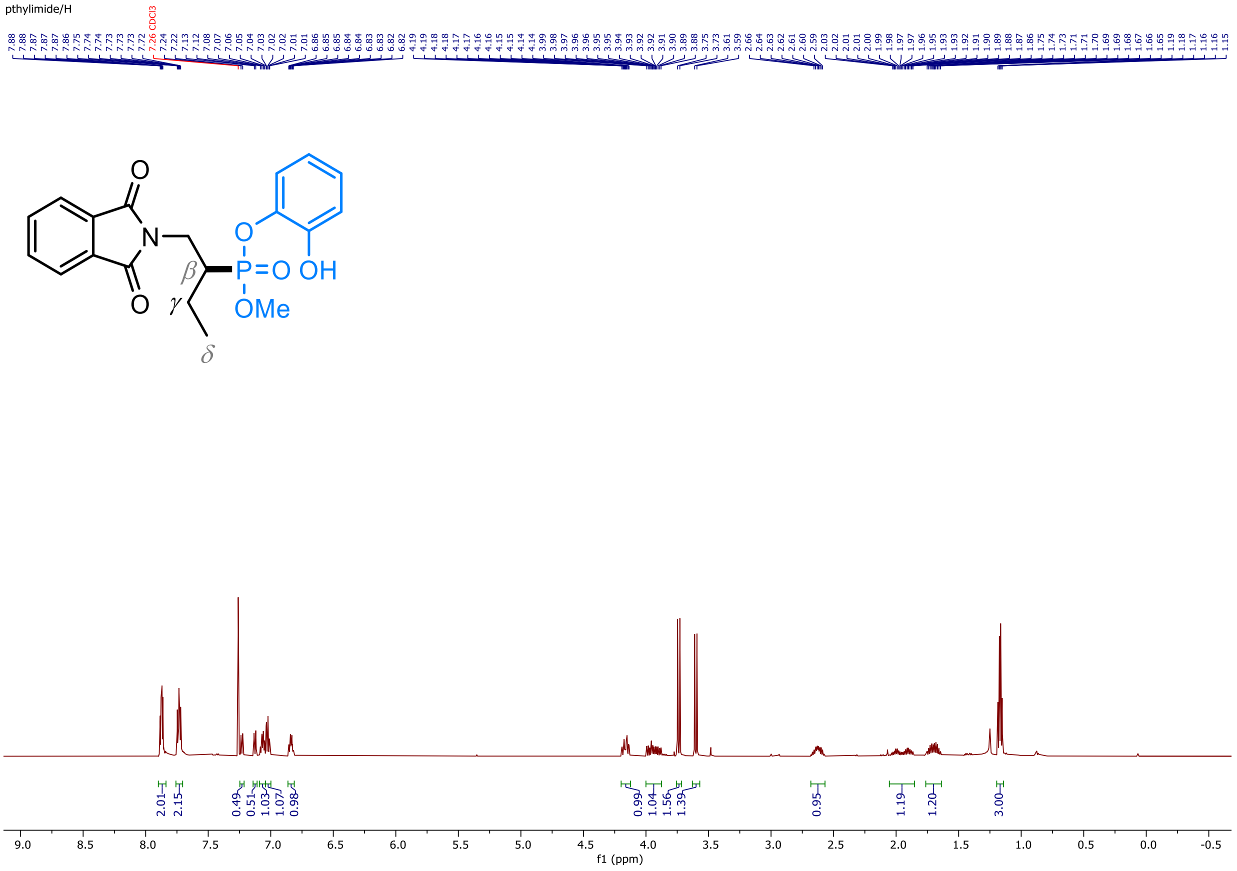

^{13}C NMR (101 MHz, CDCl$_3$) of **34**

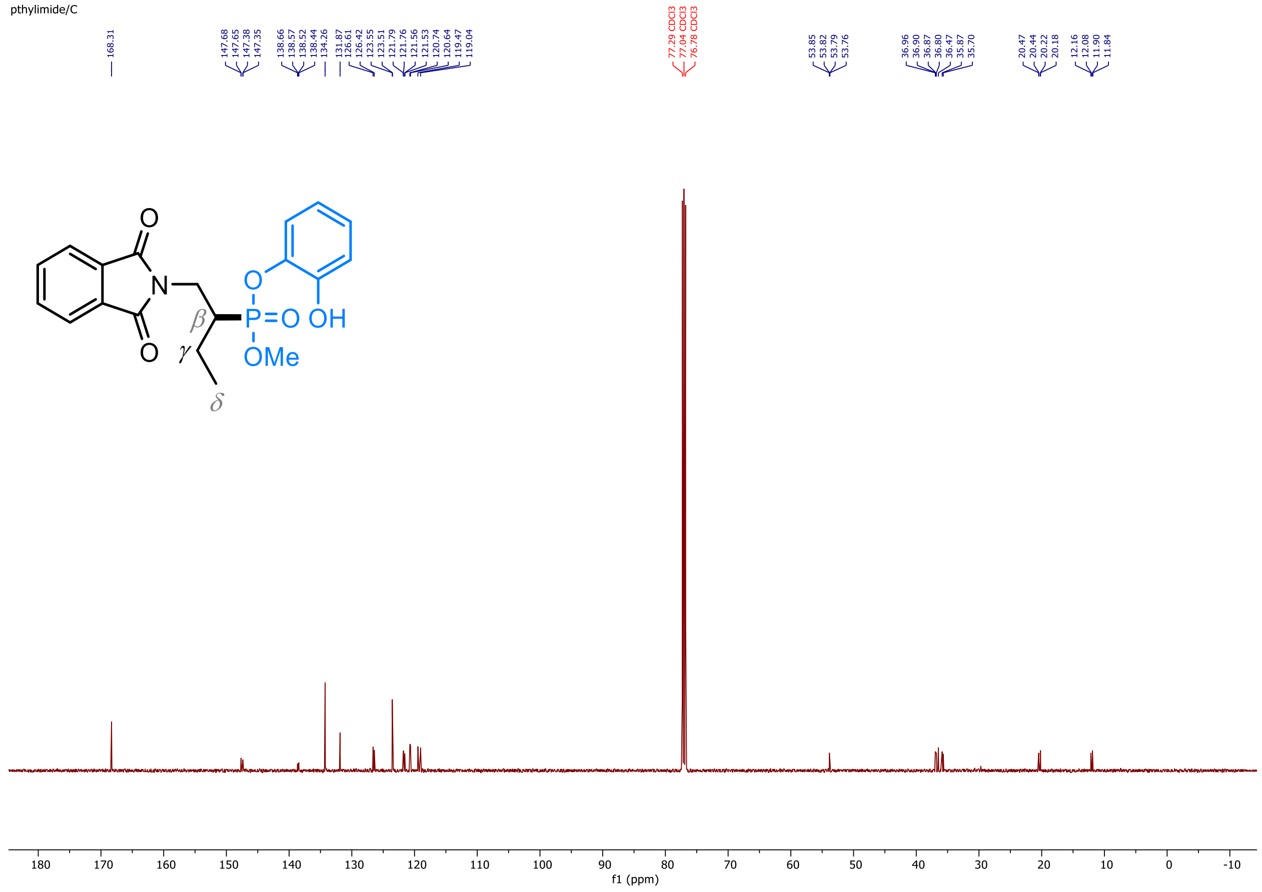

^{31}P NMR (162 MHz, CDCl$_3$) of **34**

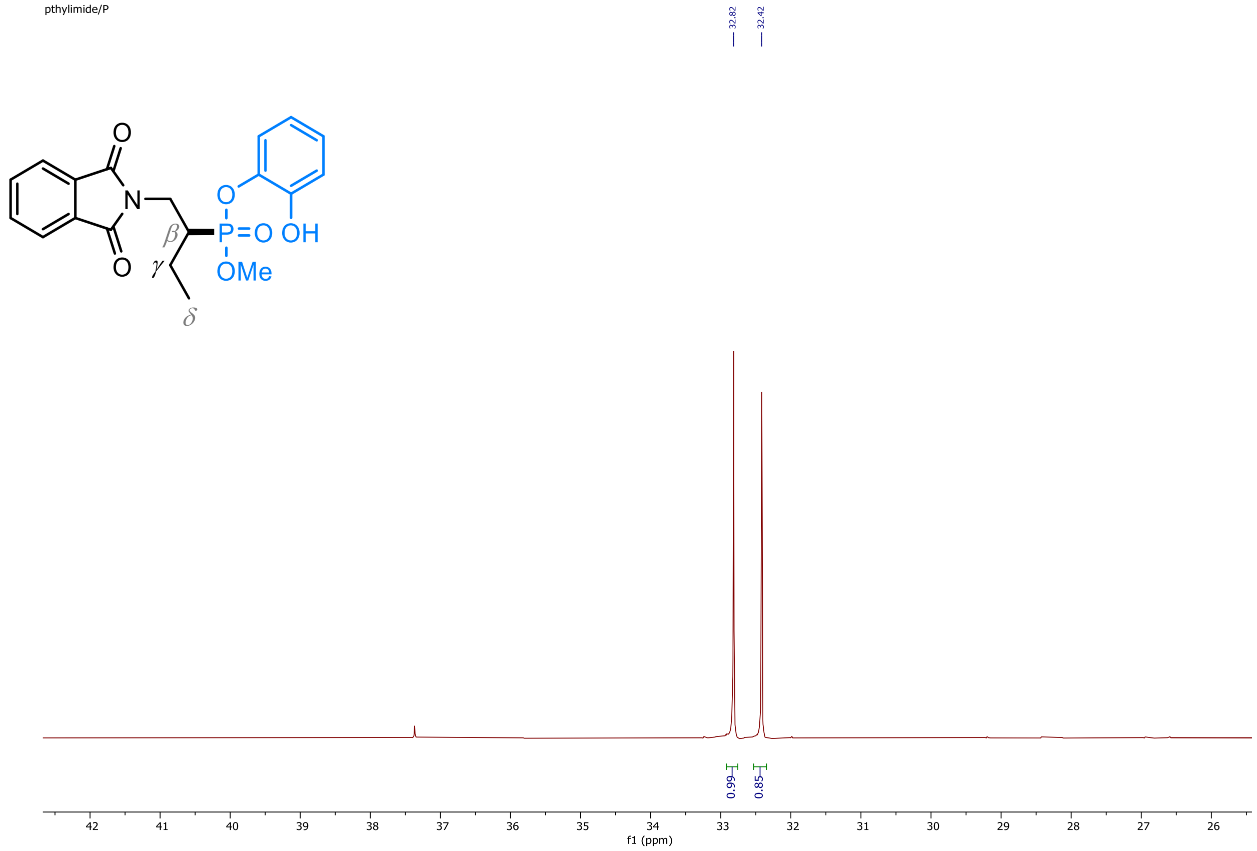

^{1}H-^{1}H COSY (600MHz, $CDCl_3$) of **34**

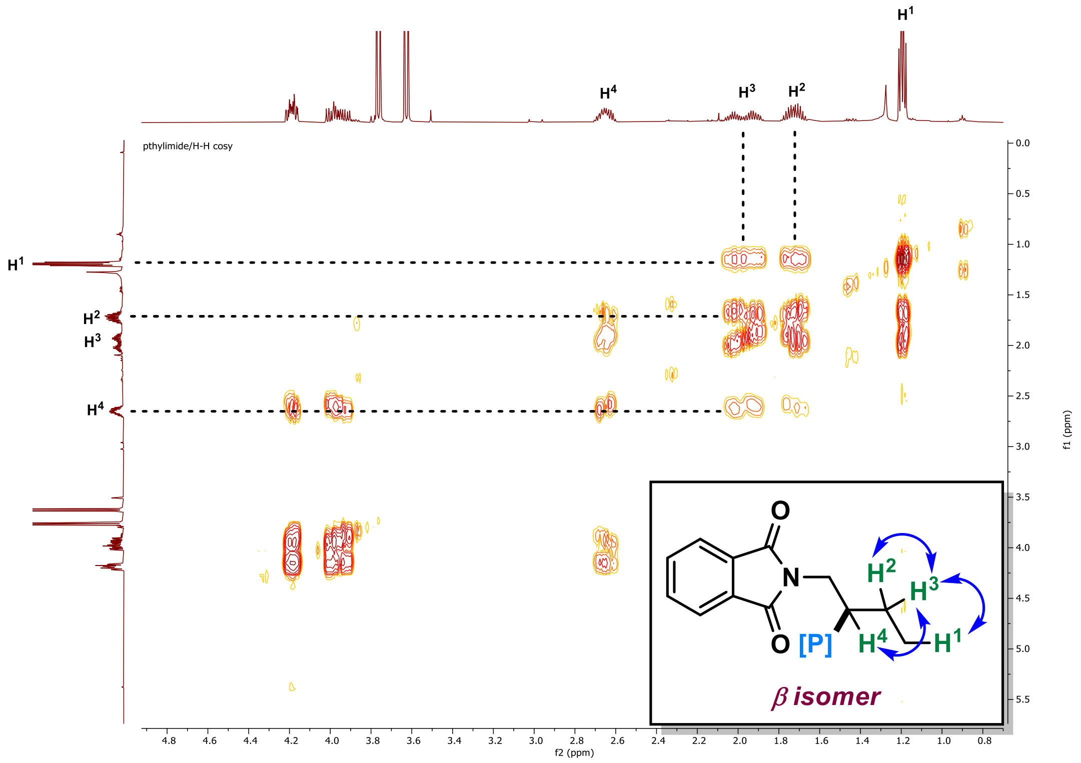

^{1}H NMR (400 MHz, $CDCl_3$) of **35** (*see procedure*)

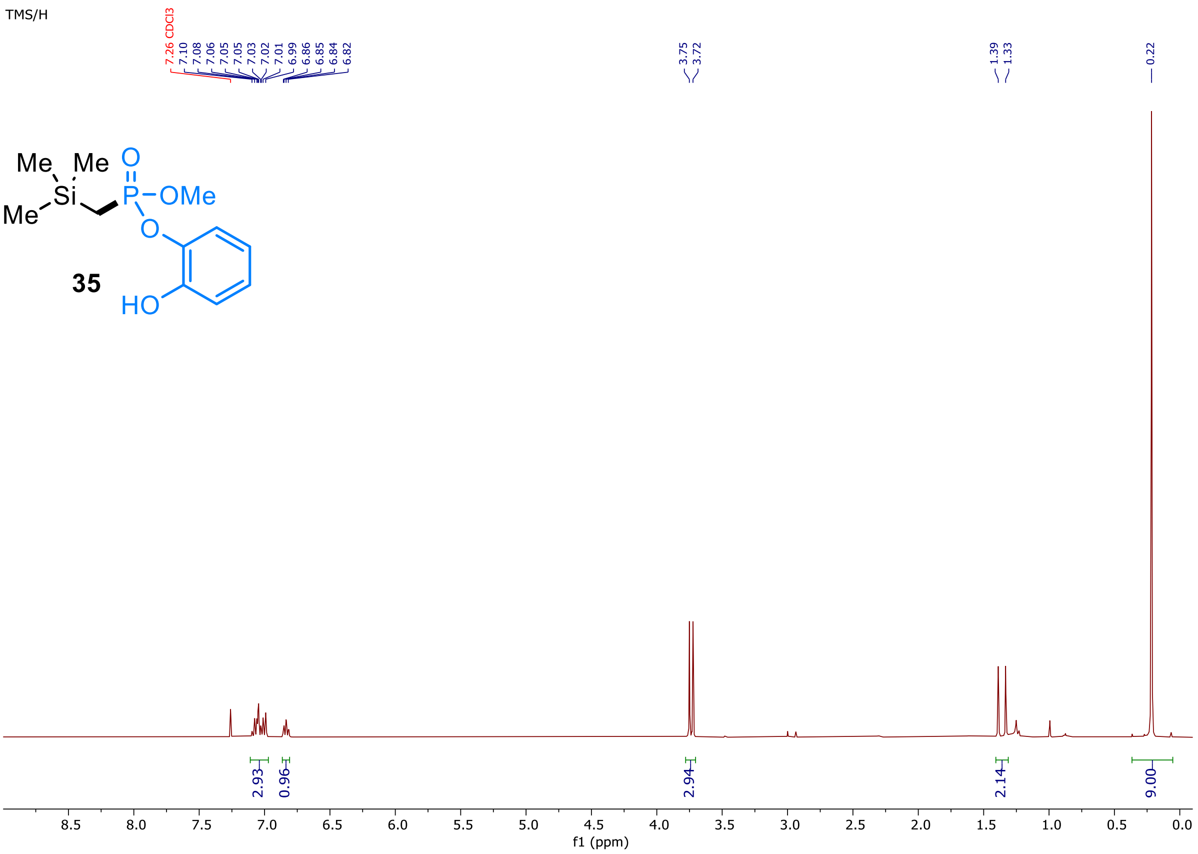

^{13}C NMR (101 MHz, $CDCl_3$) of **35**

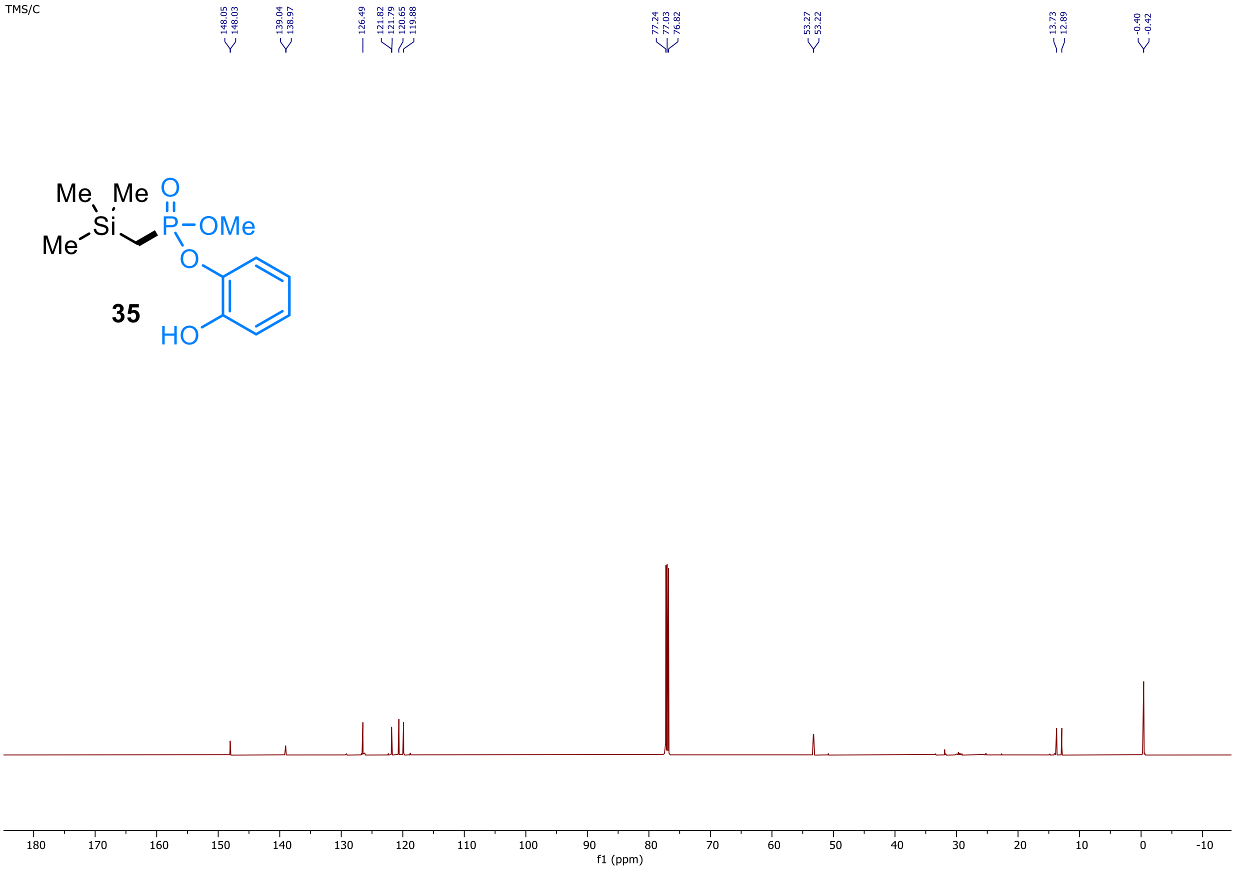

^{31}P NMR (162 MHz, $CDCl_3$) of **35**

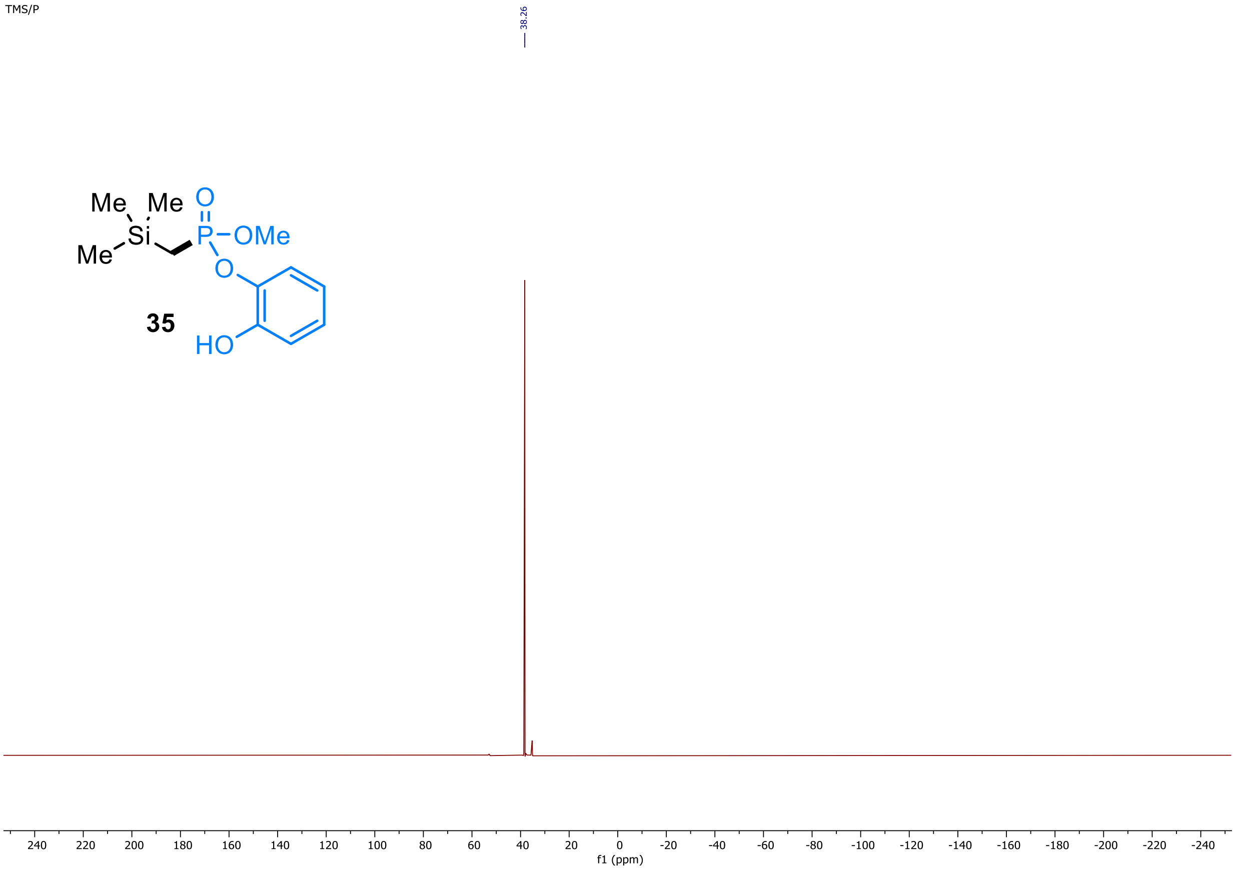

^{1}H NMR (400 MHz, $CDCl_3$) of **36** (*see procedure*)

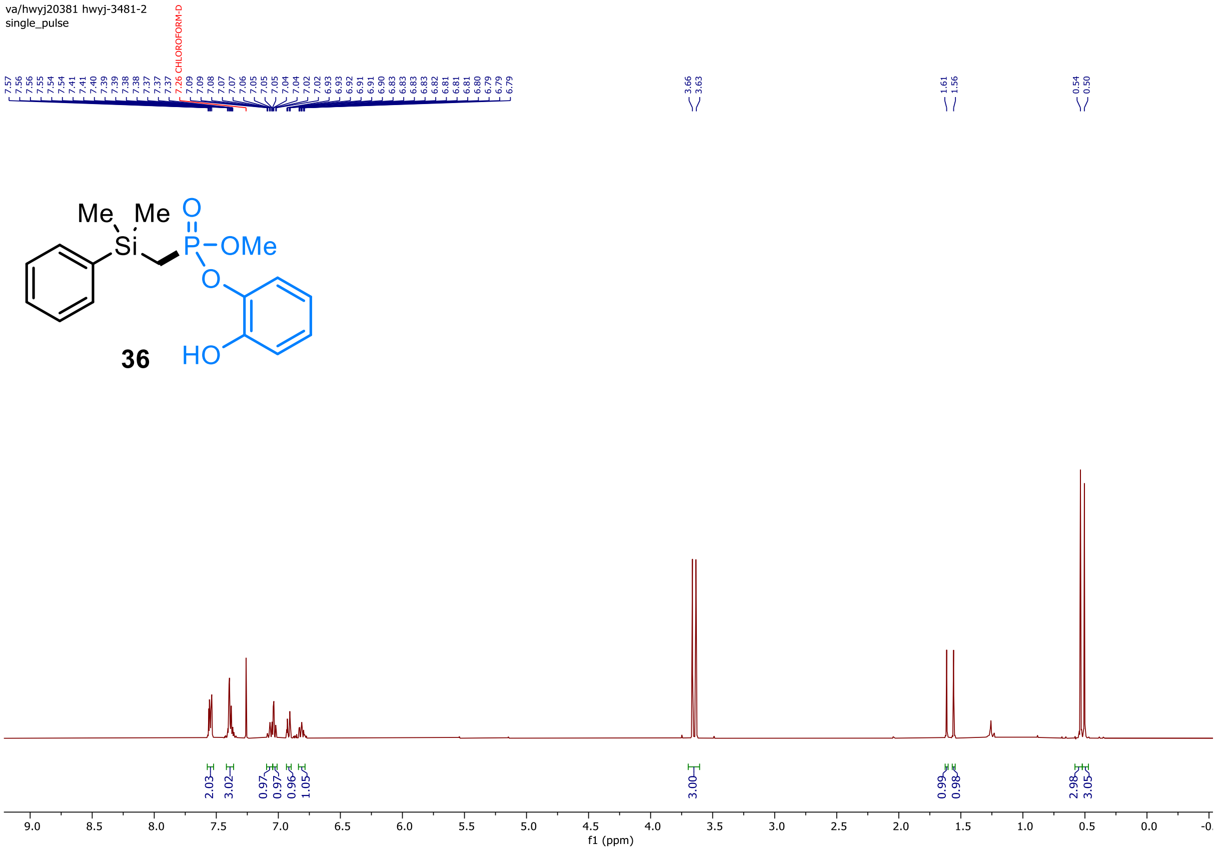

^{13}C NMR (101 MHz, $CDCl_3$) of **36**

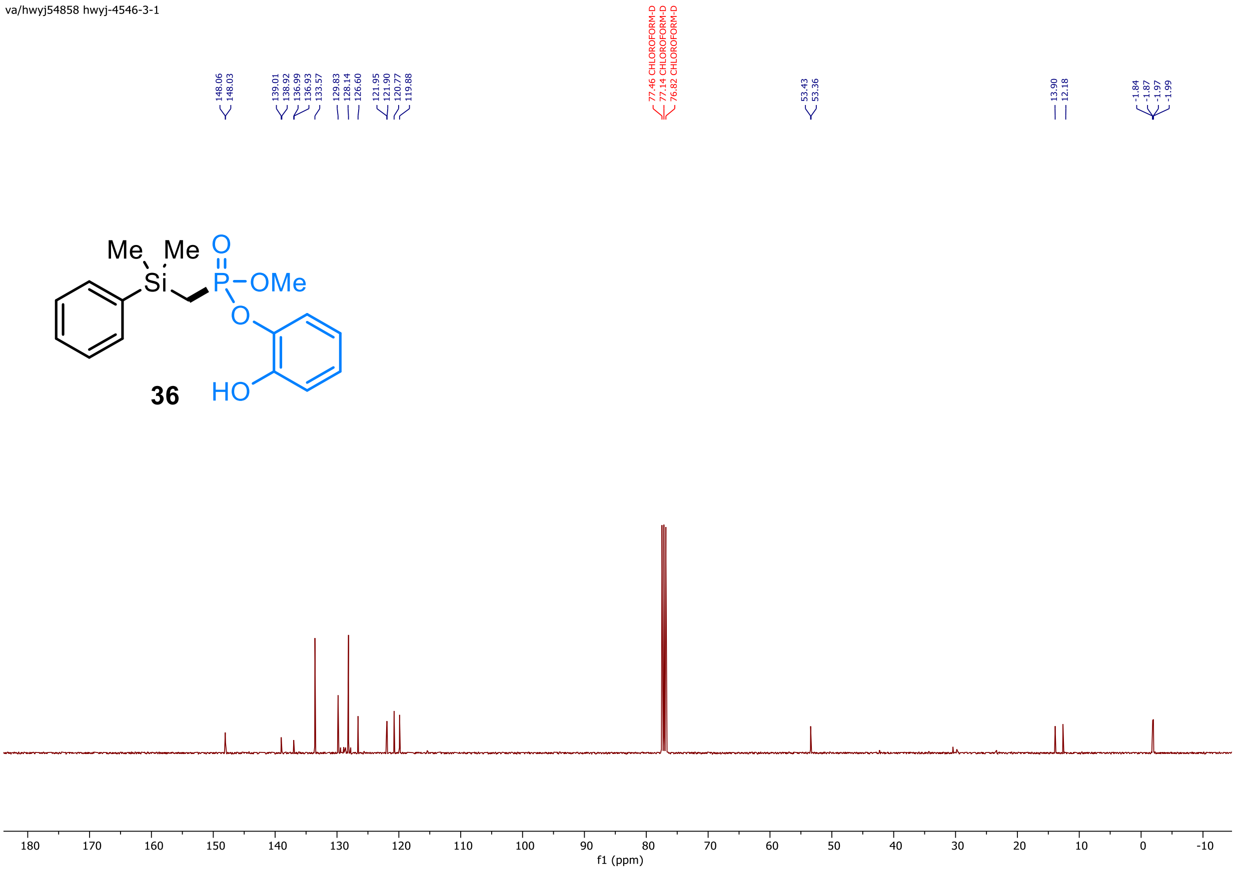

^{31}P NMR (162 MHz, $CDCl_3$) of **36**

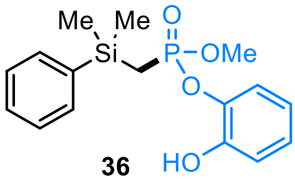

36

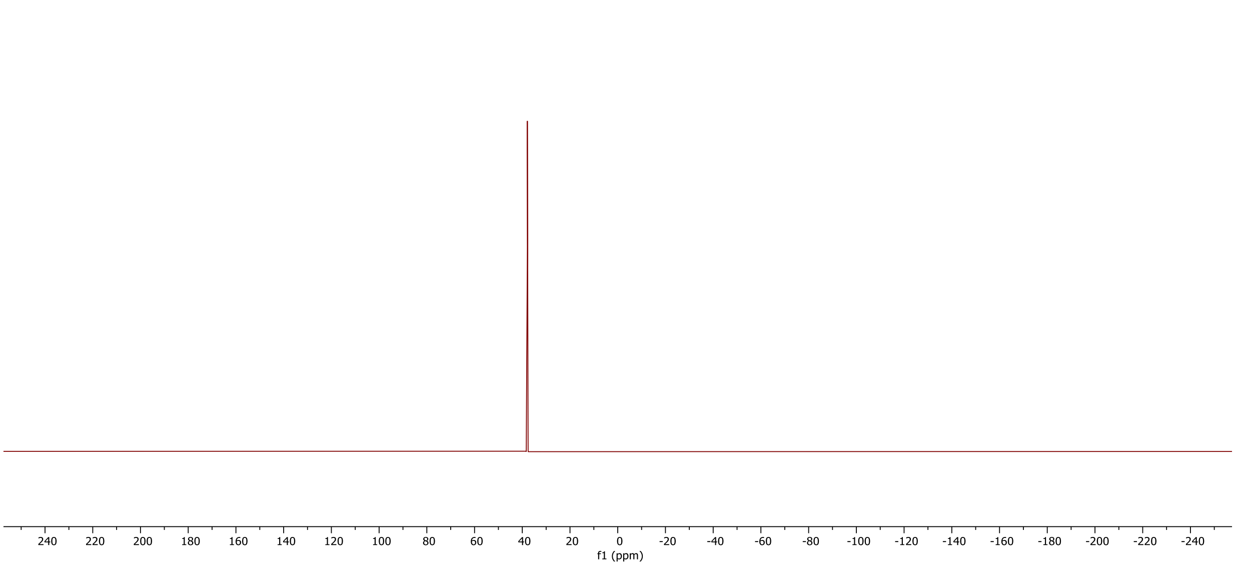

^{31}P NMR (162 MHz, $CDCl_3$) of crude **37-int** (*see procedure*)

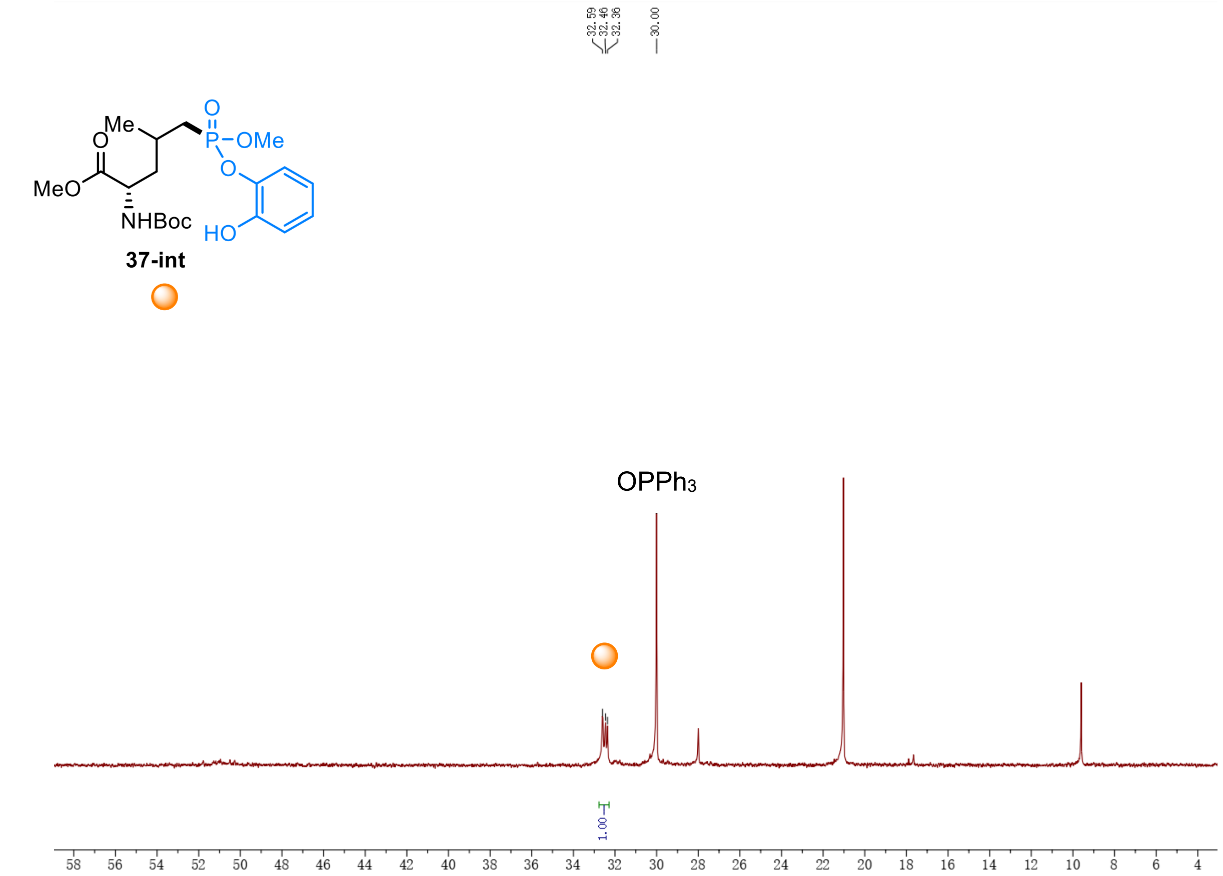

^{1}H NMR (400 MHz, $CDCl_3$) of **37** (*see procedure*)

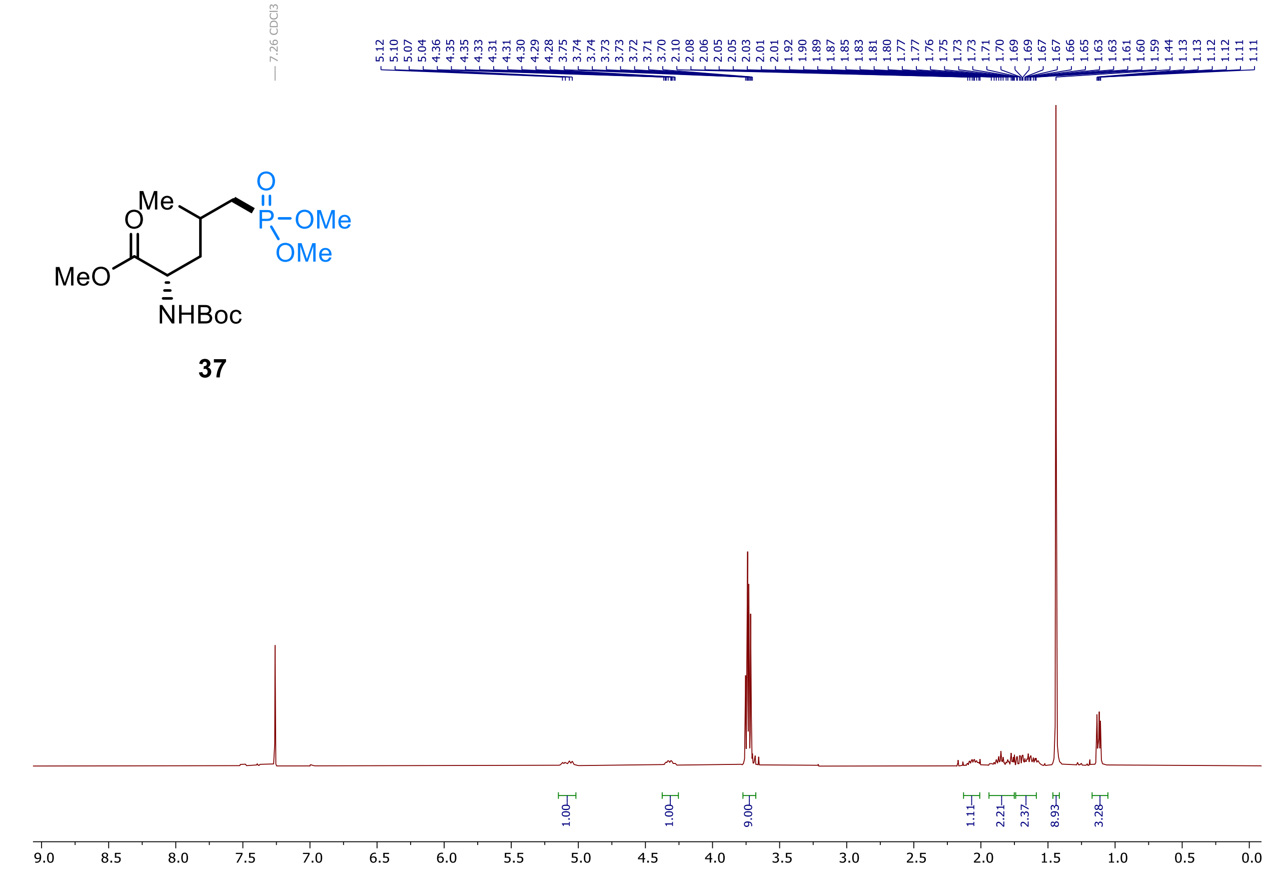

^{13}C NMR (151 MHz, $CDCl_3$) of **37**

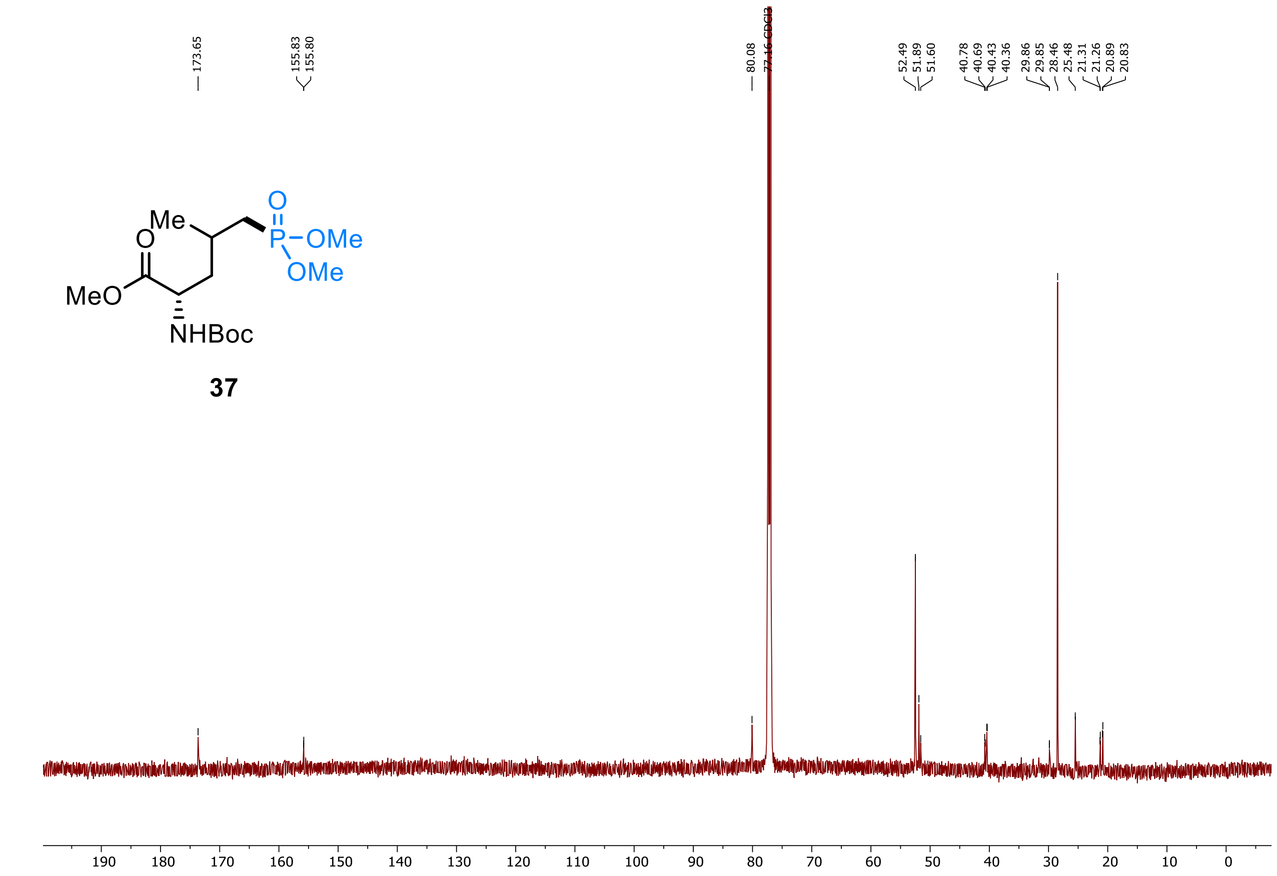

^{31}P NMR (162 MHz, $CDCl_3$) of **37**

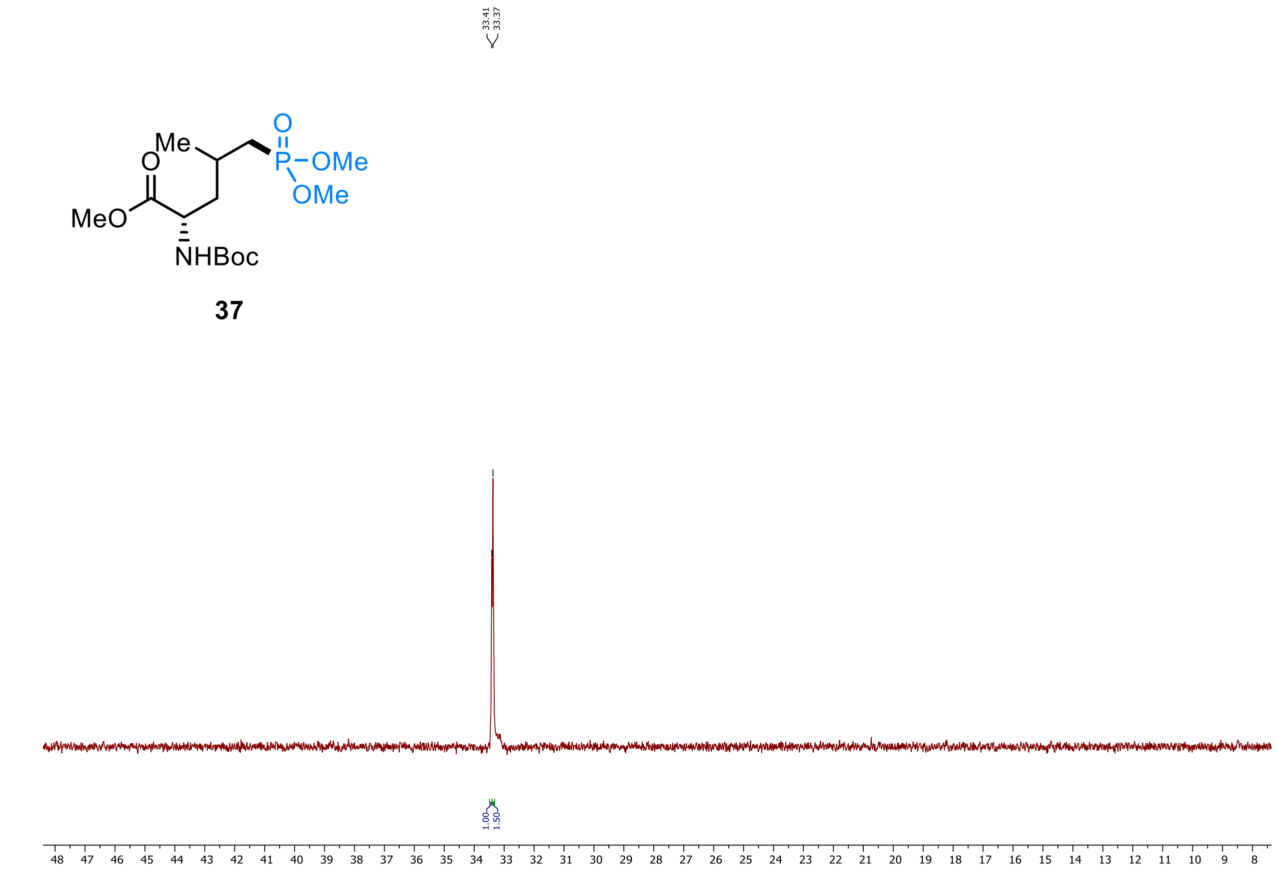

^{1}H NMR (400 MHz, $CDCl_3$) of **38** (*see procedure*)

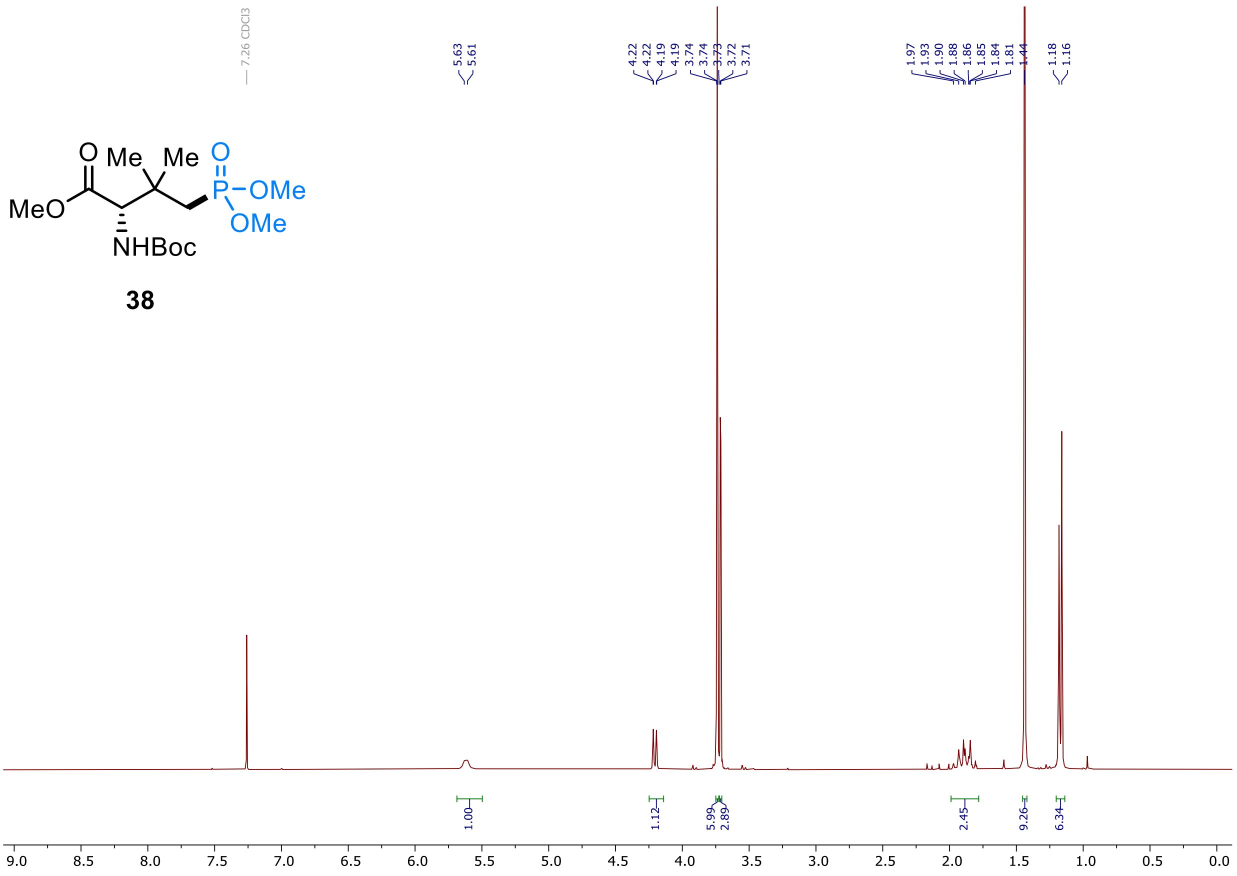

^{13}C NMR (151 MHz, $CDCl_3$) of **38**

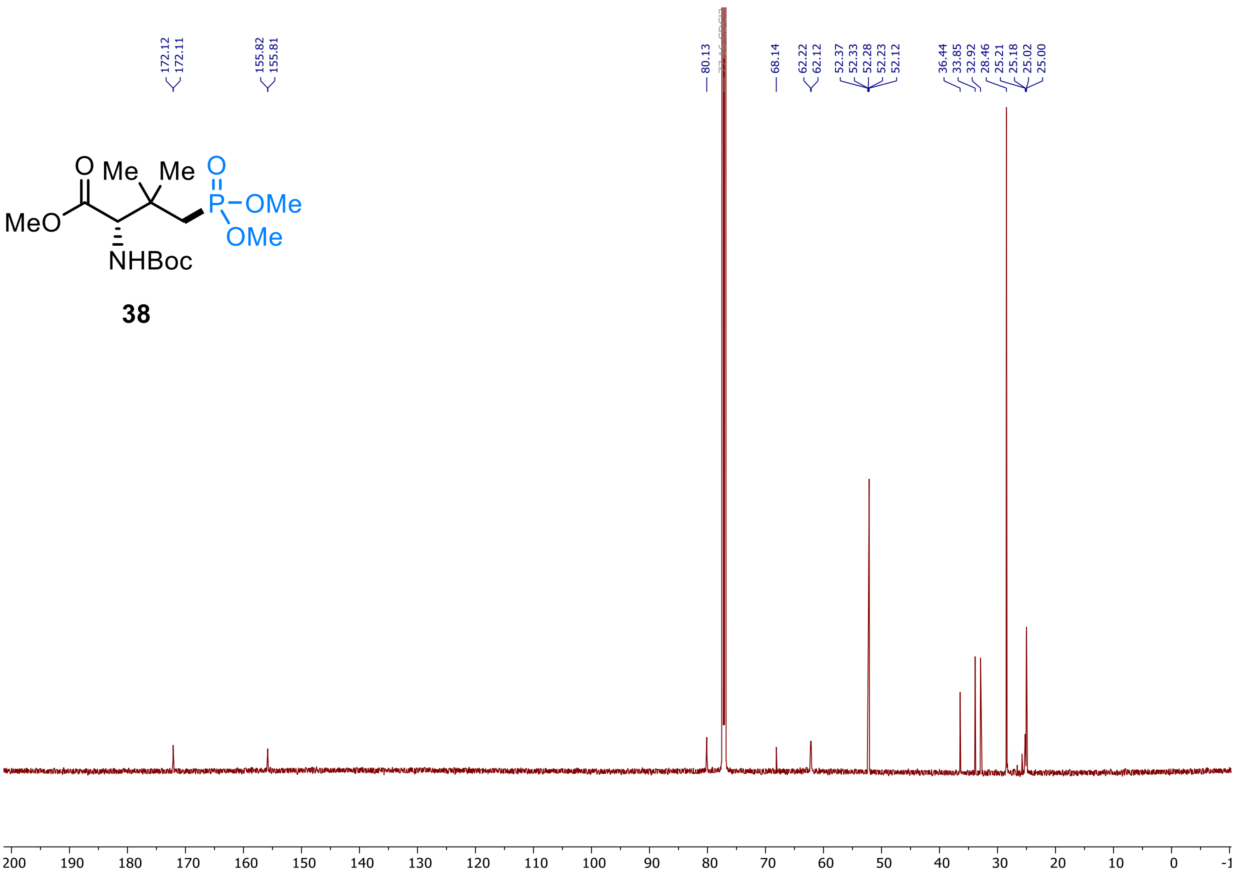

^{31}P NMR (162 MHz, $CDCl_3$) of **38**

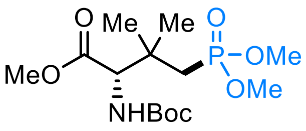

38

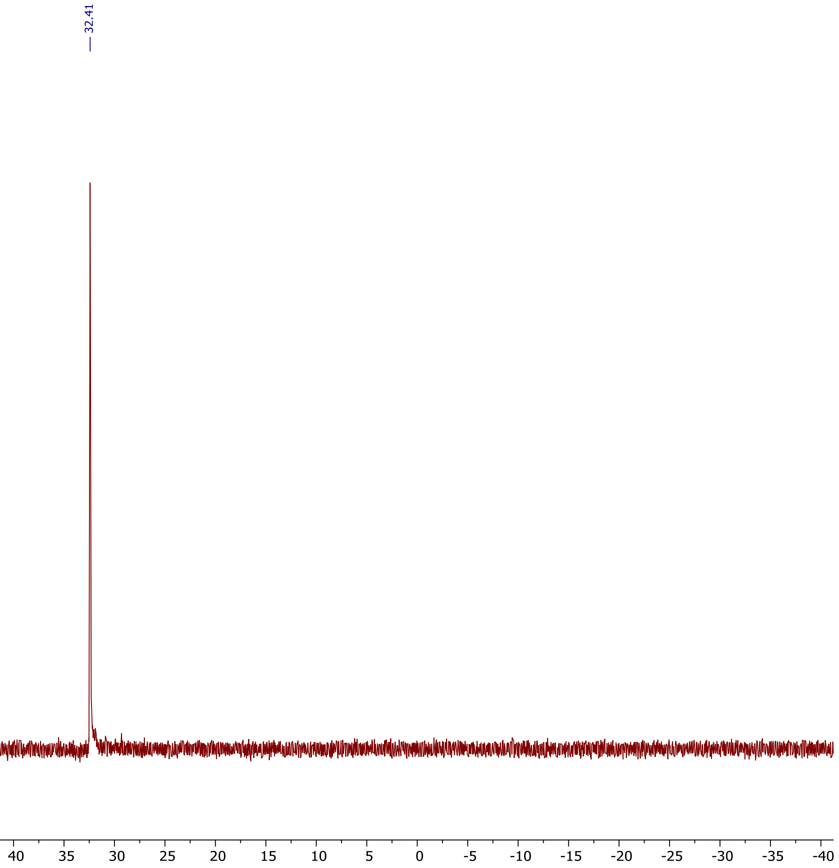

^{31}P NMR (162 MHz, $CDCl_3$) of crude **39-int** (*see procedure*)

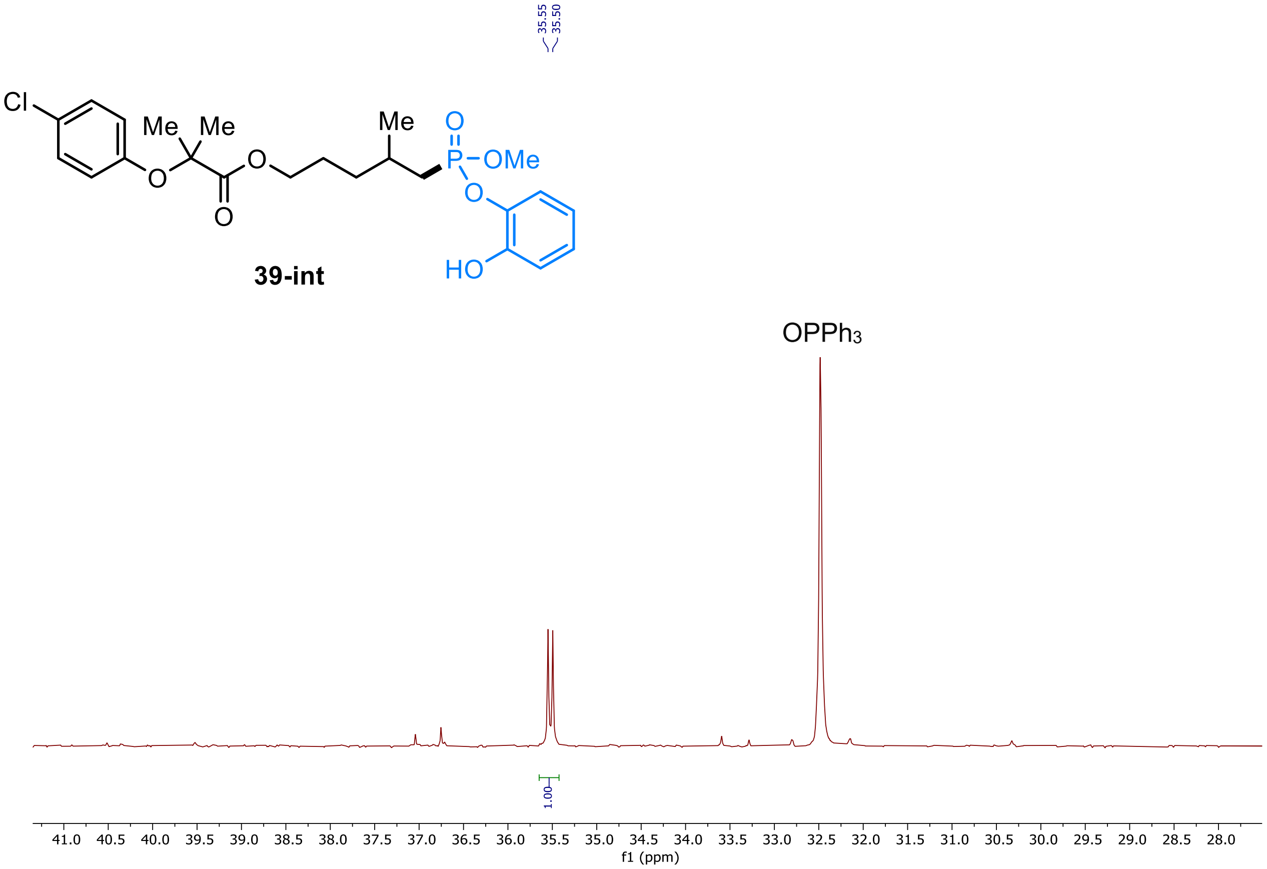

^{1}H NMR (400 MHz, $CDCl_3$) of **39** (*see procedure*)

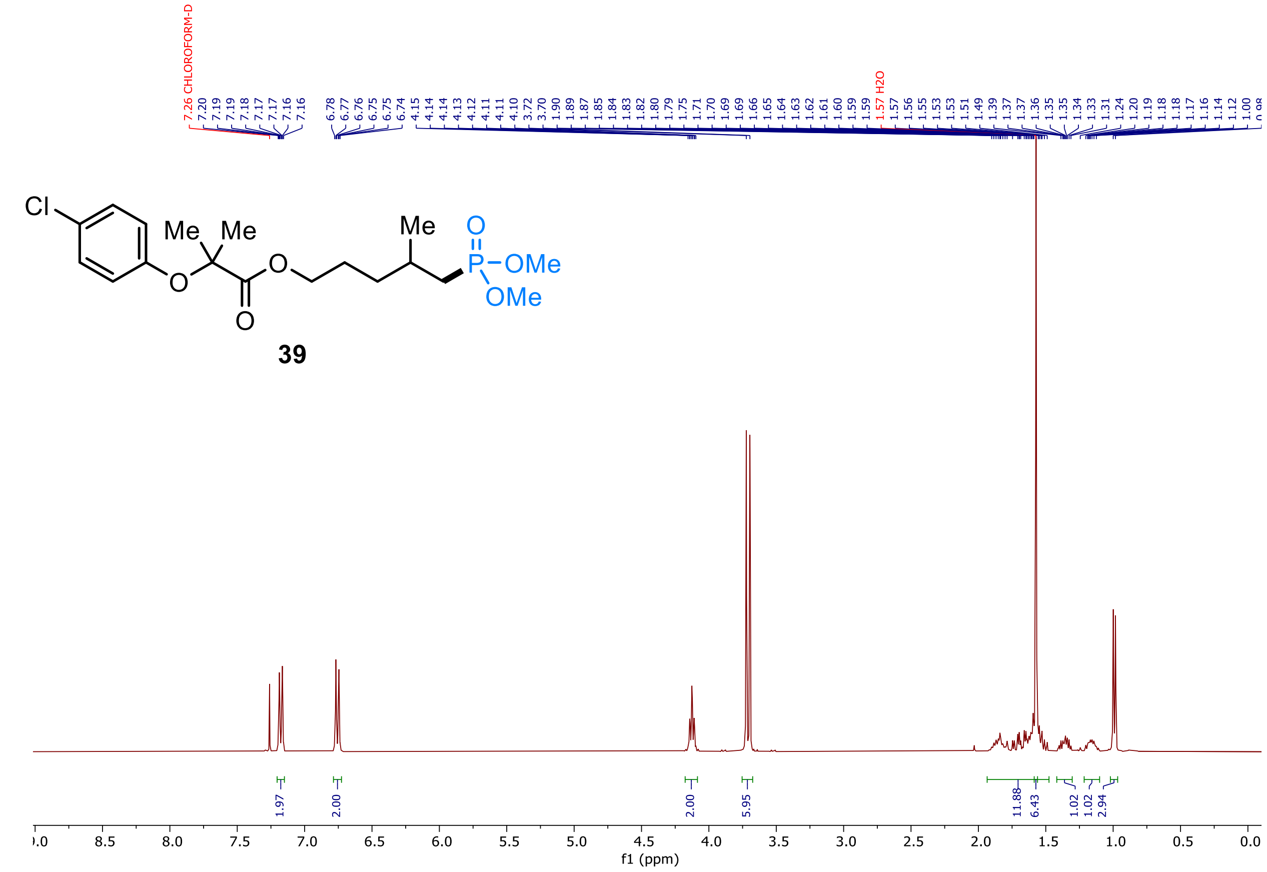

^{13}C NMR (151 MHz, $CDCl_3$) of **39**

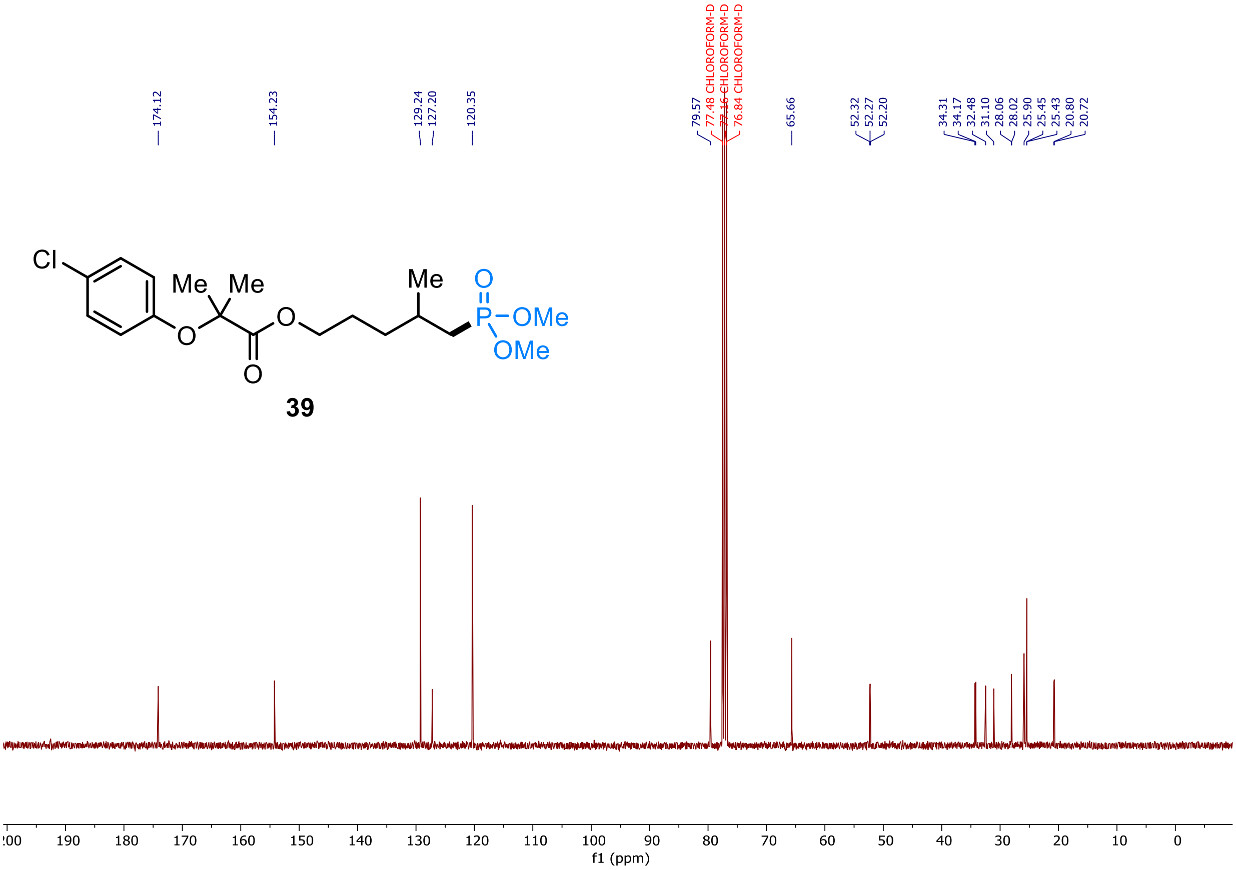

^{31}P NMR (162 MHz, $CDCl_3$) of **39**

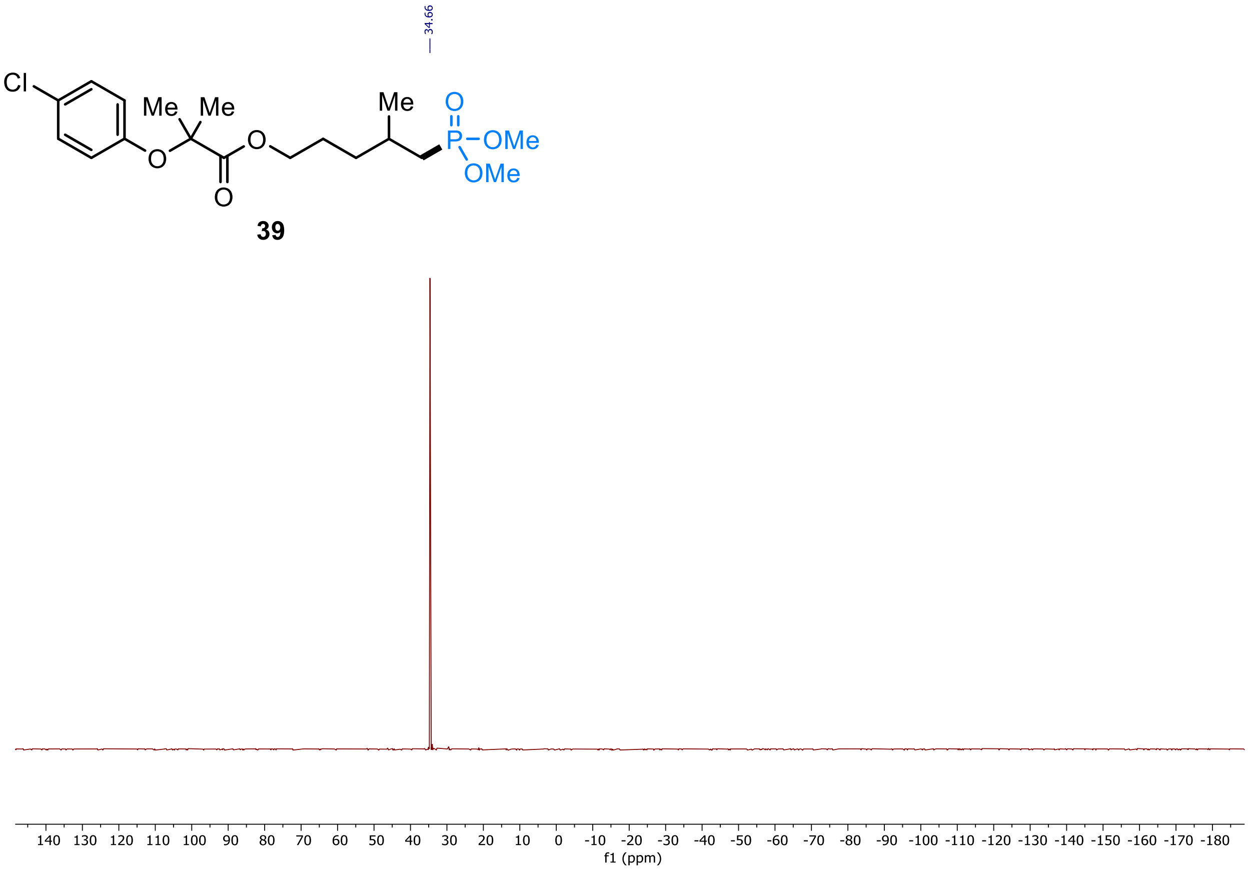

^{31}P NMR (162 MHz, $CDCl_3$) of crude **40-int** (*see procedure*)

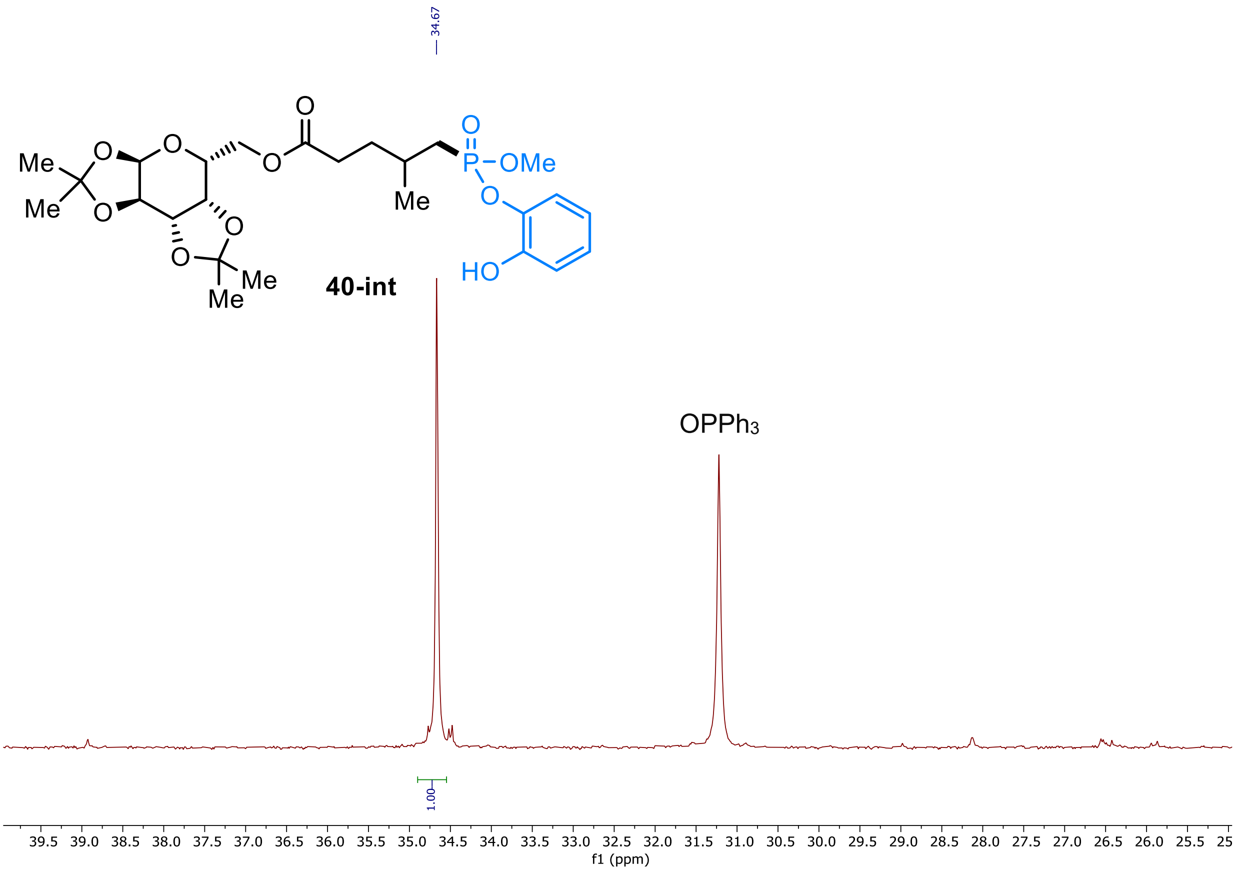

^{1}H NMR (400 MHz, $CDCl_3$) of **40** (*see procedure*)

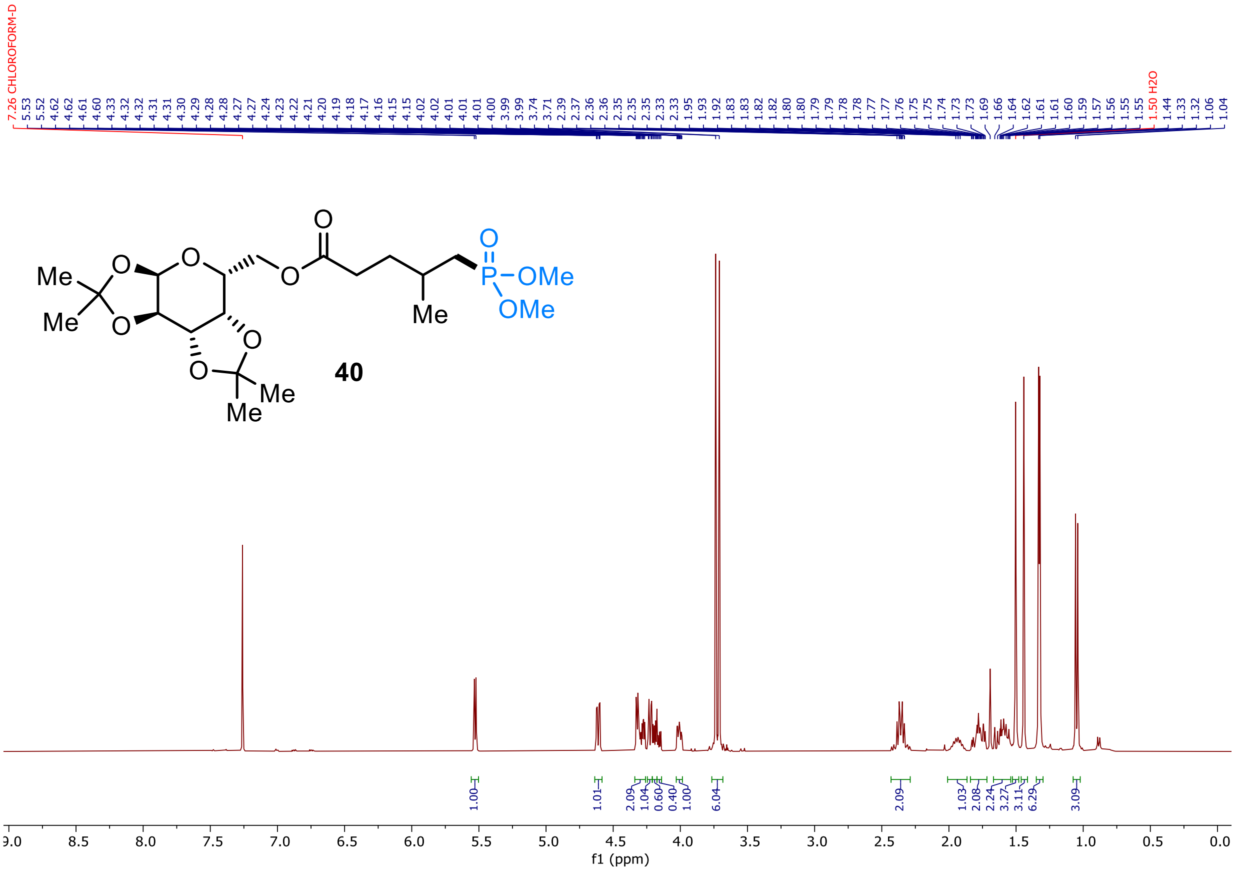

^{13}C NMR (151 MHz, $CDCl_3$) of **40**

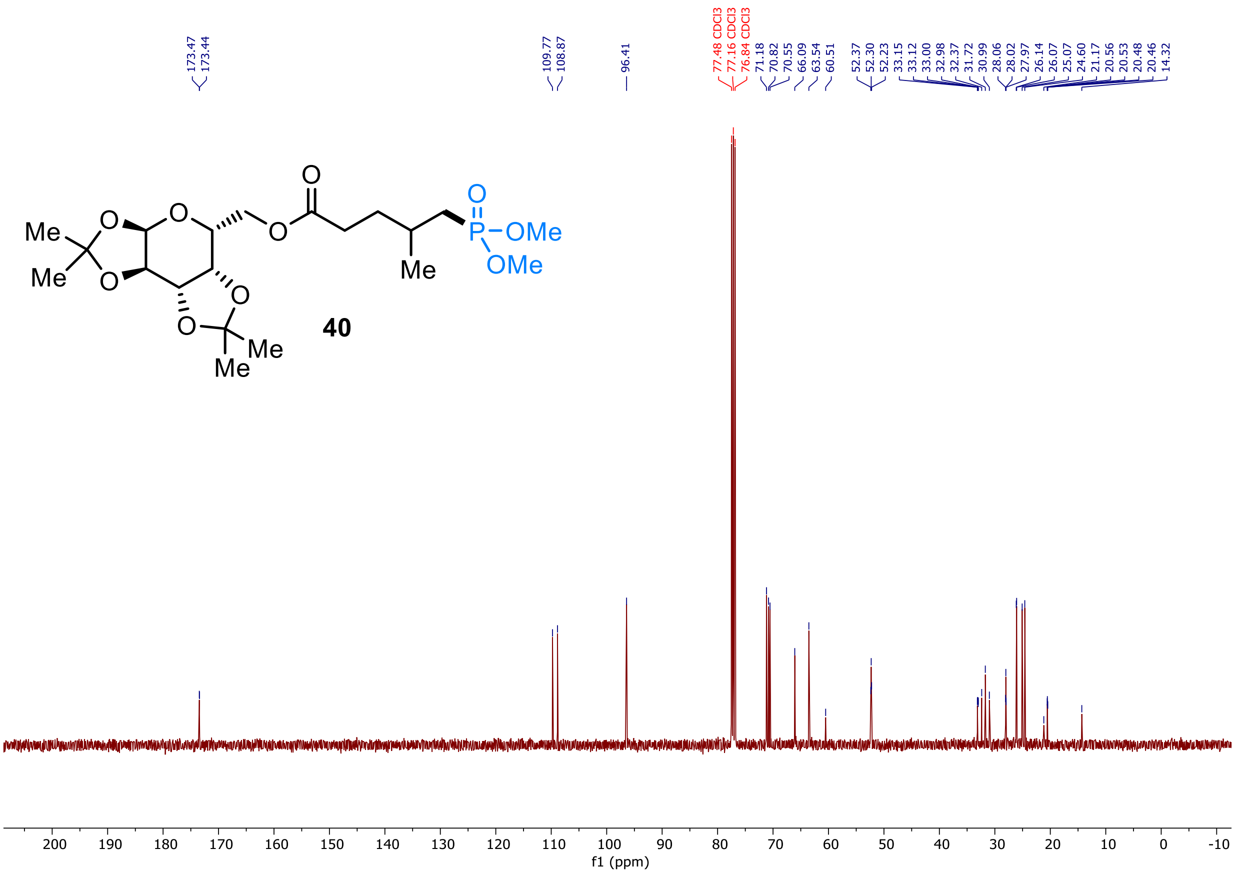

^{31}P NMR (162 MHz, $CDCl_3$) of **40**

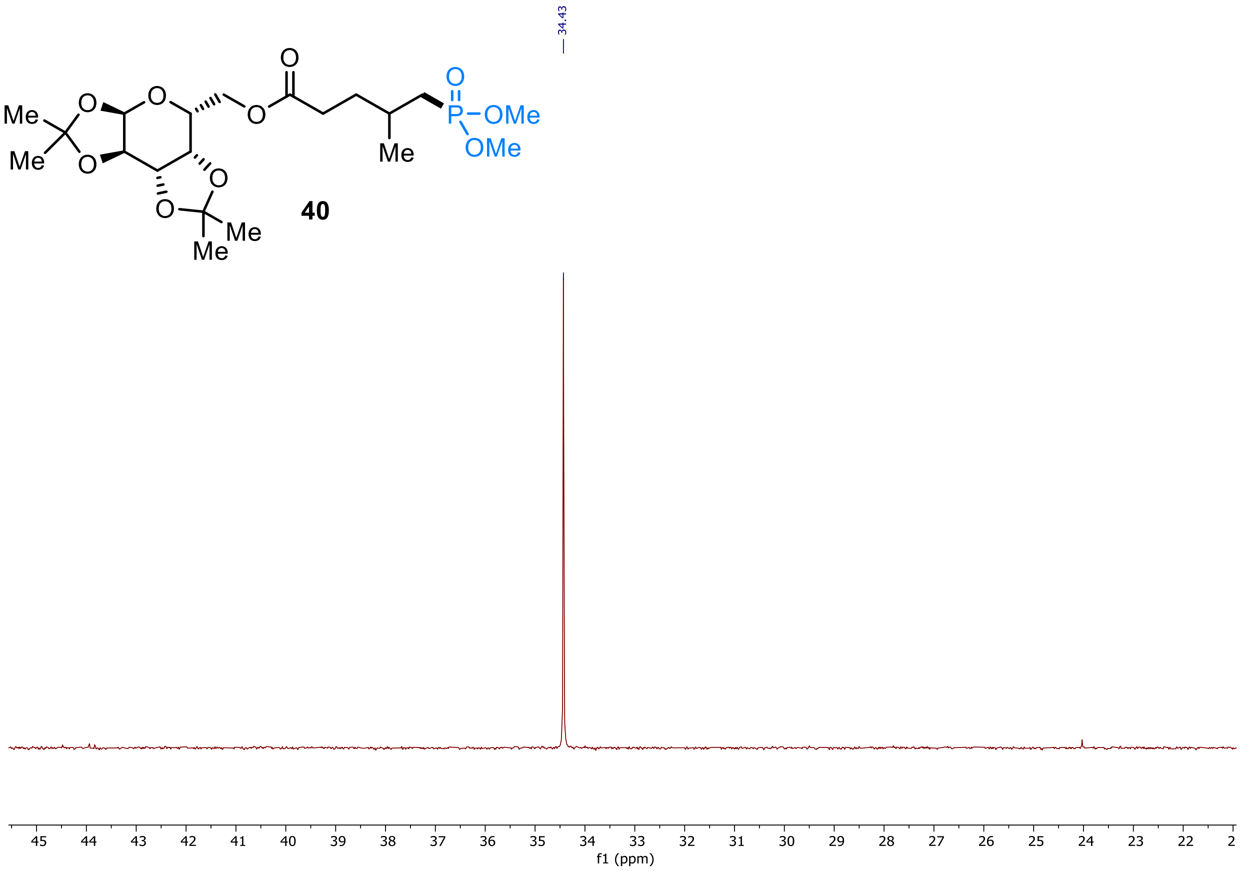

^{1}H NMR (600 MHz, $CDCl_3$) of **41** (*see procedure*)

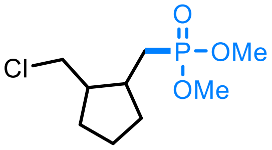

41

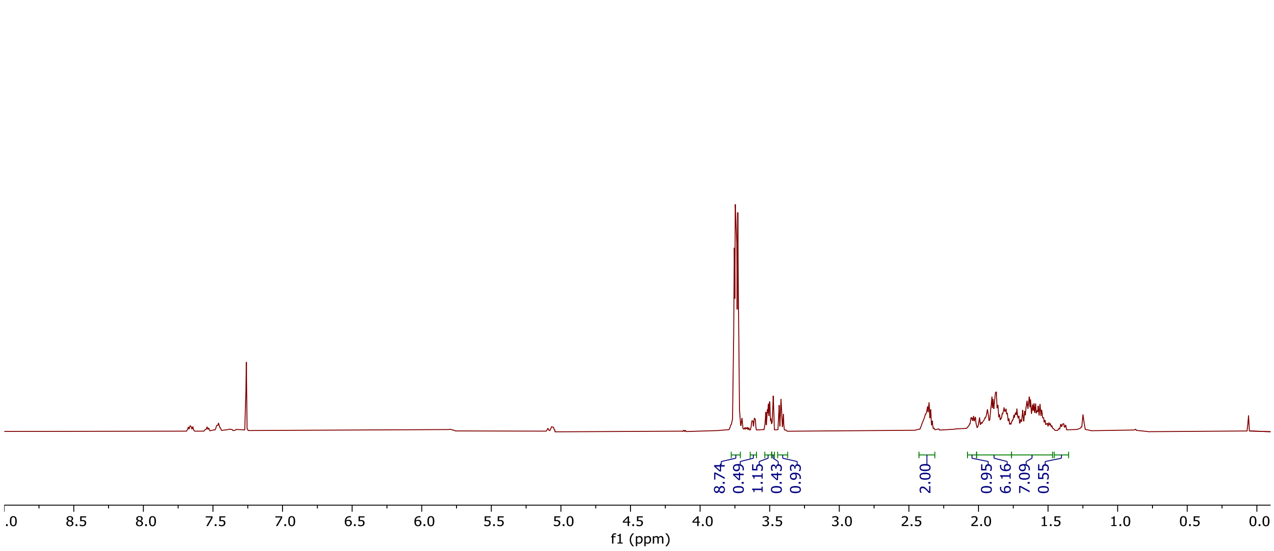

^{13}C NMR (151 MHz, $CDCl_3$) of **41**

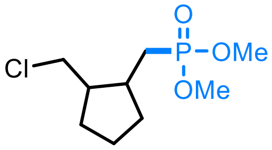

41

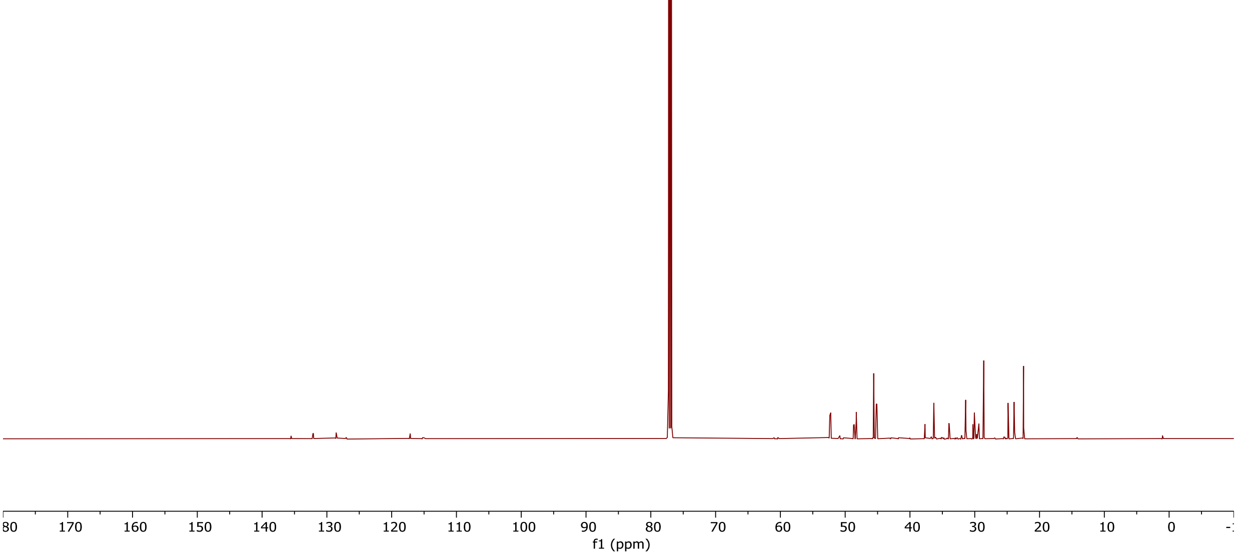

^{31}P NMR (162 MHz, CDCl$_3$) of **41**

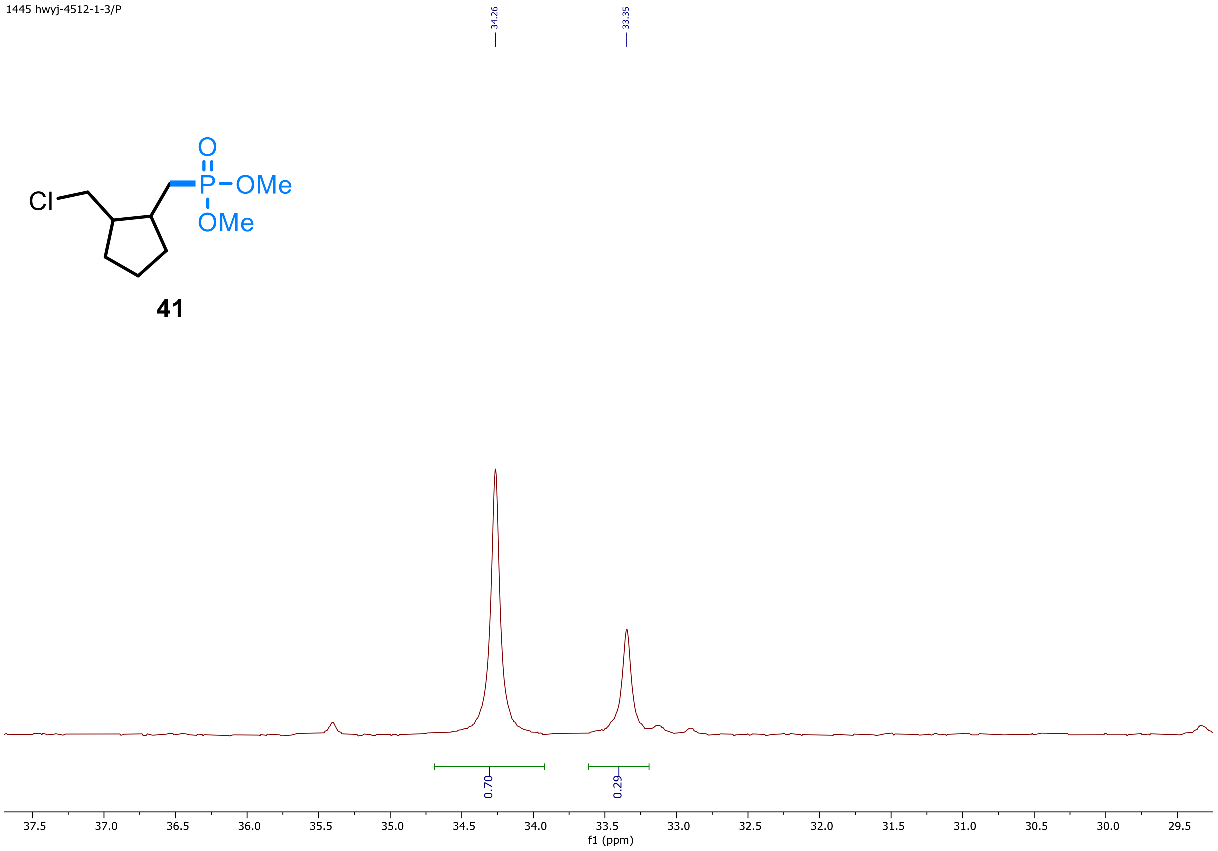

HSQC (600 MHz, CDCl$_3$) of **41**

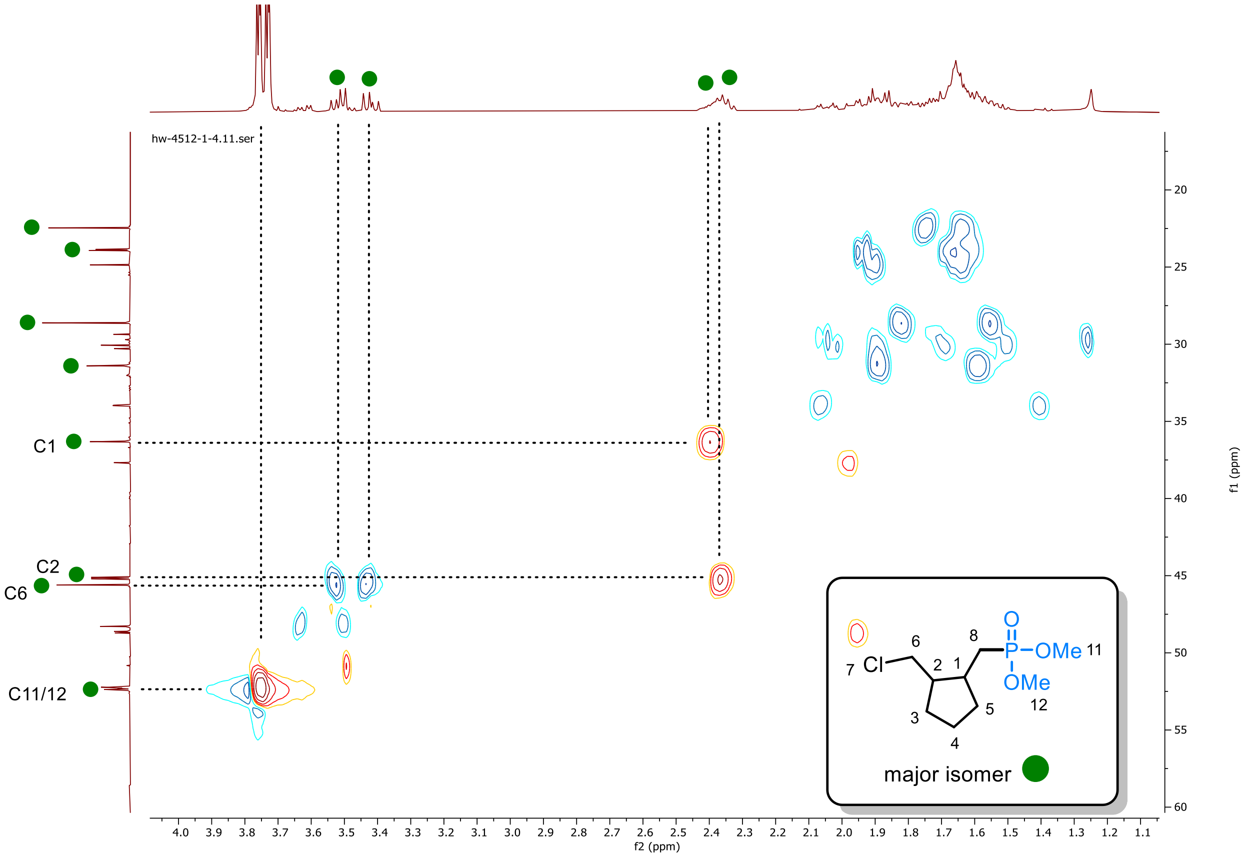

HMBC (600 MHz, $CDCl_3$) of **41**

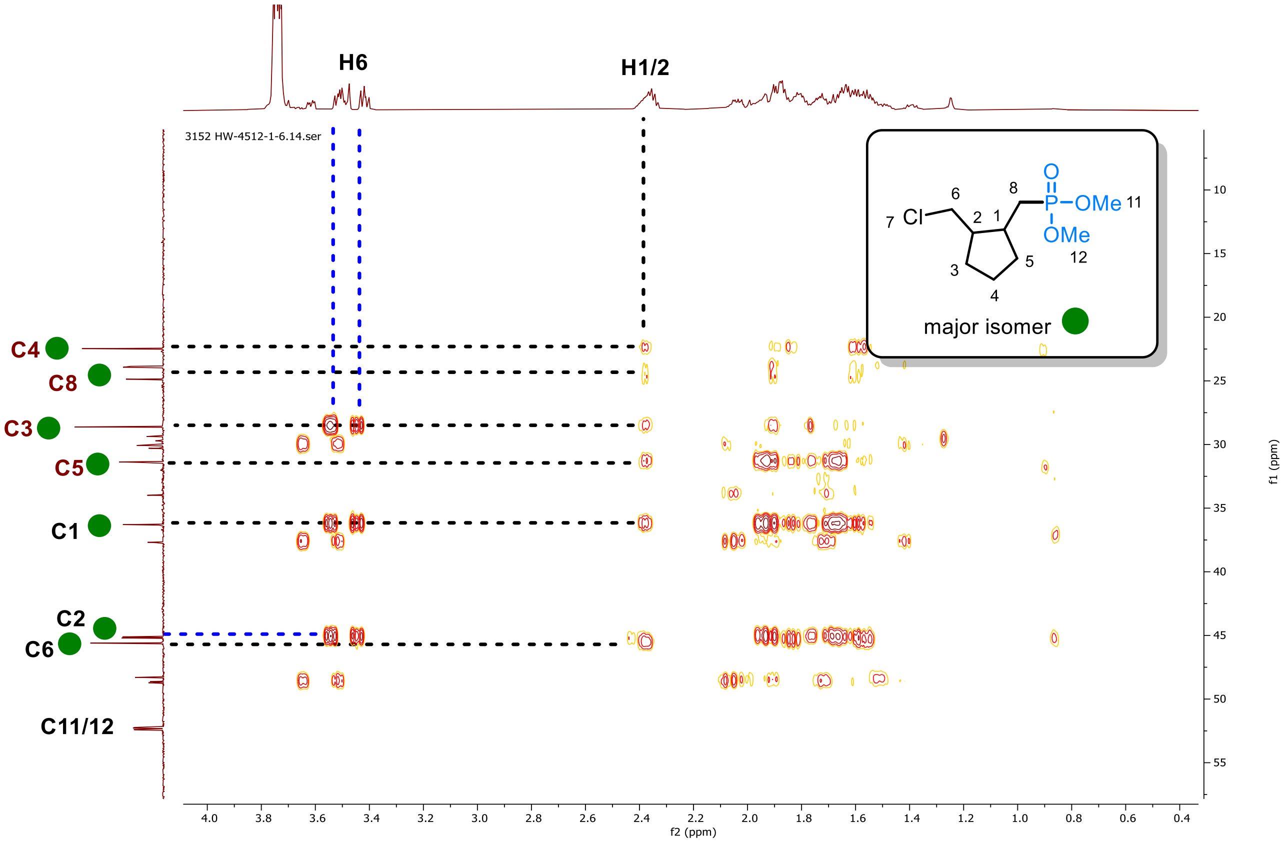

^{1}H NMR (400 MHz, $CDCl_3$) of **42** (*see procedure*)

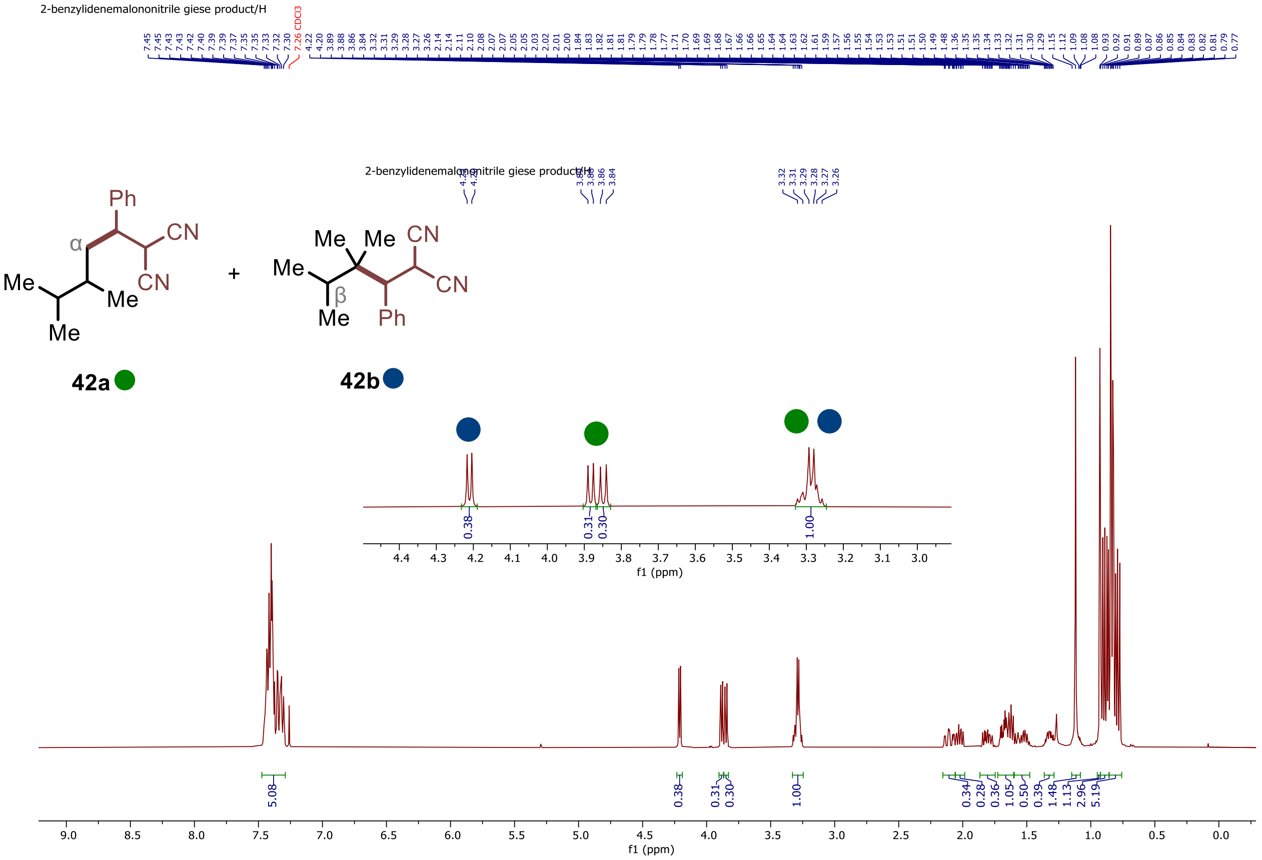

^{13}C NMR (101 MHz, $CDCl_3$) of **42**

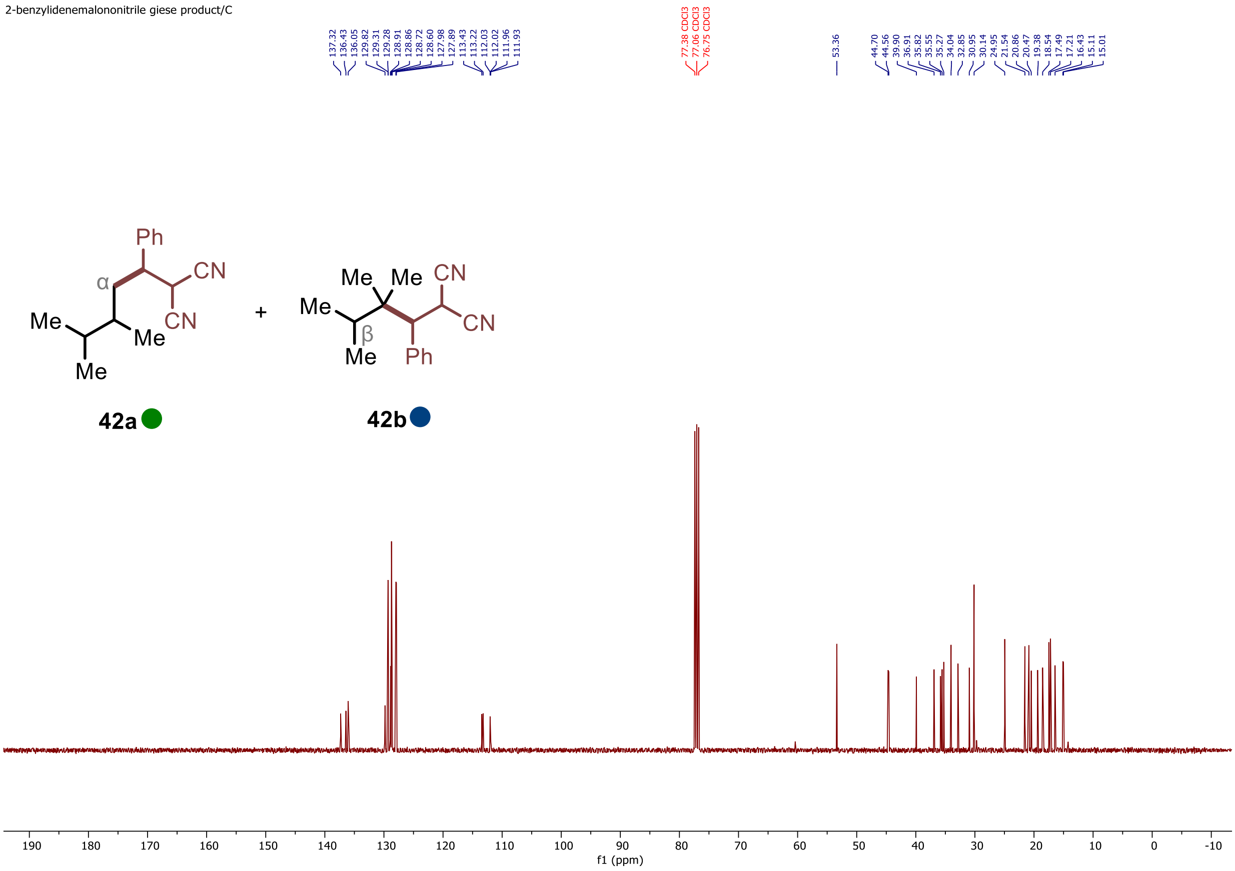

^{1}H NMR (400 MHz, $CDCl_3$) of **S1** (*see procedure*)

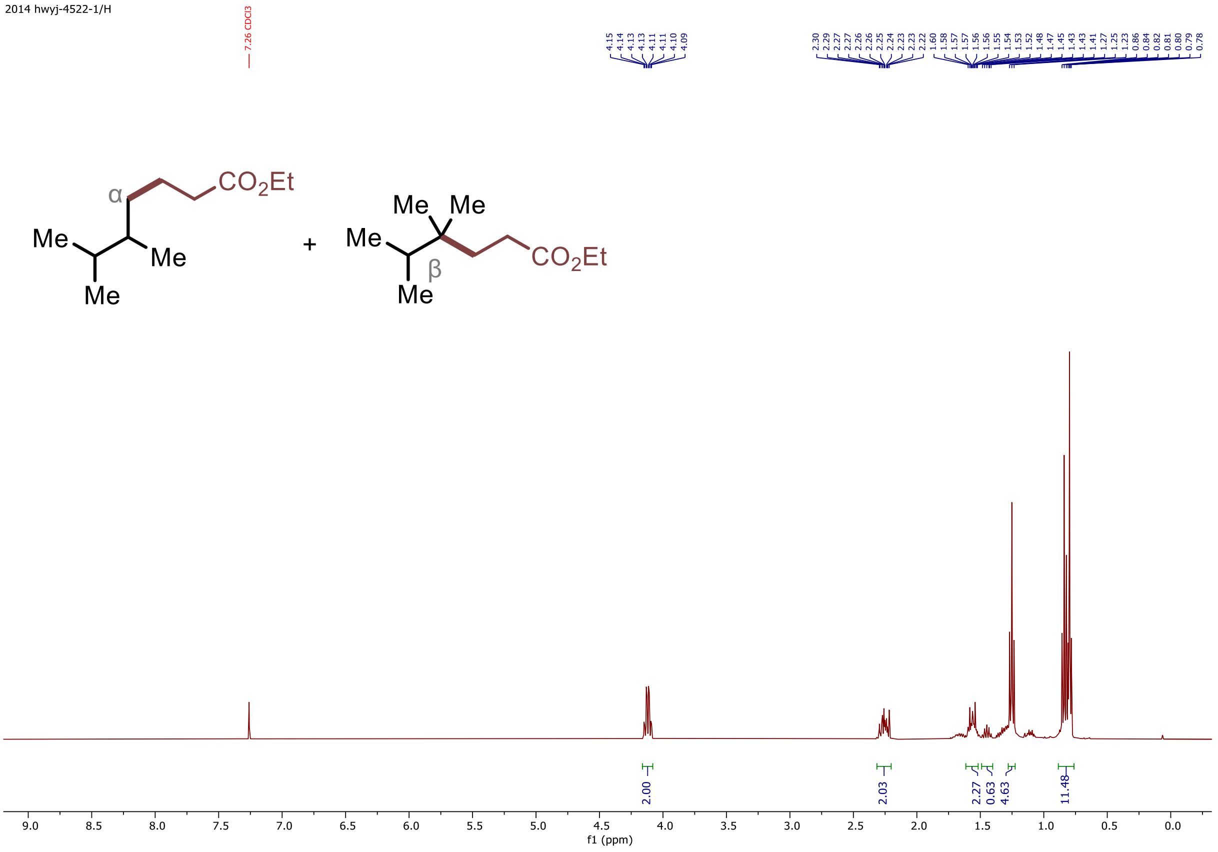

^{13}C NMR (101 MHz, $CDCl_3$) of **S1**

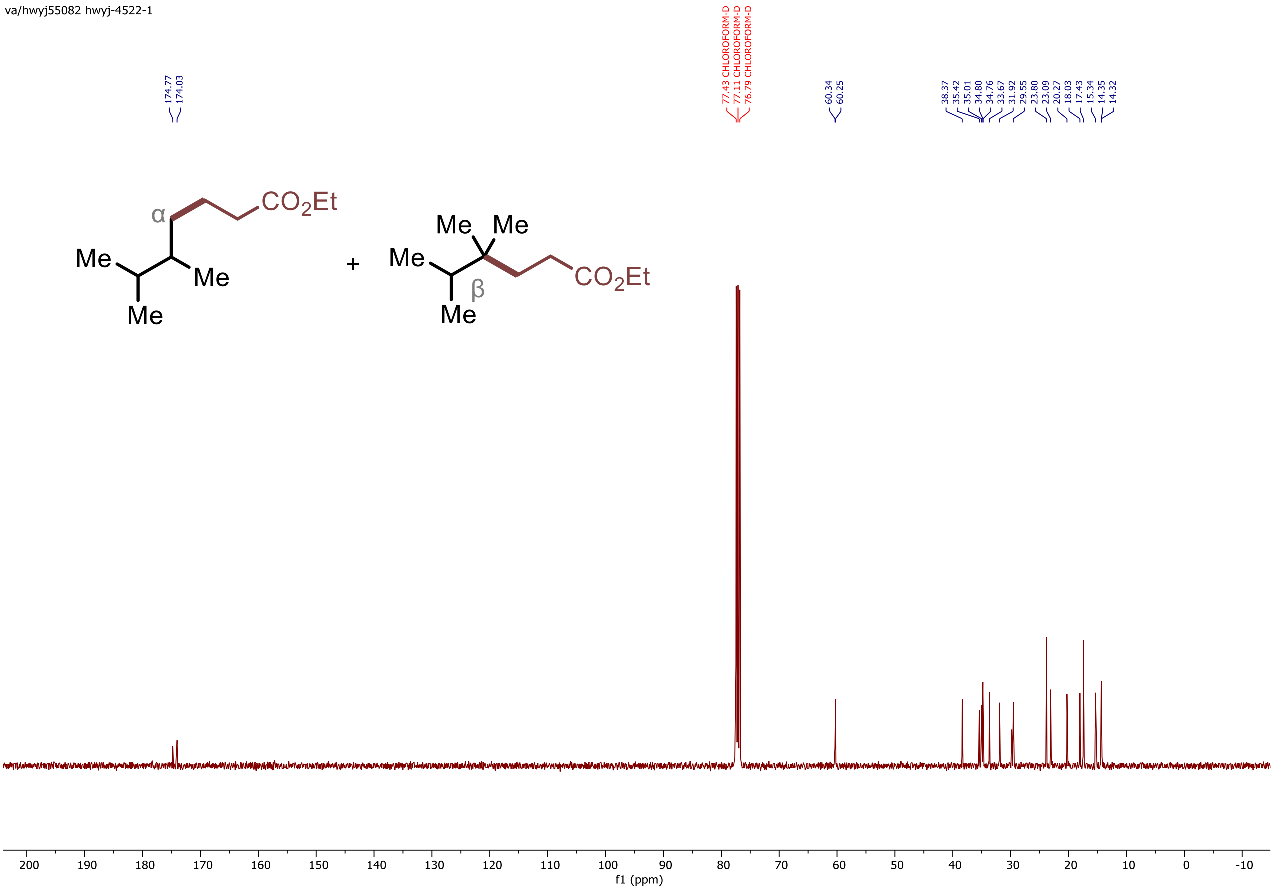

6. REFERENCES

1) Steinmetz, H.; Li, J.; Fu, C.; Zaburannyi, N.; Kunze, B.; Harmrolfs, K.; Schmitt, V.; Herrmann, J.; Reichenbach, H.; Höfle, G.; Kalesse, M.; Müller, R. Isolation, Structure Elucidation, and (Bio) Synthesis of Haprolid, a Cell-Type-Specific Myxobacterial Cytotoxin. *Angew. Chem., Int. Ed.* **2016**, *55,* 10113–10117.

2) Sudhakar, G.; Bayya, S.; Reddy, K.J.; Sridhar, B.; Sharma, K.; Bathula, S. R. Synthesis and Cytotoxicity of the Proposed Structure of Piperazirum, Its Stereoisomers and Analogues. *Eur. J. Org. Chem.* **2014**, *6*, 1253–1265.

3) Juhl, M.; Petersen, A. R.; Lee, J.-W. CO_2-Enabled Cyanohydrin Synthesis and Facile Iterative Homologation Reactions. *Chem. Eur. J.* **2021**, *27*, 228–232.

4) Lin, Z.-C.; Chen, C. Asymmetric Synthesis of a New Salen Type-titanium Complex as the Catalyst for Asymmetric Trimethylsilylcyanation of Aldehydes. *J. Chin. Chem. Soc.* **2010**, *57*, 726–737.

5) Pagire, S. K.; Shu, C.; Reich, D.; Noble, A. Aggarwal, V. K. Convergent Deboronative and Decarboxylative Phosphonylation Enabled by the Phosphite Radical Trap "BecaP". *J. Am. Chem. Soc.* **2023**, *145*, 18649–18657.

6) Zhang, H.; Zheng, X.; Xie, N.; He, Z.; Liu, J.; Leung, N. L. C.; Niu, Y.; Huang, X.; Wong, K. S.; Kwok, R. T. K.; Sung, H. H. Y.; Williams, I. D.; Qin, A.; Lam, J. W. Y.; Tang, B. Z. Why Do Simple Molecules with "Isolated" Phenyl Rings Emit Visible Light? *J. Am. Chem. Soc.* **2017**, *139*, 16264–16272.

7) Chai, L.; Wang, J.; Yang, J.; Yin, J. Zhang, Z.; Cheng, Y.; Zhu, L.; Xue, X.-S.; Li, C. Radical Arbuzov Reaction. *CCS Chem.* **2023**, *6*, 1312–1323.

8) Antczak, M. I.; Montchamp, J.-L. Mild Synthesis of Organophosphorus Compounds: Reaction of Phosphorus-Containing Carbenoids with Organoboranes. *Org. Lett.* **2008**, *10*, 977-980.

9) Dai, Z.; Zhang, S.; Hong, X.; Wang, P.; Gong, L. A Practical $FeCl_3$/HCl Photocatalyst for Versatile Aliphatic C–H Functionalization. *Chem Catal.* **2022**, *2*, 1211–1222.

10) Carré, V.; Godard, P.; Méreau, R.; Jacquot de Rouville, H.-P.; Jonusauskas, G.; McClenaghan, N.; Tassaing, T.; Vincent, J.-M. Photogeneration of Chlorine Radical from a Self-Assembled Fluorous 4CzIPN•Chloride Complex: Application in C−H Bond Functionalization. *Angew. Chem., Int. Ed.* **2024**, *63*, e202402964.

11) Treacy, S. M.; Rovis, T. Copper Catalyzed C(sp^3)–H Bond Alkylation via Photoinduced Ligand-to-Metal Charge Transfer. *J. Am. Chem. Soc.* **2021**, *143,* 2729–2735.

12) Sang, R.; Han, W.; Zhang, H.; Noble, A.; Aggarwal, V. K. Copper-Mediated Dehydrogenative C(sp^3)–H Borylation of Alkanes. *J. Am. Chem. Soc.* **2023**, *145*, 15207−15217.
